# Supplementary material for: The Origin and Evolution of Release Factors: Implications for Translation Termination, Ribosome Rescue, and Quality Control Pathways
Source: Int J Mol Sci. 2019 Apr 23;20(8):1981. doi: 10.3390/ijms20081981 (PMC6514570; doi:10.3390/ijms20081981)
Supplement: Supplementary file 1 [file Supplementary_Material.pdf › Supplementary_Material.pdf]

The origin and evolution of release factors: implications for translation termination, ribosome rescue and quality control pathways

A. Maxwell Burroughs[1] and L Aravind[1]

SUPPLEMENTARY MATERIAL

[1] Computational Biology Branch, National Center for Biotechnology Information, National Library of Medicine, National Institutes of Health, Bethesda, MD 20894, USA

Table of Contents

I. Supplementary Figure Legends.
II. Supplementary Figures
III. Supplementary Data

Supplementary Figure S1. Length diversity of catalytic loop in aeRF-1 families. Families are labeled on the *y*-axis and length of catalytic loops are plotted by total amino acid number on the *x*-axis. Sequences within a family corresponding to a specific length are depicted as a circle centered at that length, with circle radius scaled by the number of sequences in the family with that length, normalized by the total sequence count for the family. Loop sequence lengths which are unique (only a single sequence is observed at that length) within a family are not assigned circles. Coloring of circles within a family is based on an automatically-generated rainbow spectrum with red coloring for smaller numbers of sequences and violet coloring for larger numbers of sequences. Black lines depict the range of loop lengths across a family. Blue vertical bars in each family provide the median length in a family. Dotted blue vertical line indicates the median for all loop lengths across all plotted families.

Supplementary Figure S2. Positional entropy values for baeRF-1 families (right column) compared to the classical aeRF-1 families (left column). Mean entropy values within a family are plotted as blue horizontal lines and secondary structures are provided at bottom of plots. Catalytic loop region is labeled and shown as pink line in the secondary structure. Entropy was calculated using the Shannon entropy formula $-\sum_{i=1}^{M} P_i \log 2 P_i$ where P_i is the fraction of a given amino acid *i* and *M* is the total number of different amino acids.

Supplementary Figure S1.

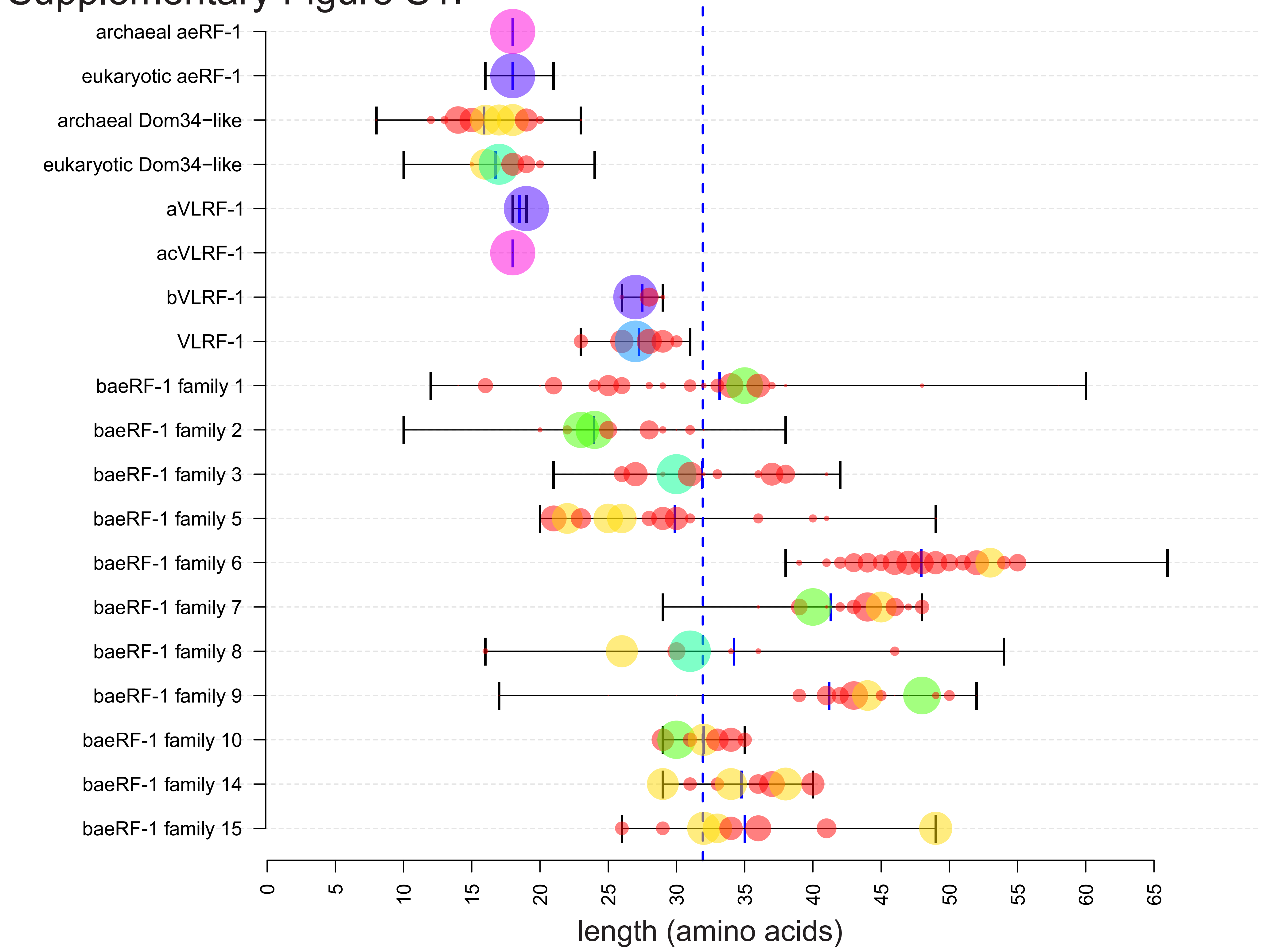

Supplementary Figure S2.

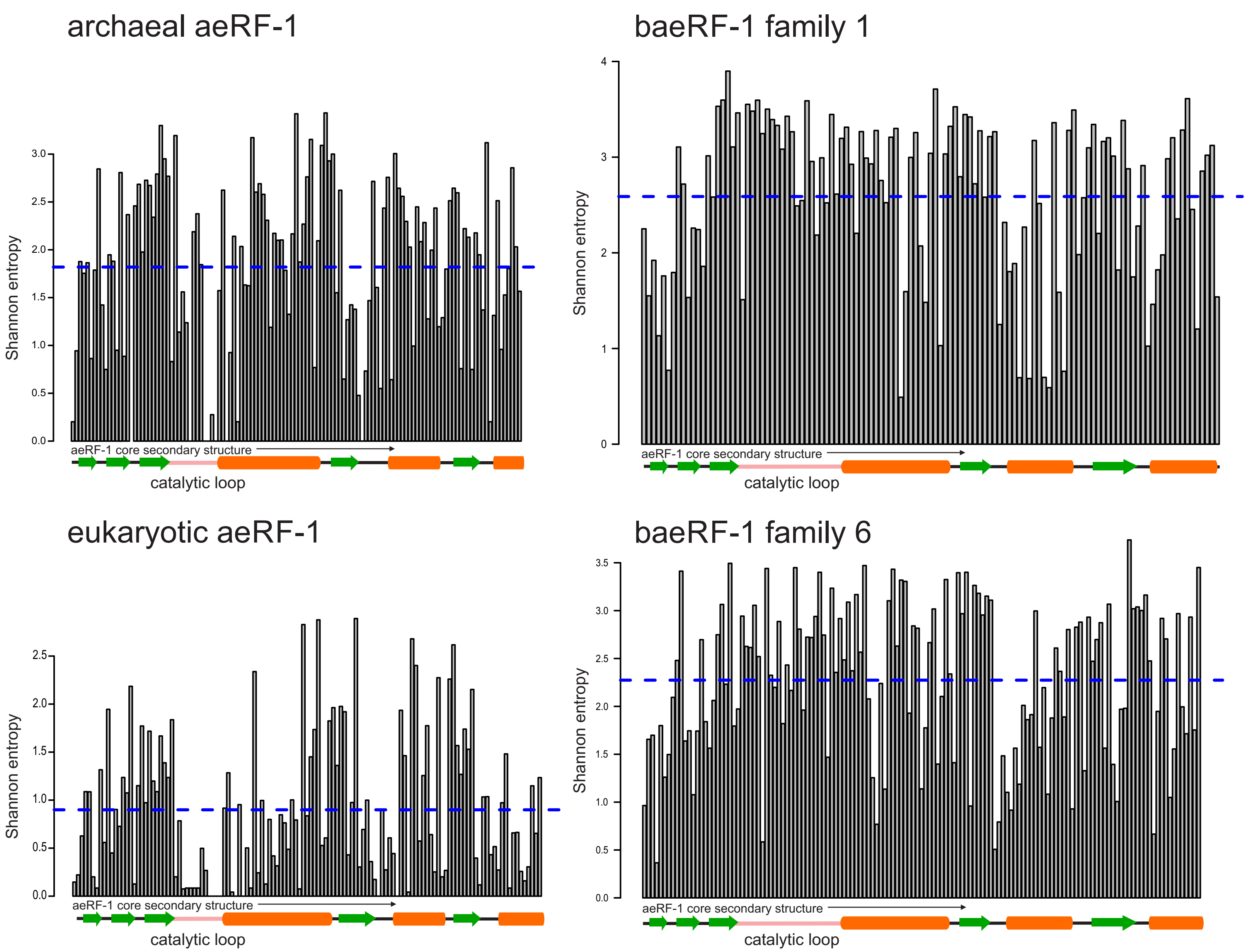

SUPPLEMENTARY DATA

The origin and evolution of release factors: implications for translation termination, ribosome rescue and quality control pathways

A. Maxwell Burroughs, L Aravind

National Center for Biotechnology Information, National Library of Medicine, National Institutes of Health, Bethesda, MD, USA

ABSTRACT

The evolution of the release factor-peptidyl hydrolases (RF-PHs), which catalyze the breakage of the final tRNA-peptide bond during translation termination, is a longstanding evolutionary paradox. While the components of the translation apparatus are generally well-conserved across extant life, the bacterial and archaeo-eukaryotic RF-PHs emerged from distinct protein folds at the base of their respective lineages. Recent computational surveys and targeted experimental work have uncovered previously-undocumented diversity in both RF-PH domains, notably the report of the long-missing Vms1/ANKZF1 release factor in the eukaryotic quality control pathway.

This recent work is distilled into an accounting of the diversification of these RF-PH domains, and this is in turn placed into the broader evolutionary framework of the evolution of the translation apparatus. We review the deep evolutionary scenarios for the origins of these RF-PH domains in light of this new information and reconstruct the likely state of translation termination in the Last Universal Common Ancestor (LUCA) of Life. Further, we trace the evolutionary trajectories of the several offshoots involving release and the ribosome, including pathways activated in response to ribosomal stalling such as ribosome quality control and ribosome rescue. Multiple solutions to this rescue problem have emerged in the three superkingdoms of Life and several of these solutions were subsequently transferred across superkingdoms. We report the first global survey of one such solution, prototyped by the recently-characterized ArfT rescue factor. The eukaryotic ribosome quality control pathway is observed here as pieced together from components with disparate evolutionary histories. Finally, we report on an under-appreciated aspect that appears to have frequently driven innovation in rescue pathways: biological conflict with genic entities likely to seeking to disable translation directly at the ribosome. At least three rescue pathways (centered on the RFH, baeRF-1, and c12orf65 RF-PH domains), were likely innovated in response to such conflict.

TABLE OF CONTENTS

I. List of sequences for novel archaeo-eukaryotic release factors
    A. baeRF1 clade
        1. family 1
        2. family 2
            a. 2 beta-strand PELOTA insert
            b. zinc ribbon PELOTA insert
        3. family 3
        4. family 5
        5. family 6
        6. family 7
        7. family 8
        8. family 9
        9. family 10
        10. family 11
        11. family 12
        12. family 13
        13. family 14
        14. family 15
    B. Vms1-like aeRF1 (VLRF1) clade
        1. archaeal VLRF1 (aVLRF1) family
        2. actinobacterial/chloroflexi VLRF1 (acVLRF1) family
        3. bacteroidetes VLRF1 (bVLFR1) family
        4. eukaryotic VLRF1 family
            a. canonical Vms1-like architecture and variants
            b. other architectures
II. baeRF1 gene neighborhoods linking release factor to ribosome hibernation factors (YfiA/HPF-like domain)
III. Other lists of sequences
    A. Newly-defined ArfT-like family of ribosome rescue factors
    B. MRPS35 bRF-PH family
    C. eukaryotic SmpB sequences
IV. Other gene neighborhoods of interest
    A. Coupling of ArfB ribosome rescue factor with rRNA pseudouridine synthase
    B. RFH neighborhoods
V. Alignments of interest
    A. ArfT core consisting of several ArfT helix repeats
    B. MRPS28

DATA

I. List of sequences for novel archaeo-eukaryotic release factors

A. baeRF1 clade

1. family 1

##;family 1 is a standalone baeRF1, there are no N- or C-terminal fusions

EPX81431.1	Salmuc_05097	152	bacteria>proteobacteria>alphaproteobacteria	Salipiger mucosus DSM 16094	hypothetical protein Salmuc_05097 [Salipiger mucosus DSM 16094].
WP_057819397.1	-	152	bacteria>proteobacteria>alphaproteobacteria	Roseovarius indicus	host attachment protein [Roseovarius indicus].
KZY35075.1	A3731_02640	152	bacteria>proteobacteria>alphaproteobacteria	Roseovarius sp. HI0049	Host attachment protein [Roseovarius sp. HI0049].
WP_064259332.1	-	152	bacteria>proteobacteria>alphaproteobacteria	Roseovarius indicus	Host attachment protein [Roseovarius indicus].
WP_007120166.1	-	151	bacteria>proteobacteria>alphaproteobacteria	Oceanibulbus indolifex	protein required for attachment to host cells-like protein [Oceanibulbus indolifex].
WP_008328302.1	-	151	bacteria>proteobacteria>alphaproteobacteria	Maritimibacter alkaliphilus	hypothetical protein [Maritimibacter alkaliphilus].
WP_008557062.1	-	151	bacteria>proteobacteria>alphaproteobacteria	Leisingera daeponensis	host cell attachment protein [Leisingera daeponensis].
WP_019295433.1	-	151	bacteria>proteobacteria>alphaproteobacteria	Leisingera	MULTISPECIES: host attachment protein [Leisingera].
WP_027237902.1	-	151	bacteria>proteobacteria>alphaproteobacteria	Leisingera caerulea	host attachment protein [Leisingera caerulea].
WP_039169878.1	-	151	bacteria>proteobacteria>alphaproteobacteria	Leisingera sp. ANG-S5	host attachment protein [Leisingera sp. ANG-S5].
WP_043768875.1	-	151	bacteria>proteobacteria>alphaproteobacteria	Roseivivax isoporae	host attachment protein [Roseivivax isoporae].
WP_043841247.1	-	151	bacteria>proteobacteria>alphaproteobacteria	Roseivivax atlanticus	host attachment protein [Roseivivax atlanticus].
WP_058287111.1	-	151	bacteria>proteobacteria>alphaproteobacteria	Leisingera aquaemixtae	host attachment protein [Leisingera aquaemixtae].
KZX90745.1	A3720_09600	151	bacteria>proteobacteria>alphaproteobacteria	Oceanibulbus sp. HI0021	Host attachment protein [Oceanibulbus sp. HI0021].
KZY50889.1	A3734_07150	151	bacteria>proteobacteria>alphaproteobacteria	Sulfitobacter sp. HI0054	Host attachment protein [Sulfitobacter sp. HI0054].
KZZ21175.1	A3753_07200	151	bacteria>proteobacteria>alphaproteobacteria	Oceanibulbus sp. HI0082	Host attachment protein [Oceanibulbus sp. HI0082].
WP_065267030.1	-	151	bacteria>proteobacteria>alphaproteobacteria	Leisingera sp. JC1	Host attachment protein [Leisingera sp. JC1].
WP_037262758.1	-	149	bacteria>proteobacteria>alphaproteobacteria	Roseivivax halodurans	host attachment protein [Roseivivax halodurans].
AHM05160.1	roselon_02862	170	bacteria>proteobacteria>alphaproteobacteria	Roseibacterium elongatum DSM 19469	hypothetical protein roselon_02862 [Roseibacterium elongatum DSM 19469].
OAN81175.1	A8B78_09190	169	bacteria>proteobacteria>alphaproteobacteria	Jannaschia sp. EhC01	Host attachment protein [Jannaschia sp. EhC01].
WP_052453245.1	-	159	bacteria>proteobacteria>alphaproteobacteria	Celeribacter indicus	host attachment protein [Celeribacter indicus].
WP_055663293.1	-	157	bacteria>proteobacteria>alphaproteobacteria	Jannaschia seosinensis	host attachment protein [Jannaschia seosinensis].
WP_007426159.1	-	156	bacteria>proteobacteria>alphaproteobacteria	Oceaniovalibus guishaninsula	hypothetical protein [Oceaniovalibus guishaninsula].
WP_043919047.1	-	155	bacteria>proteobacteria>alphaproteobacteria	Jannaschia aquimarina	host attachment protein [Jannaschia aquimarina].
WP_055683450.1	-	155	bacteria>proteobacteria>alphaproteobacteria	Jannaschia rubra	host attachment protein [Jannaschia rubra].
WP_010138830.1	-	154	bacteria>proteobacteria>alphaproteobacteria	Oceanicola sp. S124	hypothetical protein [Oceanicola sp. S124].
WP_024092032.1	-	154	bacteria>proteobacteria>alphaproteobacteria	Leisingera methylohalidivorans	host attachment protein [Leisingera methylohalidivorans].
WP_018303484.1	-	152	bacteria>proteobacteria>alphaproteobacteria	Wenxinia marina	hypothetical protein [Wenxinia marina].
WP_022704983.1	-	152	bacteria>proteobacteria>alphaproteobacteria	Pseudorhodobacter ferrugineus	host attachment protein [Pseudorhodobacter ferrugineus].
WP_050525729.1	-	152	bacteria>proteobacteria>alphaproteobacteria	Pseudorhodobacter aquimaris	host attachment protein [Pseudorhodobacter aquimaris].
WP_050662441.1	-	152	bacteria>proteobacteria>alphaproteobacteria	Roseovarius tolerans	host attachment protein [Roseovarius tolerans].
WP_055086336.1	-	152	bacteria>proteobacteria>alphaproteobacteria	Jannaschia donghaensis	host attachment protein [Jannaschia donghaensis].
WP_005852614.1	-	151	bacteria>proteobacteria>alphaproteobacteria	Sulfitobacter sp. EE-36	hypothetical protein [Sulfitobacter sp. EE-36].
WP_005854861.1	-	151	bacteria>proteobacteria>alphaproteobacteria	Sagittula stellata	hypothetical protein [Sagittula stellata].
WP_007253734.1	-	151	bacteria>proteobacteria>alphaproteobacteria	Oceanicola granulosus	hypothetical protein [Oceanicola granulosus].
WP_007792549.1	-	151	bacteria>proteobacteria>alphaproteobacteria	Pelagibaca bermudensis	hypothetical protein [Pelagibaca bermudensis].
WP_009812361.1	-	151	bacteria>proteobacteria>alphaproteobacteria	Roseovarius nubinhibens	hypothetical protein [Roseovarius nubinhibens].
WP_009826642.1	-	151	bacteria>proteobacteria>alphaproteobacteria	Sulfitobacter sp. NAS-14.1	hypothetical protein [Sulfitobacter sp. NAS-14.1].
WP_022706183.1	-	151	bacteria>proteobacteria>alphaproteobacteria	Paracoccus zeaxanthinifaciens	host attachment protein [Paracoccus zeaxanthinifaciens].
WP_023851027.1	-	151	bacteria>proteobacteria>alphaproteobacteria	Rhodobacteraceae bacterium PD-2	host attachment protein [Rhodobacteraceae bacterium PD-2].
WP_025048697.1	-	151	bacteria>proteobacteria>alphaproteobacteria	Sulfitobacter mediterraneus	host attachment protein [Sulfitobacter mediterraneus].
WP_025052545.1	-	151	bacteria>proteobacteria>alphaproteobacteria	Sulfitobacter noctilucae	host attachment protein [Sulfitobacter noctilucae].
WP_028288322.1	-	151	bacteria>proteobacteria>alphaproteobacteria	Pseudooceanicola nanhaiensis	host attachment protein [Pseudooceanicola nanhaiensis].
WP_037910668.1	-	151	bacteria>proteobacteria>alphaproteobacteria	Sulfitobacter mediterraneus	host attachment protein [Sulfitobacter mediterraneus].
WP_037943926.1	-	151	bacteria>proteobacteria>alphaproteobacteria	Sulfitobacter sp. CB2047	host attachment protein [Sulfitobacter sp. CB2047].
WP_037962902.1	-	151	bacteria>proteobacteria>alphaproteobacteria	Sulfitobacter pontiacus	host attachment protein [Sulfitobacter pontiacus].
WP_038063128.1	-	151	bacteria>proteobacteria>alphaproteobacteria	Thioclava dalianensis	host attachment protein [Thioclava dalianensis].
WP_038127357.1	-	151	bacteria>proteobacteria>alphaproteobacteria	Thioclava indica	host attachment protein [Thioclava indica].
WP_038143176.1	-	151	bacteria>proteobacteria>alphaproteobacteria	Thioclava atlantica	host attachment protein [Thioclava atlantica].
WP_048529629.1	-	151	bacteria>proteobacteria>alphaproteobacteria	Marinovum algicola	host attachment protein [Marinovum algicola].
WP_050532343.1	-	151	bacteria>proteobacteria>alphaproteobacteria	Aestuariivita atlantica	host attachment protein [Aestuariivita atlantica].
WP_057795025.1	-	151	bacteria>proteobacteria>alphaproteobacteria	Roseovarius atlanticus	host attachment protein [Roseovarius atlanticus].
WP_058860447.1	-	151	bacteria>proteobacteria>alphaproteobacteria	Ponticoccus sp. SJ5A-1	host attachment protein [Ponticoccus sp. SJ5A-1].
ANT59195.1	AYJ57_01765	151	bacteria>proteobacteria>alphaproteobacteria	Yangia sp. CCB-MM3	Host attachment protein [Yangia sp. CCB-MM3].
WP_005620169.1	-	150	bacteria>proteobacteria>alphaproteobacteria	Ruegeria	MULTISPECIES: hypothetical protein [Ruegeria].
WP_008561319.1	-	150	bacteria>proteobacteria>alphaproteobacteria	Rhodobacteraceae	MULTISPECIES: host cell attachment protein [Rhodobacteraceae].
WP_014874930.1	-	150	bacteria>proteobacteria>alphaproteobacteria	Phaeobacter	MULTISPECIES: hypothetical protein [Phaeobacter].
WP_017928829.1	-	150	bacteria>proteobacteria>alphaproteobacteria	Loktanella hongkongensis	hypothetical protein [Loktanella hongkongensis].
WP_021693054.1	-	150	bacteria>proteobacteria>alphaproteobacteria	Loktanella cinnabarina	hypothetical protein [Loktanella cinnabarina].
WP_024096933.1	-	150	bacteria>proteobacteria>alphaproteobacteria	Phaeobacter	MULTISPECIES: protein required for attachment to host cell [Phaeobacter].
WP_036181991.1	-	150	bacteria>proteobacteria>alphaproteobacteria	Maribius sp. MOLA 401	host attachment protein [Maribius sp. MOLA 401].
WP_040817308.1	-	150	bacteria>proteobacteria>alphaproteobacteria	Litoreibacter arenae	host attachment protein [Litoreibacter arenae].
WP_056031768.1	-	150	bacteria>proteobacteria>alphaproteobacteria	Loktanella sp. 3ANDIMAR09	host attachment protein [Loktanella sp. 3ANDIMAR09].
KUP91210.1	TRIHO_39460	150	bacteria>proteobacteria>alphaproteobacteria	Rhodobacteraceae bacterium O3.65	protein required for attachment to host cells [Rhodobacteraceae bacterium O3.65].
WP_061047762.1	-	150	bacteria>proteobacteria>alphaproteobacteria	Phaeobacter inhibens	Host attachment protein [Phaeobacter inhibens].
OAN75232.1	A8B82_16760	150	bacteria>proteobacteria>alphaproteobacteria	Sulfitobacter sp. EhC04	Host attachment protein [Sulfitobacter sp. EhC04].
WP_065323219.1	-	150	bacteria>proteobacteria>alphaproteobacteria	Ruegeria mobilis	Host attachment protein [Ruegeria mobilis].
WP_008833776.1	-	149	bacteria>proteobacteria>alphaproteobacteria	Mesorhizobium alhagi	host attachment protein [Mesorhizobium alhagi].
WP_009805180.1	-	149	bacteria>proteobacteria>alphaproteobacteria	Pseudooceanicola batsensis	hypothetical protein [Pseudooceanicola batsensis].
KZY03383.1	A3721_03500	149	bacteria>proteobacteria>alphaproteobacteria	Oceanibulbus sp. HI0023	Host attachment protein [Oceanibulbus sp. HI0023].
EPX79279.1	thalar_02104	139	bacteria>proteobacteria>alphaproteobacteria	Litoreibacter arenae DSM 19593	hypothetical protein thalar_02104 [Litoreibacter arenae DSM 19593].
WP_052726625.1	-	212	bacteria>proteobacteria>alphaproteobacteria	Devosia epidermidihirudinis	hypothetical protein [Devosia epidermidihirudinis].
WP_046169242.1	-	174	bacteria>proteobacteria>alphaproteobacteria	Devosia psychrophila	hypothetical protein [Devosia psychrophila].
WP_007813681.1	-	151	bacteria>proteobacteria>alphaproteobacteria	Roseobacter sp. AzwK-3b	hypothetical protein [Roseobacter sp. AzwK-3b].
WP_026757743.1	-	151	bacteria>proteobacteria>alphaproteobacteria	Sediminimonas qiaohouensis	host attachment protein [Sediminimonas qiaohouensis].
WP_039146387.1	-	151	bacteria>proteobacteria>alphaproteobacteria	Leisingera	MULTISPECIES: host attachment protein [Leisingera].
OAN68435.1	A8B83_03265	151	bacteria>proteobacteria>alphaproteobacteria	Rhodobacteraceae bacterium EhC02	Host attachment protein [Rhodobacteraceae bacterium EhC02].
WP_051213523.1	-	149	bacteria>proteobacteria>alphaproteobacteria	Maritalea myrionectae	hypothetical protein [Maritalea myrionectae].
WP_008028955.1	-	141	bacteria>proteobacteria>alphaproteobacteria	Rhodobacter sp. SW2	hypothetical protein [Rhodobacter sp. SW2].
WP_020698430.1	-	167	bacteria>proteobacteria>alphaproteobacteria	Reyranella massiliensis	hypothetical protein [Reyranella massiliensis].
EES53958.1	UBAL3_44810095	160	bacteria>nitrospirae	Leptospirillum ferrodiazotrophum	protein of unknown function [Leptospirillum ferrodiazotrophum].
KPQ04953.1	HLUCCA12_16005	159	bacteria>proteobacteria>alphaproteobacteria	Rhodobacteraceae bacterium HLUCCA12	hypothetical protein HLUCCA12_16005 [Rhodobacteraceae bacterium HLUCCA12].
WP_052951534.1	-	154	bacteria>proteobacteria>alphaproteobacteria	Devosia soli	hypothetical protein [Devosia soli].
WP_009799216.1	-	151	bacteria>proteobacteria>alphaproteobacteria	Nitrobacter sp. Nb-311A	hypothetical protein [Nitrobacter sp. Nb-311A].
WP_011510417.1	-	151	bacteria>proteobacteria>alphaproteobacteria	Nitrobacter hamburgensis	hypothetical protein [Nitrobacter hamburgensis].
WP_012332696.1	-	151	bacteria>proteobacteria>alphaproteobacteria	Methylobacterium sp. 4-46	hypothetical protein [Methylobacterium sp. 4-46].
WP_026190615.1	-	151	bacteria>proteobacteria>alphaproteobacteria	Methylobacterium sp. WSM2598	host cell attachment protein [Methylobacterium sp. WSM2598].
WP_035030328.1	-	149	bacteria>proteobacteria>alphaproteobacteria	Devosia	MULTISPECIES: hypothetical protein [Devosia].
WP_045680226.1	-	149	bacteria>proteobacteria>alphaproteobacteria	Martelella endophytica	hypothetical protein [Martelella endophytica].
WP_049705927.1	-	149	bacteria>proteobacteria>alphaproteobacteria	Devosia sp. H5989	hypothetical protein [Devosia sp. H5989].
WP_056307056.1	-	149	bacteria>proteobacteria>alphaproteobacteria	Devosia sp. Root685	hypothetical protein [Devosia sp. Root685].
WP_061910943.1	-	149	bacteria>proteobacteria>alphaproteobacteria	Devosia sp. Leaf420	hypothetical protein [Devosia sp. Leaf420].
WP_062631745.1	-	149	bacteria>proteobacteria>alphaproteobacteria	Devosia sp. Leaf64	hypothetical protein [Devosia sp. Leaf64].
WP_019216024.1	-	148	bacteria>proteobacteria>gammaproteobacteria	Legionella tunisiensis	hypothetical protein [Legionella tunisiensis].
WP_020175792.1	-	148	bacteria>proteobacteria>alphaproteobacteria	Methyloferula stellata	hypothetical protein [Methyloferula stellata].
WP_048576341.1	-	146	bacteria>proteobacteria>alphaproteobacteria	Sphingomonas sp. MEA3-1	hypothetical protein [Sphingomonas sp. MEA3-1].
WP_051520110.1	-	146	bacteria>proteobacteria>alphaproteobacteria	Sphingobium sp. Ant17	hypothetical protein [Sphingobium sp. Ant17].
WP_058470858.1	-	146	bacteria>proteobacteria>gammaproteobacteria	Legionella jordanis	hypothetical protein [Legionella jordanis].
AMK22907.1	K426_09810	146	bacteria>proteobacteria>alphaproteobacteria	Sphingobium sp. TKS	hypothetical protein K426_09810 [Sphingobium sp. TKS].
WP_056601589.1	-	145	bacteria>proteobacteria>alphaproteobacteria	Sphingomonas sp. Root50	hypothetical protein [Sphingomonas sp. Root50].
WP_046104710.1	-	144	bacteria>proteobacteria>alphaproteobacteria	Devosia chinhatensis	hypothetical protein [Devosia chinhatensis].
WP_035720012.1	-	142	bacteria>proteobacteria>gammaproteobacteria	Francisella sp. W12-1067	hypothetical protein [Francisella sp. W12-1067].
WP_039125577.1	-	142	bacteria>proteobacteria>gammaproteobacteria	Francisella guangzhouensis	hypothetical protein [Francisella guangzhouensis].
WP_014449401.1	-	105	bacteria>nitrospirae	Leptospirillum ferrooxidans	hypothetical protein [Leptospirillum ferrooxidans].
WP_017464142.1	-	147	bacteria>proteobacteria>gammaproteobacteria	Dyella ginsengisoli	hypothetical protein [Dyella ginsengisoli].
KPK08788.1	AMJ64_02345	147	bacteria>proteobacteria>betaproteobacteria	Betaproteobacteria bacterium SG8_39	hypothetical protein AMJ64_02345 [Betaproteobacteria bacterium SG8_39].
WP_063671004.1	-	147	bacteria>proteobacteria>gammaproteobacteria	Dyella thiooxydans	hypothetical protein [Dyella thiooxydans].
WP_028462252.1	-	145	bacteria>proteobacteria>betaproteobacteria	Nitrosomonas cryotolerans	hypothetical protein [Nitrosomonas cryotolerans].
WP_062559201.1	-	145	bacteria>proteobacteria>betaproteobacteria	Nitrosomonas ureae	hypothetical protein [Nitrosomonas ureae].
WP_054117356.1	-	135	bacteria>proteobacteria>alphaproteobacteria	Porphyrobacter sp. AAP60	hypothetical protein [Porphyrobacter sp. AAP60].
KPJ79289.1	AMJ58_12530	144	bacteria>proteobacteria>gammaproteobacteria	Gammaproteobacteria bacterium SG8_30	hypothetical protein AMJ58_12530 [Gammaproteobacteria bacterium SG8_30].
WP_008261598.1	-	159	bacteria>proteobacteria>alphaproteobacteria	Brevundimonas sp. BAL3	hypothetical protein [Brevundimonas sp. BAL3].
WP_035306598.1	-	159	bacteria>proteobacteria>alphaproteobacteria	Brevundimonas sp. EAKA	hypothetical protein [Brevundimonas sp. EAKA].
WP_012521203.1	-	149	bacteria>proteobacteria>alphaproteobacteria	Phenylobacterium zucineum	hypothetical protein [Phenylobacterium zucineum].
WP_049450882.1	-	143	bacteria>proteobacteria>gammaproteobacteria	Stenotrophomonas maltophilia	attachment protein [Stenotrophomonas maltophilia].
WP_038692204.1	-	140	bacteria>proteobacteria>gammaproteobacteria	Stenotrophomonas rhizophila	attachment protein [Stenotrophomonas rhizophila].
WP_042613567.1	-	139	bacteria>proteobacteria>gammaproteobacteria	Stenotrophomonas maltophilia	attachment protein [Stenotrophomonas maltophilia].
WP_057507665.1	-	139	bacteria>proteobacteria>gammaproteobacteria	Stenotrophomonas chelatiphaga	attachment protein [Stenotrophomonas chelatiphaga].
AOA72559.1	BAY15_2125	139	bacteria>proteobacteria>gammaproteobacteria	Stenotrophomonas rhizophila	attachment protein [Stenotrophomonas rhizophila].
CCP11899.1	SMSKK35_4121	138	bacteria>proteobacteria>gammaproteobacteria	Stenotrophomonas maltophilia SKK35	AtsE [Stenotrophomonas maltophilia SKK35].
WP_005409854.1	-	138	bacteria>proteobacteria>gammaproteobacteria	Stenotrophomonas maltophilia	attachment protein [Stenotrophomonas maltophilia].
WP_005416741.1	-	138	bacteria>proteobacteria>gammaproteobacteria	Stenotrophomonas maltophilia	host attachment protein [Stenotrophomonas maltophilia].
WP_006370287.1	-	138	bacteria>proteobacteria>gammaproteobacteria	Stenotrophomonas maltophilia	host cell attachment protein [Stenotrophomonas maltophilia].
WP_008267365.1	-	138	bacteria>proteobacteria>gammaproteobacteria	Stenotrophomonas sp. SKA14	attachment protein [Stenotrophomonas sp. SKA14].
WP_012511222.1	-	138	bacteria>proteobacteria>gammaproteobacteria	Stenotrophomonas maltophilia	attachment protein [Stenotrophomonas maltophilia].
WP_014037377.1	-	138	bacteria>proteobacteria>gammaproteobacteria	Stenotrophomonas	MULTISPECIES: attachment protein [Stenotrophomonas].
WP_014647339.1	-	138	bacteria>proteobacteria>gammaproteobacteria	Stenotrophomonas maltophilia	attachment protein [Stenotrophomonas maltophilia].
WP_019337746.1	-	138	bacteria>proteobacteria>gammaproteobacteria	Stenotrophomonas maltophilia	attachment protein [Stenotrophomonas maltophilia].
WP_025874400.1	-	138	bacteria>proteobacteria>gammaproteobacteria	Stenotrophomonas maltophilia	attachment protein [Stenotrophomonas maltophilia].
WP_032962601.1	-	138	bacteria>proteobacteria>gammaproteobacteria	Stenotrophomonas maltophilia	attachment protein [Stenotrophomonas maltophilia].
WP_032974590.1	-	138	bacteria>proteobacteria>gammaproteobacteria	Stenotrophomonas	MULTISPECIES: attachment protein [Stenotrophomonas].
WP_043033333.1	-	138	bacteria>proteobacteria>gammaproteobacteria	Stenotrophomonas maltophilia	attachment protein [Stenotrophomonas maltophilia].
CRP76375.1	PAERUG_E15_London_28_01_14_00952	138	bacteria>proteobacteria>gammaproteobacteria	Pseudomonas aeruginosa	Protein required for attachment to host cells [Pseudomonas aeruginosa].
WP_049399748.1	-	138	bacteria>proteobacteria>gammaproteobacteria	Stenotrophomonas maltophilia	attachment protein [Stenotrophomonas maltophilia].
WP_049411560.1	-	138	bacteria>proteobacteria>gammaproteobacteria	Stenotrophomonas maltophilia	attachment protein [Stenotrophomonas maltophilia].
WP_049422968.1	-	138	bacteria>proteobacteria>gammaproteobacteria	Stenotrophomonas maltophilia	attachment protein [Stenotrophomonas maltophilia].
WP_049439519.1	-	138	bacteria>proteobacteria>gammaproteobacteria	Stenotrophomonas maltophilia	attachment protein [Stenotrophomonas maltophilia].
WP_049448044.1	-	138	bacteria>proteobacteria>gammaproteobacteria	Stenotrophomonas maltophilia	attachment protein [Stenotrophomonas maltophilia].
WP_049455130.1	-	138	bacteria>proteobacteria>gammaproteobacteria	Stenotrophomonas maltophilia	attachment protein [Stenotrophomonas maltophilia].
WP_049457047.1	-	138	bacteria>proteobacteria>gammaproteobacteria	Stenotrophomonas maltophilia	attachment protein [Stenotrophomonas maltophilia].
WP_053449896.1	-	138	bacteria>proteobacteria>gammaproteobacteria	Stenotrophomonas maltophilia	attachment protein [Stenotrophomonas maltophilia].
CRX70035.1	BN2069_35040	138	bacteria>proteobacteria>gammaproteobacteria	Stenotrophomonas maltophilia	unnamed protein product [Stenotrophomonas maltophilia].
WP_053500442.1	-	138	bacteria>proteobacteria>gammaproteobacteria	Stenotrophomonas maltophilia	attachment protein [Stenotrophomonas maltophilia].
WP_053508815.1	-	138	bacteria>proteobacteria>gammaproteobacteria	Stenotrophomonas maltophilia	attachment protein [Stenotrophomonas maltophilia].
WP_053516277.1	-	138	bacteria>proteobacteria>gammaproteobacteria	Stenotrophomonas maltophilia	attachment protein [Stenotrophomonas maltophilia].
WP_054171871.1	-	138	bacteria>proteobacteria>gammaproteobacteria	Stenotrophomonas maltophilia	attachment protein [Stenotrophomonas maltophilia].
WP_057496082.1	-	138	bacteria>proteobacteria>gammaproteobacteria	Stenotrophomonas maltophilia	attachment protein [Stenotrophomonas maltophilia].
WP_057501570.1	-	138	bacteria>proteobacteria>gammaproteobacteria	Stenotrophomonas maltophilia	attachment protein [Stenotrophomonas maltophilia].
WP_058980568.1	-	138	bacteria>proteobacteria>gammaproteobacteria	Stenotrophomonas maltophilia	attachment protein [Stenotrophomonas maltophilia].
WP_061479426.1	-	138	bacteria>proteobacteria>gammaproteobacteria	Stenotrophomonas sp. DDT-1	attachment protein [Stenotrophomonas sp. DDT-1].
WP_064238671.1	-	138	bacteria>proteobacteria>gammaproteobacteria	Stenotrophomonas maltophilia	attachment protein [Stenotrophomonas maltophilia].
WP_065176180.1	-	138	bacteria>proteobacteria>gammaproteobacteria	Stenotrophomonas maltophilia	attachment protein [Stenotrophomonas maltophilia].
WP_065177692.1	-	138	bacteria>proteobacteria>gammaproteobacteria	Stenotrophomonas maltophilia	attachment protein [Stenotrophomonas maltophilia].
WP_065186507.1	-	138	bacteria>proteobacteria>gammaproteobacteria	Stenotrophomonas maltophilia	attachment protein [Stenotrophomonas maltophilia].
WP_065198467.1	-	138	bacteria>proteobacteria>gammaproteobacteria	Stenotrophomonas maltophilia	attachment protein [Stenotrophomonas maltophilia].
WP_065723418.1	-	138	bacteria>proteobacteria>gammaproteobacteria	Stenotrophomonas maltophilia	attachment protein [Stenotrophomonas maltophilia].
WP_017357372.1	-	137	bacteria>proteobacteria>gammaproteobacteria	Stenotrophomonas maltophilia	attachment protein [Stenotrophomonas maltophilia].
WP_019184575.1	-	137	bacteria>proteobacteria>gammaproteobacteria	Stenotrophomonas maltophilia	attachment protein [Stenotrophomonas maltophilia].
WP_055941681.1	-	133	bacteria>proteobacteria>gammaproteobacteria	Pseudoxanthomonas sp. Root65	attachment protein [Pseudoxanthomonas sp. Root65].
WP_056878569.1	-	133	bacteria>proteobacteria>gammaproteobacteria	Pseudoxanthomonas sp. Root630	attachment protein [Pseudoxanthomonas sp. Root630].
WP_003487911.1	-	132	bacteria>proteobacteria>gammaproteobacteria	Xanthomonas	MULTISPECIES: attachment protein [Xanthomonas].
WP_005921770.1	-	132	bacteria>proteobacteria>gammaproteobacteria	Xanthomonas axonopodis	protein required for attachment to host cell [Xanthomonas axonopodis].
WP_006453262.1	-	132	bacteria>proteobacteria>gammaproteobacteria	Xanthomonas	MULTISPECIES: attachment protein [Xanthomonas].
WP_007962293.1	-	132	bacteria>proteobacteria>gammaproteobacteria	Xanthomonas fuscans	attachment protein [Xanthomonas fuscans].
WP_008577854.1	-	132	bacteria>proteobacteria>gammaproteobacteria	Xanthomonas	MULTISPECIES: attachment protein [Xanthomonas].
WP_010364644.1	-	132	bacteria>proteobacteria>gammaproteobacteria	Xanthomonas	MULTISPECIES: attachment protein [Xanthomonas].
WP_011035339.1	-	132	bacteria>proteobacteria>gammaproteobacteria	Xanthomonas campestris	attachment protein [Xanthomonas campestris].
WP_011050095.1	-	132	bacteria>proteobacteria>gammaproteobacteria	Xanthomonas	MULTISPECIES: attachment protein [Xanthomonas].
WP_014159189.1	-	132	bacteria>proteobacteria>gammaproteobacteria	Pseudoxanthomonas spadix	attachment protein [Pseudoxanthomonas spadix].
WP_014506015.1	-	132	bacteria>proteobacteria>gammaproteobacteria	Xanthomonas campestris	attachment protein [Xanthomonas campestris].
WP_016902952.1	-	132	bacteria>proteobacteria>gammaproteobacteria	Xanthomonas	MULTISPECIES: attachment protein [Xanthomonas].
WP_017157986.1	-	132	bacteria>proteobacteria>gammaproteobacteria	Xanthomonas axonopodis	attachment protein [Xanthomonas axonopodis].
WP_022970655.1	-	132	bacteria>proteobacteria>gammaproteobacteria	Xanthomonas maliensis	attachment protein [Xanthomonas maliensis].
WP_024743101.1	-	132	bacteria>proteobacteria>gammaproteobacteria	Xanthomonas oryzae	attachment protein [Xanthomonas oryzae].
WP_024891678.1	-	132	bacteria>proteobacteria>gammaproteobacteria	Luteimonas huabeiensis	attachment protein [Luteimonas huabeiensis].
KGE53569.1	GW15_0201325	132	bacteria>proteobacteria>gammaproteobacteria	Xanthomonas axonopodis pv. vasculorum	attachment protein [Xanthomonas axonopodis pv. vasculorum].
WP_039580160.1	-	132	bacteria>proteobacteria>gammaproteobacteria	Xanthomonas axonopodis	attachment protein [Xanthomonas axonopodis].
WP_040940290.1	-	132	bacteria>proteobacteria>gammaproteobacteria	Xanthomonas campestris	attachment protein [Xanthomonas campestris].
WP_042595503.1	-	132	bacteria>proteobacteria>gammaproteobacteria	Xanthomonas campestris	attachment protein [Xanthomonas campestris].
WP_047129852.1	-	132	bacteria>proteobacteria>gammaproteobacteria	Xanthomonas arboricola	attachment protein [Xanthomonas arboricola].
WP_047135492.1	-	132	bacteria>proteobacteria>gammaproteobacteria	Luteimonas sp. FCS-9	attachment protein [Luteimonas sp. FCS-9].
KPL49533.1	XAXN_06945	132	bacteria>proteobacteria>gammaproteobacteria	Xanthomonas axonopodis	attachment protein [Xanthomonas axonopodis].
WP_055252126.1	-	132	bacteria>proteobacteria>gammaproteobacteria	Xanthomonas sp. Mitacek01	attachment protein [Xanthomonas sp. Mitacek01].
WP_055823009.1	-	132	bacteria>proteobacteria>gammaproteobacteria	Xanthomonas sp. Leaf131	attachment protein [Xanthomonas sp. Leaf131].
WP_057684060.1	-	132	bacteria>proteobacteria>gammaproteobacteria	Xanthomonas axonopodis	attachment protein [Xanthomonas axonopodis].
WP_065467627.1	-	132	bacteria>proteobacteria>gammaproteobacteria	Xanthomonas bromi	attachment protein [Xanthomonas bromi].
WP_057649197.1	-	130	bacteria>proteobacteria>gammaproteobacteria	Stenotrophomonas panacihumi	attachment protein [Stenotrophomonas panacihumi].
WP_007970382.1	-	127	bacteria>proteobacteria>gammaproteobacteria	Xanthomonas fuscans	attachment protein [Xanthomonas fuscans].
EKS08522.1	LEP1GSC071_2673	281	bacteria>spirochaetes	Leptospira santarosai str. JET	host cell attachment protein [Leptospira santarosai str. JET].
KKO69772.1	XB16_0533	277	bacteria>spirochaetes	Leptospira santarosai	Host cell attachment protein [Leptospira santarosai].
EMO60140.1	LEP1GSC161_1163	268	bacteria>spirochaetes	Leptospira santarosai str. CBC1416	host cell attachment protein [Leptospira santarosai str. CBC1416].
EMO98784.1	LEP1GSC120_1543	268	bacteria>spirochaetes	Leptospira santarosai str. 200702252	host cell attachment protein [Leptospira santarosai str. 200702252].
EMP01131.1	LEP1GSC171_3768	268	bacteria>spirochaetes	Leptospira santarosai str. HAI1380	host cell attachment protein [Leptospira santarosai str. HAI1380].
KKZ81489.1	XB15_0842	246	bacteria>spirochaetes	Leptospira santarosai	Host cell attachment protein [Leptospira santarosai].
AGW94167.1	N234_29445	228	bacteria>proteobacteria>betaproteobacteria	Ralstonia pickettii DTP0602	hypothetical protein N234_29445 [Ralstonia pickettii DTP0602].
EMN21915.1	LEP1GSC063_3510	214	bacteria>spirochaetes	Leptospira santarosai serovar Arenal str. MAVJ 401	host cell attachment protein [Leptospira santarosai serovar Arenal str. MAVJ 401].
KKZ81023.1	XB17_1232	212	bacteria>spirochaetes	Leptospira santarosai	Host cell attachment protein [Leptospira santarosai].
EMO12738.1	LEP1GSC165_3685	210	bacteria>spirochaetes	Leptospira santarosai str. CBC523	host cell attachment protein [Leptospira santarosai str. CBC523].
EMP81867.1	LEP1GSC162_1964	210	bacteria>spirochaetes	Leptospira santarosai str. CBC1531	host cell attachment protein [Leptospira santarosai str. CBC1531].
WP_019463890.1	-	199	bacteria>proteobacteria>gammaproteobacteria	Dyella japonica	hypothetical protein [Dyella japonica].
WP_036142636.1	-	185	bacteria>proteobacteria>gammaproteobacteria	Luteibacter sp. 9135	hypothetical protein [Luteibacter sp. 9135].
WP_011297409.1	-	184	bacteria>proteobacteria>betaproteobacteria	Cupriavidus pinatubonensis	hypothetical protein [Cupriavidus pinatubonensis].
WP_051595328.1	-	183	bacteria>proteobacteria>gammaproteobacteria	Dyella jiangningensis	hypothetical protein [Dyella jiangningensis].
SCB30702.1	GA0116996_111104	183	bacteria>proteobacteria>betaproteobacteria	Cupriavidus alkaliphilus	Protein required for attachment to host cells [Cupriavidus alkaliphilus].
WP_011088071.1	-	182	bacteria>proteobacteria>alphaproteobacteria	Bradyrhizobium diazoefficiens	hypothetical protein [Bradyrhizobium diazoefficiens].
CCU62030.1	EBAPG3_10630	179	bacteria>proteobacteria>betaproteobacteria	Nitrosospira lacus	hypothetical protein EBAPG3_10630 [Nitrosospira lacus].
CEF57303.1	AGA_2620	179	bacteria>proteobacteria>alphaproteobacteria	Acetobacter ghanensis	AtsE protein [Acetobacter ghanensis].
WP_051822669.1	-	178	bacteria>proteobacteria>deltaproteobacteria	Desulfonatronum thiodismutans	hypothetical protein [Desulfonatronum thiodismutans].
WP_013953182.1	-	175	bacteria>proteobacteria>betaproteobacteria	Cupriavidus necator	hypothetical protein [Cupriavidus necator].
WP_010814514.1	-	174	bacteria>proteobacteria>betaproteobacteria	Burkholderiaceae	MULTISPECIES: hypothetical protein [Burkholderiaceae].
WP_011617292.1	-	174	bacteria>proteobacteria>betaproteobacteria	Cupriavidus necator	hypothetical protein [Cupriavidus necator].
WP_012356147.1	-	174	bacteria>proteobacteria>betaproteobacteria	Cupriavidus taiwanensis	hypothetical protein [Cupriavidus taiwanensis].
WP_018007252.1	-	174	bacteria>proteobacteria>betaproteobacteria	Cupriavidus taiwanensis	hypothetical protein [Cupriavidus taiwanensis].
WP_018310933.1	-	174	bacteria>proteobacteria>betaproteobacteria	Cupriavidus sp. UYPR2.512	hypothetical protein [Cupriavidus sp. UYPR2.512].
WP_029049902.1	-	174	bacteria>proteobacteria>betaproteobacteria	Cupriavidus sp. amp6	hypothetical protein [Cupriavidus sp. amp6].
WP_035815373.1	-	174	bacteria>proteobacteria>betaproteobacteria	Cupriavidus sp. SK-4	hypothetical protein [Cupriavidus sp. SK-4].
WP_042878962.1	-	174	bacteria>proteobacteria>betaproteobacteria	Cupriavidus necator	hypothetical protein [Cupriavidus necator].
WP_053824242.1	-	174	bacteria>proteobacteria>betaproteobacteria	Cupriavidus gilardii	hypothetical protein [Cupriavidus gilardii].
WP_062803022.1	-	174	bacteria>proteobacteria>betaproteobacteria	Cupriavidus nantongensis	hypothetical protein [Cupriavidus nantongensis].
WP_063239075.1	-	174	bacteria>proteobacteria>betaproteobacteria	Cupriavidus oxalaticus	hypothetical protein [Cupriavidus oxalaticus].
CAN95436.1	sce5273	173	bacteria>proteobacteria>deltaproteobacteria	Sorangium cellulosum So ce56	hypothetical protein sce5273 [Sorangium cellulosum So ce56].
WP_047195216.1	-	173	bacteria>proteobacteria>deltaproteobacteria	[Polyangium] brachysporum	hypothetical protein [[Polyangium] brachysporum].
KLU35264.1	AB595_19210	173	bacteria>proteobacteria>betaproteobacteria	Massilia sp. WF1	hypothetical protein AB595_19210 [Massilia sp. WF1].
WP_012061418.1	-	172	bacteria>proteobacteria>alphaproteobacteria	Sinorhizobium medicae	hypothetical protein [Sinorhizobium medicae].
WP_007104594.1	-	171	bacteria>proteobacteria>gammaproteobacteria	Paraglaciecola polaris	hypothetical protein [Paraglaciecola polaris].
WP_007403368.1	-	171	bacteria>proteobacteria>alphaproteobacteria	Sphingomonas	MULTISPECIES: required for attachment to host cells family protein [Sphingomonas].
WP_019430679.1	-	171	bacteria>proteobacteria>betaproteobacteria	Limnohabitans sp. Rim47	hypothetical protein [Limnohabitans sp. Rim47].
WP_051243436.1	-	171	bacteria>proteobacteria>betaproteobacteria	Azohydromonas australica	hypothetical protein [Azohydromonas australica].
WP_056437714.1	-	171	bacteria>proteobacteria>alphaproteobacteria	Sphingomonas sp. Leaf257	hypothetical protein [Sphingomonas sp. Leaf257].
WP_022732502.1	-	170	bacteria>proteobacteria>alphaproteobacteria	Thalassospira lucentensis	host attachment protein [Thalassospira lucentensis].
KFI07867.1	JN27_07260	170	bacteria>proteobacteria>betaproteobacteria	Massilia sp. BSC265	hypothetical protein JN27_07260 [Massilia sp. BSC265].
WP_046195133.1	-	170	bacteria>proteobacteria>alphaproteobacteria	Sphingomonas sp. SRS2	required for attachment to host cells family protein [Sphingomonas sp. SRS2].
WP_065309991.1	-	170	bacteria>proteobacteria>betaproteobacteria	Janthinobacterium sp. S3-2	hypothetical protein [Janthinobacterium sp. S3-2].
WP_010543414.1	-	169	bacteria>proteobacteria>alphaproteobacteria	Sphingomonas elodea	hypothetical protein [Sphingomonas elodea].
EPG64268.1	LEP1GSC061_3706	169	bacteria>spirochaetes	Leptospira wolffii serovar Khorat str. Khorat-H2	host cell attachment protein [Leptospira wolffii serovar Khorat str. Khorat-H2].
WP_019371039.1	-	169	bacteria>proteobacteria>alphaproteobacteria	Sphingomonas	MULTISPECIES: host attachment protein [Sphingomonas].
WP_022681595.1	-	169	bacteria>proteobacteria>alphaproteobacteria	Sphingobium sp. YL23	host attachment protein [Sphingobium sp. YL23].
WP_022686320.1	-	169	bacteria>proteobacteria>alphaproteobacteria	Sphingomonas phyllosphaerae	host attachment protein [Sphingomonas phyllosphaerae].
WP_026326074.1	-	169	bacteria>proteobacteria>alphaproteobacteria	Sphingomonas sp. Mn802worker	required for attachment to host cells family protein [Sphingomonas sp. Mn802worker].
WP_028965708.1	-	169	bacteria>proteobacteria>alphaproteobacteria	Sphingomonas phyllosphaerae	hypothetical protein [Sphingomonas phyllosphaerae].
KFC76566.1	FG94_00041	169	bacteria>proteobacteria>betaproteobacteria	Massilia sp. LC238	host cell attachment protein [Massilia sp. LC238].
WP_046410749.1	-	169	bacteria>proteobacteria>alphaproteobacteria	Sphingomonas sp. Ag1	required for attachment to host cells family protein [Sphingomonas sp. Ag1].
WP_051726805.1	-	169	bacteria>proteobacteria>alphaproteobacteria	Mesorhizobium huakuii	hypothetical protein [Mesorhizobium huakuii].
WP_055847809.1	-	169	bacteria>proteobacteria>alphaproteobacteria	Sphingomonas sp. Leaf343	hypothetical protein [Sphingomonas sp. Leaf343].
WP_056637029.1	-	169	bacteria>proteobacteria>alphaproteobacteria	Sphingomonas sp. Leaf231	hypothetical protein [Sphingomonas sp. Leaf231].
WP_058754089.1	-	169	bacteria>proteobacteria>alphaproteobacteria	Sphingomonas endophytica	hypothetical protein [Sphingomonas endophytica].
KYF53639.1	BE08_11610	169	bacteria>proteobacteria>deltaproteobacteria	Sorangium cellulosum	hypothetical protein BE08_11610 [Sorangium cellulosum].
WP_005663003.1	-	168	bacteria>proteobacteria>betaproteobacteria	Massilia timonae	hypothetical protein [Massilia timonae].
WP_009538991.1	-	168	bacteria>proteobacteria>alphaproteobacteria	Caenispirillum salinarum	hypothetical protein [Caenispirillum salinarum].
WP_036172513.1	-	168	bacteria>proteobacteria>betaproteobacteria	Massilia sp. 9096	hypothetical protein [Massilia sp. 9096].
WP_040787599.1	-	168	bacteria>proteobacteria>betaproteobacteria	Massilia niastensis	hypothetical protein [Massilia niastensis].
ABG63654.1	Meso_2265	167	bacteria>proteobacteria>alphaproteobacteria	Chelativorans sp. BNC1	conserved hypothetical protein [Chelativorans sp. BNC1].
WP_006913960.1	-	167	bacteria>proteobacteria>gammaproteobacteria	Salinisphaera shabanensis	hypothetical protein [Salinisphaera shabanensis].
WP_022728395.1	-	167	bacteria>proteobacteria>alphaproteobacteria	Fodinicurvata sediminis	host cell attachment-required protein [Fodinicurvata sediminis].
WP_035724632.1	-	167	bacteria>proteobacteria>alphaproteobacteria	Fodinicurvata fenggangensis	hypothetical protein [Fodinicurvata fenggangensis].
WP_008944356.1	-	166	bacteria>proteobacteria>alphaproteobacteria	Oceanibaculum indicum	hypothetical protein [Oceanibaculum indicum].
KYF89717.1	BE18_27600	166	bacteria>proteobacteria>deltaproteobacteria	Sorangium cellulosum	hypothetical protein BE18_27600 [Sorangium cellulosum].
WP_061619406.1	-	166	bacteria>proteobacteria>deltaproteobacteria	Sorangium cellulosum	hypothetical protein [Sorangium cellulosum].
WP_050431722.1	-	165	bacteria>proteobacteria>deltaproteobacteria	Chondromyces crocatus	hypothetical protein [Chondromyces crocatus].
WP_051487667.1	-	165	bacteria>proteobacteria>alphaproteobacteria	Roseivivax atlanticus	hypothetical protein [Roseivivax atlanticus].
WP_061609448.1	-	165	bacteria>proteobacteria>deltaproteobacteria	Sorangium cellulosum	hypothetical protein [Sorangium cellulosum].
EPG83504.1	LEP1GSC048_2572	164	bacteria>spirochaetes	Leptospira santarosai serovar Shermani str. 1342KT	host cell attachment protein [Leptospira santarosai serovar Shermani str. 1342KT].
EYF04621.1	CAP_4297	164	bacteria>proteobacteria>deltaproteobacteria	Chondromyces apiculatus DSM 436	Hypothetical protein CAP_4297 [Chondromyces apiculatus DSM 436].
BAV52618.1	MLTONO_p0148	164	bacteria>proteobacteria>alphaproteobacteria	Mesorhizobium loti	Protein required for attachment to host cells (plasmid) [Mesorhizobium loti].
WP_009802552.1	-	163	bacteria>proteobacteria>alphaproteobacteria	Oceanicaulis sp. HTCC2633	hypothetical protein [Oceanicaulis sp. HTCC2633].
WP_011583296.1	-	163	bacteria>proteobacteria>alphaproteobacteria	Chelativorans sp. BNC1	protein required for attachment to host cells-like [Chelativorans sp. BNC1].
WP_019403674.1	-	163	bacteria>proteobacteria>alphaproteobacteria	Chelatococcus	MULTISPECIES: hypothetical protein [Chelatococcus].
WP_022700982.1	-	163	bacteria>proteobacteria>alphaproteobacteria	Oceanicaulis alexandrii	hypothetical protein [Oceanicaulis alexandrii].
BAE52584.1	amb3780	162	bacteria>proteobacteria>alphaproteobacteria	Magnetospirillum magneticum AMB-1	Protein required for attachment to host cells [Magnetospirillum magneticum AMB-1].
WP_024894828.1	-	162	bacteria>proteobacteria	Acidithiobacillus thiooxidans	hypothetical protein [Acidithiobacillus thiooxidans].
WP_009858385.1	-	161	bacteria>proteobacteria>betaproteobacteria	Rubrivivax benzoatilyticus	host attachment protein [Rubrivivax benzoatilyticus].
WP_014426832.1	-	161	bacteria>proteobacteria>betaproteobacteria	Rubrivivax gelatinosus	hypothetical protein [Rubrivivax gelatinosus].
WP_050986113.1	-	161	bacteria>planctomycetes	Schlesneria paludicola	hypothetical protein [Schlesneria paludicola].
WP_058496148.1	-	161	bacteria>proteobacteria>gammaproteobacteria	Legionella drozanskii	hypothetical protein [Legionella drozanskii].
WP_058574615.1	-	161	bacteria>proteobacteria>gammaproteobacteria	Halothiobacillus	MULTISPECIES: hypothetical protein [Halothiobacillus].
AMV20129.1	VT03_19695	161	bacteria>planctomycetes	Planctomyces sp. SH-PL14	host attachment protein [Planctomyces sp. SH-PL14].
WP_013543450.1	-	160	bacteria>proteobacteria>betaproteobacteria	Variovorax paradoxus	host attachment protein [Variovorax paradoxus].
KEC76177.1	RLPCCGM1_c0280	160	bacteria>proteobacteria>alphaproteobacteria	Rhizobium leguminosarum bv. phaseoli CCGM1	hypothetical protein RLPCCGM1_c0280 [Rhizobium leguminosarum bv. phaseoli CCGM1].
GAN81835.1	Aam_123_001	160	bacteria>proteobacteria>alphaproteobacteria	Acidocella aminolytica 101 = DSM 11237	hypothetical protein Aam_123_001 [Acidocella aminolytica 101 = DSM 11237].
WP_051434963.1	-	160	bacteria>proteobacteria>alphaproteobacteria	Microvirga flocculans	hypothetical protein [Microvirga flocculans].
WP_051613821.1	-	160	bacteria>nitrospirae	Leptospirillum ferriphilum	hypothetical protein [Leptospirillum ferriphilum].
WP_062011160.1	-	160	bacteria>proteobacteria>alphaproteobacteria	Aureimonas sp. AU4	hypothetical protein [Aureimonas sp. AU4].
EAU55308.1	SPV1_11266	159	bacteria>proteobacteria	Mariprofundus ferrooxydans PV-1	hypothetical protein SPV1_11266 [Mariprofundus ferrooxydans PV-1].
WP_011090683.1	-	159	bacteria>proteobacteria>alphaproteobacteria	Bradyrhizobium diazoefficiens	hypothetical protein [Bradyrhizobium diazoefficiens].
WP_022697103.1	-	159	bacteria>proteobacteria>alphaproteobacteria	Euryhalocaulis caribicus	host attachment protein [Euryhalocaulis caribicus].
KGJ64245.1	BJA5080_06047	159	bacteria>proteobacteria>alphaproteobacteria	Bradyrhizobium diazoefficiens SEMIA 5080	hypothetical protein BJA5080_06047 [Bradyrhizobium diazoefficiens SEMIA 5080].
WP_040455167.1	-	159	bacteria>proteobacteria>gammaproteobacteria	Hydrocarboniphaga effusa	hypothetical protein [Hydrocarboniphaga effusa].
WP_051677340.1	-	159	bacteria>proteobacteria>alphaproteobacteria	Bradyrhizobium sp. URHD0069	hypothetical protein [Bradyrhizobium sp. URHD0069].
KPQ16173.1	HLUCCO18_09475	159	bacteria>proteobacteria>alphaproteobacteria	Rhodobacteraceae bacterium HLUCCO18	hypothetical protein HLUCCO18_09475 [Rhodobacteraceae bacterium HLUCCO18].
WP_056873294.1	-	159	bacteria>proteobacteria>betaproteobacteria	Pelomonas sp. Root1444	hypothetical protein [Pelomonas sp. Root1444].
KSV88568.1	N184_28870	159	bacteria>proteobacteria>alphaproteobacteria	Sinorhizobium sp. GL28	hypothetical protein N184_28870 [Sinorhizobium sp. GL28].
WP_020649878.1	-	158	bacteria>proteobacteria>gammaproteobacteria	Solimonas variicoloris	hypothetical protein [Solimonas variicoloris].
WP_043112111.1	-	158	bacteria>proteobacteria>gammaproteobacteria	Solimonas flava	hypothetical protein [Solimonas flava].
WP_051255423.1	-	158	bacteria>proteobacteria>betaproteobacteria	Variovorax paradoxus	hypothetical protein [Variovorax paradoxus].
WP_053063343.1	-	158	bacteria>proteobacteria>betaproteobacteria	Variovorax paradoxus	hypothetical protein [Variovorax paradoxus].
EKR65607.1	LEP1GSC036_4074	157	bacteria>spirochaetes	Leptospira weilii str. 2006001853	host cell attachment protein [Leptospira weilii str. 2006001853].
WP_008437076.1	-	157	bacteria>proteobacteria>gammaproteobacteria	Rhodanobacter thiooxydans	host attachment protein [Rhodanobacter thiooxydans].
WP_034759695.1	-	157	bacteria>proteobacteria>betaproteobacteria	Janthinobacterium lividum	hypothetical protein [Janthinobacterium lividum].
WP_035825512.1	-	157	bacteria>proteobacteria>betaproteobacteria	Janthinobacterium sp. RA13	hypothetical protein [Janthinobacterium sp. RA13].
KIA77256.1	DB43_GR00230	157	bacteria>chlamydiae	Parachlamydia acanthamoebae	hypothetical protein DB43_GR00230 [Parachlamydia acanthamoebae].
KIC74076.1	DB42_BL00010	157	bacteria>chlamydiae	Neochlamydia sp. EPS4	hypothetical protein DB42_BL00010 [Neochlamydia sp. EPS4].
WP_046685283.1	-	157	bacteria>proteobacteria>betaproteobacteria	Janthinobacterium sp. KBS0711	hypothetical protein [Janthinobacterium sp. KBS0711].
KPP86821.1	HLUCCO07_13330	157	bacteria>proteobacteria>alphaproteobacteria	Rhodobacteraceae bacterium HLUCCO07	hypothetical protein HLUCCO07_13330 [Rhodobacteraceae bacterium HLUCCO07].
WP_058051376.1	-	157	bacteria>proteobacteria>betaproteobacteria	Janthinobacterium sp. Ant5-2-1	hypothetical protein [Janthinobacterium sp. Ant5-2-1].
BAB54785.1	ml19589	156	bacteria>proteobacteria>alphaproteobacteria	Mesorhizobium loti MAFF303099	AtsE (plasmid) [Mesorhizobium loti MAFF303099].
WP_028079584.1	-	156	bacteria>proteobacteria>gammaproteobacteria	Solimonas soli	hypothetical protein [Solimonas soli].
WP_029942340.1	-	156	bacteria>proteobacteria>alphaproteobacteria	Sphingomonas astaxanthinifaciens	hypothetical protein [Sphingomonas astaxanthinifaciens].
WP_037501510.1	-	156	bacteria>proteobacteria>alphaproteobacteria	Sphingomonas jaspsi	hypothetical protein [Sphingomonas jaspsi].
WP_057289567.1	-	156	bacteria>proteobacteria>betaproteobacteria	Noviherbaspirillum sp. Root189	hypothetical protein [Noviherbaspirillum sp. Root189].
KUJ77189.1	AVO44_18460	156	bacteria>proteobacteria>alphaproteobacteria	Ruegeria sp. ZGT108	hypothetical protein AVO44_18460 [Ruegeria sp. ZGT108].
WP_011942242.1	-	155	bacteria>proteobacteria>alphaproteobacteria	Acidiphilium	MULTISPECIES: hypothetical protein [Acidiphilium].
WP_012187303.1	-	155	bacteria>proteobacteria>alphaproteobacteria	Dinoroseobacter shibae	hypothetical protein [Dinoroseobacter shibae].
WP_012591414.1	-	155	bacteria>proteobacteria>alphaproteobacteria	Methylocella silvestris	hypothetical protein [Methylocella silvestris].
WP_026608396.1	-	155	bacteria>proteobacteria>alphaproteobacteria	Methylocapsa acidiphila	host attachment protein [Methylocapsa acidiphila].
KHQ51482.1	OA50_03977	155	bacteria>proteobacteria>alphaproteobacteria	Mameliella alba	Host attachment protein [Mameliella alba].
WP_036237887.1	-	155	bacteria>proteobacteria>betaproteobacteria	Massilia sp. JS1662	hypothetical protein [Massilia sp. JS1662].
WP_036257457.1	-	155	bacteria>proteobacteria>alphaproteobacteria	Methylocapsa aurea	host attachment protein [Methylocapsa aurea].
WP_039888129.1	-	155	bacteria>proteobacteria>alphaproteobacteria	Acidiphilium sp. PM	hypothetical protein [Acidiphilium sp. PM].
WP_043747645.1	-	155	bacteria>proteobacteria>alphaproteobacteria	Pseudooceanicola atlanticus	host attachment protein [Pseudooceanicola atlanticus].
WP_046347110.1	-	155	bacteria>proteobacteria>alphaproteobacteria	Sphingomonas changbaiensis	host attachment protein [Sphingomonas changbaiensis].
WP_051531874.1	-	155	bacteria>proteobacteria>alphaproteobacteria	Sphingomonas sp. URHD0057	hypothetical protein [Sphingomonas sp. URHD0057].
WP_056129841.1	-	155	bacteria>proteobacteria>betaproteobacteria	Massilia sp. Root133	hypothetical protein [Massilia sp. Root133].
WP_056328765.1	-	155	bacteria>proteobacteria>betaproteobacteria	Massilia sp. Root1485	hypothetical protein [Massilia sp. Root1485].
WP_056435145.1	-	155	bacteria>proteobacteria>betaproteobacteria	Massilia sp. Root335	hypothetical protein [Massilia sp. Root335].
WP_056586355.1	-	155	bacteria>proteobacteria>alphaproteobacteria	Mesorhizobium	MULTISPECIES: hypothetical protein [Mesorhizobium].
WP_058246082.1	-	155	bacteria>proteobacteria>alphaproteobacteria	Tropicibacter naphthalenivorans	host attachment protein [Tropicibacter naphthalenivorans].
ANJ66332.1	A9404_02110	155	bacteria>proteobacteria>gammaproteobacteria	Halothiobacillus sp. LS2	hypothetical protein A9404_02110 [Halothiobacillus sp. LS2].
EMG21364.1	LEP1GSC150_4227	154	bacteria>spirochaetes	Leptospira interrogans serovar Copenhageni str. LT2050	host cell attachment protein, partial [Leptospira interrogans serovar Copenhageni str. LT2050].
WP_018267035.1	-	154	bacteria>proteobacteria>alphaproteobacteria	Methylosinus sp. LW4	host attachment protein [Methylosinus sp. LW4].
WP_019142134.1	-	154	bacteria>proteobacteria>betaproteobacteria	Herbaspirillum massiliense	hypothetical protein [Herbaspirillum massiliense].
WP_020040366.1	-	154	bacteria>proteobacteria>alphaproteobacteria	Salipiger mucosus	hypothetical protein [Salipiger mucosus].
WP_024878403.1	-	154	bacteria>proteobacteria>alphaproteobacteria	Methylocystaceae	MULTISPECIES: host cell attachment protein [Methylocystaceae].
WP_025307795.1	-	154	bacteria>proteobacteria>deltaproteobacteria	Bdellovibrio bacteriovorus	hypothetical protein [Bdellovibrio bacteriovorus].
AIL12828.1	IM40_03725	154	bacteria>proteobacteria>alphaproteobacteria	Candidatus Caedibacter acanthamoebae	hypothetical protein IM40_03725 [Candidatus Caedibacter acanthamoebae].
WP_036296145.1	-	154	bacteria>proteobacteria>alphaproteobacteria	Methylosinus sp. PW1	host cell attachment protein [Methylosinus sp. PW1].
WP_038386902.1	-	154	bacteria>proteobacteria>alphaproteobacteria	Bradyrhizobium elkanii	host cell attachment protein [Bradyrhizobium elkanii].
WP_054360454.1	-	154	bacteria>proteobacteria>alphaproteobacteria	Prosthecomicrobium hirschii	hypothetical protein [Prosthecomicrobium hirschii].
WP_057750230.1	-	154	bacteria>proteobacteria>alphaproteobacteria	Bradyrhizobium manausense	host cell attachment protein [Bradyrhizobium manausense].
KUO56748.1	APF78_00475	154	bacteria>proteobacteria>alphaproteobacteria	Sphingomonadales bacterium BRH_c3	hypothetical protein APF78_00475 [Sphingomonadales bacterium BRH_c3].
WP_063693761.1	-	154	bacteria>proteobacteria>alphaproteobacteria	Bradyrhizobium stylosanthis	host cell attachment protein [Bradyrhizobium stylosanthis].
ACC75912.1	Bphy_6901	153	bacteria>proteobacteria>betaproteobacteria	Paraburkholderia phymatum STM815	hypothetical protein Bphy_6901 (plasmid) [Paraburkholderia phymatum STM815].
ACM25965.1	Arad_1550	153	bacteria>proteobacteria>alphaproteobacteria	Agrobacterium radiobacter K84	conserved hypothetical protein [Agrobacterium radiobacter K84].
WP_002717325.1	-	153	bacteria>proteobacteria>alphaproteobacteria	Afipia felis	hypothetical protein [Afipia felis].
WP_007603102.1	-	153	bacteria>proteobacteria>alphaproteobacteria	Bradyrhizobium sp. WSM1253	protein required for attachment to host cells [Bradyrhizobium sp. WSM1253].
WP_007611511.1	-	153	bacteria>proteobacteria>alphaproteobacteria	Bradyrhizobium	MULTISPECIES: host cell attachment protein [Bradyrhizobium].
WP_008145017.1	-	153	bacteria>proteobacteria>alphaproteobacteria	Bradyrhizobium sp. YR681	protein required for attachment to host cell [Bradyrhizobium sp. YR681].
WP_008549027.1	-	153	bacteria>proteobacteria>alphaproteobacteria	Bradyrhizobium	MULTISPECIES: hypothetical protein [Bradyrhizobium].
WP_009337666.1	-	153	bacteria>proteobacteria>alphaproteobacteria	Afipia	MULTISPECIES: host attachment protein [Afipia].
WP_011910523.1	-	153	bacteria>proteobacteria>alphaproteobacteria	Rhodobacter sphaeroides	hypothetical protein [Rhodobacter sphaeroides].
WP_012562183.1	-	153	bacteria>proteobacteria>alphaproteobacteria	Oligotropha carboxidovorans	host cell attachment protein [Oligotropha carboxidovorans].
WP_013505665.1	-	153	bacteria>chrysiogenetes	Desulfurispirillum indicum	host attachment protein [Desulfurispirillum indicum].
WP_015685472.1	-	153	bacteria>proteobacteria>alphaproteobacteria	Bradyrhizobium sp. S23321	hypothetical protein [Bradyrhizobium sp. S23321].
WP_017667697.1	-	153	bacteria>proteobacteria>alphaproteobacteria	Sandarakinorhabdus sp. AAP62	hypothetical protein [Sandarakinorhabdus sp. AAP62].
WP_018268717.1	-	153	bacteria>proteobacteria>alphaproteobacteria	Bradyrhizobium elkanii	hypothetical protein [Bradyrhizobium elkanii].
WP_018268817.1	-	153	bacteria>proteobacteria>alphaproteobacteria	Bradyrhizobium elkanii	hypothetical protein [Bradyrhizobium elkanii].
WP_018983877.1	-	153	bacteria>proteobacteria>gammaproteobacteria	Salinimonas chungwhensis	hypothetical protein [Salinimonas chungwhensis].
WP_024342507.1	-	153	bacteria>proteobacteria>alphaproteobacteria	Bradyrhizobium japonicum	host attachment protein [Bradyrhizobium japonicum].
WP_025036933.1	-	153	bacteria>proteobacteria>alphaproteobacteria	Bradyrhizobium sp. DOA9	host attachment protein [Bradyrhizobium sp. DOA9].
WP_026202722.1	-	153	bacteria>proteobacteria>alphaproteobacteria	Bradyrhizobium sp. WSM2793	host attachment protein [Bradyrhizobium sp. WSM2793].
WP_026312678.1	-	153	bacteria>proteobacteria>alphaproteobacteria	Bradyrhizobium japonicum	host attachment protein [Bradyrhizobium japonicum].
WP_027518541.1	-	153	bacteria>proteobacteria>alphaproteobacteria	Bradyrhizobium sp. WSM1417	host attachment protein [Bradyrhizobium sp. WSM1417].
WP_027527205.1	-	153	bacteria>proteobacteria>alphaproteobacteria	Bradyrhizobium sp. Ec3.3	host attachment protein [Bradyrhizobium sp. Ec3.3].
WP_027535480.1	-	153	bacteria>proteobacteria>alphaproteobacteria	Bradyrhizobium sp. WSM3983	host attachment protein [Bradyrhizobium sp. WSM3983].
WP_027549260.1	-	153	bacteria>proteobacteria>alphaproteobacteria	Bradyrhizobium sp. WSM2254	host attachment protein [Bradyrhizobium sp. WSM2254].
WP_027555135.1	-	153	bacteria>proteobacteria>alphaproteobacteria	Bradyrhizobium	MULTISPECIES: host attachment protein [Bradyrhizobium].
WP_027559838.1	-	153	bacteria>proteobacteria>alphaproteobacteria	Bradyrhizobium	MULTISPECIES: host attachment protein [Bradyrhizobium].
WP_027567448.1	-	153	bacteria>proteobacteria>alphaproteobacteria	Bradyrhizobium sp. URHA0013	host attachment protein [Bradyrhizobium sp. URHA0013].
WP_028136962.1	-	153	bacteria>proteobacteria>alphaproteobacteria	Bradyrhizobium japonicum	host attachment protein [Bradyrhizobium japonicum].
WP_028140960.1	-	153	bacteria>proteobacteria>alphaproteobacteria	Bradyrhizobium	MULTISPECIES: host attachment protein [Bradyrhizobium].
WP_028153617.1	-	153	bacteria>proteobacteria>alphaproteobacteria	Bradyrhizobium japonicum	host attachment protein [Bradyrhizobium japonicum].
WP_028178619.1	-	153	bacteria>proteobacteria>alphaproteobacteria	Bradyrhizobium	MULTISPECIES: host attachment protein [Bradyrhizobium].
WP_028342834.1	-	153	bacteria>proteobacteria>alphaproteobacteria	Bradyrhizobium elkanii	host cell attachment protein [Bradyrhizobium elkanii].
KHD87870.1	OM95_12330	153	bacteria>proteobacteria>deltaproteobacteria	Bdellovibrio sp. ArHS	hypothetical protein OM95_12330 [Bdellovibrio sp. ArHS].
WP_035709674.1	-	153	bacteria>proteobacteria>alphaproteobacteria	Bradyrhizobium sp. CCBAU 43298	host attachment protein [Bradyrhizobium sp. CCBAU 43298].
WP_036011176.1	-	153	bacteria>proteobacteria>alphaproteobacteria	Bradyrhizobium yuanmingense	host attachment protein [Bradyrhizobium yuanmingense].
WP_036031617.1	-	153	bacteria>proteobacteria>alphaproteobacteria	Bradyrhizobium yuanmingense	host attachment protein [Bradyrhizobium yuanmingense].
WP_036041427.1	-	153	bacteria>proteobacteria>alphaproteobacteria	Bradyrhizobium yuanmingense	host attachment protein [Bradyrhizobium yuanmingense].
WP_037310071.1	-	153	bacteria>proteobacteria>alphaproteobacteria	Ruegeria halocynthiae	attachment protein [Ruegeria halocynthiae].
WP_037515330.1	-	153	bacteria>proteobacteria>alphaproteobacteria	Sphingopyxis sp. LC81	hypothetical protein [Sphingopyxis sp. LC81].
WP_037555677.1	-	153	bacteria>proteobacteria>alphaproteobacteria	Sphingopyxis sp. LC363	hypothetical protein [Sphingopyxis sp. LC363].
WP_038950353.1	-	153	bacteria>proteobacteria>alphaproteobacteria	Bradyrhizobium	MULTISPECIES: host attachment protein [Bradyrhizobium].
WP_039574174.1	-	153	bacteria>proteobacteria>alphaproteobacteria	Sphingopyxis fribergensis	hypothetical protein [Sphingopyxis fribergensis].
KIG14360.1	DB30_06835	153	bacteria>proteobacteria>deltaproteobacteria	Enhygromyxa salina	hypothetical protein DB30_06835 [Enhygromyxa salina].
WP_045465757.1	-	153	bacteria>proteobacteria>betaproteobacteria	Burkholderiales bacterium GJ-E10	hypothetical protein [Burkholderiales bacterium GJ-E10].
WP_046829524.1	-	153	bacteria>proteobacteria>alphaproteobacteria	Afipia massiliensis	host cell attachment protein [Afipia massiliensis].
WP_050602065.1	-	153	bacteria>proteobacteria>alphaproteobacteria	Ruegeria sp. 6PALISEP08	attachment protein [Ruegeria sp. 6PALISEP08].
WP_051420086.1	-	153	bacteria>proteobacteria>alphaproteobacteria	Rhizobium sp. IBUN	hypothetical protein [Rhizobium sp. IBUN].
WP_051883158.1	-	153	bacteria>proteobacteria>gammaproteobacteria	Salinisphaera hydrothermalis	hypothetical protein [Salinisphaera hydrothermalis].
WP_052472389.1	-	153	bacteria>proteobacteria>betaproteobacteria	Burkholderiales bacterium GJ-E10	hypothetical protein [Burkholderiales bacterium GJ-E10].
WP_052743644.1	-	153	bacteria>proteobacteria>alphaproteobacteria	Candidatus Filomicrobium marinum	hypothetical protein [Candidatus Filomicrobium marinum].
WP_053554009.1	-	153	bacteria>proteobacteria>alphaproteobacteria	Sphingopyxis sp. 113P3	hypothetical protein [Sphingopyxis sp. 113P3].
WP_054587571.1	-	153	bacteria>proteobacteria>alphaproteobacteria	Sphingopyxis macrogoltabida	hypothetical protein [Sphingopyxis macrogoltabida].
WP_054726515.1	-	153	bacteria>proteobacteria>alphaproteobacteria	Sphingopyxis macrogoltabida	hypothetical protein [Sphingopyxis macrogoltabida].
WP_056266214.1	-	153	bacteria>proteobacteria>betaproteobacteria	Pelomonas	MULTISPECIES: hypothetical protein [Pelomonas].
WP_056374787.1	-	153	bacteria>proteobacteria>alphaproteobacteria	Sphingopyxis	MULTISPECIES: hypothetical protein [Sphingopyxis].
WP_057029669.1	-	153	bacteria>proteobacteria>alphaproteobacteria	Bradyrhizobium yuanmingense	host attachment protein [Bradyrhizobium yuanmingense].
WP_057198961.1	-	153	bacteria>proteobacteria>alphaproteobacteria	Bradyrhizobium sp. Leaf396	host attachment protein [Bradyrhizobium sp. Leaf396].
WP_057751655.1	-	153	bacteria>proteobacteria>alphaproteobacteria	Bradyrhizobium manausense	host attachment protein [Bradyrhizobium manausense].
WP_058456064.1	-	153	bacteria>proteobacteria>alphaproteobacteria	Sphingopyxis	MULTISPECIES: hypothetical protein [Sphingopyxis].
WP_058538477.1	-	153	bacteria>proteobacteria>alphaproteobacteria	Sphingopyxis	MULTISPECIES: hypothetical protein [Sphingopyxis].
WP_058809464.1	-	153	bacteria>proteobacteria>alphaproteobacteria	Sphingopyxis	MULTISPECIES: hypothetical protein [Sphingopyxis].
WP_058934460.1	-	153	bacteria>proteobacteria>betaproteobacteria	Roseateles depolymerans	hypothetical protein [Roseateles depolymerans].
WP_060912564.1	-	153	bacteria>proteobacteria>alphaproteobacteria	Bradyrhizobium diazoefficiens	host attachment protein [Bradyrhizobium diazoefficiens].
AMG74311.1	SGRAN_1934	153	bacteria>proteobacteria>alphaproteobacteria	Sphingopyxis granuli	Required for attachment to host cells family protein [Sphingopyxis granuli].
WP_061027173.1	-	153	bacteria>proteobacteria>alphaproteobacteria	Bradyrhizobium sp. CCH5-F6	host attachment protein [Bradyrhizobium sp. CCH5-F6].
WP_061882750.1	-	153	bacteria>proteobacteria>alphaproteobacteria	Bradyrhizobium liaoningense	host attachment protein [Bradyrhizobium liaoningense].
WP_062902334.1	-	153	bacteria>proteobacteria>alphaproteobacteria	Sphingopyxis terrae	hypothetical protein [Sphingopyxis terrae].
AMY69562.1	AKL17_2316	153	bacteria>proteobacteria>alphaproteobacteria	Defluviimonas alba	hypothetical protein AKL17_2316 [Defluviimonas alba].
WP_063203374.1	-	153	bacteria>proteobacteria>alphaproteobacteria	Bradyrhizobium sp. AT1	host attachment protein [Bradyrhizobium sp. AT1].
WP_063208471.1	-	153	bacteria>proteobacteria>deltaproteobacteria	Bdellovibrio bacteriovorus	hypothetical protein [Bdellovibrio bacteriovorus].
WP_063245114.1	-	153	bacteria>proteobacteria>deltaproteobacteria	Bdellovibrio bacteriovorus	hypothetical protein [Bdellovibrio bacteriovorus].
WP_063682828.1	-	153	bacteria>proteobacteria>alphaproteobacteria	Bradyrhizobium stylosanthis	host attachment protein [Bradyrhizobium stylosanthis].
WP_063982648.1	-	153	bacteria>proteobacteria>alphaproteobacteria	Bradyrhizobium sp. G22	host attachment protein [Bradyrhizobium sp. G22].
ANH71046.1	ABE85_26455	153	bacteria>proteobacteria>betaproteobacteria	Mitsuaria sp. 7	hypothetical protein ABE85_26455 (plasmid) [Mitsuaria sp. 7].
WP_065091747.1	-	153	bacteria>proteobacteria>alphaproteobacteria	Rhizobium leucaenae	hypothetical protein [Rhizobium leucaenae].
SCB37571.1	GA0061098_1007146	153	bacteria>proteobacteria>alphaproteobacteria	Bradyrhizobium sp. err11	Protein required for attachment to host cells [Bradyrhizobium sp. err11].
SCK27883.1	VAR608DRAFT_2308	153	bacteria>proteobacteria>betaproteobacteria	Variovorax sp. HW608	Protein required for attachment to host cells [Variovorax sp. HW608].
WP_065968688.1	-	153	bacteria>proteobacteria>gammaproteobacteria	Acidiferrobacter thiooxydans	hypothetical protein [Acidiferrobacter thiooxydans].
EIM71623.1	A33O_23664	152	bacteria>proteobacteria>alphaproteobacteria	Nitratireductor aquibiodomus RA22	protein required for attachment to host cells [Nitratireductor aquibiodomus RA22].
WP_002712754.1	-	152	bacteria>proteobacteria>alphaproteobacteria	Afipia clevelandensis	hypothetical protein [Afipia clevelandensis].
WP_003613058.1	-	152	bacteria>proteobacteria>alphaproteobacteria	Methylosinus trichosporium	host attachment protein [Methylosinus trichosporium].
WP_006021560.1	-	152	bacteria>proteobacteria>alphaproteobacteria	Afipia broomeae	hypothetical protein [Afipia broomeae].
WP_011164009.1	-	152	bacteria>proteobacteria>deltaproteobacteria	Bdellovibrio bacteriovorus	hypothetical protein [Bdellovibrio bacteriovorus].
WP_011465287.1	-	152	bacteria>proteobacteria>betaproteobacteria	Rhodoferax ferrireducens	hypothetical protein [Rhodoferax ferrireducens].
WP_011799816.1	-	152	bacteria>proteobacteria>betaproteobacteria	Polaromonas naphthalenivorans	hypothetical protein [Polaromonas naphthalenivorans].
WP_011870005.1	-	152	bacteria>proteobacteria>betaproteobacteria	Herminiimonas arsenicoxydans	hypothetical protein [Herminiimonas arsenicoxydans].
WP_014494571.1	-	152	bacteria>proteobacteria>alphaproteobacteria	Bradyrhizobium japonicum	hypothetical protein [Bradyrhizobium japonicum].
WP_015090673.1	-	152	bacteria>proteobacteria>deltaproteobacteria	Bdellovibrio bacteriovorus	hypothetical protein [Bdellovibrio bacteriovorus].
WP_016921930.1	-	152	bacteria>proteobacteria>alphaproteobacteria	Methylocystis parvus	host attachment protein [Methylocystis parvus].
WP_018388186.1	-	152	bacteria>proteobacteria>alphaproteobacteria	Ancylobacter sp. FA202	protein required for attachment to host cells [Ancylobacter sp. FA202].
WP_019016254.1	-	152	bacteria>proteobacteria>alphaproteobacteria	Elioraea tepidiphila	hypothetical protein [Elioraea tepidiphila].
WP_019198231.1	-	152	bacteria>proteobacteria>alphaproteobacteria	Bradyrhizobiaceae	MULTISPECIES: hypothetical protein [Bradyrhizobiaceae].
WP_021096639.1	-	152	bacteria>proteobacteria>alphaproteobacteria	Rubellimicrobium thermophilum	protein required for attachment to host cell [Rubellimicrobium thermophilum].
WP_024574703.1	-	152	bacteria>proteobacteria>alphaproteobacteria	Afipia	MULTISPECIES: host cell attachment protein [Afipia].
WP_024707616.1	-	152	bacteria>proteobacteria>alphaproteobacteria	Martelella sp. AD-3	protein required for attachment to host cells [Martelella sp. AD-3].
WP_024920791.1	-	152	bacteria>proteobacteria>alphaproteobacteria	Afipia	MULTISPECIES: host cell attachment protein [Afipia].
WP_028154933.1	-	152	bacteria>proteobacteria>alphaproteobacteria	Bradyrhizobium japonicum	host attachment protein [Bradyrhizobium japonicum].
WP_029351941.1	-	152	bacteria>proteobacteria>alphaproteobacteria	Bosea sp. 117	host attachment protein [Bosea sp. 117].
WP_029934950.1	-	152	bacteria>proteobacteria>alphaproteobacteria	Sphingomonas sp. UNC305MFCol5.2	hypothetical protein [Sphingomonas sp. UNC305MFCol5.2].
KEO56276.1	TP2_01765	152	bacteria>proteobacteria>alphaproteobacteria	Thioclava pacifica DSM 10166	hypothetical protein TP2_01765 [Thioclava pacifica DSM 10166].
WP_034469517.1	-	152	bacteria>proteobacteria>alphaproteobacteria	Afipia sp. P52-10	host cell attachment protein [Afipia sp. P52-10].
WP_037247416.1	-	152	bacteria>proteobacteria>alphaproteobacteria	Roseovarius sp. MCTG156(2b)	host attachment protein [Roseovarius sp. MCTG156(2b)].
WP_038620775.1	-	152	bacteria>proteobacteria>gammaproteobacteria	Dyella jiangningensis	hypothetical protein [Dyella jiangningensis].
WP_038936223.1	-	152	bacteria>proteobacteria>alphaproteobacteria	Bradyrhizobium japonicum	host attachment protein [Bradyrhizobium japonicum].
WP_038956428.1	-	152	bacteria>proteobacteria>alphaproteobacteria	Bradyrhizobium japonicum	host attachment protein [Bradyrhizobium japonicum].
WP_040638653.1	-	152	bacteria>proteobacteria>alphaproteobacteria	Microvirga lotononidis	hypothetical protein [Microvirga lotononidis].
WP_041751142.1	-	152	bacteria>proteobacteria>alphaproteobacteria	Bradyrhizobium sp. BTAi1	host cell attachment protein [Bradyrhizobium sp. BTAi1].
WP_045684800.1	-	152	bacteria>proteobacteria>alphaproteobacteria	Martelella endophytica	protein required for attachment to host cells [Martelella endophytica].
WP_046973130.1	-	152	bacteria>proteobacteria>gammaproteobacteria	Dyella japonica	hypothetical protein [Dyella japonica].
WP_051856200.1	-	152	bacteria>proteobacteria>alphaproteobacteria	Thioclava atlantica	hypothetical protein [Thioclava atlantica].
WP_052743888.1	-	152	bacteria>proteobacteria>alphaproteobacteria	Candidatus Filomicrobium marinum	hypothetical protein [Candidatus Filomicrobium marinum].
WP_055036034.1	-	152	bacteria>proteobacteria>alphaproteobacteria	Blastochloris viridis	hypothetical protein [Blastochloris viridis].
WP_055458329.1	-	152	bacteria>proteobacteria>alphaproteobacteria	Chelatococcus sambhunathii	hypothetical protein [Chelatococcus sambhunathii].
WP_055828685.1	-	152	bacteria>proteobacteria>alphaproteobacteria	Brevundimonas	MULTISPECIES: hypothetical protein [Brevundimonas].
WP_056614962.1	-	152	bacteria>proteobacteria>alphaproteobacteria	Sphingomonas sp. Root241	host attachment protein [Sphingomonas sp. Root241].
KWT73313.1	APY03_5928	152	bacteria>proteobacteria>betaproteobacteria	Variovorax sp. WDL1	hypothetical protein APY03_5928 [Variovorax sp. WDL1].
KWV52835.1	AS156_09285	152	bacteria>proteobacteria>alphaproteobacteria	Bradyrhizobium sp. BR 10303	host cell attachment protein [Bradyrhizobium sp. BR 10303].
AMS42179.1	AA2016_3257	152	bacteria>proteobacteria>alphaproteobacteria	Aminobacter aminovorans	hypothetical protein AA2016_3257 [Aminobacter aminovorans].
WP_063697365.1	-	152	bacteria>proteobacteria>alphaproteobacteria	Bradyrhizobium sp. BR 10245	host attachment protein [Bradyrhizobium sp. BR 10245].
EKO50452.1	LEP1GSC131_1189	151	bacteria>spirochaetes	Leptospira kirschneri str. 200802841	host cell attachment protein [Leptospira kirschneri str. 200802841].
WP_006035101.1	-	151	bacteria>proteobacteria>gammaproteobacteria	Rickettsiella grylli	hypothetical protein [Rickettsiella grylli].
WP_007089312.1	-	151	bacteria>proteobacteria>alphaproteobacteria	Thalassospira xiamenensis	hypothetical protein [Thalassospira xiamenensis].
WP_008883950.1	-	151	bacteria>proteobacteria>alphaproteobacteria	Citreicella sp. SE45	hypothetical protein [Citreicella sp. SE45].
WP_009506352.1	-	151	bacteria>proteobacteria>alphaproteobacteria	Citreicella sp. 357	hypothetical protein [Citreicella sp. 357].
WP_013168222.1	-	151	bacteria>proteobacteria>alphaproteobacteria	Starkeya novella	host attachment protein [Starkeya novella].
WP_013214630.1	-	151	bacteria>proteobacteria>alphaproteobacteria	Hyphomicrobium denitrificans	host attachment protein [Hyphomicrobium denitrificans].
WP_014130620.1	-	151	bacteria>proteobacteria>alphaproteobacteria	Pelagibacterium halotolerans	hypothetical protein [Pelagibacterium halotolerans].
WP_024510998.1	-	151	bacteria>proteobacteria>alphaproteobacteria	Bradyrhizobium sp. ARR65	host cell attachment protein [Bradyrhizobium sp. ARR65].
WP_025044152.1	-	151	bacteria>proteobacteria>alphaproteobacteria	Sulfitobacter geojensis	host attachment protein [Sulfitobacter geojensis].
WP_027577243.1	-	151	bacteria>proteobacteria>alphaproteobacteria	Bradyrhizobium sp. WSM1743	host attachment protein [Bradyrhizobium sp. WSM1743].
WP_027795717.1	-	151	bacteria>proteobacteria>betaproteobacteria	Paraburkholderia acidipaludis	hypothetical protein [Paraburkholderia acidipaludis].
WP_028202093.1	-	151	bacteria>proteobacteria>betaproteobacteria	Paraburkholderia nodosa	hypothetical protein [Paraburkholderia nodosa].
WP_028209573.1	-	151	bacteria>proteobacteria>betaproteobacteria	Paraburkholderia mimosarum	hypothetical protein [Paraburkholderia mimosarum].
WP_028226648.1	-	151	bacteria>proteobacteria>betaproteobacteria	Paraburkholderia ferrariae	hypothetical protein [Paraburkholderia ferrariae].
WP_028231473.1	-	151	bacteria>proteobacteria>betaproteobacteria	Paraburkholderia mimosarum	hypothetical protein [Paraburkholderia mimosarum].
WP_028348802.1	-	151	bacteria>proteobacteria>alphaproteobacteria	Bradyrhizobium elkanii	hypothetical protein [Bradyrhizobium elkanii].
WP_028957263.1	-	151	bacteria>proteobacteria>alphaproteobacteria	Sulfitobacter sp. 20_GPM-1509m	host attachment protein [Sulfitobacter sp. 20_GPM-1509m].
WP_029041655.1	-	151	bacteria>proteobacteria>alphaproteobacteria	Cucumibacter marinus	host attachment protein [Cucumibacter marinus].
KEO53757.1	SMB34_06795	151	bacteria>proteobacteria>alphaproteobacteria	Thalassospira permensis NBRC 106175	hypothetical protein SMB34_06795 [Thalassospira permensis NBRC 106175].
WP_037063589.1	-	151	bacteria>proteobacteria>alphaproteobacteria	Rhizobium leguminosarum	hypothetical protein [Rhizobium leguminosarum].
WP_037924524.1	-	151	bacteria>proteobacteria>alphaproteobacteria	Sulfitobacter pseudonitzschiae	host attachment protein [Sulfitobacter pseudonitzschiae].
WP_038959235.1	-	151	bacteria>proteobacteria>alphaproteobacteria	Bradyrhizobium japonicum	host cell attachment protein [Bradyrhizobium japonicum].
WP_040042297.1	-	151	bacteria>proteobacteria>betaproteobacteria	Herbaspirillum sp. TSA66	hypothetical protein [Herbaspirillum sp. TSA66].
WP_042262296.1	-	151	bacteria>proteobacteria>betaproteobacteria	Paraburkholderia heleia	hypothetical protein [Paraburkholderia heleia].
WP_046115585.1	-	151	bacteria>proteobacteria>betaproteobacteria	Aquincola tertiaricarbonis	hypothetical protein [Aquincola tertiaricarbonis].
CEJ86522.1	HYPGJ_30525	151	bacteria>proteobacteria>alphaproteobacteria	Hyphomicrobium sp. GJ21	Host attachment protein [Hyphomicrobium sp. GJ21].
WP_050631646.1	-	151	bacteria>proteobacteria>alphaproteobacteria	Bradyrhizobium viridifuturi	host cell attachment protein [Bradyrhizobium viridifuturi].
WP_051544298.1	-	151	bacteria>proteobacteria>alphaproteobacteria	Acetobacter okinawensis	peptidase [Acetobacter okinawensis].
WP_052830764.1	-	151	bacteria>proteobacteria>alphaproteobacteria	Skermanella aerolata	hypothetical protein [Skermanella aerolata].
WP_056425362.1	-	151	bacteria>proteobacteria>alphaproteobacteria	Sphingomonas sp. Leaf33	host attachment protein [Sphingomonas sp. Leaf33].
WP_057847726.1	-	151	bacteria>proteobacteria>alphaproteobacteria	Bradyrhizobium retamae	hypothetical protein [Bradyrhizobium retamae].
WP_057851123.1	-	151	bacteria>proteobacteria>alphaproteobacteria	Bradyrhizobium valentinum	hypothetical protein [Bradyrhizobium valentinum].
WP_058261631.1	-	151	bacteria>proteobacteria>alphaproteobacteria	Thalassobius gelatinovorus	hypothetical protein [Thalassobius gelatinovorus].
KUJ85213.1	AVO45_17045	151	bacteria>proteobacteria>alphaproteobacteria	Ruegeria sp. ZGT118	hypothetical protein AVO45_17045 [Ruegeria sp. ZGT118].
KUO50972.1	APF82_07365	151	bacteria>proteobacteria>alphaproteobacteria	Sphingomonadales bacterium BRH_c42	hypothetical protein APF82_07365 [Sphingomonadales bacterium BRH_c42].
WP_060913093.1	-	151	bacteria>proteobacteria>alphaproteobacteria	Bradyrhizobium diazoefficiens	host cell attachment protein [Bradyrhizobium diazoefficiens].
KXS52295.1	AWU57_3325	151	bacteria>proteobacteria>gammaproteobacteria	Marinobacter sp. T13-3	host cell attachment-required protein [Marinobacter sp. T13-3].
WP_062953068.1	-	151	bacteria>proteobacteria>alphaproteobacteria	Thalassospira	MULTISPECIES: hypothetical protein [Thalassospira].
WP_063091861.1	-	151	bacteria>proteobacteria>alphaproteobacteria	Thalassospira	MULTISPECIES: hypothetical protein [Thalassospira].
KZM50470.1	OA90_08305	151	bacteria>proteobacteria>alphaproteobacteria	Labrenzia sp. OB1	Host attachment protein [Labrenzia sp. OB1].
WP_063805905.1	-	151	bacteria>proteobacteria>alphaproteobacteria	Rhizobium sp. BR10423	hypothetical protein [Rhizobium sp. BR10423].
WP_064223070.1	-	151	bacteria>proteobacteria>alphaproteobacteria	Sulfitobacter geojensis	Host attachment protein [Sulfitobacter geojensis].
WP_064312987.1	-	151	bacteria>proteobacteria>alphaproteobacteria	Sphingomonas sp. TDK1	hypothetical protein [Sphingomonas sp. TDK1].
WP_065057224.1	-	151	bacteria>proteobacteria>betaproteobacteria	Paraburkholderia tropica	hypothetical protein [Paraburkholderia tropica].
WP_065273484.1	-	151	bacteria>proteobacteria>alphaproteobacteria	Phaeobacter gallaeciensis	hypothetical protein [Phaeobacter gallaeciensis].
WP_065690779.1	-	151	bacteria>proteobacteria>alphaproteobacteria	Rhizobium sp. AC44/96	hypothetical protein [Rhizobium sp. AC44/96].
WP_065728283.1	-	151	bacteria>proteobacteria>alphaproteobacteria	Bradyrhizobium icense	Host attachment protein [Bradyrhizobium icense].
WP_065748378.1	-	151	bacteria>proteobacteria>alphaproteobacteria	Bradyrhizobium sp. LMTR 3	Host attachment protein [Bradyrhizobium sp. LMTR 3].
AAY61677.1	RF_0826	150	bacteria>proteobacteria>alphaproteobacteria	Rickettsia felis URRWXCal2	unknown [Rickettsia felis URRWXCal2].
ABV84927.1	RMA_0805	150	bacteria>proteobacteria>alphaproteobacteria	Rickettsia massiliae MTU5	AtsE-like protein [Rickettsia massiliae MTU5].
ABY72681.1	RrIowa_0844	150	bacteria>proteobacteria>alphaproteobacteria	Rickettsia rickettsii str. Iowa	hypothetical protein RrIowa_0844 [Rickettsia rickettsii str. Iowa].
ACP53585.1	RAF_ORF0695	150	bacteria>proteobacteria>alphaproteobacteria	Rickettsia africae ESF-5	AtsE-like protein [Rickettsia africae ESF-5].
AEM47136.1	Acife_0966	150	bacteria>proteobacteria	Acidithiobacillus ferrivorans SS3	Host attachment protein [Acidithiobacillus ferrivorans SS3].
BAK96775.1	RJP_0567	150	bacteria>proteobacteria>alphaproteobacteria	Rickettsia japonica YH	atsE-like protein [Rickettsia japonica YH].
EIM27016.1	MicloDRAFT_00035700	150	bacteria>proteobacteria>alphaproteobacteria	Microvirga lotononidis	protein required for attachment to host cells [Microvirga lotononidis].
EKD74745.1	ACD_44C00348G0001	150	bacteria	uncultured bacterium	hypothetical protein ACD_44C00348G0001 [uncultured bacterium].
WP_003535926.1	-	150	bacteria>proteobacteria>alphaproteobacteria	Sinorhizobium meliloti	host attachment protein [Sinorhizobium meliloti].
WP_006870194.1	-	150	bacteria>proteobacteria>gammaproteobacteria	Legionella drancourtii	hypothetical protein [Legionella drancourtii].
WP_006940352.1	-	150	bacteria>proteobacteria>alphaproteobacteria	Labrenzia aggregata	hypothetical protein [Labrenzia aggregata].
WP_007066454.1	-	150	bacteria>proteobacteria>alphaproteobacteria	Fulvimarina pelagi	hypothetical protein [Fulvimarina pelagi].
WP_009449374.1	-	150	bacteria>proteobacteria>alphaproteobacteria	Nitratireductor indicus	attachment protein [Nitratireductor indicus].
WP_009570265.1	-	150	bacteria>proteobacteria>alphaproteobacteria	Celeribacter baekdonensis	hypothetical protein [Celeribacter baekdonensis].
WP_010442028.1	-	150	bacteria>proteobacteria>alphaproteobacteria	Ruegeria conchae	attachment protein [Ruegeria conchae].
WP_010652940.1	-	150	bacteria>proteobacteria>gammaproteobacteria	Fluoribacter dumoffii	hypothetical protein [Fluoribacter dumoffii].
WP_010886301.1	-	150	bacteria>proteobacteria>alphaproteobacteria	Rickettsia prowazekii	hypothetical protein [Rickettsia prowazekii].
WP_011769619.1	-	150	bacteria>proteobacteria>gammaproteobacteria	Psychromonas ingrahamii	hypothetical protein [Psychromonas ingrahamii].
WP_012520326.1	-	150	bacteria>proteobacteria>alphaproteobacteria	Phenylobacterium zucineum	hypothetical protein [Phenylobacterium zucineum].
WP_012708918.1	-	150	bacteria>proteobacteria>alphaproteobacteria	Sinorhizobium fredii	hypothetical protein [Sinorhizobium fredii].

WP_013651001.1	-	150	bacteria>proteobacteria>alphaproteobacteria	Polymorphum gilvum	protein required for attachment to host cells-like protein [Polymorphum gilvum].
WP_014329204.1	-	150	bacteria>proteobacteria>alphaproteobacteria	Sinorhizobium fredii	hypothetical protein [Sinorhizobium fredii].
WP_014753305.1	-	150	bacteria>proteobacteria>alphaproteobacteria	Tistrella mobilis	hypothetical protein [Tistrella mobilis].
WP_014765420.1	-	150	bacteria>proteobacteria>alphaproteobacteria	Sinorhizobium fredii	hypothetical protein [Sinorhizobium fredii].
WP_016844687.1	-	150	bacteria>proteobacteria>alphaproteobacteria	Bradyrhizobium elkanii	hypothetical protein [Bradyrhizobium elkanii].
WP_017269933.1	-	150	bacteria>proteobacteria>alphaproteobacteria	Sinorhizobium meliloti	host attachment protein [Sinorhizobium meliloti].
WP_018098317.1	-	150	bacteria>proteobacteria>alphaproteobacteria	Sinorhizobium meliloti	host attachment protein [Sinorhizobium meliloti].
WP_018236527.1	-	150	bacteria>proteobacteria>alphaproteobacteria	Ensifer sp. BR816	host attachment protein [Ensifer sp. BR816].
WP_019233285.1	-	150	bacteria>proteobacteria>gammaproteobacteria	Legionella anisa	hypothetical protein [Legionella anisa].
WP_019936507.1	-	150	bacteria>proteobacteria>betaproteobacteria	Bordetella sp. FB-8	hypothetical protein [Bordetella sp. FB-8].
WP_020565769.1	-	150	bacteria>proteobacteria>gammaproteobacteria	Methylosarcina fibrata	hypothetical protein [Methylosarcina fibrata].
WP_023002960.1	-	150	bacteria>proteobacteria>alphaproteobacteria	Labrenzia	MULTISPECIES: host attachment protein [Labrenzia].
WP_023512043.1	-	150	bacteria>proteobacteria>alphaproteobacteria	Shinella	MULTISPECIES: hypothetical protein [Shinella].
WP_023686586.1	-	150	bacteria>proteobacteria>alphaproteobacteria	Mesorhizobium	MULTISPECIES: host attachment protein [Mesorhizobium].
WP_023700117.1	-	150	bacteria>proteobacteria>alphaproteobacteria	Mesorhizobium	MULTISPECIES: host attachment protein [Mesorhizobium].
WP_023737531.1	-	150	bacteria>proteobacteria>alphaproteobacteria	Mesorhizobium	MULTISPECIES: host attachment protein [Mesorhizobium].
WP_023751413.1	-	150	bacteria>proteobacteria>alphaproteobacteria	Mesorhizobium sp. LNJC380A00	host attachment protein [Mesorhizobium sp. LNJC380A00].
WP_024312158.1	-	150	bacteria>proteobacteria>alphaproteobacteria	Sinorhizobium	MULTISPECIES: host attachment protein [Sinorhizobium].
WP_024846504.1	-	150	bacteria>proteobacteria>alphaproteobacteria	Aminobacter sp. J41	host attachment protein [Aminobacter sp. J41].
WP_026618025.1	-	150	bacteria>proteobacteria>alphaproteobacteria	Sinorhizobium/Ensifer group	MULTISPECIES: host attachment protein [Sinorhizobium/Ensifer group].
WP_026868946.1	-	150	bacteria>proteobacteria>alphaproteobacteria	Inquilinus limosus	hypothetical protein [Inquilinus limosus].
WP_027238325.1	-	150	bacteria>proteobacteria>alphaproteobacteria	Pseudophaeobacter arcticus	hypothetical protein [Pseudophaeobacter arcticus].
WP_027816745.1	-	150	bacteria>proteobacteria>betaproteobacteria	Paraburkholderia bannensis	hypothetical protein [Paraburkholderia bannensis].
WP_027997317.1	-	150	bacteria>proteobacteria>alphaproteobacteria	Sinorhizobium arboris	host attachment protein [Sinorhizobium arboris].
WP_028032153.1	-	150	bacteria>proteobacteria>alphaproteobacteria	Chelativorans sp. J32	host attachment protein [Chelativorans sp. J32].
WP_028094100.1	-	150	bacteria>proteobacteria>alphaproteobacteria	Pseudodonghicola xiamenensis	host attachment protein [Pseudodonghicola xiamenensis].
WP_028220976.1	-	150	bacteria>proteobacteria>betaproteobacteria	Paraburkholderia oxyphila	hypothetical protein [Paraburkholderia oxyphila].
WP_028736195.1	-	150	bacteria>proteobacteria>alphaproteobacteria	Rhizobium selenitireducens	protein required for attachment to host cells [Rhizobium selenitireducens].
WP_029057928.1	-	150	bacteria>proteobacteria>alphaproteobacteria	Stappia stellulata	host attachment protein [Stappia stellulata].
WP_029961046.1	-	150	bacteria>proteobacteria>alphaproteobacteria	Ensifer sp. USDA 6670	host attachment protein [Ensifer sp. USDA 6670].
CDQ10928.1	AFERRI_430030	150	bacteria>proteobacteria	Acidithiobacillus ferrivorans	Host attachment protein [Acidithiobacillus ferrivorans].
WP_031563902.1	-	150	bacteria>proteobacteria>gammaproteobacteria	Legionella wadsworthii	hypothetical protein [Legionella wadsworthii].
WP_035365151.1	-	150	bacteria>proteobacteria>alphaproteobacteria	Dongia sp. URHE0060	hypothetical protein [Dongia sp. URHE0060].
WP_035526651.1	-	150	bacteria>proteobacteria>betaproteobacteria	Paraburkholderia sacchari	hypothetical protein [Paraburkholderia sacchari].
WP_037387508.1	-	150	bacteria>proteobacteria>alphaproteobacteria	Sinorhizobium	MULTISPECIES: host attachment protein [Sinorhizobium].
WP_038379070.1	-	150	bacteria>proteobacteria>alphaproteobacteria	Bradyrhizobium elkanii	host cell attachment protein [Bradyrhizobium elkanii].
WP_039529768.1	-	150	bacteria>proteobacteria>alphaproteobacteria	Ruegeria sp. ANG-S4	attachment protein [Ruegeria sp. ANG-S4].
WP_039534673.1	-	150	bacteria>proteobacteria>alphaproteobacteria	Ruegeria sp. ANG-R	attachment protein [Ruegeria sp. ANG-R].
WP_040960954.1	-	150	bacteria>proteobacteria>alphaproteobacteria	Sinorhizobium fredii	host attachment protein [Sinorhizobium fredii].
WP_050409618.1	-	150	bacteria>proteobacteria>betaproteobacteria	Massilia sp. NR 4-1	hypothetical protein [Massilia sp. NR 4-1].
WP_051134280.1	-	150	bacteria>proteobacteria>alphaproteobacteria	Methylocystis sp. ATCC 49242	host cell attachment protein [Methylocystis sp. ATCC 49242].
WP_051212419.1	-	150	bacteria>proteobacteria>alphaproteobacteria	Rubritepida flocculans	hypothetical protein [Rubritepida flocculans].
WP_055045202.1	-	150	bacteria>proteobacteria>alphaproteobacteria	Devosia sp. A16	hypothetical protein [Devosia sp. A16].
WP_055756495.1	-	150	bacteria>proteobacteria>alphaproteobacteria	Sphingomonas	MULTISPECIES: host attachment protein [Sphingomonas].
WP_055887878.1	-	150	bacteria>proteobacteria>alphaproteobacteria	Aureimonas sp. Leaf324	hypothetical protein [Aureimonas sp. Leaf324].
WP_055938152.1	-	150	bacteria>proteobacteria>betaproteobacteria	Duganella	MULTISPECIES: hypothetical protein [Duganella].
WP_055994377.1	-	150	bacteria>proteobacteria>alphaproteobacteria	Sphingomonas	MULTISPECIES: host attachment protein [Sphingomonas].
WP_056451675.1	-	150	bacteria>proteobacteria>alphaproteobacteria	Sphingomonas sp. Leaf10	host attachment protein [Sphingomonas sp. Leaf10].
WP_056494597.1	-	150	bacteria>proteobacteria>alphaproteobacteria	Sphingomonas sp. Leaf25	host attachment protein [Sphingomonas sp. Leaf25].
WP_056623410.1	-	150	bacteria>proteobacteria>alphaproteobacteria	Sphingomonas sp. Leaf62	host attachment protein [Sphingomonas sp. Leaf62].
WP_057264411.1	-	150	bacteria>proteobacteria>betaproteobacteria	Duganella sp. Root1480D1	hypothetical protein [Duganella sp. Root1480D1].
WP_057837111.1	-	150	bacteria>proteobacteria>alphaproteobacteria	Bradyrhizobium jicamae	hypothetical protein [Bradyrhizobium jicamae].
WP_057861400.1	-	150	bacteria>proteobacteria>alphaproteobacteria	Bradyrhizobium lablabi	hypothetical protein [Bradyrhizobium lablabi].
WP_058274058.1	-	150	bacteria>proteobacteria>alphaproteobacteria	Ruegeria atlantica	attachment protein [Ruegeria atlantica].
WP_058277460.1	-	150	bacteria>proteobacteria>alphaproteobacteria	Ruegeria atlantica	attachment protein [Ruegeria atlantica].
WP_058460735.1	-	150	bacteria>proteobacteria>gammaproteobacteria	Fluoribacter bozemanae	hypothetical protein [Fluoribacter bozemanae].
WP_058466853.1	-	150	bacteria>proteobacteria>gammaproteobacteria	Fluoribacter gormanii	hypothetical protein [Fluoribacter gormanii].
WP_058500309.1	-	150	bacteria>proteobacteria>gammaproteobacteria	Legionella gratiana	hypothetical protein [Legionella gratiana].
WP_058534025.1	-	150	bacteria>proteobacteria>gammaproteobacteria	Legionella sp. LH-SWC	hypothetical protein [Legionella sp. LH-SWC].
AMN38507.1	RHPLAN_00420	150	bacteria>proteobacteria>alphaproteobacteria	Rhodoplanes sp. Z2-YC6860	host attachment protein [Rhodoplanes sp. Z2-YC6860].
WP_062475476.1	-	150	bacteria>proteobacteria>gammaproteobacteria	Lacimicrobium alkaliphilum	hypothetical protein [Lacimicrobium alkaliphilum].
WP_062766309.1	-	150	bacteria>proteobacteria>alphaproteobacteria	Tistrella mobilis	hypothetical protein [Tistrella mobilis].
OAP46521.1	ATB98_14190	150	bacteria>proteobacteria>alphaproteobacteria	Sinorhizobium saheli	host attachment protein [Sinorhizobium saheli].
WP_064240113.1	-	150	bacteria>proteobacteria>alphaproteobacteria	Ensifer glycinis	host attachment protein [Ensifer glycinis].
WP_064330856.1	-	150	bacteria>proteobacteria>alphaproteobacteria	Shinella sp. HZN7	host attachment protein [Shinella sp. HZN7].
WP_064696277.1	-	150	bacteria>proteobacteria>alphaproteobacteria	Rhizobium aegyptiacum	hypothetical protein [Rhizobium aegyptiacum].
WP_064713611.1	-	150	bacteria>proteobacteria>alphaproteobacteria	Rhizobium bangladeshense	hypothetical protein [Rhizobium bangladeshense].
WP_065753201.1	-	150	bacteria>proteobacteria>alphaproteobacteria	Bradyrhizobium paxllaeri	Host attachment protein [Bradyrhizobium paxllaeri].
CCB66545.1	HYPMC_3331	149	bacteria>proteobacteria>alphaproteobacteria	Hyphomicrobium sp. MC1	conserved protein of unknown function [Hyphomicrobium sp. MC1].
WP_003600853.1	-	149	bacteria>proteobacteria>alphaproteobacteria	Methylobacterium	MULTISPECIES: host attachment protein [Methylobacterium].
WP_005768749.1	-	149	bacteria>proteobacteria>gammaproteobacteria	Coxiella burnetii	hypothetical protein [Coxiella burnetii].
WP_005982335.1	-	149	bacteria>proteobacteria>alphaproteobacteria	Ruegeria lacuscaerulensis	attachment protein [Ruegeria lacuscaerulensis].
WP_006200416.1	-	149	bacteria>proteobacteria>alphaproteobacteria	Mesorhizobium amorphae	host attachment protein [Mesorhizobium amorphae].
WP_006339232.1	-	149	bacteria>proteobacteria>alphaproteobacteria	Mesorhizobium sp. STM 4661	hypothetical protein [Mesorhizobium sp. STM 4661].
WP_006747731.1	-	149	bacteria>proteobacteria>gammaproteobacteria	Thioalkalivibrio paradoxus	host attachment protein [Thioalkalivibrio paradoxus].
WP_008123944.1	-	149	bacteria>proteobacteria>alphaproteobacteria	Phyllobacterium sp. YR531	hypothetical protein [Phyllobacterium sp. YR531].
WP_008874272.1	-	149	bacteria>proteobacteria>alphaproteobacteria	Mesorhizobium metallidurans	hypothetical protein [Mesorhizobium metallidurans].
WP_009517455.1	-	149	bacteria>proteobacteria>betaproteobacteria	Hydrogenophaga	MULTISPECIES: host attachment protein [Hydrogenophaga].
WP_009569165.1	-	149	unclassified	Bacteria	MULTISPECIES: hypothetical protein [Bacteria].
WP_010911517.1	-	149	bacteria>proteobacteria>alphaproteobacteria	Mesorhizobium loti	hypothetical protein [Mesorhizobium loti].
WP_011087415.1	-	149	bacteria>proteobacteria>alphaproteobacteria	Bradyrhizobium	MULTISPECIES: hypothetical protein [Bradyrhizobium].
WP_011636619.1	-	149	bacteria>proteobacteria>gammaproteobacteria	Shewanella frigidimarina	host cell attachment-required protein [Shewanella frigidimarina].
WP_012142912.1	-	149	bacteria>proteobacteria>gammaproteobacteria	Shewanella sediminis	host cell attachment-required protein [Shewanella sediminis].
WP_012254737.1	-	149	bacteria>proteobacteria>alphaproteobacteria	Methylobacterium	MULTISPECIES: hypothetical protein [Methylobacterium].
WP_012455715.1	-	149	bacteria>proteobacteria>alphaproteobacteria	Methylobacterium populi	hypothetical protein [Methylobacterium populi].
WP_013529560.1	-	149	bacteria>proteobacteria>alphaproteobacteria	Mesorhizobium	MULTISPECIES: host attachment protein [Mesorhizobium].
WP_013948930.1	-	149	bacteria>proteobacteria>alphaproteobacteria	Hyphomicrobium sp. MC1	hypothetical protein [Hyphomicrobium sp. MC1].
WP_014892009.1	-	149	bacteria>proteobacteria>alphaproteobacteria	Methylocystis sp. SC2	host attachment protein [Methylocystis sp. SC2].
WP_015315693.1	-	149	bacteria>proteobacteria>alphaproteobacteria	Mesorhizobium	MULTISPECIES: protein required for attachment to host cells [Mesorhizobium].
WP_015823644.1	-	149	bacteria>proteobacteria>alphaproteobacteria	Methylobacterium extorquens	hypothetical protein [Methylobacterium extorquens].
WP_015951827.1	-	149	bacteria>proteobacteria>alphaproteobacteria	Methylobacterium extorquens	hypothetical protein [Methylobacterium extorquens].
WP_017484670.1	-	149	bacteria>proteobacteria>alphaproteobacteria	Methylobacterium sp. MB200	host attachment protein [Methylobacterium sp. MB200].
WP_017941324.1	-	149	bacteria>proteobacteria>gammaproteobacteria	Thioalkalivibrio	MULTISPECIES: host attachment protein [Thioalkalivibrio].
WP_018137852.1	-	149	bacteria>proteobacteria>gammaproteobacteria	Thioalkalivibrio	MULTISPECIES: host attachment protein [Thioalkalivibrio].
WP_018141794.1	-	149	bacteria>proteobacteria>gammaproteobacteria	Thioalkalivibrio	MULTISPECIES: host attachment protein [Thioalkalivibrio].
WP_018146209.1	-	149	bacteria>proteobacteria>gammaproteobacteria	Thioalkalivibrio	MULTISPECIES: host attachment protein [Thioalkalivibrio].
WP_018184282.1	-	149	bacteria>proteobacteria>alphaproteobacteria	Kaistia granuli	hypothetical protein [Kaistia granuli].
WP_018862144.1	-	149	bacteria>proteobacteria>gammaproteobacteria	Thioalkalivibrio	MULTISPECIES: host attachment protein [Thioalkalivibrio].
WP_018865219.1	-	149	bacteria>proteobacteria>gammaproteobacteria	Thioalkalivibrio	MULTISPECIES: host attachment protein [Thioalkalivibrio].
WP_018867815.1	-	149	bacteria>proteobacteria>gammaproteobacteria	Thioalkalivibrio	MULTISPECIES: host attachment protein [Thioalkalivibrio].
WP_018870043.1	-	149	bacteria>proteobacteria>gammaproteobacteria	Thioalkalivibrio	MULTISPECIES: hypothetical protein [Thioalkalivibrio].
WP_018872395.1	-	149	bacteria>proteobacteria>gammaproteobacteria	Thioalkalivibrio sp. ALJ16	host attachment protein [Thioalkalivibrio sp. ALJ16].
WP_018934683.1	-	149	bacteria>proteobacteria>gammaproteobacteria	Thioalkalivibrio sp. ALJ24	hypothetical protein [Thioalkalivibrio sp. ALJ24].
WP_018940691.1	-	149	bacteria>proteobacteria>gammaproteobacteria	Thioalkalivibrio sp. AKL11	host attachment protein [Thioalkalivibrio sp. AKL11].
WP_018946430.1	-	149	bacteria>proteobacteria>gammaproteobacteria	Thioalkalivibrio sp. AKL17	host attachment protein [Thioalkalivibrio sp. AKL17].
WP_018948768.1	-	149	bacteria>proteobacteria>gammaproteobacteria	Thioalkalivibrio	MULTISPECIES: host attachment protein [Thioalkalivibrio].
WP_018995195.1	-	149	bacteria>proteobacteria>gammaproteobacteria	Thioalkalivibrio	MULTISPECIES: host attachment protein [Thioalkalivibrio].
WP_019140112.1	-	149	bacteria>proteobacteria>betaproteobacteria	Herbaspirillum massiliense	hypothetical protein [Herbaspirillum massiliense].
WP_019400250.1	-	149	bacteria>proteobacteria>alphaproteobacteria	Chelatococcus	MULTISPECIES: hypothetical protein [Chelatococcus].
WP_019569133.1	-	149	bacteria>proteobacteria>gammaproteobacteria	Thioalkalivibrio sp. ALE11	host attachment protein [Thioalkalivibrio sp. ALE11].
WP_019626291.1	-	149	bacteria>proteobacteria>gammaproteobacteria	Thioalkalivibrio sp. ALJT	host attachment protein [Thioalkalivibrio sp. ALJT].
WP_019861223.1	-	149	bacteria>proteobacteria>alphaproteobacteria	Mesorhizobium	MULTISPECIES: host attachment protein [Mesorhizobium].
WP_021003783.1	-	149	bacteria>proteobacteria>betaproteobacteria	Variovorax paradoxus	host attachment protein [Variovorax paradoxus].
WP_021244762.1	-	149	bacteria>proteobacteria>alphaproteobacteria	Sphingobium baderi	hypothetical protein [Sphingobium baderi].
WP_023432746.1	-	149	bacteria>proteobacteria>alphaproteobacteria	Lutibaculum baratangense	hypothetical protein [Lutibaculum baratangense].
WP_023669773.1	-	149	bacteria>proteobacteria>alphaproteobacteria	Mesorhizobium	MULTISPECIES: host attachment protein [Mesorhizobium].
WP_023675241.1	-	149	bacteria>proteobacteria>alphaproteobacteria	Mesorhizobium sp. LSJC280B00	host attachment protein [Mesorhizobium sp. LSJC280B00].
WP_023704214.1	-	149	bacteria>proteobacteria>alphaproteobacteria	Mesorhizobium	MULTISPECIES: host attachment protein [Mesorhizobium].
WP_023718936.1	-	149	bacteria>proteobacteria>alphaproteobacteria	Mesorhizobium sp. LSHC420B00	host attachment protein [Mesorhizobium sp. LSHC420B00].
WP_023726453.1	-	149	bacteria>proteobacteria>alphaproteobacteria	Mesorhizobium	MULTISPECIES: host attachment protein [Mesorhizobium].
WP_023760768.1	-	149	bacteria>proteobacteria>alphaproteobacteria	Mesorhizobium sp. LNHC252B00	host attachment protein [Mesorhizobium sp. LNHC252B00].
WP_023797422.1	-	149	bacteria>proteobacteria>alphaproteobacteria	Mesorhizobium sp. L48C026A00	host attachment protein [Mesorhizobium sp. L48C026A00].
WP_023812513.1	-	149	bacteria>proteobacteria>alphaproteobacteria	Mesorhizobium sp. L2C084A000	host attachment protein [Mesorhizobium sp. L2C084A000].
WP_023831679.1	-	149	bacteria>proteobacteria>alphaproteobacteria	Mesorhizobium sp. L103C119B0	host attachment protein [Mesorhizobium sp. L103C119B0].
WP_024326927.1	-	149	bacteria>proteobacteria>gammaproteobacteria	Thioalkalivibrio sp. AKL19	host attachment protein [Thioalkalivibrio sp. AKL19].
WP_024328563.1	-	149	bacteria>proteobacteria>gammaproteobacteria	Thioalkalivibrio sp. ALR17-21	host attachment protein [Thioalkalivibrio sp. ALR17-21].
WP_024502842.1	-	149	bacteria>proteobacteria>alphaproteobacteria	Mesorhizobium ciceri	host attachment protein [Mesorhizobium ciceri].
WP_024587897.1	-	149	bacteria>proteobacteria>alphaproteobacteria	Aliihoeflea sp. 2WW	host attachment protein [Aliihoeflea sp. 2WW].
WP_025428027.1	-	149	bacteria>proteobacteria>alphaproteobacteria	Ensifer	MULTISPECIES: host attachment protein [Ensifer].
WP_026222602.1	-	149	bacteria>proteobacteria>alphaproteobacteria	Methylocystis rosea	host cell attachment protein [Methylocystis rosea].
WP_026780574.1	-	149	bacteria>proteobacteria>betaproteobacteria	Polaromonas sp. EUR3 1.2.1	hypothetical protein [Polaromonas sp. EUR3 1.2.1].
WP_027023643.1	-	149	bacteria>proteobacteria>alphaproteobacteria	Mesorhizobium sp. URHA0056	host attachment protein [Mesorhizobium sp. URHA0056].
WP_027037733.1	-	149	bacteria>proteobacteria>alphaproteobacteria	Mesorhizobium ciceri	host attachment protein [Mesorhizobium ciceri].
WP_027043279.1	-	149	bacteria>proteobacteria>alphaproteobacteria	Mesorhizobium sp. URHC0008	host attachment protein [Mesorhizobium sp. URHC0008].
WP_027056601.1	-	149	bacteria>proteobacteria>alphaproteobacteria	Mesorhizobium	MULTISPECIES: host attachment protein [Mesorhizobium].
WP_027060228.1	-	149	bacteria>proteobacteria>alphaproteobacteria	Mesorhizobium loti	host attachment protein [Mesorhizobium loti].
WP_027143060.1	-	149	bacteria>proteobacteria>alphaproteobacteria	Mesorhizobium sp. WSM3626	host attachment protein [Mesorhizobium sp. WSM3626].
WP_027153079.1	-	149	bacteria>proteobacteria>alphaproteobacteria	Mesorhizobium sp. WSM2561	host attachment protein [Mesorhizobium sp. WSM2561].
WP_027158393.1	-	149	bacteria>proteobacteria>gammaproteobacteria	Methylobacter luteus	host attachment protein [Methylobacter luteus].
WP_027233263.1	-	149	bacteria>proteobacteria>alphaproteobacteria	Phyllobacterium sp. UNC302MFCol5.2	host attachment protein [Phyllobacterium sp. UNC302MFCol5.2].
WP_027288446.1	-	149	bacteria>proteobacteria>alphaproteobacteria	Rhodovibrio salinarum	hypothetical protein [Rhodovibrio salinarum].
WP_027315758.1	-	149	bacteria>proteobacteria>alphaproteobacteria	Microvirga flocculans	hypothetical protein [Microvirga flocculans].
WP_027520757.1	-	149	bacteria>proteobacteria>alphaproteobacteria	Bradyrhizobium sp. Ec3.3	host cell attachment protein [Bradyrhizobium sp. Ec3.3].
WP_028485123.1	-	149	bacteria>proteobacteria>gammaproteobacteria	Thioalkalivibrio sp. ALE17	host attachment protein [Thioalkalivibrio sp. ALE17].
WP_028492511.1	-	149	bacteria>proteobacteria>gammaproteobacteria	Thioalkalivibrio sp. ALE19	host attachment protein [Thioalkalivibrio sp. ALE19].
WP_029074123.1	-	149	bacteria>proteobacteria>alphaproteobacteria	Kaistia adipata	hypothetical protein [Kaistia adipata].
WP_029348209.1	-	149	bacteria>proteobacteria>alphaproteobacteria	Mesorhizobium ciceri	host attachment protein [Mesorhizobium ciceri].
WP_029651684.1	-	149	bacteria>proteobacteria>alphaproteobacteria	Methylocystis sp. SB2	host cell attachment protein [Methylocystis sp. SB2].
CDZ80835.1	BN1013_01358	149	bacteria>chlamydiae	Candidatus Rubidus massiliensis	Protein required for attachment to host cells [Candidatus Rubidus massiliensis].
WP_032114147.1	-	149	bacteria>proteobacteria>alphaproteobacteria	Candidatus Paracaedibacter symbiosus	hypothetical protein [Candidatus Paracaedibacter symbiosus].
WP_034789016.1	-	149	bacteria>proteobacteria>alphaproteobacteria	Ensifer	MULTISPECIES: host attachment protein [Ensifer].
WP_034851629.1	-	149	bacteria>proteobacteria>alphaproteobacteria	Ensifer sojae	host attachment protein [Ensifer sojae].
WP_035101144.1	-	149	bacteria>proteobacteria>alphaproteobacteria	Devosia sp. LC5	hypothetical protein [Devosia sp. LC5].
WP_035521661.1	-	149	bacteria>proteobacteria>alphaproteobacteria	Hoeflea sp. BAL378	host attachment protein [Hoeflea sp. BAL378].
WP_035689896.1	-	149	bacteria>proteobacteria>alphaproteobacteria	Bradyrhizobium elkanii	host cell attachment protein [Bradyrhizobium elkanii].
WP_036037626.1	-	149	bacteria>proteobacteria>alphaproteobacteria	Bradyrhizobium yuanmingense	host cell attachment protein [Bradyrhizobium yuanmingense].
WP_036486341.1	-	149	bacteria>proteobacteria>alphaproteobacteria	Nitratireductor basaltis	host attachment protein [Nitratireductor basaltis].
WP_037452981.1	-	149	bacteria>proteobacteria>alphaproteobacteria	Sphingobium chlorophenolicum	hypothetical protein [Sphingobium chlorophenolicum].
WP_038051393.1	-	149	bacteria>proteobacteria>gammaproteobacteria	Thioalkalivibrio sp. ALJ1	host attachment protein [Thioalkalivibrio sp. ALJ1].
WP_042526337.1	-	149	bacteria>proteobacteria>gammaproteobacteria	Coxiella burnetii	hypothetical protein [Coxiella burnetii].
WP_043623693.1	-	149	bacteria>proteobacteria>alphaproteobacteria	Ensifer sp. ZNC0028	host attachment protein [Ensifer sp. ZNC0028].
WP_043739460.1	-	149	bacteria>proteobacteria>gammaproteobacteria	Thioalkalivibrio nitratireducens	host attachment protein [Thioalkalivibrio nitratireducens].
KJS09085.1	VR78_16890	149	bacteria>proteobacteria>alphaproteobacteria	Hoeflea sp. BRH_c9	Host attachment protein [Hoeflea sp. BRH_c9].
WP_045835379.1	-	149	bacteria>proteobacteria>alphaproteobacteria	Hyphomicrobium sp. 99	hypothetical protein [Hyphomicrobium sp. 99].
WP_046107549.1	-	149	bacteria>proteobacteria>alphaproteobacteria	Devosia geojensis	hypothetical protein [Devosia geojensis].
WP_046134953.1	-	149	bacteria>proteobacteria>alphaproteobacteria	Devosia limi	hypothetical protein [Devosia limi].
WP_047030772.1	-	149	bacteria>proteobacteria>alphaproteobacteria	Hoeflea sp. IMCC20628	host attachment protein [Hoeflea sp. IMCC20628].
WP_047145354.1	-	149	bacteria>proteobacteria>alphaproteobacteria	Mesorhizobium sp. LC103	host attachment protein [Mesorhizobium sp. LC103].
WP_047188076.1	-	149	bacteria>proteobacteria>alphaproteobacteria	Microvirga vignae	hypothetical protein [Microvirga vignae].
WP_047786931.1	-	149	bacteria>proteobacteria>betaproteobacteria	Variovorax paradoxus	hypothetical protein [Variovorax paradoxus].
WP_048449849.1	-	149	bacteria>proteobacteria>alphaproteobacteria	Methylobacterium tarhaniae	host cell attachment protein [Methylobacterium tarhaniae].
WP_048575988.1	-	149	bacteria>proteobacteria>alphaproteobacteria	Sphingomonas sp. MEA3-1	hypothetical protein [Sphingomonas sp. MEA3-1].
WP_048645524.1	-	149	bacteria>proteobacteria>alphaproteobacteria	Nitratireductor sp. ZZ-1	host attachment protein [Nitratireductor sp. ZZ-1].
WP_051240648.1	-	149	bacteria>proteobacteria>betaproteobacteria	Tepidiphilus margaritifer	hypothetical protein [Tepidiphilus margaritifer].
WP_051961249.1	-	149	bacteria>proteobacteria>alphaproteobacteria	Devosia riboflavina	hypothetical protein [Devosia riboflavina].
WP_053248423.1	-	149	bacteria>proteobacteria>alphaproteobacteria	Ensifer adhaerens	host attachment protein [Ensifer adhaerens].
KPK14144.1	AMJ62_13730	149	bacteria>proteobacteria>deltaproteobacteria	Myxococcales bacterium SG8_38	hypothetical protein AMJ62_13730 [Myxococcales bacterium SG8_38].
KPQ08936.1	HLUCCO17_16735	149	bacteria>proteobacteria>alphaproteobacteria	Rhizobiales bacterium HL-109	hypothetical protein HLUCCO17_16735 [Rhizobiales bacterium HL-109].
WP_055423731.1	-	149	bacteria>proteobacteria>betaproteobacteria	Tepidiphilus thermophilus	hypothetical protein [Tepidiphilus thermophilus].
WP_055459903.1	-	149	bacteria>proteobacteria>alphaproteobacteria	Chelatococcus sambhunathii	host attachment protein [Chelatococcus sambhunathii].
WP_055686026.1	-	149	bacteria>proteobacteria>alphaproteobacteria	Loktanella sp. 5RATIMAR09	host attachment protein [Loktanella sp. 5RATIMAR09].
WP_055880175.1	-	149	bacteria>proteobacteria>alphaproteobacteria	Devosia	MULTISPECIES: hypothetical protein [Devosia].
WP_055981283.1	-	149	bacteria>proteobacteria>alphaproteobacteria	Aminobacter sp. Root100	host attachment protein [Aminobacter sp. Root100].
WP_056111795.1	-	149	bacteria>proteobacteria>alphaproteobacteria	Mesorhizobium	MULTISPECIES: host attachment protein [Mesorhizobium].
WP_056113548.1	-	149	bacteria>proteobacteria>alphaproteobacteria	Methylobacterium	MULTISPECIES: host cell attachment protein [Methylobacterium].
WP_056203559.1	-	149	bacteria>proteobacteria>alphaproteobacteria	Methylobacterium sp. Leaf123	host cell attachment protein [Methylobacterium sp. Leaf123].
WP_056253912.1	-	149	bacteria>proteobacteria>alphaproteobacteria	Devosia sp. Root436	hypothetical protein [Devosia sp. Root436].
WP_056277033.1	-	149	bacteria>proteobacteria>betaproteobacteria	Hydrogenophaga sp. Root209	hypothetical protein [Hydrogenophaga sp. Root209].
WP_056321673.1	-	149	bacteria>proteobacteria>alphaproteobacteria	Rhizobium	MULTISPECIES: host attachment protein [Rhizobium].
WP_056498160.1	-	149	bacteria>proteobacteria>alphaproteobacteria	Methylobacterium sp. Leaf121	host cell attachment protein [Methylobacterium sp. Leaf121].
WP_056577830.1	-	149	bacteria>proteobacteria>alphaproteobacteria	Mesorhizobium	MULTISPECIES: host attachment protein [Mesorhizobium].
WP_056595243.1	-	149	bacteria>proteobacteria>alphaproteobacteria	Mesorhizobium sp. Root102	host attachment protein [Mesorhizobium sp. Root102].
WP_056598729.1	-	149	bacteria>proteobacteria>betaproteobacteria	Variovorax sp. Root434	hypothetical protein [Variovorax sp. Root434].
WP_057144900.1	-	149	bacteria>proteobacteria>alphaproteobacteria	Mesorhizobium sp. Root695	host attachment protein [Mesorhizobium sp. Root695].
WP_057252966.1	-	149	bacteria>proteobacteria>alphaproteobacteria	Ensifer sp. Root278	host attachment protein [Ensifer sp. Root278].
KSV77995.1	N182_21795	149	bacteria>proteobacteria>alphaproteobacteria	Sinorhizobium sp. GL2	hypothetical protein N182_21795 [Sinorhizobium sp. GL2].
WP_058280660.1	-	149	bacteria>proteobacteria>alphaproteobacteria	Ruegeria sp. CECT 5091	attachment protein [Ruegeria sp. CECT 5091].
WP_058288122.1	-	149	bacteria>proteobacteria>alphaproteobacteria	Leisingera aquaemixtae	hypothetical protein [Leisingera aquaemixtae].
WP_058321185.1	-	149	bacteria>proteobacteria>alphaproteobacteria	Sinorhizobium/Ensifer group	MULTISPECIES: host attachment protein [Sinorhizobium/Ensifer group].
WP_058525274.1	-	149	bacteria>proteobacteria>gammaproteobacteria	Legionella erythra	hypothetical protein [Legionella erythra].
WP_058531154.1	-	149	bacteria>proteobacteria>gammaproteobacteria	Legionella rubrilucens	hypothetical protein [Legionella rubrilucens].
CUW38649.1	XM1_1580	149	bacteria>proteobacteria>alphaproteobacteria	Magnetospirillum sp. XM-1	conserved protein of unknown function (Host attachment protein,7-143) [Magnetospirillum sp. XM-1].
KUJ82179.1	AVO44_02615	149	bacteria>proteobacteria>alphaproteobacteria	Ruegeria sp. ZGT108	attachment protein [Ruegeria sp. ZGT108].
WP_060609349.1	-	149	bacteria>proteobacteria>alphaproteobacteria	Sinorhizobium sp. GW3	host attachment protein [Sinorhizobium sp. GW3].
WP_060770877.1	-	149	bacteria>proteobacteria>alphaproteobacteria	Methylobacterium sp. AMS5	host cell attachment protein [Methylobacterium sp. AMS5].
WP_062114840.1	-	149	bacteria>proteobacteria>alphaproteobacteria	Sphingobium sp. MI1205	hypothetical protein [Sphingobium sp. MI1205].
AMS39212.1	AA2016_0272	149	bacteria>proteobacteria>alphaproteobacteria	Aminobacter aminovorans	Host attachment protein [Aminobacter aminovorans].
SAK89382.1	AWB81_04739	149	bacteria>proteobacteria>betaproteobacteria	Burkholderia arationis	Protein required for attachment to host cells [Burkholderia arationis].
WP_063188357.1	-	149	bacteria>proteobacteria>alphaproteobacteria	Chelatococcus daeguensis	Host attachment protein [Chelatococcus daeguensis].
BAU92391.1	MPPM_3786	149	bacteria>proteobacteria>alphaproteobacteria	Methylobacterium populi	host attachment protein [Methylobacterium populi].
WP_064219093.1	-	149	bacteria>proteobacteria	Acidithiobacillus ferrooxidans	Host attachment protein [Acidithiobacillus ferrooxidans].
ANK82578.1	TEF_18590	149	bacteria>proteobacteria>alphaproteobacteria	Rhizobiales bacterium NRL2	hypothetical protein TEF_18590 [Rhizobiales bacterium NRL2].
WP_065010171.1	-	149	bacteria>proteobacteria>alphaproteobacteria	Mesorhizobium sp. AA22	Host attachment protein [Mesorhizobium sp. AA22].
WP_065267437.1	-	149	bacteria>proteobacteria>alphaproteobacteria	Leisingera sp. JC1	hypothetical protein [Leisingera sp. JC1].
WP_065413364.1	-	149	bacteria>proteobacteria	Acidithiobacillus ferrivorans	Host attachment protein [Acidithiobacillus ferrivorans].
OCC05301.1	BA190_10385	149	bacteria>proteobacteria>alphaproteobacteria	Labrys sp. WJW	Host attachment protein [Labrys sp. WJW].
WP_065775408.1	-	149	bacteria>proteobacteria>alphaproteobacteria	Ensifer	MULTISPECIES: host attachment protein [Ensifer].
WP_065786984.1	-	149	bacteria>proteobacteria>alphaproteobacteria	Ensifer	MULTISPECIES: host attachment protein [Ensifer].
WP_065794200.1	-	149	bacteria>proteobacteria>alphaproteobacteria	Ensifer sp. LC163	host attachment protein [Ensifer sp. LC163].
BAV47917.1	MLTONO_3014	149	bacteria>proteobacteria>alphaproteobacteria	Mesorhizobium loti	protein required for attachment to host cells [Mesorhizobium loti].
EAQ28274.1	NAP1_11783	148	bacteria>proteobacteria>alphaproteobacteria	Erythrobacter sp. NAP1	AtsE [Erythrobacter sp. NAP1].
EJC78597.1	Rleg4DRAFT_0164	148	bacteria>proteobacteria>alphaproteobacteria	Rhizobium leguminosarum bv. trifolii WSM2297	protein required for attachment to host cells [Rhizobium leguminosarum bv. trifolii WSM2297].
EKD72943.1	ACD_45C00507G0008	148	bacteria	uncultured bacterium	hypothetical protein ACD_45C00507G0008 [uncultured bacterium].
WP_003546882.1	-	148	bacteria>proteobacteria>alphaproteobacteria	Rhizobium leguminosarum	protein required for attachment to host cells [Rhizobium leguminosarum].
WP_003569233.1	-	148	bacteria>proteobacteria>alphaproteobacteria	Rhizobium leguminosarum	protein required for attachment to host cells [Rhizobium leguminosarum].
WP_003587623.1	-	148	bacteria>proteobacteria>alphaproteobacteria	Rhizobium leguminosarum	protein required for attachment to host cells [Rhizobium leguminosarum].
WP_004872337.1	-	148	bacteria>proteobacteria	Acidithiobacillus caldus	hypothetical protein [Acidithiobacillus caldus].
WP_005370161.1	-	148	bacteria>proteobacteria>gammaproteobacteria	Methylomicrobium album	protein required for attachment to host cells [Methylomicrobium album].
WP_005878450.1	-	148	bacteria>proteobacteria>betaproteobacteria	Oxalobacter formigenes	hypothetical protein [Oxalobacter formigenes].
WP_005882229.1	-	148	bacteria>proteobacteria>betaproteobacteria	Oxalobacter formigenes	hypothetical protein [Oxalobacter formigenes].
WP_006612573.1	-	148	bacteria>proteobacteria>alphaproteobacteria	Bradyrhizobium sp. ORS 285	hypothetical protein [Bradyrhizobium sp. ORS 285].
WP_007531131.1	-	148	bacteria>proteobacteria>alphaproteobacteria	Rhizobium mesoamericanum	hypothetical protein [Rhizobium mesoamericanum].
WP_007626563.1	-	148	bacteria>proteobacteria>alphaproteobacteria	Rhizobium sp. CCGE 510	hypothetical protein [Rhizobium sp. CCGE 510].
WP_007668139.1	-	148	bacteria>proteobacteria>alphaproteobacteria	Caulobacter sp. AP07	protein required for attachment to host cell [Caulobacter sp. AP07].
WP_007802975.1	-	148	bacteria>proteobacteria>alphaproteobacteria	Rhizobium sp. CF122	protein required for attachment to host cell [Rhizobium sp. CF122].
WP_007821673.1	-	148	bacteria>proteobacteria>alphaproteobacteria	Rhizobium sp. CF142	protein required for attachment to host cell [Rhizobium sp. CF142].
WP_008123122.1	-	148	bacteria>proteobacteria>alphaproteobacteria	Phyllobacterium sp. YR531	protein required for attachment to host cell [Phyllobacterium sp. YR531].
WP_008522583.1	-	148	bacteria>proteobacteria>alphaproteobacteria	Rhizobium sp. Pop5	hypothetical protein [Rhizobium sp. Pop5].
WP_008596196.1	-	148	bacteria>proteobacteria>alphaproteobacteria	Nitratireductor pacificus	protein required for attachment to host cells-like protein [Nitratireductor pacificus].
WP_008961728.1	-	148	bacteria>proteobacteria>alphaproteobacteria	Bradyrhizobium sp. STM 3809	hypothetical protein [Bradyrhizobium sp. STM 3809].
WP_008972826.1	-	148	bacteria>proteobacteria>alphaproteobacteria	Bradyrhizobium sp. STM 3843	hypothetical protein [Bradyrhizobium sp. STM 3843].
WP_009027570.1	-	148	bacteria>proteobacteria>alphaproteobacteria	Bradyrhizobium sp. ORS 375	hypothetical protein [Bradyrhizobium sp. ORS 375].
WP_009210304.1	-	148	bacteria>proteobacteria>alphaproteobacteria	Aurantimonas manganoxydans	hypothetical protein [Aurantimonas manganoxydans].
WP_009517967.1	-	148	bacteria>proteobacteria>betaproteobacteria	Hydrogenophaga	MULTISPECIES: hypothetical protein [Hydrogenophaga].
WP_010300168.1	-	148	bacteria>proteobacteria>alphaproteobacteria	Candidatus Odyssella thessalonicensis	hypothetical protein [Candidatus Odyssella thessalonicensis].
WP_011424673.1	-	148	bacteria>proteobacteria>alphaproteobacteria	Rhizobium etli	hypothetical protein [Rhizobium etli].
WP_011651181.1	-	148	bacteria>proteobacteria>alphaproteobacteria	Rhizobium leguminosarum	hypothetical protein [Rhizobium leguminosarum].
WP_011814453.1	-	148	bacteria>proteobacteria>gammaproteobacteria	Halorhodospira halophila	hypothetical protein [Halorhodospira halophila].
WP_011927169.1	-	148	bacteria>proteobacteria>alphaproteobacteria	Bradyrhizobium sp. ORS 278	hypothetical protein [Bradyrhizobium sp. ORS 278].
WP_011947290.1	-	148	bacteria>proteobacteria>gammaproteobacteria	Legionella pneumophila	hypothetical protein [Legionella pneumophila].
WP_012044980.1	-	148	bacteria>proteobacteria>alphaproteobacteria	Bradyrhizobium sp. BTAi1	hypothetical protein [Bradyrhizobium sp. BTAi1].
WP_012114192.1	-	148	bacteria>proteobacteria>alphaproteobacteria	Xanthobacter autotrophicus	hypothetical protein [Xanthobacter autotrophicus].
WP_012224863.1	-	148	bacteria>proteobacteria>alphaproteobacteria	Gluconacetobacter diazotrophicus	hypothetical protein [Gluconacetobacter diazotrophicus].
WP_012286483.1	-	148	bacteria>proteobacteria>alphaproteobacteria	Caulobacter sp. K31	hypothetical protein [Caulobacter sp. K31].
WP_012385642.1	-	148	bacteria>proteobacteria>alphaproteobacteria	Beijerinckia indica	hypothetical protein [Beijerinckia indica].
WP_012483243.1	-	148	bacteria>proteobacteria>alphaproteobacteria	Rhizobium	MULTISPECIES: hypothetical protein [Rhizobium].
WP_012557101.1	-	148	bacteria>proteobacteria>alphaproteobacteria	Rhizobium	MULTISPECIES: hypothetical protein [Rhizobium].
WP_012756763.1	-	148	bacteria>proteobacteria>alphaproteobacteria	Rhizobium leguminosarum	hypothetical protein [Rhizobium leguminosarum].
WP_012983044.1	-	148	bacteria>proteobacteria>gammaproteobacteria	Thioalkalivibrio sp. K90mix	host attachment protein [Thioalkalivibrio sp. K90mix].
WP_013101798.1	-	148	bacteria>proteobacteria>gammaproteobacteria	Legionella pneumophila	hypothetical protein [Legionella pneumophila].
WP_013420175.1	-	148	bacteria>proteobacteria>alphaproteobacteria	Rhodomicrobium vannielii	host attachment protein [Rhodomicrobium vannielii].
WP_014844542.1	-	148	bacteria>proteobacteria>gammaproteobacteria	Legionella pneumophila	hypothetical protein [Legionella pneumophila].
WP_014857844.1	-	148	bacteria>proteobacteria>alphaproteobacteria	Sinorhizobium/Ensifer group	MULTISPECIES: hypothetical protein [Sinorhizobium/Ensifer group].
WP_014857910.1	-	148	bacteria>proteobacteria>alphaproteobacteria	Sinorhizobium fredii	hypothetical protein [Sinorhizobium fredii].
WP_015666196.1	-	148	bacteria>proteobacteria>alphaproteobacteria	Bradyrhizobium oligotrophicum	hypothetical protein [Bradyrhizobium oligotrophicum].
WP_016553355.1	-	148	bacteria>proteobacteria>alphaproteobacteria	Rhizobium grahamii	hypothetical protein [Rhizobium grahamii].
WP_017926736.1	-	148	bacteria>proteobacteria>gammaproteobacteria	Thioalkalivibrio sp. HL-Eb18	host attachment protein [Thioalkalivibrio sp. HL-Eb18].
WP_018326837.1	-	148	bacteria>proteobacteria>alphaproteobacteria	Rhizobium giardinii	hypothetical protein [Rhizobium giardinii].
WP_018857452.1	-	148	bacteria>proteobacteria>alphaproteobacteria	Rhizobium sp. 42MFCr.1	protein required for attachment to host cells [Rhizobium sp. 42MFCr.1].
WP_019172783.1	-	148	bacteria>proteobacteria>alphaproteobacteria	Pseudaminobacter salicylatoxidans	hypothetical protein [Pseudaminobacter salicylatoxidans].
WP_020486064.1	-	148	bacteria>proteobacteria>alphaproteobacteria	Rhizobium leguminosarum	protein required for attachment to host cells [Rhizobium leguminosarum].
WP_020920831.1	-	148	bacteria>proteobacteria>alphaproteobacteria	Rhizobium etli	host attachment protein [Rhizobium etli].
EQB06299.1	L485_01360	148	bacteria>proteobacteria>alphaproteobacteria	Sphingobium baderi LL03	Host attachment protein [Sphingobium baderi LL03].
EQD24550.1	D084_Lepto4C00415G0006	148	bacteria>nitrospirae	Leptospirillum sp. Group IV 'UBA BS'	hypothetical protein D084_Lepto4C00415G0006 [Leptospirillum sp. Group IV 'UBA BS'].
WP_022714615.1	-	148	bacteria>proteobacteria>alphaproteobacteria	Rhizobium mongolense	protein required for attachment to host cells [Rhizobium mongolense].
KCZ91880.1	HJO_12202	148	bacteria>proteobacteria>alphaproteobacteria	Hyphomonas johnsonii MHS-2	attachment protein [Hyphomonas johnsonii MHS-2].
WP_024276808.1	-	148	bacteria>proteobacteria>alphaproteobacteria	Xanthobacter sp. 126	hypothetical protein [Xanthobacter sp. 126].
WP_024313717.1	-	148	bacteria>proteobacteria>alphaproteobacteria	Rhizobium favelukesii	protein required for attachment to host cells [Rhizobium favelukesii].
WP_024323750.1	-	148	bacteria>proteobacteria>alphaproteobacteria	Rhizobium leguminosarum	protein required for attachment to host cells [Rhizobium leguminosarum].
WP_024350365.1	-	148	bacteria>proteobacteria>alphaproteobacteria	Aurantimonas coralicida	host attachment protein [Aurantimonas coralicida].
WP_024811577.1	-	148	bacteria>proteobacteria>alphaproteobacteria	Oceanicola sp. HL-35	hypothetical protein [Oceanicola sp. HL-35].
WP_025029874.1	-	148	bacteria>proteobacteria>alphaproteobacteria	Nitratireductor aquibiodomus	host attachment protein [Nitratireductor aquibiodomus].
WP_025393907.1	-	148	bacteria>proteobacteria>alphaproteobacteria	Rhizobium leguminosarum	protein required for attachment to host cells [Rhizobium leguminosarum].
WP_025659880.1	-	148	bacteria>proteobacteria>alphaproteobacteria	Rhizobium sp. IBUN	protein required for attachment to host cells [Rhizobium sp. IBUN].
WP_026158605.1	-	148	bacteria>proteobacteria>alphaproteobacteria	Rhizobium leguminosarum	protein required for attachment to host cells [Rhizobium leguminosarum].
WP_026170816.1	-	148	bacteria>proteobacteria>alphaproteobacteria	Rhizobium leguminosarum	protein required for attachment to host cells [Rhizobium leguminosarum].
WP_026188334.1	-	148	bacteria>proteobacteria>alphaproteobacteria	Rhizobium	MULTISPECIES: protein required for attachment to host cells [Rhizobium].
WP_026236807.1	-	148	bacteria>proteobacteria>alphaproteobacteria	Rhizobium leguminosarum	protein required for attachment to host cells [Rhizobium leguminosarum].
WP_026238926.1	-	148	bacteria>proteobacteria>alphaproteobacteria	Rhizobium leguminosarum	protein required for attachment to host cells [Rhizobium leguminosarum].
WP_026265109.1	-	148	bacteria>proteobacteria>alphaproteobacteria	Rhizobium leguminosarum	protein required for attachment to host cells [Rhizobium leguminosarum].
WP_027147424.1	-	148	bacteria>proteobacteria>gammaproteobacteria	Methylobacter	MULTISPECIES: host attachment protein [Methylobacter].
WP_027268294.1	-	148	bacteria>proteobacteria>gammaproteobacteria	Legionella pneumophila	hypothetical protein [Legionella pneumophila].
WP_027288081.1	-	148	bacteria>proteobacteria>alphaproteobacteria	Rhodovibrio salinarum	hypothetical protein [Rhodovibrio salinarum].
WP_027507456.1	-	148	bacteria>proteobacteria>alphaproteobacteria	Rhizobium sullae	protein required for attachment to host cells [Rhizobium sullae].
WP_027582686.1	-	148	bacteria>proteobacteria>alphaproteobacteria	Bradyrhizobium sp. Ai1a-2	host cell attachment protein [Bradyrhizobium sp. Ai1a-2].
WP_027665200.1	-	148	bacteria>proteobacteria>alphaproteobacteria	Rhizobium leguminosarum	protein required for attachment to host cells [Rhizobium leguminosarum].
WP_027685320.1	-	148	bacteria>proteobacteria>alphaproteobacteria	Rhizobium leguminosarum	protein required for attachment to host cells [Rhizobium leguminosarum].
WP_028151315.1	-	148	bacteria>proteobacteria>alphaproteobacteria	Bradyrhizobium japonicum	host cell attachment protein [Bradyrhizobium japonicum].
WP_028153126.1	-	148	bacteria>proteobacteria>alphaproteobacteria	Bradyrhizobium japonicum	host cell attachment protein [Bradyrhizobium japonicum].
WP_028734295.1	-	148	bacteria>proteobacteria>alphaproteobacteria	Rhizobium leguminosarum	protein required for attachment to host cells [Rhizobium leguminosarum].
WP_028746041.1	-	148	bacteria>proteobacteria>alphaproteobacteria	Rhizobium mesoamericanum	protein required for attachment to host cells [Rhizobium mesoamericanum].
WP_028753448.1	-	148	bacteria>proteobacteria>alphaproteobacteria	Rhizobium leucaenae	protein required for attachment to host cells [Rhizobium leucaenae].
WP_028755926.1	-	148	bacteria>proteobacteria>alphaproteobacteria	Rhizobium	MULTISPECIES: protein required for attachment to host cells [Rhizobium].
WP_028990309.1	-	148	bacteria>proteobacteria	Thermithiobacillus tepidarius	hypothetical protein [Thermithiobacillus tepidarius].
WP_029004276.1	-	148	bacteria>proteobacteria>alphaproteobacteria	Azorhizobium doebereinerae	protein required for attachment to host cells [Azorhizobium doebereinerae].
WP_029556084.1	-	148	bacteria>proteobacteria>alphaproteobacteria	Xanthobacter sp. 91	hypothetical protein [Xanthobacter sp. 91].
WP_029871490.1	-	148	bacteria>proteobacteria>alphaproteobacteria	Rhizobium leguminosarum	protein required for attachment to host cells [Rhizobium leguminosarum].
WP_031431051.1	-	148	bacteria>proteobacteria>gammaproteobacteria	Methylomicrobium agile	host attachment protein [Methylomicrobium agile].
WP_033180697.1	-	148	bacteria>proteobacteria>alphaproteobacteria	Rhizobium leguminosarum	protein required for attachment to host cells [Rhizobium leguminosarum].
WP_034519019.1	-	148	bacteria>proteobacteria>alphaproteobacteria	Rhizobium/Agrobacterium group	MULTISPECIES: protein required for attachment to host cells [Rhizobium/Agrobacterium group].
WP_034839402.1	-	148	bacteria>proteobacteria>gammaproteobacteria	Endozoicomonas numazuensis	hypothetical protein [Endozoicomonas numazuensis].
WP_034993304.1	-	148	bacteria>proteobacteria>alphaproteobacteria	Beijerinckia mobilis	protein required for attachment to host cells [Beijerinckia mobilis].
WP_035891113.1	-	148	bacteria>proteobacteria>gammaproteobacteria	Legionella norrlandica	hypothetical protein [Legionella norrlandica].
WP_036250449.1	-	148	bacteria>proteobacteria>gammaproteobacteria	Methylobacter sp. BBA5.1	host attachment protein [Methylobacter sp. BBA5.1].
WP_037062095.1	-	148	bacteria>proteobacteria>alphaproteobacteria	Rhizobium leguminosarum	protein required for attachment to host cells [Rhizobium leguminosarum].
WP_037095723.1	-	148	bacteria>proteobacteria>alphaproteobacteria	Rhizobium alamii	protein required for attachment to host cells [Rhizobium alamii].
WP_037098918.1	-	148	bacteria>proteobacteria>alphaproteobacteria	Rhizobium sp. OK665	host attachment protein [Rhizobium sp. OK665].
WP_037117121.1	-	148	bacteria>proteobacteria>alphaproteobacteria	Rhizobium	MULTISPECIES: protein required for attachment to host cells [Rhizobium].
WP_037127060.1	-	148	bacteria>proteobacteria>alphaproteobacteria	Rhizobium sp. CF097	host attachment protein [Rhizobium sp. CF097].
WP_037134512.1	-	148	bacteria>proteobacteria>alphaproteobacteria	Rhizobium sp. CF394	protein required for attachment to host cells [Rhizobium sp. CF394].
WP_037165435.1	-	148	bacteria>proteobacteria>alphaproteobacteria	Rhizobium sp. CF258	host attachment protein [Rhizobium sp. CF258].
WP_037180373.1	-	148	bacteria>proteobacteria>alphaproteobacteria	Rhizobium sp. YR519	protein required for attachment to host cells [Rhizobium sp. YR519].
WP_037206712.1	-	148	bacteria>proteobacteria>alphaproteobacteria	Rhizobium sp. YR295	host attachment protein [Rhizobium sp. YR295].
WP_038692389.1	-	148	bacteria>proteobacteria>alphaproteobacteria	Rhizobium etli	protein required for attachment to host cells [Rhizobium etli].
WP_038958712.1	-	148	bacteria>proteobacteria>alphaproteobacteria	Bradyrhizobium japonicum	host cell attachment protein [Bradyrhizobium japonicum].
WP_039623153.1	-	148	bacteria>proteobacteria>alphaproteobacteria	Rhizobium etli	protein required for attachment to host cells [Rhizobium etli].
WP_039844393.1	-	148	bacteria>proteobacteria>alphaproteobacteria	Rhizobium gallicum	protein required for attachment to host cells [Rhizobium gallicum].
WP_040147821.1	-	148	bacteria>proteobacteria>gammaproteobacteria	Legionella pneumophila	hypothetical protein [Legionella pneumophila].
WP_042646268.1	-	148	bacteria>proteobacteria>gammaproteobacteria	Legionella pneumophila	hypothetical protein [Legionella pneumophila].
WP_043352661.1	-	148	unclassified	Bacteria	MULTISPECIES: host cell attachment protein [Bacteria].
WP_045095802.1	-	148	bacteria>proteobacteria>gammaproteobacteria	Legionella fallonii	hypothetical protein [Legionella fallonii].
WP_045099242.1	-	148	bacteria>proteobacteria>gammaproteobacteria	Tatlockia micdadei	hypothetical protein [Tatlockia micdadei].
CEJ14592.1	BN1110_04924	148	bacteria	bacterium YEK0313	Protein required for attachment to host cells [bacterium YEK0313].
WP_046903011.1	-	148	bacteria>proteobacteria>alphaproteobacteria	Altererythrobacter atlanticus	hypothetical protein [Altererythrobacter atlanticus].
WP_047464584.1	-	148	bacteria>proteobacteria>alphaproteobacteria	Agrobacterium rhizogenes	protein required for attachment to host cells [Agrobacterium rhizogenes].
WP_047621633.1	-	148	bacteria>proteobacteria>alphaproteobacteria	Rhizobium leguminosarum	protein required for attachment to host cells [Rhizobium leguminosarum].
WP_048860271.1	-	148	bacteria>proteobacteria>alphaproteobacteria	Acidisphaera rubrifaciens	hypothetical protein [Acidisphaera rubrifaciens].
WP_049733846.1	-	148	bacteria>proteobacteria>alphaproteobacteria	Rhizobium ecuadorense	hypothetical protein [Rhizobium ecuadorense].
WP_052036959.1	-	148	bacteria>proteobacteria>alphaproteobacteria	Rhodomicrobium udaipurense	hypothetical protein [Rhodomicrobium udaipurense].
KFP86685.1	HLUCCO07_14225	148	bacteria>proteobacteria>alphaproteobacteria	Rhodobacteraceae bacterium HLUCCO07	hypothetical protein HLUCCO07_14225 [Rhodobacteraceae bacterium HLUCCO07].
WP_056220105.1	-	148	bacteria>proteobacteria>alphaproteobacteria	Methylobacterium	MULTISPECIES: host cell attachment protein [Methylobacterium].
WP_056230299.1	-	148	bacteria>proteobacteria>alphaproteobacteria	Devosia sp. Root635	hypothetical protein [Devosia sp. Root635].
WP_056270483.1	-	148	bacteria>proteobacteria>alphaproteobacteria	Methylobacterium sp. Leaf88	host cell attachment protein [Methylobacterium sp. Leaf88].
WP_056334241.1	-	148	bacteria>proteobacteria>alphaproteobacteria	Rhizobium sp. Root482	host attachment protein [Rhizobium sp. Root482].
WP_056348488.1	-	148	bacteria>proteobacteria>alphaproteobacteria	Methylobacterium sp. Leaf89	host cell attachment protein [Methylobacterium sp. Leaf89].
WP_056382686.1	-	148	bacteria>proteobacteria>alphaproteobacteria	Methylobacterium sp. Leaf465	host cell attachment protein [Methylobacterium sp. Leaf465].
WP_056547685.1	-	148	bacteria>proteobacteria>alphaproteobacteria	Rhizobium sp. Root1220	hypothetical protein [Rhizobium sp. Root1220].
WP_056825372.1	-	148	bacteria>proteobacteria>alphaproteobacteria	Rhizobium sp. Root708	hypothetical protein [Rhizobium sp. Root708].
WP_056913410.1	-	148	bacteria>proteobacteria>alphaproteobacteria	Pseudolabrys sp. Root1462	host attachment protein [Pseudolabrys sp. Root1462].
WP_057185749.1	-	148	bacteria>proteobacteria>alphaproteobacteria	Caulobacter sp. Root656	protein required for attachment to host cell [Caulobacter sp. Root656].
WP_057471903.1	-	148	bacteria>proteobacteria>alphaproteobacteria	Rhizobium sp. Root1203	hypothetical protein [Rhizobium sp. Root1203].
WP_060505827.1	-	148	bacteria>proteobacteria>alphaproteobacteria	Rhizobium	MULTISPECIES: host attachment protein [Rhizobium].
WP_060637077.1	-	148	bacteria>proteobacteria>alphaproteobacteria	Rhizobium sp. Root483D2	host attachment protein [Rhizobium sp. Root483D2].
KXF79477.1	ATN84_01450	148	bacteria>proteobacteria>alphaproteobacteria	Paramesorhizobium deserti	Host attachment protein [Paramesorhizobium deserti].
BAU58164.1	HH1059_1854	148	bacteria>proteobacteria>gammaproteobacteria	Halorhodospira halochloris	hypothetical protein HH1059_1854 [Halorhodospira halochloris].
AMN39644.1	RHPLAN_11830	148	bacteria>proteobacteria>alphaproteobacteria	Rhodoplanes sp. Z2-YC6860	host cell attachment protein [Rhodoplanes sp. Z2-YC6860].
WP_061484638.1	-	148	bacteria>proteobacteria>gammaproteobacteria	Legionella pneumophila	hypothetical protein [Legionella pneumophila].
WP_061835443.1	-	148	bacteria>proteobacteria>deltaproteobacteria	Bdellovibrio bacteriovorus	hypothetical protein [Bdellovibrio bacteriovorus].
WP_062269107.1	-	148	bacteria>proteobacteria>gammaproteobacteria	Endozoicomonas sp. ab112	hypothetical protein [Endozoicomonas sp. ab112].
WP_062374632.1	-	148	bacteria>proteobacteria>alphaproteobacteria	Rhizobium sp. BR10423	hypothetical protein [Rhizobium sp. BR10423].
WP_062576785.1	-	148	bacteria>proteobacteria>alphaproteobacteria	Rhizobium	MULTISPECIES: host attachment protein [Rhizobium].
WP_062944225.1	-	148	bacteria>proteobacteria>alphaproteobacteria	Rhizobium leguminosarum	hypothetical protein [Rhizobium leguminosarum].
WP_063474329.1	-	148	bacteria>proteobacteria>alphaproteobacteria	Rhizobium sp. WYCCWR10015	hypothetical protein [Rhizobium sp. WYCCWR10015].
WP_063986311.1	-	148	bacteria>proteobacteria>alphaproteobacteria	Bradyrhizobium sp. G22	host cell attachment protein [Bradyrhizobium sp. G22].
WP_064250303.1	-	148	bacteria>proteobacteria>alphaproteobacteria	Rhizobium leguminosarum	hypothetical protein [Rhizobium leguminosarum].
WP_064654166.1	-	148	bacteria>proteobacteria>alphaproteobacteria	Rhizobium sp. WYCCWR10014	hypothetical protein [Rhizobium sp. WYCCWR10014].
WP_064682322.1	-	148	bacteria>proteobacteria>alphaproteobacteria	Rhizobium bangladeshense	hypothetical protein [Rhizobium bangladeshense].
WP_064692380.1	-	148	bacteria>proteobacteria>alphaproteobacteria	Rhizobium aegyptiacum	hypothetical protein [Rhizobium aegyptiacum].
WP_064706710.1	-	148	bacteria>proteobacteria>alphaproteobacteria	Rhizobium bangladeshense	hypothetical protein [Rhizobium bangladeshense].
WP_064806044.1	-	148	bacteria>proteobacteria>alphaproteobacteria	Rhizobium	MULTISPECIES: hypothetical protein [Rhizobium].
WP_064837830.1	-	148	bacteria>proteobacteria>alphaproteobacteria	Rhizobium	MULTISPECIES: hypothetical protein [Rhizobium].
WP_065282392.1	-	148	bacteria>proteobacteria>alphaproteobacteria	Rhizobium leguminosarum	hypothetical protein [Rhizobium leguminosarum].
OBZ93167.1	ADU59_22090	148	bacteria>proteobacteria>alphaproteobacteria	Pararhizobium polonicum	host attachment protein [Pararhizobium polonicum].
WP_065689967.1	-	148	bacteria>proteobacteria>alphaproteobacteria	Rhizobium sp. AC44/96	hypothetical protein [Rhizobium sp. AC44/96].
WP_065706462.1	-	148	bacteria>proteobacteria>alphaproteobacteria	Agrobacterium sp. 13-626	hypothetical protein [Agrobacterium sp. 13-626].
WP_065748036.1	-	148	bacteria>proteobacteria>alphaproteobacteria	Bradyrhizobium sp. LMTR 3	host attachment protein [Bradyrhizobium sp. LMTR 3].
WP_065817439.1	-	148	bacteria>proteobacteria>alphaproteobacteria	Nitratireductor aquibiodomus	Host attachment protein [Nitratireductor aquibiodomus].
EHQ06064.1	Lepil_1374	147	bacteria>spirochaetes	Leptonema illini DSM 21528	Host attachment protein [Leptonema illini DSM 21528].
EKD54855.1	ACD_60C00038G0017	147	bacteria	uncultured bacterium	hypothetical protein ACD_60C00038G0017 [uncultured bacterium].
WP_002155224.1	-	147	bacteria>spirochaetes	Leptospira noguchii	host cell attachment protein [Leptospira noguchii].
WP_002180414.1	-	147	bacteria>spirochaetes	Leptospira	MULTISPECIES: host cell attachment protein [Leptospira].
WP_002723775.1	-	147	bacteria>spirochaetes	Leptospira borgpetersenii	host cell attachment protein [Leptospira borgpetersenii].
WP_002735509.1	-	147	bacteria>spirochaetes	Leptospira	MULTISPECIES: host cell attachment protein [Leptospira].
WP_002764708.1	-	147	bacteria>spirochaetes	Leptospira mayottensis	host cell attachment protein [Leptospira mayottensis].
WP_006893070.1	-	147	bacteria>proteobacteria>gammaproteobacteria	Methylobacter tundripaludum	host attachment protein [Methylobacter tundripaludum].
WP_006893072.1	-	147	bacteria>proteobacteria>gammaproteobacteria	Methylobacter tundripaludum	host attachment protein [Methylobacter tundripaludum].
WP_007510948.1	-	147	bacteria>proteobacteria>gammaproteobacteria	Rhodanobacter denitrificans	host cell attachment-required protein [Rhodanobacter denitrificans].
WP_008870483.1	-	147	bacteria>proteobacteria>deltaproteobacteria	Desulfonatronospira thiodismutans	host attachment protein [Desulfonatronospira thiodismutans].
WP_010127633.1	-	147	bacteria>proteobacteria>alphaproteobacteria	Sphingomonas sp. KC8	hypothetical protein [Sphingomonas sp. KC8].
WP_010575742.1	-	147	bacteria>spirochaetes	Leptospira kmetyi	hypothetical protein [Leptospira kmetyi].
WP_011481380.1	-	147	bacteria>proteobacteria>betaproteobacteria	Polaromonas sp. JS666	hypothetical protein [Polaromonas sp. JS666].
WP_011670882.1	-	147	bacteria>spirochaetes	Leptospira borgpetersenii	hypothetical protein [Leptospira borgpetersenii].
WP_012523344.1	-	147	bacteria>proteobacteria>alphaproteobacteria	Phenylobacterium zucineum	hypothetical protein [Phenylobacterium zucineum].
WP_013892977.1	-	147	bacteria>proteobacteria>alphaproteobacteria	Mesorhizobium opportunistum	host attachment protein [Mesorhizobium opportunistum].
WP_014857854.1	-	147	bacteria>proteobacteria>alphaproteobacteria	Sinorhizobium/Ensifer group	MULTISPECIES: hypothetical protein [Sinorhizobium/Ensifer group].
WP_015446785.1	-	147	bacteria>proteobacteria>gammaproteobacteria	Rhodanobacter	MULTISPECIES: protein required for attachment to host cells [Rhodanobacter].
WP_015460186.1	-	147	bacteria>proteobacteria>alphaproteobacteria	Sphingomonas sp. MM-1	hypothetical protein [Sphingomonas sp. MM-1].
WP_018749221.1	-	147	bacteria>proteobacteria>betaproteobacteria	Chitiniphilus shinanonensis	hypothetical protein [Chitiniphilus shinanonensis].
WP_018898832.1	-	147	bacteria>proteobacteria>alphaproteobacteria	Rhizobium sp. 2MFCol3.1	hypothetical protein [Rhizobium sp. 2MFCol3.1].
WP_019556106.1	-	147	bacteria>proteobacteria>gammaproteobacteria	Thiomicrospira arctica	hypothetical protein [Thiomicrospira arctica].
WP_019865363.1	-	147	bacteria>proteobacteria>gammaproteobacteria	Methylovulum miyakonense	hypothetical protein [Methylovulum miyakonense].
WP_020087128.1	-	147	bacteria>proteobacteria>alphaproteobacteria	Hyphomicrobium zavarzinii	hypothetical protein [Hyphomicrobium zavarzinii].
WP_020774782.1	-	147	bacteria>spirochaetes	Leptospira alstonii	host cell attachment protein [Leptospira alstonii].
WP_021698194.1	-	147	bacteria>proteobacteria>alphaproteobacteria	Brevundimonas abyssalis	hypothetical protein [Brevundimonas abyssalis].
WP_022945874.1	-	147	bacteria>proteobacteria>gammaproteobacteria	Pseudoalteromonas ruthenica	hypothetical protein [Pseudoalteromonas ruthenica].
WP_023494459.1	-	147	bacteria>proteobacteria>gammaproteobacteria	Methyloglobulus morosus	host attachment protein [Methyloglobulus morosus].
WP_027149217.1	-	147	bacteria>proteobacteria>gammaproteobacteria	Methylobacter tundripaludum	host attachment protein [Methylobacter tundripaludum].
WP_027149219.1	-	147	bacteria>proteobacteria>gammaproteobacteria	Methylobacter tundripaludum	host attachment protein [Methylobacter tundripaludum].
WP_027369843.1	-	147	bacteria>proteobacteria>deltaproteobacteria	Desulfovermiculus halophilus	hypothetical protein [Desulfovermiculus halophilus].
WP_028133234.1	-	147	bacteria>proteobacteria>alphaproteobacteria	Bradyrhizobium japonicum	host cell attachment protein [Bradyrhizobium japonicum].
WP_028486452.1	-	147	bacteria>proteobacteria>gammaproteobacteria	Thiomicrospira chilensis	hypothetical protein [Thiomicrospira chilensis].
WP_031250270.1	-	147	bacteria>proteobacteria>alphaproteobacteria	Mesorhizobium	MULTISPECIES: host attachment protein [Mesorhizobium].
WP_031437839.1	-	147	bacteria>proteobacteria>gammaproteobacteria	Methylobacter tundripaludum	host attachment protein [Methylobacter tundripaludum].
WP_031437841.1	-	147	bacteria>proteobacteria>gammaproteobacteria	Methylobacter tundripaludum	host attachment protein [Methylobacter tundripaludum].
WP_033920038.1	-	147	bacteria>proteobacteria>alphaproteobacteria	Sphingomonas sp. 37zxx	host attachment protein [Sphingomonas sp. 37zxx].
WP_037153703.1	-	147	bacteria>proteobacteria>alphaproteobacteria	Rhizobium sp. YS-1r	hypothetical protein [Rhizobium sp. YS-1r].
WP_037199508.1	-	147	bacteria>proteobacteria>alphaproteobacteria	Rhizobium sp. OK494	hypothetical protein [Rhizobium sp. OK494].
WP_037200766.1	-	147	bacteria>proteobacteria>alphaproteobacteria	Rhizobium	MULTISPECIES: hypothetical protein [Rhizobium].
WP_038036957.1	-	147	bacteria>proteobacteria>betaproteobacteria	Thermopetrobacter sp. TC1	hypothetical protein [Thermopetrobacter sp. TC1].
WP_038464652.1	-	147	bacteria>proteobacteria>alphaproteobacteria	Candidatus Paracaedibacter acanthamoebae	hypothetical protein [Candidatus Paracaedibacter acanthamoebae].
WP_039718399.1	-	147	bacteria>cyanobacteria	Scytonema millei	hypothetical protein [Scytonema millei].
WP_044331891.1	-	147	bacteria>proteobacteria>alphaproteobacteria	Sphingomonas hengshuiensis	host attachment protein [Sphingomonas hengshuiensis].
WP_045978080.1	-	147	bacteria>proteobacteria>gammaproteobacteria	Pseudoalteromonas ruthenica	host attachment protein [Pseudoalteromonas ruthenica].
WP_047168721.1	-	147	bacteria>proteobacteria>alphaproteobacteria	Sphingomonas sp. Y57	host attachment protein [Sphingomonas sp. Y57].
WP_047513517.1	-	147	bacteria>proteobacteria>alphaproteobacteria	Rhizobium sp. YR528	protein required for attachment to host cells [Rhizobium sp. YR528].
WP_047560313.1	-	147	bacteria>proteobacteria>alphaproteobacteria	Rhizobium leguminosarum	hypothetical protein [Rhizobium leguminosarum].
WP_047640475.1	-	147	bacteria>proteobacteria>alphaproteobacteria	Rhizobium tropici	protein required for attachment to host cells [Rhizobium tropici].
WP_047640847.1	-	147	bacteria>proteobacteria>alphaproteobacteria	Rhizobium tropici	hypothetical protein [Rhizobium tropici].
WP_049622367.1	-	147	bacteria>proteobacteria>gammaproteobacteria	Dyella-like sp. DHo	hypothetical protein [Dyella-like sp. DHo].
WP_051698844.1	-	147	bacteria>proteobacteria>alphaproteobacteria	Erythrobacter longus	hypothetical protein [Erythrobacter longus].
WP_053911571.1	-	147	bacteria>proteobacteria>gammaproteobacteria	Pseudoalteromonas sp. SW0106-04	hypothetical protein [Pseudoalteromonas sp. SW0106-04].
KPK12350.1	AMJ68_02355	147	bacteria>proteobacteria	Acidithiobacillales bacterium SG8_45	hypothetical protein AMJ68_02355 [Acidithiobacillales bacterium SG8_45].
WP_058445155.1	-	147	bacteria>proteobacteria>gammaproteobacteria	Legionella feeleii	hypothetical protein [Legionella feeleii].
WP_058452840.1	-	147	bacteria>proteobacteria>gammaproteobacteria	Legionella maceachernii	hypothetical protein [Legionella maceachernii].
KUO54404.1	APF80_08180	147	bacteria>proteobacteria>alphaproteobacteria	Alphaproteobacteria bacterium BRH_c36	hypothetical protein APF80_08180 [Alphaproteobacteria bacterium BRH_c36].
KWT65405.1	APY04_2867	147	bacteria>proteobacteria>alphaproteobacteria	Hyphomicrobium sulfonivorans	hypothetical protein APY04_2867 [Hyphomicrobium sulfonivorans].
BAU48688.1	SVA_2138	147	bacteria>proteobacteria>gammaproteobacteria	Sulfurifustis variabilis	host cell attachment-required protein [Sulfurifustis variabilis].
WP_061219080.1	-	147	bacteria>spirochaetes	Leptospira weilii	host cell attachment protein [Leptospira weilii].
WP_061221612.1	-	147	bacteria>spirochaetes	Leptospira borgpetersenii	host cell attachment protein [Leptospira borgpetersenii].
WP_061223217.1	-	147	bacteria>spirochaetes	Leptospira weilii	host cell attachment protein [Leptospira weilii].
AMO72093.1	AZE99_09765	147	bacteria>proteobacteria>alphaproteobacteria	Sphingorhabdus sp. M41	hypothetical protein AZE99_09765 [Sphingorhabdus sp. M41].
WP_062537500.1	-	147	bacteria>proteobacteria>gammaproteobacteria	Mizugakiibacter sediminis	hypothetical protein [Mizugakiibacter sediminis].
AMV46423.1	ATN79_31240	147	bacteria>proteobacteria>betaproteobacteria	Paraburkholderia caribensis	hypothetical protein ATN79_31240 [Paraburkholderia caribensis].
OAM79203.1	A3840_03810	147	bacteria>proteobacteria>alphaproteobacteria	Devosia sp. S37	hypothetical protein A3840_03810 [Devosia sp. S37].
OAO04584.1	A8B75_07050	147	bacteria>proteobacteria>alphaproteobacteria	Sphingomonadales bacterium EhC05	hypothetical protein A8B75_07050 [Sphingomonadales bacterium EhC05].
ACR47581.1	RPR_04585	146	bacteria>proteobacteria>alphaproteobacteria	Rickettsia peacockii str. Rustic	hypothetical protein RPR_04585 [Rickettsia peacockii str. Rustic].
EKD45521.1	ACD_69C00255G0002	146	bacteria	uncultured bacterium	hypothetical protein ACD_69C00255G0002 [uncultured bacterium].
EKD71309.1	ACD_46C00219G0005	146	bacteria	uncultured bacterium	hypothetical protein ACD_46C00219G0005 [uncultured bacterium].
EKE10572.1	ACD_16C00001G0003	146	bacteria	uncultured bacterium	hypothetical protein ACD_16C00001G0003 [uncultured bacterium].
WP_000792514.1	-	146	bacteria>spirochaetes	Leptospira interrogans	hypothetical protein [Leptospira interrogans].
WP_000792515.1	-	146	bacteria>spirochaetes	Leptospira	MULTISPECIES: hypothetical protein [Leptospira].
WP_000792518.1	-	146	bacteria>spirochaetes	Leptospira interrogans	hypothetical protein [Leptospira interrogans].
EMD83943.1	C725_0915	146	bacteria>proteobacteria>alphaproteobacteria	alpha proteobacterium JLT2015	hypothetical protein C725_0915 [alpha proteobacterium JLT2015].
WP_002188338.1	-	146	bacteria>spirochaetes	Leptospira interrogans	host cell attachment protein [Leptospira interrogans].
WP_002635632.1	-	146	bacteria>proteobacteria>deltaproteobacteria	Myxococcus hansupus	hypothetical protein [Myxococcus hansupus].
WP_002928963.1	-	146	bacteria>proteobacteria>betaproteobacteria	Thauera	MULTISPECIES: hypothetical protein [Thauera].
WP_004211399.1	-	146	bacteria>proteobacteria>alphaproteobacteria	Sphingobium yanoikuyae	hypothetical protein [Sphingobium yanoikuyae].
WP_004274126.1	-	146	bacteria>proteobacteria>alphaproteobacteria	Nitrospirillum amazonense	hypothetical protein [Nitrospirillum amazonense].
WP_004302099.1	-	146	bacteria>proteobacteria>betaproteobacteria	Thauera	MULTISPECIES: hypothetical protein [Thauera].
WP_004337964.1	-	146	bacteria>proteobacteria>betaproteobacteria	Thauera linaloolentis	hypothetical protein [Thauera linaloolentis].
WP_004372236.1	-	146	bacteria>proteobacteria>betaproteobacteria	Thauera phenylacetica	hypothetical protein [Thauera phenylacetica].
WP_004782728.1	-	146	bacteria>spirochaetes	Leptospira kirschneri	host cell attachment protein [Leptospira kirschneri].
WP_004998294.1	-	146	bacteria>proteobacteria>alphaproteobacteria	Rickettsia	MULTISPECIES: hypothetical protein [Rickettsia].
WP_006966552.1	-	146	bacteria>proteobacteria>alphaproteobacteria	Sphingobium japonicum	hypothetical protein [Sphingobium japonicum].
WP_007684014.1	-	146	bacteria>proteobacteria>alphaproteobacteria	Sphingomonadaceae	MULTISPECIES: protein required for attachment to host cells [Sphingomonadaceae].
WP_009205640.1	-	146	bacteria>proteobacteria>betaproteobacteria	Sulfuricella denitrificans	hypothetical protein [Sulfuricella denitrificans].
WP_009821354.1	-	146	bacteria>proteobacteria>alphaproteobacteria	Sphingomonas sp. SKA58	hypothetical protein [Sphingomonas sp. SKA58].
WP_010183975.1	-	146	bacteria>proteobacteria>alphaproteobacteria	Sphingomonas sp. PAMC 26605	hypothetical protein [Sphingomonas sp. PAMC 26605].
WP_010422898.1	-	146	bacteria>proteobacteria>alphaproteobacteria	Rickettsia helvetica	hypothetical protein [Rickettsia helvetica].
WP_010626252.1	-	146	bacteria>proteobacteria>gammaproteobacteria	Halomonas sp. KM-1	host cell attachment-required protein [Halomonas sp. KM-1].
WP_011175471.1	-	146	bacteria>chlamydiae	Candidatus Protochlamydia amoebophila	hypothetical protein [Candidatus Protochlamydia amoebophila].
WP_011190915.1	-	146	bacteria>proteobacteria>alphaproteobacteria	Rickettsia typhi	hypothetical protein [Rickettsia typhi].
WP_011239513.1	-	146	bacteria>proteobacteria>betaproteobacteria	Aromatoleum aromaticum	hypothetical protein [Aromatoleum aromaticum].
WP_011381426.1	-	146	bacteria>proteobacteria>betaproteobacteria	Nitrosospira multiformis	hypothetical protein [Nitrosospira multiformis].
WP_011484271.1	-	146	bacteria>proteobacteria>betaproteobacteria	Polaromonas sp. JS666	hypothetical protein [Polaromonas sp. JS666].
WP_011551000.1	-	146	bacteria>proteobacteria>deltaproteobacteria	Myxococcus xanthus	hypothetical protein [Myxococcus xanthus].
WP_012148625.1	-	146	bacteria>proteobacteria>alphaproteobacteria	Rickettsia canadensis	hypothetical protein [Rickettsia canadensis].
WP_012149683.1	-	146	bacteria>proteobacteria>alphaproteobacteria	Rickettsia akari	hypothetical protein [Rickettsia akari].
WP_013846218.1	-	146	bacteria>proteobacteria>alphaproteobacteria	Sphingobium chlorophenolicum	host attachment protein [Sphingobium chlorophenolicum].
WP_013935956.1	-	146	bacteria>proteobacteria>deltaproteobacteria	Myxococcus fulvus	hypothetical protein [Myxococcus fulvus].
WP_014014417.1	-	146	bacteria>proteobacteria>alphaproteobacteria	Rickettsia heilongjiangensis	hypothetical protein [Rickettsia heilongjiangensis].
WP_014076096.1	-	146	bacteria>proteobacteria>alphaproteobacteria	Sphingobium sp. SYK-6	hypothetical protein [Sphingobium sp. SYK-6].
WP_014109754.1	-	146	bacteria>proteobacteria>gammaproteobacteria	Glaciecola nitratireducens	hypothetical protein [Glaciecola nitratireducens].
WP_014363857.1	-	146	bacteria>proteobacteria>alphaproteobacteria	Rickettsia canadensis	hypothetical protein [Rickettsia canadensis].
WP_014365721.1	-	146	bacteria>proteobacteria>alphaproteobacteria	spotted fever group	MULTISPECIES: hypothetical protein [spotted fever group].
WP_014392448.1	-	146	bacteria>proteobacteria>alphaproteobacteria	Candidatus Rickettsia amblyommii	hypothetical protein [Candidatus Rickettsia amblyommii].
WP_014408731.1	-	146	bacteria>proteobacteria>alphaproteobacteria	Rickettsia rhipicephali	hypothetical protein [Rickettsia rhipicephali].
WP_014409288.1	-	146	bacteria>proteobacteria>alphaproteobacteria	Rickettsia montanensis	hypothetical protein [Rickettsia montanensis].
WP_014412664.1	-	146	bacteria>proteobacteria>alphaproteobacteria	Rickettsia australis	hypothetical protein [Rickettsia australis].
WP_015346497.1	-	146	bacteria>proteobacteria>deltaproteobacteria	Myxococcus stipitatus	hypothetical protein [Myxococcus stipitatus].
WP_015436369.1	-	146	bacteria>proteobacteria>betaproteobacteria	Azoarcus sp. KH32C	hypothetical protein [Azoarcus sp. KH32C].
WP_016751934.1	-	146	bacteria>spirochaetes	Leptospira kirschneri	protein required for attachment to host cells [Leptospira kirschneri].
WP_017184716.1	-	146	bacteria>proteobacteria>alphaproteobacteria	Sphingobium xenophagum	host attachment protein [Sphingobium xenophagum].
WP_017442880.1	-	146	bacteria>proteobacteria>alphaproteobacteria	Candidatus Rickettsia gravesii	hypothetical protein [Candidatus Rickettsia gravesii].
WP_018989964.1	-	146	bacteria>proteobacteria>betaproteobacteria	Azoarcus toluclasticus	hypothetical protein [Azoarcus toluclasticus].
WP_019231447.1	-	146	bacteria>proteobacteria>alphaproteobacteria	Rickettsiaceae bacterium Os18	hypothetical protein [Rickettsiaceae bacterium Os18].
WP_019833209.1	-	146	bacteria>proteobacteria>alphaproteobacteria	Sphingomonas sp. PR090111-T3T-6A	hypothetical protein [Sphingomonas sp. PR090111-T3T-6A].
WP_020818928.1	-	146	bacteria>proteobacteria>alphaproteobacteria	Sphingobium	MULTISPECIES: host attachment protein [Sphingobium].
WP_021224923.1	-	146	bacteria>proteobacteria>alphaproteobacteria	Sphingobium lactosutens	host attachment protein [Sphingobium lactosutens].
WP_021317719.1	-	146	bacteria>proteobacteria>alphaproteobacteria	Sphingobium ummariense	host attachment protein [Sphingobium ummariense].
ESY70566.1	X742_04350	146	bacteria>proteobacteria>alphaproteobacteria	Mesorhizobium sp. LNHC232B00	Host attachment protein [Mesorhizobium sp. LNHC232B00].
WP_024021514.1	-	146	bacteria>proteobacteria>alphaproteobacteria	Sphingobium sp. C100	host attachment protein [Sphingobium sp. C100].
WP_025041107.1	-	146	bacteria>proteobacteria>betaproteobacteria	Nitrosospira briensis	hypothetical protein [Nitrosospira briensis].
WP_025292032.1	-	146	bacteria>proteobacteria>alphaproteobacteria	Sphingomonas sanxanigenens	host attachment protein [Sphingomonas sanxanigenens].
WP_025548877.1	-	146	bacteria>proteobacteria>alphaproteobacteria	Sphingomonadaceae	MULTISPECIES: host attachment protein [Sphingomonadaceae].
WP_026094873.1	-	146	bacteria>proteobacteria>alphaproteobacteria	Sphingomonas-like bacterium B12	host attachment protein [Sphingomonas-like bacterium B12].
WP_026109258.1	-	146	bacteria>proteobacteria>alphaproteobacteria	Sphingobium yanoikuyae	host attachment protein [Sphingobium yanoikuyae].
WP_027165389.1	-	146	bacteria>proteobacteria>alphaproteobacteria	Mesorhizobium sp. WSM3224	host attachment protein [Mesorhizobium sp. WSM3224].
WP_027854557.1	-	146	bacteria>proteobacteria>gammaproteobacteria	Marinobacterium litorale	hypothetical protein [Marinobacterium litorale].
WP_028465770.1	-	146	bacteria>proteobacteria>alphaproteobacteria	Nisaea denitrificans	hypothetical protein [Nisaea denitrificans].
WP_028572604.1	-	146	bacteria>proteobacteria>deltaproteobacteria	Desulfonatronum lacustre	hypothetical protein [Desulfonatronum lacustre].
WP_028971457.1	-	146	bacteria>proteobacteria>alphaproteobacteria	Sphingopyxis baekryungensis	host attachment protein [Sphingopyxis baekryungensis].
WP_029374944.1	-	146	bacteria>proteobacteria>alphaproteobacteria	Rickettsia honei	hypothetical protein [Rickettsia honei].
WP_029547763.1	-	146	bacteria>proteobacteria>alphaproteobacteria	Sphingobium yanoikuyae	host attachment protein [Sphingobium yanoikuyae].
WP_031396300.1	-	146	bacteria>proteobacteria>alphaproteobacteria	Sphingomonas sp. STIS6.2	host attachment protein [Sphingomonas sp. STIS6.2].
WP_031441820.1	-	146	bacteria>proteobacteria>alphaproteobacteria	Sphingomonas	MULTISPECIES: host attachment protein [Sphingomonas].
WP_032073404.1	-	146	bacteria>proteobacteria>alphaproteobacteria	Rickettsia aeschlimannii	hypothetical protein [Rickettsia aeschlimannii].
WP_032138801.1	-	146	bacteria>proteobacteria>alphaproteobacteria	Rickettsia tamurae	hypothetical protein [Rickettsia tamurae].
WP_035244003.1	-	146	bacteria>proteobacteria>deltaproteobacteria	Desulfonatronovibrio hydrogenovorans	hypothetical protein [Desulfonatronovibrio hydrogenovorans].
WP_035915586.1	-	146	bacteria>proteobacteria>gammaproteobacteria	Legionella lansingensis	hypothetical protein [Legionella lansingensis].
WP_037463403.1	-	146	bacteria>proteobacteria>alphaproteobacteria	Sphingobium herbicidovorans	host attachment protein [Sphingobium herbicidovorans].
WP_037479872.1	-	146	bacteria>proteobacteria>alphaproteobacteria	Sphingobium sp. ba1	host attachment protein [Sphingobium sp. ba1].
WP_037511351.1	-	146	bacteria>proteobacteria>alphaproteobacteria	Sphingobium yanoikuyae	host attachment protein [Sphingobium yanoikuyae].
WP_037522800.1	-	146	bacteria>proteobacteria>alphaproteobacteria	Sphingomonas wittichii	host attachment protein [Sphingomonas wittichii].
WP_040255803.1	-	146	bacteria>proteobacteria>alphaproteobacteria	Rickettsia hoogstraalii	hypothetical protein [Rickettsia hoogstraalii].
WP_040973903.1	-	146	bacteria>proteobacteria>alphaproteobacteria	Mesorhizobium sp. ORS3324	host attachment protein [Mesorhizobium sp. ORS3324].
WP_040990337.1	-	146	bacteria>proteobacteria>alphaproteobacteria	Mesorhizobium plurifarium	host attachment protein [Mesorhizobium plurifarium].
WP_040997525.1	-	146	bacteria>proteobacteria>alphaproteobacteria	Mesorhizobium sp. SOD10	host attachment protein [Mesorhizobium sp. SOD10].
WP_041004942.1	-	146	bacteria>proteobacteria>alphaproteobacteria	Mesorhizobium plurifarium	host attachment protein [Mesorhizobium plurifarium].
WP_041007841.1	-	146	bacteria>proteobacteria>alphaproteobacteria	Mesorhizobium plurifarium	host attachment protein [Mesorhizobium plurifarium].
WP_041079575.1	-	146	bacteria>proteobacteria>alphaproteobacteria	Rickettsia asembonensis	hypothetical protein [Rickettsia asembonensis].
WP_041512726.1	-	146	bacteria>proteobacteria>betaproteobacteria	Nitrosospira sp. NpAV	hypothetical protein [Nitrosospira sp. NpAV].
WP_042104531.1	-	146	bacteria>chlamydiae	Parachlamydiaceae bacterium HS-T3	hypothetical protein [Parachlamydiaceae bacterium HS-T3].
WP_042278894.1	-	146	bacteria>chlamydiae	Candidatus Protochlamydia sp. R18	hypothetical protein [Candidatus Protochlamydia sp. R18].
WP_042641015.1	-	146	bacteria>proteobacteria>alphaproteobacteria	Mesorhizobium sp. ORS3359	host attachment protein [Mesorhizobium sp. ORS3359].
WP_043151165.1	-	146	bacteria>proteobacteria>alphaproteobacteria	Sphingobium sp. Ant17	host attachment protein [Sphingobium sp. Ant17].
WP_044450959.1	-	146	bacteria>cyanobacteria	Mastigocladus laminosus	host attachment protein [Mastigocladus laminosus].
WP_044559002.1	-	146	bacteria>proteobacteria>alphaproteobacteria	Azospirillum sp. B4	hypothetical protein [Azospirillum sp. B4].
WP_045214724.1	-	146	bacteria>proteobacteria>deltaproteobacteria	Desulfonatronovibrio magnus	hypothetical protein [Desulfonatronovibrio magnus].
WP_045218963.1	-	146	bacteria>proteobacteria>deltaproteobacteria	Desulfonatronum thioautotrophicum	hypothetical protein [Desulfonatronum thioautotrophicum].
WP_045800554.1	-	146	bacteria>proteobacteria>alphaproteobacteria	Candidatus Rickettsia amblyommii	hypothetical protein [Candidatus Rickettsia amblyommii].
WP_046711014.1	-	146	bacteria>proteobacteria>deltaproteobacteria	Myxococcus fulvus	hypothetical protein [Myxococcus fulvus].
WP_046764522.1	-	146	bacteria>proteobacteria>alphaproteobacteria	Sphingobium chungbukense	host attachment protein [Sphingobium chungbukense].
KMS57669.1	V473_05500	146	bacteria>proteobacteria>alphaproteobacteria	Sphingobium czechense LL01	Host attachment protein [Sphingobium czechense LL01].
WP_048939100.1	-	146	bacteria>proteobacteria>alphaproteobacteria	Sphingobium yanoikuyae	host attachment protein [Sphingobium yanoikuyae].
WP_050415789.1	-	146	bacteria>proteobacteria>betaproteobacteria	Azoarcus sp. CIB	hypothetical protein [Azoarcus sp. CIB].
WP_051935141.1	-	146	bacteria>proteobacteria>alphaproteobacteria	Sphingomonas wittichii	hypothetical protein [Sphingomonas wittichii].
WP_052640638.1	-	146	bacteria>proteobacteria>alphaproteobacteria	Rhizobium sp. NT-26	hypothetical protein [Rhizobium sp. NT-26].
WP_053351146.1	-	146	bacteria>proteobacteria>alphaproteobacteria	Candidatus Rickettsia amblyommii	hypothetical protein [Candidatus Rickettsia amblyommii].
WP_053421843.1	-	146	bacteria>proteobacteria>betaproteobacteria	Azoarcus sp. PA01	hypothetical protein [Azoarcus sp. PA01].
CDX63090.1	MPL1032_80123	146	bacteria>proteobacteria>alphaproteobacteria	Mesorhizobium plurifarium	Host attachment protein [Mesorhizobium plurifarium].
WP_055778507.1	-	146	bacteria>proteobacteria>alphaproteobacteria	Sphingomonas sp. Leaf357	host attachment protein [Sphingomonas sp. Leaf357].
WP_055877407.1	-	146	bacteria>proteobacteria>alphaproteobacteria	Sphingomonas sp. Leaf34	host attachment protein [Sphingomonas sp. Leaf34].
WP_055931174.1	-	146	bacteria>proteobacteria>alphaproteobacteria	Sphingomonas sp. Leaf17	host attachment protein [Sphingomonas sp. Leaf17].
WP_055983562.1	-	146	bacteria>proteobacteria>alphaproteobacteria	Sphingomonas sp. Leaf412	host attachment protein [Sphingomonas sp. Leaf412].
WP_056015489.1	-	146	bacteria>proteobacteria>alphaproteobacteria	Sphingomonas sp. Leaf20	host attachment protein [Sphingomonas sp. Leaf20].
WP_056048704.1	-	146	bacteria>proteobacteria>alphaproteobacteria	Sphingomonas sp. Leaf198	host attachment protein [Sphingomonas sp. Leaf198].
WP_056058501.1	-	146	bacteria>proteobacteria>alphaproteobacteria	Sphingomonas sp. Leaf230	host attachment protein [Sphingomonas sp. Leaf230].
WP_056366381.1	-	146	bacteria>proteobacteria>alphaproteobacteria	Sphingomonas	MULTISPECIES: host attachment protein [Sphingomonas].
WP_056378147.1	-	146	bacteria>proteobacteria>alphaproteobacteria	Sphingomonas sp. Root710	host attachment protein [Sphingomonas sp. Root710].
WP_056410566.1	-	146	bacteria>proteobacteria>alphaproteobacteria	Sphingomonas sp. Leaf28	host attachment protein [Sphingomonas sp. Leaf28].
WP_056479109.1	-	146	bacteria>proteobacteria>alphaproteobacteria	Sphingomonas sp. Leaf38	host attachment protein [Sphingomonas sp. Leaf38].
WP_056482080.1	-	146	bacteria>proteobacteria>alphaproteobacteria	Sphingomonas sp. Leaf208	host attachment protein [Sphingomonas sp. Leaf208].
WP_056528127.1	-	146	bacteria>proteobacteria>alphaproteobacteria	Sphingomonas sp. Leaf339	host attachment protein [Sphingomonas sp. Leaf339].
WP_056678783.1	-	146	bacteria>proteobacteria>alphaproteobacteria	Sphingomonas sp. Leaf30	host attachment protein [Sphingomonas sp. Leaf30].
WP_057298464.1	-	146	bacteria>proteobacteria>betaproteobacteria	Pelomonas sp. Root1217	hypothetical protein [Pelomonas sp. Root1217].
WP_057638731.1	-	146	bacteria>proteobacteria>gammaproteobacteria	Stenotrophomonas ginsengisoli	hypothetical protein [Stenotrophomonas ginsengisoli].
WP_057700019.1	-	146	bacteria>proteobacteria>alphaproteobacteria	Rickettsia rhipicephali	hypothetical protein [Rickettsia rhipicephali].
WP_059423996.1	-	146	bacteria>proteobacteria>betaproteobacteria	Sulfuricella sp. T08	hypothetical protein [Sulfuricella sp. T08].
WP_060525539.1	-	146	bacteria>proteobacteria>alphaproteobacteria	Sphingomonas sp. Leaf242	Host attachment protein [Sphingomonas sp. Leaf242].
AMK22489.1	K426_07710	146	bacteria>proteobacteria>alphaproteobacteria	Sphingobium sp. TKS	hypothetical protein K426_07710 [Sphingobium sp. TKS].
WP_061226338.1	-	146	bacteria>spirochaetes	Leptospira interrogans	host cell attachment protein [Leptospira interrogans].
WP_062068929.1	-	146	bacteria>proteobacteria>alphaproteobacteria	Sphingobium baderi	Host attachment protein [Sphingobium baderi].
WP_062117384.1	-	146	bacteria>proteobacteria>alphaproteobacteria	Sphingobium sp. MI1205	Host attachment protein [Sphingobium sp. MI1205].
WP_062734354.1	-	146	bacteria>proteobacteria>alphaproteobacteria	Sphingobium abikonense	Host attachment protein [Sphingobium abikonense].
OAN53579.1	A7Q26_06100	146	bacteria>proteobacteria>alphaproteobacteria	Sphingobium sp. TCM1	Host attachment protein [Sphingobium sp. TCM1].
WP_064464699.1	-	146	bacteria>proteobacteria>alphaproteobacteria	Rickettsia raoultii	hypothetical protein [Rickettsia raoultii].
ANI78720.1	EP837_02319	146	bacteria>proteobacteria>alphaproteobacteria	Sphingobium sp. EP60837	hypothetical protein EP837_02319 [Sphingobium sp. EP60837].
OBS10415.1	Thpro_020131	146	bacteria>proteobacteria>gammaproteobacteria	Acidihalobacter prosperus	hypothetical protein Thpro_020131 [Acidihalobacter prosperus].
OBV10998.1	I603_1406	146	bacteria>proteobacteria>alphaproteobacteria	Porphyrobacter dokdonensis DSW-74	Host attachment protein [Porphyrobacter dokdonensis DSW-74].
EKD72807.1	ACD_45C00582G0002	145	bacteria	uncultured bacterium	hypothetical protein ACD_45C00582G0002 [uncultured bacterium].
WP_003002446.1	-	145	bacteria>spirochaetes	Leptospira weilii	host cell attachment protein [Leptospira weilii].
WP_003637145.1	-	145	bacteria>proteobacteria>gammaproteobacteria	Legionella longbeachae	hypothetical protein [Legionella longbeachae].
WP_004251484.1	-	145	bacteria>proteobacteria>betaproteobacteria	Thauera	MULTISPECIES: hypothetical protein [Thauera].
WP_005795767.1	-	145	bacteria>proteobacteria>betaproteobacteria	Acidovorax delafieldii	hypothetical protein [Acidovorax delafieldii].
WP_006007435.1	-	145	bacteria>proteobacteria>gammaproteobacteria	Glaciecola punicea	hypothetical protein [Glaciecola punicea].
WP_007714520.1	-	145	bacteria>proteobacteria>alphaproteobacteria	Sphingobium sp. AP49	protein required for attachment to host cell [Sphingobium sp. AP49].
WP_008229827.1	-	145	bacteria>proteobacteria>alphaproteobacteria	Roseobacter sp. GAI101	hypothetical protein [Roseobacter sp. GAI101].
WP_011628426.1	-	145	bacteria>proteobacteria>gammaproteobacteria	Alkalilimnicola ehrlichii	hypothetical protein [Alkalilimnicola ehrlichii].
WP_011765417.1	-	145	bacteria>proteobacteria>betaproteobacteria	Azoarcus	MULTISPECIES: hypothetical protein [Azoarcus].
WP_014393617.1	-	145	bacteria>proteobacteria>deltaproteobacteria	Corallococcus coralloides	hypothetical protein [Corallococcus coralloides].
WP_014949626.1	-	145	bacteria>proteobacteria>gammaproteobacteria	Alteromonas macleodii	hypothetical protein [Alteromonas macleodii].
WP_014976659.1	-	145	bacteria>proteobacteria>gammaproteobacteria	Alteromonas macleodii	hypothetical protein [Alteromonas macleodii].
WP_014979395.1	-	145	bacteria>proteobacteria>gammaproteobacteria	Alteromonas macleodii	hypothetical protein [Alteromonas macleodii].
WP_014998484.1	-	145	bacteria>proteobacteria>gammaproteobacteria	Alteromonas macleodii	hypothetical protein [Alteromonas macleodii].
WP_015067313.1	-	145	bacteria>proteobacteria>gammaproteobacteria	Alteromonas mediterranea	hypothetical protein [Alteromonas mediterranea].
WP_018701043.1	-	145	bacteria>proteobacteria>alphaproteobacteria	Amorphus coralli	hypothetical protein [Amorphus coralli].
WP_020414540.1	-	145	bacteria>proteobacteria>gammaproteobacteria	Microbulbifer variabilis	hypothetical protein [Microbulbifer variabilis].
WP_020743689.1	-	145	bacteria>proteobacteria>gammaproteobacteria	Alteromonas mediterranea	hypothetical protein [Alteromonas mediterranea].
WP_020746042.1	-	145	bacteria>proteobacteria>gammaproteobacteria	Alteromonas mediterranea	hypothetical protein [Alteromonas mediterranea].
WP_021238216.1	-	145	bacteria>proteobacteria>alphaproteobacteria	Sphingomonadaceae	MULTISPECIES: hypothetical protein [Sphingomonadaceae].
WP_021247830.1	-	145	bacteria>proteobacteria>betaproteobacteria	Thauera terpenica	hypothetical protein [Thauera terpenica].
WP_022681067.1	-	145	bacteria>proteobacteria>alphaproteobacteria	Sandarakinorhabdus limnophila	host attachment protein [Sandarakinorhabdus limnophila].
WP_022947570.1	-	145	bacteria>proteobacteria>gammaproteobacteria	Methylohalobius crimeensis	hypothetical protein [Methylohalobius crimeensis].
AHD03700.1	METH_22990	145	bacteria>proteobacteria>alphaproteobacteria	Leisingera methylohalidivorans DSM 14336	hypothetical protein METH_22990 (plasmid) [Leisingera methylohalidivorans DSM 14336].
WP_024300006.1	-	145	bacteria>proteobacteria>gammaproteobacteria	Methylosarcina lacus	host attachment protein [Methylosarcina lacus].

WP_025255357.1	-	145	bacteria>proteobacteria>gammaproteobacteria	Alteromonas sp. ALT199	hypothetical protein [Alteromonas sp. ALT199].
WP_027270040.1	-	145	bacteria>proteobacteria>gammaproteobacteria	Legionella sainthelensi	hypothetical protein [Legionella sainthelensi].
WP_028794070.1	-	145	bacteria>proteobacteria>alphaproteobacteria	Thalassobaculum salexigens	host attachment protein [Thalassobaculum salexigens].
WP_032125570.1	-	145	bacteria>chlamydiae	Chlamydiales	MULTISPECIES: hypothetical protein [Chlamydiales].
WP_034812718.1	-	145	bacteria>proteobacteria>alphaproteobacteria	Hyphomonas sp. L-53-1-40	hypothetical protein [Hyphomonas sp. L-53-1-40].
WP_034903565.1	-	145	bacteria>proteobacteria>alphaproteobacteria	Erythrobacter litoralis	hypothetical protein [Erythrobacter litoralis].
WP_037279509.1	-	145	bacteria>proteobacteria>alphaproteobacteria	Rubellimicrobium mesophilum	host attachment protein [Rubellimicrobium mesophilum].
WP_039001356.1	-	145	bacteria>proteobacteria>alphaproteobacteria	Halocynthiibacter arcticus	hypothetical protein [Halocynthiibacter arcticus].
KIE05368.1	NF27_DT01420	145	bacteria>proteobacteria>alphaproteobacteria	Candidatus Jidaibacter acanthamoeba	hypothetical protein NF27_DT01420 [Candidatus Jidaibacter acanthamoeba].
WP_039220875.1	-	145	bacteria>proteobacteria>gammaproteobacteria	Alteromonas marina	hypothetical protein [Alteromonas marina].
WP_039234668.1	-	145	bacteria>proteobacteria>gammaproteobacteria	Alteromonas macleodii	hypothetical protein [Alteromonas macleodii].
WP_041018578.1	-	145	bacteria>chlamydiae	Criblamydia sequanensis	hypothetical protein [Criblamydia sequanensis].
WP_041065494.1	-	145	bacteria>proteobacteria>gammaproteobacteria	Thiolapillus brandeum	host attachment protein [Thiolapillus brandeum].
WP_043319204.1	-	145	bacteria>proteobacteria>gammaproteobacteria	Microbulbifer sp. HZ11	host cell attachment-required protein [Microbulbifer sp. HZ11].
KJS24554.1	VR75_14705	145	bacteria>proteobacteria>alphaproteobacteria	Hyphomonadaceae bacterium BRH_c29	hypothetical protein VR75_14705 [Hyphomonadaceae bacterium BRH_c29].
KJS38220.1	VR74_06290	145	bacteria>proteobacteria>alphaproteobacteria	Hyphomonas sp. BRH_c22	hypothetical protein VR74_06290 [Hyphomonas sp. BRH_c22].
WP_050532743.1	-	145	bacteria>proteobacteria>alphaproteobacteria	Aestuariivita atlantica	hypothetical protein [Aestuariivita atlantica].
WP_050929750.1	-	145	bacteria>proteobacteria>alphaproteobacteria	Aestuariivita boseongensis	hypothetical protein [Aestuariivita boseongensis].
WP_051371736.1	-	145	bacteria>proteobacteria>alphaproteobacteria	Leisingera aquimarina	hypothetical protein [Leisingera aquimarina].
WP_051596426.1	-	145	bacteria>proteobacteria>alphaproteobacteria	Hyphomonas adhaerens	hypothetical protein [Hyphomonas adhaerens].
WP_051597799.1	-	145	bacteria>proteobacteria>alphaproteobacteria	Hyphomonas jannaschiana	hypothetical protein [Hyphomonas jannaschiana].
WP_051600202.1	-	145	bacteria>proteobacteria>alphaproteobacteria	Hyphomonas	MULTISPECIES: hypothetical protein [Hyphomonas].
WP_051882885.1	-	145	bacteria>proteobacteria>gammaproteobacteria	Salinisphaera hydrothermalis	hypothetical protein [Salinisphaera hydrothermalis].
KPK58006.1	AMJ59_17025	145	bacteria>proteobacteria>gammaproteobacteria	Gammaproteobacteria bacterium SG8_31	hypothetical protein AMJ59_17025 [Gammaproteobacteria bacterium SG8_31].
KPK61594.1	AMJ59_00845	145	bacteria>proteobacteria>gammaproteobacteria	Gammaproteobacteria bacterium SG8_31	hypothetical protein AMJ59_00845 [Gammaproteobacteria bacterium SG8_31].
WP_055295107.1	-	145	bacteria>proteobacteria>alphaproteobacteria	Loktanella sp. 1ANDIMAR09	hypothetical protein [Loktanella sp. 1ANDIMAR09].
WP_055684882.1	-	145	bacteria>proteobacteria>alphaproteobacteria	Loktanella sp. 5RATIMAR09	hypothetical protein [Loktanella sp. 5RATIMAR09].
WP_056689937.1	-	145	bacteria>proteobacteria>alphaproteobacteria	Sphingobium sp. Leaf26	host attachment protein [Sphingobium sp. Leaf26].
WP_058462758.1	-	145	bacteria>proteobacteria>gammaproteobacteria	Legionella adelaidensis	hypothetical protein [Legionella adelaidensis].
WP_058514418.1	-	145	bacteria>proteobacteria>gammaproteobacteria	Legionella santicrucis	hypothetical protein [Legionella santicrucis].
KUJ83499.1	AVO43_06455	145	bacteria>proteobacteria>gammaproteobacteria	Microbulbifer sp. ZGT114	host cell attachment-required protein [Microbulbifer sp. ZGT114].
WP_059186520.1	-	145	bacteria>proteobacteria>alphaproteobacteria	Mesorhizobium loti	Host attachment protein [Mesorhizobium loti].
WP_061095154.1	-	145	bacteria>proteobacteria>gammaproteobacteria	Alteromonas macleodii	hypothetical protein [Alteromonas macleodii].
KXJ39607.1	AXA67_14185	145	bacteria>proteobacteria>gammaproteobacteria	Methylothermaceae bacteria B42	hypothetical protein AXA67_14185 [Methylothermaceae bacteria B42].
AMX04227.1	A3224_12405	145	bacteria>proteobacteria>gammaproteobacteria	Microbulbifer thermotolerans	host cell attachment-required protein [Microbulbifer thermotolerans].
OBQ74142.1	A9K71_13645	145	bacteria>proteobacteria>alphaproteobacteria	Mesorhizobium sp. WSM3873	Host attachment protein [Mesorhizobium sp. WSM3873].
OBQ97059.1	A9K66_00955	145	bacteria>proteobacteria>alphaproteobacteria	Mesorhizobium sp. AA23	Host attachment protein [Mesorhizobium sp. AA23].
WP_065089239.1	-	145	bacteria>proteobacteria>gammaproteobacteria	Acidihalobacter prosperus	hypothetical protein [Acidihalobacter prosperus].
WP_002611509.1	-	144	bacteria>proteobacteria>deltaproteobacteria	Stigmatella aurantiaca	hypothetical protein [Stigmatella aurantiaca].
WP_002628943.1	-	144	bacteria>proteobacteria>deltaproteobacteria	Cystobacter fuscus	hypothetical protein [Cystobacter fuscus].
WP_004467392.1	-	144	bacteria>spirochaetes	Leptospira santarosai	host cell attachment protein [Leptospira santarosai].
WP_004469326.1	-	144	bacteria>spirochaetes	Leptospira santarosai	host cell attachment protein [Leptospira santarosai].
WP_007144473.1	-	144	bacteria>proteobacteria>gammaproteobacteria	Methylophaga aminisulfidivorans	hypothetical protein [Methylophaga aminisulfidivorans].
WP_007226525.1	-	144	bacteria>proteobacteria>gammaproteobacteria	marine gamma proteobacterium HTCC2143	hypothetical protein [marine gamma proteobacterium HTCC2143].
WP_008933036.1	-	144	bacteria>proteobacteria>gammaproteobacteria	Ectothiorhodospira sp. PHS-1	host cell attachment-required protein [Ectothiorhodospira sp. PHS-1].
WP_010133576.1	-	144	bacteria>proteobacteria>gammaproteobacteria	Microbulbifer agarilyticus	host cell attachment-required protein [Microbulbifer agarilyticus].
WP_010322562.1	-	144	bacteria>proteobacteria>gammaproteobacteria	Marinobacterium stanieri	host attachment protein [Marinobacterium stanieri].
WP_011505435.1	-	144	bacteria>proteobacteria>gammaproteobacteria	Chromohalobacter	MULTISPECIES: hypothetical protein [Chromohalobacter].
WP_012637852.1	-	144	bacteria>proteobacteria>gammaproteobacteria	Thioalkalivibrio sulfidiphilus	host cell attachment-required protein [Thioalkalivibrio sulfidiphilus].
WP_014777374.1	-	144	bacteria>proteobacteria>gammaproteobacteria	Thiocystis violascens	attachment to host cells protein [Thiocystis violascens].
WP_015280718.1	-	144	bacteria>proteobacteria>gammaproteobacteria	Thioflavicoccus mobilis	attachment to host cells protein [Thioflavicoccus mobilis].
WP_018231026.1	-	144	bacteria>proteobacteria>gammaproteobacteria	Thioalkalivibrio thiocyanodenitrificans	host cell attachment-required protein [Thioalkalivibrio thiocyanodenitrificans].
WP_018293874.1	-	144	bacteria>proteobacteria	Mariprofundus ferrooxydans	host cell attachment-required protein [Mariprofundus ferrooxydans].
WP_018954464.1	-	144	bacteria>proteobacteria>gammaproteobacteria	Thioalkalivibrio sulfidiphilus	host cell attachment-required protein [Thioalkalivibrio sulfidiphilus].
WP_020582530.1	-	144	bacteria>proteobacteria>gammaproteobacteria	Endozoicomonas elysicola	hypothetical protein [Endozoicomonas elysicola].
CDQ34255.1	BN993_03709	144	bacteria>firmicutes	Virgibacillus halodenitrificans	Protein required for attachment to host cells [Virgibacillus halodenitrificans].
WP_025281126.1	-	144	bacteria>proteobacteria>gammaproteobacteria	Ectothiorhodospira haloalkaliphila	host attachment protein [Ectothiorhodospira haloalkaliphila].
WP_029132124.1	-	144	bacteria>proteobacteria>gammaproteobacteria	Sedimenticola selenatireducens	hypothetical protein [Sedimenticola selenatireducens].
WP_034959117.1	-	144	bacteria>proteobacteria>alphaproteobacteria	Komagataeibacter kakiaceti	hypothetical protein [Komagataeibacter kakiaceti].
WP_037207291.1	-	144	bacteria>proteobacteria>alphaproteobacteria	Rhodovulum sp. NI22	hypothetical protein [Rhodovulum sp. NI22].
WP_040240594.1	-	144	bacteria>proteobacteria>gammaproteobacteria	Chromohalobacter japonicus	hypothetical protein [Chromohalobacter japonicus].
WP_042459338.1	-	144	bacteria>proteobacteria>alphaproteobacteria	Rhodovulum sulfidophilum	hypothetical protein [Rhodovulum sulfidophilum].
WP_043403981.1	-	144	bacteria>proteobacteria>deltaproteobacteria	Cystobacter violaceus	hypothetical protein [Cystobacter violaceus].
WP_044182982.1	-	144	bacteria>proteobacteria>deltaproteobacteria	Hyalangium minutum	hypothetical protein [Hyalangium minutum].
BAR55369.1	NK6_2188	144	bacteria>proteobacteria>alphaproteobacteria	Bradyrhizobium diazoefficiens	hypothetical protein NK6_2188 [Bradyrhizobium diazoefficiens].
WP_046860547.1	-	144	bacteria>proteobacteria>gammaproteobacteria	Sedimenticola thiotaurini	hypothetical protein [Sedimenticola thiotaurini].
WP_047855102.1	-	144	bacteria>proteobacteria>deltaproteobacteria	Archangium gephyra	hypothetical protein [Archangium gephyra].
CRX39424.1	ELAC_2103	144	bacteria>chlamydiae	Estrella lausannensis	Conserved hypothetical protein [Estrella lausannensis].
WP_053175108.1	-	144	bacteria>proteobacteria>betaproteobacteria	Limnohabitans planktonicus	hypothetical protein [Limnohabitans planktonicus].
KPK55490.1	AMS22_04110	144	bacteria>proteobacteria>gammaproteobacteria	Thiotrichales bacterium SG8_50	hypothetical protein AMS22_04110 [Thiotrichales bacterium SG8_50].
WP_054539556.1	-	144	bacteria>proteobacteria>alphaproteobacteria	Confluentimicrobium sp. EMB200-NS6	hypothetical protein [Confluentimicrobium sp. EMB200-NS6].
WP_054774059.1	-	144	bacteria>proteobacteria>gammaproteobacteria	Methylogaea oryzae	hypothetical protein [Methylogaea oryzae].
WP_056711376.1	-	144	bacteria>proteobacteria>alphaproteobacteria	Bosea sp. Leaf344	hypothetical protein [Bosea sp. Leaf344].
WP_058554776.1	-	144	bacteria>proteobacteria>gammaproteobacteria	Thiohalocapsa sp. ML1	hypothetical protein [Thiohalocapsa sp. ML1].
WP_063465322.1	-	144	bacteria>proteobacteria>gammaproteobacteria	Ectothiorhodospira sp. BSL-9	Host attachment protein [Ectothiorhodospira sp. BSL-9].
AAD44005.1	atsE	143	bacteria>proteobacteria>alphaproteobacteria	Agrobacterium fabrum str. C58	AtsE, partial [Agrobacterium fabrum str. C58].
EER21651.1	REIS_0814	143	bacteria>proteobacteria>alphaproteobacteria	Rickettsia endosymbiont of Ixodes scapularis	conserved hypothetical protein [Rickettsia endosymbiont of Ixodes scapularis].
EGE48736.1	APO_0326	143	bacteria>proteobacteria>alphaproteobacteria	Acetobacter pomorum DM001	AtsE Protein [Acetobacter pomorum DM001].
EKD61489.1	ACD_54C00215G0006	143	bacteria	uncultured bacterium	hypothetical protein ACD_54C00215G0006 [uncultured bacterium].
EKE01703.1	ACD_21C00072G0026	143	bacteria	uncultured bacterium	hypothetical protein ACD_21C00072G0026 [uncultured bacterium].
WP_007191648.1	-	143	bacteria>proteobacteria>gammaproteobacteria	Thiocapsa marina	host attachment protein [Thiocapsa marina].
WP_008956565.1	-	143	bacteria>proteobacteria>gammaproteobacteria	Halomonas sp. HAL1	hypothetical protein [Halomonas sp. HAL1].
WP_009098345.1	-	143	bacteria>proteobacteria>gammaproteobacteria	Halomonas sp. GFAJ-1	hypothetical protein [Halomonas sp. GFAJ-1].
WP_009566631.1	-	143	bacteria>proteobacteria>alphaproteobacteria	Rhodobacter sp. AKP1	hypothetical protein [Rhodobacter sp. AKP1].
WP_009723190.1	-	143	bacteria>proteobacteria>gammaproteobacteria	Halomonas sp. TD01	hypothetical protein [Halomonas sp. TD01].
WP_010959910.1	-	143	bacteria>proteobacteria>gammaproteobacteria	Methylococcus capsulatus	hypothetical protein [Methylococcus capsulatus].
WP_012971689.1	-	143	bacteria>proteobacteria>gammaproteobacteria	Allochromatium vinosum	host attachment protein [Allochromatium vinosum].
WP_017228987.1	-	143	bacteria>proteobacteria>betaproteobacteria	Cupriavidus basilensis	hypothetical protein [Cupriavidus basilensis].
WP_017978699.1	-	143	bacteria>proteobacteria>alphaproteobacteria	Sphingomonas	MULTISPECIES: hypothetical protein [Sphingomonas].
WP_025275270.1	-	143	bacteria>proteobacteria>gammaproteobacteria	Marichromatium	MULTISPECIES: host attachment protein [Marichromatium].
WP_026001891.1	-	143	bacteria>proteobacteria>gammaproteobacteria	Halomonas stevensii	hypothetical protein [Halomonas stevensii].
WP_026285651.1	-	143	bacteria>proteobacteria>gammaproteobacteria	Halomonas zhanjiangensis	hypothetical protein [Halomonas zhanjiangensis].
WP_026634141.1	-	143	bacteria>proteobacteria>gammaproteobacteria	Dyella japonica	hypothetical protein [Dyella japonica].
WP_027337174.1	-	143	bacteria>proteobacteria>gammaproteobacteria	Halomonas sp. HL-48	hypothetical protein [Halomonas sp. HL-48].
WP_030071122.1	-	143	bacteria>proteobacteria>gammaproteobacteria	Halomonas alkaliantarctica	hypothetical protein [Halomonas alkaliantarctica].
KFC50817.1	DK37_06680	143	bacteria>proteobacteria>gammaproteobacteria	Halomonas sp. SUBG004	hypothetical protein DK37_06680 [Halomonas sp. SUBG004].
WP_031432754.1	-	143	bacteria>proteobacteria>gammaproteobacteria	Methylomarinum vadi	hypothetical protein [Methylomarinum vadi].
WP_035863444.1	-	143	bacteria>proteobacteria>betaproteobacteria	Cupriavidus sp. SK-3	hypothetical protein [Cupriavidus sp. SK-3].
WP_036134559.1	-	143	bacteria>proteobacteria>gammaproteobacteria	Lysobacter daejeonensis	hypothetical protein [Lysobacter daejeonensis].
WP_037536214.1	-	143	bacteria>proteobacteria>alphaproteobacteria	Sphingomonas sp. RIT328	host attachment protein [Sphingomonas sp. RIT328].
WP_038479465.1	-	143	bacteria>proteobacteria>gammaproteobacteria	Halomonas campaniensis	hypothetical protein [Halomonas campaniensis].
WP_038658288.1	-	143	bacteria>proteobacteria>alphaproteobacteria	Sphingomonas taxi	host attachment protein [Sphingomonas taxi].
WP_039179255.1	-	143	bacteria>proteobacteria>gammaproteobacteria	Halomonas hydrothermalis	hypothetical protein [Halomonas hydrothermalis].
WP_042482890.1	-	143	bacteria>proteobacteria>alphaproteobacteria	Sphingomonas parapaucimobilis	host attachment protein [Sphingomonas parapaucimobilis].
WP_044629926.1	-	143	bacteria>proteobacteria	Gammaproteobacteria	MULTISPECIES: hypothetical protein [Gammaproteobacteria].
WP_045242718.1	-	143	bacteria>proteobacteria>betaproteobacteria	Burkholderiaceae bacterium 16	hypothetical protein [Burkholderiaceae bacterium 16].
WP_045993902.1	-	143	bacteria>proteobacteria>gammaproteobacteria	Halomonas sp. S2151	hypothetical protein [Halomonas sp. S2151].
WP_046477992.1	-	143	bacteria>proteobacteria>alphaproteobacteria	Candidatus Filomicrobium marinum	hypothetical protein [Candidatus Filomicrobium marinum].
WP_048308309.1	-	143	bacteria>proteobacteria>gammaproteobacteria	Halomonas sp. PR-M31	hypothetical protein [Halomonas sp. PR-M31].
WP_051682333.1	-	143	bacteria>proteobacteria>alphaproteobacteria	Thalassospira permensis	hypothetical protein [Thalassospira permensis].
KPQ23271.1	HLUCCO06_16215	143	bacteria>proteobacteria>gammaproteobacteria	Halomonas sp. HL-93	hypothetical protein HLUCCO06_16215 [Halomonas sp. HL-93].
WP_055756267.1	-	143	bacteria>proteobacteria>alphaproteobacteria	Sphingomonas	MULTISPECIES: host attachment protein [Sphingomonas].
WP_055784201.1	-	143	bacteria>proteobacteria>alphaproteobacteria	Sphingomonas	MULTISPECIES: host attachment protein [Sphingomonas].
WP_058732122.1	-	143	bacteria>proteobacteria>alphaproteobacteria	Sphingomonas sanguinis	Host attachment protein [Sphingomonas sanguinis].
WP_058744011.1	-	143	bacteria>proteobacteria>alphaproteobacteria	Sphingomonas yabuuchiae	Host attachment protein [Sphingomonas yabuuchiae].
WP_058752841.1	-	143	bacteria>proteobacteria>alphaproteobacteria	Sphingomonas sanguinis	Host attachment protein [Sphingomonas sanguinis].
CRI64125.1	THIOKS11730034	143	bacteria>proteobacteria>gammaproteobacteria	Thiocapsa sp. KS1	Host attachment protein [Thiocapsa sp. KS1].
WP_061779063.1	-	143	bacteria>proteobacteria>alphaproteobacteria	Sphingomonas sanguinis	Host attachment protein [Sphingomonas sanguinis].
KZE18854.1	AVT10_02135	143	bacteria>proteobacteria>alphaproteobacteria	Sphingomonas hankookensis	Host attachment protein [Sphingomonas hankookensis].
OAZ93420.1	ADS46_04175	143	bacteria>proteobacteria>gammaproteobacteria	Halomonas sp. G11	hypothetical protein ADS46_04175 [Halomonas sp. G11].
WP_004581195.1	-	142	bacteria>proteobacteria>gammaproteobacteria	Marinobacter nanhaiticus	hypothetical protein [Marinobacter nanhaiticus].
WP_007039757.1	-	142	bacteria>proteobacteria>gammaproteobacteria	Thiorhodococcus drewsii	host attachment protein [Thiorhodococcus drewsii].
WP_007112370.1	-	142	bacteria>proteobacteria>gammaproteobacteria	Halomonas boliviensis	hypothetical protein [Halomonas boliviensis].
WP_008594114.1	-	142	bacteria>spirochaetes	Leptospira licerasiae	host cell attachment protein [Leptospira licerasiae].
WP_008739463.1	-	142	bacteria>proteobacteria>gammaproteobacteria	Alcanivorax pacificus	hypothetical protein [Alcanivorax pacificus].
WP_010625811.1	-	142	bacteria>proteobacteria>gammaproteobacteria	Halomonas sp. KM-1	hypothetical protein [Halomonas sp. KM-1].
WP_011397756.1	-	142	bacteria>proteobacteria>gammaproteobacteria	Hahella chejuensis	attachment to host cells protein [Hahella chejuensis].
WP_013182330.1	-	142	bacteria>chlamydiae	Waddlia chondrophila	hypothetical protein [Waddlia chondrophila].
WP_016418188.1	-	142	bacteria>proteobacteria>gammaproteobacteria	Halomonas anticariensis	hypothetical protein [Halomonas anticariensis].
WP_016854108.1	-	142	bacteria>proteobacteria>gammaproteobacteria	Halomonas smyrnensis	hypothetical protein [Halomonas smyrnensis].
WP_019019176.1	-	142	bacteria>proteobacteria>gammaproteobacteria	Halomonas lutea	hypothetical protein [Halomonas lutea].
WP_020407233.1	-	142	bacteria>proteobacteria>gammaproteobacteria	Hahella ganghwensis	hypothetical protein [Hahella ganghwensis].
WP_020768770.1	-	142	bacteria>spirochaetes	Leptospira sp. B5-022	host cell attachment protein [Leptospira sp. B5-022].
WP_021268242.1	-	142	bacteria>proteobacteria>deltaproteobacteria	Bacteriovorax sp. BAL6_X	host cell attachment protein [Bacteriovorax sp. BAL6_X].
WP_022521305.1	-	142	bacteria>proteobacteria>gammaproteobacteria	Halomonas	MULTISPECIES: hypothetical protein [Halomonas].
WP_023004274.1	-	142	bacteria>proteobacteria>gammaproteobacteria	Halomonas sp. PBN3	hypothetical protein [Halomonas sp. PBN3].
WP_024951510.1	-	142	bacteria>proteobacteria>gammaproteobacteria	Cobetia crustatorum	hypothetical protein [Cobetia crustatorum].
WP_025869822.1	-	142	bacteria>proteobacteria>betaproteobacteria	Methylobacillus glycogenes	hypothetical protein [Methylobacillus glycogenes].
WP_027960567.1	-	142	bacteria>proteobacteria>gammaproteobacteria	Halomonas halodenitrificans	hypothetical protein [Halomonas halodenitrificans].
AIL13155.1	IM40_05980	142	bacteria>proteobacteria>alphaproteobacteria	Candidatus Caedibacter acanthamoebae	hypothetical protein IM40_05980 [Candidatus Caedibacter acanthamoebae].
WP_035555087.1	-	142	bacteria>proteobacteria>gammaproteobacteria	Halomonas sp. KO116	hypothetical protein [Halomonas sp. KO116].
WP_035581907.1	-	142	bacteria>proteobacteria>gammaproteobacteria	Halomonas sp. TG39a	hypothetical protein [Halomonas sp. TG39a].
WP_035599752.1	-	142	bacteria>proteobacteria>gammaproteobacteria	Halomonas salina	hypothetical protein [Halomonas salina].
WP_036273574.1	-	142	bacteria>proteobacteria>gammaproteobacteria	Methylomonas	MULTISPECIES: hypothetical protein [Methylomonas].
WP_040183759.1	-	142	bacteria>proteobacteria>gammaproteobacteria	Halomonas salina	hypothetical protein [Halomonas salina].
WP_043107585.1	-	142	bacteria>proteobacteria>gammaproteobacteria	endosymbiont of unidentified scaly snail isolate Monju	hypothetical protein [endosymbiont of unidentified scaly snail isolate Monju].
WP_043331587.1	-	142	bacteria>proteobacteria>gammaproteobacteria	Cobetia	MULTISPECIES: hypothetical protein [Cobetia].
WP_043517200.1	-	142	bacteria>proteobacteria>gammaproteobacteria	Halomonas sp. BC04	hypothetical protein [Halomonas sp. BC04].
WP_043518549.1	-	142	bacteria>proteobacteria>gammaproteobacteria	Halomonas sp. BC04	hypothetical protein [Halomonas sp. BC04].
WP_043753094.1	-	142	bacteria>proteobacteria>gammaproteobacteria	Thiorhodococcus sp. AK35	host attachment protein [Thiorhodococcus sp. AK35].
WP_046079037.1	-	142	bacteria>proteobacteria>gammaproteobacteria	Halomonas sp. HG01	hypothetical protein [Halomonas sp. HG01].
WP_052234471.1	-	142	bacteria>proteobacteria>betaproteobacteria	Massilia	MULTISPECIES: hypothetical protein [Massilia].
WP_054762209.1	-	142	bacteria>proteobacteria>gammaproteobacteria	Methylomonas koyamae	hypothetical protein [Methylomonas koyamae].
WP_058524793.1	-	142	bacteria>proteobacteria>gammaproteobacteria	Legionella birminghamensis	hypothetical protein [Legionella birminghamensis].
KUJ86307.1	XD36_3258	142	bacteria>proteobacteria>gammaproteobacteria	Halomonas sp. 54_146	hypothetical protein XD36_3258 [Halomonas sp. 54_146].
AMD02581.1	LOKO_03541	142	bacteria>proteobacteria>gammaproteobacteria	Halomonas chromatireducens	host cell attachment protein [Halomonas chromatireducens].
KXS37515.1	AWU55_2260	142	bacteria>proteobacteria>gammaproteobacteria	Halomonadaceae bacterium T82-2	hypothetical protein AWU55_2260 [Halomonadaceae bacterium T82-2].
WP_064023619.1	-	142	bacteria>proteobacteria>gammaproteobacteria	Methylomonas sp. DH-1	hypothetical protein [Methylomonas sp. DH-1].
WP_064027752.1	-	142	bacteria>proteobacteria>gammaproteobacteria	Methylomonas koyamae	hypothetical protein [Methylomonas koyamae].
WP_064235682.1	-	142	bacteria>proteobacteria>gammaproteobacteria	Halomonas sp. ALS9	hypothetical protein [Halomonas sp. ALS9].
EIT67664.1	WQQ_40990	141	bacteria>proteobacteria>gammaproteobacteria	Hydrocarboniphaga effusa AP103	hypothetical protein WQQ_40990 [Hydrocarboniphaga effusa AP103].
EMN19166.1	LEP1GSC056_2073	141	bacteria>spirochaetes	Leptospira borgpetersenii str. Brem 328	host cell attachment protein [Leptospira borgpetersenii str. Brem 328].
EMO50359.1	LEP1GSC190_2152	141	bacteria>spirochaetes	Leptospira mayottensis 200901116	host cell attachment protein [Leptospira mayottensis 200901116].
WP_010570691.1	-	141	bacteria>spirochaetes	Leptospira	MULTISPECIES: attachment to host cells protein [Leptospira].
WP_021235421.1	-	141	bacteria>proteobacteria>alphaproteobacteria	Novosphingobium lindaniclasticum	attachment protein [Novosphingobium lindaniclasticum].
WP_021817009.1	-	141	bacteria>proteobacteria>gammaproteobacteria	Halomonas huangheensis	hypothetical protein [Halomonas huangheensis].
WP_029604663.1	-	141	bacteria>proteobacteria>alphaproteobacteria	Kozakia baliensis	attachment protein [Kozakia baliensis].
WP_029921120.1	-	141	bacteria>proteobacteria>gammaproteobacteria	Nevskia soli	hypothetical protein [Nevskia soli].
WP_052646257.1	-	141	bacteria>proteobacteria>alphaproteobacteria	endosymbiont of Acanthamoeba sp. UWC8	hypothetical protein [endosymbiont of Acanthamoeba sp. UWC8].
WP_056404340.1	-	141	bacteria>proteobacteria>betaproteobacteria	Massilia sp. Root418	hypothetical protein [Massilia sp. Root418].
KXJ48189.1	AXW13_08290	141	bacteria>proteobacteria>gammaproteobacteria	Alcanivorax sp. Nap_24	hypothetical protein AXW13_08290 [Alcanivorax sp. Nap_24].
WP_003624226.1	-	140	bacteria>proteobacteria>alphaproteobacteria	Acetobacter pasteurianus	peptidase [Acetobacter pasteurianus].
WP_007014911.1	-	140	bacteria>proteobacteria>alphaproteobacteria	Novosphingobium	MULTISPECIES: attachment protein [Novosphingobium].
WP_007281780.1	-	140	bacteria>proteobacteria>alphaproteobacteria	Gluconobacter	MULTISPECIES: AtsE protein [Gluconobacter].
WP_007683563.1	-	140	bacteria>proteobacteria>alphaproteobacteria	Novosphingobium sp. AP12	attachment protein [Novosphingobium sp. AP12].
WP_008832325.1	-	140	bacteria>proteobacteria>alphaproteobacteria	Sphingomonadaceae	MULTISPECIES: attachment protein [Sphingomonadaceae].
WP_008851902.1	-	140	bacteria>proteobacteria>alphaproteobacteria	Gluconobacter morbifer	attachment protein [Gluconobacter morbifer].
WP_010503385.1	-	140	bacteria>proteobacteria>alphaproteobacteria	Gluconobacter	MULTISPECIES: AtsE protein [Gluconobacter].
WP_011252444.1	-	140	bacteria>proteobacteria>alphaproteobacteria	Gluconobacter oxydans	attachment protein [Gluconobacter oxydans].
WP_015073054.1	-	140	bacteria>proteobacteria>alphaproteobacteria	Gluconobacter oxydans	AtsE protein [Gluconobacter oxydans].
WP_019088531.1	-	140	bacteria>proteobacteria>alphaproteobacteria	Acetobacter pasteurianus	peptidase [Acetobacter pasteurianus].
WP_034880738.1	-	140	bacteria>proteobacteria>alphaproteobacteria	Gluconobacter frateurii	attachment protein [Gluconobacter frateurii].
WP_035476355.1	-	140	bacteria>proteobacteria>gammaproteobacteria	Gammaproteobacteria bacterium MFB021	hypothetical protein [Gammaproteobacteria bacterium MFB021].
WP_039283418.1	-	140	bacteria>proteobacteria>alphaproteobacteria	Novosphingobium malaysiense	attachment protein [Novosphingobium malaysiense].
WP_043531014.1	-	140	bacteria>proteobacteria>gammaproteobacteria	Halomonas xinjiangensis	hypothetical protein [Halomonas xinjiangensis].
WP_043975320.1	-	140	bacteria>proteobacteria>alphaproteobacteria	Novosphingobium sp. P6W	attachment protein [Novosphingobium sp. P6W].
WP_046900557.1	-	140	bacteria>proteobacteria>alphaproteobacteria	Gluconobacter	MULTISPECIES: attachment protein [Gluconobacter].
WP_048850420.1	-	140	bacteria>proteobacteria>alphaproteobacteria	Tanticharoenia sakaeratensis	attachment protein [Tanticharoenia sakaeratensis].
BAR98747.1	BV133_1154	140	bacteria>proteobacteria>alphaproteobacteria	Blastochloris viridis	hypothetical protein BV133_1154 [Blastochloris viridis].
WP_050818595.1	-	140	bacteria>proteobacteria>alphaproteobacteria	Acetobacter pasteurianus	peptidase [Acetobacter pasteurianus].
WP_054435844.1	-	140	bacteria>proteobacteria>alphaproteobacteria	Novosphingobium sp. ST904	attachment protein [Novosphingobium sp. ST904].
WP_054947356.1	-	140	bacteria>proteobacteria>alphaproteobacteria	Novosphingobium sp. KN65.2	attachment protein [Novosphingobium sp. KN65.2].
WP_056444504.1	-	140	bacteria>proteobacteria>alphaproteobacteria	Caulobacter sp. Root1455	hypothetical protein [Caulobacter sp. Root1455].
WP_056460713.1	-	140	bacteria>proteobacteria>alphaproteobacteria	Caulobacter sp. Root487D2Y	hypothetical protein [Caulobacter sp. Root487D2Y].
WP_058737612.1	-	140	bacteria>proteobacteria>alphaproteobacteria	Novosphingobium barchaimii	attachment protein [Novosphingobium barchaimii].
WP_059149875.1	-	140	bacteria>proteobacteria>alphaproteobacteria	Novosphingobium barchaimii	attachment protein [Novosphingobium barchaimii].
KWV92116.1	AUC45_13335	140	bacteria>proteobacteria>alphaproteobacteria	Erythrobacter sp. YT30	hypothetical protein AUC45_13335 [Erythrobacter sp. YT30].
KXV29778.1	AD936_18050	140	bacteria>proteobacteria>alphaproteobacteria	Gluconobacter japonicus	attachment protein [Gluconobacter japonicus].
WP_061929825.1	-	140	bacteria>proteobacteria>alphaproteobacteria	Gluconobacter japonicus	attachment protein [Gluconobacter japonicus].
WP_062025537.1	-	140	bacteria>proteobacteria>alphaproteobacteria	Gluconobacter japonicus	attachment protein [Gluconobacter japonicus].
WP_062031097.1	-	140	bacteria>proteobacteria>alphaproteobacteria	Gluconobacter albidus	attachment protein [Gluconobacter albidus].
WP_062504545.1	-	140	bacteria>proteobacteria>alphaproteobacteria	Gluconobacter japonicus	attachment protein [Gluconobacter japonicus].
WP_062511023.1	-	140	bacteria>proteobacteria>alphaproteobacteria	Gluconobacter roseus	attachment protein [Gluconobacter roseus].
WP_063905626.1	-	140	bacteria>proteobacteria>alphaproteobacteria	Gluconobacter japonicus	attachment protein [Gluconobacter japonicus].
WP_020988647.1	-	139	bacteria>spirochaetes	Leptospira inadai	host cell attachment protein [Leptospira inadai].
WP_023977894.1	-	139	bacteria>proteobacteria>alphaproteobacteria	Asaia	MULTISPECIES: attachment protein [Asaia].
WP_025840607.1	-	139	bacteria>proteobacteria>alphaproteobacteria	Asaia platycodi	attachment protein [Asaia platycodi].
WP_025884970.1	-	139	bacteria>proteobacteria>alphaproteobacteria	Asaia prunellae	attachment protein [Asaia prunellae].
WP_039392867.1	-	139	bacteria>proteobacteria>alphaproteobacteria	Novosphingobium sp. MBES04	attachment protein [Novosphingobium sp. MBES04].
WP_042058139.1	-	139	bacteria>proteobacteria>alphaproteobacteria	Acidomonas methanolica	attachment protein [Acidomonas methanolica].
KWV93414.1	AUC45_00670	139	bacteria>proteobacteria>alphaproteobacteria	Erythrobacter sp. YT30	Host attachment protein [Erythrobacter sp. YT30].
WP_004622779.1	-	138	bacteria>proteobacteria>alphaproteobacteria	Caulobacter vibrioides	protein required for attachment to host cell [Caulobacter vibrioides].
WP_008952415.1	-	138	bacteria>proteobacteria>betaproteobacteria	Pseudogulbenkiania ferrooxidans	host cell attachment-required protein [Pseudogulbenkiania ferrooxidans].
WP_010485385.1	-	138	bacteria	Proteobacteria	MULTISPECIES: attachment protein [Proteobacteria].
WP_012640514.1	-	138	bacteria>proteobacteria>alphaproteobacteria	Caulobacter vibrioides	attachment protein [Caulobacter vibrioides].
WP_013078356.1	-	138	bacteria>proteobacteria>alphaproteobacteria	Caulobacter segnis	attachment protein [Caulobacter segnis].
WP_014088414.1	-	138	bacteria>proteobacteria>betaproteobacteria	Pseudogulbenkiania sp. NH8B	host cell attachment-required protein [Pseudogulbenkiania sp. NH8B].
WP_024304107.1	-	138	bacteria>proteobacteria>betaproteobacteria	Pseudogulbenkiania sp. MAI-1	host cell attachment-required protein [Pseudogulbenkiania sp. MAI-1].
KFL44807.1	IL54_0173	138	bacteria>proteobacteria>alphaproteobacteria	Sphingobium sp. ba1	hypothetical protein IL54_0173 [Sphingobium sp. ba1].
WP_035371461.1	-	138	bacteria>proteobacteria>alphaproteobacteria	Acetobacter papayae	peptidase [Acetobacter papayae].
WP_056344947.1	-	138	bacteria>proteobacteria>betaproteobacteria	Massilia sp. Leaf139	hypothetical protein [Massilia sp. Leaf139].
WP_062093466.1	-	138	bacteria>proteobacteria>alphaproteobacteria	Caulobacter sp. CCH5-E12	attachment protein [Caulobacter sp. CCH5-E12].
OAI16096.1	A1359_08750	138	bacteria>proteobacteria>gammaproteobacteria	Methylomonas lenta	hypothetical protein A1359_08750 [Methylomonas lenta].
WP_064992360.1	-	138	bacteria>proteobacteria>alphaproteobacteria	Mesorhizobium loti	host cell attachment protein [Mesorhizobium loti].
WP_012287545.1	-	137	bacteria>proteobacteria>alphaproteobacteria	Caulobacter sp. K31	attachment protein [Caulobacter sp. K31].
WP_014147786.1	-	137	bacteria>proteobacteria>gammaproteobacteria	Methylomicrobium alcaliphilum	hypothetical protein [Methylomicrobium alcaliphilum].
WP_017839867.1	-	137	bacteria>proteobacteria>gammaproteobacteria	Methylomicrobium buryatense	hypothetical protein [Methylomicrobium buryatense].
WP_018687377.1	-	137	bacteria>proteobacteria>alphaproteobacteria	Ahrensia kielensis	hypothetical protein [Ahrensia kielensis].
WP_023508230.1	-	137	bacteria>proteobacteria>alphaproteobacteria	Rickettsia monacensis	protein required for attachment to host cells [Rickettsia monacensis].
WP_026480000.1	-	137	bacteria>proteobacteria>alphaproteobacteria	Ahrensia sp. 13_GOM-1096m	hypothetical protein [Ahrensia sp. 13_GOM-1096m].
WP_035373240.1	-	137	bacteria>proteobacteria>alphaproteobacteria	Acetobacter persici	peptidase [Acetobacter persici].
WP_035382419.1	-	137	bacteria>proteobacteria>alphaproteobacteria	Acetobacter tropicalis	peptidase [Acetobacter tropicalis].
WP_039903160.1	-	137	bacteria>proteobacteria>alphaproteobacteria	Acetobacter tropicalis	peptidase [Acetobacter tropicalis].
WP_042788021.1	-	137	bacteria>proteobacteria>alphaproteobacteria	Acetobacter aceti	peptidase [Acetobacter aceti].
WP_043549288.1	-	137	bacteria>proteobacteria>alphaproteobacteria	Acetobacter malorum	peptidase [Acetobacter malorum].
WP_045542570.1	-	137	bacteria>proteobacteria>alphaproteobacteria	Acetobacter tropicalis	peptidase [Acetobacter tropicalis].
WP_048835082.1	-	137	bacteria>proteobacteria>alphaproteobacteria	Acetobacter orleanensis	peptidase [Acetobacter orleanensis].
WP_048837513.1	-	137	bacteria>proteobacteria>alphaproteobacteria	Acetobacter cibinongensis	peptidase [Acetobacter cibinongensis].
WP_048842237.1	-	137	bacteria>proteobacteria>alphaproteobacteria	Acetobacter orientalis	peptidase [Acetobacter orientalis].
WP_048846322.1	-	137	bacteria>proteobacteria>alphaproteobacteria	Acetobacter indonesiensis	peptidase [Acetobacter indonesiensis].
WP_048853373.1	-	137	bacteria>proteobacteria>alphaproteobacteria	Acetobacter syzygii	peptidase [Acetobacter syzygii].
WP_051306247.1	-	137	bacteria>proteobacteria>betaproteobacteria	Massilia alkalitolerans	hypothetical protein [Massilia alkalitolerans].
WP_051454546.1	-	137	bacteria>proteobacteria>alphaproteobacteria	Microvirga lupini	hypothetical protein [Microvirga lupini].
WP_053997906.1	-	137	bacteria>proteobacteria>alphaproteobacteria	Ahrensia marina	hypothetical protein [Ahrensia marina].
WP_056102258.1	-	137	bacteria>proteobacteria>alphaproteobacteria	Brevundimonas sp. Leaf363	host cell attachment protein [Brevundimonas sp. Leaf363].
WP_056445983.1	-	137	bacteria>proteobacteria>alphaproteobacteria	Caulobacter	MULTISPECIES: attachment protein [Caulobacter].
WP_058988134.1	-	137	bacteria>proteobacteria>alphaproteobacteria	Acetobacter senegalensis	peptidase [Acetobacter senegalensis].
WP_061490631.1	-	137	bacteria>proteobacteria>alphaproteobacteria	Acetobacter malorum	peptidase [Acetobacter malorum].
WP_061502711.1	-	137	bacteria>proteobacteria>alphaproteobacteria	Acetobacter malorum	peptidase [Acetobacter malorum].
WP_062142200.1	-	137	bacteria>proteobacteria>alphaproteobacteria	Acetobacter cerevisiae	peptidase [Acetobacter cerevisiae].
WP_062249444.1	-	137	bacteria>proteobacteria>alphaproteobacteria	Acetobacter cerevisiae	peptidase [Acetobacter cerevisiae].
OCW57888.1	AWJ14_03605	137	bacteria>proteobacteria>alphaproteobacteria	Hoeflea olei	hypothetical protein AWJ14_03605 [Hoeflea olei].
WP_008291060.1	-	136	bacteria>proteobacteria>gammaproteobacteria	Methylophaga thiooxydans	hypothetical protein [Methylophaga thiooxydans].
WP_010668811.1	-	136	bacteria>proteobacteria>alphaproteobacteria	Acetobacter aceti	hypothetical protein [Acetobacter aceti].
WP_026398041.1	-	136	bacteria>proteobacteria>alphaproteobacteria	Acetobacter nitrogenifigens	peptidase [Acetobacter nitrogenifigens].
WP_028220285.1	-	136	bacteria>proteobacteria>betaproteobacteria	Paraburkholderia oxyphila	hypothetical protein [Paraburkholderia oxyphila].
KGM08072.1	LP43_0495	136	bacteria>proteobacteria>gammaproteobacteria	Methylophaga thiooxydans	hypothetical protein LP43_0495 [Methylophaga thiooxydans].
WP_035527803.1	-	136	bacteria>proteobacteria>alphaproteobacteria	Hoeflea sp. BAL378	hypothetical protein [Hoeflea sp. BAL378].
WP_036798164.1	-	136	bacteria>proteobacteria>alphaproteobacteria	Porphyrobacter sp. HL-46	attachment protein [Porphyrobacter sp. HL-46].
KJS19310.1	VR78_03535	136	bacteria>proteobacteria>alphaproteobacteria	Hoeflea sp. BRH_c9	hypothetical protein VR78_03535 [Hoeflea sp. BRH_c9].
WP_047029615.1	-	136	bacteria>proteobacteria>alphaproteobacteria	Hoeflea sp. IMCC20628	hypothetical protein [Hoeflea sp. IMCC20628].
WP_054309885.1	-	136	bacteria>proteobacteria>alphaproteobacteria	Mesorhizobium sp. 1M-11	host cell attachment protein [Mesorhizobium sp. 1M-11].
KZX52537.1	A3711_06310	136	bacteria>proteobacteria>alphaproteobacteria	Erythrobacter sp. HI00D59	attachment protein [Erythrobacter sp. HI00D59].
KZY56934.1	A3736_06930	136	bacteria>proteobacteria>alphaproteobacteria	Erythrobacter sp. HI0063	attachment protein [Erythrobacter sp. HI0063].
OBX19754.1	A9995_04105	136	bacteria>proteobacteria>alphaproteobacteria	Erythrobacter sp. QSSC1-22B	attachment protein [Erythrobacter sp. QSSC1-22B].
WP_006833054.1	-	135	bacteria>proteobacteria>alphaproteobacteria	Erythrobacter sp. SD-21	attachment protein [Erythrobacter sp. SD-21].
WP_010233101.1	-	135	bacteria>proteobacteria>alphaproteobacteria	Citromicrobium	MULTISPECIES: attachment protein [Citromicrobium].
WP_010409873.1	-	135	bacteria>proteobacteria>alphaproteobacteria	Citromicrobium sp. JLT1363	attachment protein [Citromicrobium sp. JLT1363].
WP_011415783.1	-	135	bacteria>proteobacteria>alphaproteobacteria	Erythrobacter litoralis	attachment protein [Erythrobacter litoralis].
WP_011806968.1	-	135	bacteria>proteobacteria>betaproteobacteria	Acidovorax sp. JS42	hypothetical protein [Acidovorax sp. JS42].
WP_027069520.1	-	135	bacteria>proteobacteria>gammaproteobacteria	Lysobacter defluvii	attachment protein [Lysobacter defluvii].
WP_029619788.1	-	135	bacteria>proteobacteria>alphaproteobacteria	Rhizobium sp. MGL06	attachment protein [Rhizobium sp. MGL06].
WP_034220786.1	-	135	bacteria>proteobacteria>gammaproteobacteria	Arenimonas donghaensis	attachment protein [Arenimonas donghaensis].
WP_034906564.1	-	135	bacteria>proteobacteria>alphaproteobacteria	Erythrobacter litoralis	attachment protein [Erythrobacter litoralis].
WP_036185021.1	-	135	bacteria>proteobacteria>gammaproteobacteria	Marinobacterium sp. AK27	host cell attachment-required protein [Marinobacterium sp. AK27].
WP_037167321.1	-	135	bacteria>proteobacteria>alphaproteobacteria	Rhizobium	MULTISPECIES: attachment protein [Rhizobium].
WP_037514908.1	-	135	bacteria>proteobacteria>alphaproteobacteria	Sphingomonas sp. PAMC 26621	host cell attachment protein [Sphingomonas sp. PAMC 26621].
WP_039097317.1	-	135	bacteria>proteobacteria>alphaproteobacteria	Porphyrobacter mercurialis	attachment protein [Porphyrobacter mercurialis].
WP_040966283.1	-	135	bacteria>proteobacteria>alphaproteobacteria	Erythrobacter vulgaris	attachment protein [Erythrobacter vulgaris].
WP_046904155.1	-	135	bacteria>proteobacteria>alphaproteobacteria	Altererythrobacter atlanticus	attachment protein [Altererythrobacter atlanticus].
WP_047004180.1	-	135	bacteria>proteobacteria>alphaproteobacteria	Erythrobacter luteus	attachment protein [Erythrobacter luteus].
WP_047007089.1	-	135	bacteria>proteobacteria>alphaproteobacteria	Erythrobacter gangjinensis	attachment protein [Erythrobacter gangjinensis].
WP_047092787.1	-	135	bacteria>proteobacteria>alphaproteobacteria	Erythrobacter marinus	attachment protein [Erythrobacter marinus].
WP_047349277.1	-	135	bacteria>proteobacteria>betaproteobacteria	Diaphorobacter sp. J5-51	hypothetical protein [Diaphorobacter sp. J5-51].
WP_047806944.1	-	135	bacteria>proteobacteria>alphaproteobacteria	Altererythrobacter marensis	attachment protein [Altererythrobacter marensis].
WP_047822191.1	-	135	bacteria>proteobacteria>alphaproteobacteria	Croceicoccus naphthovorans	attachment protein [Croceicoccus naphthovorans].
WP_048884786.1	-	135	bacteria>proteobacteria>alphaproteobacteria	Erythrobacter atlanticus	attachment protein [Erythrobacter atlanticus].
WP_050601737.1	-	135	bacteria>proteobacteria>alphaproteobacteria	Erythrobacter	MULTISPECIES: attachment protein [Erythrobacter].
KPP94043.1	HLUCCO15_04820	135	bacteria>proteobacteria>alphaproteobacteria	Erythrobacteraceae bacterium HL-111	hypothetical protein HLUCCO15_04820 [Erythrobacteraceae bacterium HL-111].
WP_055925058.1	-	135	bacteria>proteobacteria>alphaproteobacteria	Altererythrobacter sp. Root672	attachment protein [Altererythrobacter sp. Root672].
WP_056100379.1	-	135	bacteria>proteobacteria>alphaproteobacteria	Mesorhizobium sp. Root157	hypothetical protein [Mesorhizobium sp. Root157].
KWV93936.1	ASS64_13695	135	bacteria>proteobacteria>alphaproteobacteria	Erythrobacter sp. AP23	attachment protein [Erythrobacter sp. AP23].
WP_061927404.1	-	135	bacteria>proteobacteria>alphaproteobacteria	Altererythrobacter epoxidivorans	attachment protein [Altererythrobacter epoxidivorans].
ANK13156.1	A9D12_09600	135	bacteria>proteobacteria>alphaproteobacteria	Porphyrobacter neustonensis	hypothetical protein A9D12_09600 [Porphyrobacter neustonensis].
ANU06527.1	A6F65_00200	135	bacteria>proteobacteria>alphaproteobacteria	Altererythrobacter namhicola	host cell attachment protein [Altererythrobacter namhicola].
CAK03746.1	pRL90036	134	bacteria>proteobacteria>alphaproteobacteria	Rhizobium leguminosarum bv. viciae 3841	putative attachment-related protein (plasmid) [Rhizobium leguminosarum bv. viciae 3841].
WP_003166088.1	-	134	bacteria>proteobacteria>alphaproteobacteria	Brevundimonas diminuta	hypothetical protein [Brevundimonas diminuta].
WP_003167835.1	-	134	bacteria>proteobacteria>alphaproteobacteria	Brevundimonas diminuta	hypothetical protein [Brevundimonas diminuta].
WP_003517267.1	-	134	bacteria>proteobacteria>alphaproteobacteria	Agrobacterium tumefaciens	hypothetical protein [Agrobacterium tumefaciens].
WP_006337414.1	-	134	bacteria>proteobacteria>alphaproteobacteria	Mesorhizobium sp. STM 4661	host cell attachment protein [Mesorhizobium sp. STM 4661].
WP_006727153.1	-	134	bacteria>proteobacteria>alphaproteobacteria	Agrobacterium albertimagni	hypothetical protein [Agrobacterium albertimagni].
WP_007598137.1	-	134	bacteria>proteobacteria>alphaproteobacteria	Rhizobium sp. PDO1-076	hypothetical protein [Rhizobium sp. PDO1-076].
WP_008994367.1	-	134	bacteria>proteobacteria>alphaproteobacteria	Novosphingobium sp. Rr 2-17	hypothetical protein [Novosphingobium sp. Rr 2-17].
WP_009449898.1	-	134	bacteria>proteobacteria>alphaproteobacteria	Nitratireductor indicus	hypothetical protein [Nitratireductor indicus].
WP_009453012.1	-	134	bacteria>proteobacteria>alphaproteobacteria	Nitratireductor indicus	hypothetical protein [Nitratireductor indicus].
WP_010916107.1	-	134	bacteria>proteobacteria>alphaproteobacteria	Mesorhizobium loti	hypothetical protein [Mesorhizobium loti].
WP_011445629.1	-	134	bacteria>proteobacteria>alphaproteobacteria	Novosphingobium aromaticivorans	host cell attachment protein [Novosphingobium aromaticivorans].
WP_011472706.1	-	134	bacteria>proteobacteria>alphaproteobacteria	Rhodopseudomonas palustris	hypothetical protein [Rhodopseudomonas palustris].
WP_011662090.1	-	134	bacteria>proteobacteria>alphaproteobacteria	Rhodopseudomonas palustris	hypothetical protein [Rhodopseudomonas palustris].
WP_013536322.1	-	134	bacteria>proteobacteria>gammaproteobacteria	Pseudoxanthomonas suwonensis	host attachment protein [Pseudoxanthomonas suwonensis].
WP_013637403.1	-	134	bacteria>proteobacteria>alphaproteobacteria	Agrobacterium sp. H13-3	hypothetical protein [Agrobacterium sp. H13-3].
WP_014493429.1	-	134	bacteria>proteobacteria>alphaproteobacteria	Bradyrhizobium japonicum	hypothetical protein [Bradyrhizobium japonicum].
WP_015448028.1	-	134	bacteria>proteobacteria>gammaproteobacteria	Rhodanobacter denitrificans	protein required for attachment to host cells [Rhodanobacter denitrificans].
WP_018485459.1	-	134	bacteria>proteobacteria>alphaproteobacteria	Rhizobium leguminosarum	hypothetical protein [Rhizobium leguminosarum].
WP_018494579.1	-	134	bacteria>proteobacteria>alphaproteobacteria	Rhizobium leguminosarum	hypothetical protein [Rhizobium leguminosarum].
WP_019565720.1	-	134	bacteria>proteobacteria>alphaproteobacteria	Rhizobium/Agrobacterium group	MULTISPECIES: hypothetical protein [Rhizobium/Agrobacterium group].
WP_019935582.1	-	134	bacteria>proteobacteria>gammaproteobacteria	Oceanimonas smirnovii	hypothetical protein [Oceanimonas smirnovii].
WP_024849352.1	-	134	bacteria>proteobacteria>alphaproteobacteria	Aminobacter sp. J41	hypothetical protein [Aminobacter sp. J41].
WP_024870158.1	-	134	bacteria>proteobacteria>gammaproteobacteria	Pseudoxanthomonas suwonensis	host attachment protein [Pseudoxanthomonas suwonensis].
WP_025591460.1	-	134	bacteria>proteobacteria>alphaproteobacteria	Agrobacterium tumefaciens	hypothetical protein [Agrobacterium tumefaciens].
WP_027047156.1	-	134	bacteria>proteobacteria>alphaproteobacteria	Mesorhizobium loti	hypothetical protein [Mesorhizobium loti].
WP_027054295.1	-	134	bacteria>proteobacteria>alphaproteobacteria	Mesorhizobium erdmanii	hypothetical protein [Mesorhizobium erdmanii].
WP_027061998.1	-	134	bacteria>proteobacteria>alphaproteobacteria	Mesorhizobium loti	hypothetical protein [Mesorhizobium loti].
WP_027484324.1	-	134	bacteria>proteobacteria>gammaproteobacteria	Rhodanobacter sp. OR87	hypothetical protein [Rhodanobacter sp. OR87].
WP_028150437.1	-	134	bacteria>proteobacteria>alphaproteobacteria	Bradyrhizobium japonicum	hypothetical protein [Bradyrhizobium japonicum].
WP_028167118.1	-	134	bacteria>proteobacteria>alphaproteobacteria	Bradyrhizobium elkanii	hypothetical protein [Bradyrhizobium elkanii].
WP_028337084.1	-	134	bacteria>proteobacteria>alphaproteobacteria	Bradyrhizobium elkanii	hypothetical protein [Bradyrhizobium elkanii].
WP_028341929.1	-	134	bacteria>proteobacteria>alphaproteobacteria	Bradyrhizobium elkanii	hypothetical protein [Bradyrhizobium elkanii].
WP_029583591.1	-	134	bacteria>proteobacteria>alphaproteobacteria	Bradyrhizobium sp. URHD0069	hypothetical protein [Bradyrhizobium sp. URHD0069].
WP_029620381.1	-	134	bacteria>proteobacteria>alphaproteobacteria	Rhizobium sp. MGL06	hypothetical protein [Rhizobium sp. MGL06].
WP_029623812.1	-	134	bacteria>proteobacteria>alphaproteobacteria	Sphingomonas sp. PAMC 26617	hypothetical protein [Sphingomonas sp. PAMC 26617].
WP_031392868.1	-	134	bacteria>proteobacteria>alphaproteobacteria	Sphingomonas sp. STIS6.2	hypothetical protein [Sphingomonas sp. STIS6.2].
WP_033183988.1	-	134	bacteria>proteobacteria>alphaproteobacteria	Rhizobium leguminosarum	host cell attachment protein [Rhizobium leguminosarum].
WP_033184558.1	-	134	bacteria>proteobacteria>alphaproteobacteria	Rhizobium leguminosarum	host cell attachment protein [Rhizobium leguminosarum].
WP_035031096.1	-	134	bacteria	Proteobacteria	MULTISPECIES: host cell attachment protein [Proteobacteria].
WP_035302822.1	-	134	bacteria>proteobacteria>alphaproteobacteria	Brevundimonas naejangsanensis	host cell attachment protein [Brevundimonas naejangsanensis].
WP_035690434.1	-	134	bacteria>proteobacteria>alphaproteobacteria	Bradyrhizobium elkanii	host cell attachment protein [Bradyrhizobium elkanii].
WP_037088593.1	-	134	bacteria>proteobacteria>alphaproteobacteria	Rhizobium vignae	host cell attachment protein [Rhizobium vignae].
WP_037163587.1	-	134	bacteria>proteobacteria>alphaproteobacteria	Rhizobium	MULTISPECIES: host cell attachment protein [Rhizobium].
WP_039152920.1	-	134	bacteria>proteobacteria>alphaproteobacteria	Bradyrhizobium japonicum	host cell attachment protein [Bradyrhizobium japonicum].
WP_039192016.1	-	134	bacteria>proteobacteria>alphaproteobacteria	Aureimonas altamirensis	host cell attachment protein [Aureimonas altamirensis].
WP_040971269.1	-	134	bacteria>proteobacteria>alphaproteobacteria	Mesorhizobium sp. ORS3324	host cell attachment protein [Mesorhizobium sp. ORS3324].
WP_041005200.1	-	134	bacteria>proteobacteria>alphaproteobacteria	Mesorhizobium plurifarium	host cell attachment protein [Mesorhizobium plurifarium].
WP_041005710.1	-	134	bacteria>proteobacteria>alphaproteobacteria	Mesorhizobium plurifarium	host cell attachment protein [Mesorhizobium plurifarium].
WP_041365264.1	-	134	bacteria>proteobacteria>alphaproteobacteria	Neorhizobium galegae	host cell attachment protein [Neorhizobium galegae].
WP_041958911.1	-	134	bacteria>proteobacteria>alphaproteobacteria	Bradyrhizobium japonicum	host cell attachment protein [Bradyrhizobium japonicum].
WP_042616971.1	-	134	bacteria>proteobacteria>alphaproteobacteria	Agrobacterium tumefaciens	host cell attachment protein [Agrobacterium tumefaciens].
WP_042638558.1	-	134	bacteria>proteobacteria>alphaproteobacteria	Mesorhizobium sp. ORS3359	host cell attachment protein [Mesorhizobium sp. ORS3359].
WP_043152600.1	-	134	bacteria>proteobacteria>alphaproteobacteria	Sphingobium sp. Ant17	host cell attachment protein [Sphingobium sp. Ant17].
WP_044419249.1	-	134	bacteria>proteobacteria>alphaproteobacteria	Rhodopseudomonas palustris	host cell attachment protein [Rhodopseudomonas palustris].
WP_045229589.1	-	134	bacteria>proteobacteria>alphaproteobacteria	Agrobacterium rubi	host cell attachment protein [Agrobacterium rubi].
WP_046606047.1	-	134	bacteria>proteobacteria>alphaproteobacteria	Neorhizobium galegae	host cell attachment protein [Neorhizobium galegae].
WP_046630396.1	-	134	bacteria>proteobacteria>alphaproteobacteria	Neorhizobium galegae	host cell attachment protein [Neorhizobium galegae].
WP_047562578.1	-	134	bacteria>proteobacteria>alphaproteobacteria	Mesorhizobium sp. F7	host attachment protein [Mesorhizobium sp. F7].
WP_050387301.1	-	134	bacteria>proteobacteria>alphaproteobacteria	Bradyrhizobium pachyrhizi	host cell attachment protein [Bradyrhizobium pachyrhizi].
WP_051412492.1	-	134	bacteria>proteobacteria>gammaproteobacteria	Pseudoxanthomonas sp. J35	host attachment protein [Pseudoxanthomonas sp. J35].
WP_051414628.1	-	134	bacteria>proteobacteria>gammaproteobacteria	Pseudoxanthomonas suwonensis	host attachment protein [Pseudoxanthomonas suwonensis].
WP_052382587.1	-	134	bacteria>proteobacteria>betaproteobacteria	Comamonas granuli	hypothetical protein [Comamonas granuli].
WP_052632046.1	-	134	bacteria>proteobacteria>gammaproteobacteria	Pseudoxanthomonas suwonensis	host attachment protein [Pseudoxanthomonas suwonensis].
CDX58321.1	MPL1032_240016	134	bacteria>proteobacteria>alphaproteobacteria	Mesorhizobium plurifarium	putative attachment-related protein [Mesorhizobium plurifarium].
WP_054108390.1	-	134	bacteria>proteobacteria>alphaproteobacteria	Novosphingobium sp. AAP83	hypothetical protein [Novosphingobium sp. AAP83].
WP_054128036.1	-	134	bacteria>proteobacteria>alphaproteobacteria	alpha proteobacterium AAP81b	host cell attachment protein [alpha proteobacterium AAP81b].
WP_054144623.1	-	134	bacteria>proteobacteria>alphaproteobacteria	Bosea sp. AAP35	hypothetical protein [Bosea sp. AAP35].
WP_054151135.1	-	134	bacteria>proteobacteria>alphaproteobacteria	Rhizobium sp. AAP116	host cell attachment protein [Rhizobium sp. AAP116].
WP_054156737.1	-	134	bacteria>proteobacteria>alphaproteobacteria	Rhizobium sp. AAP43	host cell attachment protein [Rhizobium sp. AAP43].
WP_054210158.1	-	134	bacteria>proteobacteria>alphaproteobacteria	Bosea vaviloviae	hypothetical protein [Bosea vaviloviae].
WP_054529751.1	-	134	bacteria>proteobacteria>alphaproteobacteria	Erythrobacter sp. SG61-1L	hypothetical protein [Erythrobacter sp. SG61-1L].
WP_055850521.1	-	134	bacteria>proteobacteria>alphaproteobacteria	Aurantimonas sp. Leaf443	host cell attachment protein [Aurantimonas sp. Leaf443].
WP_055892415.1	-	134	bacteria>proteobacteria>alphaproteobacteria	Aureimonas sp. Leaf324	host cell attachment protein [Aureimonas sp. Leaf324].
WP_056102729.1	-	134	bacteria>proteobacteria>alphaproteobacteria	Mesorhizobium sp. Root157	host cell attachment protein [Mesorhizobium sp. Root157].
WP_056115471.1	-	134	bacteria>proteobacteria>alphaproteobacteria	Mesorhizobium	MULTISPECIES: host cell attachment protein [Mesorhizobium].
WP_056242247.1	-	134	bacteria>proteobacteria>alphaproteobacteria	Mesorhizobium sp. Root552	host cell attachment protein [Mesorhizobium sp. Root552].
WP_056385310.1	-	134	bacteria>proteobacteria>gammaproteobacteria	Rhodanobacter sp. Soil772	hypothetical protein [Rhodanobacter sp. Soil772].
WP_056500929.1	-	134	bacteria>proteobacteria>alphaproteobacteria	Aureimonas sp. Leaf454	host cell attachment protein [Aureimonas sp. Leaf454].
WP_056536660.1	-	134	bacteria>proteobacteria>alphaproteobacteria	Rhizobium sp. Root1220	host cell attachment protein [Rhizobium sp. Root1220].
WP_056590630.1	-	134	bacteria>proteobacteria>alphaproteobacteria	Mesorhizobium	MULTISPECIES: host cell attachment protein [Mesorhizobium].
WP_056698547.1	-	134	bacteria>proteobacteria>alphaproteobacteria	Aureimonas	MULTISPECIES: host cell attachment protein [Aureimonas].
WP_057015981.1	-	134	bacteria>proteobacteria>alphaproteobacteria	Bradyrhizobium pachyrhizi	host cell attachment protein [Bradyrhizobium pachyrhizi].
WP_057844341.1	-	134	bacteria>proteobacteria>alphaproteobacteria	Bradyrhizobium retamae	host cell attachment protein [Bradyrhizobium retamae].
BAT59505.1	GJW-30_1_02038	134	bacteria>proteobacteria>alphaproteobacteria	Variibacter gotjawalensis	protein required for attachment to host cells [Variibacter gotjawalensis].
WP_058599219.1	-	134	bacteria>proteobacteria>alphaproteobacteria	Aureimonas ureilytica	host cell attachment protein [Aureimonas ureilytica].
WP_060600345.1	-	134	bacteria>proteobacteria>alphaproteobacteria	Aureimonas altamirensis	host cell attachment protein [Aureimonas altamirensis].
WP_060639526.1	-	134	bacteria>proteobacteria>alphaproteobacteria	Rhizobium sp. Root483D2	host cell attachment protein [Rhizobium sp. Root483D2].
WP_060726757.1	-	134	bacteria>proteobacteria>alphaproteobacteria	Agrobacterium tumefaciens	host cell attachment protein [Agrobacterium tumefaciens].
KWV60388.1	AS156_28730	134	bacteria>proteobacteria>alphaproteobacteria	Bradyrhizobium sp. BR 10303	host cell attachment protein [Bradyrhizobium sp. BR 10303].
KXF75856.1	ATN84_18040	134	bacteria>proteobacteria>alphaproteobacteria	Paramesorhizobium deserti	host cell attachment protein [Paramesorhizobium deserti].
AMJ62516.1	AXW83_21395	134	bacteria>proteobacteria>alphaproteobacteria	Bosea sp. PAMC 26642	hypothetical protein AXW83_21395 [Bosea sp. PAMC 26642].
KXG85156.1	ATO67_08025	134	bacteria>proteobacteria>alphaproteobacteria	Agrobacterium sp. R89-1	host cell attachment protein [Agrobacterium sp. R89-1].
CZT34066.1	-	134	bacteria>proteobacteria>alphaproteobacteria	Rhizobium sp. 9140	Protein required for attachment to host cells [Rhizobium sp. 9140].
WP_061938319.1	-	134	bacteria>proteobacteria>alphaproteobacteria	Aureimonas sp. AU22	host cell attachment protein [Aureimonas sp. AU22].
WP_061979067.1	-	134	bacteria>proteobacteria>alphaproteobacteria	Aureimonas	MULTISPECIES: host cell attachment protein [Aureimonas].
WP_062016182.1	-	134	bacteria>proteobacteria>alphaproteobacteria	Aureimonas sp. AU4	host cell attachment protein [Aureimonas sp. AU4].
WP_062206070.1	-	134	bacteria>proteobacteria>alphaproteobacteria	Aureimonas sp. AU12	host cell attachment protein [Aureimonas sp. AU12].
WP_062224423.1	-	134	bacteria>proteobacteria>alphaproteobacteria	Aureimonas sp. D3	host cell attachment protein [Aureimonas sp. D3].
WP_062227611.1	-	134	bacteria>proteobacteria>alphaproteobacteria	Aureimonas frigidaquae	host cell attachment protein [Aureimonas frigidaquae].
WP_062458727.1	-	134	bacteria>proteobacteria>alphaproteobacteria	Rhizobium sp. Leaf306	host cell attachment protein [Rhizobium sp. Leaf306].
WP_062468611.1	-	134	bacteria>proteobacteria>alphaproteobacteria	Rhizobium	MULTISPECIES: host cell attachment protein [Rhizobium].
WP_062576571.1	-	134	bacteria>proteobacteria>alphaproteobacteria	Rhizobium sp. Leaf321	host cell attachment protein [Rhizobium sp. Leaf321].
WP_062587869.1	-	134	bacteria>proteobacteria>alphaproteobacteria	Rhizobium sp. Leaf311	host cell attachment protein [Rhizobium sp. Leaf311].
WP_062596589.1	-	134	bacteria>proteobacteria>alphaproteobacteria	Rhizobium sp. Leaf371	host cell attachment protein [Rhizobium sp. Leaf371].
WP_062941740.1	-	134	bacteria>proteobacteria>alphaproteobacteria	Rhizobium leguminosarum	host cell attachment protein [Rhizobium leguminosarum].
KZC17356.1	RHOFW104R8_12050	134	bacteria>proteobacteria>gammaproteobacteria	Rhodanobacter sp. FW104-R8	hypothetical protein RHOFW104R8_12050 [Rhodanobacter sp. FW104-R8].
WP_063981738.1	-	134	bacteria>proteobacteria>alphaproteobacteria	Bradyrhizobium sp. G22	host cell attachment protein [Bradyrhizobium sp. G22].
WP_064652312.1	-	134	bacteria>proteobacteria>alphaproteobacteria	Rhizobium sp. WYCCWR10014	host cell attachment protein [Rhizobium sp. WYCCWR10014].
OCC24797.1	MB02_04830	134	bacteria>proteobacteria>alphaproteobacteria	Altererythrobacter sp. MH-B5	attachment protein [Altererythrobacter sp. MH-B5].
WP_065687578.1	-	134	bacteria>proteobacteria>alphaproteobacteria	Agrobacterium tumefaciens	host cell attachment protein [Agrobacterium tumefaciens].
WP_065699226.1	-	134	bacteria>proteobacteria>alphaproteobacteria	Agrobacterium rubi	host cell attachment protein [Agrobacterium rubi].
WP_065703129.1	-	134	bacteria>proteobacteria>alphaproteobacteria	Agrobacterium sp. 13-2099-1-2	host cell attachment protein [Agrobacterium sp. 13-2099-1-2].
WP_065752360.1	-	134	bacteria>proteobacteria>alphaproteobacteria	Bradyrhizobium paxllaeri	host cell attachment protein [Bradyrhizobium paxllaeri].
BAV46465.1	MLTONO_1562	134	bacteria>proteobacteria>alphaproteobacteria	Mesorhizobium loti	AtsE protein [Mesorhizobium loti].
BAV47047.1	MLTONO_2144	134	bacteria>proteobacteria>alphaproteobacteria	Mesorhizobium loti	Putative attachment-related protein [Mesorhizobium loti].
BAV48054.1	MLTONO_3151	134	bacteria>proteobacteria>alphaproteobacteria	Mesorhizobium loti	AtsE protein [Mesorhizobium loti].
WP_002718159.1	-	133	bacteria>proteobacteria>alphaproteobacteria	Afipia	MULTISPECIES: hypothetical protein [Afipia].
WP_014291972.1	-	133	bacteria>proteobacteria>gammaproteobacteria	Oceanimonas sp. GK1	host cell attachment-required protein [Oceanimonas sp. GK1].
WP_025978619.1	-	133	bacteria>proteobacteria>alphaproteobacteria	Brevundimonas naejangsanensis	hypothetical protein [Brevundimonas naejangsanensis].
WP_026010608.1	-	133	bacteria>proteobacteria>gammaproteobacteria	Pseudoxanthomonas	MULTISPECIES: host attachment protein [Pseudoxanthomonas].
WP_027582956.1	-	133	bacteria>proteobacteria>alphaproteobacteria	Bradyrhizobium sp. Ai1a-2	hypothetical protein [Bradyrhizobium sp. Ai1a-2].
WP_043838692.1	-	133	bacteria>proteobacteria>alphaproteobacteria	Roseomonas aerilata	attachment protein [Roseomonas aerilata].
WP_046134042.1	-	133	bacteria>proteobacteria>alphaproteobacteria	Devosia limi	host cell attachment protein [Devosia limi].
WP_046172008.1	-	133	bacteria>proteobacteria>alphaproteobacteria	Devosia psychrophila	host cell attachment protein [Devosia psychrophila].
WP_055832637.1	-	133	bacteria>proteobacteria>alphaproteobacteria	Brevundimonas	MULTISPECIES: host cell attachment protein [Brevundimonas].
ANP45639.1	ATE48_06750	133	bacteria>proteobacteria>alphaproteobacteria	Caulobacteraceae bacterium OTSz_A_272	hypothetical protein ATE48_06750 [Caulobacteraceae bacterium OTSz_A_272].
ANP45834.1	ATE48_07800	133	bacteria>proteobacteria>alphaproteobacteria	Caulobacteraceae bacterium OTSz_A_272	hypothetical protein ATE48_07800 [Caulobacteraceae bacterium OTSz_A_272].
WP_002809798.1	-	132	bacteria>proteobacteria>gammaproteobacteria	Xanthomonas fragariae	protein required for attachment to host cell [Xanthomonas fragariae].
WP_005994775.1	-	132	bacteria>proteobacteria>gammaproteobacteria	Xanthomonas vesicatoria	attachment protein [Xanthomonas vesicatoria].
WP_008259789.1	-	132	bacteria>proteobacteria>alphaproteobacteria	Brevundimonas	MULTISPECIES: hypothetical protein [Brevundimonas].
WP_011157588.1	-	132	bacteria>proteobacteria>alphaproteobacteria	Rhodopseudomonas palustris	attachment protein [Rhodopseudomonas palustris].
WP_011257041.1	-	132	bacteria>proteobacteria>gammaproteobacteria	Xanthomonas oryzae	attachment protein [Xanthomonas oryzae].
WP_011442223.1	-	132	bacteria>proteobacteria>alphaproteobacteria	Rhodopseudomonas palustris	attachment protein [Rhodopseudomonas palustris].
WP_011473683.1	-	132	bacteria>proteobacteria>alphaproteobacteria	Rhodopseudomonas palustris	attachment protein [Rhodopseudomonas palustris].
WP_011502518.1	-	132	bacteria>proteobacteria>alphaproteobacteria	Rhodopseudomonas palustris	attachment protein [Rhodopseudomonas palustris].
WP_012284834.1	-	132	bacteria>proteobacteria>alphaproteobacteria	Caulobacter sp. K31	hypothetical protein [Caulobacter sp. K31].
WP_012443578.1	-	132	bacteria>proteobacteria>gammaproteobacteria	Xanthomonas oryzae	attachment protein [Xanthomonas oryzae].
WP_012495549.1	-	132	bacteria>proteobacteria>alphaproteobacteria	Rhodopseudomonas palustris	attachment protein [Rhodopseudomonas palustris].
WP_013267875.1	-	132	bacteria>proteobacteria>alphaproteobacteria	Brevundimonas subvibrioides	hypothetical protein [Brevundimonas subvibrioides].
WP_013503165.1	-	132	bacteria>proteobacteria>alphaproteobacteria	Rhodopseudomonas palustris	attachment protein [Rhodopseudomonas palustris].
WP_014501403.1	-	132	bacteria>proteobacteria>gammaproteobacteria	Xanthomonas oryzae	attachment protein [Xanthomonas oryzae].
WP_017504716.1	-	132	bacteria>proteobacteria>alphaproteobacteria	Brevundimonas	MULTISPECIES: hypothetical protein [Brevundimonas].
WP_017932053.1	-	132	bacteria>proteobacteria>alphaproteobacteria	Robiginitomaculum antarcticum	hypothetical protein [Robiginitomaculum antarcticum].
WP_019302284.1	-	132	bacteria>proteobacteria>gammaproteobacteria	Xanthomonas oryzae	attachment protein [Xanthomonas oryzae].
WP_024712268.1	-	132	bacteria>proteobacteria>gammaproteobacteria	Xanthomonas oryzae	attachment protein [Xanthomonas oryzae].
WP_029218222.1	-	132	bacteria>proteobacteria>gammaproteobacteria	Xanthomonas cassavae	attachment protein [Xanthomonas cassavae].
WP_039406470.1	-	132	bacteria>proteobacteria>gammaproteobacteria	Xanthomonas cannabis	attachment protein [Xanthomonas cannabis].
KJS08251.1	VR73_06350	132	bacteria>proteobacteria>gammaproteobacteria	Gammaproteobacteria bacterium BRH_c0	hypothetical protein VR73_06350 [Gammaproteobacteria bacterium BRH_c0].
WP_045804068.1	-	132	bacteria>proteobacteria>alphaproteobacteria	Rickettsia endosymbiont of Ixodes pacificus	hypothetical protein [Rickettsia endosymbiont of Ixodes pacificus].
WP_047308512.1	-	132	bacteria>proteobacteria>alphaproteobacteria	Rhodopseudomonas palustris	attachment protein [Rhodopseudomonas palustris].
WP_047339429.1	-	132	bacteria>proteobacteria>gammaproteobacteria	Xanthomonas oryzae	attachment protein [Xanthomonas oryzae].
WP_051648671.1	-	132	bacteria>proteobacteria>betaproteobacteria	Cupriavidus sp. SK-3	hypothetical protein [Cupriavidus sp. SK-3].
WP_054164373.1	-	132	bacteria>proteobacteria>alphaproteobacteria	Rhodopseudomonas sp. AAP120	attachment protein [Rhodopseudomonas sp. AAP120].
WP_054930863.1	-	132	bacteria>proteobacteria>betaproteobacteria	Burkholderiaceae	MULTISPECIES: hypothetical protein [Burkholderiaceae].
WP_055806084.1	-	132	bacteria>proteobacteria>alphaproteobacteria	Brevundimonas sp. Leaf168	host cell attachment protein [Brevundimonas sp. Leaf168].
WP_055852817.1	-	132	bacteria>proteobacteria>gammaproteobacteria	Xanthomonas sp. Leaf148	attachment protein [Xanthomonas sp. Leaf148].
WP_062918223.1	-	132	bacteria>proteobacteria>betaproteobacteria	Paraburkholderia caribensis	hypothetical protein [Paraburkholderia caribensis].
KZD20438.1	A4A58_19635	132	bacteria>proteobacteria>alphaproteobacteria	Tardiphaga sp. Vaf07	host cell attachment protein [Tardiphaga sp. Vaf07].
WP_064509302.1	-	132	bacteria>proteobacteria>gammaproteobacteria	Xanthomonas sp. WHRI 8848	attachment protein [Xanthomonas sp. WHRI 8848].
WP_064630151.1	-	132	bacteria>proteobacteria>gammaproteobacteria	Xanthomonas sp. WHRI 8853	attachment protein [Xanthomonas sp. WHRI 8853].
WP_006474260.1	-	131	bacteria>proteobacteria>gammaproteobacteria	endosymbiont of Tevnia jerichonana	host attachment protein [endosymbiont of Tevnia jerichonana].
WP_011663616.1	-	131	bacteria>proteobacteria>alphaproteobacteria	Rhodopseudomonas palustris	attachment protein [Rhodopseudomonas palustris].
WP_019459464.1	-	131	bacteria>proteobacteria>alphaproteobacteria	Roseomonas	MULTISPECIES: hypothetical protein [Roseomonas].
WP_020071432.1	-	131	bacteria>proteobacteria>betaproteobacteria	Paraburkholderia bryophila	hypothetical protein [Paraburkholderia bryophila].
WP_022724184.1	-	131	bacteria>proteobacteria>alphaproteobacteria	Rhodopseudomonas sp. B29	attachment protein [Rhodopseudomonas sp. B29].
WP_027281278.1	-	131	bacteria>proteobacteria>alphaproteobacteria	Roseomonas gilardii	attachment protein [Roseomonas gilardii].
WP_044407533.1	-	131	bacteria>proteobacteria>alphaproteobacteria	Rhodopseudomonas palustris	attachment protein [Rhodopseudomonas palustris].
WP_045455495.1	-	131	bacteria>proteobacteria>betaproteobacteria	Burkholderiaceae	MULTISPECIES: hypothetical protein [Burkholderiaceae].
WP_054659069.1	-	131	bacteria>proteobacteria>gammaproteobacteria	Stenotrophomonas maltophilia	hypothetical protein [Stenotrophomonas maltophilia].
WP_056440894.1	-	131	bacteria>proteobacteria>alphaproteobacteria	Caulobacter sp. Root1455	host cell attachment protein [Caulobacter sp. Root1455].
WP_056459824.1	-	131	bacteria>proteobacteria>alphaproteobacteria	Caulobacter sp. Root487D2Y	host cell attachment protein [Caulobacter sp. Root487D2Y].
WP_057955209.1	-	131	bacteria>proteobacteria>gammaproteobacteria	endosymbiont of Ridgeia piscesae	host attachment protein [endosymbiont of Ridgeia piscesae].
WP_058389839.1	-	131	bacteria>proteobacteria>alphaproteobacteria	Roseomonas mucosa	attachment protein [Roseomonas mucosa].
SAL36645.1	AWB73_03618	131	bacteria>proteobacteria>betaproteobacteria	Burkholderia turbans	Protein required for attachment to host cells [Burkholderia turbans].
SAK96063.1	AWB83_05676	131	bacteria>proteobacteria>betaproteobacteria	Burkholderia ptereochthonis	Protein required for attachment to host cells [Burkholderia ptereochthonis].
SAK95270.1	AWB75_06901	131	bacteria>proteobacteria>betaproteobacteria	Burkholderia catudaia	Protein required for attachment to host cells [Burkholderia catudaia].
SAK67815.1	AWB80_03353	131	bacteria>proteobacteria>betaproteobacteria	Burkholderia pedi	Protein required for attachment to host cells [Burkholderia pedi].
WP_032993236.1	-	130	bacteria>proteobacteria>alphaproteobacteria	Rhizobium leguminosarum	host cell attachment protein [Rhizobium leguminosarum].
WP_036206394.1	-	130	bacteria>proteobacteria>gammaproteobacteria	Lysobacter arseniciresistens	attachment protein [Lysobacter arseniciresistens].
WP_057661968.1	-	130	bacteria>proteobacteria>gammaproteobacteria	Stenotrophomonas koreensis	hypothetical protein [Stenotrophomonas koreensis].
WP_058835575.1	-	130	bacteria>proteobacteria>gammaproteobacteria	Luteimonas abyssi	attachment protein [Luteimonas abyssi].
AMO71147.1	AZE99_04145	130	bacteria>proteobacteria>alphaproteobacteria	Sphingorhabdus sp. M41	hypothetical protein AZE99_04145 [Sphingorhabdus sp. M41].
WP_062726227.1	-	130	bacteria>proteobacteria>gammaproteobacteria	Legionella pneumophila	hypothetical protein [Legionella pneumophila].
WP_013949636.1	-	129	bacteria>proteobacteria>alphaproteobacteria	Hyphomicrobium	MULTISPECIES: host attachment protein [Hyphomicrobium].
WP_039332263.1	-	128	bacteria>proteobacteria>alphaproteobacteria	Novosphingobium subterraneum	hypothetical protein [Novosphingobium subterraneum].
WP_062343568.1	-	128	bacteria>proteobacteria>alphaproteobacteria	Novosphingobium sp. CCH12-A3	hypothetical protein [Novosphingobium sp. CCH12-A3].
WP_063708273.1	-	128	bacteria>proteobacteria>alphaproteobacteria	Bradyrhizobium sp. BR 10245	host cell attachment protein [Bradyrhizobium sp. BR 10245].
WP_008066409.1	-	127	bacteria>proteobacteria>alphaproteobacteria	Novosphingobium nitrogenifigens	host cell attachment protein [Novosphingobium nitrogenifigens].
ESW60471.1	Q27BPR15_11785	127	bacteria>proteobacteria>alphaproteobacteria	Rhodobacter sp. CACIA14H1	Host attachment protein [Rhodobacter sp. CACIA14H1].
WP_036704206.1	-	127	bacteria>proteobacteria>alphaproteobacteria	Paracoccus sanguinis	hypothetical protein [Paracoccus sanguinis].
WP_036711766.1	-	127	bacteria>proteobacteria>alphaproteobacteria	Paracoccus sanguinis	hypothetical protein [Paracoccus sanguinis].
WP_036717478.1	-	127	bacteria>proteobacteria>alphaproteobacteria	Paracoccus sphaerophysae	hypothetical protein [Paracoccus sphaerophysae].
WP_039573438.1	-	127	bacteria>proteobacteria>alphaproteobacteria	Sphingopyxis fribergensis	hypothetical protein [Sphingopyxis fribergensis].
WP_045999590.1	-	127	bacteria>proteobacteria>alphaproteobacteria	Paracoccus sp. 228	hypothetical protein [Paracoccus sp. 228].
WP_048880242.1	-	127	bacteria>proteobacteria>alphaproteobacteria	Acidocella aminolytica	hypothetical protein [Acidocella aminolytica].
WP_056372043.1	-	127	bacteria>proteobacteria>alphaproteobacteria	Sphingopyxis	MULTISPECIES: hypothetical protein [Sphingopyxis].
WP_003051203.1	-	126	bacteria>proteobacteria>alphaproteobacteria	Sphingopyxis sp. MC1	hypothetical protein [Sphingopyxis sp. MC1].
WP_003617524.1	-	126	bacteria>proteobacteria>alphaproteobacteria	Komagataeibacter	MULTISPECIES: attachment protein [Komagataeibacter].
WP_010343256.1	-	126	bacteria>proteobacteria>gammaproteobacteria	Xanthomonas sacchari	host attachment protein [Xanthomonas sacchari].
WP_010508763.1	-	126	bacteria>proteobacteria>alphaproteobacteria	Komagataeibacter europaeus	attachment protein [Komagataeibacter europaeus].
WP_010514688.1	-	126	bacteria>proteobacteria>alphaproteobacteria	Komagataeibacter	MULTISPECIES: attachment protein [Komagataeibacter].
WP_014106134.1	-	126	bacteria>proteobacteria>alphaproteobacteria	Komagataeibacter medellinensis	attachment protein [Komagataeibacter medellinensis].
WP_017908761.1	-	126	bacteria>proteobacteria>gammaproteobacteria	Xanthomonas	MULTISPECIES: hypothetical protein [Xanthomonas].
WP_017914941.1	-	126	bacteria>proteobacteria>gammaproteobacteria	Xanthomonas	MULTISPECIES: hypothetical protein [Xanthomonas].
WP_025439549.1	-	126	bacteria>proteobacteria>alphaproteobacteria	Komagataeibacter xylinus	attachment protein [Komagataeibacter xylinus].
WP_025813349.1	-	126	bacteria>proteobacteria>alphaproteobacteria	Komagataeibacter kakiaceti	attachment protein [Komagataeibacter kakiaceti].
WP_033073375.1	-	126	bacteria>proteobacteria>alphaproteobacteria	Sphingopyxis sp. MWB1	hypothetical protein [Sphingopyxis sp. MWB1].
WP_034927320.1	-	126	bacteria>proteobacteria>alphaproteobacteria	Komagataeibacter rhaeticus	attachment protein [Komagataeibacter rhaeticus].
WP_039735115.1	-	126	bacteria>proteobacteria>alphaproteobacteria	Komagataeibacter intermedius	attachment protein [Komagataeibacter intermedius].
WP_039999211.1	-	126	bacteria>proteobacteria>alphaproteobacteria	Gluconacetobacter sp. SXCC-1	attachment protein [Gluconacetobacter sp. SXCC-1].
WP_048492458.1	-	126	bacteria>proteobacteria>gammaproteobacteria	Xanthomonas axonopodis	host attachment protein [Xanthomonas axonopodis].
WP_056350872.1	-	126	bacteria>proteobacteria>alphaproteobacteria	Sphingopyxis sp. Root1497	hypothetical protein [Sphingopyxis sp. Root1497].
WP_058803877.1	-	126	bacteria>proteobacteria>alphaproteobacteria	Sphingopyxis sp. H115	hypothetical protein [Sphingopyxis sp. H115].
WP_058811748.1	-	126	bacteria>proteobacteria>alphaproteobacteria	Sphingopyxis	MULTISPECIES: hypothetical protein [Sphingopyxis].
WP_061275508.1	-	126	bacteria>proteobacteria>alphaproteobacteria	Komagataeibacter xylinus	attachment protein [Komagataeibacter xylinus].
WP_062902325.1	-	126	bacteria>proteobacteria>alphaproteobacteria	Sphingopyxis terrae	hypothetical protein [Sphingopyxis terrae].
GAA07156.1	ATPR_0160	125	bacteria>proteobacteria>alphaproteobacteria	Acetobacter tropicalis NBRC 101654	hypothetical protein ATPR_0160 [Acetobacter tropicalis NBRC 101654].
WP_003465663.1	-	125	bacteria>proteobacteria>gammaproteobacteria	Xanthomonas translucens	host attachment protein [Xanthomonas translucens].
WP_009588701.1	-	125	bacteria>proteobacteria>gammaproteobacteria	Xanthomonas translucens	hypothetical protein [Xanthomonas translucens].
WP_039007975.1	-	125	bacteria>proteobacteria>gammaproteobacteria	Xanthomonas translucens	host attachment protein [Xanthomonas translucens].
WP_046978330.1	-	125	bacteria>proteobacteria>gammaproteobacteria	Xanthomonas hyacinthi	host attachment protein [Xanthomonas hyacinthi].
WP_052650073.1	-	125	bacteria>proteobacteria>alphaproteobacteria	Paracoccus sp. PAMC 22219	hypothetical protein [Paracoccus sp. PAMC 22219].
WP_052714643.1	-	125	bacteria>proteobacteria>alphaproteobacteria	Paracoccus sp. S4493	hypothetical protein [Paracoccus sp. S4493].
WP_053840693.1	-	125	bacteria>proteobacteria>gammaproteobacteria	Xanthomonas translucens	host attachment protein [Xanthomonas translucens].
KUR77833.1	AQZ50_08630	124	bacteria>proteobacteria>alphaproteobacteria	Novosphingobium sp. Fuku2-ISO-50	host cell attachment protein [Novosphingobium sp. Fuku2-ISO-50].
EGG76324.1	SXCC_02948	123	bacteria>proteobacteria>alphaproteobacteria	Gluconacetobacter sp. SXCC-1	AtsE protein [Gluconacetobacter sp. SXCC-1].
KUR80534.1	AQZ49_00230	123	bacteria>proteobacteria>alphaproteobacteria	Novosphingobium sp. FSW06-99	host cell attachment protein [Novosphingobium sp. FSW06-99].
WP_029934597.1	-	121	bacteria>proteobacteria>alphaproteobacteria	Sphingomonas sp. UNC305MFCol5.2	hypothetical protein [Sphingomonas sp. UNC305MFCol5.2].
AGA33018.1	TVNIR_1347	120	bacteria>proteobacteria>gammaproteobacteria	Thioalkalivibrio nitratireducens DSM 14787	Host attachment protein [Thioalkalivibrio nitratireducens DSM 14787].
EGO95364.1	APM_1800	119	bacteria>proteobacteria>alphaproteobacteria	Acidiphilium sp. PM	hypothetical protein APM_1800 [Acidiphilium sp. PM].
WP_028194733.1	-	119	bacteria>proteobacteria>betaproteobacteria	Burkholderiaceae	MULTISPECIES: hypothetical protein [Burkholderiaceae].
WP_038652791.1	-	119	bacteria>proteobacteria>alphaproteobacteria	Mesorhizobium huakuii	host cell attachment protein, partial [Mesorhizobium huakuii].
WP_008353653.1	-	118	bacteria>proteobacteria>betaproteobacteria	Burkholderiaceae	MULTISPECIES: hypothetical protein [Burkholderiaceae].
WP_012427347.1	-	118	bacteria>proteobacteria>betaproteobacteria	Paraburkholderia phytofirmans	hypothetical protein [Paraburkholderia phytofirmans].
WP_014251090.1	-	118	bacteria>proteobacteria>betaproteobacteria	Burkholderia sp. YI23	hypothetical protein [Burkholderia sp. YI23].
WP_016354563.1	-	118	bacteria>proteobacteria>betaproteobacteria	Burkholderia sp. RPE64	hypothetical protein [Burkholderia sp. RPE64].
WP_020071076.1	-	118	bacteria>proteobacteria>betaproteobacteria	Paraburkholderia bryophila	hypothetical protein [Paraburkholderia bryophila].
KDP85870.1	CF70_011380	118	bacteria>proteobacteria>betaproteobacteria	Cupriavidus sp. SK-3	hypothetical protein CF70_011380 [Cupriavidus sp. SK-3].
WP_027779592.1	-	118	bacteria>proteobacteria>betaproteobacteria	Paraburkholderia caledonica	hypothetical protein [Paraburkholderia caledonica].
WP_028208268.1	-	118	bacteria>proteobacteria>betaproteobacteria	Paraburkholderia nodosa	hypothetical protein [Paraburkholderia nodosa].
WP_028211014.1	-	118	bacteria>proteobacteria>betaproteobacteria	Paraburkholderia mimosarum	hypothetical protein [Paraburkholderia mimosarum].
WP_028221476.1	-	118	bacteria>proteobacteria>betaproteobacteria	Paraburkholderia oxyphila	hypothetical protein [Paraburkholderia oxyphila].
WP_028229832.1	-	118	bacteria>proteobacteria>betaproteobacteria	Paraburkholderia mimosarum	hypothetical protein [Paraburkholderia mimosarum].
WP_035501558.1	-	118	bacteria>proteobacteria>betaproteobacteria	Burkholderia jiangsuensis	hypothetical protein [Burkholderia jiangsuensis].
WP_035932906.1	-	118	bacteria>proteobacteria>betaproteobacteria	Paraburkholderia glathei	hypothetical protein [Paraburkholderia glathei].
WP_035966719.1	-	118	bacteria>proteobacteria>betaproteobacteria	Paraburkholderia grimmiae	hypothetical protein [Paraburkholderia grimmiae].
WP_040052329.1	-	118	bacteria>proteobacteria>betaproteobacteria	Burkholderia sp. MR1	hypothetical protein [Burkholderia sp. MR1].
WP_040052385.1	-	118	bacteria>proteobacteria>betaproteobacteria	Burkholderia sp. MR1	hypothetical protein [Burkholderia sp. MR1].
WP_045454673.1	-	118	bacteria>proteobacteria>betaproteobacteria	Burkholderiaceae	MULTISPECIES: hypothetical protein [Burkholderiaceae].
WP_052279646.1	-	118	bacteria>proteobacteria>betaproteobacteria	Cupriavidus sp. IDO	hypothetical protein [Cupriavidus sp. IDO].
KPD18375.1	ADM96_12930	118	bacteria>proteobacteria>betaproteobacteria	Burkholderia sp. ST111	hypothetical protein ADM96_12930 [Burkholderia sp. ST111].
WP_054921967.1	-	118	bacteria>proteobacteria>betaproteobacteria	Burkholderia sp. E7m39	hypothetical protein [Burkholderia sp. E7m39].
WP_054933599.1	-	118	bacteria>proteobacteria>betaproteobacteria	Burkholderia sp. E7m48	hypothetical protein [Burkholderia sp. E7m48].
WP_061137780.1	-	118	bacteria>proteobacteria>betaproteobacteria	Burkholderia fortuita	hypothetical protein [Burkholderia fortuita].
WP_061159887.1	-	118	bacteria>proteobacteria>betaproteobacteria	Burkholderia temeraria	hypothetical protein [Burkholderia temeraria].
WP_061167717.1	-	118	bacteria>proteobacteria>betaproteobacteria	Burkholderia hypogeia	hypothetical protein [Burkholderia hypogeia].
WP_061957403.1	-	118	bacteria>proteobacteria>betaproteobacteria	Cupriavidus pauculus	hypothetical protein [Cupriavidus pauculus].
WP_062253883.1	-	118	bacteria>proteobacteria>betaproteobacteria	Burkholderia megalochromosomata	hypothetical protein [Burkholderia megalochromosomata].
WP_062258591.1	-	118	bacteria>proteobacteria>betaproteobacteria	Burkholderia megalochromosomata	hypothetical protein [Burkholderia megalochromosomata].
WP_062604213.1	-	118	bacteria>proteobacteria>betaproteobacteria	Burkholderia calidae	hypothetical protein [Burkholderia calidae].
SAL23310.1	AWB71_01224	118	bacteria>proteobacteria>betaproteobacteria	Burkholderia peredens	Protein required for attachment to host cells [Burkholderia peredens].
SAL12658.1	AWB66_00397	118	bacteria>proteobacteria>betaproteobacteria	Paraburkholderia telluris	Protein required for attachment to host cells [Paraburkholderia telluris].
SAL13243.1	AWB73_00479	118	bacteria>proteobacteria>betaproteobacteria	Burkholderia turbans	Protein required for attachment to host cells [Burkholderia turbans].
SAL17124.1	AWB65_00731	118	bacteria>proteobacteria>betaproteobacteria	Paraburkholderia humi	Protein required for attachment to host cells [Paraburkholderia humi].
SAK74378.1	AWB83_03706	118	bacteria>proteobacteria>betaproteobacteria	Burkholderia ptereochthonis	Protein required for attachment to host cells [Burkholderia ptereochthonis].
SAK62767.1	AWB83_02547	118	bacteria>proteobacteria>betaproteobacteria	Burkholderia ptereochthonis	Protein required for attachment to host cells [Burkholderia ptereochthonis].
SAK88556.1	AWB75_06069	118	bacteria>proteobacteria>betaproteobacteria	Burkholderia catudaia	Protein required for attachment to host cells [Burkholderia catudaia].
SAK50838.1	AWB75_01460	118	bacteria>proteobacteria>betaproteobacteria	Burkholderia catudaia	Protein required for attachment to host cells [Burkholderia catudaia].
SAK40929.1	AWB82_00257	118	bacteria>proteobacteria>betaproteobacteria	Burkholderia glebae	Protein required for attachment to host cells [Burkholderia glebae].
SAK74458.1	AWB76_04783	118	bacteria>proteobacteria>betaproteobacteria	Burkholderia temeraria	Protein required for attachment to host cells [Burkholderia temeraria].
SAK53998.1	AWB80_01941	118	bacteria>proteobacteria>betaproteobacteria	Burkholderia pedi	Protein required for attachment to host cells [Burkholderia pedi].
SAK54063.1	AWB81_01193	118	bacteria>proteobacteria>betaproteobacteria	Burkholderia arationis	Protein required for attachment to host cells [Burkholderia arationis].
SAL62582.1	AWB74_03233	118	bacteria>proteobacteria>betaproteobacteria	Burkholderia arvi	Protein required for attachment to host cells [Burkholderia arvi].
SAL12577.1	AWB74_00246	118	bacteria>proteobacteria>betaproteobacteria	Burkholderia arvi	Protein required for attachment to host cells [Burkholderia arvi].
SAL77497.1	AWB68_05207	118	bacteria>proteobacteria>betaproteobacteria	Paraburkholderia choica	Protein required for attachment to host cells [Paraburkholderia choica].
SAL59661.1	AWB68_03003	118	bacteria>proteobacteria>betaproteobacteria	Paraburkholderia choica	Protein required for attachment to host cells [Paraburkholderia choica].
WP_063500751.1	-	118	bacteria>proteobacteria>betaproteobacteria	Burkholderia sp. OLGA172	hypothetical protein [Burkholderia sp. OLGA172].
WP_064269171.1	-	118	bacteria>proteobacteria>betaproteobacteria	Paraburkholderia	MULTISPECIES: hypothetical protein [Paraburkholderia].
WP_065855975.1	-	118	bacteria>proteobacteria>betaproteobacteria	Ralstonia pickettii	hypothetical protein [Ralstonia pickettii].
WP_012428033.1	-	117	bacteria>proteobacteria>betaproteobacteria	Paraburkholderia phytofirmans	hypothetical protein [Paraburkholderia phytofirmans].
KYF71305.1	BE17_12420	117	bacteria>proteobacteria>deltaproteobacteria	Sorangium cellulosum	hypothetical protein BE17_12420 [Sorangium cellulosum].
WP_035968362.1	-	116	bacteria>proteobacteria>betaproteobacteria	Paraburkholderia grimmiae	hypothetical protein [Paraburkholderia grimmiae].
KYG03813.1	BE21_04290	115	bacteria>proteobacteria>deltaproteobacteria	Sorangium cellulosum	hypothetical protein BE21_04290 [Sorangium cellulosum].
WP_010637152.1	-	111	bacteria>proteobacteria	Acidithiobacillus thiooxidans	host cell attachment-required protein [Acidithiobacillus thiooxidans].
WP_012220421.1	-	111	bacteria>proteobacteria>gammaproteobacteria	Coxiella burnetii	hypothetical protein [Coxiella burnetii].
WP_010598813.1	-	110	bacteria>proteobacteria>gammaproteobacteria	Diplorickettsia massiliensis	hypothetical protein [Diplorickettsia massiliensis].
WP_022706862.1	-	109	bacteria>proteobacteria>alphaproteobacteria	Paracoccus zeaxanthinifaciens	hypothetical protein [Paracoccus zeaxanthinifaciens].
WP_050455452.1	-	109	bacteria>proteobacteria>betaproteobacteria	Candidatus Burkholderia verschuerenii	hypothetical protein [Candidatus Burkholderia verschuerenii].
KMQ79897.1	BPMI_01349	107	bacteria>proteobacteria>betaproteobacteria	Candidatus Burkholderia pumila	hypothetical protein BPMI_01349 [Candidatus Burkholderia pumila].
EKS42904.1	HMPREF9696_00447	101	bacteria>proteobacteria>alphaproteobacteria	Afipia clevelandensis ATCC 49720	hypothetical protein HMPREF9696_00447 [Afipia clevelandensis ATCC 49720].
WP_017253027.1	-	101	bacteria>proteobacteria>gammaproteobacteria	Coxiella burnetii	hypothetical protein [Coxiella burnetii].
WP_061301481.1	-	101	bacteria>proteobacteria>gammaproteobacteria	Coxiella burnetii	hypothetical protein [Coxiella burnetii].
WP_062917429.1	-	100	bacteria>proteobacteria>betaproteobacteria	Paraburkholderia caribensis	hypothetical protein [Paraburkholderia caribensis].
AMV45894.1	ATN79_28535	98	bacteria>proteobacteria>betaproteobacteria	Paraburkholderia caribensis	hypothetical protein ATN79_28535 [Paraburkholderia caribensis].
KPP91286.1	HLUCCA08_08580	94	bacteria>proteobacteria>alphaproteobacteria	Rhodobacteraceae bacterium HLUCCA08	hypothetical protein HLUCCA08_08580 [Rhodobacteraceae bacterium HLUCCA08].
ACM34587.1	Dtpsy_3154	90	bacteria>proteobacteria>betaproteobacteria	Acidovorax ebreus TPSY	conserved hypothetical protein [Acidovorax ebreus TPSY].
ESH93179.1	B551_0221080	90	bacteria>proteobacteria>betaproteobacteria	Cupriavidus sp. HPC(L)	hypothetical protein B551_0221080, partial [Cupriavidus sp. HPC(L)].
ABQ39599.1	BBta_7762	83	bacteria>proteobacteria>alphaproteobacteria	Bradyrhizobium sp. BTAi1	hypothetical protein BBta_7762 [Bradyrhizobium sp. BTAi1].
BAR62125.1	NK6_8981	82	bacteria>proteobacteria>alphaproteobacteria	Bradyrhizobium diazoefficiens	hypothetical protein NK6_8981 [Bradyrhizobium diazoefficiens].

2. family 2

##;baeRF1 family 2 follows the standard core architecture: codon_recognition_domain+baeRF1+Pelota.
##;The Pelota domain typically contains the 2 beta-strand insert characteristic of this clade, although
##;a few contain the zinc finger insert observed in eukaryotes and some archaeal members of the
##;classical aeRF1 family.

a. 2 beta-strand PELOTA insert

WP_013016396.1	-	536	bacteria>actinobacteria	Stackebrandtia nassauensis	hypothetical protein [Stackebrandtia nassauensis].

WP_051162328.1	-	481	bacteria>actinobacteria	Nocardia brevicatena	hypothetical protein [Nocardia brevicatena].
WP_051264587.1	-	410	bacteria>actinobacteria	Streptomyces sp. TAA204	hypothetical protein [Streptomyces sp. TAA204].
WP_045864720.1	-	408	bacteria>actinobacteria	Streptomyces sp. WMMB 714	hypothetical protein [Streptomyces sp. WMMB 714].
WP_051345220.1	-	408	bacteria>actinobacteria	Streptomyces sp. TAA486	hypothetical protein [Streptomyces sp. TAA486].
WP_055487092.1	-	408	bacteria>actinobacteria	Streptomyces sp. WMMB 322	hypothetical protein [Streptomyces sp. WMMB 322].
WP_059141935.1	-	399	bacteria>actinobacteria	Streptomyces violaceusniger	hypothetical protein [Streptomyces violaceusniger].
WP_037577142.1	-	398	bacteria>actinobacteria	Streptacidiphilus oryzae	hypothetical protein [Streptacidiphilus oryzae].
WP_030832545.1	-	394	bacteria>actinobacteria	Streptomyces hygroscopicus	hypothetical protein [Streptomyces hygroscopicus].
WP_060944210.1	-	394	bacteria>actinobacteria	Streptomyces	MULTISPECIES: hypothetical protein [Streptomyces].
WP_060949809.1	-	394	bacteria>actinobacteria	Streptomyces hygroscopicus	hypothetical protein [Streptomyces hygroscopicus].
WP_062008426.1	-	394	bacteria>actinobacteria	Streptomyces sporocinereus	hypothetical protein [Streptomyces sporocinereus].
WP_062975167.1	-	394	bacteria>actinobacteria	Nocardia flavorosea	hypothetical protein [Nocardia flavorosea].
WP_064456946.1	-	394	bacteria>actinobacteria	Streptomyces sp. NBRC 109436	hypothetical protein [Streptomyces sp. NBRC 109436].
WP_055549179.1	-	390	bacteria>actinobacteria	Streptomyces sp. NBRC 110028	hypothetical protein [Streptomyces sp. NBRC 110028].
WP_009714144.1	-	389	bacteria>actinobacteria	Streptomyces himastatinicus	hypothetical protein [Streptomyces himastatinicus].
AGP54223.1	M271_13145	389	bacteria>actinobacteria	Streptomyces rapamycinicus NRRL 5491	hypothetical protein M271_13145 [Streptomyces rapamycinicus NRRL 5491].
WP_037962495.1	-	389	bacteria>actinobacteria	Streptomyces sp. PRh5	hypothetical protein [Streptomyces sp. PRh5].
WP_044569024.1	-	389	bacteria>actinobacteria	Streptomyces iranensis	hypothetical protein [Streptomyces iranensis].
WP_062781647.1	-	389	bacteria>actinobacteria	Streptomyces sp. NRRL S-1521	hypothetical protein [Streptomyces sp. NRRL S-1521].
WP_063730713.1	-	389	bacteria>actinobacteria	Streptomyces sp. RTd22	hypothetical protein [Streptomyces sp. RTd22].
WP_019710939.1	-	388	bacteria>actinobacteria	Streptomyces xinghaiensis	hypothetical protein [Streptomyces xinghaiensis].
WP_030547396.1	-	388	bacteria>actinobacteria	Streptomyces albus	hypothetical protein [Streptomyces albus].
WP_043463966.1	-	388	bacteria>actinobacteria	Streptomyces fradiae	hypothetical protein [Streptomyces fradiae].
WP_030492203.1	-	382	bacteria>actinobacteria	Microtetraspora glauca	hypothetical protein [Microtetraspora glauca].
WP_017976094.1	-	379	bacteria>actinobacteria	Actinopolyspora halophila	hypothetical protein [Actinopolyspora halophila].
WP_019853579.1	-	379	bacteria>actinobacteria	Actinopolyspora mortivallis	hypothetical protein [Actinopolyspora mortivallis].
WP_055516703.1	-	379	bacteria>actinobacteria	Streptomyces aurantiacus	hypothetical protein [Streptomyces aurantiacus].
WP_014375824.1	-	376	bacteria>actinobacteria	Blastococcus saxobsidens	hypothetical protein [Blastococcus saxobsidens].
WP_051757920.1	-	376	bacteria>actinobacteria	Prauserella rugosa	hypothetical protein [Prauserella rugosa].
WP_003972465.1	-	373	bacteria>actinobacteria	Streptomyces	MULTISPECIES: hypothetical protein [Streptomyces].
WP_007445588.1	-	373	bacteria>actinobacteria	Streptomyces	MULTISPECIES: hypothetical protein [Streptomyces].
WP_011030976.1	-	373	bacteria>actinobacteria	Streptomyces coelicolor	hypothetical protein [Streptomyces coelicolor].
WP_029932330.1	-	373	bacteria>actinobacteria	Nocardia otitidiscaviarum	hypothetical protein [Nocardia otitidiscaviarum].
WP_030178428.1	-	373	bacteria>actinobacteria	Streptomyces violaceorubidus	hypothetical protein [Streptomyces violaceorubidus].
WP_031037526.1	-	373	bacteria>actinobacteria	Streptomyces olivaceus	hypothetical protein [Streptomyces olivaceus].
WP_037769489.1	-	373	bacteria>actinobacteria	Streptomyces sp. FXJ7.023	hypothetical protein [Streptomyces sp. FXJ7.023].
WP_039813980.1	-	373	bacteria>actinobacteria	Nocardia otitidiscaviarum	hypothetical protein [Nocardia otitidiscaviarum].
WP_046470858.1	-	373	bacteria>actinobacteria	Allosalinactinospora lopnorensis	hypothetical protein [Allosalinactinospora lopnorensis].
WP_052840526.1	-	373	bacteria>actinobacteria	Streptomyces aureofaciens	hypothetical protein [Streptomyces aureofaciens].
WP_055419328.1	-	373	bacteria>actinobacteria	Streptomyces pactum	hypothetical protein [Streptomyces pactum].
WP_059254271.1	-	373	bacteria>actinobacteria	Streptomyces reticuli	hypothetical protein [Streptomyces reticuli].
WP_059300105.1	-	373	bacteria>actinobacteria	Streptomyces canus	hypothetical protein [Streptomyces canus].
WP_061441433.1	-	373	bacteria>actinobacteria	Streptomyces sp. CCM_MD2014	hypothetical protein [Streptomyces sp. CCM_MD2014].
WP_006142937.1	-	372	bacteria>actinobacteria	Streptomyces griseoaurantiacus	hypothetical protein [Streptomyces griseoaurantiacus].
WP_014676025.1	-	372	bacteria>actinobacteria	Streptomyces	MULTISPECIES: hypothetical protein [Streptomyces].
WP_018382930.1	-	372	bacteria>actinobacteria	Streptomyces vitaminophilus	hypothetical protein [Streptomyces vitaminophilus].
WP_030605827.1	-	372	bacteria>actinobacteria	Streptomyces achromogenes	hypothetical protein [Streptomyces achromogenes].
WP_030643580.1	-	372	bacteria>actinobacteria	Streptomyces	MULTISPECIES: hypothetical protein [Streptomyces].
WP_030789226.1	-	372	bacteria>actinobacteria	Streptomyces lavenduligriseus	hypothetical protein [Streptomyces lavenduligriseus].
WP_030983961.1	-	372	bacteria>actinobacteria	Streptomyces sp. NRRL WC-3744	hypothetical protein [Streptomyces sp. NRRL WC-3744].
WP_031025532.1	-	372	bacteria>actinobacteria	Streptomyces sp. NRRL WC-3725	hypothetical protein [Streptomyces sp. NRRL WC-3725].
WP_031096113.1	-	372	bacteria>actinobacteria	Streptomyces	MULTISPECIES: hypothetical protein [Streptomyces].
KMS82614.1	ACZ91_58195	372	bacteria>actinobacteria	Streptomyces regensis	hypothetical protein ACZ91_58195 [Streptomyces regensis].
WP_053211348.1	-	372	bacteria>actinobacteria	Streptomyces antibioticus	hypothetical protein [Streptomyces antibioticus].
WP_020133308.1	-	371	bacteria>actinobacteria	Streptomyces sp. 351MFTsu5.1	hypothetical protein [Streptomyces sp. 351MFTsu5.1].
WP_028424580.1	-	371	bacteria>actinobacteria	Streptomyces sp. GXT6	hypothetical protein [Streptomyces sp. GXT6].
WP_030040743.1	-	371	bacteria>actinobacteria	Streptomyces resistomycificus	hypothetical protein [Streptomyces resistomycificus].
WP_055619336.1	-	371	bacteria>actinobacteria	Streptomyces sp. JHA19	hypothetical protein [Streptomyces sp. JHA19].
WP_004922801.1	-	370	bacteria>actinobacteria	Streptomyces griseoflavus	hypothetical protein [Streptomyces griseoflavus].
WP_024883836.1	-	370	bacteria>actinobacteria	Streptomyces sp. CNH189	hypothetical protein [Streptomyces sp. CNH189].
WP_030819107.1	-	370	bacteria>actinobacteria	Streptomyces	MULTISPECIES: hypothetical protein [Streptomyces].
WP_030856361.1	-	370	bacteria>actinobacteria	Streptomyces sp. NRRL S-37	hypothetical protein [Streptomyces sp. NRRL S-37].
WP_053635919.1	-	370	bacteria>actinobacteria	Streptomyces sp. XY152	hypothetical protein [Streptomyces sp. XY152].
WP_063036035.1	-	370	bacteria>actinobacteria	Nocardia grenadensis	hypothetical protein [Nocardia grenadensis].
WP_063059125.1	-	370	bacteria>actinobacteria	Nocardia sienata	hypothetical protein [Nocardia sienata].
WP_037926662.1	-	369	bacteria>actinobacteria	Streptomyces toyocaensis	hypothetical protein [Streptomyces toyocaensis].
WP_051313792.1	-	369	bacteria>actinobacteria	Actinoalloteichus cyanogriseus	hypothetical protein [Actinoalloteichus cyanogriseus].
WP_051712786.1	-	369	bacteria>actinobacteria	Actinoalloteichus cyanogriseus	hypothetical protein [Actinoalloteichus cyanogriseus].
OAH12060.1	STSP_46350	369	bacteria>actinobacteria	Streptomyces sp. G25(2015)	hypothetical protein STSP_46350 [Streptomyces sp. G25(2015)].
KWW98342.1	TH66_23025	368	bacteria>actinobacteria	Streptomyces thermoautotrophicus	hypothetical protein TH66_23025 [Streptomyces thermoautotrophicus].
WP_007385873.1	-	367	bacteria>actinobacteria	Streptomyces sviceus	hypothetical protein [Streptomyces sviceus].
WP_037706483.1	-	367	bacteria>actinobacteria	Streptomyces mirabilis	hypothetical protein [Streptomyces mirabilis].
WP_030246158.1	-	366	bacteria>actinobacteria	Streptomyces sp. NRRL S-455	hypothetical protein [Streptomyces sp. NRRL S-455].
WP_030897195.1	-	366	bacteria>actinobacteria	Streptomyces	MULTISPECIES: hypothetical protein [Streptomyces].
WP_033309162.1	-	366	bacteria>actinobacteria	Streptomyces iakyrus	hypothetical protein [Streptomyces iakyrus].
WP_062930125.1	-	366	bacteria>actinobacteria	Streptomyces sp. S10(2016)	hypothetical protein [Streptomyces sp. S10(2016)].
WP_030428514.1	-	365	bacteria>actinobacteria	Allokutzneria albata	hypothetical protein [Allokutzneria albata].
WP_051743126.1	-	363	bacteria>actinobacteria	Nocardia carnea	hypothetical protein [Nocardia carnea].
WP_063916322.1	-	363	bacteria>actinobacteria	Nocardia jinanensis	hypothetical protein [Nocardia jinanensis].
WP_060591334.1	-	361	bacteria>actinobacteria	Nocardia farcinica	hypothetical protein [Nocardia farcinica].
WP_030519207.1	-	360	bacteria>actinobacteria	Nocardia rhamnosiphila	hypothetical protein [Nocardia rhamnosiphila].
WP_063814716.1	-	360	bacteria>actinobacteria	Nocardia fusca	hypothetical protein [Nocardia fusca].
WP_036536283.1	-	358	bacteria>actinobacteria	Nocardia cyriacigeorgica	hypothetical protein [Nocardia cyriacigeorgica].
WP_014351326.1	-	357	bacteria>actinobacteria	Nocardia cyriacigeorgica	hypothetical protein [Nocardia cyriacigeorgica].
WP_030509634.1	-	357	bacteria>actinobacteria	Microbispora rosea	hypothetical protein [Microbispora rosea].
WP_036510874.1	-	356	bacteria>actinobacteria	Nocardia aobensis	hypothetical protein [Nocardia aobensis].
WP_039782504.1	-	356	bacteria>actinobacteria	Nocardia cerradoensis	hypothetical protein [Nocardia cerradoensis].
WP_040718765.1	-	356	bacteria>actinobacteria	Nocardia veterana	hypothetical protein [Nocardia veterana].
WP_055477507.1	-	356	bacteria>actinobacteria	Microbispora	MULTISPECIES: hypothetical protein [Microbispora].
WP_063064328.1	-	356	bacteria>actinobacteria	Nocardia violaceofusca	hypothetical protein [Nocardia violaceofusca].
WP_063910823.1	-	356	bacteria>actinobacteria	Corynebacteriales	MULTISPECIES: hypothetical protein [Corynebacteriales].
WP_063917467.1	-	356	bacteria>actinobacteria	Nocardia africana	hypothetical protein [Nocardia africana].
WP_064910742.1	-	356	bacteria>actinobacteria	Nocardia sp. 852002-20019_SCH5090214	hypothetical protein [Nocardia sp. 852002-20019_SCH5090214].
WP_036331876.1	-	355	bacteria>actinobacteria	Microbispora sp. ATCC PTA-5024	hypothetical protein [Microbispora sp. ATCC PTA-5024].
CRY75535.1	ERS450000_01385	355	bacteria>actinobacteria	Nocardia farcinica	Uncharacterised protein [Nocardia farcinica].
WP_061260549.1	-	355	bacteria>actinobacteria	Microtetraspora glauca	hypothetical protein [Microtetraspora glauca].
WP_027344335.1	-	354	bacteria>actinobacteria	Hamadaea tsunoensis	hypothetical protein [Hamadaea tsunoensis].
WP_043640244.1	-	339	bacteria>actinobacteria	Nonomuraea candida	hypothetical protein [Nonomuraea candida].
WP_020542089.1	-	336	bacteria>actinobacteria	Nonomuraea coxensis	hypothetical protein [Nonomuraea coxensis].
WP_049570051.1	-	336	bacteria>actinobacteria	Nonomuraea sp. SBT364	hypothetical protein [Nonomuraea sp. SBT364].
WP_052313843.1	-	336	bacteria>actinobacteria	Nocardia thailandica	hypothetical protein [Nocardia thailandica].
WP_055510237.1	-	336	bacteria>actinobacteria	Nonomuraea sp. NBRC 110462	hypothetical protein [Nonomuraea sp. NBRC 110462].
WP_051164643.1	-	295	bacteria>actinobacteria	Nocardia testacea	hypothetical protein [Nocardia testacea].
WP_063024679.1	-	170	bacteria>actinobacteria	Nocardia elegans	hypothetical protein [Nocardia elegans].

WP_012949449.1	-	377	bacteria>actinobacteria	Geodermatophilus obscurus	hypothetical protein [Geodermatophilus obscurus].
WP_029433853.1	-	376	bacteria>actinobacteria	Blastococcus sp. URHD0036	hypothetical protein [Blastococcus sp. URHD0036].
WP_014739922.1	-	375	bacteria>actinobacteria	Modestobacter marinus	hypothetical protein [Modestobacter marinus].
WP_026845329.1	-	375	bacteria>actinobacteria	Geodermatophilaceae bacterium URHA0031	hypothetical protein [Geodermatophilaceae bacterium URHA0031].
WP_036334795.1	-	375	bacteria>actinobacteria	Modestobacter caceresii	hypothetical protein [Modestobacter caceresii].
WP_026854681.1	-	374	bacteria>actinobacteria	Geodermatophilaceae bacterium URHB0062	hypothetical protein [Geodermatophilaceae bacterium URHB0062].
WP_029339571.1	-	374	bacteria>actinobacteria	Geodermatophilaceae bacterium URHB0048	hypothetical protein [Geodermatophilaceae bacterium URHB0048].
WP_040336803.1	-	374	bacteria>actinobacteria	Candidatus Blastococcus massiliensis	hypothetical protein [Candidatus Blastococcus massiliensis].
WP_028281209.1	-	380	bacteria>actinobacteria	Arthrobacter sp. H5	hypothetical protein [Arthrobacter sp. H5].
WP_056297828.1	-	378	bacteria>actinobacteria	Modestobacter sp. Leaf380	hypothetical protein [Modestobacter sp. Leaf380].
CKP28294.1	ERS024227_03657	554	bacteria>actinobacteria	Mycobacterium tuberculosis	Hypothetical protein ERS024227_03657 [Mycobacterium tuberculosis].
WP_003902322.1	-	374	bacteria>actinobacteria	Mycobacterium tuberculosis	hypothetical protein [Mycobacterium tuberculosis].
WP_014001364.1	-	374	bacteria>actinobacteria	Mycobacterium canettii	hypothetical protein [Mycobacterium canettii].
WP_015288338.1	-	374	bacteria>actinobacteria	Mycobacterium canettii	hypothetical protein [Mycobacterium canettii].
WP_015290839.1	-	374	bacteria>actinobacteria	Mycobacterium canettii	hypothetical protein [Mycobacterium canettii].
WP_015293826.1	-	374	bacteria>actinobacteria	Mycobacterium canettii	hypothetical protein [Mycobacterium canettii].
WP_015303544.1	-	374	bacteria>actinobacteria	Mycobacterium canettii	hypothetical protein [Mycobacterium canettii].
WP_015630479.1	-	374	bacteria>actinobacteria	Mycobacterium tuberculosis	hypothetical protein [Mycobacterium tuberculosis].
WP_023349797.1	-	374	bacteria>actinobacteria	Mycobacterium bovis	hypothetical protein [Mycobacterium bovis].
WP_024456307.1	-	374	bacteria>actinobacteria	Mycobacterium tuberculosis complex	MULTISPECIES: hypothetical protein [Mycobacterium tuberculosis complex].
AIH55592.1	IQ41_10990	374	bacteria>actinobacteria	Mycobacterium tuberculosis	hypothetical protein IQ41_10990 [Mycobacterium tuberculosis].
AIH67756.1	IU17_11340	374	bacteria>actinobacteria	Mycobacterium tuberculosis	hypothetical protein IU17_11340 [Mycobacterium tuberculosis].
AIH88876.1	IU19_11360	374	bacteria>actinobacteria	Mycobacterium tuberculosis	hypothetical protein IU19_11360 [Mycobacterium tuberculosis].
WP_031580436.1	-	374	bacteria>actinobacteria	Mycobacterium tuberculosis	hypothetical protein [Mycobacterium tuberculosis].
WP_031647640.1	-	374	bacteria>actinobacteria	Mycobacterium tuberculosis	hypothetical protein [Mycobacterium tuberculosis].
WP_031653302.1	-	374	bacteria>actinobacteria	Mycobacterium tuberculosis	hypothetical protein [Mycobacterium tuberculosis].
WP_031678140.1	-	374	bacteria>actinobacteria	Mycobacterium tuberculosis	hypothetical protein [Mycobacterium tuberculosis].
WP_031716273.1	-	374	bacteria>actinobacteria	Mycobacterium tuberculosis	hypothetical protein [Mycobacterium tuberculosis].
WP_031724141.1	-	374	bacteria>actinobacteria	Mycobacterium tuberculosis	hypothetical protein [Mycobacterium tuberculosis].
WP_031728329.1	-	374	bacteria>actinobacteria	Mycobacterium tuberculosis	hypothetical protein [Mycobacterium tuberculosis].
WP_031738268.1	-	374	bacteria>actinobacteria	Mycobacterium tuberculosis	hypothetical protein [Mycobacterium tuberculosis].
WP_031739651.1	-	374	bacteria>actinobacteria	Mycobacterium tuberculosis	hypothetical protein [Mycobacterium tuberculosis].
WP_042915426.1	-	374	bacteria>actinobacteria	Mycobacterium canettii	hypothetical protein [Mycobacterium canettii].
WP_044096963.1	-	374	bacteria>actinobacteria	Mycobacterium canettii	hypothetical protein [Mycobacterium canettii].
WP_050186619.1	-	374	bacteria>actinobacteria	Mycobacterium tuberculosis	hypothetical protein [Mycobacterium tuberculosis].
WP_052636842.1	-	374	bacteria>actinobacteria	Mycobacterium tuberculosis	hypothetical protein [Mycobacterium tuberculosis].
WP_052660471.1	-	374	bacteria>actinobacteria	Mycobacterium tuberculosis	hypothetical protein [Mycobacterium tuberculosis].
WP_055351533.1	-	374	bacteria>actinobacteria	Mycobacterium tuberculosis	hypothetical protein [Mycobacterium tuberculosis].
WP_055365290.1	-	374	bacteria>actinobacteria	Mycobacterium tuberculosis	hypothetical protein [Mycobacterium tuberculosis].
WP_055371722.1	-	374	bacteria>actinobacteria	Mycobacterium tuberculosis	hypothetical protein [Mycobacterium tuberculosis].
WP_055380339.1	-	374	bacteria>actinobacteria	Mycobacterium tuberculosis	hypothetical protein [Mycobacterium tuberculosis].
WP_055381239.1	-	374	bacteria>actinobacteria	Mycobacterium tuberculosis	hypothetical protein [Mycobacterium tuberculosis].
WP_057127982.1	-	374	bacteria>actinobacteria	Mycobacterium tuberculosis	hypothetical protein [Mycobacterium tuberculosis].
WP_057131291.1	-	374	bacteria>actinobacteria	Mycobacterium tuberculosis	hypothetical protein [Mycobacterium tuberculosis].
WP_057132228.1	-	374	bacteria>actinobacteria	Mycobacterium tuberculosis	hypothetical protein [Mycobacterium tuberculosis].
WP_057132722.1	-	374	bacteria>actinobacteria	Mycobacterium tuberculosis	hypothetical protein [Mycobacterium tuberculosis].
WP_057150739.1	-	374	bacteria>actinobacteria	Mycobacterium tuberculosis	hypothetical protein [Mycobacterium tuberculosis].
WP_057171501.1	-	374	bacteria>actinobacteria	Mycobacterium tuberculosis	hypothetical protein [Mycobacterium tuberculosis].
WP_057288076.1	-	374	bacteria>actinobacteria	Mycobacterium tuberculosis	hypothetical protein [Mycobacterium tuberculosis].
WP_057308215.1	-	374	bacteria>actinobacteria	Mycobacterium tuberculosis	hypothetical protein [Mycobacterium tuberculosis].
WP_057319550.1	-	374	bacteria>actinobacteria	Mycobacterium tuberculosis	hypothetical protein [Mycobacterium tuberculosis].
WP_057320809.1	-	374	bacteria>actinobacteria	Mycobacterium tuberculosis	hypothetical protein [Mycobacterium tuberculosis].
WP_057332091.1	-	374	bacteria>actinobacteria	Mycobacterium tuberculosis	hypothetical protein [Mycobacterium tuberculosis].
WP_057333577.1	-	374	bacteria>actinobacteria	Mycobacterium tuberculosis	hypothetical protein [Mycobacterium tuberculosis].
WP_057336345.1	-	374	bacteria>actinobacteria	Mycobacterium tuberculosis	hypothetical protein [Mycobacterium tuberculosis].
WP_057338196.1	-	374	bacteria>actinobacteria	Mycobacterium tuberculosis	hypothetical protein [Mycobacterium tuberculosis].
WP_057341544.1	-	374	bacteria>actinobacteria	Mycobacterium tuberculosis	hypothetical protein [Mycobacterium tuberculosis].
WP_057345631.1	-	374	bacteria>actinobacteria	Mycobacterium tuberculosis	hypothetical protein [Mycobacterium tuberculosis].
WP_057346105.1	-	374	bacteria>actinobacteria	Mycobacterium tuberculosis	hypothetical protein [Mycobacterium tuberculosis].
WP_057347818.1	-	374	bacteria>actinobacteria	Mycobacterium tuberculosis	hypothetical protein [Mycobacterium tuberculosis].
WP_057351709.1	-	374	bacteria>actinobacteria	Mycobacterium tuberculosis	hypothetical protein [Mycobacterium tuberculosis].
WP_057355996.1	-	374	bacteria>actinobacteria	Mycobacterium tuberculosis	hypothetical protein [Mycobacterium tuberculosis].
WP_057362328.1	-	374	bacteria>actinobacteria	Mycobacterium tuberculosis	hypothetical protein [Mycobacterium tuberculosis].
WP_057370645.1	-	374	bacteria>actinobacteria	Mycobacterium tuberculosis	hypothetical protein [Mycobacterium tuberculosis].
WP_057372831.1	-	374	bacteria>actinobacteria	Mycobacterium tuberculosis	hypothetical protein [Mycobacterium tuberculosis].
WP_031724235.1	-	373	bacteria>actinobacteria	Mycobacterium tuberculosis	hypothetical protein [Mycobacterium tuberculosis].
CKR68095.1	ERS027656_01867	313	bacteria>actinobacteria	Mycobacterium tuberculosis	Uncharacterised protein [Mycobacterium tuberculosis].
OBH27566.1	A5692_24485	374	bacteria>actinobacteria	Mycobacterium sp. E342	hypothetical protein A5692_24485 [Mycobacterium sp. E342].
WP_062053734.1	-	389	bacteria>bacteroidetes	Sediminicola sp. YIK13	hypothetical protein [Sediminicola sp. YIK13].
WP_048419580.1	-	387	bacteria>actinobacteria	Mycobacterium chubuense	hypothetical protein [Mycobacterium chubuense].
WP_055580094.1	-	377	bacteria>actinobacteria	Mycobacterium gordonae	hypothetical protein [Mycobacterium gordonae].
WP_065134723.1	-	377	bacteria>actinobacteria	Mycobacterium gordonae	hypothetical protein [Mycobacterium gordonae].
WP_048471829.1	-	375	bacteria>actinobacteria	Mycobacterium chlorophenolicum	hypothetical protein [Mycobacterium chlorophenolicum].
ABL05476.1	MUL_3275	374	bacteria>actinobacteria	Mycobacterium ulcerans Agy99	conserved hypothetical protein [Mycobacterium ulcerans Agy99].
WP_012393845.1	-	374	bacteria>actinobacteria	Mycobacterium marinum	hypothetical protein [Mycobacterium marinum].
WP_015355909.1	-	374	bacteria>actinobacteria	Mycobacterium liflandii	hypothetical protein [Mycobacterium liflandii].
WP_020724867.1	-	374	bacteria>actinobacteria	Mycobacterium marinum	hypothetical protein [Mycobacterium marinum].
WP_020787936.1	-	374	bacteria>actinobacteria	Mycobacterium sp. 012931	hypothetical protein [Mycobacterium sp. 012931].
WP_023372808.1	-	374	bacteria>actinobacteria	Mycobacterium kansasii	hypothetical protein [Mycobacterium kansasii].
WP_036412644.1	-	374	bacteria>actinobacteria	Mycobacterium gastri	hypothetical protein [Mycobacterium gastri].
WP_036455504.1	-	374	bacteria>actinobacteria	Mycobacterium marinum	hypothetical protein [Mycobacterium marinum].
WP_038579398.1	-	374	bacteria>actinobacteria	Mycobacterium marinum	hypothetical protein [Mycobacterium marinum].
CQD12135.1	BN1232_02302	374	bacteria>actinobacteria	Mycobacterium lentiflavum	hypothetical protein BN1232_02302 [Mycobacterium lentiflavum].
CQD22053.1	BN000_05463	374	bacteria>actinobacteria	Mycobacterium europaeum	hypothetical protein BN000_05463 [Mycobacterium europaeum].
WP_062654995.1	-	374	bacteria>actinobacteria	Mycobacterium canariasense	hypothetical protein [Mycobacterium canariasense].
OBH02144.1	A5696_11295	374	bacteria>actinobacteria	Mycobacterium sp. E2699	hypothetical protein A5696_11295 [Mycobacterium sp. E2699].
OBH17568.1	A9X04_09835	374	bacteria>actinobacteria	Mycobacterium sp. E3247	hypothetical protein A9X04_09835 [Mycobacterium sp. E3247].
OBI15982.1	A5713_22925	374	bacteria>actinobacteria	Mycobacterium sp. E2497	hypothetical protein A5713_22925 [Mycobacterium sp. E2497].
BAV42154.1	SHTP_3094	374	bacteria>actinobacteria	Mycobacterium ulcerans subsp. shinshuense	hypothetical protein SHTP_3094 [Mycobacterium ulcerans subsp. shinshuense].
OBH06904.1	A5695_03910	373	bacteria>actinobacteria	Mycobacterium sp. E1747	hypothetical protein A5695_03910 [Mycobacterium sp. E1747].
OBH49875.1	A5688_22365	372	bacteria>actinobacteria	Mycobacterium sp. E3121	hypothetical protein A5688_22365 [Mycobacterium sp. E3121].
OBH50845.1	A5687_12620	372	bacteria>actinobacteria	Mycobacterium sp. E2660	hypothetical protein A5687_12620 [Mycobacterium sp. E2660].
WP_036403360.1	-	371	bacteria>actinobacteria	Mycobacterium cosmeticum	hypothetical protein [Mycobacterium cosmeticum].
WP_055589133.1	-	365	bacteria>actinobacteria	Streptomyces griseoplanus	hypothetical protein [Streptomyces griseoplanus].
EPQ79703.1	MMEU_0227	356	bacteria>actinobacteria	Mycobacterium marinum str. Europe	hypothetical protein MMEU_0227 [Mycobacterium marinum str. Europe].
WP_011778667.1	-	368	bacteria>actinobacteria	Mycobacterium vanbaalenii	hypothetical protein [Mycobacterium vanbaalenii].
WP_036369999.1	-	368	bacteria>actinobacteria	Mycobacterium austroafricanum	hypothetical protein [Mycobacterium austroafricanum].
WP_023544087.1	-	367	bacteria>actinobacteria	Streptomyces roseochromogenus	hypothetical protein [Streptomyces roseochromogenus].
WP_061917113.1	-	367	bacteria>actinobacteria	Streptomyces bungoensis	hypothetical protein [Streptomyces bungoensis].
WP_041857704.1	-	399	bacteria>acidobacteria	Candidatus Solibacter usitatus	hypothetical protein [Candidatus Solibacter usitatus].
ANZ14776.1	SNOUR_07285	530	bacteria>actinobacteria	Streptomyces noursei ATCC 11455	hypothetical protein SNOUR_07285 [Streptomyces noursei ATCC 11455].
WP_015662197.1	-	450	bacteria>actinobacteria	Streptomyces davawensis	hypothetical protein [Streptomyces davawensis].
WP_030906443.1	-	432	bacteria>actinobacteria	Streptomyces sp. NRRL F-5126	hypothetical protein [Streptomyces sp. NRRL F-5126].
WP_028425368.1	-	430	bacteria>actinobacteria	Streptomyces sp. GXT6	hypothetical protein [Streptomyces sp. GXT6].
KUL38490.1	ADL22_18420	420	bacteria>actinobacteria	Streptomyces sp. NRRL F-4489	hypothetical protein ADL22_18420 [Streptomyces sp. NRRL F-4489].
ANY09274.1	AFB00_26915	419	bacteria>actinobacteria	Pseudonocardia sp. HH130630-07	hypothetical protein AFB00_26915 [Pseudonocardia sp. HH130630-07].
WP_012877897.1	-	418	bacteria>actinobacteria	Xylanimonas cellulosilytica	hypothetical protein [Xylanimonas cellulosilytica].
WP_013838265.1	-	416	bacteria>actinobacteria	Isoptericola variabilis	hypothetical protein [Isoptericola variabilis].
ANC30694.1	I598_1125	415	bacteria>actinobacteria	Isoptericola dokdonensis DS-3	hypothetical protein I598_1125 [Isoptericola dokdonensis DS-3].
WP_052425911.1	-	413	bacteria>actinobacteria	Cellulomonas carbonis	hypothetical protein [Cellulomonas carbonis].
WP_040255293.1	-	411	bacteria>actinobacteria	Streptomyces albus	hypothetical protein [Streptomyces albus].
WP_034244135.1	-	410	bacteria>actinobacteria	Actinotalea fermentans	hypothetical protein [Actinotalea fermentans].
WP_043496939.1	-	409	bacteria>actinobacteria	Georgenia sp. SUBG003	hypothetical protein [Georgenia sp. SUBG003].
WP_018955611.1	-	406	bacteria>actinobacteria	Streptomyces sp. CNB091	hypothetical protein [Streptomyces sp. CNB091].
WP_030570594.1	-	403	bacteria>actinobacteria	Streptomyces cyaneofuscatus	hypothetical protein [Streptomyces cyaneofuscatus].
WP_027762145.1	-	402	bacteria>actinobacteria	Streptomyces sp. CNS606	hypothetical protein [Streptomyces sp. CNS606].
WP_024288884.1	-	401	bacteria>actinobacteria	Cellulomonas sp. KRMCY2	hypothetical protein [Cellulomonas sp. KRMCY2].
GAT06560.1	RMCFA_6671	401	bacteria>actinobacteria	Mycobacterium fortuitum subsp. acetamidolyticum	uncharacterized protein RMCFA_6671 [Mycobacterium fortuitum subsp. acetamidolyticum].
WP_013116629.1	-	400	bacteria>actinobacteria	Cellulomonas flavigena	hypothetical protein [Cellulomonas flavigena].
WP_013884185.1	-	400	bacteria>actinobacteria	Cellulomonas gilvus	hypothetical protein [Cellulomonas gilvus].
WP_034624795.1	-	400	bacteria>actinobacteria	Cellulomonas cellasea	hypothetical protein [Cellulomonas cellasea].
WP_051717838.1	-	400	bacteria>actinobacteria	Streptomyces megasporus	hypothetical protein [Streptomyces megasporus].
WP_013770599.1	-	399	bacteria>actinobacteria	Cellulomonas fimi	hypothetical protein [Cellulomonas fimi].
WP_021482740.1	-	399	bacteria>firmicutes	Bacillus sp. EGD-AK10	hypothetical protein [Bacillus sp. EGD-AK10].
WP_024840477.1	-	399	bacteria>actinobacteria	Cellulosimicrobium	MULTISPECIES: hypothetical protein [Cellulosimicrobium].
WP_030150971.1	-	399	bacteria>actinobacteria	Oerskovia turbata	hypothetical protein [Oerskovia turbata].
WP_034224180.1	-	399	bacteria>actinobacteria	Actinotalea ferrariae	hypothetical protein [Actinotalea ferrariae].
WP_034655925.1	-	399	bacteria>actinobacteria	Cellulosimicrobium	MULTISPECIES: hypothetical protein [Cellulosimicrobium].
WP_036951667.1	-	399	bacteria>actinobacteria	Promicromonosporaceae bacterium W15	hypothetical protein [Promicromonosporaceae bacterium W15].
WP_047232618.1	-	399	bacteria>actinobacteria	Cellulosimicrobium	MULTISPECIES: hypothetical protein [Cellulosimicrobium].
WP_048342249.1	-	399	bacteria>actinobacteria	Cellulomonas sp. A375-1	hypothetical protein [Cellulomonas sp. A375-1].
WP_051269784.1	-	399	bacteria>actinobacteria	Streptomyces sp. TAA040	hypothetical protein [Streptomyces sp. TAA040].
WP_051737210.1	-	399	bacteria>actinobacteria	Pseudonocardia autotrophica	hypothetical protein [Pseudonocardia autotrophica].
WP_056648600.1	-	399	bacteria>actinobacteria	Oerskovia	MULTISPECIES: hypothetical protein [Oerskovia].
WP_061269477.1	-	399	bacteria>actinobacteria	Cellulosimicrobium funkei	hypothetical protein [Cellulosimicrobium funkei].
OCI31093.1	OERS_21670	399	bacteria>actinobacteria	Oerskovia enterophila	hypothetical protein OERS_21670 [Oerskovia enterophila].
WP_012867417.1	-	398	bacteria>actinobacteria	Sanguibacter keddieii	hypothetical protein [Sanguibacter keddieii].
WP_028047100.1	-	398	bacteria>actinobacteria	Cellulomonas sp. URHE0023	hypothetical protein [Cellulomonas sp. URHE0023].
WP_028049178.1	-	398	bacteria>actinobacteria	Cellulomonas sp. URHD0024	hypothetical protein [Cellulomonas sp. URHD0024].
WP_035059558.1	-	398	bacteria>actinobacteria	Cellulomonas bogoriensis	hypothetical protein [Cellulomonas bogoriensis].
WP_042173726.1	-	398	bacteria>actinobacteria	Streptomyces	MULTISPECIES: hypothetical protein [Streptomyces].
WP_046528051.1	-	398	bacteria>actinobacteria	Cellulomonas sp. FA1	hypothetical protein [Cellulomonas sp. FA1].
WP_052850300.1	-	398	bacteria>actinobacteria	Streptomyces avicenniae	hypothetical protein [Streptomyces avicenniae].
WP_055922426.1	-	398	bacteria>actinobacteria	Cellulomonas sp. Root485	hypothetical protein [Cellulomonas sp. Root485].
WP_056021386.1	-	398	bacteria>actinobacteria	Cellulomonas sp. Leaf395	hypothetical protein [Cellulomonas sp. Leaf395].
WP_056092939.1	-	398	bacteria>actinobacteria	Cellulomonas sp. Root137	hypothetical protein [Cellulomonas sp. Root137].
WP_056129820.1	-	398	bacteria>actinobacteria	Sanguibacter sp. Leaf3	hypothetical protein [Sanguibacter sp. Leaf3].
WP_056591164.1	-	398	bacteria>actinobacteria	Cellulomonas sp. Leaf334	hypothetical protein [Cellulomonas sp. Leaf334].
WP_057212181.1	-	398	bacteria>actinobacteria	Cellulomonas sp. Root930	hypothetical protein [Cellulomonas sp. Root930].
WP_062104176.1	-	398	bacteria>actinobacteria	Cellulomonas sp. B6	hypothetical protein [Cellulomonas sp. B6].
WP_029289410.1	-	397	bacteria>actinobacteria	Cellulomonas sp. HZM	hypothetical protein [Cellulomonas sp. HZM].
WP_015400486.1	-	395	bacteria>actinobacteria	Corynebacterium halotolerans	hypothetical protein [Corynebacterium halotolerans].
WP_019137580.1	-	395	bacteria>actinobacteria	Cellulomonas massiliensis	hypothetical protein [Cellulomonas massiliensis].
GAT79896.1	STXM2123_596	395	bacteria>actinobacteria	Streptomyces sp. F-3	hypothetical protein STXM2123_596 [Streptomyces sp. F-3].
WP_029252512.1	-	394	bacteria>actinobacteria	Paraoerskovia marina	hypothetical protein [Paraoerskovia marina].
WP_030555036.1	-	394	bacteria>actinobacteria	Streptomyces exfoliatus	hypothetical protein [Streptomyces exfoliatus].
WP_046260696.1	-	394	bacteria>actinobacteria	Streptomyces sp. WM6386	hypothetical protein [Streptomyces sp. WM6386].
WP_055499007.1	-	394	bacteria>actinobacteria	Streptomyces flocculus	hypothetical protein [Streptomyces flocculus].
EQM17092.1	GuangZ0019_3758	393	bacteria>actinobacteria	Mycobacterium tuberculosis GuangZ0019	hypothetical protein GuangZ0019_3758 [Mycobacterium tuberculosis GuangZ0019].
WP_020016947.1	-	392	bacteria>actinobacteria	Promicromonospora sukumoe	hypothetical protein [Promicromonospora sukumoe].
WP_036969267.1	-	392	bacteria>actinobacteria	Promicromonospora kroppenstedtii	hypothetical protein [Promicromonospora kroppenstedtii].
WP_042621106.1	-	392	bacteria>actinobacteria	Corynebacterium marinum	hypothetical protein [Corynebacterium marinum].
WP_049568387.1	-	392	bacteria>actinobacteria	Streptomyces sp. SBT349	hypothetical protein [Streptomyces sp. SBT349].
WP_016466974.1	-	391	bacteria>actinobacteria	Streptomyces	MULTISPECIES: hypothetical protein [Streptomyces].
WP_020934963.1	-	391	bacteria>actinobacteria	Corynebacterium maris	hypothetical protein [Corynebacterium maris].
WP_030407109.1	-	391	bacteria>actinobacteria	Streptomyces sp. NRRL F-5917	hypothetical protein [Streptomyces sp. NRRL F-5917].
WP_031027872.1	-	391	bacteria>actinobacteria	Streptomyces	MULTISPECIES: hypothetical protein [Streptomyces].
WP_031175125.1	-	391	bacteria>actinobacteria	Streptomyces	MULTISPECIES: hypothetical protein [Streptomyces].
WP_052527081.1	-	391	bacteria>actinobacteria	Kineosporia aurantiaca	hypothetical protein [Kineosporia aurantiaca].
WP_027765017.1	-	390	bacteria>actinobacteria	Streptomyces sp. CNS606	hypothetical protein [Streptomyces sp. CNS606].
WP_042422805.1	-	390	bacteria>actinobacteria	Streptacidiphilus anmyonensis	hypothetical protein [Streptacidiphilus anmyonensis].
WP_043058189.1	-	390	unclassified	Bacteria	MULTISPECIES: hypothetical protein [Bacteria].
WP_059005966.1	-	390	bacteria>actinobacteria	Streptomyces specialis	hypothetical protein [Streptomyces specialis].
WP_027753821.1	-	389	bacteria>actinobacteria	Streptomyces sp. CNH099	hypothetical protein [Streptomyces sp. CNH099].
WP_030213690.1	-	389	bacteria>actinobacteria	Streptomyces griseoluteus	hypothetical protein [Streptomyces griseoluteus].
WP_017537702.1	-	388	bacteria>actinobacteria	Nocardiopsis halophila	hypothetical protein [Nocardiopsis halophila].
WP_027759445.1	-	388	bacteria>actinobacteria	Streptomyces sp. CNT318	hypothetical protein [Streptomyces sp. CNT318].
WP_031130622.1	-	388	bacteria>actinobacteria	Streptomyces sp. NRRL WC-3719	hypothetical protein [Streptomyces sp. NRRL WC-3719].
WP_040250964.1	-	388	bacteria>actinobacteria	Streptomyces albus	hypothetical protein [Streptomyces albus].
WP_065965776.1	-	388	bacteria>actinobacteria	Streptomyces sparsogenes	hypothetical protein [Streptomyces sparsogenes].
AGM05563.1	AORI_2977	387	bacteria>actinobacteria	Amycolatopsis orientalis HCCB10007	hypothetical protein AORI_2977 [Amycolatopsis orientalis HCCB10007].
WP_023590819.1	-	387	bacteria>actinobacteria	Streptomyces thermolilacinus	hypothetical protein [Streptomyces thermolilacinus].
WP_026129027.1	-	387	bacteria>actinobacteria	Nocardiopsis prasina	hypothetical protein [Nocardiopsis prasina].
WP_029553371.1	-	387	bacteria>actinobacteria	Streptomyces somaliensis	hypothetical protein [Streptomyces somaliensis].
WP_031074053.1	-	387	bacteria>actinobacteria	Streptomyces sp. NRRL S-118	hypothetical protein [Streptomyces sp. NRRL S-118].
WP_037606199.1	-	387	bacteria>actinobacteria	Streptacidiphilus rugosus	hypothetical protein [Streptacidiphilus rugosus].
WP_042447212.1	-	387	bacteria>actinobacteria	Streptacidiphilus jiangxiensis	hypothetical protein [Streptacidiphilus jiangxiensis].
WP_049573062.1	-	387	bacteria>actinobacteria	Nocardiopsis sp. SBT366	hypothetical protein [Nocardiopsis sp. SBT366].
WP_058940141.1	-	387	bacteria>actinobacteria	Streptomyces kanasensis	hypothetical protein [Streptomyces kanasensis].
KZF39514.1	AVR91_05555	387	bacteria>actinobacteria	Amycolatopsis keratiniphila subsp. keratiniphila	hypothetical protein AVR91_05555 [Amycolatopsis keratiniphila subsp. keratiniphila].
WP_014141000.1	-	386	bacteria>actinobacteria	Streptomyces cattleya	hypothetical protein [Streptomyces cattleya].
WP_062213389.1	-	386	bacteria>actinobacteria	Streptomyces sp. NBRC 109706	hypothetical protein [Streptomyces sp. NBRC 109706].
WP_064072336.1	-	386	bacteria>actinobacteria	Streptomyces albulus	hypothetical protein [Streptomyces albulus].
WP_009712470.1	-	385	bacteria>actinobacteria	Streptomyces himastatinicus	hypothetical protein [Streptomyces himastatinicus].
WP_013674482.1	-	385	bacteria>actinobacteria	Pseudonocardia dioxanivorans	hypothetical protein [Pseudonocardia dioxanivorans].
WP_026119143.1	-	385	bacteria>actinobacteria	Nocardiopsis ganjiahuensis	hypothetical protein [Nocardiopsis ganjiahuensis].
WP_026862686.1	-	385	bacteria>actinobacteria	Intrasporangiaceae bacterium URHB0013	hypothetical protein [Intrasporangiaceae bacterium URHB0013].
WP_027738272.1	-	385	bacteria>actinobacteria	Streptomyces sp. CNT360	hypothetical protein [Streptomyces sp. CNT360].
WP_030231696.1	-	385	bacteria>actinobacteria	Streptomyces sp. NRRL S-350	hypothetical protein [Streptomyces sp. NRRL S-350].
WP_030270306.1	-	385	bacteria>actinobacteria	Streptomyces sp. NRRL B-24484	hypothetical protein [Streptomyces sp. NRRL B-24484].
WP_030284967.1	-	385	bacteria>actinobacteria	Streptomyces	MULTISPECIES: hypothetical protein [Streptomyces].
WP_033350129.1	-	385	bacteria>actinobacteria	Streptomyces aureofaciens	hypothetical protein [Streptomyces aureofaciens].
WP_045700291.1	-	385	bacteria>actinobacteria	Streptomyces rubellomurinus	hypothetical protein [Streptomyces rubellomurinus].
WP_055555690.1	-	385	bacteria>actinobacteria	Streptomyces sp. NBRC 110028	hypothetical protein [Streptomyces sp. NBRC 110028].
WP_063731246.1	-	385	bacteria>actinobacteria	Streptomyces sp. RTd22	hypothetical protein [Streptomyces sp. RTd22].
WP_014057034.1	-	384	bacteria>actinobacteria	Streptomyces violaceusniger	hypothetical protein [Streptomyces violaceusniger].
WP_014173536.1	-	384	bacteria>actinobacteria	Streptomyces bingchenggensis	hypothetical protein [Streptomyces bingchenggensis].
AGP52842.1	M271_06080	384	bacteria>actinobacteria	Streptomyces rapamycinicus NRRL 5491	hypothetical protein M271_06080 [Streptomyces rapamycinicus NRRL 5491].
WP_026116460.1	-	384	bacteria>actinobacteria	Nocardiopsis valliformis	hypothetical protein [Nocardiopsis valliformis].
WP_030841542.1	-	384	bacteria>actinobacteria	Streptomyces hygroscopicus	hypothetical protein [Streptomyces hygroscopicus].
WP_030877419.1	-	384	bacteria>actinobacteria	Streptomyces varsoviensis	hypothetical protein [Streptomyces varsoviensis].
WP_037950357.1	-	384	bacteria>actinobacteria	Streptomyces sp. PRh5	hypothetical protein [Streptomyces sp. PRh5].
WP_044567852.1	-	384	bacteria>actinobacteria	Streptomyces iranensis	hypothetical protein [Streptomyces iranensis].
AJT62674.1	T261_0986	384	bacteria>actinobacteria	Streptomyces lydicus A02	hypothetical protein T261_0986 [Streptomyces lydicus A02].
WP_046088249.1	-	384	bacteria>actinobacteria	Streptomyces sp. MUSC164	hypothetical protein [Streptomyces sp. MUSC164].
WP_048455371.1	-	384	bacteria>actinobacteria	Streptomyces sp. HNS054	hypothetical protein [Streptomyces sp. HNS054].
WP_059144604.1	-	384	bacteria>actinobacteria	Streptomyces violaceusniger	hypothetical protein [Streptomyces violaceusniger].
WP_060947925.1	-	384	bacteria>actinobacteria	Streptomyces hygroscopicus	hypothetical protein [Streptomyces hygroscopicus].
WP_060948583.1	-	384	bacteria>actinobacteria	Streptomyces	MULTISPECIES: hypothetical protein [Streptomyces].
WP_065967383.1	-	384	bacteria>actinobacteria	Streptomyces sparsogenes	hypothetical protein [Streptomyces sparsogenes].
WP_016471103.1	-	383	bacteria>actinobacteria	Streptomyces sp. HPH0547	hypothetical protein [Streptomyces sp. HPH0547].
WP_022916774.1	-	383	bacteria>actinobacteria	Ruania albidiflava	hypothetical protein [Ruania albidiflava].
WP_024449060.1	-	383	bacteria>actinobacteria	Mycobacterium iranicum	hypothetical protein [Mycobacterium iranicum].
WP_026115690.1	-	383	bacteria>actinobacteria	Nocardiopsis kunsanensis	hypothetical protein [Nocardiopsis kunsanensis].
WP_030405437.1	-	383	bacteria>actinobacteria	Streptomyces	MULTISPECIES: hypothetical protein [Streptomyces].
WP_031073890.1	-	383	bacteria>actinobacteria	Streptomyces sp. NRRL S-118	hypothetical protein [Streptomyces sp. NRRL S-118].
WP_042380511.1	-	383	bacteria>actinobacteria	Streptacidiphilus melanogenes	hypothetical protein [Streptacidiphilus melanogenes].
WP_055590281.1	-	383	bacteria>actinobacteria	Streptomyces griseoplanus	hypothetical protein [Streptomyces griseoplanus].
WP_064280909.1	-	383	bacteria>actinobacteria	Mycobacterium iranicum	hypothetical protein [Mycobacterium iranicum].
EFG74207.1	HMPREF0591_5892	382	bacteria>actinobacteria	Mycobacterium parascrofulaceum ATCC BAA-614	hypothetical protein HMPREF0591_5892 [Mycobacterium parascrofulaceum ATCC BAA-614].
WP_013153159.1	-	382	bacteria>actinobacteria	Nocardiopsis dassonvillei	hypothetical protein [Nocardiopsis dassonvillei].
WP_017565896.1	-	382	bacteria>actinobacteria	Nocardiopsis synnemataformans	hypothetical protein [Nocardiopsis synnemataformans].
WP_017568933.1	-	382	bacteria>actinobacteria	Nocardiopsis halotolerans	hypothetical protein [Nocardiopsis halotolerans].
WP_019607210.1	-	382	bacteria>actinobacteria	Nocardiopsis sp. CNS-639	hypothetical protein [Nocardiopsis sp. CNS-639].
AGZ51256.1	MKAN_14005	382	bacteria>actinobacteria	Mycobacterium kansasii ATCC 12478	hypothetical protein MKAN_14005 [Mycobacterium kansasii ATCC 12478].
WP_030206206.1	-	382	bacteria>actinobacteria	Streptomyces sp. NRRL S-87	hypothetical protein [Streptomyces sp. NRRL S-87].
WP_030217353.1	-	382	bacteria>actinobacteria	Streptomyces bikiniensis	hypothetical protein [Streptomyces bikiniensis].
WP_030293258.1	-	382	bacteria>actinobacteria	Streptomyces katrae	hypothetical protein [Streptomyces katrae].
WP_030936843.1	-	382	bacteria>actinobacteria	Streptomyces sp. NRRL S-646	hypothetical protein [Streptomyces sp. NRRL S-646].
WP_037868937.1	-	382	bacteria>actinobacteria	Streptomyces sp. NRRL S-813	hypothetical protein [Streptomyces sp. NRRL S-813].
WP_041132319.1	-	382	bacteria>actinobacteria	Streptomyces vietnamensis	hypothetical protein [Streptomyces vietnamensis].
WP_044364385.1	-	382	bacteria>actinobacteria	Streptomyces natalensis	hypothetical protein [Streptomyces natalensis].
WP_046362720.1	-	382	bacteria>actinobacteria	Mycobacterium obuense	hypothetical protein [Mycobacterium obuense].
WP_047869533.1	-	382	bacteria>actinobacteria	Nocardiopsis sp. RV163	hypothetical protein [Nocardiopsis sp. RV163].
WP_048424690.1	-	382	bacteria>actinobacteria	Mycobacterium obuense	hypothetical protein [Mycobacterium obuense].
WP_053640410.1	-	382	bacteria>actinobacteria	Streptomyces	MULTISPECIES: hypothetical protein [Streptomyces].
WP_053747238.1	-	382	bacteria>actinobacteria	Streptomyces sp. MMG1533	hypothetical protein [Streptomyces sp. MMG1533].
WP_053790582.1	-	382	bacteria>actinobacteria	Streptomyces sp. XY332	hypothetical protein [Streptomyces sp. XY332].
WP_053924581.1	-	382	bacteria>actinobacteria	Streptomyces chattanoogensis	hypothetical protein [Streptomyces chattanoogensis].
WP_058845549.1	-	382	bacteria>actinobacteria	Streptomyces silvensis	hypothetical protein [Streptomyces silvensis].
WP_061079815.1	-	382	bacteria>actinobacteria	Nocardiopsis dassonvillei	hypothetical protein [Nocardiopsis dassonvillei].
WP_062760133.1	-	382	bacteria>actinobacteria	Streptomyces sp. WAC04657	hypothetical protein [Streptomyces sp. WAC04657].
WP_009315555.1	-	381	bacteria>actinobacteria	Streptomyces ipomoeae	hypothetical protein [Streptomyces ipomoeae].
WP_014814305.1	-	381	bacteria>actinobacteria	Mycobacterium chubuense	hypothetical protein [Mycobacterium chubuense].
WP_017611701.1	-	381	bacteria>actinobacteria	Nocardiopsis salina	hypothetical protein [Nocardiopsis salina].
WP_030690429.1	-	381	bacteria>actinobacteria	Streptomyces globisporus	hypothetical protein [Streptomyces globisporus].
WP_030878279.1	-	381	bacteria>actinobacteria	Streptomyces sp. NRRL S-1868	hypothetical protein [Streptomyces sp. NRRL S-1868].
WP_030890357.1	-	381	bacteria>actinobacteria	Streptomyces sp. NRRL F-5053	hypothetical protein [Streptomyces sp. NRRL F-5053].
AJC61194.1	GZL_08671	381	bacteria>actinobacteria	Streptomyces sp. 769	hypothetical protein GZL_08671 [Streptomyces sp. 769].
WP_042364605.1	-	381	bacteria>actinobacteria	Streptacidiphilus neutrinimicus	hypothetical protein [Streptacidiphilus neutrinimicus].
WP_045947267.1	-	381	bacteria>actinobacteria	Streptomyces katrae	hypothetical protein [Streptomyces katrae].
KUL38959.1	ADL22_16485	381	bacteria>actinobacteria	Streptomyces sp. NRRL F-4489	hypothetical protein ADL22_16485 [Streptomyces sp. NRRL F-4489].
WP_003982354.1	-	380	bacteria>actinobacteria	Streptomyces	MULTISPECIES: hypothetical protein [Streptomyces].
WP_030021859.1	-	380	bacteria>actinobacteria	Streptomyces monomycini	hypothetical protein [Streptomyces monomycini].
WP_030374255.1	-	380	bacteria>actinobacteria	Streptomyces rimosus	hypothetical protein [Streptomyces rimosus].
WP_030412663.1	-	380	bacteria>actinobacteria	Streptomyces sp. NRRL S-1448	hypothetical protein [Streptomyces sp. NRRL S-1448].
WP_030594808.1	-	380	bacteria>actinobacteria	Streptomyces	MULTISPECIES: hypothetical protein [Streptomyces].
WP_030632118.1	-	380	bacteria>actinobacteria	Streptomyces rimosus	hypothetical protein [Streptomyces rimosus].
WP_030639503.1	-	380	bacteria>actinobacteria	Streptomyces rimosus	hypothetical protein [Streptomyces rimosus].
WP_030678729.1	-	380	bacteria>actinobacteria	Streptomyces rimosus	hypothetical protein [Streptomyces rimosus].
WP_030804181.1	-	380	bacteria>actinobacteria	Streptomyces sp. NRRL S-337	hypothetical protein [Streptomyces sp. NRRL S-337].
WP_031006087.1	-	380	bacteria>actinobacteria	Streptomyces sp. NRRL WC-3773	hypothetical protein [Streptomyces sp. NRRL WC-3773].
WP_031189766.1	-	380	bacteria>actinobacteria	Streptomyces	MULTISPECIES: hypothetical protein [Streptomyces].
WP_033320247.1	-	380	bacteria>actinobacteria	Streptomyces yerevanensis	hypothetical protein [Streptomyces yerevanensis].
WP_042830231.1	-	380	bacteria>actinobacteria	Streptomyces rimosus	hypothetical protein [Streptomyces rimosus].
AJT63141.1	T261_1455	380	bacteria>actinobacteria	Streptomyces lydicus A02	hypothetical protein T261_1455 [Streptomyces lydicus A02].
CNG72380.1	ERS075342_07756	380	bacteria>actinobacteria	Mycobacterium tuberculosis	Uncharacterised protein [Mycobacterium tuberculosis].
WP_053698021.1	-	380	bacteria>actinobacteria	Streptomyces sp. NRRL F-5755	hypothetical protein [Streptomyces sp. NRRL F-5755].
WP_053802083.1	-	380	bacteria>actinobacteria	Streptomyces rimosus	hypothetical protein [Streptomyces rimosus].
WP_055690303.1	-	380	bacteria>actinobacteria	Streptomyces prasinus	hypothetical protein [Streptomyces prasinus].
KUL36869.1	ADL22_23520	380	bacteria>actinobacteria	Streptomyces sp. NRRL F-4489	hypothetical protein ADL22_23520 [Streptomyces sp. NRRL F-4489].
WP_060730408.1	-	380	bacteria>actinobacteria	Streptomyces albus	hypothetical protein [Streptomyces albus].
WP_065000517.1	-	380	bacteria>actinobacteria	Streptomyces sp. H-KF8	hypothetical protein [Streptomyces sp. H-KF8].
WP_016640011.1	-	379	bacteria>actinobacteria	Streptomyces aurantiacus	hypothetical protein [Streptomyces aurantiacus].
WP_017607739.1	-	379	bacteria>actinobacteria	Nocardiopsis xinjiangensis	hypothetical protein [Nocardiopsis xinjiangensis].
WP_018088707.1	-	379	bacteria>actinobacteria	Streptomyces sp. FxanaC1	hypothetical protein [Streptomyces sp. FxanaC1].
WP_030079982.1	-	379	bacteria>actinobacteria	Streptomyces decoyicus	hypothetical protein [Streptomyces decoyicus].
WP_030984823.1	-	379	bacteria>actinobacteria	Streptomyces sp. NRRL S-1813	hypothetical protein [Streptomyces sp. NRRL S-1813].
WP_033265326.1	-	379	bacteria>actinobacteria	Streptomyces lydicus	hypothetical protein [Streptomyces lydicus].
WP_042157974.1	-	379	bacteria>actinobacteria	Streptomyces sp. NBRC 110027	hypothetical protein [Streptomyces sp. NBRC 110027].
WP_017824657.1	-	378	bacteria>actinobacteria	Brachybacterium muris	hypothetical protein [Brachybacterium muris].
WP_026127415.1	-	378	bacteria>actinobacteria	Nocardiopsis lucentensis	hypothetical protein [Nocardiopsis lucentensis].
WP_028647697.1	-	378	bacteria>actinobacteria	Nocardiopsis sp. CNT312	hypothetical protein [Nocardiopsis sp. CNT312].
WP_030250989.1	-	378	bacteria>actinobacteria	Streptomyces violens	hypothetical protein [Streptomyces violens].
WP_030610853.1	-	378	bacteria>actinobacteria	Streptomyces sclerotialus	hypothetical protein [Streptomyces sclerotialus].
WP_030746267.1	-	378	bacteria>actinobacteria	Streptomyces sp. NRRL F-5135	hypothetical protein [Streptomyces sp. NRRL F-5135].
WP_031061571.1	-	378	bacteria>actinobacteria	Streptomyces ochraceiscleroticus	hypothetical protein [Streptomyces ochraceiscleroticus].
WP_052867283.1	-	378	bacteria>actinobacteria	Streptomyces niger	hypothetical protein [Streptomyces niger].
WP_006436581.1	-	377	bacteria>actinobacteria	Gordonia amicalis	hypothetical protein [Gordonia amicalis].
WP_010843177.1	-	377	bacteria>actinobacteria	Gordonia terrae	hypothetical protein [Gordonia terrae].
WP_014928537.1	-	377	bacteria>actinobacteria	Gordonia sp. KTR9	hypothetical protein [Gordonia sp. KTR9].
WP_024499187.1	-	377	bacteria>actinobacteria	Gordonia amicalis	hypothetical protein [Gordonia amicalis].
WP_024876547.1	-	377	bacteria>actinobacteria	Saccharomonospora sp. CNQ490	hypothetical protein [Saccharomonospora sp. CNQ490].
WP_055475965.1	-	377	bacteria>actinobacteria	Gordonia sp. HS-NH1	hypothetical protein [Gordonia sp. HS-NH1].
WP_057151093.1	-	377	bacteria>actinobacteria	Mycobacterium sp. Soil538	hypothetical protein [Mycobacterium sp. Soil538].
WP_058253792.1	-	377	bacteria>actinobacteria	Gordonia sp. SGD-V-85	hypothetical protein [Gordonia sp. SGD-V-85].
WP_065049361.1	-	377	bacteria>actinobacteria	Mycobacterium gordonae	hypothetical protein [Mycobacterium gordonae].
WP_065164478.1	-	377	bacteria>actinobacteria	Mycobacterium gordonae	hypothetical protein [Mycobacterium gordonae].
WP_065630255.1	-	377	bacteria>actinobacteria	Gordonia sp. UCD-TK1	hypothetical protein [Gordonia sp. UCD-TK1].
ANW65059.1	BCA37_16970	377	bacteria>actinobacteria	Mycobacterium sp. djl-10	hypothetical protein BCA37_16970 [Mycobacterium sp. djl-10].
OCW86358.1	A8M60_21595	377	bacteria>actinobacteria	Nocardia farcinica	hypothetical protein A8M60_21595 [Nocardia farcinica].
WP_003881952.1	-	376	bacteria>actinobacteria	Mycobacterium	MULTISPECIES: hypothetical protein [Mycobacterium].
WP_014145592.1	-	376	bacteria>actinobacteria	Streptomyces cattleya	hypothetical protein [Streptomyces cattleya].
WP_014911467.1	-	376	bacteria>actinobacteria	Nocardiopsis alba	hypothetical protein [Nocardiopsis alba].
WP_017534845.1	-	376	bacteria>actinobacteria	Nocardiopsis	MULTISPECIES: hypothetical protein [Nocardiopsis].
WP_017602766.1	-	376	bacteria>actinobacteria	Nocardiopsis alkaliphila	hypothetical protein [Nocardiopsis alkaliphila].
WP_029109455.1	-	376	bacteria>actinobacteria	Mycobacterium sp. URHD0025	hypothetical protein [Mycobacterium sp. URHD0025].
WP_053617339.1	-	376	bacteria>actinobacteria	Nocardiopsis sp. NRRL B-16309	hypothetical protein [Nocardiopsis sp. NRRL B-16309].
WP_054602402.1	-	376	bacteria>actinobacteria	Mycobacterium fortuitum	hypothetical protein [Mycobacterium fortuitum].
OBG41826.1	A5672_12195	376	bacteria>actinobacteria	Mycobacterium sp. E2978	hypothetical protein A5672_12195 [Mycobacterium sp. E2978].
OBJ05709.1	A5660_15475	376	bacteria>actinobacteria	Mycobacterium sp. 1137317.9	hypothetical protein A5660_15475 [Mycobacterium sp. 1137317.9].
WP_064848354.1	-	376	bacteria>actinobacteria	Mycobacterium fortuitum	hypothetical protein [Mycobacterium fortuitum].
WP_064912441.1	-	376	bacteria>actinobacteria	Mycobacterium fortuitum	hypothetical protein [Mycobacterium fortuitum].
WP_064927006.1	-	376	bacteria>actinobacteria	Mycobacterium fortuitum	hypothetical protein [Mycobacterium fortuitum].
WP_065068855.1	-	376	bacteria>actinobacteria	Mycobacterium fortuitum	hypothetical protein [Mycobacterium fortuitum].
WP_065071487.1	-	376	bacteria>actinobacteria	Mycobacterium fortuitum	hypothetical protein [Mycobacterium fortuitum].
WP_065148312.1	-	376	bacteria>actinobacteria	Mycobacterium fortuitum	hypothetical protein [Mycobacterium fortuitum].
ABS01526.1	Krad_0034	375	bacteria>actinobacteria	Kineococcus radiotolerans SRS30216 = ATCC BAA-149	conserved hypothetical protein [Kineococcus radiotolerans SRS30216 = ATCC BAA-149].
WP_009310743.1	-	375	bacteria>actinobacteria	Streptomyces ipomoeae	hypothetical protein [Streptomyces ipomoeae].
WP_010038692.1	-	375	bacteria>actinobacteria	Streptomyces chartreusis	hypothetical protein [Streptomyces chartreusis].
WP_010643216.1	-	375	bacteria>actinobacteria	Streptomyces sp. S4	hypothetical protein [Streptomyces sp. S4].
WP_015507321.1	-	375	bacteria>actinobacteria	Streptomyces albus	hypothetical protein [Streptomyces albus].
WP_018383287.1	-	375	bacteria>actinobacteria	Streptomyces vitaminophilus	hypothetical protein [Streptomyces vitaminophilus].
WP_018471749.1	-	375	bacteria>actinobacteria	Streptomyces sp. LaPpAH-202	hypothetical protein [Streptomyces sp. LaPpAH-202].
WP_018895981.1	-	375	bacteria>actinobacteria	Streptomyces sp. CNY228	hypothetical protein [Streptomyces sp. CNY228].
WP_023417704.1	-	375	bacteria>actinobacteria	Streptomyces	MULTISPECIES: hypothetical protein [Streptomyces].
WP_027747444.1	-	375	bacteria>actinobacteria	Streptomyces sp. CNH287	hypothetical protein [Streptomyces sp. CNH287].
WP_029385297.1	-	375	bacteria>actinobacteria	Streptomyces leeuwenhoekii	hypothetical protein [Streptomyces leeuwenhoekii].
WP_030310460.1	-	375	bacteria>actinobacteria	Streptomyces albidoflavus	hypothetical protein [Streptomyces albidoflavus].
WP_030699309.1	-	375	bacteria>actinobacteria	Streptomyces griseus	hypothetical protein [Streptomyces griseus].
WP_033240949.1	-	375	bacteria>actinobacteria	Streptomyces griseus	hypothetical protein [Streptomyces griseus].
WP_033953128.1	-	375	bacteria>actinobacteria	Streptomyces sp. CNQ431	hypothetical protein [Streptomyces sp. CNQ431].
WP_044385641.1	-	375	bacteria>actinobacteria	Streptomyces cyaneogriseus	hypothetical protein [Streptomyces cyaneogriseus].
WP_047142823.1	-	375	bacteria>actinobacteria	Streptomyces sp. KE1	hypothetical protein [Streptomyces sp. KE1].
WP_047467893.1	-	375	bacteria>actinobacteria	Streptomyces sp. M10	hypothetical protein [Streptomyces sp. M10].
WP_049978888.1	-	375	bacteria>actinobacteria	Streptomyces	MULTISPECIES: hypothetical protein [Streptomyces].
SCF15871.1	GA0070214_107176	375	bacteria>actinobacteria	Micromonospora chaiyaphumensis	hypothetical protein GA0070214_107176 [Micromonospora chaiyaphumensis].
WP_005321243.1	-	374	bacteria>actinobacteria	Streptomyces pristinaespiralis	hypothetical protein [Streptomyces pristinaespiralis].
WP_007076361.1	-	374	bacteria>actinobacteria	Micromonospora sp. ATCC 39149	hypothetical protein [Micromonospora sp. ATCC 39149].
WP_007459689.1	-	374	bacteria>actinobacteria	Micromonospora lupini	hypothetical protein [Micromonospora lupini].
WP_009946651.1	-	374	bacteria>actinobacteria	Saccharopolyspora erythraea	hypothetical protein [Saccharopolyspora erythraea].
WP_011906021.1	-	374	bacteria>actinobacteria	Salinispora	MULTISPECIES: hypothetical protein [Salinispora].
WP_012182451.1	-	374	bacteria>actinobacteria	Salinispora arenicola	hypothetical protein [Salinispora arenicola].
WP_013286024.1	-	374	bacteria>actinobacteria	Micromonospora	MULTISPECIES: hypothetical protein [Micromonospora].
WP_013477007.1	-	374	bacteria>actinobacteria	Micromonospora sp. L5	hypothetical protein [Micromonospora sp. L5].
WP_014691701.1	-	374	bacteria>actinobacteria	Actinoplanes sp. SE50/110	peptide chain release factor 1 [Actinoplanes sp. SE50/110].
WP_016574937.1	-	374	bacteria>actinobacteria	Streptomyces albulus	hypothetical protein [Streptomyces albulus].
WP_016812237.1	-	374	bacteria>actinobacteria	Salinispora arenicola	hypothetical protein [Salinispora arenicola].
WP_018215284.1	-	374	bacteria>actinobacteria	Salinispora pacifica	hypothetical protein [Salinispora pacifica].
WP_018219911.1	-	374	bacteria>actinobacteria	Salinispora pacifica	hypothetical protein [Salinispora pacifica].
WP_018252829.1	-	374	bacteria>actinobacteria	Salinispora pacifica	hypothetical protein [Salinispora pacifica].
WP_018537254.1	-	374	bacteria>actinobacteria	Streptomyces sp. MspMP-M5	hypothetical protein [Streptomyces sp. MspMP-M5].
WP_018587377.1	-	374	bacteria>actinobacteria	Salinispora arenicola	hypothetical protein [Salinispora arenicola].
WP_018723471.1	-	374	bacteria>actinobacteria	Salinispora pacifica	hypothetical protein [Salinispora pacifica].
WP_018725130.1	-	374	bacteria>actinobacteria	Salinispora pacifica	hypothetical protein [Salinispora pacifica].
WP_018731054.1	-	374	bacteria>actinobacteria	Salinispora pacifica	hypothetical protein [Salinispora pacifica].
WP_018736469.1	-	374	bacteria>actinobacteria	Salinispora pacifica	hypothetical protein [Salinispora pacifica].
WP_018739630.1	-	374	bacteria>actinobacteria	Salinispora pacifica	hypothetical protein [Salinispora pacifica].
WP_018788433.1	-	374	bacteria>actinobacteria	Micromonospora sp. CNB394	hypothetical protein [Micromonospora sp. CNB394].
WP_018792916.1	-	374	bacteria>actinobacteria	Salinispora arenicola	hypothetical protein [Salinispora arenicola].
WP_018795683.1	-	374	bacteria>actinobacteria	Salinispora arenicola	hypothetical protein [Salinispora arenicola].
WP_018803149.1	-	374	bacteria>actinobacteria	Salinispora arenicola	hypothetical protein [Salinispora arenicola].
WP_018813470.1	-	374	bacteria>actinobacteria	Salinispora pacifica	hypothetical protein [Salinispora pacifica].
WP_018818276.1	-	374	bacteria>actinobacteria	Salinispora pacifica	hypothetical protein [Salinispora pacifica].
WP_018821010.1	-	374	bacteria>actinobacteria	Salinispora pacifica	hypothetical protein [Salinispora pacifica].
WP_018826880.1	-	374	bacteria>actinobacteria	Salinispora pacifica	hypothetical protein [Salinispora pacifica].
WP_019871977.1	-	374	bacteria>actinobacteria	Salinispora pacifica	hypothetical protein [Salinispora pacifica].
WP_020216276.1	-	374	bacteria>actinobacteria	Salinispora arenicola	hypothetical protein [Salinispora arenicola].
WP_023361236.1	-	374	bacteria>actinobacteria	Actinoplanes friuliensis	hypothetical protein [Actinoplanes friuliensis].
WP_025617782.1	-	374	bacteria>actinobacteria	Salinispora pacifica	hypothetical protein [Salinispora pacifica].
WP_026325131.1	-	374	bacteria>actinobacteria	Salinispora pacifica	hypothetical protein [Salinispora pacifica].
WP_027647166.1	-	374	bacteria>actinobacteria	Salinispora pacifica	hypothetical protein [Salinispora pacifica].
WP_027652856.1	-	374	bacteria>actinobacteria	Salinispora pacifica	hypothetical protein [Salinispora pacifica].
WP_027658021.1	-	374	bacteria>actinobacteria	Salinispora pacifica	hypothetical protein [Salinispora pacifica].
WP_027661327.1	-	374	bacteria>actinobacteria	Salinispora pacifica	hypothetical protein [Salinispora pacifica].
WP_028564163.1	-	374	bacteria>actinobacteria	Salinispora tropica	hypothetical protein [Salinispora tropica].
WP_028675745.1	-	374	bacteria>actinobacteria	Salinispora arenicola	hypothetical protein [Salinispora arenicola].
WP_029023069.1	-	374	bacteria>actinobacteria	Salinispora arenicola	hypothetical protein [Salinispora arenicola].
WP_029127768.1	-	374	bacteria>actinobacteria	Salinispora pacifica	hypothetical protein [Salinispora pacifica].
WP_029129638.1	-	374	bacteria>actinobacteria	Salinispora pacifica	hypothetical protein [Salinispora pacifica].
WP_030332679.1	-	374	bacteria>actinobacteria	Micromonospora parva	hypothetical protein [Micromonospora parva].
WP_030490969.1	-	374	bacteria>actinobacteria	Micromonospora chokoriensis	hypothetical protein [Micromonospora chokoriensis].
WP_030504049.1	-	374	bacteria>actinobacteria	Micromonospora	MULTISPECIES: hypothetical protein [Micromonospora].
WP_033366990.1	-	374	bacteria>actinobacteria	Dactylosporangium aurantiacum	hypothetical protein [Dactylosporangium aurantiacum].
WP_036403601.1	-	374	bacteria>actinobacteria	Mycobacterium kansasii	hypothetical protein [Mycobacterium kansasii].
WP_038846384.1	-	374	bacteria>actinobacteria	Salinispora pacifica	hypothetical protein [Salinispora pacifica].
WP_043968705.1	-	374	bacteria>actinobacteria	Micromonospora carbonacea	hypothetical protein [Micromonospora carbonacea].
WP_044372671.1	-	374	bacteria>actinobacteria	Streptomyces ahygroscopicus	hypothetical protein [Streptomyces ahygroscopicus].
WP_046184260.1	-	374	bacteria>actinobacteria	Mycobacterium nebraskense	hypothetical protein [Mycobacterium nebraskense].
WP_046565056.1	-	374	bacteria>actinobacteria	Micromonospora sp. HK10	hypothetical protein [Micromonospora sp. HK10].
CNW66397.1	ERS013418_03762	374	bacteria>actinobacteria	Mycobacterium tuberculosis	Uncharacterised protein [Mycobacterium tuberculosis].
COV72265.1	ERS007720_00797	374	bacteria>actinobacteria	Mycobacterium tuberculosis	Uncharacterised protein [Mycobacterium tuberculosis].
WP_053655741.1	-	374	bacteria>actinobacteria	Micromonospora sp. NRRL B-16802	hypothetical protein [Micromonospora sp. NRRL B-16802].
WP_055583732.1	-	374	bacteria>actinobacteria	Streptomyces diastatochromogenes	hypothetical protein [Streptomyces diastatochromogenes].
WP_057353484.1	-	374	bacteria>actinobacteria	Mycobacterium tuberculosis	hypothetical protein [Mycobacterium tuberculosis].
KZS57847.1	A4G28_23690	374	bacteria>actinobacteria	Mycobacterium kansasii	hypothetical protein A4G28_23690 [Mycobacterium kansasii].
KZS59329.1	A4G26_12105	374	bacteria>actinobacteria	Mycobacterium kansasii	hypothetical protein A4G26_12105 [Mycobacterium kansasii].
KZS83577.1	A4G31_24100	374	bacteria>actinobacteria	Mycobacterium kansasii	hypothetical protein A4G31_24100 [Mycobacterium kansasii].
WP_063469042.1	-	374	bacteria>actinobacteria	Mycobacterium kansasii	hypothetical protein [Mycobacterium kansasii].
SBT39622.1	GA0070611_0969	374	bacteria>actinobacteria	Micromonospora auratinigra	hypothetical protein GA0070611_0969 [Micromonospora auratinigra].
SBT41608.1	GA0070621_1280	374	bacteria>actinobacteria	Micromonospora narathiwatensis	hypothetical protein GA0070621_1280 [Micromonospora narathiwatensis].
OBB63611.1	A5758_21515	374	bacteria>actinobacteria	Mycobacterium sp. 852014-50255_SCH5639931	hypothetical protein A5758_21515 [Mycobacterium sp. 852014-50255_SCH5639931].
OBB91035.1	A5781_22375	374	bacteria>actinobacteria	Mycobacterium sp. 852002-30065_SCH5024008	hypothetical protein A5781_22375 [Mycobacterium sp. 852002-30065_SCH5024008].
OBF57648.1	A5756_08400	374	bacteria>actinobacteria	Mycobacterium sp. 852002-53434_SCH5985345	hypothetical protein A5756_08400 [Mycobacterium sp. 852002-53434_SCH5985345].
OBF75058.1	A5750_11070	374	bacteria>actinobacteria	Mycobacterium sp. 852002-51613_SCH5001154	hypothetical protein A5750_11070 [Mycobacterium sp. 852002-51613_SCH5001154].
OBG98786.1	A5697_15450	374	bacteria>actinobacteria	Mycobacterium sp. E3251	hypothetical protein A5697_15450 [Mycobacterium sp. E3251].
OBH98377.1	A5678_01735	374	bacteria>actinobacteria	Mycobacterium sp. E2733	hypothetical protein A5678_01735 [Mycobacterium sp. E2733].
OBI33700.1	A5709_21395	374	bacteria>actinobacteria	Mycobacterium sp. E1386	hypothetical protein A5709_21395 [Mycobacterium sp. E1386].
OBI60752.1	A5706_01255	374	bacteria>actinobacteria	Mycobacterium sp. E796	hypothetical protein A5706_01255 [Mycobacterium sp. E796].
SBT67050.1	GA0070622_4102	374	bacteria>actinobacteria	Micromonospora sediminicola	hypothetical protein GA0070622_4102 [Micromonospora sediminicola].
SBV26185.1	GA0070620_1670	374	bacteria>actinobacteria	Micromonospora krabiensis	hypothetical protein GA0070620_1670 [Micromonospora krabiensis].
WP_065443603.1	-	374	bacteria>actinobacteria	Mycobacterium malmoense	hypothetical protein [Mycobacterium malmoense].
WP_065470433.1	-	374	bacteria>actinobacteria	Mycobacterium malmoense	hypothetical protein [Mycobacterium malmoense].
WP_065495743.1	-	374	bacteria>actinobacteria	Mycobacterium malmoense	hypothetical protein [Mycobacterium malmoense].
ANZ20426.1	SNOUR_35990	374	bacteria>actinobacteria	Streptomyces noursei ATCC 11455	hypothetical protein SNOUR_35990 [Streptomyces noursei ATCC 11455].
SCF18532.1	GA0070612_4570	374	bacteria>actinobacteria	Micromonospora chokoriensis	hypothetical protein GA0070612_4570 [Micromonospora chokoriensis].
SCF24094.1	GA0074695_4672	374	bacteria>actinobacteria	Micromonospora viridifaciens	hypothetical protein GA0074695_4672 [Micromonospora viridifaciens].
SCF14159.1	GA0070607_6043	374	bacteria>actinobacteria	Micromonospora coriariae	hypothetical protein GA0070607_6043 [Micromonospora coriariae].
SCF39300.1	GA0070618_6166	374	bacteria>actinobacteria	Micromonospora echinospora	hypothetical protein GA0070618_6166 [Micromonospora echinospora].
SCG16826.1	GA0070610_3100	374	bacteria>actinobacteria	Micromonospora echinofusca	hypothetical protein GA0070610_3100 [Micromonospora echinofusca].
SCF23510.1	GA0070564_104328	374	bacteria>actinobacteria	Micromonospora mirobrigensis	hypothetical protein GA0070564_104328 [Micromonospora mirobrigensis].
SCF44423.1	GA0070215_13046	374	bacteria>actinobacteria	Micromonospora marina	hypothetical protein GA0070215_13046 [Micromonospora marina].
SCF12177.1	GA0070562_0306	374	bacteria>actinobacteria	Micromonospora tulbaghiae	hypothetical protein GA0070562_0306 [Micromonospora tulbaghiae].
SCF41761.1	GA0070561_6496	374	bacteria>actinobacteria	Micromonospora saelicesensis	hypothetical protein GA0070561_6496 [Micromonospora saelicesensis].
SCF10284.1	GA0070558_12854	374	bacteria>actinobacteria	Micromonospora haikouensis	hypothetical protein GA0070558_12854 [Micromonospora haikouensis].
SCE87018.1	GA0070563_102496	374	bacteria>actinobacteria	Micromonospora carbonacea	hypothetical protein GA0070563_102496 [Micromonospora carbonacea].
SCG49083.1	GA0070609_2170	374	bacteria>actinobacteria	Micromonospora echinaurantiaca	hypothetical protein GA0070609_2170 [Micromonospora echinaurantiaca].
SCG56061.1	GA0074704_3222	374	bacteria>actinobacteria	Micromonospora siamensis	hypothetical protein GA0074704_3222 [Micromonospora siamensis].
SCG74201.1	GA0070613_5480	374	bacteria>actinobacteria	Micromonospora inositola	hypothetical protein GA0070613_5480 [Micromonospora inositola].
SCG49461.1	GA0070619_2315	374	bacteria>actinobacteria	Micromonospora zamorensis	hypothetical protein GA0070619_2315 [Micromonospora zamorensis].
SCG43281.1	GA0070213_102539	374	bacteria>actinobacteria	Micromonospora humi	hypothetical protein GA0070213_102539 [Micromonospora humi].
EFE83250.1	SSHG_03692	373	bacteria>actinobacteria	Streptomyces albus J1074	conserved hypothetical protein [Streptomyces albus J1074].
WP_007771956.1	-	373	bacteria>actinobacteria	Mycobacterium colombiense	hypothetical protein [Mycobacterium colombiense].
WP_019328665.1	-	373	bacteria>actinobacteria	Streptomyces sp. TOR3209	hypothetical protein [Streptomyces sp. TOR3209].
WP_030400958.1	-	373	bacteria>actinobacteria	Streptomyces	MULTISPECIES: hypothetical protein [Streptomyces].
WP_030418789.1	-	373	bacteria>actinobacteria	Streptomyces sp. NRRL F-5065	hypothetical protein [Streptomyces sp. NRRL F-5065].
WP_031017940.1	-	373	bacteria>actinobacteria	Streptomyces	MULTISPECIES: hypothetical protein [Streptomyces].
WP_031042032.1	-	373	bacteria>actinobacteria	Streptomyces sp. NRRL F-5650	hypothetical protein [Streptomyces sp. NRRL F-5650].
WP_033178364.1	-	373	bacteria>actinobacteria	Streptomyces sp. URHA0041	hypothetical protein [Streptomyces sp. URHA0041].
WP_037569880.1	-	373	bacteria>actinobacteria	Streptacidiphilus oryzae	hypothetical protein [Streptacidiphilus oryzae].
WP_040270705.1	-	373	bacteria>actinobacteria	Streptomonospora alba	hypothetical protein [Streptomonospora alba].
WP_043377875.1	-	373	bacteria>actinobacteria	Streptomyces mutabilis	hypothetical protein [Streptomyces mutabilis].
WP_044482941.1	-	373	bacteria>actinobacteria	Mycobacterium tuberculosis	hypothetical protein [Mycobacterium tuberculosis].
AKN73602.1	QR97_31015	373	bacteria>actinobacteria	Streptomyces sp. PBH53	hypothetical protein QR97_31015 [Streptomyces sp. PBH53].
WP_051798272.1	-	373	bacteria>actinobacteria	Streptomyces sp. NRRL S-337	hypothetical protein [Streptomyces sp. NRRL S-337].
WP_051823240.1	-	373	bacteria>actinobacteria	Streptomyces sp. NRRL S-1448	hypothetical protein [Streptomyces sp. NRRL S-1448].
WP_053139620.1	-	373	bacteria>actinobacteria	Streptomyces ambofaciens	hypothetical protein [Streptomyces ambofaciens].
WP_053914220.1	-	373	bacteria>actinobacteria	Streptomyces sp. TP-A0875	hypothetical protein [Streptomyces sp. TP-A0875].
WP_055521459.1	-	373	bacteria>actinobacteria	Streptomyces ossamyceticus	hypothetical protein [Streptomyces ossamyceticus].
WP_055538367.1	-	373	bacteria>actinobacteria	Streptomyces neyagawaensis	hypothetical protein [Streptomyces neyagawaensis].
WP_055716884.1	-	373	bacteria>actinobacteria	Streptomyces torulosus	hypothetical protein [Streptomyces torulosus].
ALM39396.1	SFR_2781	373	bacteria>actinobacteria	Streptomyces sp. FR-008	hypothetical protein SFR_2781 [Streptomyces sp. FR-008].
WP_064730645.1	-	373	bacteria>actinobacteria	Streptomyces parvulus	hypothetical protein [Streptomyces parvulus].
OBH41317.1	A5688_17975	373	bacteria>actinobacteria	Mycobacterium sp. E3121	hypothetical protein A5688_17975 [Mycobacterium sp. E3121].
OBH48743.1	A5687_15100	373	bacteria>actinobacteria	Mycobacterium sp. E2660	hypothetical protein A5687_15100 [Mycobacterium sp. E2660].
OBJ05899.1	A5625_18820	373	bacteria>actinobacteria	Mycobacterium sp. 1465703.0	hypothetical protein A5625_18820 [Mycobacterium sp. 1465703.0].
OBJ21672.1	A9W93_14130	373	bacteria>actinobacteria	Mycobacterium colombiense	hypothetical protein A9W93_14130 [Mycobacterium colombiense].
OBJ28199.1	A5621_25335	373	bacteria>actinobacteria	Mycobacterium colombiense	hypothetical protein A5621_25335 [Mycobacterium colombiense].
OBJ67055.1	A5627_03925	373	bacteria>actinobacteria	Mycobacterium colombiense	hypothetical protein A5627_03925 [Mycobacterium colombiense].
OBJ68429.1	A5626_00820	373	bacteria>actinobacteria	Mycobacterium sp. 1165549.7	hypothetical protein A5626_00820 [Mycobacterium sp. 1165549.7].
WP_064880223.1	-	373	bacteria>actinobacteria	Mycobacterium colombiense	hypothetical protein [Mycobacterium colombiense].
WP_064953935.1	-	373	bacteria>actinobacteria	Mycobacterium colombiense	hypothetical protein [Mycobacterium colombiense].
WP_065027479.1	-	373	bacteria>actinobacteria	Mycobacterium colombiense	hypothetical protein [Mycobacterium colombiense].
WP_065052714.1	-	373	bacteria>actinobacteria	Mycobacterium colombiense	hypothetical protein [Mycobacterium colombiense].
WP_065073913.1	-	373	bacteria>actinobacteria	Mycobacterium colombiense	hypothetical protein [Mycobacterium colombiense].
WP_065127784.1	-	373	bacteria>actinobacteria	Mycobacterium colombiense	hypothetical protein [Mycobacterium colombiense].
WP_003872604.1	-	372	bacteria>actinobacteria	Mycobacterium avium	hypothetical protein [Mycobacterium avium].
WP_003876968.1	-	372	bacteria>actinobacteria	Mycobacterium avium	hypothetical protein [Mycobacterium avium].
WP_007166793.1	-	372	bacteria>actinobacteria	Mycobacterium	MULTISPECIES: hypothetical protein [Mycobacterium].
WP_008263086.1	-	372	bacteria>actinobacteria	Mycobacterium	MULTISPECIES: hypothetical protein [Mycobacterium].
WP_009956756.1	-	372	bacteria>actinobacteria	Mycobacterium intracellulare	hypothetical protein [Mycobacterium intracellulare].
WP_009974499.1	-	372	bacteria>actinobacteria	Mycobacterium avium	hypothetical protein [Mycobacterium avium].
WP_011723421.1	-	372	bacteria>actinobacteria	Mycobacterium avium	hypothetical protein [Mycobacterium avium].
WP_013734391.1	-	372	bacteria>actinobacteria	Verrucosispora maris	hypothetical protein [Verrucosispora maris].
WP_014378871.1	-	372	bacteria>actinobacteria	Mycobacterium avium complex (MAC)	MULTISPECIES: hypothetical protein [Mycobacterium avium complex (MAC)].
WP_014381623.1	-	372	bacteria>actinobacteria	Mycobacterium intracellulare	hypothetical protein [Mycobacterium intracellulare].
WP_018571756.1	-	372	bacteria>actinobacteria	Streptomyces sp. PsTaAH-124	hypothetical protein [Streptomyces sp. PsTaAH-124].
WP_019734585.1	-	372	bacteria>actinobacteria	Mycobacterium avium	hypothetical protein [Mycobacterium avium].
WP_019985170.1	-	372	bacteria>actinobacteria	Streptomyces sp. Amel2xE9	hypothetical protein [Streptomyces sp. Amel2xE9].
WP_020821354.1	-	372	bacteria>actinobacteria	Mycobacterium yongonense	hypothetical protein [Mycobacterium yongonense].
WP_023862030.1	-	372	bacteria>actinobacteria	Mycobacterium avium	hypothetical protein [Mycobacterium avium].
WP_023866761.1	-	372	bacteria>actinobacteria	Mycobacterium avium	hypothetical protein [Mycobacterium avium].
WP_023868904.1	-	372	bacteria>actinobacteria	Mycobacterium avium	hypothetical protein [Mycobacterium avium].
KDE97775.1	Y900_002200	372	bacteria>actinobacteria	Mycobacterium aromaticivorans JS19b1 = JCM 16368	hypothetical protein Y900_002200 [Mycobacterium aromaticivorans JS19b1 = JCM 16368].
WP_030378339.1	-	372	bacteria>actinobacteria	Streptomyces	MULTISPECIES: hypothetical protein [Streptomyces].
WP_033319298.1	-	372	bacteria>actinobacteria	Streptomyces yerevanensis	hypothetical protein [Streptomyces yerevanensis].
WP_033710884.1	-	372	bacteria>actinobacteria	Mycobacterium avium complex (MAC)	MULTISPECIES: hypothetical protein [Mycobacterium avium complex (MAC)].
WP_033720169.1	-	372	bacteria>actinobacteria	Mycobacterium avium	hypothetical protein [Mycobacterium avium].
WP_034715430.1	-	372	bacteria>actinobacteria	Intrasporangium chromatireducens	hypothetical protein [Intrasporangium chromatireducens].
WP_037858220.1	-	372	bacteria>actinobacteria	Streptomyces sp. NRRL S-340	hypothetical protein [Streptomyces sp. NRRL S-340].
WP_037889612.1	-	372	bacteria>actinobacteria	Streptomyces sp. Tu 6176	hypothetical protein [Streptomyces sp. Tu 6176].

WP_038370766.1	-	372	bacteria>actinobacteria	Brachybacterium phenoliresistens	hypothetical protein [Brachybacterium phenoliresistens].
WP_039638559.1	-	372	bacteria>actinobacteria	Streptomyces sp. 769	hypothetical protein [Streptomyces sp. 769].
WP_041344230.1	-	372	bacteria>actinobacteria	Mycobacterium avium	hypothetical protein [Mycobacterium avium].
WP_042632772.1	-	372	bacteria>actinobacteria	Mycobacterium avium	hypothetical protein [Mycobacterium avium].
WP_043505130.1	-	372	bacteria>actinobacteria	Streptomyces glaucescens	hypothetical protein [Streptomyces glaucescens].
WP_044543376.1	-	372	bacteria>actinobacteria	Mycobacterium avium	hypothetical protein [Mycobacterium avium].
KWV31263.1	AWV63_18620	372	bacteria>actinobacteria	Micromonospora rifamycinica	peptide chain release factor 1 [Micromonospora rifamycinica].
KWX20966.1	AFM11_28275	372	bacteria>actinobacteria	Mycobacterium wolinskyi	hypothetical protein AFM11_28275 [Mycobacterium wolinskyi].
WP_062909092.1	-	372	bacteria>actinobacteria	Mycobacterium avium	hypothetical protein [Mycobacterium avium].
WP_064536109.1	-	372	bacteria>actinobacteria	Streptomyces sp. SAT1	hypothetical protein [Streptomyces sp. SAT1].
OBC02990.1	A5782_00260	372	bacteria>actinobacteria	Mycobacterium sp. 852002-40037_SCH5390672	hypothetical protein A5782_00260 [Mycobacterium sp. 852002-40037_SCH5390672].
OBE96166.1	A5775_09940	372	bacteria>actinobacteria	Mycobacterium sp. 852002-10029_SCH5224772	hypothetical protein A5775_09940 [Mycobacterium sp. 852002-10029_SCH5224772].
OBG95434.1	A5697_24555	372	bacteria>actinobacteria	Mycobacterium sp. E3251	hypothetical protein A5697_24555 [Mycobacterium sp. E3251].
OBH14327.1	A9X04_14745	372	bacteria>actinobacteria	Mycobacterium sp. E3247	hypothetical protein A9X04_14745 [Mycobacterium sp. E3247].
OBH49165.1	A5686_15640	372	bacteria>actinobacteria	Mycobacterium sp. E2479	hypothetical protein A5686_15640 [Mycobacterium sp. E2479].
OBH98455.1	A5678_22060	372	bacteria>actinobacteria	Mycobacterium sp. E2733	hypothetical protein A5678_22060 [Mycobacterium sp. E2733].
OBI18228.1	A5713_18645	372	bacteria>actinobacteria	Mycobacterium sp. E2497	hypothetical protein A5713_18645 [Mycobacterium sp. E2497].
OBJ13157.1	A5622_06455	372	bacteria>actinobacteria	Mycobacterium sp. 1245801.1	hypothetical protein A5622_06455 [Mycobacterium sp. 1245801.1].
OBJ84625.1	A9W96_27050	372	bacteria>actinobacteria	Mycobacterium sp. 1245852.3	hypothetical protein A9W96_27050 [Mycobacterium sp. 1245852.3].
OBK28137.1	A5659_06620	372	bacteria>actinobacteria	Mycobacterium sp. 1165196.3	hypothetical protein A5659_06620 [Mycobacterium sp. 1165196.3].
OBK99232.1	A5652_05625	372	bacteria>actinobacteria	Mycobacterium sp. 1165178.9	hypothetical protein A5652_05625 [Mycobacterium sp. 1165178.9].
WP_064934111.1	-	372	bacteria>actinobacteria	Mycobacterium intracellulare	hypothetical protein [Mycobacterium intracellulare].
WP_064943348.1	-	372	bacteria>actinobacteria	Mycobacterium intracellulare	hypothetical protein [Mycobacterium intracellulare].
WP_065440886.1	-	372	bacteria>actinobacteria	Mycobacterium malmoense	hypothetical protein [Mycobacterium malmoense].
WP_065474094.1	-	372	bacteria>actinobacteria	Mycobacterium malmoense	hypothetical protein [Mycobacterium malmoense].
WP_065482709.1	-	372	bacteria>actinobacteria	Mycobacterium malmoense	hypothetical protein [Mycobacterium malmoense].
WP_065496918.1	-	372	bacteria>actinobacteria	Mycobacterium malmoense	hypothetical protein [Mycobacterium malmoense].
SCF26493.1	GA0074696_3908	372	bacteria>actinobacteria	Micromonospora purpureochromogenes	hypothetical protein GA0074696_3908 [Micromonospora purpureochromogenes].
SCE78838.1	GA0070216_102114	372	bacteria>actinobacteria	Micromonospora matsumotoense	hypothetical protein GA0070216_102114 [Micromonospora matsumotoense].
SCG75009.1	GA0070614_5581	372	bacteria>actinobacteria	Micromonospora coxensis	hypothetical protein GA0070614_5581 [Micromonospora coxensis].
SCG60167.1	GA0070560_114116	372	bacteria>actinobacteria	Micromonospora halophytica	hypothetical protein GA0070560_114116 [Micromonospora halophytica].
SCG50232.1	GA0070560_106144	372	bacteria>actinobacteria	Micromonospora halophytica	hypothetical protein GA0070560_106144 [Micromonospora halophytica].
WP_030170716.1	-	371	bacteria>actinobacteria	Streptomyces sp. NRRL S-813	hypothetical protein [Streptomyces sp. NRRL S-813].
WP_030313107.1	-	371	bacteria>actinobacteria	Streptomyces sp. NRRL B-3229	hypothetical protein [Streptomyces sp. NRRL B-3229].
WP_030662242.1	-	371	bacteria>actinobacteria	Streptomyces cellulosae	hypothetical protein [Streptomyces cellulosae].
WP_031475823.1	-	371	bacteria>actinobacteria	Streptomyces bicolor	hypothetical protein [Streptomyces bicolor].
AKJ09629.1	ABB07_06235	371	bacteria>actinobacteria	Streptomyces incarnatus	hypothetical protein ABB07_06235 [Streptomyces incarnatus].
WP_055709994.1	-	371	bacteria>actinobacteria	Streptomyces puniciscabiei	hypothetical protein [Streptomyces puniciscabiei].
KXK61999.1	AWW66_10670	371	bacteria>actinobacteria	Micromonospora rosaria	peptide chain release factor 1 [Micromonospora rosaria].
WP_062021078.1	-	371	bacteria>actinobacteria	Streptomyces	MULTISPECIES: hypothetical protein [Streptomyces].
OAK56898.1	A3K89_14870	371	bacteria>actinobacteria	Rhodococcus kyotonensis	hypothetical protein A3K89_14870 [Rhodococcus kyotonensis].
OBJ69690.1	A5643_12000	371	bacteria>actinobacteria	Mycobacterium sp. 1274756.6	hypothetical protein A5643_12000 [Mycobacterium sp. 1274756.6].
WP_004980999.1	-	370	bacteria>actinobacteria	Streptomyces ghanaensis	hypothetical protein [Streptomyces ghanaensis].
WP_016823845.1	-	370	bacteria>actinobacteria	Streptomyces viridosporus	hypothetical protein [Streptomyces viridosporus].
WP_017587789.1	-	370	bacteria>actinobacteria	Nocardiopsis ganjiahuensis	hypothetical protein [Nocardiopsis ganjiahuensis].
WP_019891634.1	-	370	bacteria>actinobacteria	Streptomyces purpureus	hypothetical protein [Streptomyces purpureus].
WP_029116329.1	-	370	bacteria>actinobacteria	Mycobacterium sp. URHB0044	hypothetical protein [Mycobacterium sp. URHB0044].
WP_034277321.1	-	370	bacteria>actinobacteria	Amycolatopsis halophila	hypothetical protein [Amycolatopsis halophila].
WP_036440048.1	-	370	bacteria>actinobacteria	Mycobacterium mageritense	hypothetical protein [Mycobacterium mageritense].
OAH12804.1	STSP_38410	370	bacteria>actinobacteria	Streptomyces sp. G25(2015)	hypothetical protein STSP_38410 [Streptomyces sp. G25(2015)].
WP_006137588.1	-	369	bacteria>actinobacteria	Streptomyces griseoaurantiacus	hypothetical protein [Streptomyces griseoaurantiacus].
WP_016188039.1	-	369	bacteria>actinobacteria	Thermobifida fusca	hypothetical protein [Thermobifida fusca].
WP_016699773.1	-	369	bacteria>actinobacteria	Actinoalloteichus spitiensis	hypothetical protein [Actinoalloteichus spitiensis].
WP_017542733.1	-	369	bacteria>actinobacteria	Nocardiopsis prasina	hypothetical protein [Nocardiopsis prasina].
WP_017606189.1	-	369	bacteria>actinobacteria	Nocardiopsis alkaliphila	hypothetical protein [Nocardiopsis alkaliphila].
WP_030581102.1	-	369	bacteria>actinobacteria	Streptomyces sclerotialus	hypothetical protein [Streptomyces sclerotialus].
WP_030929580.1	-	369	bacteria>actinobacteria	Streptomyces violaceoruber	hypothetical protein [Streptomyces violaceoruber].
WP_034262461.1	-	369	bacteria>actinobacteria	Actinospica robiniae	hypothetical protein [Actinospica robiniae].
WP_041427554.1	-	369	bacteria>actinobacteria	Thermobifida fusca	hypothetical protein [Thermobifida fusca].
WP_043409131.1	-	369	bacteria>actinobacteria	Mycobacterium rufum	hypothetical protein [Mycobacterium rufum].
WP_048417514.1	-	369	bacteria>actinobacteria	Mycobacterium chubuense	hypothetical protein [Mycobacterium chubuense].
WP_049573715.1	-	369	bacteria>actinobacteria	Nocardiopsis sp. SBT366	hypothetical protein [Nocardiopsis sp. SBT366].
WP_057146979.1	-	369	bacteria>actinobacteria	Mycobacterium sp. Soil538	hypothetical protein [Mycobacterium sp. Soil538].
KWX69140.1	ASJ79_14700	369	bacteria>actinobacteria	Mycobacterium sp. NAZ190054	hypothetical protein ASJ79_14700 [Mycobacterium sp. NAZ190054].
OBG29902.1	A5764_20445	369	bacteria>actinobacteria	Mycobacterium sp. 852002-51057_SCH5723018	hypothetical protein A5764_20445 [Mycobacterium sp. 852002-51057_SCH5723018].
WP_003996530.1	-	368	bacteria>actinobacteria	Streptomyces viridochromogenes	hypothetical protein [Streptomyces viridochromogenes].
WP_010550185.1	-	368	bacteria>actinobacteria	Brachybacterium paraconglomeratum	hypothetical protein [Brachybacterium paraconglomeratum].
WP_015776244.1	-	368	bacteria>actinobacteria	Brachybacterium faecium	hypothetical protein [Brachybacterium faecium].
WP_017574508.1	-	368	bacteria>actinobacteria	Nocardiopsis kunsanensis	hypothetical protein [Nocardiopsis kunsanensis].
WP_026818744.1	-	368	bacteria>actinobacteria	Arthrobacter castelli	hypothetical protein [Arthrobacter castelli].
WP_030047992.1	-	368	bacteria>actinobacteria	Streptomyces peruviensis	hypothetical protein [Streptomyces peruviensis].
WP_031075080.1	-	368	bacteria>actinobacteria	Streptomyces sp. NRRL S-118	hypothetical protein [Streptomyces sp. NRRL S-118].
WP_036362577.1	-	368	bacteria>actinobacteria	Mycobacterium asiaticum	hypothetical protein [Mycobacterium asiaticum].
WP_036454369.1	-	368	bacteria>actinobacteria	Mycobacterium sp. TKK-01-0059	hypothetical protein [Mycobacterium sp. TKK-01-0059].
WP_037307873.1	-	368	bacteria>actinobacteria	Amycolatopsis orientalis	hypothetical protein [Amycolatopsis orientalis].
WP_040276034.1	-	368	bacteria>actinobacteria	Streptomonospora alba	hypothetical protein [Streptomonospora alba].
WP_042910410.1	-	368	bacteria>actinobacteria	Mycobacterium avium complex (MAC)	MULTISPECIES: hypothetical protein [Mycobacterium avium complex (MAC)].
WP_044508986.1	-	368	bacteria>actinobacteria	Mycobacterium simiae	hypothetical protein [Mycobacterium simiae].
WP_045375262.1	-	368	bacteria>actinobacteria	Mycobacterium kyorinense	hypothetical protein [Mycobacterium kyorinense].
WP_048579414.1	-	368	bacteria>actinobacteria	Streptomyces viridochromogenes	hypothetical protein [Streptomyces viridochromogenes].
WP_053918520.1	-	368	bacteria>actinobacteria	Brachybacterium sp. SW0106-09	hypothetical protein [Brachybacterium sp. SW0106-09].
WP_055493800.1	-	368	bacteria>actinobacteria	Streptomyces sp. TP-A0356	hypothetical protein [Streptomyces sp. TP-A0356].
WP_055576956.1	-	368	bacteria>actinobacteria	Mycobacterium gordonae	hypothetical protein [Mycobacterium gordonae].
WP_058922128.1	-	368	bacteria>actinobacteria	Streptomyces sp. CdTB01	hypothetical protein [Streptomyces sp. CdTB01].
WP_059129380.1	-	368	bacteria>actinobacteria	Streptomyces sp. NRRL F-5122	hypothetical protein [Streptomyces sp. NRRL F-5122].
KUO20003.1	AQJ91_16955	368	bacteria>actinobacteria	Streptomyces sp. RV15	hypothetical protein AQJ91_16955 [Streptomyces sp. RV15].
WP_059195284.1	-	368	bacteria>actinobacteria	Streptomyces antibioticus	hypothetical protein [Streptomyces antibioticus].
KUP96522.1	AC529_11730	368	bacteria>actinobacteria	Thermobifida cellulosilytica TB100	hypothetical protein AC529_11730 [Thermobifida cellulosilytica TB100].
WP_061557582.1	-	368	bacteria>actinobacteria	Mycobacterium simiae	hypothetical protein [Mycobacterium simiae].
WP_062147704.1	-	368	bacteria>actinobacteria	Streptomyces curacoi	hypothetical protein [Streptomyces curacoi].
WP_062540664.1	-	368	bacteria>actinobacteria	Mycobacterium celatum	hypothetical protein [Mycobacterium celatum].
WP_062699814.1	-	368	bacteria>actinobacteria	Streptomyces regalis	hypothetical protein [Streptomyces regalis].
WP_062716495.1	-	368	bacteria>actinobacteria	Streptomyces caeruleatus	hypothetical protein [Streptomyces caeruleatus].
WP_062827162.1	-	368	bacteria>actinobacteria	Mycobacterium brisbanense	hypothetical protein [Mycobacterium brisbanense].
WP_062947752.1	-	368	bacteria>actinobacteria	Brachybacterium sp. sponge	hypothetical protein [Brachybacterium sp. sponge].
KZS57905.1	A4G28_12855	368	bacteria>actinobacteria	Mycobacterium kansasii	hypothetical protein A4G28_12855 [Mycobacterium kansasii].
OBA61101.1	A5647_11875	368	bacteria>actinobacteria	Mycobacterium sp. 1100029.7	hypothetical protein A5647_11875 [Mycobacterium sp. 1100029.7].
OBA69089.1	A5641_03855	368	bacteria>actinobacteria	Mycobacterium sp. 1554424.7	hypothetical protein A5641_03855 [Mycobacterium sp. 1554424.7].
OBA83305.1	A9W99_08250	368	bacteria>actinobacteria	Mycobacterium sp. 1164966.3	hypothetical protein A9W99_08250 [Mycobacterium sp. 1164966.3].
OBF15420.1	A5725_04280	368	bacteria>actinobacteria	Mycobacterium szulgai	hypothetical protein A5725_04280 [Mycobacterium szulgai].
OBG35690.1	A5673_19745	368	bacteria>actinobacteria	Mycobacterium sp. E3198	hypothetical protein A5673_19745 [Mycobacterium sp. E3198].
OBG61063.1	A5703_23880	368	bacteria>actinobacteria	Mycobacterium sp. E188	hypothetical protein A5703_23880 [Mycobacterium sp. E188].
OBG65827.1	A5704_11690	368	bacteria>actinobacteria	Mycobacterium sp. E735	hypothetical protein A5704_11690 [Mycobacterium sp. E735].
OBG72049.1	A5700_00735	368	bacteria>actinobacteria	Mycobacterium sp. E1214	hypothetical protein A5700_00735 [Mycobacterium sp. E1214].
OBG93386.1	A9X05_09710	368	bacteria>actinobacteria	Mycobacterium sp. E3298	hypothetical protein A9X05_09710 [Mycobacterium sp. E3298].
OBH07298.1	A5695_03430	368	bacteria>actinobacteria	Mycobacterium sp. E1747	hypothetical protein A5695_03430 [Mycobacterium sp. E1747].
OBH87821.1	A5681_13015	368	bacteria>actinobacteria	Mycobacterium scrofulaceum	hypothetical protein A5681_13015 [Mycobacterium scrofulaceum].
OBI07822.1	A5679_10300	368	bacteria>actinobacteria	Mycobacterium scrofulaceum	hypothetical protein A5679_10300 [Mycobacterium scrofulaceum].
OBI21083.1	A5712_16275	368	bacteria>actinobacteria	Mycobacterium sp. E2327	hypothetical protein A5712_16275 [Mycobacterium sp. E2327].
OBI48598.1	A5706_27355	368	bacteria>actinobacteria	Mycobacterium sp. E796	hypothetical protein A5706_27355 [Mycobacterium sp. E796].
OBI74769.1	A5663_05010	368	bacteria>actinobacteria	Mycobacterium sp. E740	hypothetical protein A5663_05010 [Mycobacterium sp. E740].
OBI86710.1	A9X00_25600	368	bacteria>actinobacteria	Mycobacterium sp. 1245805.9	hypothetical protein A9X00_25600 [Mycobacterium sp. 1245805.9].
OBJ61507.1	A9W95_09390	368	bacteria>actinobacteria	Mycobacterium sp. 1423905.2	hypothetical protein A9W95_09390 [Mycobacterium sp. 1423905.2].
OBK47362.1	A5655_07035	368	bacteria>actinobacteria	Mycobacterium sp. 1081908.1	hypothetical protein A5655_07035 [Mycobacterium sp. 1081908.1].
OBK54504.1	A5657_13180	368	bacteria>actinobacteria	Mycobacterium szulgai	hypothetical protein A5657_13180 [Mycobacterium szulgai].
WP_064921773.1	-	368	bacteria>actinobacteria	Mycobacterium sinense	hypothetical protein [Mycobacterium sinense].
WP_065015758.1	-	368	bacteria>actinobacteria	Mycobacterium kyorinense	hypothetical protein [Mycobacterium kyorinense].
WP_065026196.1	-	368	bacteria>actinobacteria	Mycobacterium sinense	hypothetical protein [Mycobacterium sinense].
WP_065034017.1	-	368	bacteria>actinobacteria	Mycobacterium asiaticum	hypothetical protein [Mycobacterium asiaticum].
WP_065045892.1	-	368	bacteria>actinobacteria	Mycobacterium gordonae	hypothetical protein [Mycobacterium gordonae].
WP_065120385.1	-	368	bacteria>actinobacteria	Mycobacterium asiaticum	hypothetical protein [Mycobacterium asiaticum].
WP_065128133.1	-	368	bacteria>actinobacteria	Mycobacterium asiaticum	hypothetical protein [Mycobacterium asiaticum].
WP_065137681.1	-	368	bacteria>actinobacteria	Mycobacterium gordonae	hypothetical protein [Mycobacterium gordonae].
WP_065138456.1	-	368	bacteria>actinobacteria	Mycobacterium asiaticum	hypothetical protein [Mycobacterium asiaticum].
WP_065143616.1	-	368	bacteria>actinobacteria	Mycobacterium asiaticum	hypothetical protein [Mycobacterium asiaticum].
WP_065150833.1	-	368	bacteria>actinobacteria	Mycobacterium asiaticum	hypothetical protein [Mycobacterium asiaticum].
WP_065154567.1	-	368	bacteria>actinobacteria	Mycobacterium asiaticum	hypothetical protein [Mycobacterium asiaticum].
WP_065158233.1	-	368	bacteria>actinobacteria	Mycobacterium asiaticum	hypothetical protein [Mycobacterium asiaticum].
WP_065498710.1	-	368	bacteria>actinobacteria	Mycobacterium yongonense	hypothetical protein [Mycobacterium yongonense].
WP_014058256.1	-	367	bacteria>actinobacteria	Streptomyces violaceusniger	hypothetical protein [Streptomyces violaceusniger].
WP_017607208.1	-	367	bacteria>actinobacteria	Nocardiopsis xinjiangensis	hypothetical protein [Nocardiopsis xinjiangensis].
WP_017613145.1	-	367	bacteria>actinobacteria	Nocardiopsis salina	hypothetical protein [Nocardiopsis salina].
WP_019971536.1	-	367	bacteria>actinobacteria	Mycobacterium sp. 141	hypothetical protein [Mycobacterium sp. 141].
WP_020119161.1	-	367	bacteria>actinobacteria	Streptomyces canus	hypothetical protein [Streptomyces canus].
WP_029367397.1	-	367	bacteria>actinobacteria	Mycobacterium sp. UM_WWY	hypothetical protein [Mycobacterium sp. UM_WWY].
WP_030170792.1	-	367	bacteria>actinobacteria	Spirillospora albida	hypothetical protein [Spirillospora albida].
WP_030293261.1	-	367	bacteria>actinobacteria	Streptomyces katrae	hypothetical protein [Streptomyces katrae].
WP_031043272.1	-	367	bacteria>actinobacteria	Streptomyces	MULTISPECIES: hypothetical protein [Streptomyces].
WP_033431382.1	-	367	bacteria>actinobacteria	Saccharothrix syringae	hypothetical protein [Saccharothrix syringae].
WP_037673673.1	-	367	bacteria>actinobacteria	Streptomyces griseus	hypothetical protein [Streptomyces griseus].
WP_037913532.1	-	367	bacteria>actinobacteria	Streptomyces yeochonensis	hypothetical protein [Streptomyces yeochonensis].
WP_037965719.1	-	367	bacteria>actinobacteria	Streptomyces sp. PRh5	hypothetical protein [Streptomyces sp. PRh5].
WP_039657309.1	-	367	bacteria>actinobacteria	Streptomyces	MULTISPECIES: hypothetical protein [Streptomyces].
WP_045875939.1	-	367	bacteria>actinobacteria	Frankia sp. DC12	hypothetical protein [Frankia sp. DC12].
WP_046282947.1	-	367	bacteria>actinobacteria	Mycobacterium	MULTISPECIES: hypothetical protein [Mycobacterium].
WP_047333345.1	-	367	bacteria>actinobacteria	Mycobacterium sp. EPa45	hypothetical protein [Mycobacterium sp. EPa45].
WP_053790568.1	-	367	bacteria>actinobacteria	Streptomyces sp. XY332	hypothetical protein [Streptomyces sp. XY332].
WP_053850370.1	-	367	bacteria>actinobacteria	Streptomyces sp. NRRL B-24085	hypothetical protein [Streptomyces sp. NRRL B-24085].
WP_057608224.1	-	367	bacteria>actinobacteria	Streptomyces sp. Root369	hypothetical protein [Streptomyces sp. Root369].
WP_058079675.1	-	367	bacteria>actinobacteria	Streptomyces hygroscopicus	hypothetical protein [Streptomyces hygroscopicus].
KUO19287.1	AQJ91_20165	367	bacteria>actinobacteria	Streptomyces sp. RV15	hypothetical protein AQJ91_20165 [Streptomyces sp. RV15].
WP_059209536.1	-	367	bacteria>actinobacteria	Streptomyces canus	hypothetical protein [Streptomyces canus].
WP_062036942.1	-	367	bacteria>actinobacteria	Streptomyces ciscaucasicus	hypothetical protein [Streptomyces ciscaucasicus].
WP_062829847.1	-	367	bacteria>actinobacteria	Mycobacterium brisbanense	hypothetical protein [Mycobacterium brisbanense].
OBH08050.1	A5696_20580	367	bacteria>actinobacteria	Mycobacterium sp. E2699	hypothetical protein A5696_20580 [Mycobacterium sp. E2699].
OBI52771.1	A5705_05160	367	bacteria>actinobacteria	Mycobacterium sp. E787	hypothetical protein A5705_05160 [Mycobacterium sp. E787].
WP_064890789.1	-	367	bacteria>actinobacteria	Mycobacterium heraklionense	hypothetical protein [Mycobacterium heraklionense].
WP_064949300.1	-	367	bacteria>actinobacteria	Mycobacterium sp. 852013-50091_SCH5140682	hypothetical protein [Mycobacterium sp. 852013-50091_SCH5140682].
WP_003994127.1	-	366	bacteria>actinobacteria	Streptomyces viridochromogenes	hypothetical protein [Streptomyces viridochromogenes].
WP_005146890.1	-	366	bacteria>actinobacteria	Mycobacterium rhodesiae	hypothetical protein [Mycobacterium rhodesiae].
WP_005452806.1	-	366	bacteria>actinobacteria	Saccharomonospora cyanea	hypothetical protein [Saccharomonospora cyanea].
WP_005461035.1	-	366	bacteria>actinobacteria	Saccharomonospora glauca	hypothetical protein [Saccharomonospora glauca].
WP_006238250.1	-	366	bacteria>actinobacteria	Saccharomonospora xinjiangensis	hypothetical protein [Saccharomonospora xinjiangensis].
WP_011560138.1	-	366	bacteria>actinobacteria	Mycobacterium	MULTISPECIES: hypothetical protein [Mycobacterium].
WP_012795701.1	-	366	bacteria>actinobacteria	Saccharomonospora viridis	peptide chain release factor 2 [Saccharomonospora viridis].
WP_013829985.1	-	366	bacteria>actinobacteria	Mycobacterium sinense	hypothetical protein [Mycobacterium sinense].
WP_014448008.1	-	366	bacteria>actinobacteria	Actinoplanes missouriensis	hypothetical protein [Actinoplanes missouriensis].
WP_016467676.1	-	366	bacteria>actinobacteria	Streptomyces	MULTISPECIES: hypothetical protein [Streptomyces].
WP_019818604.1	-	366	bacteria>actinobacteria	Saccharomonospora saliphila	hypothetical protein [Saccharomonospora saliphila].
WP_020270854.1	-	366	bacteria>actinobacteria	Streptomyces afghaniensis	hypothetical protein [Streptomyces afghaniensis].
WP_026404369.1	-	366	bacteria>actinobacteria	Actinomadura rifamycini	hypothetical protein [Actinomadura rifamycini].
WP_028801198.1	-	366	bacteria>actinobacteria	Streptomyces sp. 142MFCol3.1	hypothetical protein [Streptomyces sp. 142MFCol3.1].
WP_030406835.1	-	366	bacteria>actinobacteria	Streptomyces sp. NRRL F-5917	hypothetical protein [Streptomyces sp. NRRL F-5917].
WP_031025567.1	-	366	bacteria>actinobacteria	Streptomyces sp. NRRL F-5639	hypothetical protein [Streptomyces sp. NRRL F-5639].
WP_031109775.1	-	366	bacteria>actinobacteria	Streptomyces sp. NRRL S-146	hypothetical protein [Streptomyces sp. NRRL S-146].
WP_031136391.1	-	366	bacteria>actinobacteria	Streptomyces	MULTISPECIES: hypothetical protein [Streptomyces].
WP_037606115.1	-	366	bacteria>actinobacteria	Streptacidiphilus rugosus	hypothetical protein [Streptacidiphilus rugosus].
WP_037611842.1	-	366	bacteria>actinobacteria	Streptomyces albus	hypothetical protein [Streptomyces albus].
WP_037759670.1	-	366	bacteria>actinobacteria	Streptomyces olindensis	hypothetical protein [Streptomyces olindensis].
WP_043907562.1	-	366	bacteria>actinobacteria	Kitasatospora griseola	hypothetical protein [Kitasatospora griseola].
WP_045947269.1	-	366	bacteria>actinobacteria	Streptomyces katrae	hypothetical protein [Streptomyces katrae].
WP_047331867.1	-	366	bacteria>actinobacteria	Mycobacterium sp. EPa45	hypothetical protein [Mycobacterium sp. EPa45].
CRL72273.1	CPGR_02268	366	bacteria>actinobacteria	Mycobacterium sp. WCM 7299	hypothetical protein CPGR_02268 [Mycobacterium sp. WCM 7299].
WP_053673819.1	-	366	bacteria>actinobacteria	Streptomyces sp. NRRL B-1140	hypothetical protein [Streptomyces sp. NRRL B-1140].
WP_055498197.1	-	366	bacteria>actinobacteria	Streptomyces flocculus	hypothetical protein [Streptomyces flocculus].
WP_058079662.1	-	366	bacteria>actinobacteria	Streptomyces hygroscopicus	hypothetical protein [Streptomyces hygroscopicus].
WP_059089534.1	-	366	bacteria>actinobacteria	Mycobacterium sp. GA-2829	hypothetical protein [Mycobacterium sp. GA-2829].
WP_059096341.1	-	366	bacteria>actinobacteria	Mycobacterium sp. IS-1742	hypothetical protein [Mycobacterium sp. IS-1742].
WP_059098491.1	-	366	bacteria>actinobacteria	Mycobacterium	MULTISPECIES: hypothetical protein [Mycobacterium].
WP_059158013.1	-	366	bacteria>actinobacteria	Mycobacterium sp. IS-1496	hypothetical protein [Mycobacterium sp. IS-1496].
WP_059165883.1	-	366	bacteria>actinobacteria	Mycobacterium sp. IS-3022	hypothetical protein [Mycobacterium sp. IS-3022].
WP_060894940.1	-	366	bacteria>actinobacteria	Streptomyces diastatochromogenes	hypothetical protein [Streptomyces diastatochromogenes].
GAT10692.1	RMCN_3825	366	bacteria>actinobacteria	Mycobacterium novocastrense	uncharacterized protein RMCN_3825 [Mycobacterium novocastrense].
WP_062394770.1	-	366	bacteria>actinobacteria	Pseudonocardia sp. AL041005-10	hypothetical protein [Pseudonocardia sp. AL041005-10].
WP_063473095.1	-	366	bacteria>actinobacteria	Mycobacterium kansasii	hypothetical protein [Mycobacterium kansasii].
WP_064346341.1	-	366	bacteria>actinobacteria	Mycobacterium sp. GA-1199	hypothetical protein [Mycobacterium sp. GA-1199].
WP_064393763.1	-	366	bacteria>actinobacteria	Mycobacterium sp. IS-1744	hypothetical protein [Mycobacterium sp. IS-1744].
WP_064401787.1	-	366	bacteria>actinobacteria	Mycobacterium sp. IS-1590	hypothetical protein [Mycobacterium sp. IS-1590].
WP_064413157.1	-	366	bacteria>actinobacteria	Mycobacterium	MULTISPECIES: hypothetical protein [Mycobacterium].
WP_064418625.1	-	366	bacteria>actinobacteria	Mycobacterium sp. GA-1683	hypothetical protein [Mycobacterium sp. GA-1683].
WP_064421006.1	-	366	bacteria>actinobacteria	Mycobacterium sp. GA-1285	hypothetical protein [Mycobacterium sp. GA-1285].
WP_064425497.1	-	366	bacteria>actinobacteria	Mycobacterium sp. GA-1331	hypothetical protein [Mycobacterium sp. GA-1331].
WP_064427735.1	-	366	bacteria>actinobacteria	Mycobacterium sp. GA-1379	hypothetical protein [Mycobacterium sp. GA-1379].
OBB46611.1	A5752_01720	366	bacteria>actinobacteria	Mycobacterium sp. 852002-51961_SCH5331710	hypothetical protein A5752_01720 [Mycobacterium sp. 852002-51961_SCH5331710].
OBB62039.1	A5757_05460	366	bacteria>actinobacteria	Mycobacterium sp. 852013-51886_SCH5428379	hypothetical protein A5757_05460 [Mycobacterium sp. 852013-51886_SCH5428379].
OBB70814.1	A5759_24215	366	bacteria>actinobacteria	Mycobacterium sp. 852014-52144_SCH5372336	hypothetical protein A5759_24215 [Mycobacterium sp. 852014-52144_SCH5372336].
OBF93099.1	A5790_11860	366	bacteria>actinobacteria	Mycobacterium sp. 852002-51152_SCH6134967	hypothetical protein A5790_11860 [Mycobacterium sp. 852002-51152_SCH6134967].
OBG14511.1	A5765_11185	366	bacteria>actinobacteria	Mycobacterium sp. 852002-51296_SCH5728562-a	hypothetical protein A5765_11185 [Mycobacterium sp. 852002-51296_SCH5728562-a].
OBG99493.1	A5698_01125	366	bacteria>actinobacteria	Mycobacterium sp. E136	hypothetical protein A5698_01125 [Mycobacterium sp. E136].
OBK73888.1	A5650_19670	366	bacteria>actinobacteria	Mycobacterium sp. 1164985.4	hypothetical protein A5650_19670 [Mycobacterium sp. 1164985.4].
WP_064875206.1	-	366	bacteria>actinobacteria	Mycobacterium sp. 852013-50142_SCH4511227	hypothetical protein [Mycobacterium sp. 852013-50142_SCH4511227].
WP_064916318.1	-	366	bacteria>actinobacteria	Mycobacterium sp. 852002-10379_SCH5584320	hypothetical protein [Mycobacterium sp. 852002-10379_SCH5584320].
WP_005442875.1	-	365	bacteria>actinobacteria	Saccharomonospora azurea	hypothetical protein [Saccharomonospora azurea].
WP_005450557.1	-	365	bacteria>actinobacteria	Saccharomonospora azurea	hypothetical protein [Saccharomonospora azurea].
WP_006243563.1	-	365	bacteria>actinobacteria	Mycobacterium tusciae	hypothetical protein [Mycobacterium tusciae].
WP_007023642.1	-	365	bacteria>actinobacteria	Saccharomonospora paurometabolica	hypothetical protein [Saccharomonospora paurometabolica].
WP_009152097.1	-	365	bacteria>actinobacteria	Saccharomonospora marina	hypothetical protein [Saccharomonospora marina].
WP_019811566.1	-	365	bacteria>actinobacteria	Saccharomonospora halophila	peptide chain release factor 2 [Saccharomonospora halophila].
WP_020626522.1	-	365	bacteria>actinobacteria	Pseudonocardia sp. P2	hypothetical protein [Pseudonocardia sp. P2].
WP_021595710.1	-	365	bacteria>actinobacteria	Actinomadura madurae	hypothetical protein [Actinomadura madurae].
WP_023552684.1	-	365	bacteria>actinobacteria	Streptomyces roseochromogenus	hypothetical protein [Streptomyces roseochromogenus].
WP_026416265.1	-	365	bacteria>actinobacteria	Actinomadura oligospora	hypothetical protein [Actinomadura oligospora].
WP_026453488.1	-	365	bacteria>actinobacteria	[Actinopolyspora] iraqiensis	peptide chain release factor 2 [[Actinopolyspora] iraqiensis].
WP_030319950.1	-	365	bacteria>actinobacteria	Streptomyces flavochromogenes	hypothetical protein [Streptomyces flavochromogenes].
WP_030475805.1	-	365	bacteria>actinobacteria	Lentzea albidocapillata	hypothetical protein [Lentzea albidocapillata].
WP_030843797.1	-	365	bacteria>actinobacteria	Streptomyces sp. NRRL S-475	hypothetical protein [Streptomyces sp. NRRL S-475].
WP_030916653.1	-	365	bacteria>actinobacteria	Streptomyces sp. NRRL B-24720	hypothetical protein [Streptomyces sp. NRRL B-24720].
WP_030971630.1	-	365	bacteria>actinobacteria	Streptomyces sp. NRRL S-1824	hypothetical protein [Streptomyces sp. NRRL S-1824].
WP_031160398.1	-	365	bacteria>actinobacteria	Streptomyces durhamensis	hypothetical protein [Streptomyces durhamensis].
WP_037255429.1	-	365	bacteria>actinobacteria	Rhodococcus rhodnii	hypothetical protein [Rhodococcus rhodnii].
WP_037345159.1	-	365	bacteria>actinobacteria	Amycolatopsis sp. MJM2582	hypothetical protein [Amycolatopsis sp. MJM2582].
WP_038510862.1	-	365	bacteria>actinobacteria	Amycolatopsis japonica	hypothetical protein [Amycolatopsis japonica].
WP_041133022.1	-	365	bacteria>actinobacteria	Streptomyces vietnamensis	hypothetical protein [Streptomyces vietnamensis].
WP_042573641.1	-	365	bacteria>actinobacteria	Rhodococcus sp. MEB064	hypothetical protein [Rhodococcus sp. MEB064].
WP_043848758.1	-	365	bacteria>actinobacteria	Amycolatopsis orientalis	hypothetical protein [Amycolatopsis orientalis].
WP_051021811.1	-	365	bacteria>actinobacteria	Nocardia araoensis	hypothetical protein [Nocardia araoensis].
WP_053718843.1	-	365	bacteria>actinobacteria	Saccharothrix sp. NRRL B-16348	hypothetical protein [Saccharothrix sp. NRRL B-16348].
WP_053927296.1	-	365	bacteria>actinobacteria	Streptomyces chattanoogensis	hypothetical protein [Streptomyces chattanoogensis].
WP_056069780.1	-	365	bacteria>actinobacteria	Rhodococcus	MULTISPECIES: hypothetical protein [Rhodococcus].
KUJ69405.1	ACZ90_10915	365	bacteria>actinobacteria	Streptomyces albus subsp. albus	hypothetical protein ACZ90_10915 [Streptomyces albus subsp. albus].
KUM93204.1	AQI88_27910	365	bacteria>actinobacteria	Streptomyces cellostaticus	hypothetical protein AQI88_27910 [Streptomyces cellostaticus].
WP_059420081.1	-	365	bacteria>actinobacteria	Streptomyces azureus	hypothetical protein [Streptomyces azureus].
WP_060572110.1	-	365	bacteria>actinobacteria	Pseudonocardia	MULTISPECIES: hypothetical protein [Pseudonocardia].
WP_062699484.1	-	365	bacteria>actinobacteria	Streptomyces regalis	hypothetical protein [Streptomyces regalis].
WP_063823081.1	-	365	bacteria>actinobacteria	Amycolatopsis keratiniphila	hypothetical protein [Amycolatopsis keratiniphila].
OBF33548.1	A5724_18385	365	bacteria>actinobacteria	Mycobacterium sp. ACS1612	hypothetical protein A5724_18385 [Mycobacterium sp. ACS1612].
ANP51786.1	AVL59_21300	365	bacteria>actinobacteria	Streptomyces griseochromogenes	hypothetical protein AVL59_21300 [Streptomyces griseochromogenes].
ANS69154.1	SLINC_6930	365	bacteria>actinobacteria	Streptomyces lincolnensis	hypothetical protein SLINC_6930 [Streptomyces lincolnensis].
SCF59663.1	GA0115254_106217	365	bacteria>actinobacteria	Streptomyces sp. Ncost-T10-10d	hypothetical protein GA0115254_106217 [Streptomyces sp. Ncost-T10-10d].
WP_005321798.1	-	364	bacteria>actinobacteria	Streptomyces pristinaespiralis	hypothetical protein [Streptomyces pristinaespiralis].
WP_018656762.1	-	364	bacteria>actinobacteria	Actinomadura flavalba	hypothetical protein [Actinomadura flavalba].
WP_033096778.1	-	364	bacteria>actinobacteria	Rhodococcus sp. p52	hypothetical protein [Rhodococcus sp. p52].
WP_037603540.1	-	364	bacteria>actinobacteria	Streptacidiphilus rugosus	hypothetical protein [Streptacidiphilus rugosus].
WP_041803553.1	-	364	bacteria>actinobacteria	Rhodococcus pyridinivorans	hypothetical protein [Rhodococcus pyridinivorans].
WP_042381043.1	-	364	bacteria>actinobacteria	Streptacidiphilus melanogenes	hypothetical protein [Streptacidiphilus melanogenes].
WP_042418190.1	-	364	bacteria>actinobacteria	Streptacidiphilus anmyonensis	hypothetical protein [Streptacidiphilus anmyonensis].
WP_064255907.1	-	364	bacteria>actinobacteria	Rhodococcus sp. HS-D2	hypothetical protein [Rhodococcus sp. HS-D2].
WP_003934834.1	-	363	bacteria>actinobacteria	Rhodococcus ruber	hypothetical protein [Rhodococcus ruber].
WP_006601783.1	-	363	bacteria>actinobacteria	Streptomyces auratus	hypothetical protein [Streptomyces auratus].
WP_009077582.1	-	363	bacteria>actinobacteria	Streptomyces sp. AA4	hypothetical protein [Streptomyces sp. AA4].
WP_017680786.1	-	363	bacteria>actinobacteria	Rhodococcus ruber	hypothetical protein [Rhodococcus ruber].
WP_020659597.1	-	363	bacteria>actinobacteria	Amycolatopsis benzoatilytica	hypothetical protein [Amycolatopsis benzoatilytica].
WP_026341370.1	-	363	bacteria>actinobacteria	Actinomadura atramentaria	hypothetical protein [Actinomadura atramentaria].
WP_030282185.1	-	363	bacteria>actinobacteria	Streptomyces catenulae	hypothetical protein [Streptomyces catenulae].
WP_030435049.1	-	363	bacteria>actinobacteria	Actinoplanes subtropicus	hypothetical protein [Actinoplanes subtropicus].
WP_037370567.1	-	363	bacteria>actinobacteria	Amycolatopsis orientalis	hypothetical protein [Amycolatopsis orientalis].
WP_037812958.1	-	363	bacteria>actinobacteria	Streptomyces sp. NRRL F-3213	hypothetical protein [Streptomyces sp. NRRL F-3213].
WP_040274798.1	-	363	bacteria>actinobacteria	Rhodococcus	MULTISPECIES: hypothetical protein [Rhodococcus].
WP_043840100.1	-	363	bacteria>actinobacteria	Amycolatopsis orientalis	hypothetical protein [Amycolatopsis orientalis].
WP_054372529.1	-	363	bacteria>actinobacteria	Rhodococcus rhodochrous	hypothetical protein [Rhodococcus rhodochrous].
WP_059017310.1	-	363	bacteria>actinobacteria	Mycobacterium sp. M26	hypothetical protein [Mycobacterium sp. M26].
OAH14590.1	STSP_21010	363	bacteria>actinobacteria	Streptomyces sp. G25(2015)	hypothetical protein STSP_21010 [Streptomyces sp. G25(2015)].
OAP21377.1	A4R44_07895	363	bacteria>actinobacteria	Amycolatopsis sp. M39	hypothetical protein A4R44_07895 [Amycolatopsis sp. M39].
WP_033437138.1	-	362	bacteria>actinobacteria	Saccharothrix sp. NRRL B-16314	hypothetical protein [Saccharothrix sp. NRRL B-16314].
WP_051169669.1	-	362	bacteria>actinobacteria	Nocardia abscessus	hypothetical protein [Nocardia abscessus].
WP_062699705.1	-	362	bacteria>actinobacteria	Streptomyces regalis	hypothetical protein [Streptomyces regalis].
AAZ54198.1	Tfu_0160	361	bacteria>actinobacteria	Thermobifida fusca YX	conserved hypothetical protein [Thermobifida fusca YX].
WP_003091307.1	-	361	bacteria>actinobacteria	Amycolatopsis vancoresmycina	hypothetical protein [Amycolatopsis vancoresmycina].
WP_043445145.1	-	361	bacteria>actinobacteria	Streptomyces nodosus	hypothetical protein [Streptomyces nodosus].
WP_043739303.1	-	361	bacteria>actinobacteria	Nocardia asiatica	hypothetical protein [Nocardia asiatica].
WP_043779820.1	-	361	bacteria>actinobacteria	Amycolatopsis rifamycinica	hypothetical protein [Amycolatopsis rifamycinica].
WP_043910339.1	-	361	bacteria>actinobacteria	Kitasatospora griseola	hypothetical protein [Kitasatospora griseola].
WP_049716073.1	-	361	bacteria>actinobacteria	Streptomyces caatingaensis	hypothetical protein [Streptomyces caatingaensis].
WP_050768020.1	-	361	bacteria>actinobacteria	Nocardia farcinica	hypothetical protein [Nocardia farcinica].
WP_012854486.1	-	360	bacteria>actinobacteria	Thermomonospora curvata	hypothetical protein [Thermomonospora curvata].
WP_019630422.1	-	360	bacteria>actinobacteria	Actinomadura atramentaria	hypothetical protein [Actinomadura atramentaria].
WP_024804604.1	-	358	bacteria>actinobacteria	Nocardia sp. BMG51109	hypothetical protein [Nocardia sp. BMG51109].
WP_040787105.1	-	358	bacteria>actinobacteria	Nocardia pneumoniae	hypothetical protein [Nocardia pneumoniae].
WP_063020766.1	-	358	bacteria>actinobacteria	Nocardia niwae	hypothetical protein [Nocardia niwae].
WP_063049911.1	-	358	bacteria>actinobacteria	Nocardia arthritidis	hypothetical protein [Nocardia arthritidis].
WP_063910399.1	-	358	bacteria>actinobacteria	Nocardia gamkensis	hypothetical protein [Nocardia gamkensis].
WP_043704183.1	-	357	bacteria>actinobacteria	Nocardia abscessus	hypothetical protein [Nocardia abscessus].
GAT70919.1	PS9374_06608	357	bacteria>actinobacteria	Planomonospora sphaerica	peptide chain release factor 1 [Planomonospora sphaerica].
WP_013132801.1	-	356	bacteria>actinobacteria	Thermobispora bispora	hypothetical protein [Thermobispora bispora].
EUA88292.1	I551_5228	356	bacteria>actinobacteria	Mycobacterium ulcerans str. Harvey	hypothetical protein I551_5228 [Mycobacterium ulcerans str. Harvey].
WP_040866310.1	-	356	bacteria>actinobacteria	Nocardia exalbida	hypothetical protein [Nocardia exalbida].
WP_020672853.1	-	355	bacteria>actinobacteria	Amycolatopsis nigrescens	hypothetical protein [Amycolatopsis nigrescens].
WP_010231117.1	-	354	bacteria>actinobacteria	Pseudonocardia sp. P1	hypothetical protein, partial [Pseudonocardia sp. P1].
OBK36601.1	A5658_06005	354	bacteria>actinobacteria	Mycobacterium sp. 1245111.1	hypothetical protein A5658_06005 [Mycobacterium sp. 1245111.1].
WP_013491520.1	-	352	bacteria>actinobacteria	Intrasporangium calvum	hypothetical protein [Intrasporangium calvum].
WP_017986242.1	-	351	bacteria>actinobacteria	Amycolatopsis methanolica	hypothetical protein [Amycolatopsis methanolica].
WP_020416495.1	-	351	bacteria>actinobacteria	Amycolatopsis sp. ATCC 39116	hypothetical protein [Amycolatopsis sp. ATCC 39116].
WP_027932947.1	-	351	bacteria>actinobacteria	Amycolatopsis thermoflava	hypothetical protein [Amycolatopsis thermoflava].
WP_051659833.1	-	349	bacteria>actinobacteria	Mycobacterium aromaticivorans	hypothetical protein [Mycobacterium aromaticivorans].
WP_025358023.1	-	347	bacteria>actinobacteria	Kutzneria albida	hypothetical protein [Kutzneria albida].
WP_055574486.1	-	347	bacteria>actinobacteria	Streptomyces emeiensis	hypothetical protein, partial [Streptomyces emeiensis].
WP_003945635.1	-	344	bacteria>actinobacteria	Rhodococcus erythropolis	hypothetical protein [Rhodococcus erythropolis].
WP_007734628.1	-	344	bacteria>actinobacteria	Rhodococcus	MULTISPECIES: hypothetical protein [Rhodococcus].
WP_018534255.1	-	344	bacteria>actinobacteria	Streptomyces sp. HmicA12	hypothetical protein [Streptomyces sp. HmicA12].
WP_019747188.1	-	344	bacteria>actinobacteria	Rhodococcus	MULTISPECIES: hypothetical protein [Rhodococcus].
WP_020968638.1	-	344	bacteria>actinobacteria	Rhodococcus	MULTISPECIES: hypothetical protein [Rhodococcus].
WP_021333185.1	-	344	bacteria>actinobacteria	Rhodococcus erythropolis	hypothetical protein [Rhodococcus erythropolis].
WP_033231657.1	-	344	bacteria>actinobacteria	Rhodococcus	MULTISPECIES: hypothetical protein [Rhodococcus].
WP_050656149.1	-	344	bacteria>actinobacteria	Rhodococcus sp. 311R	hypothetical protein [Rhodococcus sp. 311R].
WP_060938315.1	-	344	bacteria>actinobacteria	Rhodococcus erythropolis	hypothetical protein [Rhodococcus erythropolis].
WP_019928480.1	-	343	bacteria>actinobacteria	Nocardia sp. BMG111209	hypothetical protein [Nocardia sp. BMG111209].
WP_028921645.1	-	342	bacteria>actinobacteria	Pseudonocardia acaciae	hypothetical protein [Pseudonocardia acaciae].
OBA61100.1	A5647_11870	342	bacteria>actinobacteria	Mycobacterium sp. 1100029.7	hypothetical protein A5647_11870 [Mycobacterium sp. 1100029.7].
WP_018557699.1	-	341	bacteria>actinobacteria	Streptomyces sp. BoleA5	hypothetical protein [Streptomyces sp. BoleA5].
KZM73184.1	AWN90_31330	341	bacteria>actinobacteria	Nocardia terpenica	hypothetical protein AWN90_31330 [Nocardia terpenica].
WP_036465398.1	-	340	bacteria>actinobacteria	Mycobacterium triplex	hypothetical protein [Mycobacterium triplex].
WP_060633744.1	-	340	bacteria>actinobacteria	Pseudonocardia sp. EC080610-09	hypothetical protein [Pseudonocardia sp. EC080610-09].
WP_060711732.1	-	339	bacteria>actinobacteria	Pseudonocardia sp. HH130629-09	hypothetical protein [Pseudonocardia sp. HH130629-09].
WP_014695116.1	-	338	bacteria>actinobacteria	Actinoplanes sp. SE50/110	hypothetical protein [Actinoplanes sp. SE50/110].
WP_062503669.1	-	334	bacteria>actinobacteria	Nocardia pseudobrasiliensis	hypothetical protein, partial [Nocardia pseudobrasiliensis].
EFD78336.1	TBEG_04038	330	bacteria>actinobacteria	Mycobacterium tuberculosis T85	conserved hypothetical protein [Mycobacterium tuberculosis T85].
KBJ66482.1	Q628_02451	329	bacteria>actinobacteria	Mycobacterium tuberculosis OFXR-18	hypothetical protein Q628_02451 [Mycobacterium tuberculosis OFXR-18].
WP_055473522.1	-	327	bacteria>actinobacteria	Streptomyces pathocidini	hypothetical protein [Streptomyces pathocidini].
WP_042365660.1	-	320	bacteria>actinobacteria	Streptacidiphilus neutrinimicus	hypothetical protein [Streptacidiphilus neutrinimicus].
WP_033391053.1	-	317	bacteria>actinobacteria	Kibdelosporangium aridum	hypothetical protein, partial [Kibdelosporangium aridum].
ETB49507.1	O981_21580	297	bacteria>actinobacteria	Mycobacterium avium 10-5560	hypothetical protein O981_21580 [Mycobacterium avium 10-5560].
KCN58133.1	X104_03822	289	bacteria>actinobacteria	Mycobacterium tuberculosis BTB06-494	hypothetical protein X104_03822 [Mycobacterium tuberculosis BTB06-494].
WP_010593644.1	-	273	bacteria>actinobacteria	Rhodococcus sp. P14	hypothetical protein, partial [Rhodococcus sp. P14].
WP_019359220.1	-	267	bacteria>actinobacteria	Streptomyces sp. AA1529	hypothetical protein, partial [Streptomyces sp. AA1529].
WP_030108248.1	-	265	bacteria>actinobacteria	Kutzneria albida	hypothetical protein [Kutzneria albida].
KAZ34925.1	AN95_02443	259	bacteria>actinobacteria	Mycobacterium tuberculosis M1400	hypothetical protein AN95_02443 [Mycobacterium tuberculosis M1400].
CKR16838.1	ERS027603_03817	231	bacteria>actinobacteria	Mycobacterium tuberculosis	Uncharacterised protein [Mycobacterium tuberculosis].
CFS33795.1	ERS013504_03784	224	bacteria>actinobacteria	Mycobacterium tuberculosis	Uncharacterised protein [Mycobacterium tuberculosis].
KID27755.1	HQ32_04981	206	bacteria>actinobacteria	Prauserella sp. Am3	hypothetical protein HQ32_04981, partial [Prauserella sp. Am3].
CFB05823.1	ERS024256_03644	187	bacteria>actinobacteria	Mycobacterium tuberculosis	Uncharacterised protein [Mycobacterium tuberculosis].
EWM10758.1	KUTG_01062	178	bacteria>actinobacteria	Kutzneria sp. 744	PE-PGRS family protein [Kutzneria sp. 744].
CFB05821.1	ERS024256_03643	177	bacteria>actinobacteria	Mycobacterium tuberculosis	Uncharacterised protein [Mycobacterium tuberculosis].
CNN41514.1	ERS023435_03590	177	bacteria>actinobacteria	Mycobacterium tuberculosis	Uncharacterised protein [Mycobacterium tuberculosis].
CKV03644.1	ERS031539_03816	174	bacteria>actinobacteria	Mycobacterium tuberculosis	Uncharacterised protein [Mycobacterium tuberculosis].
OCI27454.1	A7K94_12035	159	bacteria>actinobacteria	Modestobacter sp. VKM Ac-2676	hypothetical protein A7K94_12035 [Modestobacter sp. VKM Ac-2676].
CNN43591.1	ERS023439_03911	155	bacteria>actinobacteria	Mycobacterium tuberculosis	Uncharacterised protein [Mycobacterium tuberculosis].
EWM10757.1	KUTG_01061	153	bacteria>actinobacteria	Kutzneria sp. 744	LOW QUALITY PROTEIN: hypothetical protein KUTG_01061, partial [Kutzneria sp. 744].
SCG13086.1	GA0115260_122341	153	bacteria>actinobacteria	Streptomyces sp. MnatMP-M27	hypothetical protein GA0115260_122341, partial [Streptomyces sp. MnatMP-M27].
WP_036395143.1	-	113	bacteria>actinobacteria	Mycobacterium kansasii	hypothetical protein [Mycobacterium kansasii].
WP_030466813.1	-	100	bacteria>actinobacteria	Lechevalieria aerocolonigenes	hypothetical protein [Lechevalieria aerocolonigenes].
CFR87143.1	ERS007657_02570	97	bacteria>actinobacteria	Mycobacterium tuberculosis	Uncharacterised protein [Mycobacterium tuberculosis].

b. zinc ribbon PELOTA insert

WP_043813721.1	-	443	bacteria>proteobacteria>deltaproteobacteria	Desulfarculus baarsii	hypothetical protein [Desulfarculus baarsii]
WP_012099273.1	-	418	bacteria>proteobacteria>deltaproteobacteria	Anaeromyxobacter sp. Fw109-5	hypothetical protein [Anaeromyxobacter sp. Fw109-5]
WP_041453777.1	-	418	bacteria>proteobacteria>deltaproteobacteria	Anaeromyxobacter dehalogenans	hypothetical protein [Anaeromyxobacter dehalogenans]
WP_012528389.1	-	417	bacteria>proteobacteria>deltaproteobacteria	Anaeromyxobacter sp. K	hypothetical protein [Anaeromyxobacter sp. K]
WP_015935458.1	-	417	bacteria>proteobacteria>deltaproteobacteria	Anaeromyxobacter dehalogenans	hypothetical protein [Anaeromyxobacter dehalogenans]
WP_059435815.1	-	417	bacteria>proteobacteria>deltaproteobacteria	Anaeromyxobacter sp. PSR-1	hypothetical protein [Anaeromyxobacter sp. PSR-1]
WP_008476919.1	-	410	bacteria>chloroflexi	Nitrolancea hollandica	hypothetical protein [Nitrolancea hollandica]
CEK12418.1	CWRG_00014	407	bacteria>armatimonadetes	Chthonomonas calidirosea	peptide chain release factor 1 (eRF1) [Chthonomonas calidirosea]
CEK12419.1	CP488_00014	407	bacteria>armatimonadetes	Chthonomonas calidirosea	peptide chain release factor 1 (eRF1) [Chthonomonas calidirosea]
WP_016482508.1	-	407	bacteria>armatimonadetes	Chthonomonas calidirosea	peptide chain release factor 1 (eRF1) [Chthonomonas calidirosea]
WP_024342320.1	-	407	bacteria>proteobacteria>alphaproteobacteria	Bradyrhizobium japonicum	hypothetical protein [Bradyrhizobium japonicum]
WP_061027336.1	-	407	bacteria>proteobacteria>alphaproteobacteria	Bradyrhizobium sp. CCH5-F6	hypothetical protein [Bradyrhizobium sp. CCH5-F6]
WP_054491788.1	-	406	bacteria>chloroflexi	Ardenticatena maritima	hypothetical protein [Ardenticatena maritima]
WP_012845018.1	-	405	bacteria>bacteroidetes	Rhodothermus marinus	peptide chain release factor 1 [Rhodothermus marinus]
WP_014067991.1	-	405	bacteria>bacteroidetes	Rhodothermus marinus	peptide chain release factor 1 [Rhodothermus marinus]
WP_008474723.1	-	404	bacteria>chloroflexi	Nitrolancea hollandica	peptide chain release factor 1 [Nitrolancea hollandica]
AKU90366.1	AKJ08_0753	402	bacteria>proteobacteria>deltaproteobacteria	Vulgatibacter incomptus	hypothetical protein AKJ08_0753 [Vulgatibacter incomptus]
EFQ24116.1	Apau_1698	398	bacteria>synergistetes	Aminomonas paucivorans DSM 12260	conserved hypothetical protein [Aminomonas paucivorans DSM 12260]
WP_020576629.1	-	392	bacteria>actinobacteria	Actinopolymorpha alba	hypothetical protein [Actinopolymorpha alba]
WP_035239340.1	-	391	bacteria>acidobacteria	Acidobacteriaceae bacterium URHE0068	hypothetical protein [Acidobacteriaceae bacterium URHE0068]
WP_028072765.1	-	390	bacteria>actinobacteria	Solirubrobacterales bacterium URHD0059	hypothetical protein [Solirubrobacterales bacterium URHD0059]
WP_012641757.1	-	389	bacteria>chloroflexi	Thermomicrobium roseum	putative peptide chain release factor subunit 1 [Thermomicrobium roseum]
WP_011521408.1	-	388	bacteria>acidobacteria	Candidatus Koribacter versatilis	hypothetical protein [Candidatus Koribacter versatilis]
WP_038038985.1	-	388	bacteria>chloroflexi	Thermorudis peleae	hypothetical protein [Thermorudis peleae]
BAL59479.1	HGMM_OP4C115	387	bacteria	Candidatus Acetothermus autotrophicum	peptide chain release factor eRF subunit 1 [Candidatus Acetothermus autotrophicum]
WP_041730604.1	-	387	bacteria>actinobacteria	Conexibacter woesei	hypothetical protein [Conexibacter woesei]
KUK31656.1	XD63_1088	386	bacteria>firmicutes	Thermoanaerobacterales bacterium 50_218	hypothetical protein XD63_1088 [Thermoanaerobacterales bacterium 50_218]
WP_012871507.1	-	386	bacteria>chloroflexi	Sphaerobacter thermophilus	peptide chain release factor 1 [Sphaerobacter thermophilus]
KXS42730.1	AWU59_1482	385	archaea>euryarchaeota	Methanolobus sp. T82-4	peptide chain release factor subunit 1 [Methanolobus sp. T82-4]
WP_027006544.1	-	385	bacteria>actinobacteria	Conexibacter woesei	hypothetical protein [Conexibacter woesei]
CUT00080.1	JGI23_00781	384	bacteria	Candidatus Chrysopegis kryptomonas	peptide chain release factor subunit 1 [Candidatus Chrysopegis kryptomonas]
WP_014057090.1	-	384	bacteria>actinobacteria	Streptomyces violaceusniger	hypothetical protein [Streptomyces violaceusniger]
WP_037950474.1	-	384	bacteria>actinobacteria	Streptomyces sp. PRh5	hypothetical protein [Streptomyces sp. PRh5]
EKE03515.1	ACD_20C00196G0011	383	bacteria	uncultured bacterium	eRF1 protein [uncultured bacterium]
KUN44606.1	AQJ27_24540	383	bacteria>actinobacteria	Streptomyces olivochromogenes	hypothetical protein AQJ27_24540 [Streptomyces olivochromogenes]
SCE21441.1	GA0115240_14426	383	bacteria>actinobacteria	Streptomyces sp. DvalAA-14	peptide chain release factor subunit 1 [Streptomyces sp. DvalAA-14]
WP_006603518.1	-	383	bacteria>actinobacteria	Streptomyces auratus	hypothetical protein [Streptomyces auratus]
WP_013194456.1	-	383	archaea>euryarchaeota	Methanohalobium evestigatum	eRF1 domain 2 protein [Methanohalobium evestigatum]
WP_054228857.1	-	383	bacteria>actinobacteria	Actinobacteria bacterium OK006	hypothetical protein [Actinobacteria bacterium OK006]
WP_060899658.1	-	383	bacteria>actinobacteria	Streptomyces diastatochromogenes	hypothetical protein [Streptomyces diastatochromogenes]
WP_015227738.1	-	382	bacteria>cyanobacteria	Halothece sp. PCC 7418	hypothetical protein [Halothece sp. PCC 7418]
WP_026388860.1	-	382	bacteria>acidobacteria	Acidobacteria bacterium KBS 146	hypothetical protein [Acidobacteria bacterium KBS 146]
WP_041973770.1	-	382	bacteria>acidobacteria	Pyrinomonas methylaliphatogenes	hypothetical protein [Pyrinomonas methylaliphatogenes]
KUK39857.1	XD68_0603	381	bacteria>synergistetes	Synergistales bacterium 54_24	Uncharacterized protein XD68_0603 [Synergistales bacterium 54_24]
WP_009201527.1	-	381	bacteria>synergistetes	Acetomicrobium hydrogeniformans	hypothetical protein [Acetomicrobium hydrogeniformans]
ADB51863.1	Cwoe_3445	380	bacteria>actinobacteria	Conexibacter woesei DSM 14684	hypothetical protein Cwoe_3445 [Conexibacter woesei DSM 14684]
WP_013675599.1	-	380	bacteria>actinobacteria	Pseudonocardia dioxanivorans	hypothetical protein [Pseudonocardia dioxanivorans]
WP_030915820.1	-	380	bacteria>actinobacteria	Streptosporangium amethystogenes	hypothetical protein [Streptosporangium amethystogenes]
CUS97741.1	JGI24_00329	379	bacteria	Candidatus Kryptobacter tengchongensis	peptide chain release factor subunit 1 [Candidatus Kryptobacter tengchongensis]
CUU01526.1	JGI2_00727	379	bacteria	Candidatus Kryptobacter tengchongensis	peptide chain release factor subunit 1 [Candidatus Kryptobacter tengchongensis]
EFL34634.1	SSQG_05152	379	bacteria>actinobacteria	Streptomyces viridochromogenes DSM 40736	predicted protein [Streptomyces viridochromogenes DSM 40736]
KPJ60699.1	AMJ46_05445	379	bacteria	Latescibacteria bacterium DG_63	hypothetical protein AMJ46_05445 [Latescibacteria bacterium DG_63]
WP_040773352.1	-	379	bacteria>actinobacteria	Nocardia pneumoniae	hypothetical protein [Nocardia pneumoniae]
WP_040864580.1	-	379	bacteria>actinobacteria	Nocardia exalbida	hypothetical protein [Nocardia exalbida]
WP_043718680.1	-	379	bacteria>actinobacteria	Nocardia asiatica	hypothetical protein [Nocardia asiatica]
WP_014806327.1	-	378	bacteria>synergistetes	Acetomicrobium mobile	hypothetical protein [Acetomicrobium mobile]
WP_047133257.1	-	378	bacteria	Candidatus Kryptonium thompsoni	hypothetical protein [Candidatus Kryptonium thompsoni]
KRT75070.1	XU13_C0005G0023	376	bacteria	Candidatus Rokubacteria bacterium CSP1-6	eRF1 domain-containing protein [Candidatus Rokubacteria bacterium CSP1-6]
KUK55618.1	XD77_0382	376	bacteria	Marinimicrobia bacterium 46_47	Putative peptide chain release factor subunit 1 [Marinimicrobia bacterium 46_47]
WP_050792501.1	-	376	bacteria>synergistetes	Aminomonas paucivorans	zinc ribbon domain-containing protein [Aminomonas paucivorans]
WP_049674057.1	-	375	bacteria>proteobacteria>deltaproteobacteria	Desulfocarbo indianensis	hypothetical protein [Desulfocarbo indianensis]
KUK41780.1	XD69_0167	374	bacteria>firmicutes	Clostridia bacterium 62_21	hypothetical protein XD69_0167 [Clostridia bacterium 62_21]
WP_038048532.1	-	374	bacteria>acidobacteria	Thermoanaerobaculum aquaticum	hypothetical protein [Thermoanaerobaculum aquaticum]
KPK45031.1	AMK74_04170	373	bacteria>nitrospirae	Nitrospira bacterium SM23_35	hypothetical protein AMK74_04170 [Nitrospira bacterium SM23_35]
WP_044351840.1	-	373	bacteria>proteobacteria>deltaproteobacteria	Dethiosulfatarculus sandiegensis	hypothetical protein [Dethiosulfatarculus sandiegensis]
WP_046471535.1	-	373	bacteria>actinobacteria	Allosalinactinospora lopnorensis	hypothetical protein [Allosalinactinospora lopnorensis]
WP_012546631.1	-	372	bacteria>nitrospirae	Thermodesulfovibrio yellowstonii	hypothetical protein [Thermodesulfovibrio yellowstonii]
WP_028842812.1	-	372	bacteria>nitrospirae	Thermodesulfovibrio islandicus	hypothetical protein [Thermodesulfovibrio islandicus]
WP_059176691.1	-	372	bacteria>nitrospirae	Thermodesulfovibrio aggregans	hypothetical protein [Thermodesulfovibrio aggregans]
WP_025321727.1	-	371	bacteria>proteobacteria>deltaproteobacteria	Deferrisoma camini	hypothetical protein [Deferrisoma camini]
KKR18287.1	UT47_C0002G0026	370	bacteria	candidate division CPR2 bacterium GW2011_GWC2_39_35	hypothetical protein UT47_C0002G0026 [candidate division CPR2 bacterium GW2011_GWC2_39_35]
WP_028845434.1	-	370	bacteria>nitrospirae	Thermodesulfovibrio thiophilus	hypothetical protein [Thermodesulfovibrio thiophilus]
WP_012548230.1	-	369	bacteria>dictyoglomi	Dictyoglomus thermophilum	hypothetical protein [Dictyoglomus thermophilum]
WP_012583910.1	-	369	bacteria>dictyoglomi	Dictyoglomus turgidum	hypothetical protein [Dictyoglomus turgidum]
KPK92091.1	AMJ88_11785	368	bacteria>chloroflexi	Anaerolineae bacterium SM23_ 63	hypothetical protein AMJ88_11785 [Anaerolineae bacterium SM23_ 63]
WP_007569800.1	-	367	bacteria>actinobacteria	Patulibacter medicamentivorans	hypothetical protein [Patulibacter medicamentivorans]
WP_030466803.1	-	367	bacteria>actinobacteria	Lechevalieria aerocolonigenes	hypothetical protein [Lechevalieria aerocolonigenes]
GAP12371.1	LARV_00105	366	bacteria>chloroflexi	Longilinea arvoryzae	hypothetical protein LARV_00105 [Longilinea arvoryzae]
KPL79312.1	ADN00_02990	366	bacteria>chloroflexi	Ornatilinea apprima	hypothetical protein ADN00_02990 [Ornatilinea apprima]
WP_054521936.1	-	366	bacteria>chloroflexi	Thermanaerothrix daxensis	hypothetical protein [Thermanaerothrix daxensis]
WP_061913942.1	-	366	bacteria>chloroflexi	Bellilinea caldifistulae	hypothetical protein [Bellilinea caldifistulae]
WP_062422550.1	-	366	bacteria>chloroflexi	Leptolinea tardivitalis	hypothetical protein [Leptolinea tardivitalis]
BAL54759.1	HGMM_F19G07C10	365	bacteria>chloroflexi	uncultured Chloroflexi bacterium	hypothetical conserved protein [uncultured Chloroflexi bacterium]
KUK94360.1	XE06_0820	365	bacteria>chloroflexi	Anaerolineaceae bacterium 46_22	hypothetical protein XE06_0820 [Anaerolineaceae bacterium 46_22]
WP_062416922.1	-	365	bacteria>chloroflexi	Levilinea saccharolytica	hypothetical protein [Levilinea saccharolytica]
KQC12194.1	APR63_11630	363	bacteria>proteobacteria>deltaproteobacteria	Desulfuromonas sp. SDB	hypothetical protein APR63_11630 [Desulfuromonas sp. SDB]
KPL23918.1	AMJ93_03545	362	bacteria>chloroflexi	Anaerolineae bacterium SM23_84	hypothetical protein AMJ93_03545 [Anaerolineae bacterium SM23_84]
WP_006583196.1	-	358	bacteria>synergistetes	Thermanaerovibrio velox	hypothetical protein [Thermanaerovibrio velox]
WP_012869966.1	-	354	bacteria>synergistetes	Thermanaerovibrio acidaminovorans	hypothetical protein [Thermanaerovibrio acidaminovorans]
CUS80909.1	JGI14_10099	349	bacteria	Candidatus Kryptonium thompsoni	Peptide chain release factor 1 (eRF1) [Candidatus Kryptonium thompsoni]

3. family 3

##;baeRF1 family 3 follows the standard core architecture: codon_recognition_domain+baeRF1+Pelota.
##;The Pelota domain appears to contain the 2 beta-strand insert characteristic of the clade.

WP_035767954.1	-	350	bacteria>actinobacteria	Arthrobacter castelli	hypothetical protein [Arthrobacter castelli].
WP_022871234.1	-	346	bacteria>actinobacteria	Yaniella halotolerans	hypothetical protein [Yaniella halotolerans].
ALM38599.1	SFR_1984	552	bacteria>actinobacteria	Streptomyces sp. FR-008	chemotaxis protein [Streptomyces sp. FR-008].
ALO91883.1	SHL15_0686	485	bacteria>actinobacteria	Streptomyces hygroscopicus subsp. limoneus	chemotaxis protein [Streptomyces hygroscopicus subsp. limoneus].
WP_030812309.1	-	394	bacteria>actinobacteria	Streptomyces sp. NRRL F-2799	hypothetical protein [Streptomyces sp. NRRL F-2799].
WP_031181370.1	-	394	bacteria>actinobacteria	Streptomyces seoulensis	hypothetical protein [Streptomyces seoulensis].
WP_018549393.1	-	393	bacteria>actinobacteria	Streptomyces sp. LaPpAH-108	hypothetical protein [Streptomyces sp. LaPpAH-108].
WP_015793177.1	-	391	bacteria>actinobacteria	Catenulispora acidiphila	hypothetical protein [Catenulispora acidiphila].
WP_030118968.1	-	391	bacteria>actinobacteria	Streptomyces	MULTISPECIES: hypothetical protein [Streptomyces].
WP_030194167.1	-	391	bacteria>actinobacteria	Streptomyces	MULTISPECIES: hypothetical protein [Streptomyces].
WP_030297274.1	-	391	bacteria>actinobacteria	Streptomyces sp. NRRL F-5681	hypothetical protein [Streptomyces sp. NRRL F-5681].
WP_030334133.1	-	391	bacteria>actinobacteria	Streptomyces sp. NRRL B-1381	hypothetical protein [Streptomyces sp. NRRL B-1381].
WP_030344986.1	-	391	bacteria>actinobacteria	Streptomyces floridae	hypothetical protein [Streptomyces floridae].
WP_030849741.1	-	391	bacteria>actinobacteria	Streptomyces griseus	hypothetical protein [Streptomyces griseus].
WP_031058298.1	-	391	bacteria>actinobacteria	Streptomyces sp. NRRL F-5702	hypothetical protein [Streptomyces sp. NRRL F-5702].
WP_032773245.1	-	391	bacteria>actinobacteria	Streptomyces	MULTISPECIES: hypothetical protein [Streptomyces].
WP_042422626.1	-	391	bacteria>actinobacteria	Streptacidiphilus anmyonensis	hypothetical protein [Streptacidiphilus anmyonensis].
WP_043252324.1	-	391	bacteria>actinobacteria	Streptomyces vinaceus	hypothetical protein [Streptomyces vinaceus].
KDN88041.1	KCH_01180	390	bacteria>actinobacteria	Kitasatospora cheerisanensis KCTC 2395	hypothetical protein KCH_01180 [Kitasatospora cheerisanensis KCTC 2395].
KKS68731.1	UV38_C0001G0272	389	bacteria	candidate division TM6 bacterium GW2011_GWE2_42_60	hypothetical protein UV38_C0001G0272 [candidate division TM6 bacterium GW2011_GWE2_42_60].
WP_037571260.1	-	388	bacteria>actinobacteria	Streptacidiphilus oryzae	hypothetical protein [Streptacidiphilus oryzae].
CUW26265.1	TUE45_00976	388	bacteria>actinobacteria	Streptomyces reticuli	hypothetical protein TUE45_00976 [Streptomyces reticuli].
KOU06441.1	ADK88_14420	386	bacteria>actinobacteria	Streptomyces sp. NRRL F-2295	hypothetical protein ADK88_14420 [Streptomyces sp. NRRL F-2295].
KOU50942.1	ADK56_11820	386	bacteria>actinobacteria	Streptomyces sp. MMG1522	hypothetical protein ADK56_11820 [Streptomyces sp. MMG1522].
EKC91522.1	SM8_05793	384	bacteria>actinobacteria	Streptomyces sp. SM8	hypothetical protein SM8_05793 [Streptomyces sp. SM8].
WP_003950844.1	-	384	bacteria>actinobacteria	Streptomyces	MULTISPECIES: hypothetical protein [Streptomyces].
WP_018470779.1	-	384	bacteria>actinobacteria	Streptomyces	MULTISPECIES: hypothetical protein [Streptomyces].
WP_018893550.1	-	384	bacteria>actinobacteria	Streptomyces sp. CNY228	hypothetical protein [Streptomyces sp. CNY228].
WP_023417087.1	-	384	bacteria>actinobacteria	Streptomyces	MULTISPECIES: hypothetical protein [Streptomyces].
WP_023421708.1	-	384	bacteria>actinobacteria	Streptomyces sp. GBA 94-10	hypothetical protein [Streptomyces sp. GBA 94-10].
WP_030306303.1	-	384	bacteria>actinobacteria	Streptomyces	MULTISPECIES: hypothetical protein [Streptomyces].
WP_030695604.1	-	384	bacteria>actinobacteria	Streptomyces griseus	hypothetical protein [Streptomyces griseus].
WP_030765354.1	-	384	bacteria>actinobacteria	Streptomyces griseus	hypothetical protein [Streptomyces griseus].
WP_033238098.1	-	384	bacteria>actinobacteria	Streptomyces griseus	hypothetical protein [Streptomyces griseus].
WP_047142667.1	-	384	bacteria>actinobacteria	Streptomyces sp. KE1	hypothetical protein [Streptomyces sp. KE1].
WP_047467254.1	-	384	bacteria>actinobacteria	Streptomyces sp. M10	hypothetical protein [Streptomyces sp. M10].
WP_049977077.1	-	384	bacteria>actinobacteria	Streptomyces wadayamensis	hypothetical protein [Streptomyces wadayamensis].
WP_061404217.1	-	384	bacteria>actinobacteria	Streptomyces albus	hypothetical protein [Streptomyces albus].
SCD32668.1	GA0115250_102718	384	bacteria>actinobacteria	Streptomyces sp. BvitLS-983	hypothetical protein GA0115250_102718 [Streptomyces sp. BvitLS-983].
AIR96610.1	SGLAU_02910	382	bacteria>actinobacteria	Streptomyces glaucescens	hypothetical protein SGLAU_02910 [Streptomyces glaucescens].
SCF70784.1	GA0115259_101336	382	bacteria>actinobacteria	Streptomyces sp. MnatMP-M17	hypothetical protein GA0115259_101336 [Streptomyces sp. MnatMP-M17].
AJC59911.1	GZL_07359	381	bacteria>actinobacteria	Streptomyces sp. 769	hypothetical protein GZL_07359 [Streptomyces sp. 769].
WP_046496397.1	-	381	bacteria>actinobacteria	Streptomyces sp. NRRL B-24891	hypothetical protein [Streptomyces sp. NRRL B-24891].
WP_016909439.1	-	378	bacteria>actinobacteria	Streptomyces xiaopingdaonensis	hypothetical protein [Streptomyces xiaopingdaonensis].
WP_026328380.1	-	378	bacteria>actinobacteria	Streptomyces sulphureus	hypothetical protein [Streptomyces sulphureus].
WP_004975962.1	-	377	bacteria>proteobacteria>gammaproteobacteria	Acinetobacter towneri	hypothetical protein [Acinetobacter towneri].
WP_019356343.1	-	377	bacteria>actinobacteria	Streptomyces	MULTISPECIES: hypothetical protein [Streptomyces].
WP_019630939.1	-	377	bacteria>actinobacteria	Actinomadura atramentaria	hypothetical protein [Actinomadura atramentaria].
WP_020551223.1	-	377	bacteria>actinobacteria	Streptomyces scabrisporus	hypothetical protein [Streptomyces scabrisporus].
WP_027738333.1	-	377	bacteria>actinobacteria	Streptomyces sp. CNT360	hypothetical protein [Streptomyces sp. CNT360].
WP_014218146.1	-	376	bacteria>bacteroidetes	Niastella koreensis	hypothetical protein [Niastella koreensis].
WP_018616436.1	-	376	bacteria>bacteroidetes	Segetibacter koreensis	hypothetical protein [Segetibacter koreensis].
WP_020610307.1	-	376	bacteria>actinobacteria	Streptomyces sp. CNT372	hypothetical protein [Streptomyces sp. CNT372].
WP_030365748.1	-	376	bacteria>actinobacteria	Streptomyces roseoverticillatus	hypothetical protein [Streptomyces roseoverticillatus].
WP_042392034.1	-	376	bacteria>actinobacteria	Streptacidiphilus carbonis	hypothetical protein [Streptacidiphilus carbonis].
KKT23990.1	UW09_C0001G0053	376	bacteria	candidate division TM6 bacterium GW2011_GWF2_43_87	hypothetical protein UW09_C0001G0053 [candidate division TM6 bacterium GW2011_GWF2_43_87].
WP_004991535.1	-	375	bacteria>proteobacteria>gammaproteobacteria	Acinetobacter ursingii	hypothetical protein [Acinetobacter ursingii].
WP_005001258.1	-	375	bacteria>proteobacteria>gammaproteobacteria	Acinetobacter ursingii	hypothetical protein [Acinetobacter ursingii].
WP_034698997.1	-	375	bacteria>proteobacteria>gammaproteobacteria	Acinetobacter sp. 479375	hypothetical protein [Acinetobacter sp. 479375].
WP_044434009.1	-	375	bacteria>proteobacteria>gammaproteobacteria	Acinetobacter ursingii	hypothetical protein [Acinetobacter ursingii].
WP_049173922.1	-	375	bacteria>proteobacteria>gammaproteobacteria	Acinetobacter ursingii	hypothetical protein [Acinetobacter ursingii].
WP_058043405.1	-	375	bacteria>actinobacteria	Streptomyces sp. MBT76	hypothetical protein [Streptomyces sp. MBT76].
OBX75992.1	A9306_01605	375	bacteria>proteobacteria>gammaproteobacteria	Moraxella atlantae	hypothetical protein A9306_01605 [Moraxella atlantae].
OBX80888.1	A9308_02750	375	bacteria>proteobacteria>gammaproteobacteria	Moraxella atlantae	hypothetical protein A9308_02750 [Moraxella atlantae].
WP_005474384.1	-	374	bacteria>actinobacteria	Streptomyces bottropensis	hypothetical protein [Streptomyces bottropensis].
WP_011112527.1	-	374	bacteria>proteobacteria>betaproteobacteria	Nitrosomonas europaea	hypothetical protein [Nitrosomonas europaea].
WP_013005887.1	-	374	bacteria>actinobacteria	Streptomyces scabiei	hypothetical protein [Streptomyces scabiei].
KDN24329.1	MBO_09293	374	bacteria>proteobacteria>gammaproteobacteria	Moraxella bovoculi 237	hypothetical protein MBO_09293 [Moraxella bovoculi 237].
WP_035841428.1	-	374	bacteria>actinobacteria	Kitasatospora azatica	hypothetical protein [Kitasatospora azatica].
WP_036604822.1	-	374	bacteria>firmicutes	Paenibacillus sophorae	hypothetical protein [Paenibacillus sophorae].
WP_046918148.1	-	374	bacteria>actinobacteria	Streptomyces stelliscabiei	hypothetical protein [Streptomyces stelliscabiei].
WP_057239663.1	-	374	bacteria>actinobacteria	Kitasatospora	MULTISPECIES: hypothetical protein [Kitasatospora].
WP_059083062.1	-	374	bacteria>actinobacteria	Streptomyces scabiei	hypothetical protein [Streptomyces scabiei].
KUJ70989.1	ACZ90_01450	374	bacteria>actinobacteria	Streptomyces albus subsp. albus	chemotaxis protein [Streptomyces albus subsp. albus].
WP_060907695.1	-	374	bacteria>actinobacteria	Streptomyces scabiei	hypothetical protein [Streptomyces scabiei].
KXB96974.1	AA908_09330	374	bacteria>chlorobi	Chlorobi bacterium NICIL-2	hypothetical protein AA908_09330 [Chlorobi bacterium NICIL-2].
WP_060994964.1	-	374	bacteria>actinobacteria	Corynebacterium durum	hypothetical protein [Corynebacterium durum].
WP_062333043.1	-	374	bacteria>proteobacteria>gammaproteobacteria	Moraxella osloensis	hypothetical protein [Moraxella osloensis].
WP_065253167.1	-	374	bacteria>proteobacteria>gammaproteobacteria	Moraxella osloensis	hypothetical protein [Moraxella osloensis].
WP_065263964.1	-	374	bacteria>proteobacteria>gammaproteobacteria	Moraxella osloensis	hypothetical protein [Moraxella osloensis].
WP_065965587.1	-	374	bacteria>actinobacteria	Streptomyces sparsogenes	chemotaxis protein [Streptomyces sparsogenes].
WP_005016898.1	-	373	bacteria>proteobacteria>gammaproteobacteria	Acinetobacter	MULTISPECIES: hypothetical protein [Acinetobacter].
WP_005023144.1	-	373	bacteria>proteobacteria>gammaproteobacteria	Acinetobacter	MULTISPECIES: hypothetical protein [Acinetobacter].
WP_014175672.1	-	373	bacteria>actinobacteria	Streptomyces bingchenggensis	hypothetical protein [Streptomyces bingchenggensis].
WP_017400677.1	-	373	bacteria>proteobacteria>gammaproteobacteria	Acinetobacter radioresistens	hypothetical protein [Acinetobacter radioresistens].
WP_018560735.1	-	373	bacteria>actinobacteria	Streptomyces sp. BoleA5	hypothetical protein [Streptomyces sp. BoleA5].
WP_026444091.1	-	373	bacteria>proteobacteria>gammaproteobacteria	Acinetobacter sp. UNC436CL71CviS28	hypothetical protein [Acinetobacter sp. UNC436CL71CviS28].
WP_026898252.1	-	373	bacteria>bacteroidetes	Pedobacter oryzae	hypothetical protein [Pedobacter oryzae].
WP_034684928.1	-	373	bacteria>proteobacteria>gammaproteobacteria	Acinetobacter sp. 1294596	hypothetical protein [Acinetobacter sp. 1294596].
WP_040019251.1	-	373	bacteria>actinobacteria	Streptomyces sp. 150FB	hypothetical protein [Streptomyces sp. 150FB].
WP_046206291.1	-	373	bacteria>proteobacteria>gammaproteobacteria	Acinetobacter radioresistens	hypothetical protein [Acinetobacter radioresistens].
WP_014218538.1	-	372	bacteria>bacteroidetes	Niastella koreensis	hypothetical protein [Niastella koreensis].
WP_027748723.1	-	372	bacteria>actinobacteria	Streptomyces sp. CNH287	hypothetical protein [Streptomyces sp. CNH287].
WP_028813094.1	-	372	bacteria>actinobacteria	Streptomyces flavidovirens	hypothetical protein [Streptomyces flavidovirens].
WP_033823545.1	-	372	bacteria>actinobacteria	Kitasatospora sp. MBT63	hypothetical protein [Kitasatospora sp. MBT63].
WP_037692089.1	-	372	bacteria>actinobacteria	Streptomyces scabiei	hypothetical protein [Streptomyces scabiei].
KKR96141.1	UU47_C0018G0001	372	bacteria	candidate division TM6 bacterium GW2011_GWE2_41_16	hypothetical protein UU47_C0018G0001 [candidate division TM6 bacterium GW2011_GWE2_41_16].
WP_003971083.1	-	371	bacteria>actinobacteria	Streptomyces	MULTISPECIES: hypothetical protein [Streptomyces].
WP_007446918.1	-	371	bacteria>actinobacteria	Streptomyces sp. W007	hypothetical protein [Streptomyces sp. W007].
WP_010060771.1	-	371	bacteria>actinobacteria	Streptomyces globisporus	hypothetical protein [Streptomyces globisporus].
WP_012382196.1	-	371	bacteria>actinobacteria	Streptomyces griseus	hypothetical protein [Streptomyces griseus].
WP_014044210.1	-	371	bacteria>actinobacteria	Streptomyces	MULTISPECIES: hypothetical protein [Streptomyces].
WP_014157986.1	-	371	bacteria>actinobacteria	Streptomyces pratensis	hypothetical protein [Streptomyces pratensis].
WP_015575879.1	-	371	bacteria>actinobacteria	Streptomyces sp. PAMC 26508	hypothetical protein [Streptomyces sp. PAMC 26508].
WP_015606730.1	-	371	bacteria>actinobacteria	Streptomyces fulvissimus	methyl-accepting chemotaxis protein [Streptomyces fulvissimus].
WP_016575540.1	-	371	bacteria>actinobacteria	Streptomyces	MULTISPECIES: hypothetical protein [Streptomyces].
WP_018102893.1	-	371	bacteria>actinobacteria	Streptomyces	MULTISPECIES: hypothetical protein [Streptomyces].
WP_018491153.1	-	371	bacteria>actinobacteria	Streptomyces sp. CcalMP-8W	hypothetical protein [Streptomyces sp. CcalMP-8W].
WP_018515066.1	-	371	bacteria>actinobacteria	Streptomyces sp. ScaeMP-e10	hypothetical protein [Streptomyces sp. ScaeMP-e10].
WP_018524528.1	-	371	bacteria>actinobacteria	Streptomyces	MULTISPECIES: hypothetical protein [Streptomyces].
WP_018539943.1	-	371	bacteria>actinobacteria	Streptomyces sp. MspMP-M5	hypothetical protein [Streptomyces sp. MspMP-M5].
WP_018550697.1	-	371	bacteria>actinobacteria	Streptomyces sp. ATexAB-D23	hypothetical protein [Streptomyces sp. ATexAB-D23].
WP_018956503.1	-	371	bacteria>actinobacteria	Streptomyces sp. CNB091	hypothetical protein [Streptomyces sp. CNB091].
WP_023543629.1	-	371	bacteria>actinobacteria	Streptomyces niveus	hypothetical protein [Streptomyces niveus].
WP_024492545.1	-	371	bacteria>actinobacteria	Streptomyces sp. AW19M42	chemotaxis protein [Streptomyces sp. AW19M42].
WP_028417081.1	-	371	bacteria>actinobacteria	Streptomyces sp. SolWspMP-sol2th	chemotaxis protein [Streptomyces sp. SolWspMP-sol2th].
WP_028441945.1	-	371	bacteria>actinobacteria	Streptomyces sp. DpondAA-B6	hypothetical protein [Streptomyces sp. DpondAA-B6].
WP_030086409.1	-	371	bacteria>actinobacteria	Streptomyces	MULTISPECIES: hypothetical protein [Streptomyces].
WP_030123316.1	-	371	bacteria>actinobacteria	[Kitasatospora] papulosa	hypothetical protein [[Kitasatospora] papulosa].
WP_030265190.1	-	371	bacteria>actinobacteria	Streptomyces sp. NRRL B-24484	hypothetical protein [Streptomyces sp. NRRL B-24484].
WP_030286377.1	-	371	bacteria>actinobacteria	Streptomyces catenulae	chemotaxis protein [Streptomyces catenulae].
WP_030566164.1	-	371	bacteria>actinobacteria	Streptomyces cyaneofuscatus	chemotaxis protein [Streptomyces cyaneofuscatus].
WP_030582416.1	-	371	bacteria>actinobacteria	Streptomyces anulatus	hypothetical protein [Streptomyces anulatus].
WP_030587730.1	-	371	bacteria>actinobacteria	Streptomyces globisporus	hypothetical protein [Streptomyces globisporus].
WP_030627529.1	-	371	bacteria>actinobacteria	Streptomyces	MULTISPECIES: hypothetical protein [Streptomyces].
WP_030635137.1	-	371	bacteria>actinobacteria	Streptomyces	MULTISPECIES: hypothetical protein [Streptomyces].
WP_030721028.1	-	371	bacteria>actinobacteria	Streptomyces griseus	hypothetical protein [Streptomyces griseus].
WP_030809573.1	-	371	bacteria>actinobacteria	Streptomyces mediolani	hypothetical protein [Streptomyces mediolani].
WP_030916906.1	-	371	bacteria>actinobacteria	Streptomyces sp. NRRL B-24720	hypothetical protein [Streptomyces sp. NRRL B-24720].
WP_031087159.1	-	371	bacteria>actinobacteria	Streptomyces sp. NRRL WC-3549	hypothetical protein [Streptomyces sp. NRRL WC-3549].
WP_031095914.1	-	371	bacteria>actinobacteria	Streptomyces	MULTISPECIES: hypothetical protein [Streptomyces].
WP_031126626.1	-	371	bacteria>actinobacteria	Streptomyces sp. NRRL S-623	chemotaxis protein [Streptomyces sp. NRRL S-623].
WP_032754429.1	-	371	bacteria>actinobacteria	Streptomyces alboviridis	chemotaxis protein [Streptomyces alboviridis].
WP_032768824.1	-	371	bacteria>actinobacteria	Streptomyces sp. CNS654	hypothetical protein [Streptomyces sp. CNS654].
WP_032778179.1	-	371	bacteria>actinobacteria	Streptomyces albus	chemotaxis protein [Streptomyces albus].
WP_032788897.1	-	371	bacteria>actinobacteria	Streptomyces sp. JS01	chemotaxis protein [Streptomyces sp. JS01].
WP_033248053.1	-	371	bacteria>actinobacteria	Streptomyces	MULTISPECIES: hypothetical protein [Streptomyces].
WP_033304877.1	-	371	bacteria>actinobacteria	Streptomyces atroolivaceus	hypothetical protein [Streptomyces atroolivaceus].
WP_037692180.1	-	371	bacteria>actinobacteria	Streptomyces atratus	chemotaxis protein [Streptomyces atratus].
WP_037882929.1	-	371	bacteria>actinobacteria	Streptomyces sp. NTK 937	hypothetical protein [Streptomyces sp. NTK 937].
WP_043263394.1	-	371	bacteria>actinobacteria	Streptomyces sp. CT34	hypothetical protein [Streptomyces sp. CT34].
WP_044369888.1	-	371	bacteria>actinobacteria	Streptomyces griseus	chemotaxis protein [Streptomyces griseus].
WP_046709263.1	-	371	bacteria>actinobacteria	Streptomyces europaeiscabiei	hypothetical protein [Streptomyces europaeiscabiei].
WP_047175064.1	-	371	bacteria>actinobacteria	Streptomyces sp. MNU77	hypothetical protein [Streptomyces sp. MNU77].
WP_050361569.1	-	371	bacteria>actinobacteria	Streptomyces europaeiscabiei	hypothetical protein [Streptomyces europaeiscabiei].
WP_053562462.1	-	371	bacteria>actinobacteria	Streptomyces sp. CFMR 7	hypothetical protein [Streptomyces sp. CFMR 7].
WP_055559305.1	-	371	bacteria>actinobacteria	Streptomyces luridiscabiei	hypothetical protein [Streptomyces luridiscabiei].
WP_056707947.1	-	371	bacteria>actinobacteria	Streptomyces	MULTISPECIES: hypothetical protein [Streptomyces].
WP_056787843.1	-	371	bacteria>actinobacteria	Streptomyces	MULTISPECIES: hypothetical protein [Streptomyces].
KUL33480.1	ADL22_32955	371	bacteria>actinobacteria	Streptomyces sp. NRRL F-4489	chemotaxis protein [Streptomyces sp. NRRL F-4489].
WP_060892359.1	-	371	bacteria>actinobacteria	Streptomyces europaeiscabiei	hypothetical protein [Streptomyces europaeiscabiei].
SBU92484.1	YW5DRAFT_04003	371	bacteria>actinobacteria	Streptomyces sp. Ncost-T6T-1	hypothetical protein YW5DRAFT_04003 [Streptomyces sp. Ncost-T6T-1].
WP_065484657.1	-	371	bacteria>actinobacteria	Streptomyces sp. PTY087I2	chemotaxis protein [Streptomyces sp. PTY087I2].
ANZ20001.1	SNOUR_33850	371	bacteria>actinobacteria	Streptomyces noursei ATCC 11455	hypothetical protein SNOUR_33850 [Streptomyces noursei ATCC 11455].
SCD68365.1	GA0115247_111019	371	bacteria>actinobacteria	Streptomyces sp. PalvLS-984	hypothetical protein GA0115247_111019 [Streptomyces sp. PalvLS-984].
SCD72091.1	GA0115239_106528	371	bacteria>actinobacteria	Streptomyces sp. BpilaLS-43	hypothetical protein GA0115239_106528 [Streptomyces sp. BpilaLS-43].

SCE36954.1	GA0115243_1115236	371	bacteria>actinobacteria	Streptomyces sp. ScaeMP-e83	hypothetical protein GA0115243_1115236 [Streptomyces sp. ScaeMP-e83].
SCE60573.1	GA0115253_1086111	371	bacteria>actinobacteria	Streptomyces sp. Termitarium-T10T-6	hypothetical protein GA0115253_1086111 [Streptomyces sp. Termitarium-T10T-6].
SCD65985.1	GA0115244_107919	371	bacteria>actinobacteria	Streptomyces sp. DvalAA-19	hypothetical protein GA0115244_107919 [Streptomyces sp. DvalAA-19].
SCD80996.1	GA0115261_1018817	371	bacteria>actinobacteria	Streptomyces sp. OspMP-M43	hypothetical protein GA0115261_1018817 [Streptomyces sp. OspMP-M43].
SCE59524.1	GA0115234_1147207	371	bacteria>actinobacteria	Streptomyces sp. DvalAA-43	hypothetical protein GA0115234_1147207 [Streptomyces sp. DvalAA-43].
SCF92302.1	GA0115280_119913	371	bacteria>actinobacteria	Streptomyces sp. Cmuel-A718b	hypothetical protein GA0115280_119913 [Streptomyces sp. Cmuel-A718b].
SCF78463.1	GA0115254_116926	371	bacteria>actinobacteria	Streptomyces sp. Ncost-T10-10d	hypothetical protein GA0115254_116926 [Streptomyces sp. Ncost-T10-10d].
WP_007395944.1	-	370	bacteria>proteobacteria>gammaproteobacteria	Psychrobacter sp. 1501(2011)	hypothetical protein [Psychrobacter sp. 1501(2011)].
WP_010200740.1	-	370	bacteria>proteobacteria>gammaproteobacteria	Psychrobacter sp. PAMC 21119	hypothetical protein [Psychrobacter sp. PAMC 21119].
WP_011279392.1	-	370	bacteria>proteobacteria>gammaproteobacteria	Psychrobacter arcticus	hypothetical protein [Psychrobacter arcticus].
WP_011512460.1	-	370	bacteria>proteobacteria>gammaproteobacteria	Psychrobacter	MULTISPECIES: hypothetical protein [Psychrobacter].
WP_011961451.1	-	370	bacteria>proteobacteria>gammaproteobacteria	Psychrobacter sp. PRwf-1	hypothetical protein [Psychrobacter sp. PRwf-1].
WP_019673591.1	-	370	bacteria>proteobacteria>gammaproteobacteria	Psychrobacter lutiphocae	hypothetical protein [Psychrobacter lutiphocae].
WP_021814121.1	-	370	bacteria>proteobacteria>gammaproteobacteria	Psychrobacter aquaticus	hypothetical protein [Psychrobacter aquaticus].
WP_023790907.1	-	370	bacteria>proteobacteria>deltaproteobacteria	Candidatus Babela massiliensis	hypothetical protein [Candidatus Babela massiliensis].
GAF55803.1	JCM18901_1475	370	bacteria>proteobacteria>gammaproteobacteria	Psychrobacter sp. JCM 18901	LOW QUALITY PROTEIN: methyl-accepting chemotaxis protein [Psychrobacter sp. JCM 18901].
GAF51683.1	JCM18900_162	370	bacteria>proteobacteria>gammaproteobacteria	Psychrobacter sp. JCM 18900	LOW QUALITY PROTEIN: methyl-accepting chemotaxis protein [Psychrobacter sp. JCM 18900].
WP_025646198.1	-	370	bacteria>proteobacteria>gammaproteobacteria	Psychrobacter	MULTISPECIES: hypothetical protein [Psychrobacter].
WP_025652573.1	-	370	bacteria>proteobacteria>gammaproteobacteria	Psychrobacter	MULTISPECIES: hypothetical protein [Psychrobacter].
WP_028859213.1	-	370	bacteria>proteobacteria>gammaproteobacteria	Psychrobacter phenylpyruvicus	hypothetical protein [Psychrobacter phenylpyruvicus].
WP_030252006.1	-	370	bacteria>actinobacteria	Streptacidiphilus jeojiense	hypothetical protein [Streptacidiphilus jeojiense].
WP_030902225.1	-	370	bacteria>actinobacteria	Streptomyces sp. NRRL F-5126	hypothetical protein [Streptomyces sp. NRRL F-5126].
WP_041998490.1	-	370	bacteria>actinobacteria	Streptomyces sp. AcH 505	chemotaxis protein [Streptomyces sp. AcH 505].
WP_045443051.1	-	370	bacteria>proteobacteria>gammaproteobacteria	Psychrobacter glacincola	hypothetical protein [Psychrobacter glacincola].
WP_045454884.1	-	370	bacteria>proteobacteria>gammaproteobacteria	Psychrobacter sp. P11F6	hypothetical protein [Psychrobacter sp. P11F6].
AKG08833.1	AAX06_10860	370	bacteria>proteobacteria>gammaproteobacteria	Moraxella bovoculi	hypothetical protein AAX06_10860 [Moraxella bovoculi].
AKG17781.1	AAX10_09240	370	bacteria>proteobacteria>gammaproteobacteria	Moraxella bovoculi	hypothetical protein AAX10_09240 [Moraxella bovoculi].
AKG19562.1	AAX09_09540	370	bacteria>proteobacteria>gammaproteobacteria	Moraxella bovoculi	hypothetical protein AAX09_09540 [Moraxella bovoculi].
WP_057757779.1	-	370	bacteria>proteobacteria>gammaproteobacteria	Psychrobacter sp. P11G3	hypothetical protein [Psychrobacter sp. P11G3].
WP_058023597.1	-	370	bacteria>proteobacteria>gammaproteobacteria	Psychrobacter piscatorii	hypothetical protein [Psychrobacter piscatorii].
WP_058367866.1	-	370	bacteria>proteobacteria>gammaproteobacteria	Psychrobacter sp. ENNN9_III	hypothetical protein [Psychrobacter sp. ENNN9_III].
ALT07604.1	AAX08_09600	370	bacteria>proteobacteria>gammaproteobacteria	Moraxella bovoculi	hypothetical protein AAX08_09600 [Moraxella bovoculi].
AMN48563.1	AK823_00485	370	bacteria>proteobacteria>gammaproteobacteria	Psychrobacter sp. P2G3	hypothetical protein AK823_00485 [Psychrobacter sp. P2G3].
AMN66389.1	AK825_00470	370	bacteria>proteobacteria>gammaproteobacteria	Psychrobacter sp. P11G5	hypothetical protein AK825_00470 [Psychrobacter sp. P11G5].
WP_062843532.1	-	370	bacteria>proteobacteria>gammaproteobacteria	Psychrobacter alimentarius	hypothetical protein [Psychrobacter alimentarius].
WP_063515053.1	-	370	bacteria>proteobacteria>gammaproteobacteria	Moraxella ovis	hypothetical protein [Moraxella ovis].
OAP68171.1	A7325_04550	370	bacteria>proteobacteria>gammaproteobacteria	Psychrobacter sp. SHUES1	hypothetical protein A7325_04550 [Psychrobacter sp. SHUES1].
WP_007829881.1	-	369	bacteria>actinobacteria	Streptomyces sp. Tu6071	hypothetical protein [Streptomyces sp. Tu6071].
WP_009062962.1	-	369	bacteria>actinobacteria	Streptomyces	MULTISPECIES: hypothetical protein [Streptomyces].
WP_029103583.1	-	369	bacteria>proteobacteria>gammaproteobacteria	Moraxella caprae	hypothetical protein [Moraxella caprae].
WP_030740173.1	-	369	bacteria>actinobacteria	Streptomyces sp. NRRL F-5135	hypothetical protein [Streptomyces sp. NRRL F-5135].
WP_049236672.1	-	369	bacteria>proteobacteria>gammaproteobacteria	Moraxella catarrhalis	hypothetical protein [Moraxella catarrhalis].
WP_062501299.1	-	369	bacteria>proteobacteria>gammaproteobacteria	Moraxella lacunata	hypothetical protein [Moraxella lacunata].
WP_062534478.1	-	369	bacteria>proteobacteria>gammaproteobacteria	Psychrobacter urativorans	hypothetical protein [Psychrobacter urativorans].
WP_064604190.1	-	369	bacteria>proteobacteria>gammaproteobacteria	Moraxella catarrhalis	hypothetical protein [Moraxella catarrhalis].
WP_064607399.1	-	369	bacteria>proteobacteria>gammaproteobacteria	Moraxella catarrhalis	hypothetical protein [Moraxella catarrhalis].
WP_064611585.1	-	369	bacteria>proteobacteria>gammaproteobacteria	Moraxella catarrhalis	hypothetical protein [Moraxella catarrhalis].
WP_064619513.1	-	369	bacteria>proteobacteria>gammaproteobacteria	Moraxella catarrhalis	hypothetical protein [Moraxella catarrhalis].
WP_064625314.1	-	369	bacteria>proteobacteria>gammaproteobacteria	Moraxella catarrhalis	hypothetical protein [Moraxella catarrhalis].
WP_064636304.1	-	369	bacteria>proteobacteria>gammaproteobacteria	Moraxella catarrhalis	hypothetical protein [Moraxella catarrhalis].
WP_064644044.1	-	369	bacteria>proteobacteria>gammaproteobacteria	Moraxella catarrhalis	hypothetical protein [Moraxella catarrhalis].
WP_064646411.1	-	369	bacteria>proteobacteria>gammaproteobacteria	Moraxella catarrhalis	hypothetical protein [Moraxella catarrhalis].
WP_064647091.1	-	369	bacteria>proteobacteria>gammaproteobacteria	Moraxella catarrhalis	hypothetical protein [Moraxella catarrhalis].
WP_064653221.1	-	369	bacteria>proteobacteria>gammaproteobacteria	Moraxella catarrhalis	hypothetical protein [Moraxella catarrhalis].
OBX50702.1	A9Z65_05595	369	bacteria>proteobacteria>gammaproteobacteria	Moraxella nonliquefaciens	hypothetical protein A9Z65_05595 [Moraxella nonliquefaciens].
OBX50832.1	A9Z60_02775	369	bacteria>proteobacteria>gammaproteobacteria	Moraxella nonliquefaciens	hypothetical protein A9Z60_02775 [Moraxella nonliquefaciens].
WP_065256797.1	-	369	bacteria>proteobacteria>gammaproteobacteria	Moraxella lacunata	hypothetical protein [Moraxella lacunata].
WP_030353880.1	-	368	bacteria>actinobacteria	Streptomyces scopuliridis	hypothetical protein [Streptomyces scopuliridis].
WP_019957385.1	-	367	bacteria>proteobacteria>betaproteobacteria	Vitreoscilla stercoraria	hypothetical protein [Vitreoscilla stercoraria].
WP_033526245.1	-	367	bacteria>actinobacteria	Streptomyces galbus	chemotaxis protein [Streptomyces galbus].
WP_045555665.1	-	367	bacteria>actinobacteria	Streptomyces sp. FxanaA7	chemotaxis protein [Streptomyces sp. FxanaA7].
WP_046585373.1	-	367	bacteria>actinobacteria	Streptomyces mangrovisoli	chemotaxis protein [Streptomyces mangrovisoli].
WP_051313709.1	-	367	bacteria>bacteroidetes	Sporocytophaga myxococcoides	hypothetical protein [Sporocytophaga myxococcoides].
WP_060885881.1	-	367	bacteria>actinobacteria	Streptomyces sp. 96-12	hypothetical protein [Streptomyces sp. 96-12].
WP_018569429.1	-	366	bacteria>actinobacteria	Streptomyces sp. PsTaAH-124	hypothetical protein [Streptomyces sp. PsTaAH-124].
WP_019057861.1	-	366	bacteria>actinobacteria	Streptomyces prunicolor	chemotaxis protein [Streptomyces prunicolor].
WP_019985776.1	-	366	bacteria>actinobacteria	Streptomyces sp. Amel2xE9	hypothetical protein [Streptomyces sp. Amel2xE9].
WP_026411766.1	-	366	bacteria>actinobacteria	Actinomadura oligospora	hypothetical protein [Actinomadura oligospora].
WP_028423504.1	-	366	bacteria>actinobacteria	Streptomyces sp. GXT6	chemotaxis protein [Streptomyces sp. GXT6].
WP_030166165.1	-	366	bacteria>actinobacteria	Streptomyces sp. NRRL S-813	hypothetical protein [Streptomyces sp. NRRL S-813].
WP_033280703.1	-	366	bacteria>actinobacteria	Streptomyces sp. NRRL F-525	chemotaxis protein [Streptomyces sp. NRRL F-525].
WP_037740705.1	-	366	bacteria>actinobacteria	Streptomyces mirabilis	chemotaxis protein [Streptomyces mirabilis].
WP_037853945.1	-	366	bacteria>actinobacteria	Streptomyces sp. NRRL S-340	chemotaxis protein [Streptomyces sp. NRRL S-340].
WP_037896258.1	-	366	bacteria>actinobacteria	Streptomyces sp. Tu 6176	hypothetical protein [Streptomyces sp. Tu 6176].
WP_043666001.1	-	366	bacteria>actinobacteria	Streptomyces xylophagus	chemotaxis protein [Streptomyces xylophagus].
WP_059197018.1	-	366	bacteria>actinobacteria	Streptomyces antibioticus	hypothetical protein [Streptomyces antibioticus].
WP_063346065.1	-	366	bacteria>actinobacteria	Streptomyces sp. MJM8645	hypothetical protein [Streptomyces sp. MJM8645].
OAH12640.1	STSP_39860	366	bacteria>actinobacteria	Streptomyces sp. G25(2015)	hypothetical protein STSP_39860 [Streptomyces sp. G25(2015)].
WP_064531231.1	-	366	bacteria>actinobacteria	Streptomyces sp. SAT1	hypothetical protein [Streptomyces sp. SAT1].
WP_004003915.1	-	365	bacteria>actinobacteria	Streptomyces viridochromogenes	hypothetical protein [Streptomyces viridochromogenes].
WP_007502576.1	-	365	bacteria>actinobacteria	Streptomyces zinciresistens	hypothetical protein [Streptomyces zinciresistens].
WP_009306573.1	-	365	bacteria>actinobacteria	Streptomyces ipomoeae	hypothetical protein [Streptomyces ipomoeae].
WP_010982671.1	-	365	bacteria>actinobacteria	Streptomyces avermitilis	chemotaxis protein [Streptomyces avermitilis].
WP_014133369.1	-	365	bacteria>actinobacteria	Kitasatospora setae	hypothetical protein [Kitasatospora setae].
WP_015662896.1	-	365	bacteria>actinobacteria	Streptomyces davawensis	hypothetical protein [Streptomyces davawensis].
WP_016433296.1	-	365	bacteria>actinobacteria	Streptomyces sp. HGB0020	hypothetical protein [Streptomyces sp. HGB0020].
WP_018839571.1	-	365	bacteria>actinobacteria	Streptomyces sp. CNQ766	hypothetical protein [Streptomyces sp. CNQ766].
WP_018843254.1	-	365	bacteria>actinobacteria	Streptomyces	MULTISPECIES: hypothetical protein [Streptomyces].
WP_018854422.1	-	365	bacteria>actinobacteria	Streptomyces sp. CNY243	hypothetical protein [Streptomyces sp. CNY243].
WP_018890898.1	-	365	bacteria>actinobacteria	Streptomyces sp. CNT302	hypothetical protein [Streptomyces sp. CNT302].
WP_019069092.1	-	365	bacteria>actinobacteria	Streptomyces hokutonensis	chemotaxis protein [Streptomyces hokutonensis].
WP_020118047.1	-	365	bacteria>actinobacteria	Streptomyces canus	chemotaxis protein [Streptomyces canus].
WP_020138544.1	-	365	bacteria>actinobacteria	Streptomyces sp. 351MFTsu5.1	hypothetical protein [Streptomyces sp. 351MFTsu5.1].
WP_024936098.1	-	365	bacteria>actinobacteria	Actinomadura madurae	hypothetical protein [Actinomadura madurae].
WP_026165352.1	-	365	bacteria>actinobacteria	Streptomyces sp. CNS615	hypothetical protein [Streptomyces sp. CNS615].
WP_027732520.1	-	365	bacteria>actinobacteria	Streptomyces sp. CNR698	hypothetical protein [Streptomyces sp. CNR698].
WP_027740901.1	-	365	bacteria>actinobacteria	Streptomyces sp. CNT371	chemotaxis protein [Streptomyces sp. CNT371].
WP_027752490.1	-	365	bacteria>actinobacteria	Streptomyces sp. CNH099	hypothetical protein [Streptomyces sp. CNH099].
WP_027773351.1	-	365	bacteria>actinobacteria	Streptomyces sp. CNQ329	hypothetical protein [Streptomyces sp. CNQ329].
WP_030061342.1	-	365	bacteria>actinobacteria	Streptomyces	MULTISPECIES: hypothetical protein [Streptomyces].
WP_030145121.1	-	365	bacteria>actinobacteria	Spirillospora albida	hypothetical protein [Spirillospora albida].
WP_030244846.1	-	365	bacteria>actinobacteria	Streptomyces sp. NRRL S-350	hypothetical protein [Streptomyces sp. NRRL S-350].
WP_030290122.1	-	365	bacteria>actinobacteria	Streptomyces sp. NRRL B-5680	hypothetical protein [Streptomyces sp. NRRL B-5680].
WP_030324677.1	-	365	bacteria>actinobacteria	Streptomyces sp. NRRL B-3229	hypothetical protein [Streptomyces sp. NRRL B-3229].
WP_030395253.1	-	365	bacteria>actinobacteria	Streptomyces purpeofuscus	hypothetical protein [Streptomyces purpeofuscus].
WP_030410220.1	-	365	bacteria>actinobacteria	Streptomyces sp. NRRL S-1448	hypothetical protein [Streptomyces sp. NRRL S-1448].
WP_030464171.1	-	365	bacteria>actinobacteria	Kitasatospora sp. NRRL B-11411	hypothetical protein [Kitasatospora sp. NRRL B-11411].
WP_030557469.1	-	365	bacteria>actinobacteria	Streptomyces aureofaciens	hypothetical protein [Streptomyces aureofaciens].
WP_030585481.1	-	365	bacteria>actinobacteria	Streptomyces sclerotialus	chemotaxis protein [Streptomyces sclerotialus].
WP_030613874.1	-	365	bacteria>actinobacteria	Streptomyces fulvoviolaceus	chemotaxis protein [Streptomyces fulvoviolaceus].
WP_030676290.1	-	365	bacteria>actinobacteria	Streptomyces cellulosae	hypothetical protein [Streptomyces cellulosae].
WP_030787471.1	-	365	bacteria>actinobacteria	Streptomyces sp. NRRL F-5008	chemotaxis protein [Streptomyces sp. NRRL F-5008].
WP_030912145.1	-	365	bacteria>actinobacteria	Streptomyces sp. NRRL S-384	hypothetical protein [Streptomyces sp. NRRL S-384].
WP_030927226.1	-	365	bacteria>actinobacteria	Streptomyces sp. NRRL S-646	chemotaxis protein [Streptomyces sp. NRRL S-646].
WP_030946604.1	-	365	bacteria>actinobacteria	Streptomyces violaceoruber	hypothetical protein [Streptomyces violaceoruber].
WP_030951432.1	-	365	bacteria>actinobacteria	Streptomyces sp. NRRL F-5140	hypothetical protein [Streptomyces sp. NRRL F-5140].
WP_031045310.1	-	365	bacteria>actinobacteria	Streptomyces	MULTISPECIES: hypothetical protein [Streptomyces].
WP_031063389.1	-	365	bacteria>actinobacteria	Streptomyces sp. NRRL WC-3742	hypothetical protein [Streptomyces sp. NRRL WC-3742].
WP_031480151.1	-	365	bacteria>actinobacteria	Streptomyces bicolor	chemotaxis protein [Streptomyces bicolor].
WP_031513400.1	-	365	bacteria>actinobacteria	Streptomyces sp. NRRL F-5123	hypothetical protein [Streptomyces sp. NRRL F-5123].
WP_033174640.1	-	365	bacteria>actinobacteria	Streptomyces sp. URHA0041	chemotaxis protein [Streptomyces sp. URHA0041].
WP_033215442.1	-	365	bacteria>actinobacteria	Kitasatospora phosalacinea	hypothetical protein [Kitasatospora phosalacinea].
WP_033252155.1	-	365	bacteria>actinobacteria	Kitasatospora phosalacinea	hypothetical protein [Kitasatospora phosalacinea].
WP_034091421.1	-	365	bacteria>actinobacteria	Streptacidiphilus albus	hypothetical protein [Streptacidiphilus albus].
WP_037718553.1	-	365	bacteria>actinobacteria	Streptomyces mirabilis	chemotaxis protein [Streptomyces mirabilis].
WP_037748651.1	-	365	bacteria>actinobacteria	Streptomyces sp. CNQ-525	hypothetical protein [Streptomyces sp. CNQ-525].
WP_037915317.1	-	365	bacteria>actinobacteria	Streptomyces yeochonensis	hypothetical protein [Streptomyces yeochonensis].
WP_043446833.1	-	365	bacteria>actinobacteria	Streptomyces nodosus	hypothetical protein [Streptomyces nodosus].
WP_043465416.1	-	365	bacteria>actinobacteria	Kitasatospora sp. MBT66	hypothetical protein [Kitasatospora sp. MBT66].
WP_043907231.1	-	365	bacteria>actinobacteria	Kitasatospora griseola	hypothetical protein [Kitasatospora griseola].
WP_045940870.1	-	365	bacteria>actinobacteria	Streptomyces sp. NRRL S-495	hypothetical protein [Streptomyces sp. NRRL S-495].
WP_046259300.1	-	365	bacteria>actinobacteria	Streptomyces sp. WM6386	chemotaxis protein [Streptomyces sp. WM6386].
WP_047018590.1	-	365	bacteria>actinobacteria	Streptomyces sp. CNQ-509	hypothetical protein [Streptomyces sp. CNQ-509].
WP_048582007.1	-	365	bacteria>actinobacteria	Streptomyces viridochromogenes	chemotaxis protein [Streptomyces viridochromogenes].
CNG16913.1	ERS075342_06600	365	bacteria>actinobacteria	Mycobacterium tuberculosis	Uncharacterised protein [Mycobacterium tuberculosis].
WP_049659082.1	-	365	bacteria>actinobacteria	Kitasatospora sp. MY 5-36	hypothetical protein [Kitasatospora sp. MY 5-36].
WP_052854116.1	-	365	bacteria>actinobacteria	Streptomyces celluloflavus	chemotaxis protein [Streptomyces celluloflavus].
WP_053190142.1	-	365	bacteria>actinobacteria	Streptomyces resistomycificus	hypothetical protein [Streptomyces resistomycificus].
WP_053651213.1	-	365	bacteria>actinobacteria	Streptomyces sp. XY431	hypothetical protein [Streptomyces sp. XY431].
WP_053749745.1	-	365	bacteria>actinobacteria	Streptomyces sp. MMG1533	hypothetical protein [Streptomyces sp. MMG1533].
WP_053846850.1	-	365	bacteria>actinobacteria	Streptomyces sp. NRRL B-24085	hypothetical protein [Streptomyces sp. NRRL B-24085].
WP_054232917.1	-	365	bacteria>actinobacteria	Streptomyces diastatochromogenes	chemotaxis protein [Streptomyces diastatochromogenes].
WP_055520238.1	-	365	bacteria>actinobacteria	Streptomyces ossamyceticus	hypothetical protein [Streptomyces ossamyceticus].
WP_055540397.1	-	365	bacteria>actinobacteria	Streptomyces neyagawaensis	hypothetical protein [Streptomyces neyagawaensis].
WP_055610473.1	-	365	bacteria>actinobacteria	Streptomyces phaeochromogenes	hypothetical protein [Streptomyces phaeochromogenes].
WP_055619713.1	-	365	bacteria>actinobacteria	Streptomyces sp. JHA19	hypothetical protein [Streptomyces sp. JHA19].
WP_055711412.1	-	365	bacteria>actinobacteria	Streptomyces torulosus	hypothetical protein [Streptomyces torulosus].
WP_057611491.1	-	365	bacteria>actinobacteria	Streptomyces	MULTISPECIES: hypothetical protein [Streptomyces].
WP_058927232.1	-	365	bacteria>actinobacteria	Streptomyces sp. CdTB01	hypothetical protein [Streptomyces sp. CdTB01].
WP_059127954.1	-	365	bacteria>actinobacteria	Streptomyces sp. NRRL F-5122	hypothetical protein [Streptomyces sp. NRRL F-5122].
KUN42791.1	AQJ27_35225	365	bacteria>actinobacteria	Streptomyces olivochromogenes	chemotaxis protein [Streptomyces olivochromogenes].
KUO15603.1	AQJ91_40545	365	bacteria>actinobacteria	Streptomyces sp. RV15	chemotaxis protein [Streptomyces sp. RV15].
WP_062031976.1	-	365	bacteria>actinobacteria	Streptomyces phaeopurpureus	hypothetical protein [Streptomyces phaeopurpureus].
WP_062048483.1	-	365	bacteria>actinobacteria	Streptomyces ciscaucasicus	hypothetical protein [Streptomyces ciscaucasicus].
WP_062152286.1	-	365	bacteria>actinobacteria	Streptomyces curacoi	hypothetical protein [Streptomyces curacoi].
WP_062242265.1	-	365	bacteria>actinobacteria	Streptomyces griseorubiginosus	hypothetical protein [Streptomyces griseorubiginosus].
WP_062669649.1	-	365	bacteria>actinobacteria	Streptomyces	MULTISPECIES: hypothetical protein [Streptomyces].
WP_062697403.1	-	365	bacteria>actinobacteria	Streptomyces regalis	hypothetical protein [Streptomyces regalis].
WP_062717071.1	-	365	bacteria>actinobacteria	Streptomyces caeruleatus	hypothetical protein [Streptomyces caeruleatus].
ANS62787.1	SLINC_0563	365	bacteria>actinobacteria	Streptomyces lincolnensis	hypothetical protein SLINC_0563 [Streptomyces lincolnensis].
SCE03125.1	GA0115242_120111	365	bacteria>actinobacteria	Streptomyces sp. SolWspMP-5a-2	hypothetical protein GA0115242_120111 [Streptomyces sp. SolWspMP-5a-2].
ABA20724.1	Ava_1100	364	bacteria>cyanobacteria	Anabaena variabilis ATCC 29413	conserved hypothetical protein [Anabaena variabilis ATCC 29413].
WP_003971889.1	-	364	bacteria>actinobacteria	Streptomyces	MULTISPECIES: hypothetical protein [Streptomyces].
WP_003987983.1	-	364	bacteria>actinobacteria	Streptomyces viridochromogenes	hypothetical protein [Streptomyces viridochromogenes].
WP_004922006.1	-	364	bacteria>actinobacteria	Streptomyces griseoflavus	hypothetical protein [Streptomyces griseoflavus].
WP_004991816.1	-	364	bacteria>actinobacteria	Streptomyces	MULTISPECIES: hypothetical protein [Streptomyces].
WP_006133791.1	-	364	bacteria>actinobacteria	Streptomyces	MULTISPECIES: hypothetical protein [Streptomyces].
WP_007388069.1	-	364	bacteria>actinobacteria	Streptomyces	MULTISPECIES: hypothetical protein [Streptomyces].
WP_010033047.1	-	364	bacteria>actinobacteria	Streptomyces chartreusis	hypothetical protein [Streptomyces chartreusis].
WP_015038286.1	-	364	bacteria>actinobacteria	Streptomyces venezuelae	hypothetical protein [Streptomyces venezuelae].
WP_018530701.1	-	364	bacteria>actinobacteria	Streptomyces sp. HmicA12	hypothetical protein [Streptomyces sp. HmicA12].
WP_018654931.1	-	364	bacteria>actinobacteria	Actinomadura flavalba	hypothetical protein [Actinomadura flavalba].
WP_019327280.1	-	364	bacteria>actinobacteria	Streptomyces	MULTISPECIES: hypothetical protein [Streptomyces].
WP_019526799.1	-	364	bacteria>actinobacteria	Streptomyces	MULTISPECIES: hypothetical protein [Streptomyces].
WP_020126134.1	-	364	bacteria>actinobacteria	Streptomyces sp. 303MFCol5.2	hypothetical protein [Streptomyces sp. 303MFCol5.2].
WP_020938244.1	-	364	bacteria>actinobacteria	Streptomyces collinus	hypothetical protein [Streptomyces collinus].
WP_023553002.1	-	364	bacteria>actinobacteria	Streptomyces roseochromogenus	hypothetical protein [Streptomyces roseochromogenus].
WP_024756562.1	-	364	bacteria>actinobacteria	Streptomyces exfoliatus	hypothetical protein [Streptomyces exfoliatus].
WP_024885983.1	-	364	bacteria>actinobacteria	Streptomyces sp. CNH189	hypothetical protein [Streptomyces sp. CNH189].
WP_026405656.1	-	364	bacteria>actinobacteria	Actinomadura rifamycini	hypothetical protein [Actinomadura rifamycini].
WP_028799495.1	-	364	bacteria>actinobacteria	Streptomyces sp. 142MFCol3.1	chemotaxis protein [Streptomyces sp. 142MFCol3.1].
WP_029474890.1	-	364	bacteria>deinococcus-thermus>deinococci	Deinococcus frigens	hypothetical protein [Deinococcus frigens].
WP_030182489.1	-	364	bacteria>actinobacteria	Streptomyces violaceorubidus	hypothetical protein [Streptomyces violaceorubidus].
WP_030208788.1	-	364	bacteria>actinobacteria	Streptomyces griseoluteus	hypothetical protein [Streptomyces griseoluteus].
WP_030219068.1	-	364	bacteria>actinobacteria	Streptomyces bikiniensis	hypothetical protein [Streptomyces bikiniensis].
WP_030222939.1	-	364	bacteria>actinobacteria	Streptomyces sp. NRRL WC-3626	hypothetical protein [Streptomyces sp. NRRL WC-3626].
WP_030230777.1	-	364	bacteria>actinobacteria	Streptomyces	MULTISPECIES: hypothetical protein [Streptomyces].
WP_030312945.1	-	364	bacteria>actinobacteria	Streptomyces flavochromogenes	hypothetical protein [Streptomyces flavochromogenes].
WP_030339589.1	-	364	bacteria>actinobacteria	Streptomyces sp. NRRL S-1022	hypothetical protein [Streptomyces sp. NRRL S-1022].
WP_030403849.1	-	364	bacteria>actinobacteria	Streptomyces	MULTISPECIES: hypothetical protein [Streptomyces].
WP_030419184.1	-	364	bacteria>actinobacteria	Streptomyces sp. NRRL F-5065	chemotaxis protein [Streptomyces sp. NRRL F-5065].
WP_030545365.1	-	364	bacteria>actinobacteria	Streptomyces exfoliatus	hypothetical protein [Streptomyces exfoliatus].
WP_030602848.1	-	364	bacteria>actinobacteria	Streptomyces achromogenes	hypothetical protein [Streptomyces achromogenes].
WP_030644304.1	-	364	bacteria>actinobacteria	Streptomyces	MULTISPECIES: hypothetical protein [Streptomyces].
WP_030688950.1	-	364	bacteria>actinobacteria	Streptomyces globisporus	hypothetical protein [Streptomyces globisporus].
WP_030736754.1	-	364	bacteria>actinobacteria	Streptomyces sp. NRRL S-31	chemotaxis protein [Streptomyces sp. NRRL S-31].
WP_030752114.1	-	364	bacteria>actinobacteria	Streptomyces griseus	hypothetical protein [Streptomyces griseus].
WP_030783907.1	-	364	bacteria>actinobacteria	Streptomyces lavenduligriseus	hypothetical protein [Streptomyces lavenduligriseus].
WP_030832037.1	-	364	bacteria>actinobacteria	Streptomyces	MULTISPECIES: hypothetical protein [Streptomyces].
WP_030844278.1	-	364	bacteria>actinobacteria	Streptomyces sp. NRRL S-475	hypothetical protein [Streptomyces sp. NRRL S-475].
WP_030862674.1	-	364	bacteria>actinobacteria	Streptomyces sp. NRRL S-37	chemotaxis protein [Streptomyces sp. NRRL S-37].
WP_030866855.1	-	364	bacteria>actinobacteria	Streptomyces violaceoruber	hypothetical protein [Streptomyces violaceoruber].
WP_030976480.1	-	364	bacteria>actinobacteria	Streptomyces sp. NRRL F-4835	hypothetical protein [Streptomyces sp. NRRL F-4835].
WP_030990491.1	-	364	bacteria>actinobacteria	Streptomyces sp. NRRL WC-3744	hypothetical protein [Streptomyces sp. NRRL WC-3744].
WP_031003770.1	-	364	bacteria>actinobacteria	Streptomyces sp. NRRL F-5727	hypothetical protein [Streptomyces sp. NRRL F-5727].
WP_031016737.1	-	364	bacteria>actinobacteria	Streptomyces sp. NRRL S-1314	hypothetical protein [Streptomyces sp. NRRL S-1314].
WP_031021086.1	-	364	bacteria>actinobacteria	Streptomyces sp. NRRL WC-3795	hypothetical protein [Streptomyces sp. NRRL WC-3795].
WP_031025733.1	-	364	bacteria>actinobacteria	Streptomyces sp. NRRL WC-3725	hypothetical protein [Streptomyces sp. NRRL WC-3725].
WP_031034388.1	-	364	bacteria>actinobacteria	Streptomyces sp. NRRL F-5650	hypothetical protein [Streptomyces sp. NRRL F-5650].
WP_031081341.1	-	364	bacteria>actinobacteria	Streptomyces	MULTISPECIES: hypothetical protein [Streptomyces].
WP_031097025.1	-	364	bacteria>actinobacteria	Streptomyces	MULTISPECIES: hypothetical protein [Streptomyces].
WP_031102189.1	-	364	bacteria>actinobacteria	Streptomyces sp. NRRL WC-3683	hypothetical protein [Streptomyces sp. NRRL WC-3683].
WP_031134687.1	-	364	bacteria>actinobacteria	Streptomyces sp. NRRL WC-3719	hypothetical protein [Streptomyces sp. NRRL WC-3719].
WP_031141389.1	-	364	bacteria>actinobacteria	Streptomyces	MULTISPECIES: hypothetical protein [Streptomyces].
WP_031162700.1	-	364	bacteria>actinobacteria	Streptomyces durhamensis	hypothetical protein [Streptomyces durhamensis].
WP_033212218.1	-	364	bacteria>actinobacteria	Streptomyces	MULTISPECIES: hypothetical protein [Streptomyces].
WP_033276457.1	-	364	bacteria	Actinobacteria	MULTISPECIES: hypothetical protein [Actinobacteria].
WP_033309462.1	-	364	bacteria>actinobacteria	Streptomyces iakyrus	hypothetical protein [Streptomyces iakyrus].
WP_037638095.1	-	364	bacteria>actinobacteria	Streptomyces griseorubens	hypothetical protein [Streptomyces griseorubens].
WP_037655651.1	-	364	bacteria>actinobacteria	Streptomyces griseofuscus	chemotaxis protein [Streptomyces griseofuscus].
WP_037682070.1	-	364	bacteria>actinobacteria	Streptomyces griseus	chemotaxis protein [Streptomyces griseus].
WP_037758193.1	-	364	bacteria>actinobacteria	Streptomyces olindensis	hypothetical protein [Streptomyces olindensis].
WP_037939042.1	-	364	bacteria>actinobacteria	Streptomyces toyocaensis	hypothetical protein [Streptomyces toyocaensis].
WP_039652197.1	-	364	bacteria>actinobacteria	Streptomyces	MULTISPECIES: hypothetical protein [Streptomyces].
WP_041127317.1	-	364	bacteria>actinobacteria	Streptomyces vietnamensis	hypothetical protein [Streptomyces vietnamensis].
WP_041664965.1	-	364	bacteria>actinobacteria	Streptomyces hygroscopicus	chemotaxis protein [Streptomyces hygroscopicus].
WP_042172407.1	-	364	bacteria>actinobacteria	Streptomyces	MULTISPECIES: hypothetical protein [Streptomyces].
WP_043229968.1	-	364	bacteria>actinobacteria	Streptomyces sp. NRRL F-5193	hypothetical protein [Streptomyces sp. NRRL F-5193].
WP_043376091.1	-	364	bacteria>actinobacteria	Streptomyces mutabilis	hypothetical protein [Streptomyces mutabilis].
WP_046426207.1	-	364	bacteria>actinobacteria	Streptomyces sp. MUSC136T	hypothetical protein [Streptomyces sp. MUSC136T].
WP_046731243.1	-	364	bacteria>actinobacteria	Streptomyces sp. MUSC119T	chemotaxis protein [Streptomyces sp. MUSC119T].
WP_046907778.1	-	364	bacteria>actinobacteria	Streptomyces showdoensis	chemotaxis protein [Streptomyces showdoensis].
AKJ15013.1	ABB07_34635	364	bacteria>actinobacteria	Streptomyces incarnatus	chemotaxis protein [Streptomyces incarnatus].
AKN73143.1	QR97_28325	364	bacteria>actinobacteria	Streptomyces sp. PBH53	chemotaxis protein [Streptomyces sp. PBH53].
WP_048456563.1	-	364	bacteria>actinobacteria	Streptomyces sp. HNS054	hypothetical protein [Streptomyces sp. HNS054].
WP_052837269.1	-	364	bacteria>actinobacteria	Streptomyces aureofaciens	hypothetical protein [Streptomyces aureofaciens].
WP_053127109.1	-	364	bacteria>actinobacteria	Streptomyces ambofaciens	hypothetical protein [Streptomyces ambofaciens].
WP_053636078.1	-	364	bacteria>actinobacteria	Streptomyces sp. XY152	hypothetical protein [Streptomyces sp. XY152].
WP_053640752.1	-	364	bacteria>actinobacteria	Streptomyces	MULTISPECIES: chemotaxis protein [Streptomyces].
WP_053659807.1	-	364	bacteria>actinobacteria	Streptomyces sp. MMG1121	hypothetical protein [Streptomyces sp. MMG1121].
WP_053668086.1	-	364	bacteria>actinobacteria	Streptomyces sp. NRRL B-1140	hypothetical protein [Streptomyces sp. NRRL B-1140].
WP_053709419.1	-	364	bacteria>actinobacteria	Streptomyces sp. NRRL B-3648	hypothetical protein [Streptomyces sp. NRRL B-3648].
WP_053761819.1	-	364	bacteria>actinobacteria	Streptomyces sp. AS58	hypothetical protein [Streptomyces sp. AS58].
WP_053913103.1	-	364	bacteria>actinobacteria	Streptomyces sp. TP-A0875	chemotaxis protein [Streptomyces sp. TP-A0875].
WP_054240828.1	-	364	bacteria>actinobacteria	Actinobacteria bacterium OV320	hypothetical protein [Actinobacteria bacterium OV320].
WP_055420389.1	-	364	bacteria>actinobacteria	Streptomyces pactum	hypothetical protein [Streptomyces pactum].
WP_055468098.1	-	364	bacteria>actinobacteria	Streptomyces sp. NBRC 110030	hypothetical protein [Streptomyces sp. NBRC 110030].
WP_055546957.1	-	364	bacteria>actinobacteria	Streptomyces kanamyceticus	hypothetical protein [Streptomyces kanamyceticus].
WP_055572926.1	-	364	bacteria>actinobacteria	Streptomyces emeiensis	hypothetical protein [Streptomyces emeiensis].
WP_055597058.1	-	364	bacteria>actinobacteria	Streptomyces hirsutus	hypothetical protein [Streptomyces hirsutus].
WP_055602809.1	-	364	bacteria>actinobacteria	Streptomyces aureus	hypothetical protein [Streptomyces aureus].
WP_055609387.1	-	364	bacteria>actinobacteria	Streptomyces bambergiensis	hypothetical protein [Streptomyces bambergiensis].
WP_055630869.1	-	364	bacteria>actinobacteria	Streptomyces cyanoalbus	hypothetical protein [Streptomyces cyanoalbus].
WP_055638598.1	-	364	bacteria>actinobacteria	Streptomyces griseoruber	hypothetical protein [Streptomyces griseoruber].
WP_055640912.1	-	364	bacteria>actinobacteria	Streptomyces venezuelae	hypothetical protein [Streptomyces venezuelae].
WP_055692089.1	-	364	bacteria>actinobacteria	Streptomyces prasinus	hypothetical protein [Streptomyces prasinus].
WP_055693641.1	-	364	bacteria>actinobacteria	Streptomyces prasinopilosus	hypothetical protein [Streptomyces prasinopilosus].
WP_055704622.1	-	364	bacteria>actinobacteria	Streptomyces puniciscabiei	hypothetical protein [Streptomyces puniciscabiei].
WP_056557530.1	-	364	bacteria>actinobacteria	Streptomyces	MULTISPECIES: hypothetical protein [Streptomyces].
WP_056648902.1	-	364	bacteria>actinobacteria	Streptomyces sp. Root431	hypothetical protein [Streptomyces sp. Root431].
WP_057581696.1	-	364	bacteria>actinobacteria	Streptomyces sp. Root264	hypothetical protein [Streptomyces sp. Root264].
WP_057605194.1	-	364	bacteria>actinobacteria	Streptomyces sp. Root1310	hypothetical protein [Streptomyces sp. Root1310].
KUM95514.1	AQI88_15625	364	bacteria>actinobacteria	Streptomyces cellostaticus	chemotaxis protein [Streptomyces cellostaticus].
KUN05912.1	AQI95_13285	364	bacteria>actinobacteria	Streptomyces yokosukanensis	chemotaxis protein [Streptomyces yokosukanensis].
KUN41707.1	AQJ30_02130	364	bacteria>actinobacteria	Streptomyces longwoodensis	chemotaxis protein [Streptomyces longwoodensis].
KUO07418.1	AQJ58_35720	364	bacteria>actinobacteria	Streptomyces sp. DSM 15324	chemotaxis protein [Streptomyces sp. DSM 15324].
WP_059264302.1	-	364	bacteria>actinobacteria	Streptomyces corchorusii	hypothetical protein [Streptomyces corchorusii].
WP_059297302.1	-	364	bacteria>actinobacteria	Streptomyces canus	hypothetical protein [Streptomyces canus].
WP_059415522.1	-	364	bacteria>actinobacteria	Streptomyces azureus	hypothetical protein [Streptomyces azureus].
WP_061446270.1	-	364	bacteria>actinobacteria	Streptomyces sp. CCM_MD2014	hypothetical protein [Streptomyces sp. CCM_MD2014].
WP_061917036.1	-	364	bacteria>actinobacteria	Streptomyces bungoensis	hypothetical protein [Streptomyces bungoensis].
WP_062646024.1	-	364	bacteria>actinobacteria	Streptomyces sp. NBRC 110468	hypothetical protein [Streptomyces sp. NBRC 110468].
WP_062754619.1	-	364	bacteria>actinobacteria	Streptomyces sp. WAC04657	hypothetical protein [Streptomyces sp. WAC04657].
WP_062771157.1	-	364	bacteria>actinobacteria	Streptomyces sp. NRRL S-1521	hypothetical protein [Streptomyces sp. NRRL S-1521].
WP_062924984.1	-	364	bacteria>actinobacteria	Streptomyces sp. S10(2016)	hypothetical protein [Streptomyces sp. S10(2016)].
BAU81806.1	SLA_0852	364	bacteria>actinobacteria	Streptomyces laurentii	methyl-accepting chemotaxis protein [Streptomyces laurentii].
WP_063481304.1	-	364	bacteria>actinobacteria	Streptomyces ambofaciens	hypothetical protein [Streptomyces ambofaciens].
OAA97412.1	A6P39_27675	364	bacteria>actinobacteria	Streptomyces sp. FXJ1.172	chemotaxis protein [Streptomyces sp. FXJ1.172].
WP_064726317.1	-	364	bacteria>actinobacteria	Streptomyces parvulus	hypothetical protein [Streptomyces parvulus].
WP_065002457.1	-	364	bacteria>actinobacteria	Streptomyces sp. H-KF8	chemotaxis protein [Streptomyces sp. H-KF8].
SBT91115.1	GA0115233_102655	364	bacteria>actinobacteria	Streptomyces sp. DI166	hypothetical protein GA0115233_102655 [Streptomyces sp. DI166].
ANP52824.1	AVL59_27700	364	bacteria>actinobacteria	Streptomyces griseochromogenes	chemotaxis protein [Streptomyces griseochromogenes].
SCD39702.1	GA0115238_10714	364	bacteria>actinobacteria	Streptomyces sp. di50b	hypothetical protein GA0115238_10714 [Streptomyces sp. di50b].
SCF68688.1	GA0115258_109016	364	bacteria>actinobacteria	Streptomyces sp. LamerLS-31b	hypothetical protein GA0115258_109016 [Streptomyces sp. LamerLS-31b].
WP_002662816.1	-	363	bacteria>bacteroidetes	Bergeyella zoohelcum	hypothetical protein [Bergeyella zoohelcum].
WP_002688916.1	-	363	bacteria>bacteroidetes	Bergeyella zoohelcum	hypothetical protein [Bergeyella zoohelcum].
WP_004638825.1	-	363	bacteria>proteobacteria>gammaproteobacteria	Acinetobacter haemolyticus	hypothetical protein [Acinetobacter haemolyticus].
WP_004670803.1	-	363	bacteria>proteobacteria>gammaproteobacteria	Acinetobacter sp. CIP-A165	hypothetical protein [Acinetobacter sp. CIP-A165].
WP_005081001.1	-	363	bacteria>proteobacteria>gammaproteobacteria	Acinetobacter haemolyticus	hypothetical protein [Acinetobacter haemolyticus].
WP_005196693.1	-	363	bacteria>proteobacteria>gammaproteobacteria	Acinetobacter sp. NIPH 298	hypothetical protein [Acinetobacter sp. NIPH 298].
WP_008941513.1	-	363	bacteria>proteobacteria>gammaproteobacteria	Acinetobacter	MULTISPECIES: hypothetical protein [Acinetobacter].
EFG65701.1	SSBG_06533	362	bacteria>actinobacteria	Streptomyces sp. SPB074	LOW QUALITY PROTEIN: conserved hypothetical protein, partial [Streptomyces sp. SPB074].
WP_009116649.1	-	362	bacteria>proteobacteria>betaproteobacteria	Neisseria wadsworthii	hypothetical protein [Neisseria wadsworthii].
WP_009120165.1	-	362	bacteria>proteobacteria>betaproteobacteria	Neisseria shayeganii	hypothetical protein [Neisseria shayeganii].
WP_033361464.1	-	362	bacteria>actinobacteria	Dactylosporangium aurantiacum	hypothetical protein [Dactylosporangium aurantiacum].
WP_038032904.1	-	362	bacteria>bacteroidetes	Thermonema rossianum	hypothetical protein [Thermonema rossianum].
WP_054600343.1	-	362	bacteria>proteobacteria>betaproteobacteria	Neisseria sp. 74A18	hypothetical protein [Neisseria sp. 74A18].
WP_054618485.1	-	362	bacteria>proteobacteria>betaproteobacteria	Neisseria sp. 83E34	hypothetical protein [Neisseria sp. 83E34].
WP_005405906.1	-	361	bacteria>proteobacteria>gammaproteobacteria	Acinetobacter radioresistens	hypothetical protein [Acinetobacter radioresistens].
WP_011956789.1	-	361	bacteria>chloroflexi	Roseiflexus sp. RS-1	hypothetical protein [Roseiflexus sp. RS-1].
WP_012121513.1	-	361	bacteria>chloroflexi	Roseiflexus castenholzii	hypothetical protein [Roseiflexus castenholzii].
WP_014434231.1	-	361	bacteria>chloroflexi	Caldilinea aerophila	hypothetical protein [Caldilinea aerophila].
OAN43901.1	A6A03_17560	361	bacteria>chloroflexi	Chloroflexus sp. isl-2	hypothetical protein A6A03_17560 [Chloroflexus sp. isl-2].
WP_039727821.1	-	360	bacteria	Cyanobacteria	MULTISPECIES: hypothetical protein [Cyanobacteria].
WP_064970415.1	-	360	bacteria>bacteroidetes	Riemerella anatipestifer	hypothetical protein [Riemerella anatipestifer].
WP_002752236.1	-	359	bacteria>cyanobacteria	Microcystis aeruginosa	hypothetical protein [Microcystis aeruginosa].
WP_002762020.1	-	359	bacteria>cyanobacteria	Microcystis aeruginosa	hypothetical protein [Microcystis aeruginosa].
WP_002777171.1	-	359	bacteria>cyanobacteria	Microcystis aeruginosa	hypothetical protein [Microcystis aeruginosa].
WP_002795232.1	-	359	bacteria>cyanobacteria	Microcystis aeruginosa	hypothetical protein [Microcystis aeruginosa].
WP_004163194.1	-	359	bacteria>cyanobacteria	Microcystis aeruginosa	hypothetical protein [Microcystis aeruginosa].
WP_006843806.1	-	359	bacteria>bacteroidetes	Dysgonomonas mossii	hypothetical protein [Dysgonomonas mossii].
WP_008196944.1	-	359	bacteria>cyanobacteria	Microcystis sp. T1-4	hypothetical protein [Microcystis sp. T1-4].
WP_010049985.1	-	359	bacteria>firmicutes	Carnobacterium maltaromaticum	hypothetical protein [Carnobacterium maltaromaticum].
WP_013597201.1	-	359	bacteria>bacteroidetes	Weeksella virosa	hypothetical protein [Weeksella virosa].
WP_015075247.1	-	359	bacteria>firmicutes	Carnobacterium maltaromaticum	hypothetical protein [Carnobacterium maltaromaticum].
WP_016514823.1	-	359	bacteria>cyanobacteria	Microcystis aeruginosa	hypothetical protein [Microcystis aeruginosa].
WP_017651532.1	-	359	bacteria>cyanobacteria	Microchaete sp. PCC 7126	hypothetical protein [Microchaete sp. PCC 7126].
WP_019597862.1	-	359	bacteria>bacteroidetes	Rhodonellum psychrophilum	hypothetical protein [Rhodonellum psychrophilum].
WP_024969023.1	-	359	bacteria>cyanobacteria	Microcystis aeruginosa	hypothetical protein [Microcystis aeruginosa].
WP_027377702.1	-	359	bacteria>bacteroidetes	Chryseobacterium palustre	hypothetical protein [Chryseobacterium palustre].
WP_029904820.1	-	359	bacteria>bacteroidetes	Prevotella sp. 10(H)	hypothetical protein [Prevotella sp. 10(H)].
CDT17287.1	BN1088_1500140	359	bacteria>bacteroidetes	Sphingobacterium sp. PM2-P1-29	conserved hypothetical protein [Sphingobacterium sp. PM2-P1-29].
WP_032130747.1	-	359	bacteria>bacteroidetes	Weeksella sp. FF8	hypothetical protein [Weeksella sp. FF8].
WP_034685781.1	-	359	bacteria>bacteroidetes	Chryseobacterium piperi	hypothetical protein [Chryseobacterium piperi].
WP_034869605.1	-	359	bacteria>bacteroidetes	Elizabethkingia	MULTISPECIES: hypothetical protein [Elizabethkingia].
WP_039366944.1	-	359	bacteria>bacteroidetes	Chryseobacterium taiwanense	hypothetical protein [Chryseobacterium taiwanense].
WP_045359406.1	-	359	bacteria>cyanobacteria	Microcystis aeruginosa	hypothetical protein [Microcystis aeruginosa].
WP_046676294.1	-	359	bacteria>bacteroidetes	Sphingobacterium sp. Ag1	hypothetical protein [Sphingobacterium sp. Ag1].
WP_047440789.1	-	359	bacteria>bacteroidetes	Chryseobacterium sp. YR561	hypothetical protein [Chryseobacterium sp. YR561].
KXK47198.1	UZ05_CHB002002266	359	bacteria>chlorobi	Chlorobi bacterium OLB5	hypothetical protein UZ05_CHB002002266 [Chlorobi bacterium OLB5].
WP_061430541.1	-	359	bacteria>cyanobacteria	Microcystis aeruginosa	hypothetical protein [Microcystis aeruginosa].
WP_009769177.1	-	358	bacteria>cyanobacteria	Oscillatoriales cyanobacterium JSC-12	hypothetical protein [Oscillatoriales cyanobacterium JSC-12].
WP_010871775.1	-	358	bacteria>cyanobacteria	Synechocystis sp. PCC 6803	hypothetical protein [Synechocystis sp. PCC 6803].
WP_015125294.1	-	358	bacteria>cyanobacteria	Synechococcus sp. PCC 6312	hypothetical protein [Synechococcus sp. PCC 6312].
WP_017296569.1	-	358	bacteria>cyanobacteria	Nodosilinea nodulosa	hypothetical protein [Nodosilinea nodulosa].
WP_017300960.1	-	358	bacteria>cyanobacteria	Nodosilinea nodulosa	hypothetical protein [Nodosilinea nodulosa].
WP_021071507.1	-	358	bacteria>bacteroidetes	Sphingobacterium paucimobilis	hypothetical protein [Sphingobacterium paucimobilis].
WP_026735055.1	-	358	bacteria>cyanobacteria	Fischerella sp. PCC 9605	hypothetical protein [Fischerella sp. PCC 9605].
WP_028947326.1	-	358	bacteria>cyanobacteria	Synechocystis sp. PCC 6714	hypothetical protein [Synechocystis sp. PCC 6714].
WP_036010697.1	-	358	bacteria>cyanobacteria	Leptolyngbya sp. JSC-1	hypothetical protein [Leptolyngbya sp. JSC-1].
WP_052429895.1	-	358	bacteria>bacteroidetes	Sporocytophaga myxococcoides	hypothetical protein [Sporocytophaga myxococcoides].
WP_058883364.1	-	358	bacteria>cyanobacteria	Oscillatoriales cyanobacterium MTP1	hypothetical protein [Oscillatoriales cyanobacterium MTP1].
KXK19264.1	UZ08_BCD001001299	358	bacteria>bacteroidetes	Bacteroidetes bacterium OLB8	hypothetical protein UZ08_BCD001001299 [Bacteroidetes bacterium OLB8].
BAU63367.1	STA3757_07310	358	bacteria>cyanobacteria	Stanieria sp. NIES-3757	hypothetical protein STA3757_07310 [Stanieria sp. NIES-3757].
AEY87477.1	SHJG_2202	357	bacteria>actinobacteria	Streptomyces hygroscopicus subsp. jinggangensis 5008	hypothetical protein SHJG_2202 [Streptomyces hygroscopicus subsp. jinggangensis 5008].
WP_002734163.1	-	357	bacteria>cyanobacteria	Microcystis aeruginosa	hypothetical protein [Microcystis aeruginosa].
WP_002746833.1	-	357	bacteria>cyanobacteria	Microcystis aeruginosa	hypothetical protein [Microcystis aeruginosa].
WP_002765735.1	-	357	bacteria>cyanobacteria	Microcystis aeruginosa	hypothetical protein [Microcystis aeruginosa].
WP_002785969.1	-	357	bacteria>cyanobacteria	Microcystis aeruginosa	hypothetical protein [Microcystis aeruginosa].
WP_019973861.1	-	357	bacteria>bacteroidetes	Empedobacter brevis	hypothetical protein [Empedobacter brevis].
WP_045079969.1	-	357	bacteria>bacteroidetes	Vitellibacter vladivostokensis	hypothetical protein [Vitellibacter vladivostokensis].
WP_052275332.1	-	357	bacteria>cyanobacteria	Microcystis panniformis	hypothetical protein [Microcystis panniformis].
WP_062619225.1	-	357	bacteria>bacteroidetes	Vitellibacter sp. D-24	hypothetical protein [Vitellibacter sp. D-24].
WP_010736694.1	-	356	bacteria>firmicutes	Enterococcus	MULTISPECIES: hypothetical protein [Enterococcus].
WP_019723591.1	-	356	bacteria>firmicutes	Enterococcus mundtii	hypothetical protein [Enterococcus mundtii].
WP_034687014.1	-	356	bacteria>firmicutes	Enterococcus mundtii	hypothetical protein [Enterococcus mundtii].
WP_065096051.1	-	356	bacteria>firmicutes	Enterococcus mundtii	hypothetical protein [Enterococcus mundtii].
WP_050708965.1	-	355	bacteria>bacteroidetes	Dysgonomonas sp. HGC4	hypothetical protein [Dysgonomonas sp. HGC4].
KUK60622.1	XD81_0066	354	bacteria>bacteroidetes	Bacteroidetes bacterium 38_7	hypothetical protein XD81_0066 [Bacteroidetes bacterium 38_7].
KXK39940.1	UZ09_BCD002000623	352	bacteria>bacteroidetes	Bacteroidetes bacterium OLB9	hypothetical protein UZ09_BCD002000623 [Bacteroidetes bacterium OLB9].
GAT83479.1	STXM2123_4180	343	bacteria>actinobacteria	Streptomyces sp. F-3	chemotaxis protein [Streptomyces sp. F-3].
EPJ42639.1	STAFG_0305	341	bacteria>actinobacteria	Streptomyces afghaniensis 772	hypothetical protein STAFG_0305 [Streptomyces afghaniensis 772].
WP_034721277.1	-	339	bacteria>bacteroidetes	Chryseobacterium antarcticum	hypothetical protein [Chryseobacterium antarcticum].
WP_034259997.1	-	338	bacteria>bacteroidetes	Aequorivita capsosiphonis	hypothetical protein [Aequorivita capsosiphonis].
KJJ39178.1	MB09_05965	338	bacteria>bacteroidetes	Vitellibacter vladivostokensis	hypothetical protein MB09_05965 [Vitellibacter vladivostokensis].
KXO01414.1	LS48_01405	338	bacteria>bacteroidetes	Vitellibacter sp. D-24	hypothetical protein LS48_01405 [Vitellibacter sp. D-24].
WP_008636601.1	-	337	bacteria>bacteroidetes	Bizionia argentinensis	hypothetical protein [Bizionia argentinensis].
WP_008638952.1	-	337	bacteria>bacteroidetes	Bizionia argentinensis	hypothetical protein [Bizionia argentinensis].
WP_027126386.1	-	337	bacteria>bacteroidetes	Gelidibacter mesophilus	hypothetical protein [Gelidibacter mesophilus].
WP_036099128.1	-	325	bacteria>firmicutes	Listeria riparia	hypothetical protein [Listeria riparia].
WP_055591141.1	-	322	bacteria>actinobacteria	Streptomyces griseoplanus	hypothetical protein [Streptomyces griseoplanus].
KKX51440.1	L950_0205245	297	bacteria>bacteroidetes	Sphingobacterium sp. IITKGP-BTPF85	hypothetical protein L950_0205245 [Sphingobacterium sp. IITKGP-BTPF85].
KKX34810.1	UZ02_AOB001002599	296	bacteria>proteobacteria>betaproteobacteria	Nitrosomonas europaea	hypothetical protein UZ02_AOB001002599, partial [Nitrosomonas europaea].
WP_003673572.1	-	266	bacteria>proteobacteria>gammaproteobacteria	Moraxella catarrhalis	hypothetical protein [Moraxella catarrhalis].
WP_036335250.1	-	266	bacteria>proteobacteria>gammaproteobacteria	Moraxella catarrhalis	hypothetical protein [Moraxella catarrhalis].
WP_036336282.1	-	266	bacteria>proteobacteria>gammaproteobacteria	Moraxella catarrhalis	hypothetical protein [Moraxella catarrhalis].
WP_036344558.1	-	266	bacteria>proteobacteria>gammaproteobacteria	Moraxella catarrhalis	hypothetical protein [Moraxella catarrhalis].
WP_036350618.1	-	266	bacteria>proteobacteria>gammaproteobacteria	Moraxella catarrhalis	hypothetical protein [Moraxella catarrhalis].
WP_036378294.1	-	266	bacteria>proteobacteria>gammaproteobacteria	Moraxella catarrhalis	hypothetical protein [Moraxella catarrhalis].
WP_038520294.1	-	266	bacteria>proteobacteria>gammaproteobacteria	Moraxella catarrhalis	hypothetical protein [Moraxella catarrhalis].
WP_042510522.1	-	266	bacteria>proteobacteria>gammaproteobacteria	Moraxella catarrhalis	hypothetical protein [Moraxella catarrhalis].
WP_049148550.1	-	266	bacteria>proteobacteria>gammaproteobacteria	Moraxella catarrhalis	hypothetical protein [Moraxella catarrhalis].
WP_049156707.1	-	266	bacteria>proteobacteria>gammaproteobacteria	Moraxella catarrhalis	hypothetical protein [Moraxella catarrhalis].
WP_049158073.1	-	266	bacteria>proteobacteria>gammaproteobacteria	Moraxella catarrhalis	hypothetical protein [Moraxella catarrhalis].
WP_049167033.1	-	266	bacteria>proteobacteria>gammaproteobacteria	Moraxella catarrhalis	hypothetical protein [Moraxella catarrhalis].
WP_065248956.1	-	266	bacteria>proteobacteria>gammaproteobacteria	Moraxella catarrhalis	hypothetical protein [Moraxella catarrhalis].
KKT02298.1	UV79_C0001G0057	264	bacteria	candidate division TM6 bacterium GW2011_GWF2_43_17	hypothetical protein UV79_C0001G0057, partial [candidate division TM6 bacterium GW2011_GWF2_43_17].
EGE27628.1	EA1_01632	260	bacteria>proteobacteria>gammaproteobacteria	Moraxella catarrhalis O35E	hypothetical protein EA1_01632, partial [Moraxella catarrhalis O35E].
WP_041786591.1	-	255	bacteria>proteobacteria>gammaproteobacteria	Moraxella catarrhalis	hypothetical protein [Moraxella catarrhalis].
WP_011633796.1	-	208	bacteria>proteobacteria>betaproteobacteria	Nitrosomonas eutropha	hypothetical protein [Nitrosomonas eutropha].
ENX50356.1	F943_00218	178	bacteria>proteobacteria>gammaproteobacteria	Acinetobacter ursingii NIPH 706	hypothetical protein F943_00218 [Acinetobacter ursingii NIPH 706].

4. family 5

##;baeRF1 family 5 contains an N-terminal fusion to the codon recognition domain but lacks the C-
##;terminal fusion to the Pelota domain.

277;erf1_1+RNaseH_RF_fam3

ALS76126.1	AUC31_13405	287	bacteria>firmicutes	Planococcus rifietoensis	antiporter [Planococcus rifietoensis].
KYG59700.1	AY633_05510	287	bacteria>firmicutes	Planococcus maritimus	antiporter [Planococcus maritimus].
EHA30743.1	BSSC8_23280	279	bacteria>firmicutes	Bacillus subtilis subsp. subtilis str. SC-8	hypothetical protein BSSC8_23280 [Bacillus subtilis subsp. subtilis str. SC-8].
AGE63745.1	C663_1965	279	bacteria>firmicutes	Bacillus subtilis XF-1	hypothetical protein C663_1965 [Bacillus subtilis XF-1].
CCU58589.1	BSUBE1_1958	279	bacteria>firmicutes	Bacillus subtilis E1	hypothetical protein BSUBE1_1958 [Bacillus subtilis E1].
WP_033543965.1	-	279	bacteria>firmicutes	Planococcus sp. CAU13	hypothetical protein [Planococcus sp. CAU13].
WP_036805576.1	-	279	bacteria>firmicutes	Planomicrobium glaciei	hypothetical protein [Planomicrobium glaciei].
WP_053165253.1	-	279	bacteria>firmicutes	Planomicrobium glaciei	hypothetical protein [Planomicrobium glaciei].
KZD95281.1	B4122_0253	279	bacteria>firmicutes	Bacillus subtilis	hypothetical protein B4122_0253 [Bacillus subtilis].
WP_006830895.1	-	278	bacteria>firmicutes	Planococcus antarcticus	hypothetical protein [Planococcus antarcticus].
WP_006835520.1	-	278	bacteria>firmicutes	Bacillus sp. SG-1	hypothetical protein [Bacillus sp. SG-1].
WP_049693047.1	-	278	bacteria>firmicutes	Planococcus sp. L10.15	hypothetical protein [Planococcus sp. L10.15].
WP_052650536.1	-	278	bacteria>firmicutes	Planomicrobium sp. ES2	hypothetical protein [Planomicrobium sp. ES2].
WP_065536555.1	-	278	bacteria>firmicutes	Planococcus antarcticus	antiporter [Planococcus antarcticus].
WP_008430324.1	-	274	bacteria>firmicutes	Planococcus donghaensis	hypothetical protein [Planococcus donghaensis].
WP_008496761.1	-	274	bacteria>firmicutes	Planococcus halocryophilus	hypothetical protein [Planococcus halocryophilus].
WP_038704840.1	-	274	bacteria>firmicutes	Planococcus sp. PAMC 21323	hypothetical protein [Planococcus sp. PAMC 21323].
WP_058384332.1	-	274	bacteria>firmicutes	Planococcus kocurii	hypothetical protein [Planococcus kocurii].
WP_065525408.1	-	274	bacteria>firmicutes	Planococcus donghaensis	antiporter [Planococcus donghaensis].
WP_008587173.1	-	270	bacteria>firmicutes	Salimicrobium jeotgali	hypothetical protein [Salimicrobium jeotgali].
WP_027964361.1	-	270	bacteria>firmicutes	Halalkalibacillus halophilus	hypothetical protein [Halalkalibacillus halophilus].
KXH81873.1	AU377_06310	270	bacteria>firmicutes	Sporosarcina sp. HYO08	antiporter [Sporosarcina sp. HYO08].
WP_008635773.1	-	269	bacteria>firmicutes	Bacillaceae	MULTISPECIES: hypothetical protein [Bacillaceae].
WP_014644186.1	-	269	bacteria>firmicutes	Halobacillus halophilus	hypothetical protein [Halobacillus halophilus].
WP_018393449.1	-	269	bacteria>firmicutes	Bacillus sp. 37MA	hypothetical protein [Bacillus sp. 37MA].
WP_019391897.1	-	269	bacteria>firmicutes	Bacillus endophyticus	hypothetical protein [Bacillus endophyticus].
WP_034290333.1	-	269	bacteria>firmicutes	Bacillus sp. 72	hypothetical protein [Bacillus sp. 72].
WP_040056624.1	-	269	bacteria>firmicutes	Bacillus	MULTISPECIES: hypothetical protein [Bacillus].
WP_061801657.1	-	269	bacteria>firmicutes	Bacillus endophyticus	hypothetical protein [Bacillus endophyticus].
WP_062321265.1	-	269	bacteria>firmicutes	Halolactibacillus sp. JCM 19043	hypothetical protein [Halolactibacillus sp. JCM 19043].
WP_063965856.1	-	269	bacteria>firmicutes	[Bacillus] aminovorans	hypothetical protein [[Bacillus] aminovorans].
WP_063975653.1	-	269	bacteria>firmicutes	[Bacillus] aminovorans	hypothetical protein [[Bacillus] aminovorans].
WP_010198551.1	-	268	bacteria>firmicutes	Bacillus sp. m3-13	hypothetical protein [Bacillus sp. m3-13].
WP_010647400.1	-	268	bacteria>firmicutes	Oceanobacillus massiliensis	hypothetical protein [Oceanobacillus massiliensis].
WP_018933121.1	-	268	bacteria>firmicutes	Gracilibacillus lacisalsi	hypothetical protein [Gracilibacillus lacisalsi].
WP_019378679.1	-	268	bacteria>firmicutes	Virgibacillus halodenitrificans	hypothetical protein [Virgibacillus halodenitrificans].
CDQ30747.1	BN993_00107	268	bacteria>firmicutes	Virgibacillus halodenitrificans	hypothetical protein BN993_00107 [Virgibacillus halodenitrificans].
WP_027956395.1	-	268	bacteria>firmicutes	Halobacillus kuroshimensis	hypothetical protein [Halobacillus kuroshimensis].
WP_028783625.1	-	268	bacteria>firmicutes	Thalassobacillus devorans	hypothetical protein [Thalassobacillus devorans].
WP_035505884.1	-	268	bacteria>firmicutes	Halobacillus	MULTISPECIES: hypothetical protein [Halobacillus].
WP_035541946.1	-	268	bacteria>firmicutes	Halobacillus sp. BBL2006	hypothetical protein [Halobacillus sp. BBL2006].
WP_040955967.1	-	268	bacteria>firmicutes	Virgibacillus sp. SK37	hypothetical protein [Virgibacillus sp. SK37].
WP_040978966.1	-	268	bacteria>firmicutes	Oceanobacillus sp. S5	hypothetical protein [Oceanobacillus sp. S5].
WP_042530528.1	-	268	bacteria>firmicutes	Oceanobacillus oncorhynchi	hypothetical protein [Oceanobacillus oncorhynchi].
WP_053073815.1	-	268	bacteria>firmicutes	Bacillus sp. LL01	hypothetical protein [Bacillus sp. LL01].
ALC81294.1	AM592_06585	268	bacteria>firmicutes	Bacillus gobiensis	hypothetical protein AM592_06585 [Bacillus gobiensis].
WP_058308091.1	-	268	bacteria>firmicutes	Gracilibacillus sp. Awa-1	hypothetical protein [Gracilibacillus sp. Awa-1].
WP_010532190.1	-	267	bacteria>firmicutes	Lentibacillus jeotgali	hypothetical protein [Lentibacillus jeotgali].
WP_011066790.1	-	267	bacteria>firmicutes	Oceanobacillus iheyensis	hypothetical protein [Oceanobacillus iheyensis].
WP_017187124.1	-	267	bacteria>firmicutes	Alkalibacillus haloalkaliphilus	hypothetical protein [Alkalibacillus haloalkaliphilus].
WP_017797515.1	-	267	bacteria>firmicutes	Oceanobacillus kimchii	hypothetical protein [Oceanobacillus kimchii].
WP_017814740.1	-	267	bacteria>firmicutes	Paenibacillus sp. A9	hypothetical protein [Paenibacillus sp. A9].
WP_026569537.1	-	267	bacteria>firmicutes	Bacillaceae	MULTISPECIES: hypothetical protein [Bacillaceae].
WP_026799921.1	-	267	bacteria>firmicutes	Pontibacillus halophilus	hypothetical protein [Pontibacillus halophilus].
WP_026906743.1	-	267	bacteria>firmicutes	Paucisalibacillus globulus	hypothetical protein [Paucisalibacillus globulus].
WP_029268791.1	-	267	bacteria>firmicutes	Virgibacillus alimentarius	hypothetical protein [Virgibacillus alimentarius].
WP_036782448.1	-	267	bacteria>firmicutes	Pontibacillus chungwhensis	hypothetical protein [Pontibacillus chungwhensis].
WP_036819698.1	-	267	bacteria>firmicutes	Pontibacillus yanchengensis	hypothetical protein [Pontibacillus yanchengensis].
WP_046212617.1	-	267	bacteria>firmicutes	Paenibacillus wulumuqiensis	hypothetical protein [Paenibacillus wulumuqiensis].
WP_046227507.1	-	267	bacteria>firmicutes	Paenibacillus dauci	hypothetical protein [Paenibacillus dauci].
CQR46523.1	BN1058_00788	267	bacteria>firmicutes	Paraliobacillus sp. PM-2	hypothetical protein BN1058_00788 [Paraliobacillus sp. PM-2].
WP_047986390.1	-	267	bacteria>firmicutes	Ornithinibacillus californiensis	hypothetical protein [Ornithinibacillus californiensis].
WP_053218730.1	-	267	bacteria>firmicutes	Virgibacillus sp. SK-1	hypothetical protein [Virgibacillus sp. SK-1].
ALX47149.1	AOX59_00155	267	bacteria>firmicutes	Lentibacillus amyloliquefaciens	hypothetical protein AOX59_00155 [Lentibacillus amyloliquefaciens].
WP_060531082.1	-	267	bacteria>firmicutes	Paenibacillus bovis	hypothetical protein [Paenibacillus bovis].
WP_062440982.1	-	267	bacteria>firmicutes	Thalassobacillus sp. TM-1	hypothetical protein [Thalassobacillus sp. TM-1].
WP_010278484.1	-	266	bacteria>firmicutes	Paenibacillus senegalensis	hypothetical protein [Paenibacillus senegalensis].
WP_013487415.1	-	266	bacteria>firmicutes	Bacillus cellulosilyticus	hypothetical protein [Bacillus cellulosilyticus].
WP_017470514.1	-	266	bacteria>firmicutes	Amphibacillus jilinensis	hypothetical protein [Amphibacillus jilinensis].
WP_018923390.1	-	266	bacteria>firmicutes	Salsuginibacillus kocurii	hypothetical protein [Salsuginibacillus kocurii].
WP_022795063.1	-	266	bacteria>firmicutes	Marinococcus halotolerans	hypothetical protein [Marinococcus halotolerans].
WP_026703358.1	-	266	bacteria>firmicutes	Bacillus aidingensis	hypothetical protein [Bacillus aidingensis].
WP_027445992.1	-	266	bacteria>firmicutes	Pontibacillus marinus	hypothetical protein [Pontibacillus marinus].
WP_028559922.1	-	266	bacteria>firmicutes	Paenibacillus pinihumi	hypothetical protein [Paenibacillus pinihumi].
WP_032085586.1	-	266	bacteria>firmicutes	Bacillus aquimaris	hypothetical protein [Bacillus aquimaris].
WP_034761166.1	-	266	bacteria>firmicutes	Bacillus vietnamensis	hypothetical protein [Bacillus vietnamensis].
WP_040977696.1	-	266	bacteria>firmicutes	Oceanobacillus sp. S5	hypothetical protein [Oceanobacillus sp. S5].
WP_044339179.1	-	266	bacteria>firmicutes	Bacillus aquimaris	hypothetical protein [Bacillus aquimaris].
WP_058299009.1	-	266	bacteria>firmicutes	Bacillus enclensis	hypothetical protein [Bacillus enclensis].
WP_060671452.1	-	266	bacteria>firmicutes	Bacillus vietnamensis	hypothetical protein [Bacillus vietnamensis].
WP_018883877.1	-	265	bacteria>firmicutes	Paenibacillus massiliensis	hypothetical protein [Paenibacillus massiliensis].
WP_025676793.1	-	265	bacteria>firmicutes	Paenibacillus massiliensis	hypothetical protein [Paenibacillus massiliensis].
WP_026696903.1	-	265	bacteria>firmicutes	Bacillus chagannorensis	hypothetical protein [Bacillus chagannorensis].
WP_028590679.1	-	265	bacteria>firmicutes	Paenibacillus panacisoli	hypothetical protein [Paenibacillus panacisoli].
WP_048313587.1	-	265	bacteria>firmicutes	Anaerobacillus macyae	hypothetical protein [Anaerobacillus macyae].
WP_009498569.1	-	264	bacteria>firmicutes	Sporosarcina newyorkensis	hypothetical protein [Sporosarcina newyorkensis].
WP_016427365.1	-	264	bacteria>firmicutes	Paenisporosarcina sp. HGH0030	hypothetical protein [Paenisporosarcina sp. HGH0030].
WP_017378614.1	-	264	bacteria>firmicutes	Paenisporosarcina sp. TG-14	hypothetical protein [Paenisporosarcina sp. TG-14].
WP_017727926.1	-	264	bacteria>firmicutes	Bacillus ligniniphilus	hypothetical protein [Bacillus ligniniphilus].
WP_019413503.1	-	264	bacteria>firmicutes	Paenisporosarcina sp. TG20	hypothetical protein [Paenisporosarcina sp. TG20].
WP_026690953.1	-	264	bacteria>firmicutes	Bacillus aurantiacus	hypothetical protein [Bacillus aurantiacus].
WP_029054952.1	-	264	bacteria>firmicutes	Sporosarcina ureae	hypothetical protein [Sporosarcina ureae].
WP_038190323.1	-	264	bacteria>firmicutes	Viridibacillus arenosi	hypothetical protein [Viridibacillus arenosi].
WP_040286433.1	-	264	bacteria>firmicutes	Sporosarcina koreensis	hypothetical protein [Sporosarcina koreensis].
WP_041074327.1	-	264	bacteria>firmicutes	Bacillus sp. OxB-1	hypothetical protein [Bacillus sp. OxB-1].
WP_042360948.1	-	264	bacteria>firmicutes	Geomicrobium sp. JCM 19055	hypothetical protein [Geomicrobium sp. JCM 19055].
WP_042420737.1	-	264	bacteria>firmicutes	Geomicrobium sp. JCM 19038	hypothetical protein [Geomicrobium sp. JCM 19038].
WP_045850483.1	-	264	bacteria>firmicutes	Domibacillus enclensis	hypothetical protein [Domibacillus enclensis].
WP_046178983.1	-	264	bacteria>firmicutes	Domibacillus tundrae	hypothetical protein [Domibacillus tundrae].
WP_048006777.1	-	264	bacteria>firmicutes	Bacillus marisflavi	hypothetical protein [Bacillus marisflavi].
WP_048012218.1	-	264	bacteria>firmicutes	Bacillus marisflavi	hypothetical protein [Bacillus marisflavi].
WP_048014755.1	-	264	bacteria>firmicutes	Bacillus marisflavi	hypothetical protein [Bacillus marisflavi].
WP_053417137.1	-	264	bacteria>firmicutes	Viridibacillus arvi	hypothetical protein [Viridibacillus arvi].
WP_053428773.1	-	264	bacteria>firmicutes	Bacillus marisflavi	hypothetical protein [Bacillus marisflavi].
WP_056532902.1	-	264	bacteria>firmicutes	Bacillus sp. Leaf406	hypothetical protein [Bacillus sp. Leaf406].
WP_060210150.1	-	264	bacteria>firmicutes	Sporosarcina koreensis	hypothetical protein [Sporosarcina koreensis].
WP_013173675.1	-	263	bacteria>firmicutes	[Bacillus] selenitireducens	hypothetical protein [[Bacillus] selenitireducens].
WP_017547947.1	-	263	bacteria>firmicutes	Salinicoccus carnicancri	hypothetical protein [Salinicoccus carnicancri].
WP_025784308.1	-	263	bacteria>firmicutes	Sporosarcina sp. D27	hypothetical protein [Sporosarcina sp. D27].
WP_026859025.1	-	263	bacteria>firmicutes	Jeotgalicoccus psychrophilus	hypothetical protein [Jeotgalicoccus psychrophilus].
WP_026867044.1	-	263	bacteria>firmicutes	Jeotgalicoccus marinus	hypothetical protein [Jeotgalicoccus marinus].
WP_035418385.1	-	263	bacteria>firmicutes	Bacillus sp. JCM 19045	hypothetical protein [Bacillus sp. JCM 19045].
WP_035810348.1	-	263	bacteria>firmicutes	Jeotgalicoccus sp. 13MG44_air	hypothetical protein [Jeotgalicoccus sp. 13MG44_air].
WP_036574900.1	-	263	bacteria>firmicutes	Oceanobacillus picturae	hypothetical protein [Oceanobacillus picturae].
WP_038479249.1	-	263	bacteria>firmicutes	Bacillus	MULTISPECIES: hypothetical protein [Bacillus].
WP_039044207.1	-	263	bacteria>firmicutes	Sporosarcina sp. ZBG7A	hypothetical protein [Sporosarcina sp. ZBG7A].
WP_042222614.1	-	263	bacteria>firmicutes	Oceanobacillus manasiensis	hypothetical protein [Oceanobacillus manasiensis].
WP_046517142.1	-	263	bacteria>firmicutes	Salinicoccus sediminis	hypothetical protein [Salinicoccus sediminis].
WP_046789462.1	-	263	bacteria>firmicutes	Salinicoccus halodurans	hypothetical protein [Salinicoccus halodurans].
WP_052254586.1	-	263	bacteria>firmicutes	Salinicoccus sp. YB14-2	hypothetical protein [Salinicoccus sp. YB14-2].
ALA51037.1	DB29_00209	263	bacteria>firmicutes	Bacillus clausii	hypothetical protein DB29_00209 [Bacillus clausii].
WP_053590784.1	-	263	bacteria>firmicutes	Bacillus sp. FJAT-22090	hypothetical protein [Bacillus sp. FJAT-22090].
WP_056828607.1	-	263	bacteria>firmicutes	Psychrobacillus sp. FJAT-21963	hypothetical protein [Psychrobacillus sp. FJAT-21963].
WP_058950542.1	-	263	bacteria>firmicutes	Oceanobacillus picturae	hypothetical protein [Oceanobacillus picturae].
AMQ06947.1	AZE41_13935	263	bacteria>firmicutes	Sporosarcina psychrophila	antiporter [Sporosarcina psychrophila].
WP_062747145.1	-	263	bacteria>firmicutes	Bacillus clausii	hypothetical protein [Bacillus clausii].
WP_064094259.1	-	263	bacteria>firmicutes	Bacillus aquimaris	hypothetical protein [Bacillus aquimaris].
WP_003325815.1	-	262	bacteria>firmicutes	Bacillus subtilis group	MULTISPECIES: hypothetical protein [Bacillus subtilis group].
WP_010788910.1	-	262	bacteria>firmicutes	Bacillus atrophaeus	hypothetical protein [Bacillus atrophaeus].
WP_020618433.1	-	262	bacteria>firmicutes	Paenibacillus daejeonensis	hypothetical protein [Paenibacillus daejeonensis].
WP_024534793.1	-	262	bacteria>firmicutes	Sporosarcina sp. EUR3 2.2.2	hypothetical protein [Sporosarcina sp. EUR3 2.2.2].
WP_031547455.1	-	262	bacteria>firmicutes	Salinicoccus luteus	hypothetical protein [Salinicoccus luteus].
WP_040105059.1	-	262	bacteria>firmicutes	Salinicoccus roseus	hypothetical protein [Salinicoccus roseus].
WP_046559682.1	-	262	bacteria>firmicutes	Bacillus	MULTISPECIES: hypothetical protein [Bacillus].
WP_061573117.1	-	262	bacteria>firmicutes	Bacillus vallismortis	hypothetical protein [Bacillus vallismortis].
WP_061670197.1	-	262	bacteria>firmicutes	Bacillus atrophaeus	hypothetical protein [Bacillus atrophaeus].
WP_063562508.1	-	262	bacteria>firmicutes	Bacillus horikoshii	hypothetical protein [Bacillus horikoshii].
WP_063638414.1	-	262	bacteria>firmicutes	Bacillus atrophaeus	hypothetical protein [Bacillus atrophaeus].
WP_064098736.1	-	262	bacteria>firmicutes	Bacillus horikoshii	hypothetical protein [Bacillus horikoshii].
WP_002506133.1	-	261	bacteria>firmicutes	Staphylococcus	MULTISPECIES: hypothetical protein [Staphylococcus].
WP_002511928.1	-	261	bacteria>firmicutes	Staphylococcus equorum	hypothetical protein [Staphylococcus equorum].
WP_007407989.1	-	261	bacteria>firmicutes	Bacillus	MULTISPECIES: hypothetical protein [Bacillus].
WP_012117737.1	-	261	bacteria>firmicutes	Bacillus subtilis group	MULTISPECIES: hypothetical protein [Bacillus subtilis group].
WP_012959230.1	-	261	bacteria>firmicutes	Bacillus pseudofirmus	hypothetical protein [Bacillus pseudofirmus].
WP_015417618.1	-	261	bacteria>firmicutes	Bacillus velezensis	host attachment protein [Bacillus velezensis].
WP_017723593.1	-	261	bacteria>firmicutes	Staphylococcus xylosus	hypothetical protein [Staphylococcus xylosus].
WP_020955929.1	-	261	bacteria>firmicutes	Bacillus velezensis	host attachment protein [Bacillus velezensis].
WP_021339934.1	-	261	bacteria>firmicutes	Staphylococcus equorum	hypothetical protein [Staphylococcus equorum].
WP_022628039.1	-	261	bacteria>firmicutes	Bacillus marmarensis	hypothetical protein [Bacillus marmarensis].
WP_029378781.1	-	261	bacteria>firmicutes	Staphylococcus xylosus	hypothetical protein [Staphylococcus xylosus].
WP_034631544.1	-	261	bacteria>firmicutes	Bacillus okhensis	hypothetical protein [Bacillus okhensis].
WP_035664684.1	-	261	bacteria>firmicutes	Bacillus akibai	hypothetical protein [Bacillus akibai].
WP_039067140.1	-	261	bacteria>firmicutes	Staphylococcus xylosus	hypothetical protein [Staphylococcus xylosus].
WP_047172966.1	-	261	bacteria>firmicutes	Staphylococcus xylosus	hypothetical protein [Staphylococcus xylosus].
WP_053573308.1	-	261	bacteria>firmicutes	Bacillus amyloliquefaciens	hypothetical protein [Bacillus amyloliquefaciens].
WP_057511436.1	-	261	bacteria>firmicutes	Staphylococcus sp. NAM3COL9	hypothetical protein [Staphylococcus sp. NAM3COL9].
WP_060675205.1	-	261	bacteria>firmicutes	Bacillus amyloliquefaciens	hypothetical protein [Bacillus amyloliquefaciens].
WP_061581514.1	-	261	bacteria>firmicutes	Bacillus amyloliquefaciens	hypothetical protein [Bacillus amyloliquefaciens].
ANB83996.1	A6R78_08260	261	bacteria>firmicutes	Bacillus velezensis	hypothetical protein A6R78_08260 [Bacillus velezensis].
WP_064107650.1	-	261	bacteria>firmicutes	Bacillus velezensis	hypothetical protein [Bacillus velezensis].
WP_065337183.1	-	261	bacteria>firmicutes	Staphylococcus equorum	hypothetical protein [Staphylococcus equorum].
WP_003153800.1	-	260	bacteria>firmicutes	Bacillus subtilis group	MULTISPECIES: hypothetical protein [Bacillus subtilis group].
WP_003220152.1	-	260	bacteria>firmicutes	Bacillus	MULTISPECIES: hypothetical protein [Bacillus].
WP_003241677.1	-	260	bacteria>firmicutes	Bacillus subtilis	hypothetical protein [Bacillus subtilis].
WP_004399450.1	-	260	bacteria>firmicutes	Bacillus	MULTISPECIES: hypothetical protein [Bacillus].
WP_007611947.1	-	260	bacteria>firmicutes	Bacillus	MULTISPECIES: hypothetical protein [Bacillus].
WP_010330561.1	-	260	bacteria>firmicutes	Bacillus vallismortis	hypothetical protein [Bacillus vallismortis].
WP_013352512.1	-	260	bacteria>firmicutes	Bacillus amyloliquefaciens	hypothetical protein [Bacillus amyloliquefaciens].
WP_014114068.1	-	260	bacteria>firmicutes	Bacillales	MULTISPECIES: hypothetical protein [Bacillales].
WP_014305188.1	-	260	bacteria>firmicutes	Bacillus subtilis group	MULTISPECIES: hypothetical protein [Bacillus subtilis group].
WP_014418106.1	-	260	bacteria>firmicutes	Bacillus	MULTISPECIES: hypothetical protein [Bacillus].
WP_014470308.1	-	260	bacteria>firmicutes	Bacillus subtilis group	MULTISPECIES: hypothetical protein [Bacillus subtilis group].
WP_014477060.1	-	260	bacteria>firmicutes	Bacillus subtilis	hypothetical protein [Bacillus subtilis].
WP_014664252.1	-	260	bacteria>firmicutes	Bacillus sp. JS	hypothetical protein [Bacillus sp. JS].
WP_015239998.1	-	260	bacteria>firmicutes	Bacillus	MULTISPECIES: hypothetical protein [Bacillus].
WP_015251912.1	-	260	bacteria>firmicutes	Bacillus	MULTISPECIES: hypothetical protein [Bacillus].
WP_016936590.1	-	260	bacteria>firmicutes	Bacillus siamensis	hypothetical protein [Bacillus siamensis].
WP_017417957.1	-	260	bacteria>firmicutes	Bacillus subtilis group	MULTISPECIES: hypothetical protein [Bacillus subtilis group].
WP_017697172.1	-	260	bacteria>firmicutes	Bacillus subtilis	hypothetical protein [Bacillus subtilis].
WP_019258640.1	-	260	bacteria>firmicutes	Bacillus subtilis	hypothetical protein [Bacillus subtilis].
WP_021288990.1	-	260	bacteria>firmicutes	Virgibacillus sp. CM-4	hypothetical protein [Virgibacillus sp. CM-4].
WP_021493901.1	-	260	bacteria>firmicutes	Bacillus amyloliquefaciens	hypothetical protein [Bacillus amyloliquefaciens].
WP_024085466.1	-	260	bacteria>firmicutes	Bacillus subtilis group	MULTISPECIES: hypothetical protein [Bacillus subtilis group].
WP_024121709.1	-	260	bacteria>firmicutes	Bacillus mojavensis	hypothetical protein [Bacillus mojavensis].
WP_024573290.1	-	260	bacteria>firmicutes	Bacillus subtilis	hypothetical protein [Bacillus subtilis].
WP_024715398.1	-	260	bacteria>firmicutes	Bacillus tequilensis	hypothetical protein [Bacillus tequilensis].
WP_025852293.1	-	260	bacteria>firmicutes	Bacillus velezensis	hypothetical protein [Bacillus velezensis].
WP_026680017.1	-	260	bacteria>firmicutes	Bacillus megaterium	hypothetical protein [Bacillus megaterium].
WP_029441315.1	-	260	bacteria>firmicutes	Bacillus mojavensis	hypothetical protein [Bacillus mojavensis].
KFF56322.1	CM50_10300	260	bacteria>firmicutes	Bacillus subtilis	hypothetical protein CM50_10300 [Bacillus subtilis].
WP_032873440.1	-	260	bacteria>firmicutes	Bacillus subtilis group	MULTISPECIES: hypothetical protein [Bacillus subtilis group].
WP_033574656.1	-	260	bacteria>firmicutes	Bacillus amyloliquefaciens	hypothetical protein [Bacillus amyloliquefaciens].
WP_033884029.1	-	260	bacteria>firmicutes	Bacillus subtilis	hypothetical protein [Bacillus subtilis].
WP_038243073.1	-	260	bacteria>firmicutes	Virgibacillus sp. Vm-5	hypothetical protein [Virgibacillus sp. Vm-5].
WP_038458412.1	-	260	bacteria>firmicutes	Bacillus subtilis group	MULTISPECIES: hypothetical protein [Bacillus subtilis group].
WP_038829953.1	-	260	bacteria>firmicutes	Bacillus	MULTISPECIES: hypothetical protein [Bacillus].
WP_039074166.1	-	260	bacteria>firmicutes	Bacillus sp. MSP13	hypothetical protein [Bacillus sp. MSP13].
WP_039254902.1	-	260	bacteria>firmicutes	Bacillus sp. Pc3	hypothetical protein [Bacillus sp. Pc3].
WP_040082906.1	-	260	bacteria>firmicutes	Bacillus sp. A053	hypothetical protein [Bacillus sp. A053].
WP_040238049.1	-	260	bacteria>firmicutes	Bacillus subtilis group	MULTISPECIES: hypothetical protein [Bacillus subtilis group].
WP_041339832.1	-	260	bacteria>firmicutes	Bacillus subtilis	hypothetical protein [Bacillus subtilis].
WP_043857669.1	-	260	bacteria>firmicutes	Bacillus subtilis	hypothetical protein [Bacillus subtilis].
WP_044155575.1	-	260	bacteria>firmicutes	Salinibacillus aidingensis	hypothetical protein [Salinibacillus aidingensis].
WP_044445272.1	-	260	bacteria>firmicutes	Bacillus subtilis	hypothetical protein [Bacillus subtilis].
KJD57999.1	UZ38_10355	260	bacteria>firmicutes	Bacillus amyloliquefaciens	hypothetical protein UZ38_10355 [Bacillus amyloliquefaciens].
KJJ42508.1	UM89_05745	260	bacteria>firmicutes	Bacillus subtilis	hypothetical protein UM89_05745 [Bacillus subtilis].
WP_045207703.1	-	260	bacteria>firmicutes	Bacillus velezensis	hypothetical protein [Bacillus velezensis].
WP_045511356.1	-	260	bacteria>firmicutes	Bacillus amyloliquefaciens	hypothetical protein [Bacillus amyloliquefaciens].

WP_045926179.1	-	260	bacteria>firmicutes	Bacillus siamensis	hypothetical protein [Bacillus siamensis].
WP_046381115.1	-	260	bacteria>firmicutes	Bacillus sp. LM 4-2	hypothetical protein [Bacillus sp. LM 4-2].
WP_047182730.1	-	260	bacteria>firmicutes	Bacillus subtilis	hypothetical protein [Bacillus subtilis].
WP_047474650.1	-	260	bacteria>firmicutes	Bacillus amyloliquefaciens	hypothetical protein [Bacillus amyloliquefaciens].
WP_049628618.1	-	260	bacteria>firmicutes	Bacillus	MULTISPECIES: hypothetical protein [Bacillus].
WP_050352404.1	-	260	bacteria>firmicutes	Virgibacillus pantothenticus	hypothetical protein [Virgibacillus pantothenticus].
WP_052586090.1	-	260	bacteria>firmicutes	Bacillus sp.	hypothetical protein [Bacillus sp.].
WP_052827540.1	-	260	bacteria>firmicutes	Bacillus amyloliquefaciens	hypothetical protein [Bacillus amyloliquefaciens].
WP_059336562.1	-	260	bacteria>firmicutes	[Brevibacterium] halotolerans	hypothetical protein [[Brevibacterium] halotolerans].
WP_059352501.1	-	260	bacteria>firmicutes	Bacillus axarquiensis	hypothetical protein [Bacillus axarquiensis].
WP_060398816.1	-	260	bacteria>firmicutes	Bacillus subtilis	hypothetical protein [Bacillus subtilis].
WP_060562878.1	-	260	bacteria>firmicutes	Bacillus velezensis	hypothetical protein [Bacillus velezensis].
WP_061188159.1	-	260	bacteria>firmicutes	Bacillus subtilis	hypothetical protein [Bacillus subtilis].
WP_061521108.1	-	260	bacteria>firmicutes	Bacillus nakamurai	hypothetical protein [Bacillus nakamurai].
WP_063335161.1	-	260	bacteria>firmicutes	Bacillus subtilis	hypothetical protein [Bacillus subtilis].
WP_064671115.1	-	260	bacteria>firmicutes	Bacillus subtilis	hypothetical protein [Bacillus subtilis].
WP_064814984.1	-	260	bacteria>firmicutes	Bacillus subtilis	hypothetical protein [Bacillus subtilis].
WP_065180604.1	-	260	bacteria>firmicutes	Bacillus velezensis	hypothetical protein [Bacillus velezensis].
WP_065521111.1	-	260	bacteria>firmicutes	Bacillus amyloliquefaciens	hypothetical protein [Bacillus amyloliquefaciens].
WP_065981654.1	-	260	bacteria>firmicutes	Bacillus amyloliquefaciens	hypothetical protein [Bacillus amyloliquefaciens].
WP_012727194.1	-	259	bacteria>firmicutes	Exiguobacterium sp. AT1b	hypothetical protein [Exiguobacterium sp. AT1b].
WP_016508317.1	-	259	bacteria>firmicutes	Exiguobacterium sp. S17	hypothetical protein [Exiguobacterium sp. S17].
WP_020007853.1	-	259	bacteria>firmicutes	Salinicoccus albus	hypothetical protein [Salinicoccus albus].
WP_024370669.1	-	259	bacteria>firmicutes	Exiguobacterium sp. 8-11-1	hypothetical protein [Exiguobacterium sp. 8-11-1].
WP_024371138.1	-	259	bacteria>firmicutes	Exiguobacterium sp. 8-11-1	hypothetical protein [Exiguobacterium sp. 8-11-1].
WP_029334206.1	-	259	bacteria>firmicutes	Exiguobacterium aurantiacum	hypothetical protein [Exiguobacterium aurantiacum].
WP_034778915.1	-	259	bacteria>firmicutes	Exiguobacterium mexicanum	hypothetical protein [Exiguobacterium mexicanum].
WP_034804251.1	-	259	bacteria>firmicutes	Exiguobacterium sp. AB2	hypothetical protein [Exiguobacterium sp. AB2].
WP_034817615.1	-	259	bacteria>firmicutes	Exiguobacterium alkaliphilum	hypothetical protein [Exiguobacterium alkaliphilum].
WP_047374679.1	-	259	bacteria>firmicutes	Exiguobacterium sp. ZOR0005	hypothetical protein [Exiguobacterium sp. ZOR0005].
WP_047374959.1	-	259	bacteria>firmicutes	Exiguobacterium sp. ZOR0005	hypothetical protein [Exiguobacterium sp. ZOR0005].
WP_016509584.1	-	258	bacteria>firmicutes	Exiguobacterium sp. S17	hypothetical protein [Exiguobacterium sp. S17].
WP_021066389.1	-	258	bacteria>firmicutes	Exiguobacterium pavilionensis	hypothetical protein [Exiguobacterium pavilionensis].
WP_038566347.1	-	258	bacteria>firmicutes	Terribacillus aidingensis	hypothetical protein [Terribacillus aidingensis].
WP_059293265.1	-	258	bacteria>firmicutes	Bacillus malacitensis	hypothetical protein [Bacillus malacitensis].
WP_026672725.1	-	257	bacteria>firmicutes	Bacillus bogoriensis	hypothetical protein [Bacillus bogoriensis].
ALS75789.1	AUC31_11535	256	bacteria>firmicutes	Planococcus rifietoensis	hypothetical protein AUC31_11535 [Planococcus rifietoensis].
WP_026828446.1	-	250	bacteria>firmicutes	Exiguobacterium sibiricum	hypothetical protein [Exiguobacterium sibiricum].
WP_026831275.1	-	250	bacteria>firmicutes	Exiguobacterium antarcticum	hypothetical protein [Exiguobacterium antarcticum].
WP_026833087.1	-	250	bacteria>firmicutes	Exiguobacterium undae	hypothetical protein [Exiguobacterium undae].
WP_035397458.1	-	249	bacteria>firmicutes	Exiguobacterium sp. OS-77	hypothetical protein [Exiguobacterium sp. OS-77].
WP_050677891.1	-	249	bacteria>firmicutes	Exiguobacterium acetylicum	hypothetical protein [Exiguobacterium acetylicum].
WP_053453318.1	-	249	bacteria>firmicutes	Exiguobacterium sp. BMC-KP	hypothetical protein [Exiguobacterium sp. BMC-KP].
GAE32498.1	JCM9152_4035	244	bacteria>firmicutes	Bacillus hemicellulosilyticus JCM 9152	hypothetical protein JCM9152_4035 [Bacillus hemicellulosilyticus JCM 9152].
GAF17880.1	JCM19046_2413	244	bacteria>firmicutes	Bacillus sp. JCM 19046	LOW QUALITY PROTEIN: hypothetical protein JCM19046_2413 [Bacillus sp. JCM 19046].
GAF21361.1	JCM19047_1039	240	bacteria>firmicutes	Bacillus sp. JCM 19047	LOW QUALITY PROTEIN: hypothetical protein JCM19047_1039 [Bacillus sp. JCM 19047].
WP_011246629.1	-	222	bacteria>firmicutes	Bacillus clausii	hypothetical protein [Bacillus clausii].
WP_059103596.1	-	222	bacteria>firmicutes	Bacillus shacheensis	hypothetical protein [Bacillus shacheensis].
WP_063609388.1	-	222	bacteria>firmicutes	Bacillus clausii	hypothetical protein [Bacillus clausii].
WP_035394054.1	-	219	bacteria>firmicutes	Bacillus sp. JCM 19047	hypothetical protein [Bacillus sp. JCM 19047].
CUB45638.1	BN2127_JRS7_04019	210	bacteria>firmicutes	Bacillus subtilis	hypothetical protein BN2127_JRS7_04019 [Bacillus subtilis].
GAE94986.1	JCM21714_4189	192	bacteria>firmicutes	Gracilibacillus boraciitolerans JCM 21714	hypothetical protein JCM21714_4189 [Gracilibacillus boraciitolerans JCM 21714].
KIL32493.1	B4067_2126	188	bacteria>firmicutes	Bacillus subtilis subsp. subtilis	hypothetical protein B4067_2126 [Bacillus subtilis subsp. subtilis].
WP_060705045.1	-	188	bacteria>firmicutes	Bacillus sp. JCM 19059	hypothetical protein [Bacillus sp. JCM 19059].
WP_054636762.1	-	184	bacteria>firmicutes	Thalassobacillus sp. C254	hypothetical protein [Thalassobacillus sp. C254].
WP_042400352.1	-	154	bacteria>firmicutes	Geomicrobium sp. JCM 19037	hypothetical protein [Geomicrobium sp. JCM 19037].
WP_042428571.1	-	154	bacteria>firmicutes	Geomicrobium sp. JCM 19039	hypothetical protein [Geomicrobium sp. JCM 19039].
WP_054707196.1	-	142	bacteria>firmicutes	Paenibacillus pinihumi	hypothetical protein [Paenibacillus pinihumi].
WP_034745146.1	-	141	bacteria>firmicutes	Bacillus wakoensis	hypothetical protein [Bacillus wakoensis].
BAI85578.1	BSNT_08474	136	bacteria>firmicutes	Bacillus subtilis subsp. natto BEST195	hypothetical protein BSNT_08474 [Bacillus subtilis subsp. natto BEST195].
GAK13446.1	JCM19039_3293	125	bacteria>firmicutes	Geomicrobium sp. JCM 19039	hypothetical protein JCM19039_3293 [Geomicrobium sp. JCM 19039].
GAK03852.1	JCM19037_2208	125	bacteria>firmicutes	Geomicrobium sp. JCM 19037	hypothetical protein JCM19037_2208 [Geomicrobium sp. JCM 19037].
WP_034745148.1	-	120	bacteria>firmicutes	Bacillus wakoensis	hypothetical protein [Bacillus wakoensis].
WP_054707199.1	-	118	bacteria>firmicutes	Paenibacillus pinihumi	hypothetical protein [Paenibacillus pinihumi].
WP_042400350.1	-	106	bacteria>firmicutes	Geomicrobium sp. JCM 19037	hypothetical protein [Geomicrobium sp. JCM 19037].
WP_042428569.1	-	106	bacteria>firmicutes	Geomicrobium sp. JCM 19039	hypothetical protein [Geomicrobium sp. JCM 19039].
WP_054793987.1	-	95	bacteria>firmicutes	Bacillus sp. JCM 19035	hypothetical protein [Bacillus sp. JCM 19035].
WP_054636761.1	-	93	bacteria>firmicutes	Thalassobacillus sp. C254	hypothetical protein [Thalassobacillus sp. C254].
WP_054710823.1	-	72	bacteria>firmicutes	Bacillus sp. JCM 19041	hypothetical protein [Bacillus sp. JCM 19041].

5. family 6

##;baeRF1 family 6 follows the standard core architecture: codon_recognition_domain+baeRF1+Pelota.
##;The Pelota domain appears to contain the 2 beta-strand insert characteristic of the clade.

WP_018615171.1	-	494	bacteria>bacteroidetes	Segetibacter koreensis	hypothetical protein [Segetibacter koreensis].
WP_012794059.1	-	485	bacteria>bacteroidetes	Chitinophaga pinensis	hypothetical protein [Chitinophaga pinensis].
WP_026631811.1	-	482	bacteria>bacteroidetes	Dyadobacter alkalitolerans	hypothetical protein [Dyadobacter alkalitolerans].
WP_041734798.1	-	481	bacteria>bacteroidetes	Dyadobacter fermentans	hypothetical protein [Dyadobacter fermentans].
WP_056097004.1	-	383	bacteria>bacteroidetes	Pedobacter sp. Leaf176	hypothetical protein [Pedobacter sp. Leaf176].
WP_017731890.1	-	423	bacteria>bacteroidetes	Nafulsella turpanensis	hypothetical protein [Nafulsella turpanensis].
WP_011404253.1	-	404	bacteria>bacteroidetes	Salinibacter ruber	hypothetical protein [Salinibacter ruber].
WP_028566862.1	-	401	bacteria>bacteroidetes	Salisaeta longa	hypothetical protein [Salisaeta longa].
WP_053380320.1	-	400	bacteria>nitrospirae	Nitrospira moscoviensis	hypothetical protein [Nitrospira moscoviensis].
WP_029932937.1	-	399	bacteria>actinobacteria	Nocardia otitidiscaviarum	hypothetical protein [Nocardia otitidiscaviarum].
WP_039817779.1	-	399	bacteria>actinobacteria	Nocardia otitidiscaviarum	hypothetical protein [Nocardia otitidiscaviarum].
KPQ36153.1	HLUCCA11_08035	399	bacteria>cyanobacteria	Phormidesmis priestleyi Ana	hypothetical protein HLUCCA11_08035 [Phormidesmis priestleyi Ana].
ABR50339.1	Amet_4261	398	bacteria>firmicutes	Alkaliphilus metalliredigens QYMF	conserved hypothetical protein [Alkaliphilus metalliredigens QYMF].
WP_018397964.1	-	398	bacteria>cyanobacteria	filamentous cyanobacterium ESFC-1	hypothetical protein [filamentous cyanobacterium ESFC-1].
WP_040351793.1	-	398	bacteria>planctomycetes	Blastopirellula marina	hypothetical protein [Blastopirellula marina].
WP_018618915.1	-	397	bacteria>bacteroidetes	Spirosoma luteum	hypothetical protein [Spirosoma luteum].
WP_020607686.1	-	397	bacteria>bacteroidetes	Spirosoma spitsbergense	hypothetical protein [Spirosoma spitsbergense].
WP_027842596.1	-	397	bacteria>cyanobacteria	Mastigocoleus testarum	hypothetical protein [Mastigocoleus testarum].
WP_063718896.1	-	397	bacteria>cyanobacteria	Leptolyngbya valderiana	hypothetical protein [Leptolyngbya valderiana].
WP_025608624.1	-	396	bacteria>bacteroidetes	Pontibacter actiniarum	hypothetical protein [Pontibacter actiniarum].
WP_044238082.1	-	396	bacteria>bacteroidetes	Flexithrix dorotheae	hypothetical protein [Flexithrix dorotheae].
WP_046314162.1	-	396	bacteria>bacteroidetes	Pontibacter korlensis	hypothetical protein [Pontibacter korlensis].
WP_008277932.1	-	395	bacteria>cyanobacteria	Cyanothece sp. CCY0110	hypothetical protein [Cyanothece sp. CCY0110].
AFY86599.1	Chro_1068	394	bacteria>cyanobacteria	Chroococcidiopsis thermalis PCC 7203	hypothetical protein Chro_1068 [Chroococcidiopsis thermalis PCC 7203].
WP_006455091.1	-	394	bacteria>cyanobacteria	Synechococcus sp. PCC 7335	hypothetical protein [Synechococcus sp. PCC 7335].
WP_007222879.1	-	394	bacteria>planctomycetes	Candidatus Jettenia caeni	hypothetical protein [Candidatus Jettenia caeni].
WP_015152011.1	-	394	bacteria>cyanobacteria	Oscillatoria acuminata	hypothetical protein [Oscillatoria acuminata].
WP_029006886.1	-	394	bacteria>proteobacteria>alphaproteobacteria	Azospirillum halopraeferens	hypothetical protein [Azospirillum halopraeferens].
WP_036482027.1	-	394	bacteria>cyanobacteria	Myxosarcina sp. GI1	hypothetical protein [Myxosarcina sp. GI1].
KKS21188.1	UU77_C0007G0033	394	bacteria	candidate division WWE3 bacterium GW2011_GWC1_41_7	hypothetical protein UU77_C0007G0033 [candidate division WWE3 bacterium GW2011_GWC1_41_7].
WP_057939982.1	-	394	bacteria>bacteroidetes	Algoriphagus sp. NH1	hypothetical protein [Algoriphagus sp. NH1].
WP_028979223.1	-	393	bacteria>bacteroidetes	Sporocytophaga myxococcoides	hypothetical protein [Sporocytophaga myxococcoides].
WP_052358990.1	-	393	bacteria>actinobacteria	Corynebacterium variabile	hypothetical protein [Corynebacterium variabile].
WP_017314569.1	-	392	bacteria>cyanobacteria	Mastigocladopsis repens	hypothetical protein [Mastigocladopsis repens].
WP_041040295.1	-	392	bacteria>cyanobacteria	Tolypothrix campylonemoides	hypothetical protein [Tolypothrix campylonemoides].
WP_052299848.1	-	392	bacteria>synergistetes	Thermovirga lienii	hypothetical protein [Thermovirga lienii].
WP_009281406.1	-	391	bacteria>bacteroidetes	Fibrisoma limi	hypothetical protein [Fibrisoma limi].
WP_010997548.1	-	391	bacteria>cyanobacteria	Nostoc sp. PCC 7120	hypothetical protein [Nostoc sp. PCC 7120].
WP_014241166.1	-	391	bacteria>proteobacteria>alphaproteobacteria	Azospirillum brasilense	hypothetical protein [Azospirillum brasilense].
WP_016859881.1	-	391	bacteria>cyanobacteria	Fischerella muscicola	hypothetical protein [Fischerella muscicola].
WP_016873626.1	-	391	bacteria>cyanobacteria	Chlorogloeopsis fritschii	hypothetical protein [Chlorogloeopsis fritschii].
WP_017286018.1	-	391	bacteria>cyanobacteria	Leptolyngbya boryana	hypothetical protein [Leptolyngbya boryana].
WP_020875413.1	-	391	bacteria>proteobacteria>deltaproteobacteria	Desulfococcus multivorans	hypothetical protein [Desulfococcus multivorans].
WP_035075811.1	-	391	bacteria>bacteroidetes	Anditalea andensis	hypothetical protein [Anditalea andensis].
WP_035675274.1	-	391	bacteria>proteobacteria>alphaproteobacteria	Azospirillum brasilense	hypothetical protein [Azospirillum brasilense].
WP_038526610.1	-	391	bacteria>proteobacteria>alphaproteobacteria	Azospirillum brasilense	hypothetical protein [Azospirillum brasilense].
WP_054494451.1	-	391	bacteria>chloroflexi	Ardenticatena maritima	hypothetical protein [Ardenticatena maritima].
BAT55630.1	NOS3756_46230	391	bacteria>cyanobacteria	Nostoc sp. NIES-3756	hypothetical protein NOS3756_46230 [Nostoc sp. NIES-3756].
BAU10141.1	LEP3755_06210	391	bacteria>cyanobacteria	Leptolyngbya sp. NIES-3755	hypothetical protein LEP3755_06210 [Leptolyngbya sp. NIES-3755].
WP_059000728.1	-	391	bacteria>cyanobacteria	Leptolyngbya sp. NIES-2104	hypothetical protein [Leptolyngbya sp. NIES-2104].
BAU63982.1	STA3757_13510	391	bacteria>cyanobacteria	Stanieria sp. NIES-3757	hypothetical protein STA3757_13510 [Stanieria sp. NIES-3757].
WP_062292007.1	-	391	bacteria>cyanobacteria	Nostoc piscinale	hypothetical protein [Nostoc piscinale].
OCQ92971.1	BCD64_07680	391	bacteria>cyanobacteria	Nostoc sp. MBR 210	hypothetical protein BCD64_07680 [Nostoc sp. MBR 210].
WP_012412390.1	-	390	bacteria>cyanobacteria	Nostoc punctiforme	hypothetical protein [Nostoc punctiforme].
WP_013630742.1	-	390	bacteria>planctomycetes	Rubinisphaera brasiliensis	hypothetical protein [Rubinisphaera brasiliensis].
WP_015128234.1	-	390	bacteria>cyanobacteria	Calothrix sp. PCC 7507	hypothetical protein [Calothrix sp. PCC 7507].
WP_015139584.1	-	390	bacteria>cyanobacteria	Nostoc sp. PCC 7524	hypothetical protein [Nostoc sp. PCC 7524].
WP_018476333.1	-	390	bacteria>bacteroidetes	Pontibacter roseus	hypothetical protein [Pontibacter roseus].
WP_019668128.1	-	390	bacteria>bacteroidetes	Eudoraea adriatica	hypothetical protein [Eudoraea adriatica].
WP_026093019.1	-	390	bacteria>cyanobacteria	Calothrix sp. PCC 7103	hypothetical protein [Calothrix sp. PCC 7103].
WP_029033226.1	-	390	bacteria>bacteroidetes	Salinimicrobium terrae	hypothetical protein [Salinimicrobium terrae].
WP_029036179.1	-	390	bacteria>bacteroidetes	Salinimicrobium xinjiangense	hypothetical protein [Salinimicrobium xinjiangense].
WP_038091877.1	-	390	bacteria>cyanobacteria	Tolypothrix bouteillei	hypothetical protein [Tolypothrix bouteillei].
WP_039717500.1	-	390	bacteria>cyanobacteria	Scytonema millei	hypothetical protein [Scytonema millei].
WP_048869462.1	-	390	bacteria>cyanobacteria	Scytonema tolypothrichoides	hypothetical protein [Scytonema tolypothrichoides].
KYC39497.1	WA1_32815	390	bacteria>cyanobacteria	Scytonema hofmannii PCC 7110	hypothetical protein WA1_32815 [Scytonema hofmannii PCC 7110].
WP_009034349.1	-	389	bacteria>bacteroidetes	Indibacter alkaliphilus	hypothetical protein [Indibacter alkaliphilus].
WP_009545509.1	-	389	bacteria>cyanobacteria	Cyanothece	MULTISPECIES: hypothetical protein [Cyanothece].
WP_009578213.1	-	389	bacteria>bacteroidetes	Fulvivirga imtechensis	hypothetical protein [Fulvivirga imtechensis].
WP_013190918.1	-	389	bacteria>cyanobacteria	Trichormus azollae	hypothetical protein [Trichormus azollae].
WP_016869705.1	-	389	bacteria>cyanobacteria	Fischerella muscicola	hypothetical protein [Fischerella muscicola].
WP_017309585.1	-	389	bacteria>cyanobacteria	Fischerella sp. PCC 9339	hypothetical protein [Fischerella sp. PCC 9339].
WP_017654956.1	-	389	bacteria>cyanobacteria	Microchaete sp. PCC 7126	hypothetical protein [Microchaete sp. PCC 7126].
WP_017720482.1	-	389	bacteria>cyanobacteria	Oscillatoria sp. PCC 10802	hypothetical protein [Oscillatoria sp. PCC 10802].
WP_026723257.1	-	389	bacteria>cyanobacteria	Fischerella sp. PCC 9431	hypothetical protein [Fischerella sp. PCC 9431].
WP_026734003.1	-	389	bacteria>cyanobacteria	Fischerella sp. PCC 9605	hypothetical protein [Fischerella sp. PCC 9605].
WP_036009945.1	-	389	bacteria>cyanobacteria	Leptolyngbya sp. JSC-1	hypothetical protein [Leptolyngbya sp. JSC-1].
WP_039748452.1	-	389	bacteria>cyanobacteria	Hassallia byssoidea	hypothetical protein [Hassallia byssoidea].
WP_044446390.1	-	389	bacteria>cyanobacteria	Mastigocladus laminosus	hypothetical protein [Mastigocladus laminosus].
WP_045055836.1	-	389	bacteria>cyanobacteria	Aliterella atlantica	hypothetical protein [Aliterella atlantica].
WP_053458714.1	-	389	bacteria>cyanobacteria	Hapalosiphon sp. MRB220	hypothetical protein [Hapalosiphon sp. MRB220].
KUO67970.1	APF83_12890	389	bacteria>bacteroidetes	Lutibacter sp. BRH_c52	hypothetical protein APF83_12890 [Lutibacter sp. BRH_c52].
ABA23692.1	Ava_4088	388	bacteria>cyanobacteria	Anabaena variabilis ATCC 29413	conserved hypothetical protein [Anabaena variabilis ATCC 29413].
WP_006964593.1	-	388	bacteria>proteobacteria>deltaproteobacteria	Desulfotignum phosphitoxidans	hypothetical protein [Desulfotignum phosphitoxidans].
WP_007916216.1	-	388	bacteria>chloroflexi	Ktedonobacter racemifer	hypothetical protein [Ktedonobacter racemifer].
WP_009756818.1	-	388	bacteria>cyanobacteria	Fischerella	MULTISPECIES: hypothetical protein [Fischerella].
WP_013325235.1	-	388	bacteria>cyanobacteria	Cyanothece sp. PCC 7822	hypothetical protein [Cyanothece sp. PCC 7822].
WP_015083156.1	-	388	bacteria>cyanobacteria	Anabaena sp. 90	hypothetical protein [Anabaena sp. 90].
WP_015189357.1	-	388	bacteria>cyanobacteria	Gloeocapsa sp. PCC 7428	hypothetical protein [Gloeocapsa sp. PCC 7428].
WP_015209154.1	-	388	bacteria>cyanobacteria	Cylindrospermum stagnale	hypothetical protein [Cylindrospermum stagnale].
WP_015954243.1	-	388	bacteria>cyanobacteria	Cyanothece sp. PCC 7424	hypothetical protein [Cyanothece sp. PCC 7424].
WP_016951308.1	-	388	bacteria>cyanobacteria	Anabaena sp. PCC 7108	hypothetical protein [Anabaena sp. PCC 7108].
WP_024335127.1	-	388	bacteria>proteobacteria>deltaproteobacteria	Desulfotignum balticum	hypothetical protein [Desulfotignum balticum].
WP_027403556.1	-	388	bacteria>cyanobacteria	Aphanizomenon flos-aquae	hypothetical protein [Aphanizomenon flos-aquae].
WP_028083724.1	-	388	bacteria>cyanobacteria	Dolichospermum circinale	hypothetical protein [Dolichospermum circinale].
WP_028090208.1	-	388	bacteria>cyanobacteria	Dolichospermum circinale	hypothetical protein [Dolichospermum circinale].
WP_039201252.1	-	388	bacteria>cyanobacteria	Aphanizomenon flos-aquae	hypothetical protein [Aphanizomenon flos-aquae].
WP_041462843.1	-	388	bacteria>cyanobacteria	Chroococcidiopsis thermalis	hypothetical protein [Chroococcidiopsis thermalis].
WP_053456388.1	-	388	bacteria>cyanobacteria	Hapalosiphon sp. MRB220	hypothetical protein [Hapalosiphon sp. MRB220].
WP_053537620.1	-	388	bacteria>cyanobacteria	Anabaena sp. wa102	hypothetical protein [Anabaena sp. wa102].
KPL21813.1	AMJ93_08745	388	bacteria>chloroflexi	Anaerolineae bacterium SM23_84	hypothetical protein AMJ93_08745 [Anaerolineae bacterium SM23_84].
WP_062248008.1	-	388	bacteria>cyanobacteria	Fischerella sp. NIES-3754	hypothetical protein [Fischerella sp. NIES-3754].
OBQ08329.1	AN490_10065	388	bacteria>cyanobacteria	Anabaena sp. 39864	hypothetical protein AN490_10065 [Anabaena sp. 39864].
OBQ13469.1	AN482_04955	388	bacteria>cyanobacteria	Anabaena sp. 39865	hypothetical protein AN482_04955 [Anabaena sp. 39865].
OBQ18138.1	AN488_17520	388	bacteria>cyanobacteria	Anabaena sp. WA113	hypothetical protein AN488_17520 [Anabaena sp. WA113].
OBQ20021.1	AN481_17320	388	bacteria>cyanobacteria	Aphanizomenon flos-aquae MDT13	hypothetical protein AN481_17320 [Aphanizomenon flos-aquae MDT13].
OBQ21244.1	AN486_04970	388	bacteria>cyanobacteria	Anabaena sp. WA93	hypothetical protein AN486_04970 [Anabaena sp. WA93].
OBQ27887.1	AN483_18435	388	bacteria>cyanobacteria	Aphanizomenon flos-aquae MDT14a	hypothetical protein AN483_18435 [Aphanizomenon flos-aquae MDT14a].
OBQ39410.1	AN487_04635	388	bacteria>cyanobacteria	Anabaena sp. CRKS33	hypothetical protein AN487_04635 [Anabaena sp. CRKS33].
EAQ79924.1	DSM3645_22329	387	bacteria>planctomycetes	Blastopirellula marina DSM 3645	hypothetical protein DSM3645_22329 [Blastopirellula marina DSM 3645].
WP_006194682.1	-	387	bacteria>cyanobacteria	Nodularia spumigena	hypothetical protein [Nodularia spumigena].
WP_006276443.1	-	387	bacteria>cyanobacteria	Cylindrospermopsis raciborskii	hypothetical protein [Cylindrospermopsis raciborskii].
WP_006634544.1	-	387	bacteria>cyanobacteria	Microcoleus vaginatus	hypothetical protein [Microcoleus vaginatus].
WP_007357230.1	-	387	bacteria>cyanobacteria	[Oscillatoria] sp. PCC 6506	hypothetical protein [[Oscillatoria] sp. PCC 6506].
WP_008629327.1	-	387	bacteria>bacteroidetes	Mariniradius saccharolyticus	hypothetical protein [Mariniradius saccharolyticus].
WP_009343695.1	-	387	bacteria>cyanobacteria	Raphidiopsis brookii	hypothetical protein [Raphidiopsis brookii].
WP_012629204.1	-	387	bacteria>cyanobacteria	Cyanothece sp. PCC 7425	hypothetical protein [Cyanothece sp. PCC 7425].
WP_015174243.1	-	387	bacteria>cyanobacteria	Oscillatoria nigro-viridis	hypothetical protein [Oscillatoria nigro-viridis].
WP_015202256.1	-	387	bacteria>cyanobacteria	Crinalium epipsammum	hypothetical protein [Crinalium epipsammum].
WP_015214975.1	-	387	bacteria>cyanobacteria	Anabaena cylindrica	hypothetical protein [Anabaena cylindrica].
WP_039728624.1	-	387	bacteria	Cyanobacteria	MULTISPECIES: hypothetical protein [Cyanobacteria].
WP_057178873.1	-	387	bacteria>cyanobacteria	Cylindrospermopsis sp. CR12	hypothetical protein [Cylindrospermopsis sp. CR12].
CEJ44690.1	apha_01510	387	bacteria>cyanobacteria	Chrysosporum ovalisporum	Uncharacterized protein apha_01510 [Chrysosporum ovalisporum].
WP_057954256.1	-	387	bacteria	bacterium L21-Spi-D4	hypothetical protein [bacterium L21-Spi-D4].
WP_061546801.1	-	387	bacteria>cyanobacteria	Cylindrospermopsis raciborskii	hypothetical protein [Cylindrospermopsis raciborskii].
AMV34268.1	VN12_19235	387	bacteria>planctomycetes	Pirellula sp. SH-Sr6A	hypothetical protein VN12_19235 [Pirellula sp. SH-Sr6A].
WP_063872071.1	-	387	bacteria>cyanobacteria	Nodularia spumigena	hypothetical protein [Nodularia spumigena].
WP_025229594.1	-	386	bacteria>armatimonadetes	Fimbriimonas ginsengisoli	hypothetical protein [Fimbriimonas ginsengisoli].
GAL85276.1	MYP_2505	386	bacteria>bacteroidetes	Sporocytophaga myxococcoides	hypothetical protein MYP_2505 [Sporocytophaga myxococcoides].
WP_041387564.1	-	386	bacteria>bacteroidetes	Sphingobacterium sp. 21	hypothetical protein [Sphingobacterium sp. 21].
WP_057483249.1	-	386	bacteria>bacteroidetes	Salegentibacter mishustinae	hypothetical protein [Salegentibacter mishustinae].
WP_003437611.1	-	385	bacteria>bacteroidetes	Psychroflexus gondwanensis	hypothetical protein [Psychroflexus gondwanensis].
WP_005986527.1	-	385	bacteria>proteobacteria>deltaproteobacteria	Desulfovibrio africanus	hypothetical protein [Desulfovibrio africanus].
WP_008518811.1	-	385	bacteria>firmicutes	Dethiobacter alkaliphilus	hypothetical protein [Dethiobacter alkaliphilus].
WP_009195365.1	-	385	bacteria>bacteroidetes	Cesiribacter andamanensis	hypothetical protein [Cesiribacter andamanensis].
WP_014259120.1	-	385	bacteria>proteobacteria>deltaproteobacteria	Desulfovibrio africanus	hypothetical protein [Desulfovibrio africanus].
WP_027368766.1	-	385	bacteria>proteobacteria>deltaproteobacteria	Desulfovibrio africanus	hypothetical protein [Desulfovibrio africanus].
WP_037321481.1	-	385	bacteria>bacteroidetes	Salegentibacter sp. Hel_I_6	hypothetical protein [Salegentibacter sp. Hel_I_6].
WP_052642235.1	-	385	bacteria>fibrobacteres	Chitinispirillum alkaliphilum	hypothetical protein [Chitinispirillum alkaliphilum].
WP_061984831.1	-	385	bacteria>bacteroidetes	Flammeovirgaceae bacterium 311	hypothetical protein [Flammeovirgaceae bacterium 311].
WP_007427893.1	-	384	bacteria>proteobacteria>alphaproteobacteria	Oceaniovalibus guishaninsula	hypothetical protein [Oceaniovalibus guishaninsula].
WP_011709631.1	-	384	bacteria>bacteroidetes	Gramella forsetii	hypothetical protein [Gramella forsetii].
WP_014810483.1	-	384	bacteria>proteobacteria>deltaproteobacteria	Desulfomonile tiedjei	hypothetical protein [Desulfomonile tiedjei].
WP_015154750.1	-	384	bacteria>cyanobacteria	Chroococcidiopsis thermalis	hypothetical protein [Chroococcidiopsis thermalis].
WP_015753812.1	-	384	bacteria>bacteroidetes	Robiginitalea biformata	hypothetical protein [Robiginitalea biformata].
WP_026086769.1	-	384	bacteria>cyanobacteria	Fischerella muscicola	hypothetical protein [Fischerella muscicola].
WP_026811670.1	-	384	bacteria>bacteroidetes	Arenibacter latericius	hypothetical protein [Arenibacter latericius].
WP_026814886.1	-	384	bacteria>bacteroidetes	Arenibacter certesii	hypothetical protein [Arenibacter certesii].
WP_026916286.1	-	384	bacteria>bacteroidetes	Gramella portivictoriae	hypothetical protein [Gramella portivictoriae].
WP_026934988.1	-	384	bacteria>bacteroidetes	Gramella echinicola	hypothetical protein [Gramella echinicola].
WP_028321640.1	-	384	bacteria>proteobacteria>deltaproteobacteria	Desulfatiglans anilini	hypothetical protein [Desulfatiglans anilini].
WP_031441941.1	-	384	bacteria>bacteroidetes	Arenibacter algicola	hypothetical protein [Arenibacter algicola].
WP_035991808.1	-	384	bacteria>cyanobacteria	Leptolyngbya sp. KIOST-1	hypothetical protein [Leptolyngbya sp. KIOST-1].
WP_045801009.1	-	384	bacteria>bacteroidetes	Muricauda lutaonensis	hypothetical protein [Muricauda lutaonensis].
KPK82480.1	AMS25_02105	384	bacteria>gemmatimonadetes	Gemmatimonas sp. SM23_52	hypothetical protein AMS25_02105 [Gemmatimonas sp. SM23_52].
KXK16778.1	UZ18_ATM001001255	384	bacteria>armatimonadetes	Armatimonadetes bacterium OLB18	hypothetical protein UZ18_ATM001001255 [Armatimonadetes bacterium OLB18].
WP_009456329.1	-	383	bacteria>cyanobacteria	Fischerella sp. JSC-11	hypothetical protein [Fischerella sp. JSC-11].
WP_015190266.1	-	383	bacteria>cyanobacteria	Gloeocapsa sp. PCC 7428	hypothetical protein [Gloeocapsa sp. PCC 7428].
WP_016866434.1	-	383	bacteria>cyanobacteria	Fischerella muscicola	hypothetical protein [Fischerella muscicola].
WP_016872047.1	-	383	bacteria>cyanobacteria	Fischerella thermalis	hypothetical protein [Fischerella thermalis].
WP_017314110.1	-	383	bacteria>cyanobacteria	Mastigocladopsis repens	hypothetical protein [Mastigocladopsis repens].
WP_020539185.1	-	383	bacteria>bacteroidetes	Lewinella cohaerens	hypothetical protein [Lewinella cohaerens].
AHY48341.1	RradSPS_3058	383	bacteria>actinobacteria	Rubrobacter radiotolerans	Hypothetical Protein RradSPS_3058 (plasmid) [Rubrobacter radiotolerans].
WP_024822852.1	-	383	bacteria>synergistetes	Aminobacterium mobile	hypothetical protein [Aminobacterium mobile].
WP_026093518.1	-	383	bacteria>cyanobacteria	Calothrix sp. PCC 7103	hypothetical protein [Calothrix sp. PCC 7103].
WP_026731103.1	-	383	bacteria>cyanobacteria	Fischerella sp. PCC 9605	hypothetical protein [Fischerella sp. PCC 9605].
WP_062246043.1	-	383	bacteria>cyanobacteria	Fischerella sp. NIES-3754	hypothetical protein [Fischerella sp. NIES-3754].
WP_013047602.1	-	382	bacteria>synergistetes	Aminobacterium colombiense	hypothetical protein [Aminobacterium colombiense].
WP_013176891.1	-	382	bacteria>deinococcus-thermus>deinococci	Truepera radiovictrix	hypothetical protein [Truepera radiovictrix].
WP_016877930.1	-	382	bacteria>cyanobacteria	Chlorogloeopsis fritschii	hypothetical protein [Chlorogloeopsis fritschii].
WP_026551707.1	-	382	bacteria>actinobacteria	Arthrobacter sp. H20	hypothetical protein [Arthrobacter sp. H20].
WP_044825621.1	-	382	bacteria>firmicutes	Clostridium aceticum	hypothetical protein [Clostridium aceticum].
KUK49266.1	XD74_0154	382	bacteria>actinobacteria	Actinobacteria bacterium 66_15	Uncharacterized protein XD74_0154 [Actinobacteria bacterium 66_15].
WP_006989325.1	-	381	bacteria>bacteroidetes	Gillisia limnaea	hypothetical protein [Gillisia limnaea].
WP_013360435.1	-	381	bacteria>firmicutes	[Clostridium] sticklandii	hypothetical protein [[Clostridium] sticklandii].
WP_020403181.1	-	381	bacteria>bacteroidetes	Gracilimonas tropica	hypothetical protein [Gracilimonas tropica].
WP_044215665.1	-	381	bacteria>bacteroidetes	Phaeodactylibacter xiamenensis	hypothetical protein [Phaeodactylibacter xiamenensis].
WP_052287933.1	-	381	bacteria>cyanobacteria	Lyngbya confervoides	hypothetical protein [Lyngbya confervoides].
WP_011417633.1	-	380	bacteria>proteobacteria>deltaproteobacteria	Syntrophus aciditrophicus	hypothetical protein [Syntrophus aciditrophicus].
WP_014553651.1	-	380	bacteria>firmicutes	Halanaerobium praevalens	hypothetical protein [Halanaerobium praevalens].
WP_055683508.1	-	380	bacteria>proteobacteria>alphaproteobacteria	Jannaschia rubra	hypothetical protein [Jannaschia rubra].
KUK74223.1	XD91_0081	380	bacteria>firmicutes	Clostridiales bacterium 38_11	Uncharacterized protein XD91_0081 [Clostridiales bacterium 38_11].
WP_007628342.1	-	379	bacteria>actinobacteria	Dietzia cinnamea	hypothetical protein [Dietzia cinnamea].
WP_013406287.1	-	379	bacteria>firmicutes	Halanaerobium hydrogeniformans	hypothetical protein [Halanaerobium hydrogeniformans].
WP_036735563.1	-	379	bacteria>firmicutes	Peptococcaceae bacterium SCADC1_2_3	hypothetical protein [Peptococcaceae bacterium SCADC1_2_3].
WP_036748472.1	-	379	bacteria>firmicutes	Peptococcaceae bacterium SCADC1_2_3	hypothetical protein [Peptococcaceae bacterium SCADC1_2_3].
WP_053182439.1	-	379	bacteria>bacteroidetes	Sunxiuqinia dokdonensis	hypothetical protein [Sunxiuqinia dokdonensis].
WP_061229766.1	-	379	bacteria>actinobacteria	Dietzia cinnamea	hypothetical protein [Dietzia cinnamea].
WP_061914100.1	-	379	bacteria>actinobacteria	Dietzia	MULTISPECIES: hypothetical protein [Dietzia].
WP_063972196.1	-	379	bacteria>actinobacteria	Dietzia cinnamea	hypothetical protein [Dietzia cinnamea].
OAV77893.1	AYO52_14910	379	bacteria>actinobacteria	Dietzia sp. 111N12-1	hypothetical protein AYO52_14910 [Dietzia sp. 111N12-1].
OBQ34026.1	AN485_17115	379	bacteria>cyanobacteria	Anabaena sp. MDT14b	hypothetical protein AN485_17115, partial [Anabaena sp. MDT14b].
WP_005487297.1	-	378	bacteria>firmicutes	Halanaerobium saccharolyticum	hypothetical protein [Halanaerobium saccharolyticum].
OAN64116.1	A8B79_14065	378	bacteria	Balneola sp. EhC07	hypothetical protein A8B79_14065 [Balneola sp. EhC07].
WP_010540054.1	-	377	bacteria>actinobacteria	Dietzia alimentaria	hypothetical protein [Dietzia alimentaria].
WP_046736482.1	-	377	bacteria>chloroflexi	Dehalogenimonas sp. WBC-2	hypothetical protein [Dehalogenimonas sp. WBC-2].
WP_051510585.1	-	377	bacteria>actinobacteria	Intrasporangium oryzae	hypothetical protein [Intrasporangium oryzae].
WP_006591404.1	-	376	bacteria>actinobacteria	Kineosphaera limosa	hypothetical protein [Kineosphaera limosa].
WP_022921474.1	-	376	bacteria>actinobacteria	Ornithinimicrobium pekingense	hypothetical protein [Ornithinimicrobium pekingense].
WP_013217731.1	-	375	bacteria>chloroflexi	Dehalogenimonas lykanthroporepellens	hypothetical protein [Dehalogenimonas lykanthroporepellens].
SCE67135.1	GA0070607_0201	375	bacteria>actinobacteria	Micromonospora coriariae	hypothetical protein GA0070607_0201 [Micromonospora coriariae].
KGE89866.1	IX84_00730	372	bacteria>bacteroidetes	Phaeodactylibacter xiamenensis	hypothetical protein IX84_00730 [Phaeodactylibacter xiamenensis].
KIF43656.1	QQ91_04775	372	bacteria>cyanobacteria	Lyngbya confervoides BDU141951	hypothetical protein QQ91_04775 [Lyngbya confervoides BDU141951].
KUK84423.1	XE01_1415	372	bacteria>synergistetes	Synergistales bacterium 58_81	Uncharacterized protein XE01_1415 [Synergistales bacterium 58_81].
WP_017838371.1	-	368	bacteria>actinobacteria	Dietzia sp. UCD-THP	hypothetical protein [Dietzia sp. UCD-THP].
WP_019158080.1	-	368	bacteria>actinobacteria	Brevibacterium senegalense	hypothetical protein [Brevibacterium senegalense].
WP_019174403.1	-	368	bacteria>actinobacteria	Brevibacterium ravenspurgense	hypothetical protein [Brevibacterium ravenspurgense].
WP_038465285.1	-	368	bacteria>actinobacteria	Arthrobacter sp. PAMC 25486	hypothetical protein [Arthrobacter sp. PAMC 25486].
WP_061941089.1	-	368	bacteria>actinobacteria	Brevibacterium ravenspurgense	hypothetical protein [Brevibacterium ravenspurgense].
WP_062022403.1	-	368	bacteria>actinobacteria	Brevibacterium ravenspurgense	hypothetical protein [Brevibacterium ravenspurgense].
WP_005936247.1	-	367	bacteria>actinobacteria	Gordonia hirsuta	hypothetical protein [Gordonia hirsuta].
WP_017792660.1	-	367	bacteria>actinobacteria	Leucobacter salsicius	hypothetical protein [Leucobacter salsicius].
WP_027286502.1	-	367	bacteria>actinobacteria	Ruania albidiflava	hypothetical protein [Ruania albidiflava].
BAU32800.1	MalAC0309_1955	367	bacteria>actinobacteria	Microcella alkaliphila	uncharacterized protein MalAC0309_1955 [Microcella alkaliphila].
WP_027019557.1	-	366	bacteria>actinobacteria	Corynebacterium sputi	hypothetical protein [Corynebacterium sputi].
KXS47199.1	AWL62_2798	366	bacteria>firmicutes	Halanaerobium sp. T82-1	hypothetical protein AWL62_2798, partial [Halanaerobium sp. T82-1].
WP_052889399.1	-	365	bacteria>chloroflexi	Thermogemmatispora carboxidivorans	hypothetical protein [Thermogemmatispora carboxidivorans].
WP_010119552.1	-	364	bacteria>actinobacteria	Corynebacterium nuruki	hypothetical protein [Corynebacterium nuruki].
WP_022869245.1	-	364	bacteria>actinobacteria	Yaniella halotolerans	hypothetical protein [Yaniella halotolerans].
WP_043056826.1	-	364	bacteria>proteobacteria>alphaproteobacteria	Mesorhizobium sp. UASWS1009	hypothetical protein [Mesorhizobium sp. UASWS1009].
WP_020661536.1	-	363	bacteria>actinobacteria	Amycolatopsis benzoatilytica	hypothetical protein [Amycolatopsis benzoatilytica].
WP_006770109.1	-	362	bacteria>actinobacteria	Corynebacterium efficiens	hypothetical protein [Corynebacterium efficiens].
WP_017301345.1	-	362	bacteria>cyanobacteria	Nodosilinea nodulosa	hypothetical protein [Nodosilinea nodulosa].
WP_035121932.1	-	362	bacteria>actinobacteria	Corynebacterium freneyi	hypothetical protein [Corynebacterium freneyi].
WP_065421555.1	-	362	bacteria>actinobacteria	Corynebacterium halotolerans	hypothetical protein [Corynebacterium halotolerans].
WP_005881808.1	-	361	bacteria>actinobacteria	Brevibacterium mcbrellneri	hypothetical protein [Brevibacterium mcbrellneri].
WP_009154245.1	-	361	bacteria>actinobacteria	Saccharomonospora marina	hypothetical protein [Saccharomonospora marina].
WP_018298005.1	-	361	bacteria>actinobacteria	Corynebacterium lubricantis	hypothetical protein [Corynebacterium lubricantis].
WP_040084517.1	-	361	bacteria>actinobacteria	Corynebacterium humireducens	hypothetical protein [Corynebacterium humireducens].
WP_042620482.1	-	361	bacteria>actinobacteria	Corynebacterium marinum	hypothetical protein [Corynebacterium marinum].
WP_007659182.1	-	355	bacteria>bacteroidetes	Pontibacter sp. BAB1700	hypothetical protein [Pontibacter sp. BAB1700].
WP_024283341.1	-	323	bacteria>bacteroidetes	Algoriphagus marincola	hypothetical protein [Algoriphagus marincola].
KPJ90026.1	AMS18_11765	316	bacteria>gemmatimonadetes	Gemmatimonas sp. SG8_17	hypothetical protein AMS18_11765, partial [Gemmatimonas sp. SG8_17].
WP_017662838.1	-	282	bacteria>cyanobacteria	Geitlerinema sp. PCC 7105	hypothetical protein [Geitlerinema sp. PCC 7105].
EDX75755.1	MC7420_6410	275	bacteria>cyanobacteria	Coleofasciculus chthonoplastes PCC 7420	hypothetical protein MC7420_6410 [Coleofasciculus chthonoplastes PCC 7420].
EYT47540.1	H483_0118850	236	bacteria>actinobacteria	Dietzia sp. UCD-THP	hypothetical protein H483_0118850, partial [Dietzia sp. UCD-THP].
WP_062053734.1	-	389	bacteria>bacteroidetes	Sediminicola sp. YIK13	hypothetical protein [Sediminicola sp. YIK13].
WP_031526492.1	-	480	bacteria>bacteroidetes	Dyadobacter crusticola	hypothetical protein [Dyadobacter crusticola].
WP_056284644.1	-	480	bacteria>bacteroidetes	Dyadobacter sp. Leaf189	hypothetical protein [Dyadobacter sp. Leaf189].
WP_049768802.1	-	410	bacteria>chloroflexi	Roseiflexus castenholzii	hypothetical protein [Roseiflexus castenholzii].
WP_011958799.1	-	409	bacteria>chloroflexi	Roseiflexus sp. RS-1	hypothetical protein [Roseiflexus sp. RS-1].
OCL34576.1	BCR15_02445	382	bacteria>actinobacteria	Tessaracoccus lapidicaptus	hypothetical protein BCR15_02445 [Tessaracoccus lapidicaptus].
ADB37891.1	Slin_1846	390	bacteria>bacteroidetes	Spirosoma linguale DSM 74	putative cytoplasmic protein [Spirosoma linguale DSM 74].
WP_009186967.1	-	389	bacteria>bacteroidetes	Cecembia lonarensis	hypothetical protein [Cecembia lonarensis].
KJS28664.1	VR64_23365	387	bacteria>proteobacteria>deltaproteobacteria	Desulfatitalea sp. BRH_c12	hypothetical protein VR64_23365 [Desulfatitalea sp. BRH_c12].
WP_009784563.1	-	384	bacteria>cyanobacteria	Lyngbya sp. PCC 8106	hypothetical protein [Lyngbya sp. PCC 8106].
WP_023063904.1	-	384	bacteria>cyanobacteria	Lyngbya aestuarii	hypothetical protein [Lyngbya aestuarii].
WP_046276573.1	-	384	bacteria>cyanobacteria	Limnoraphis robusta	hypothetical protein [Limnoraphis robusta].
WP_011910713.1	-	383	bacteria>proteobacteria>alphaproteobacteria	Rhodobacter sphaeroides	hypothetical protein [Rhodobacter sphaeroides].
ANH37085.1	I601_0633	382	bacteria>actinobacteria	Nocardioides dokdonensis FR1436	hypothetical protein I601_0633 [Nocardioides dokdonensis FR1436].

6. family 7

##;baeRF1 family 7 follows the standard core architecture: codon_recognition_domain+baeRF1+Pelota.
##;The Pelota domain appears to contain the 2 beta-strand insert characteristic of the clade.

WP_018027145.1	-	405	bacteria>proteobacteria>betaproteobacteria	Oligella urethralis	hypothetical protein [Oligella urethralis].
WP_018438869.1	-	395	bacteria>proteobacteria>betaproteobacteria	Burkholderia sp. JPY347	hypothetical protein [Burkholderia sp. JPY347].
WP_051077739.1	-	395	bacteria>proteobacteria>betaproteobacteria	Oligella ureolytica	hypothetical protein [Oligella ureolytica].
WP_003179684.1	-	391	bacteria>proteobacteria>gammaproteobacteria	Pseudomonas fluorescens	hypothetical protein [Pseudomonas fluorescens].
WP_004882263.1	-	391	bacteria>proteobacteria>gammaproteobacteria	Pseudomonas syringae group	MULTISPECIES: hypothetical protein [Pseudomonas syringae group].
WP_029242660.1	-	391	bacteria>proteobacteria>gammaproteobacteria	Pseudomonas viridiflava	hypothetical protein [Pseudomonas viridiflava].
WP_030142061.1	-	391	bacteria>proteobacteria>gammaproteobacteria	Pseudomonas fluorescens	hypothetical protein [Pseudomonas fluorescens].
WP_037017868.1	-	391	bacteria>proteobacteria>gammaproteobacteria	Pseudomonas lutea	hypothetical protein [Pseudomonas lutea].
WP_039593967.1	-	391	bacteria>proteobacteria>gammaproteobacteria	Pseudomonas frederiksbergensis	hypothetical protein [Pseudomonas frederiksbergensis].
WP_043303556.1	-	391	bacteria>proteobacteria>gammaproteobacteria	Pseudomonas viridiflava	hypothetical protein [Pseudomonas viridiflava].
WP_045155545.1	-	391	bacteria>proteobacteria>gammaproteobacteria	Pseudomonas fluorescens	hypothetical protein [Pseudomonas fluorescens].
WP_057408294.1	-	391	bacteria>proteobacteria>gammaproteobacteria	Pseudomonas syringae group genomosp. 3	hypothetical protein [Pseudomonas syringae group genomosp. 3].
KTC09163.1	AO390_16490	391	bacteria>proteobacteria>gammaproteobacteria	Pseudomonas marginalis ICMP 11289	hypothetical protein AO390_16490 [Pseudomonas marginalis ICMP 11289].
WP_027589198.1	-	390	bacteria>proteobacteria>gammaproteobacteria	Pseudomonas	MULTISPECIES: hypothetical protein [Pseudomonas].
WP_003285788.1	-	388	bacteria>proteobacteria>gammaproteobacteria	Pseudomonas stutzeri	hypothetical protein [Pseudomonas stutzeri].
WP_003295035.1	-	388	bacteria>proteobacteria>gammaproteobacteria	Pseudomonas stutzeri	hypothetical protein [Pseudomonas stutzeri].
WP_003299343.1	-	388	bacteria>proteobacteria>gammaproteobacteria	Pseudomonas stutzeri	hypothetical protein [Pseudomonas stutzeri].
WP_011912723.1	-	388	bacteria>proteobacteria>gammaproteobacteria	Pseudomonas stutzeri	hypothetical protein [Pseudomonas stutzeri].
WP_013982345.1	-	388	bacteria>proteobacteria>gammaproteobacteria	Pseudomonas stutzeri	hypothetical protein [Pseudomonas stutzeri].
WP_014596303.1	-	388	bacteria>proteobacteria>gammaproteobacteria	Pseudomonas stutzeri	hypothetical protein [Pseudomonas stutzeri].
WP_014819922.1	-	388	bacteria>proteobacteria>gammaproteobacteria	Pseudomonas stutzeri group	MULTISPECIES: hypothetical protein [Pseudomonas stutzeri group].
WP_015277577.1	-	388	bacteria>proteobacteria>gammaproteobacteria	Pseudomonas stutzeri	hypothetical protein [Pseudomonas stutzeri].
WP_017245786.1	-	388	bacteria>proteobacteria>gammaproteobacteria	Pseudomonas stutzeri	hypothetical protein [Pseudomonas stutzeri].
WP_019342403.1	-	388	bacteria>proteobacteria>gammaproteobacteria	Pseudomonas stutzeri	hypothetical protein [Pseudomonas stutzeri].
WP_021206568.1	-	388	bacteria>proteobacteria>gammaproteobacteria	Pseudomonas stutzeri	hypothetical protein [Pseudomonas stutzeri].
WP_025240940.1	-	388	bacteria>proteobacteria>gammaproteobacteria	Pseudomonas stutzeri	hypothetical protein [Pseudomonas stutzeri].
WP_037042041.1	-	388	bacteria>proteobacteria>gammaproteobacteria	Pseudomonas sp. BAY1663	hypothetical protein [Pseudomonas sp. BAY1663].
WP_038661604.1	-	388	bacteria>proteobacteria>gammaproteobacteria	Pseudomonas stutzeri	hypothetical protein [Pseudomonas stutzeri].
WP_042925644.1	-	388	bacteria>proteobacteria>gammaproteobacteria	Pseudomonas chloritidismutans	hypothetical protein [Pseudomonas chloritidismutans].
WP_044314624.1	-	388	bacteria>proteobacteria>gammaproteobacteria	Pseudomonas stutzeri	hypothetical protein [Pseudomonas stutzeri].
WP_045158460.1	-	388	bacteria>proteobacteria>gammaproteobacteria	Pseudomonas stutzeri	hypothetical protein [Pseudomonas stutzeri].
WP_045161303.1	-	388	bacteria>proteobacteria>gammaproteobacteria	Pseudomonas stutzeri	hypothetical protein [Pseudomonas stutzeri].
WP_045428981.1	-	388	bacteria>proteobacteria>gammaproteobacteria	Pseudomonas	MULTISPECIES: hypothetical protein [Pseudomonas].
WP_046162022.1	-	388	bacteria>proteobacteria>gammaproteobacteria	Pseudomonas sp. 10B238	hypothetical protein [Pseudomonas sp. 10B238].
WP_049338994.1	-	388	bacteria>proteobacteria>gammaproteobacteria	Pseudomonas stutzeri	hypothetical protein [Pseudomonas stutzeri].
WP_058065700.1	-	388	bacteria>proteobacteria>gammaproteobacteria	Pseudomonas sp. TTU2014-105ASC	hypothetical protein [Pseudomonas sp. TTU2014-105ASC].
WP_063543817.1	-	388	bacteria>proteobacteria>gammaproteobacteria	Pseudomonas stutzeri	hypothetical protein [Pseudomonas stutzeri].
WP_063625703.1	-	388	bacteria>proteobacteria>gammaproteobacteria	Pseudomonas marginalis	hypothetical protein [Pseudomonas marginalis].
WP_063921074.1	-	388	bacteria>proteobacteria>gammaproteobacteria	Pseudomonas viridiflava	hypothetical protein [Pseudomonas viridiflava].
OCX95356.1	BCV62_14365	388	bacteria>proteobacteria>gammaproteobacteria	Pseudomonas sp. K35	hypothetical protein BCV62_14365 [Pseudomonas sp. K35].
WP_028239525.1	-	387	bacteria>proteobacteria>gammaproteobacteria	Pseudomonas azotifigens	hypothetical protein [Pseudomonas azotifigens].
WP_041104505.1	-	387	bacteria>proteobacteria>gammaproteobacteria	Pseudomonas stutzeri group	MULTISPECIES: hypothetical protein [Pseudomonas stutzeri group].
WP_043221030.1	-	387	bacteria>proteobacteria>gammaproteobacteria	Pseudomonas balearica	hypothetical protein [Pseudomonas balearica].
CJK44726.1	ERS022045_00214	387	bacteria>firmicutes	Streptococcus pneumoniae	Uncharacterised protein [Streptococcus pneumoniae].
WP_061337157.1	-	387	bacteria>proteobacteria>gammaproteobacteria	Pseudomonas balearica	hypothetical protein [Pseudomonas balearica].
WP_018167960.1	-	384	bacteria>proteobacteria>gammaproteobacteria	Thioalkalivibrio	MULTISPECIES: hypothetical protein [Thioalkalivibrio].
WP_018941681.1	-	384	bacteria>proteobacteria>gammaproteobacteria	Thioalkalivibrio sp. AKL11	hypothetical protein [Thioalkalivibrio sp. AKL11].
WP_018948852.1	-	384	bacteria>proteobacteria>gammaproteobacteria	Thioalkalivibrio sp. ALMg11	hypothetical protein [Thioalkalivibrio sp. ALMg11].
WP_019594886.1	-	384	bacteria>proteobacteria>gammaproteobacteria	Thioalkalivibrio sp. ALM2T	hypothetical protein [Thioalkalivibrio sp. ALM2T].
WP_019600464.1	-	384	bacteria>bacteroidetes	Rhodonellum psychrophilum	hypothetical protein [Rhodonellum psychrophilum].
WP_019627362.1	-	384	bacteria>proteobacteria>gammaproteobacteria	Thioalkalivibrio sp. AKL10	hypothetical protein [Thioalkalivibrio sp. AKL10].
WP_024326469.1	-	384	bacteria>proteobacteria>gammaproteobacteria	Thioalkalivibrio sp. AKL19	hypothetical protein [Thioalkalivibrio sp. AKL19].
WP_044117711.1	-	384	bacteria>bacteroidetes	Alkaliflexus imshenetskii	hypothetical protein [Alkaliflexus imshenetskii].
WP_051497679.1	-	384	bacteria>proteobacteria>gammaproteobacteria	Candidatus Contendobacter odensis	hypothetical protein [Candidatus Contendobacter odensis].
WP_051663980.1	-	384	bacteria>bacteroidetes	Dyadobacter crusticola	hypothetical protein [Dyadobacter crusticola].
WP_016988111.1	-	383	bacteria>bacteroidetes	Flavobacterium sp. ACAM 123	hypothetical protein [Flavobacterium sp. ACAM 123].
WP_026977713.1	-	383	bacteria>bacteroidetes	Flavobacterium tegetincola	hypothetical protein [Flavobacterium tegetincola].
WP_046851667.1	-	383	bacteria>proteobacteria>betaproteobacteria	Nitrosomonas communis	hypothetical protein [Nitrosomonas communis].
WP_058521739.1	-	382	bacteria>proteobacteria>gammaproteobacteria	Legionella tucsonensis	hypothetical protein [Legionella tucsonensis].
WP_003235861.1	-	375	bacteria>proteobacteria>gammaproteobacteria	Pseudomonas fluorescens	hypothetical protein [Pseudomonas fluorescens].
WP_054136995.1	-	375	bacteria>proteobacteria>betaproteobacteria	unclassified Betaproteobacteria (miscellaneous)	MULTISPECIES: hypothetical protein [unclassified Betaproteobacteria (miscellaneous)].
WP_057427733.1	-	375	bacteria>proteobacteria>gammaproteobacteria	Pseudomonas syringae	hypothetical protein [Pseudomonas syringae].
WP_057724992.1	-	375	bacteria>proteobacteria>gammaproteobacteria	Pseudomonas orientalis	hypothetical protein [Pseudomonas orientalis].
EKD25914.1	ACD_79C01445G0006	370	bacteria	uncultured bacterium	hypothetical protein ACD_79C01445G0006 [uncultured bacterium].
WP_035807289.1	-	384	bacteria>bacteroidetes	Lunatimonas lonarensis	hypothetical protein [Lunatimonas lonarensis].
WP_038698089.1	-	384	bacteria>bacteroidetes	Sphingobacterium sp. ML3W	hypothetical protein [Sphingobacterium sp. ML3W].
WP_052144032.1	-	351	bacteria>bacteroidetes	Sphingobacteriaceae bacterium DW12	hypothetical protein [Sphingobacteriaceae bacterium DW12].
WP_051292742.1	-	390	bacteria>bacteroidetes	Olivibacter sitiensis	hypothetical protein [Olivibacter sitiensis].
WP_011356875.1	-	382	bacteria>chlorobi	Pelodictyon luteolum	hypothetical protein [Pelodictyon luteolum].
ANT63926.1	Ptc2401_00116	391	bacteria>chlorobi	Prosthecochloris sp. CIB 2401	hypothetical protein Ptc2401_00116 [Prosthecochloris sp. CIB 2401].
WP_011889429.1	-	382	bacteria>chlorobi	Chlorobium phaeovibrioides	hypothetical protein [Chlorobium phaeovibrioides].
KXK36209.1	UZ06_CHB003000011	378	bacteria>chlorobi	Chlorobi bacterium OLB6	hypothetical protein UZ06_CHB003000011 [Chlorobi bacterium OLB6].
KZK73683.1	A3K90_00735	382	bacteria>chlorobi	Pelodictyon luteolum	hypothetical protein A3K90_00735 [Pelodictyon luteolum].
WP_010933801.1	-	381	bacteria>chlorobi	Chlorobaculum tepidum	hypothetical protein [Chlorobaculum tepidum].
WP_012501362.1	-	381	bacteria>chlorobi	Chlorobaculum parvum	hypothetical protein [Chlorobaculum parvum].
WP_012504746.1	-	381	bacteria>chlorobi	Prosthecochloris aestuarii	hypothetical protein [Prosthecochloris aestuarii].
WP_012473620.1	-	381	bacteria>chlorobi	Chlorobium phaeobacteroides	hypothetical protein [Chlorobium phaeobacteroides].
WP_006911457.1	-	384	bacteria>cyanobacteria	Cyanobium sp. PCC 7001	hypothetical protein [Cyanobium sp. PCC 7001].
WP_026393650.1	-	378	bacteria>firmicutes	Acetobacterium dehalogenans	hypothetical protein [Acetobacterium dehalogenans].
WP_026698614.1	-	381	bacteria>firmicutes	Bacillus chagannorensis	hypothetical protein [Bacillus chagannorensis].
WP_050739365.1	-	378	bacteria>firmicutes	Acetobacterium bakii	hypothetical protein [Acetobacterium bakii].
WP_051403485.1	-	378	bacteria>firmicutes	[Clostridium] ultunense	hypothetical protein [[Clostridium] ultunense].
WP_031575083.1	-	383	bacteria>firmicutes	Proteiniclasticum ruminis	hypothetical protein [Proteiniclasticum ruminis].
WP_051430948.1	-	378	bacteria>firmicutes	[Clostridium] ultunense	hypothetical protein [[Clostridium] ultunense].
WP_029052865.1	-	382	bacteria>firmicutes	Sporosarcina ureae	hypothetical protein [Sporosarcina ureae].
WP_014354865.1	-	378	bacteria>firmicutes	Acetobacterium woodii	hypothetical protein [Acetobacterium woodii].
CCQ92785.1	CULT_110022	382	bacteria>firmicutes	[Clostridium] ultunense Esp	conserved hypothetical protein [[Clostridium] ultunense Esp].
WP_013782277.1	-	384	bacteria>firmicutes	Mahella australiensis	hypothetical protein [Mahella australiensis].
WP_059031982.1	-	383	bacteria>firmicutes	Tepidanaerobacter syntrophicus	hypothetical protein [Tepidanaerobacter syntrophicus].
WP_026849032.1	-	388	bacteria>gemmatimonadetes	Gemmatimonas phototrophica	hypothetical protein [Gemmatimonas phototrophica].
AMV37310.1	VT85_07745	383	bacteria>planctomycetes	Planctomyces sp. SH-PL62	hypothetical protein VT85_07745 [Planctomyces sp. SH-PL62].
WP_013566068.1	-	383	bacteria>planctomycetes	Isosphaera pallida	hypothetical protein [Isosphaera pallida].
WP_046866880.1	-	382	bacteria>proteobacteria>alphaproteobacteria	Microvirga sp. JC119	hypothetical protein [Microvirga sp. JC119].
WP_050526057.1	-	382	bacteria>proteobacteria>alphaproteobacteria	Pseudorhodobacter aquimaris	hypothetical protein [Pseudorhodobacter aquimaris].
WP_031346136.1	-	382	bacteria>proteobacteria>betaproteobacteria	Thauera terpenica	hypothetical protein [Thauera terpenica].
WP_011633454.1	-	386	bacteria>proteobacteria>betaproteobacteria	Nitrosomonas eutropha	hypothetical protein [Nitrosomonas eutropha].
WP_053176706.1	-	379	bacteria>proteobacteria>betaproteobacteria	Limnohabitans planktonicus	hypothetical protein [Limnohabitans planktonicus].
WP_027994755.1	-	364	bacteria>proteobacteria>betaproteobacteria	Simplicispira psychrophila	hypothetical protein [Simplicispira psychrophila].
WP_019375314.1	-	357	bacteria>proteobacteria>betaproteobacteria	Alicycliphilus sp. CRZ1	hypothetical protein [Alicycliphilus sp. CRZ1].
WP_052043617.1	-	405	bacteria>proteobacteria>betaproteobacteria	Oligella urethralis	hypothetical protein [Oligella urethralis].
EPZ17149.1	M622_09370	336	bacteria>proteobacteria>betaproteobacteria	Thauera terpenica 58Eu	hypothetical protein M622_09370 [Thauera terpenica 58Eu].
ALK89261.1	L63ED372_02058	276	bacteria>proteobacteria>betaproteobacteria	Limnohabitans sp. 63ED37-2	hypothetical protein L63ED372_02058 [Limnohabitans sp. 63ED37-2].
WP_043812429.1	-	372	bacteria>proteobacteria>deltaproteobacteria	Desulfomicrobium baculatum	hypothetical protein [Desulfomicrobium baculatum].
KGO33141.1	JT06_16220	372	bacteria>proteobacteria>deltaproteobacteria	Desulfobulbus sp. Tol-SR	hypothetical protein JT06_16220 [Desulfobulbus sp. Tol-SR].
WP_045220646.1	-	384	bacteria>proteobacteria>deltaproteobacteria	Desulfonatronum thioautotrophicum	hypothetical protein [Desulfonatronum thioautotrophicum].
ACU90248.1	Dbac_2164	387	bacteria>proteobacteria>deltaproteobacteria	Desulfomicrobium baculatum DSM 4028	conserved hypothetical protein [Desulfomicrobium baculatum DSM 4028].
WP_045212238.1	-	384	bacteria>proteobacteria>deltaproteobacteria	Desulfonatronovibrio magnus	hypothetical protein [Desulfonatronovibrio magnus].
WP_031387355.1	-	384	bacteria>proteobacteria>deltaproteobacteria	Desulfonatronum thiodismutans	hypothetical protein [Desulfonatronum thiodismutans].
WP_028571751.1	-	384	bacteria>proteobacteria>deltaproteobacteria	Desulfonatronum lacustre	hypothetical protein [Desulfonatronum lacustre].
WP_005000040.1	-	382	bacteria>proteobacteria>gammaproteobacteria	Nitrococcus mobilis	hypothetical protein [Nitrococcus mobilis].
WP_013220342.1	-	384	bacteria>proteobacteria>gammaproteobacteria	Nitrosococcus watsonii	hypothetical protein [Nitrosococcus watsonii].
WP_046061147.1	-	386	bacteria>proteobacteria>gammaproteobacteria	Methylomicrobium alcaliphilum	hypothetical protein [Methylomicrobium alcaliphilum].
WP_025386347.1	-	382	bacteria>proteobacteria>gammaproteobacteria	Legionella oakridgensis	hypothetical protein [Legionella oakridgensis].
WP_017838785.1	-	386	bacteria>proteobacteria>gammaproteobacteria	Methylomicrobium buryatense	hypothetical protein [Methylomicrobium buryatense].
WP_036501203.1	-	383	bacteria>proteobacteria>gammaproteobacteria	Nitrosococcus oceani	hypothetical protein [Nitrosococcus oceani].
WP_042754943.1	-	382	bacteria>proteobacteria>gammaproteobacteria	Legionella pneumophila	hypothetical protein [Legionella pneumophila].
OCX95962.1	BFD77_07105	388	bacteria>proteobacteria>gammaproteobacteria	Pseudomonas sp. CO183	hypothetical protein BFD77_07105 [Pseudomonas sp. CO183].
WP_058793270.1	-	388	bacteria>proteobacteria>gammaproteobacteria	Pseudomonas psychrotolerans	hypothetical protein [Pseudomonas psychrotolerans].
WP_058483069.1	-	382	bacteria>proteobacteria>gammaproteobacteria	Legionella spiritensis	hypothetical protein [Legionella spiritensis].
WP_046555002.1	-	384	bacteria>proteobacteria>gammaproteobacteria	Arsukibacterium sp. MJ3	hypothetical protein [Arsukibacterium sp. MJ3].
WP_058790867.1	-	388	bacteria>proteobacteria>gammaproteobacteria	Pseudomonas psychrotolerans	hypothetical protein [Pseudomonas psychrotolerans].
WP_058785202.1	-	388	bacteria>proteobacteria>gammaproteobacteria	Pseudomonas psychrotolerans	hypothetical protein [Pseudomonas psychrotolerans].
WP_058779444.1	-	388	bacteria>proteobacteria>gammaproteobacteria	Pseudomonas psychrotolerans	hypothetical protein [Pseudomonas psychrotolerans].
WP_058772495.1	-	388	bacteria>proteobacteria>gammaproteobacteria	Pseudomonas psychrotolerans	hypothetical protein [Pseudomonas psychrotolerans].
WP_058766628.1	-	388	bacteria>proteobacteria>gammaproteobacteria	Pseudomonas psychrotolerans	hypothetical protein [Pseudomonas psychrotolerans].
WP_058762001.1	-	388	bacteria>proteobacteria>gammaproteobacteria	Pseudomonas psychrotolerans	hypothetical protein [Pseudomonas psychrotolerans].
WP_036193868.1	-	381	bacteria>proteobacteria>gammaproteobacteria	Lysobacter concretionis	hypothetical protein [Lysobacter concretionis].
WP_043959136.1	-	381	bacteria>proteobacteria>gammaproteobacteria	Lysobacter sp. A03	hypothetical protein [Lysobacter sp. A03].
WP_058500483.1	-	381	bacteria>proteobacteria>gammaproteobacteria	Legionella israelensis	hypothetical protein [Legionella israelensis].
WP_058077546.1	-	388	bacteria>proteobacteria>gammaproteobacteria	Pseudomonas sp. TTU2014-066ASC	hypothetical protein [Pseudomonas sp. TTU2014-066ASC].
WP_044341497.1	-	388	bacteria>proteobacteria>gammaproteobacteria	Pseudomonas oryzihabitans	hypothetical protein [Pseudomonas oryzihabitans].
WP_042136049.1	-	388	bacteria>proteobacteria>gammaproteobacteria	Pseudomonas oryzihabitans	hypothetical protein [Pseudomonas oryzihabitans].
WP_037023291.1	-	388	bacteria>proteobacteria>gammaproteobacteria	Pseudomonas sp. 20_BN	hypothetical protein [Pseudomonas sp. 20_BN].
WP_027602586.1	-	388	bacteria>proteobacteria>gammaproteobacteria	Pseudomonas	MULTISPECIES: hypothetical protein [Pseudomonas].
WP_027597067.1	-	388	bacteria>proteobacteria>gammaproteobacteria	Pseudomonas sp. MOIL14HWK12:I2	hypothetical protein [Pseudomonas sp. MOIL14HWK12:I2].
WP_014853629.1	-	388	bacteria>proteobacteria>gammaproteobacteria	Pseudomonas	MULTISPECIES: hypothetical protein [Pseudomonas].
WP_058529191.1	-	382	bacteria>proteobacteria>gammaproteobacteria	Legionella londiniensis	hypothetical protein [Legionella londiniensis].
WP_002809284.1	-	383	bacteria>proteobacteria>gammaproteobacteria	Nitrosococcus oceani	hypothetical protein [Nitrosococcus oceani].
WP_010797400.1	-	388	bacteria>proteobacteria>gammaproteobacteria	Pseudomonas	MULTISPECIES: hypothetical protein [Pseudomonas].
WP_007159469.1	-	388	bacteria>proteobacteria>gammaproteobacteria	Pseudomonas psychrotolerans	hypothetical protein [Pseudomonas psychrotolerans].
WP_044500148.1	-	390	bacteria>proteobacteria>gammaproteobacteria	Pseudomonas sp. 12M76_air	hypothetical protein [Pseudomonas sp. 12M76_air].
WP_036990615.1	-	390	bacteria>proteobacteria>gammaproteobacteria	Pseudomonas bauzanensis	hypothetical protein [Pseudomonas bauzanensis].
WP_026610124.1	-	383	bacteria>proteobacteria>gammaproteobacteria	Methylocaldum szegediense	hypothetical protein [Methylocaldum szegediense].
EAR20373.1	NB231_06860	413	bacteria>proteobacteria>gammaproteobacteria	Nitrococcus mobilis Nb-231	hypothetical protein NB231_06860 [Nitrococcus mobilis Nb-231].

7. family 8

##;baeRF1 family 8 follows the standard core architecture: codon_recognition_domain+baeRF1+Pelota.
##;The Pelota domain appears to contain the 2 beta-strand insert characteristic of the clade.

EOM77465.1	Rrhod_1142	596	bacteria>actinobacteria	Rhodococcus rhodnii LMG 5362	hypothetical protein Rrhod_1142 [Rhodococcus rhodnii LMG 5362].
KRQ08351.1	AOQ71_22990	404	bacteria>proteobacteria>alphaproteobacteria	Bradyrhizobium manausense	hypothetical protein AOQ71_22990 [Bradyrhizobium manausense].
WP_027531825.1	-	395	bacteria>proteobacteria>alphaproteobacteria	Bradyrhizobium sp. WSM3983	hypothetical protein [Bradyrhizobium sp. WSM3983].
WP_027563671.1	-	395	bacteria>proteobacteria>alphaproteobacteria	Bradyrhizobium	MULTISPECIES: hypothetical protein [Bradyrhizobium].
WP_057836342.1	-	395	bacteria>proteobacteria>alphaproteobacteria	Bradyrhizobium jicamae	hypothetical protein [Bradyrhizobium jicamae].
WP_003601669.1	-	393	bacteria>proteobacteria>alphaproteobacteria	Methylobacterium extorquens	hypothetical protein [Methylobacterium extorquens].
AMB43375.1	Y590_00575	393	bacteria>proteobacteria>alphaproteobacteria	Methylobacterium sp. AMS5	hypothetical protein Y590_00575 [Methylobacterium sp. AMS5].
WP_063694737.1	-	393	bacteria>proteobacteria>alphaproteobacteria	Bradyrhizobium stylosanthis	hypothetical protein [Bradyrhizobium stylosanthis].
WP_063781769.1	-	393	bacteria>proteobacteria>alphaproteobacteria	Bradyrhizobium viridifuturi	hypothetical protein [Bradyrhizobium viridifuturi].
WP_031936725.1	-	392	bacteria>actinobacteria	Rhodococcus defluvii	hypothetical protein [Rhodococcus defluvii].
WP_047407526.1	-	388	bacteria>actinobacteria	Herbiconiux sp. YR403	hypothetical protein [Herbiconiux sp. YR403].
WP_055835692.1	-	388	bacteria>actinobacteria	Leifsonia sp. Root4	hypothetical protein [Leifsonia sp. Root4].
AMB60615.1	AWU67_16305	388	bacteria>actinobacteria	Microterricola viridarii	hypothetical protein AWU67_16305 [Microterricola viridarii].
WP_060768186.1	-	385	bacteria>proteobacteria>alphaproteobacteria	Methylobacterium sp. AMS5	hypothetical protein [Methylobacterium sp. AMS5].
CCF63943.1	NOCYR_3178	384	bacteria>actinobacteria	Nocardia cyriacigeorgica GUH-2	conserved protein of unknown function [Nocardia cyriacigeorgica GUH-2].
WP_065351691.1	-	380	bacteria>actinobacteria	Rhodococcus sp. 008	hypothetical protein [Rhodococcus sp. 008].
WP_026936066.1	-	379	bacteria>actinobacteria	Gulosibacter molinativorax	hypothetical protein [Gulosibacter molinativorax].
WP_015101344.1	-	378	bacteria>actinobacteria	Saccharothrix espanaensis	hypothetical protein [Saccharothrix espanaensis].
WP_030149442.1	-	378	bacteria>actinobacteria	Mycetocola saprophilus	hypothetical protein [Mycetocola saprophilus].
WP_037056666.1	-	378	bacteria>actinobacteria	Pseudonocardia asaccharolytica	hypothetical protein [Pseudonocardia asaccharolytica].
WP_064294325.1	-	378	bacteria>actinobacteria	Plantibacter sp. H53	hypothetical protein [Plantibacter sp. H53].
ANP73672.1	PA27867_2733	378	bacteria>actinobacteria	Cryobacterium arcticum	hypothetical protein PA27867_2733 [Cryobacterium arcticum].
WP_010202244.1	-	377	bacteria>actinobacteria	Salinibacterium sp. PAMC 21357	hypothetical protein [Salinibacterium sp. PAMC 21357].
WP_021810139.1	-	377	bacteria>actinobacteria	Leifsonia rubra	hypothetical protein [Leifsonia rubra].
WP_029139038.1	-	377	bacteria>actinobacteria	Citricoccus sp. CH26A	hypothetical protein [Citricoccus sp. CH26A].
WP_056008417.1	-	377	bacteria>actinobacteria	Plantibacter	MULTISPECIES: hypothetical protein [Plantibacter].
WP_037047713.1	-	375	bacteria>actinobacteria	Pseudonocardia autotrophica	hypothetical protein [Pseudonocardia autotrophica].
WP_053547992.1	-	375	bacteria>actinobacteria	Microbacterium chocolatum	hypothetical protein [Microbacterium chocolatum].
WP_064955929.1	-	375	bacteria>actinobacteria	Microbacterium arborescens	hypothetical protein [Microbacterium arborescens].
WP_013675406.1	-	374	bacteria>actinobacteria	Pseudonocardia dioxanivorans	hypothetical protein [Pseudonocardia dioxanivorans].
WP_020505088.1	-	374	bacteria>proteobacteria>gammaproteobacteria	Lamprocystis purpurea	hypothetical protein [Lamprocystis purpurea].
WP_012590208.1	-	373	bacteria>proteobacteria>alphaproteobacteria	Methylocella silvestris	hypothetical protein [Methylocella silvestris].
WP_015110576.1	-	373	bacteria>cyanobacteria	Cyanobium gracile	hypothetical protein [Cyanobium gracile].
WP_015882585.1	-	373	bacteria>actinobacteria	Beutenbergia cavernae	hypothetical protein [Beutenbergia cavernae].
WP_017582764.1	-	373	bacteria>actinobacteria	Nocardiopsis valliformis	hypothetical protein [Nocardiopsis valliformis].
WP_017603222.1	-	373	bacteria>actinobacteria	Nocardiopsis alkaliphila	hypothetical protein [Nocardiopsis alkaliphila].
WP_018190562.1	-	373	bacteria>actinobacteria	Leifsonia sp. 109	hypothetical protein [Leifsonia sp. 109].
WP_019181024.1	-	373	bacteria>actinobacteria	Microbacterium yannicii	hypothetical protein [Microbacterium yannicii].
WP_019956906.1	-	373	bacteria>proteobacteria>alphaproteobacteria	Loktanella vestfoldensis	hypothetical protein [Loktanella vestfoldensis].
WP_019964754.1	-	373	bacteria>proteobacteria>alphaproteobacteria	Pannonibacter phragmitetus	hypothetical protein [Pannonibacter phragmitetus].
WP_020075061.1	-	373	bacteria>actinobacteria	Cryocola sp. 340MFSha3.1	hypothetical protein [Cryocola sp. 340MFSha3.1].
WP_021757990.1	-	373	bacteria>actinobacteria	Leifsonia aquatica	hypothetical protein [Leifsonia aquatica].
WP_024513205.1	-	373	bacteria>proteobacteria>alphaproteobacteria	Bradyrhizobium sp. ARR65	hypothetical protein [Bradyrhizobium sp. ARR65].
WP_025157327.1	-	373	bacteria>actinobacteria	Leifsonia aquatica	hypothetical protein [Leifsonia aquatica].
WP_028347136.1	-	373	bacteria>proteobacteria>alphaproteobacteria	Bradyrhizobium elkanii	hypothetical protein [Bradyrhizobium elkanii].
WP_029031364.1	-	373	bacteria>proteobacteria>alphaproteobacteria	Salinarimonas rosea	hypothetical protein [Salinarimonas rosea].
WP_029088960.1	-	373	bacteria>actinobacteria	Brevibacterium album	hypothetical protein [Brevibacterium album].
WP_036700807.1	-	373	bacteria>proteobacteria>alphaproteobacteria	Paracoccus sanguinis	hypothetical protein [Paracoccus sanguinis].
WP_036703368.1	-	373	bacteria>proteobacteria>alphaproteobacteria	Paracoccus sanguinis	hypothetical protein [Paracoccus sanguinis].
WP_036708647.1	-	373	bacteria>proteobacteria>alphaproteobacteria	Paracoccus sanguinis	hypothetical protein [Paracoccus sanguinis].
WP_036732483.1	-	373	bacteria>proteobacteria>alphaproteobacteria	Paracoccus sanguinis	hypothetical protein [Paracoccus sanguinis].
WP_036743069.1	-	373	bacteria>proteobacteria>alphaproteobacteria	Paracoccus halophilus	hypothetical protein [Paracoccus halophilus].
WP_043132865.1	-	373	bacteria>proteobacteria>alphaproteobacteria	Paracoccus sanguinis	hypothetical protein [Paracoccus sanguinis].
WP_046476122.1	-	373	bacteria>proteobacteria>alphaproteobacteria	Candidatus Filomicrobium marinum	hypothetical protein [Candidatus Filomicrobium marinum].
WP_046767352.1	-	373	bacteria>actinobacteria	Jiangella alkaliphila	hypothetical protein [Jiangella alkaliphila].
WP_048576503.1	-	373	bacteria>proteobacteria>alphaproteobacteria	Sphingomonas sp. MEA3-1	hypothetical protein [Sphingomonas sp. MEA3-1].
WP_050472836.1	-	373	bacteria>proteobacteria>alphaproteobacteria	Pannonibacter phragmitetus	hypothetical protein [Pannonibacter phragmitetus].
WP_054006467.1	-	373	bacteria>proteobacteria>alphaproteobacteria	Pseudorhodobacter psychrotolerans	hypothetical protein [Pseudorhodobacter psychrotolerans].
WP_055892745.1	-	373	bacteria>actinobacteria	Leifsonia sp. Root227	hypothetical protein [Leifsonia sp. Root227].
WP_055919735.1	-	373	bacteria>actinobacteria	Leifsonia sp. Leaf336	hypothetical protein [Leifsonia sp. Leaf336].
WP_057850330.1	-	373	bacteria>proteobacteria>alphaproteobacteria	Bradyrhizobium valentinum	hypothetical protein [Bradyrhizobium valentinum].
CRI63308.1	THIOKS11300010	373	bacteria>proteobacteria>gammaproteobacteria	Thiocapsa sp. KS1	conserved hypothetical protein [Thiocapsa sp. KS1].
WP_058899605.1	-	373	bacteria>proteobacteria>alphaproteobacteria	Pannonibacter phragmitetus	hypothetical protein [Pannonibacter phragmitetus].
WP_064108639.1	-	373	bacteria>actinobacteria	Leifsonia xyli	hypothetical protein [Leifsonia xyli].
WP_005516969.1	-	372	bacteria>actinobacteria	Rhodococcus equi	hypothetical protein [Rhodococcus equi].
WP_009800268.1	-	372	bacteria>proteobacteria>alphaproteobacteria	Nitrobacter sp. Nb-311A	hypothetical protein [Nitrobacter sp. Nb-311A].
WP_009800327.1	-	372	bacteria>proteobacteria>alphaproteobacteria	Nitrobacter sp. Nb-311A	hypothetical protein [Nitrobacter sp. Nb-311A].
WP_011505003.1	-	372	bacteria>proteobacteria>alphaproteobacteria	Nitrobacter hamburgensis	hypothetical protein [Nitrobacter hamburgensis].
WP_011509469.1	-	372	bacteria>proteobacteria>alphaproteobacteria	Nitrobacter hamburgensis	hypothetical protein [Nitrobacter hamburgensis].
WP_022966256.1	-	372	bacteria>proteobacteria>gammaproteobacteria	Pseudomonas caeni	hypothetical protein [Pseudomonas caeni].
WP_035599366.1	-	372	bacteria>proteobacteria>alphaproteobacteria	Hyphomonas polymorpha	hypothetical protein [Hyphomonas polymorpha].
WP_046105303.1	-	372	bacteria>proteobacteria>alphaproteobacteria	Devosia chinhatensis	hypothetical protein [Devosia chinhatensis].
WP_046862172.1	-	372	bacteria>proteobacteria>alphaproteobacteria	Microvirga sp. JC119	hypothetical protein [Microvirga sp. JC119].
WP_056567091.1	-	372	bacteria>proteobacteria>alphaproteobacteria	Mesorhizobium sp. Root172	hypothetical protein [Mesorhizobium sp. Root172].
KYH45219.1	AZH51_15235	372	bacteria>actinobacteria	Branchiibius sp. NY16-3462-2	hypothetical protein AZH51_15235 [Branchiibius sp. NY16-3462-2].
WP_064059407.1	-	372	bacteria>actinobacteria	Rhodococcus equi	hypothetical protein [Rhodococcus equi].
OAM78015.1	A3840_07630	372	bacteria>proteobacteria>alphaproteobacteria	Devosia sp. S37	hypothetical protein A3840_07630 [Devosia sp. S37].
WP_012803682.1	-	371	bacteria>actinobacteria	Brachybacterium faecium	hypothetical protein [Brachybacterium faecium].
WP_013112281.1	-	371	bacteria>planctomycetes	Planctopirus limnophila	hypothetical protein [Planctopirus limnophila].
WP_053207434.1	-	371	bacteria>actinobacteria	Jiangella muralis	hypothetical protein [Jiangella muralis].
WP_006170705.1	-	370	bacteria>cyanobacteria	Synechococcus sp. WH 5701	hypothetical protein [Synechococcus sp. WH 5701].
WP_006617630.1	-	370	bacteria>cyanobacteria	Arthrospira platensis	hypothetical protein [Arthrospira platensis].
WP_011209723.1	-	370	bacteria>actinobacteria	Nocardia farcinica	hypothetical protein [Nocardia farcinica].
WP_023786415.1	-	370	bacteria>proteobacteria>alphaproteobacteria	Hyphomicrobium nitrativorans	hypothetical protein [Hyphomicrobium nitrativorans].
WP_029535958.1	-	370	bacteria>actinobacteria	Saccharopolyspora spinosa	hypothetical protein [Saccharopolyspora spinosa].
WP_040714902.1	-	370	bacteria>actinobacteria	Nocardia takedensis	hypothetical protein [Nocardia takedensis].
CNG44824.1	ERS008523_04300	370	bacteria>proteobacteria>gammaproteobacteria	Yersinia frederiksenii	Uncharacterised protein [Yersinia frederiksenii].
WP_060590667.1	-	370	bacteria>actinobacteria	Nocardia farcinica	hypothetical protein [Nocardia farcinica].
WP_003942559.1	-	369	bacteria>actinobacteria	Rhodococcus	MULTISPECIES: hypothetical protein [Rhodococcus].
WP_007729184.1	-	369	bacteria>actinobacteria	Rhodococcus	MULTISPECIES: hypothetical protein [Rhodococcus].
WP_019746218.1	-	369	bacteria>actinobacteria	Rhodococcus erythropolis	hypothetical protein [Rhodococcus erythropolis].
WP_020967981.1	-	369	bacteria>actinobacteria	Rhodococcus	MULTISPECIES: hypothetical protein [Rhodococcus].
WP_021332615.1	-	369	bacteria>actinobacteria	Rhodococcus erythropolis	hypothetical protein [Rhodococcus erythropolis].
WP_024794904.1	-	369	bacteria>actinobacteria	Tomitella biformata	hypothetical protein [Tomitella biformata].
WP_025350499.1	-	369	bacteria>actinobacteria	Nocardia nova	hypothetical protein [Nocardia nova].
WP_030536714.1	-	369	bacteria>actinobacteria	Rhodococcus	MULTISPECIES: hypothetical protein [Rhodococcus].
WP_036508921.1	-	369	bacteria>actinobacteria	Nocardia	MULTISPECIES: hypothetical protein [Nocardia].
WP_040719816.1	-	369	bacteria>actinobacteria	Nocardia veterana	hypothetical protein [Nocardia veterana].
WP_042453370.1	-	369	bacteria>actinobacteria	Rhodococcus erythropolis	hypothetical protein [Rhodococcus erythropolis].
WP_042544924.1	-	369	bacteria>actinobacteria	Leucobacter komagatae	hypothetical protein [Leucobacter komagatae].
WP_046377647.1	-	369	bacteria>actinobacteria	Rhodococcus erythropolis	hypothetical protein [Rhodococcus erythropolis].
WP_047272674.1	-	369	bacteria>actinobacteria	Rhodococcus	MULTISPECIES: hypothetical protein [Rhodococcus].
WP_047890114.1	-	369	bacteria>actinobacteria	Rhodococcus sp. ARP2	hypothetical protein [Rhodococcus sp. ARP2].
WP_049700264.1	-	369	bacteria>actinobacteria	Gordonia jacobaea	hypothetical protein [Gordonia jacobaea].
WP_050656764.1	-	369	bacteria>actinobacteria	Rhodococcus	MULTISPECIES: hypothetical protein [Rhodococcus].
WP_054828211.1	-	369	bacteria>actinobacteria	Rhodococcus	MULTISPECIES: hypothetical protein [Rhodococcus].
WP_059060283.1	-	369	bacteria>actinobacteria	Leucobacter sp. G161	hypothetical protein [Leucobacter sp. G161].
WP_060938748.1	-	369	bacteria>actinobacteria	Rhodococcus erythropolis	hypothetical protein [Rhodococcus erythropolis].
WP_062967538.1	-	369	bacteria>actinobacteria	Nocardia africana	hypothetical protein [Nocardia africana].
WP_064864158.1	-	369	bacteria>actinobacteria	Gordonia	MULTISPECIES: hypothetical protein [Gordonia].
WP_030521578.1	-	368	bacteria>actinobacteria	Nocardia rhamnosiphila	hypothetical protein [Nocardia rhamnosiphila].
WP_033242270.1	-	368	bacteria>actinobacteria	Nocardia carnea	hypothetical protein [Nocardia carnea].
WP_039825673.1	-	368	bacteria>actinobacteria	Nocardia testacea	hypothetical protein [Nocardia testacea].
WP_040743600.1	-	368	bacteria>actinobacteria	Nocardia tenerifensis	hypothetical protein [Nocardia tenerifensis].
WP_040752984.1	-	368	bacteria>actinobacteria	Nocardia transvalensis	hypothetical protein [Nocardia transvalensis].
WP_062974576.1	-	368	bacteria>actinobacteria	Nocardia flavorosea	hypothetical protein [Nocardia flavorosea].
WP_062996104.1	-	368	bacteria>actinobacteria	Nocardia jinanensis	hypothetical protein [Nocardia jinanensis].
WP_063040305.1	-	368	bacteria>actinobacteria	Nocardia grenadensis	hypothetical protein [Nocardia grenadensis].
WP_063061111.1	-	368	bacteria>actinobacteria	Nocardia sienata	hypothetical protein [Nocardia sienata].
WP_063126843.1	-	368	bacteria>actinobacteria	Nocardia fusca	hypothetical protein [Nocardia fusca].
WP_004701170.1	-	367	bacteria>proteobacteria>gammaproteobacteria	Yersinia aldovae	hypothetical protein [Yersinia aldovae].
WP_004721797.1	-	367	bacteria>proteobacteria>gammaproteobacteria	Yersinia ruckeri	hypothetical protein [Yersinia ruckeri].
WP_012225816.1	-	367	bacteria>actinobacteria	Candidatus Microthrix parvicella	hypothetical protein [Candidatus Microthrix parvicella].
WP_019210099.1	-	367	bacteria>proteobacteria>gammaproteobacteria	Yersinia	MULTISPECIES: hypothetical protein [Yersinia].
WP_020377225.1	-	367	bacteria>actinobacteria	Candidatus Microthrix parvicella	hypothetical protein [Candidatus Microthrix parvicella].

WP_021807591.1	-	367	bacteria>proteobacteria>gammaproteobacteria	Serratia fonticola	hypothetical protein [Serratia fonticola].
WP_024365951.1	-	367	bacteria>actinobacteria	Arthrobacter sp. TB 26	hypothetical protein [Arthrobacter sp. TB 26].
WP_024487064.1	-	367	bacteria>proteobacteria>gammaproteobacteria	Serratia fonticola	hypothetical protein [Serratia fonticola].
WP_024531077.1	-	367	bacteria>proteobacteria>gammaproteobacteria	Serratia fonticola	hypothetical protein [Serratia fonticola].
WP_024913135.1	-	367	bacteria>proteobacteria>gammaproteobacteria	Chania multitudinisentens	hypothetical protein [Chania multitudinisentens].
WP_025123757.1	-	367	bacteria>proteobacteria>gammaproteobacteria	Serratia	MULTISPECIES: hypothetical protein [Serratia].
WP_027468318.1	-	367	bacteria>proteobacteria>betaproteobacteria	Deefgea rivuli	hypothetical protein [Deefgea rivuli].
WP_029095987.1	-	367	bacteria>proteobacteria>gammaproteobacteria	Budvicia aquatica	hypothetical protein [Budvicia aquatica].
WP_037399463.1	-	367	bacteria>proteobacteria>gammaproteobacteria	Serratia	MULTISPECIES: hypothetical protein [Serratia].
WP_038276058.1	-	367	bacteria>proteobacteria>gammaproteobacteria	Yersinia ruckeri	hypothetical protein [Yersinia ruckeri].
WP_040820030.1	-	367	bacteria>actinobacteria	Nocardia jiangxiensis	hypothetical protein [Nocardia jiangxiensis].
WP_042038439.1	-	367	bacteria>proteobacteria>gammaproteobacteria	Aeromonas popoffii	hypothetical protein [Aeromonas popoffii].
WP_042525148.1	-	367	bacteria>proteobacteria>gammaproteobacteria	Yersinia ruckeri	hypothetical protein [Yersinia ruckeri].
WP_042547887.1	-	367	bacteria>proteobacteria>gammaproteobacteria	Yersinia aldovae	hypothetical protein [Yersinia aldovae].
WP_043652673.1	-	367	bacteria>actinobacteria	Nocardia thailandica	hypothetical protein [Nocardia thailandica].
WP_045844140.1	-	367	bacteria>proteobacteria>gammaproteobacteria	Yersinia ruckeri	hypothetical protein [Yersinia ruckeri].
WP_049607234.1	-	367	bacteria>proteobacteria>gammaproteobacteria	Yersinia massiliensis	hypothetical protein [Yersinia massiliensis].
WP_049632990.1	-	367	bacteria>proteobacteria>gammaproteobacteria	Yersinia aldovae	hypothetical protein [Yersinia aldovae].
WP_050080731.1	-	367	bacteria>proteobacteria>gammaproteobacteria	Yersinia frederiksenii	hypothetical protein [Yersinia frederiksenii].
WP_050297658.1	-	367	bacteria>proteobacteria>gammaproteobacteria	Yersinia intermedia	hypothetical protein [Yersinia intermedia].
WP_056431994.1	-	367	bacteria>actinobacteria	Arthrobacter sp. Leaf69	hypothetical protein [Arthrobacter sp. Leaf69].
WP_057642984.1	-	367	bacteria>proteobacteria>gammaproteobacteria	Yersinia frederiksenii	hypothetical protein [Yersinia frederiksenii].
WP_057649425.1	-	367	bacteria>proteobacteria>gammaproteobacteria	Yersinia frederiksenii	hypothetical protein [Yersinia frederiksenii].
WP_059199505.1	-	367	bacteria>proteobacteria>gammaproteobacteria	Serratia fonticola	hypothetical protein [Serratia fonticola].
WP_065684065.1	-	367	bacteria>proteobacteria>gammaproteobacteria	Serratia sp. 14-2641	hypothetical protein [Serratia sp. 14-2641].
WP_040861479.1	-	366	bacteria>actinobacteria	Nocardia niigatensis	hypothetical protein [Nocardia niigatensis].
WP_050085798.1	-	366	bacteria>proteobacteria>gammaproteobacteria	Yersinia intermedia	hypothetical protein [Yersinia intermedia].
WP_050134739.1	-	366	bacteria>proteobacteria>gammaproteobacteria	Yersinia frederiksenii	hypothetical protein [Yersinia frederiksenii].
WP_006808924.1	-	365	bacteria>proteobacteria>gammaproteobacteria	Enterobacter hormaechei	hypothetical protein [Enterobacter hormaechei].
WP_012904958.1	-	365	bacteria>proteobacteria>gammaproteobacteria	Citrobacter rodentium	hypothetical protein [Citrobacter rodentium].
WP_039273648.1	-	365	bacteria>proteobacteria>gammaproteobacteria	Enterobacter cloacae complex	MULTISPECIES: hypothetical protein [Enterobacter cloacae complex].
WP_047353970.1	-	365	bacteria>proteobacteria>gammaproteobacteria	Enterobacter hormaechei	hypothetical protein [Enterobacter hormaechei].
WP_050109673.1	-	365	bacteria>proteobacteria>gammaproteobacteria	Yersinia frederiksenii	hypothetical protein [Yersinia frederiksenii].
WP_050121194.1	-	365	bacteria>proteobacteria>gammaproteobacteria	Yersinia frederiksenii	hypothetical protein [Yersinia frederiksenii].
WP_050136318.1	-	365	bacteria>proteobacteria>gammaproteobacteria	Yersinia frederiksenii	hypothetical protein [Yersinia frederiksenii].
WP_050146209.1	-	365	bacteria>proteobacteria>gammaproteobacteria	Yersinia frederiksenii	hypothetical protein [Yersinia frederiksenii].
WP_050151108.1	-	365	bacteria>proteobacteria>gammaproteobacteria	Yersinia frederiksenii	hypothetical protein [Yersinia frederiksenii].
WP_050318903.1	-	365	bacteria>proteobacteria>gammaproteobacteria	Yersinia frederiksenii	hypothetical protein [Yersinia frederiksenii].
WP_059593924.1	-	363	bacteria>proteobacteria>gammaproteobacteria	Enterobacter hormaechei	hypothetical protein [Enterobacter hormaechei].
WP_037496969.1	-	359	bacteria>actinobacteria	Solirubrobacter soli	hypothetical protein [Solirubrobacter soli].
WP_039781952.1	-	267	bacteria>actinobacteria	Nocardia cerradoensis	hypothetical protein, partial [Nocardia cerradoensis].
WP_063002017.1	-	369	bacteria>actinobacteria	Nocardia mikamii	hypothetical protein [Nocardia mikamii].
WP_063008624.1	-	369	bacteria>actinobacteria	Nocardia kruczakiae	hypothetical protein [Nocardia kruczakiae].
WP_030516373.1	-	368	bacteria>actinobacteria	Nocardia sp. NRRL WC-3656	hypothetical protein [Nocardia sp. NRRL WC-3656].
WP_063018409.1	-	368	bacteria>actinobacteria	Corynebacteriales	MULTISPECIES: hypothetical protein [Corynebacteriales].
WP_063026147.1	-	368	bacteria>actinobacteria	Nocardia elegans	hypothetical protein [Nocardia elegans].

8. family 9

##;baeRF1 family 9 follows the standard core architecture: codon_recognition_domain+baeRF1+Pelota.
##;The Pelota domain appears to contain the 2 beta-strand insert characteristic of the clade.

WP_038026945.1	-	396	bacteria>firmicutes	Tetragenococcus muriaticus	hypothetical protein, partial [Tetragenococcus muriaticus].
WP_044010800.1	-	391	bacteria>firmicutes	Lactobacillus wasatchensis	hypothetical protein [Lactobacillus wasatchensis].
ETC91310.1	T481_13410	384	bacteria>firmicutes	Enterococcus faecalis PF3	hypothetical protein T481_13410 [Enterococcus faecalis PF3].
WP_041093179.1	-	383	bacteria>firmicutes	Lactobacillus hokkaidonensis	hypothetical protein [Lactobacillus hokkaidonensis].
WP_046871024.1	-	383	bacteria>firmicutes	Pediococcus damnosus	hypothetical protein [Pediococcus damnosus].
WP_056986022.1	-	383	bacteria>firmicutes	Pediococcus damnosus	hypothetical protein [Pediococcus damnosus].
WP_057750895.1	-	383	bacteria>firmicutes	Pediococcus cellicola	hypothetical protein [Pediococcus cellicola].
WP_057774251.1	-	383	bacteria>firmicutes	Pediococcus inopinatus	hypothetical protein [Pediococcus inopinatus].
WP_057807669.1	-	383	bacteria>firmicutes	Pediococcus ethanolidurans	hypothetical protein [Pediococcus ethanolidurans].
WP_062903999.1	-	383	bacteria>firmicutes	Pediococcus damnosus	hypothetical protein [Pediococcus damnosus].
OAD63552.1	A7K95_09060	383	bacteria>firmicutes	Pediococcus parvulus	hypothetical protein A7K95_09060 [Pediococcus parvulus].
WP_063696507.1	-	383	bacteria>firmicutes	Pediococcus inopinatus	hypothetical protein [Pediococcus inopinatus].

[illegible]

SDQ25433.1	SAMN04489842_0237	316	archaea>euryarchaeota	Natronobacterium texcoconense	hypothetical protein SAMN04489842_0237 [Natronobacterium texcoconense]
WP_005581432.1	-	316	archaea>euryarchaeota	Natronobacterium gregoryi	hypothetical protein [Natronobacterium gregoryi]
WP_008895965.1	-	316	archaea>euryarchaeota	Haloterrigena salina	hypothetical protein [Haloterrigena salina]
WP_049987168.1	-	316	archaea>euryarchaeota	Halobellus rufus	hypothetical protein [Halobellus rufus]
ERG93079.1	J07HQW1_03134	313	archaea>euryarchaeota	Haloquadratum walsbyi J07HQW1	hypothetical protein J07HQW1_03134 [Haloquadratum walsbyi J07HQW1]
SFS68163.1	SAMN04488556_2117	313	archaea>euryarchaeota	Halostagnicola kamekurae	hypothetical protein SAMN04488556_2117 [Halostagnicola kamekurae]
WP_049951840.1	-	313	archaea>euryarchaeota	Halostagnicola larsenii	hypothetical protein [Halostagnicola larsenii]
WP_050051529.1	-	313	archaea>euryarchaeota	Halostagnicola sp. A56	hypothetical protein [Halostagnicola sp. A56]
WP_006110472.1	-	312	archaea>euryarchaeota	Natrialba asiatica	hypothetical protein [Natrialba asiatica]
WP_006664878.1	-	312	archaea>euryarchaeota	Natrialba aegyptia	hypothetical protein [Natrialba aegyptia]
WP_006826356.1	-	312	archaea>euryarchaeota	Natrialba taiwanensis	hypothetical protein [Natrialba taiwanensis]
WP_007275268.1	-	312	archaea>euryarchaeota	Haloferax sulfurifontis	hypothetical protein [Haloferax sulfurifontis]
WP_049922449.1	-	312	archaea>euryarchaeota	Halopiger djelfimassiliensis	hypothetical protein [Halopiger djelfimassiliensis]
WP_050459720.1	-	312	archaea>euryarchaeota	Haloferax gibbonsii	hypothetical protein [Haloferax gibbonsii]
WP_076609855.1	-	312	archaea>euryarchaeota	Natronorubrum thiooxidans	hypothetical protein [Natronorubrum thiooxidans]
ERH11069.1	J07HX64_02848	311	archaea>euryarchaeota	halophilic archaeon J07HX64	hypothetical protein J07HX64_02848 [halophilic archaeon J07HX64]
WP_004969506.1	-	311	archaea>euryarchaeota	Haloferax denitrificans	hypothetical protein [Haloferax denitrificans]
WP_006064982.1	-	311	archaea>euryarchaeota	Natronorubrum bangense	hypothetical protein [Natronorubrum bangense]
WP_008160635.1	-	311	archaea>euryarchaeota	Natronorubrum sulfidifaciens	hypothetical protein [Natronorubrum sulfidifaciens]
WP_049928793.1	-	311	archaea>euryarchaeota	Halopiger goleimassiliensis	hypothetical protein [Halopiger goleimassiliensis]
SEH16584.1	SAMN04487967_2723	310	archaea>euryarchaeota	Natronorubrum sediminis	hypothetical protein SAMN04487967_2723 [Natronorubrum sediminis]
SEH52655.1	SAMN05192561_104166	310	archaea>euryarchaeota	Halopenitus malekzadehii	hypothetical protein SAMN05192561_104166 [Halopenitus malekzadehii]
SER70680.1	SAMN04489841_4356	310	archaea>euryarchaeota	Natrinema salaciae	hypothetical protein SAMN04489841_4356 [Natrinema salaciae]
WP_004974766.1	-	310	archaea>euryarchaeota	Haloferax gibbonsii	hypothetical protein [Haloferax gibbonsii]
WP_006186305.1	-	310	archaea>euryarchaeota	Natrinema pallidum	hypothetical protein [Natrinema pallidum]
WP_006429422.1	-	310	archaea>euryarchaeota	Natrinema versiforme	hypothetical protein [Natrinema versiforme]
WP_007107793.1	-	310	archaea>euryarchaeota	Natrinema altunense	hypothetical protein [Natrinema altunense]
WP_008095161.1	-	310	archaea>euryarchaeota	Haloferax prahovense	hypothetical protein [Haloferax prahovense]
WP_008457598.1	-	310	archaea>euryarchaeota	Natrinema gari	hypothetical protein [Natrinema gari]
WP_008575975.1	-	310	archaea>euryarchaeota	Haloferax	MULTISPECIES: hypothetical protein [Haloferax]
WP_014865453.1	-	310	archaea>euryarchaeota	Natrinema sp. J7-2	hypothetical protein [Natrinema sp. J7-2]
WP_049899774.1	-	310	archaea>euryarchaeota	Natrinema sp. J7-1	hypothetical protein [Natrinema sp. J7-1]
WP_049967787.1	-	310	archaea>euryarchaeota	Haloferax alexandrinus	hypothetical protein [Haloferax alexandrinus]
WP_049988962.1	-	310	archaea>euryarchaeota	Halopiger salifodinae	hypothetical protein [Halopiger salifodinae]
WP_058569100.1	-	310	archaea>euryarchaeota	Haloferax sp. SB3	hypothetical protein [Haloferax sp. SB3]
WP_076581430.1	-	310	archaea>euryarchaeota	Haloterrigena daqingensis	hypothetical protein [Haloterrigena daqingensis]
ERG95259.1	J07HQW2_01710	309	archaea>euryarchaeota	Haloquadratum walsbyi J07HQW2	hypothetical protein J07HQW2_01710 [Haloquadratum walsbyi J07HQW2]
SDY02415.1	SAMN04487946_105158	309	archaea>euryarchaeota	Halobellus clavatus	hypothetical protein SAMN04487946_105158 [Halobellus clavatus]
SET88529.1	SAMN04488694_11575	309	archaea>euryarchaeota	Haloterrigena hispanica	hypothetical protein SAMN04488694_11575 [Haloterrigena hispanica]
WP_004042761.1	-	309	archaea>euryarchaeota	Haloferax volcanii	hypothetical protein [Haloferax volcanii]
WP_004062340.1	-	309	archaea>euryarchaeota	Haloferax	MULTISPECIES: hypothetical protein [Haloferax]
WP_008009460.1	-	309	archaea>euryarchaeota	Haloterrigena limicola	hypothetical protein [Haloterrigena limicola]
WP_008848510.1	-	309	archaea>euryarchaeota	Halorubrum kocurii	hypothetical protein [Halorubrum kocurii]
WP_004060142.1	-	308	archaea>euryarchaeota	Haloferax mediterranei	hypothetical protein [Haloferax mediterranei]
WP_049982407.1	-	307	archaea>euryarchaeota	Halorubrum sp. BV1	hypothetical protein [Halorubrum sp. BV1]
WP_050024754.1	-	307	archaea>euryarchaeota	Halorubrum saccharovorum	hypothetical protein [Halorubrum saccharovorum]
WP_050032629.1	-	307	archaea>euryarchaeota	Halorubrum halophilum	hypothetical protein [Halorubrum halophilum]
WP_066418022.1	-	307	archaea>euryarchaeota	Halorubrum sp. SAH-A6	hypothetical protein [Halorubrum sp. SAH-A6]
AGB15362.1	Halru_0735	306	archaea>euryarchaeota	Halovivax ruber XH-70	hypothetical protein Halru_0735 [Halovivax ruber XH-70]
WP_006181287.1	-	306	archaea>euryarchaeota	Natrinema pellirubrum	hypothetical protein, partial [Natrinema pellirubrum]
WP_006650250.1	-	306	archaea>euryarchaeota	Haloterrigena thermotolerans	hypothetical protein, partial [Haloterrigena thermotolerans]
WP_007698776.1	-	306	archaea>euryarchaeota	Halovivax asiaticus	hypothetical protein [Halovivax asiaticus]
WP_049964848.1	-	306	archaea>euryarchaeota	Haloterrigena jeotgali	hypothetical protein [Haloterrigena jeotgali]
WP_066300805.1	-	306	archaea>euryarchaeota	Haloterrigena mahii	hypothetical protein [Haloterrigena mahii]
WP_076146684.1	-	306	archaea>euryarchaeota	Haloterrigena saccharevitans	hypothetical protein [Haloterrigena saccharevitans]
ABQ76087.1	-	305	archaea>euryarchaeota	uncultured haloarchaeon	conserved hypothetical protein [uncultured haloarchaeon]
WP_011572236.1	-	305	archaea>euryarchaeota	Haloquadratum walsbyi	hypothetical protein [Haloquadratum walsbyi]
WP_014556704.1	-	305	archaea>euryarchaeota	Haloquadratum walsbyi	hypothetical protein [Haloquadratum walsbyi]
ERH02953.1	J07HN6_00271	303	archaea>euryarchaeota	Halonotius sp. J07HN6	hypothetical protein J07HN6_00271 [Halonotius sp. J07HN6]
ERH02957.1	J07HN6_00275	303	archaea>euryarchaeota	Halonotius sp. J07HN6	peptide chain release factor 1 (eRF1) [Halonotius sp. J07HN6]
ERH07346.1	J07HN4v3_02986	303	archaea>euryarchaeota	Halonotius sp. J07HN4	hypothetical protein J07HN4v3_02986 [Halonotius sp. J07HN4]
WP_004046256.1	-	303	archaea>euryarchaeota	Halorubrum saccharovorum	hypothetical protein [Halorubrum saccharovorum]
WP_005554226.1	-	303	archaea>euryarchaeota	Natronococcus amylolyticus	hypothetical protein [Natronococcus amylolyticus]
WP_007543258.1	-	303	archaea>euryarchaeota	Haloferax larsenii	hypothetical protein [Haloferax larsenii]
WP_008002859.1	-	303	archaea>euryarchaeota	Halorubrum lipolyticum	hypothetical protein [Halorubrum lipolyticum]
WP_008325450.1	-	303	archaea>euryarchaeota	Haloferax elongans	hypothetical protein [Haloferax elongans]
WP_008427474.1	-	303	archaea>euryarchaeota	Natronococcus jeotgali	hypothetical protein [Natronococcus jeotgali]
WP_015319739.1	-	303	archaea>euryarchaeota	Natronococcus occultus	hypothetical protein [Natronococcus occultus]
WP_073307302.1	-	303	archaea>euryarchaeota	Halobaculum gomorrense	hypothetical protein [Halobaculum gomorrense]
WP_074796365.1	-	303	archaea>euryarchaeota	Haloferax larsenii	hypothetical protein [Haloferax larsenii]
SDQ15803.1	SAMN05216278_0698	302	archaea>euryarchaeota	Halopelagius longus	hypothetical protein SAMN05216278_0698 [Halopelagius longus]
SFG46495.1	SAMN04488063_2135	302	archaea>euryarchaeota	Halopelagius inordinatus	hypothetical protein SAMN04488063_2135 [Halopelagius inordinatus]
WP_006112368.1	-	302	archaea>euryarchaeota	Halorubrum coriense	hypothetical protein [Halorubrum coriense]
WP_006628022.1	-	302	archaea>euryarchaeota	Halorubrum tebenquichense	hypothetical protein [Halorubrum tebenquichense]
WP_007345088.1	-	302	archaea>euryarchaeota	Halorubrum terrestre	hypothetical protein [Halorubrum terrestre]
WP_007996931.1	-	302	archaea>euryarchaeota	Halorubrum arcis	hypothetical protein [Halorubrum arcis]
WP_008000023.1	-	302	archaea>euryarchaeota	Halorubrum aidingense	hypothetical protein [Halorubrum aidingense]
WP_008367823.1	-	302	archaea>euryarchaeota	Halorubrum litoreum	hypothetical protein [Halorubrum litoreum]
WP_008446079.1	-	302	archaea>euryarchaeota	Halorubrum californiense	hypothetical protein [Halorubrum californiense]
WP_008583613.1	-	302	archaea>euryarchaeota	Halorubrum hochstenium	hypothetical protein [Halorubrum hochstenium]
WP_015910327.1	-	302	archaea>euryarchaeota	Halorubrum lacusprofundi	hypothetical protein [Halorubrum lacusprofundi]
WP_017344350.1	-	302	archaea>euryarchaeota	Halorubrum sp. T3	hypothetical protein [Halorubrum sp. T3]
WP_049930803.1	-	302	archaea>euryarchaeota	Halorubrum ezzemoulense	hypothetical protein [Halorubrum ezzemoulense]
WP_053771054.1	-	302	archaea>euryarchaeota	Halorubrum tropicale	hypothetical protein [Halorubrum tropicale]
SEJ28263.1	SAMN05444271_13923	301	archaea>euryarchaeota	Halohasta litchfieldiae	hypothetical protein SAMN05444271_13923 [Halohasta litchfieldiae]
SEF74472.1	SAMN04488133_0606	300	archaea>euryarchaeota	Halobellus limi	hypothetical protein SAMN04488133_0606 [Halobellus limi]
WP_020445079.1	-	300	archaea>euryarchaeota	Salinarchaeum sp. Harcht-Bsk1	hypothetical protein [Salinarchaeum sp. Harcht-Bsk1]
WP_049981964.1	-	300	archaea>euryarchaeota	Halolamina rubra	hypothetical protein [Halolamina rubra]
WP_053949077.1	-	300	archaea>euryarchaeota	Halolamina sediminis	hypothetical protein [Halolamina sediminis]
WP_058366229.1	-	300	archaea>euryarchaeota	Halopenitus sp. DYS4	hypothetical protein [Halopenitus sp. DYS4]
WP_058983887.1	-	300	archaea>euryarchaeota	Halobacterium sp. CBA1132	hypothetical protein [Halobacterium sp. CBA1132]
WP_059057394.1	-	300	archaea>euryarchaeota	Halobacterium hubeiense	hypothetical protein [Halobacterium hubeiense]
WP_074878466.1	-	300	archaea>euryarchaeota	Halolamina pelagica	hypothetical protein [Halolamina pelagica]
ESS04104.1	A07HR67_01090	299	archaea	uncultured archaeon A07HR67	hypothetical protein A07HR67_01090 [uncultured archaeon A07HR67]
ESS13033.1	A07HR60_00042	299	archaea	uncultured archaeon A07HR60	hypothetical protein A07HR60_00042 [uncultured archaeon A07HR60]
SFR31087.1	SAMN04487937_0925	299	archaea>euryarchaeota	Halorubrum sodomense	hypothetical protein SAMN04487937_0925 [Halorubrum sodomense]
WP_014051446.1	-	299	archaea	halophilic archaeon DL31	hypothetical protein [halophilic archaeon DL31]
WP_023394624.1	-	299	archaea>euryarchaeota	Candidatus Halobonum tyrrellensis	hypothetical protein [Candidatus Halobonum tyrrellensis]
WP_049930002.1	-	299	archaea>euryarchaeota	Halosimplex carlsbadense	hypothetical protein [Halosimplex carlsbadense]
WP_054582796.1	-	299	archaea>euryarchaeota	Halolamina pelagica	hypothetical protein [Halolamina pelagica]
WP_058572004.1	-	299	archaea>euryarchaeota	Haloferax sp. SB29	hypothetical protein [Haloferax sp. SB29]
SFK74530.1	SAMN04487950_0883	298	archaea>euryarchaeota	Halogranum rubrum	eRF1 domain-containing protein 2 [Halogranum rubrum]
SFR55147.1	SAMN04488124_2358	298	archaea>euryarchaeota	Halogeometricum limi	hypothetical protein SAMN04488124_2358 [Halogeometricum limi]
SFR57981.1	SAMN04487947_2507	298	archaea>euryarchaeota	Halogeometricum rufum	hypothetical protein SAMN04487947_2507 [Halogeometricum rufum]
WP_006053754.1	-	298	archaea>euryarchaeota	Halogeometricum borinquense	hypothetical protein [Halogeometricum borinquense]
WP_008384895.1	-	298	archaea>euryarchaeota	Halogeometricum pallidum	hypothetical protein [Halogeometricum pallidum]
WP_009375961.1	-	298	archaea>euryarchaeota	Halogranum salarium	hypothetical protein [Halogranum salarium]
WP_079233729.1	-	298	archaea>euryarchaeota	Halolamina sp. CBA1230	hypothetical protein [Halolamina sp. CBA1230]
CQR49437.1	BN996_00898	297	archaea>euryarchaeota	Haloferax massiliensis	hypothetical protein BN996_00898 [Haloferax massiliensis]
SDJ50251.1	SAMN05216226_104117	297	archaea>euryarchaeota	Halovenus aranensis	hypothetical protein SAMN05216226_104117 [Halovenus aranensis]
SDN34905.1	SAMN05192554_12836	297	archaea>euryarchaeota	Haloarchaeobius iranensis	hypothetical protein SAMN05192554_12836 [Haloarchaeobius iranensis]
WP_004593708.1	-	297	archaea>euryarchaeota	Haloarcula japonica	hypothetical protein [Haloarcula japonica]
WP_004963737.1	-	297	archaea>euryarchaeota	Haloarcula sinaiiensis	hypothetical protein [Haloarcula sinaiiensis]
WP_007189033.1	-	297	archaea>euryarchaeota	Haloarcula californiae	hypothetical protein [Haloarcula californiae]
WP_014039632.1	-	297	archaea>euryarchaeota	Haloarcula hispanica	hypothetical protein [Haloarcula hispanica]
WP_050037606.1	-	297	archaea>euryarchaeota	Haloarcula	MULTISPECIES: hypothetical protein [Haloarcula]
WP_053966223.1	-	297	archaea>euryarchaeota	Haloarcula rubripromontorii	hypothetical protein [Haloarcula rubripromontorii]
WP_058582317.1	-	297	archaea>euryarchaeota	Haloprofundus marisrubri	hypothetical protein [Haloprofundus marisrubri]
WP_058997280.1	-	297	archaea>euryarchaeota	Haloarcula sp. CBA1127	hypothetical protein [Haloarcula sp. CBA1127]
WP_064288263.1	-	297	archaea>euryarchaeota	Haloarcula sp. K1	hypothetical protein [Haloarcula sp. K1]
WP_005535833.1	-	296	archaea>euryarchaeota	Haloarcula argentinensis	hypothetical protein [Haloarcula argentinensis]
WP_015409124.1	-	296	archaea>euryarchaeota	Natronomonas moolapensis	hypothetical protein [Natronomonas moolapensis]
ELZ29305.1	C475_03774	295	archaea>euryarchaeota	Halosimplex carlsbadense 2-9-1	hypothetical protein C475_03774 [Halosimplex carlsbadense 2-9-1]
SDF05751.1	SAMN05216218_103217	295	archaea>euryarchaeota	Haloorientalis regularis	hypothetical protein SAMN05216218_103217 [Haloorientalis regularis]
SDM56594.1	SAMN04487949_2103	295	archaea>euryarchaeota	Halogranum gelatinilyticum	eRF1 domain-containing protein 2 [Halogranum gelatinilyticum]
SEN08965.1	SAMN05216388_1001340	295	archaea>euryarchaeota	Haloorientalis persicus	hypothetical protein SAMN05216388_1001340 [Haloorientalis persicus]
SEO25577.1	SAMN04487948_101366	295	archaea>euryarchaeota	Halogranum amylolyticum	hypothetical protein SAMN04487948_101366 [Halogranum amylolyticum]
WP_007976995.1	-	295	archaea>euryarchaeota	Haladaptatus paucihalophilus	hypothetical protein [Haladaptatus paucihalophilus]
WP_049969708.1	-	295	archaea>euryarchaeota	Haladaptatus cibarius	hypothetical protein [Haladaptatus cibarius]
WP_050049045.1	-	295	archaea>euryarchaeota	Halanaeroarchaeum sulfurireducens	hypothetical protein [Halanaeroarchaeum sulfurireducens]
WP_066141020.1	-	295	archaea>euryarchaeota	Haladaptatus sp. R4	hypothetical protein [Haladaptatus sp. R4]
WP_069450914.1	-	295	archaea>euryarchaeota	Haladaptatus sp. W1	hypothetical protein [Haladaptatus sp. W1]
WP_076430132.1	-	295	archaea>euryarchaeota	Haladaptatus litoreus	hypothetical protein [Haladaptatus litoreus]
WP_070364250.1	-	294	archaea>euryarchaeota	Halodesulfurarchaeum formicicum	hypothetical protein [Halodesulfurarchaeum formicicum]
WP_075938195.1	-	293	archaea>euryarchaeota	Natronomonas sp. CBA1134	hypothetical protein [Natronomonas sp. CBA1134]
WP_008414893.1	-	292	archaea>euryarchaeota	Halalkalicoccus jeotgali	hypothetical protein [Halalkalicoccus jeotgali]
WP_011322959.1	-	292	archaea>euryarchaeota	Natronomonas pharaonis	hypothetical protein [Natronomonas pharaonis]
WP_066381212.1	-	292	archaea>euryarchaeota	Halalkalicoccus paucihalophilus	hypothetical protein [Halalkalicoccus paucihalophilus]
SDW12429.1	SAMN05443574_101434	194	archaea>euryarchaeota	Haloarcula vallismortis	hypothetical protein SAMN05443574_101434 [Haloarcula vallismortis]
ELK46789.1	D320_20509	176	archaea>euryarchaeota	Haloferax sp. BAB2207	hypothetical protein D320_20509, partial [Haloferax sp. BAB2207]
WP_015052925.1	-	365	archaea>euryarchaeota	Methanolobus psychrophilus	hypothetical protein [Methanolobus psychrophilus]
WP_082090490.1	-	365	archaea>euryarchaeota	Methanosarcina sp. MTP4	peptide chain release factor-like protein [Methanosarcina sp. MTP4]
AKB74025.1	MSLAZ_0764	361	archaea>euryarchaeota	Methanosarcina lacustris Z-7289	hypothetical protein MSLAZ_0764 [Methanosarcina lacustris Z-7289]
SFM92597.1	SAMN04488696_2897	357	archaea>euryarchaeota	Methanolobus profundi	eRF1 domain-containing protein 2 [Methanolobus profundi]
AKB80623.1	MSBR3_0045	351	archaea>euryarchaeota	Methanosarcina barkeri 3	hypothetical protein MSBR3_0045 [Methanosarcina barkeri 3]
WP_011021302.1	-	347	archaea>euryarchaeota	Methanosarcina acetivorans	hypothetical protein [Methanosarcina acetivorans]
WP_015412441.1	-	347	archaea>euryarchaeota	Methanosarcina mazei	peptide chain release factor-like protein [Methanosarcina mazei]
WP_048044657.1	-	347	archaea>euryarchaeota	Methanosarcina mazei	peptide chain release factor-like protein [Methanosarcina mazei]
WP_048046035.1	-	347	archaea>euryarchaeota	Methanosarcina mazei	peptide chain release factor-like protein [Methanosarcina mazei]
WP_048046998.1	-	347	archaea>euryarchaeota	Methanosarcina mazei	peptide chain release factor-like protein [Methanosarcina mazei]
WP_048051696.1	-	347	archaea>euryarchaeota	Methanosarcina soligelidi	peptide chain release factor-like protein [Methanosarcina soligelidi]
WP_048128853.1	-	347	archaea>euryarchaeota	Methanosarcina	MULTISPECIES: peptide chain release factor-like protein [Methanosarcina]
WP_048133494.1	-	347	archaea>euryarchaeota	Methanosarcina sp. 1.H.T.1A.1	peptide chain release factor-like protein [Methanosarcina sp. 1.H.T.1A.1]
WP_048138005.1	-	347	archaea>euryarchaeota	Methanosarcina horonobensis	peptide chain release factor-like protein [Methanosarcina horonobensis]
WP_048141322.1	-	347	archaea>euryarchaeota	Methanosarcina	MULTISPECIES: peptide chain release factor-like protein [Methanosarcina]
WP_048162052.1	-	347	archaea>euryarchaeota	Methanosarcina sp. 1.H.A.2.2	peptide chain release factor-like protein [Methanosarcina sp. 1.H.A.2.2]
WP_048170540.1	-	347	archaea>euryarchaeota	Methanosarcina siciliae	peptide chain release factor-like protein [Methanosarcina siciliae]
WP_048171083.1	-	347	archaea>euryarchaeota	Methanosarcina sp. 2.H.A.1B.4	peptide chain release factor-like protein [Methanosarcina sp. 2.H.A.1B.4]
WP_048180745.1	-	347	archaea>euryarchaeota	Methanosarcina siciliae	peptide chain release factor-like protein [Methanosarcina siciliae]
AKB12744.1	MSTHT_0986	346	archaea>euryarchaeota	Methanosarcina thermophila TM-1	hypothetical protein MSTHT_0986 [Methanosarcina thermophila TM-1]
ODS39140.1	A7316_00625	346	archaea>euryarchaeota	Candidatus Altiarchaeales archaeon WOR_SM1_86-2	hypothetical protein A7316_00625 [Candidatus Altiarchaeales archaeon WOR_SM1_86-2]
WP_083812307.1	-	344	archaea>euryarchaeota	Methanosalsum zhilinae	hypothetical protein [Methanosalsum zhilinae]
KXS45071.1	AWU59_145	341	archaea>euryarchaeota	Methanolobus sp. T82-4	hypothetical protein AWU59_145 [Methanolobus sp. T82-4]
SDF34753.1	SAMN04488589_0422	341	archaea>euryarchaeota	Methanolobus vulcani	eRF1 domain-containing protein 2 [Methanolobus vulcani]
WP_013194275.1	-	341	archaea>euryarchaeota	Methanohalobium evestigatum	hypothetical protein [Methanohalobium evestigatum]
WP_023845919.1	-	341	archaea>euryarchaeota	Methanolobus tindarius	hypothetical protein [Methanolobus tindarius]
ODS36816.1	BEH94_05555	340	archaea>euryarchaeota	Candidatus Altiarchaeales archaeon WOR_SM1_SCG	hypothetical protein BEH94_05555 [Candidatus Altiarchaeales archaeon WOR_SM1_SCG]
ABE52111.1	Mbur_1188	339	archaea>euryarchaeota	Methanococcoides burtonii DSM 6242	Translation termination factor aRF1 family protein [Methanococcoides burtonii DSM 6242]
SES67635.1	SAMN04488587_0513	337	archaea>euryarchaeota	Methanococcoides vulcani	eRF1 domain-containing protein 2 [Methanococcoides vulcani]
WP_048195112.1	-	337	archaea>euryarchaeota	Methanococcoides methylutens	hypothetical protein [Methanococcoides methylutens]
WP_048205769.1	-	337	archaea>euryarchaeota	Methanococcoides methylutens	hypothetical protein [Methanococcoides methylutens]
OFV66668.1	SBU_000635	330	archaea>euryarchaeota	Candidatus Syntrophoarchaeum butanivorans	eRF1 domain protein 2 domain protein [Candidatus Syntrophoarchaeum butanivorans]
OFV68690.1	SCAL_000366	330	archaea>euryarchaeota	Candidatus Syntrophoarchaeum caldarius	eRF1 domain protein 2 domain protein [Candidatus Syntrophoarchaeum caldarius]
WP_004077320.1	-	327	archaea>euryarchaeota	Methanoplanus limicola	eRF1 domain 2 protein [Methanoplanus limicola]
WP_042706076.1	-	326	archaea>euryarchaeota	Methanomicrobium mobile	hypothetical protein [Methanomicrobium mobile]
WP_013720604.1	-	325	archaea>euryarchaeota	Methanosaeta concilii	hypothetical protein [Methanosaeta concilii]
CBH39825.1	prf1	324	archaea	uncultured archaeon	conserved hypothetical protein [uncultured archaeon]
WP_013330568.1	-	320	archaea>euryarchaeota	Methanolacinia petrolearia	hypothetical protein [Methanolacinia petrolearia]
WP_048149383.1	-	320	archaea>euryarchaeota	Methanolacinia paynteri	hypothetical protein [Methanolacinia paynteri]
KPQ44120.1	MPEBLZ_01316	315	archaea>euryarchaeota	Candidatus Methanoperedens sp. BLZ1	peptide chain release factor 1 [Candidatus Methanoperedens sp. BLZ1]
KUK43934.1	XD72_1701	314	archaea>euryarchaeota	Methanosaeta harundinacea	Uncharacterized protein XD72_1701 [Methanosaeta harundinacea]
WP_048092553.1	-	313	archaea>euryarchaeota	Candidatus Methanoperedens nitroreducens	hypothetical protein [Candidatus Methanoperedens nitroreducens]
WP_014586549.1	-	312	archaea>euryarchaeota	Methanosaeta harundinacea	hypothetical protein [Methanosaeta harundinacea]
KXB07168.1	AKJ51_01880	310	archaea>euryarchaeota	candidate divison MSBL1 archaeon SCGC-AAA382A20	hypothetical protein AKJ51_01880 [candidate divison MSBL1 archaeon SCGC-AAA382A20]
ABK13951.1	Mthe_0151	305	archaea>euryarchaeota	Methanosaeta thermophila PT	hypothetical protein Mthe_0151 [Methanosaeta thermophila PT]
AAM31982.1	MM_2286	298	archaea>euryarchaeota	Methanosarcina mazei Go1	conserved protein [Methanosarcina mazei Go1]
OEU42068.1	BGV40_11570	298	archaea>euryarchaeota	Methanosarcina sp. Ant1	peptide chain release factor-like protein [Methanosarcina sp. Ant1]
WP_011308178.1	-	298	archaea>euryarchaeota	Methanosarcina barkeri	hypothetical protein [Methanosarcina barkeri]
WP_048109810.1	-	298	archaea>euryarchaeota	Methanosarcina barkeri	peptide chain release factor-like protein [Methanosarcina barkeri]
WP_048117234.1	-	298	archaea>euryarchaeota	Methanosarcina barkeri	peptide chain release factor-like protein [Methanosarcina barkeri]
WP_048120180.1	-	298	archaea>euryarchaeota	Methanosarcina vacuolata	peptide chain release factor-like protein [Methanosarcina vacuolata]
WP_048155109.1	-	298	archaea>euryarchaeota	Methanosarcina sp. Kolksee	peptide chain release factor-like protein [Methanosarcina sp. Kolksee]
WP_048155583.1	-	298	archaea>euryarchaeota	Methanosarcina	MULTISPECIES: peptide chain release factor-like protein [Methanosarcina]
WP_048168398.1	-	298	archaea>euryarchaeota	Methanosarcina thermophila	peptide chain release factor-like protein [Methanosarcina thermophila]
WP_048176030.1	-	298	archaea>euryarchaeota	Methanosarcina barkeri	peptide chain release factor-like protein [Methanosarcina barkeri]
WP_054299502.1	-	298	archaea>euryarchaeota	Methanosarcina flavescens	peptide chain release factor-like protein [Methanosarcina flavescens]
KXA96505.1	AKJ38_03185	279	archaea>euryarchaeota	candidate divison MSBL1 archaeon SCGC-AAA259I14	hypothetical protein AKJ38_03185, partial [candidate divison MSBL1 archaeon SCGC-AAA259I14]
EGQ42875.1	J07AB43_08640	298	archaea	Candidatus Nanosalina sp. J07AB43	hypothetical protein J07AB43_08640 [Candidatus Nanosalina sp. J07AB43]
EHK00705.1	HRED_00673	295	archaea	Candidatus Haloredivivus sp. G17	hypothetical protein HRED_00673 [Candidatus Haloredivivus sp. G17]
AOV95241.1	AQV86_04975	291	archaea	Nanohaloarchaea archaeon SG9	hypothetical protein AQV86_04975 [Nanohaloarchaea archaeon SG9]
AJW71433.1	NADRNF5_1755	367	archaea>thaumarchaeota	Candidatus Nitrosopumilus adriaticus	Peptide chain release factor 1 [Candidatus Nitrosopumilus adriaticus]
WP_048069696.1	-	352	archaea>thaumarchaeota	Marine Group I	MULTISPECIES: peptide chain release factor 1 [Marine Group I]
WP_048079408.1	-	352	archaea>thaumarchaeota	Marine Group I thaumarchaeote SCGC AAA799-N04	peptide chain release factor 1 [Marine Group I thaumarchaeote SCGC AAA799-N04]
KUO41552.1	AVW06_01035	341	archaea>euryarchaeota	Hadesarchaea archaeon DG-33-1	hypothetical protein AVW06_01035 [Hadesarchaea archaeon DG-33-1]

2. actinobacterial/chloroflexi VLRF1 (acVLRF1) family

##;the acVLRF1 clade is predicted to lack any genunie domain fusions; however, it does
##;contain two conserved N-terminal beta-strands, which we predict form an extension
##;to the core VLRF1 domain and may have originally been derived from the codon
##;recognition domain.

SEQ62162.1	SAMN05444745_108124	302	bacteria>actinobacteria	Arthrobacter sp. OV608	hypothetical protein SAMN05444745_108124 [Arthrobacter sp. OV608]
ABP55192.1	Strop_2749	293	bacteria>actinobacteria	Salinispora tropica CNB-440	hypothetical protein Strop_2749 [Salinispora tropica CNB-440]
PKB78511.1	BZY88_18025	277	bacteria>chloroflexi	SAR202 cluster bacterium Io17-Chloro-G9	hypothetical protein BZY88_18025 [SAR202 cluster bacterium Io17-Chloro-G9]
WP_095566057.1	-	274	bacteria>actinobacteria	Plantactinospora sp. KBS50	hypothetical protein [Plantactinospora sp. KBS50]
KID30337.1	HQ32_02590	265	bacteria>actinobacteria	Prauserella sp. Am3	hypothetical protein HQ32_02590 [Prauserella sp. Am3]
WP_061255601.1	-	261	bacteria>actinobacteria	Microtetraspora glauca	hypothetical protein [Microtetraspora glauca]
WP_079318773.1	-	261	bacteria>actinobacteria	Microbispora sp. GKU 823	hypothetical protein [Microbispora sp. GKU 823]
WP_092882995.1	-	261	bacteria>actinobacteria	Actinopolymorpha cephalotaxi	hypothetical protein [Actinopolymorpha cephalotaxi]
ABZ08905.1	ALOHA_HF4000APKG5H11ctg2g23	259		uncultured marine microorganism HF4000_APKG5H11	hypothetical protein ALOHA_HF4000APKG5H11ctg2g23 [uncultured marine microorganism HF4000_APKG5H11]
WP_084258815.1	-	259	bacteria>actinobacteria	Microtetraspora malaysiensis	hypothetical protein [Microtetraspora malaysiensis]
SDT09679.1	SAMN04489717_5018	257	bacteria>actinobacteria	Actinopolymorpha singaporensis	hypothetical protein SAMN04489717_5018 [Actinopolymorpha singaporensis]
PCI20392.1	COB68_02345	256	bacteria>chloroflexi	SAR202 cluster bacterium	hypothetical protein COB68_02345 [SAR202 cluster bacterium]
WP_030447514.1	-	256	bacteria>actinobacteria	Actinocatenispora sera	hypothetical protein [Actinocatenispora sera]
PKB57035.1	BZY73_05305	255	bacteria>chloroflexi	SAR202 cluster bacterium Casp-Chloro-G3	hypothetical protein BZY73_05305 [SAR202 cluster bacterium Casp-Chloro-G3]
OGO05274.1	A2Y91_01640	254	bacteria>chloroflexi	Chloroflexi bacterium RBG_13_54_8	hypothetical protein A2Y91_01640 [Chloroflexi bacterium RBG_13_54_8]
OGO31417.1	A2Z29_04640	253	bacteria>chloroflexi	Chloroflexi bacterium RBG_16_56_11	hypothetical protein A2Z29_04640 [Chloroflexi bacterium RBG_16_56_11]
SCE65270.1	GA0070216_101164	253	bacteria>actinobacteria	Micromonospora matsumotoense	hypothetical protein GA0070216_101164 [Micromonospora matsumotoense]
BAS09177.1	AHiyo4_25990	252	bacteria>actinobacteria	Arthrobacter sp. Hiyo4	hypothetical protein AHiyo4_25990 [Arthrobacter sp. Hiyo4]
WP_018806012.1	-	252	bacteria>actinobacteria	Salinispora arenicola	hypothetical protein [Salinispora arenicola]
WP_066635324.1	-	252	bacteria>actinobacteria	Serinicoccus sp. JLT9	hypothetical protein [Serinicoccus sp. JLT9]
ONH24273.1	BL253_30670	251	bacteria>actinobacteria	Frankia asymbiotica	hypothetical protein BL253_30670 [Frankia asymbiotica]
PKB69582.1	BZY77_04325	251	bacteria>chloroflexi	SAR202 cluster bacterium Io17-Chloro-G5	hypothetical protein BZY77_04325 [SAR202 cluster bacterium Io17-Chloro-G5]
PKB81562.1	BZY84_05830	251	bacteria>chloroflexi	SAR202 cluster bacterium MP-SInd-SRR3963457-G1	hypothetical protein BZY84_05830 [SAR202 cluster bacterium MP-SInd-SRR3963457-G1]
OGN97668.1	A2Z77_04705	250	bacteria>chloroflexi	Chloroflexi bacterium RBG_13_51_36	hypothetical protein A2Z77_04705 [Chloroflexi bacterium RBG_13_51_36]
OGN98995.1	A2Y89_00195	250	bacteria>chloroflexi	Chloroflexi bacterium RBG_13_51_18	hypothetical protein A2Y89_00195 [Chloroflexi bacterium RBG_13_51_18]
OGN99170.1	A2Y58_02590	250	bacteria>chloroflexi	Chloroflexi bacterium RBG_13_51_52	hypothetical protein A2Y58_02590 [Chloroflexi bacterium RBG_13_51_52]
PIP48955.1	COX14_01875	250	bacteria>chloroflexi	Chloroflexi bacterium CG23_combo_of_CG06-09_8_20_14_all_45_10	hypothetical protein COX14_01875 [Chloroflexi bacterium CG23_combo_of_CG06-09_8_20_14_all_45_10]
PKB60348.1	BZY64_01210	249	bacteria>chloroflexi	SAR202 cluster bacterium Ae2-Chloro-G1	hypothetical protein BZY64_01210 [SAR202 cluster bacterium Ae2-Chloro-G1]
SEC39753.1	SAMN04489727_3608	248	bacteria>actinobacteria	Amycolatopsis tolypomycina	hypothetical protein SAMN04489727_3608 [Amycolatopsis tolypomycina]
WP_051620189.1	-	248	bacteria>actinobacteria	Haematomicrobium sanguinis	hypothetical protein [Haematomicrobium sanguinis]
AJG38147.1	-	247	bacteria	bacterium enrichment culture clone fosmid MGS-K1	hypothetical protein [bacterium enrichment culture clone fosmid MGS-K1]
PKB66527.1	BZY82_05510	247	bacteria>chloroflexi	SAR202 cluster bacterium Io17-Chloro-G3	hypothetical protein BZY82_05510 [SAR202 cluster bacterium Io17-Chloro-G3]
SDT67965.1	SAMN04515669_5799	247	bacteria>actinobacteria	Jiangella sp. DSM 45060	eRF1 domain-containing protein 2 [Jiangella sp. DSM 45060]
SEF15218.1	SAMN04488561_4886	247	bacteria>actinobacteria	Jiangella alba	hypothetical protein SAMN04488561_4886 [Jiangella alba]
WP_028130829.1	-	247	bacteria>actinobacteria	Serinicoccus marinus	hypothetical protein [Serinicoccus marinus]
WP_075814070.1	-	247	bacteria>actinobacteria	Serinicoccus sp. CUA-874	hypothetical protein [Serinicoccus sp. CUA-874]
WP_075816886.1	-	247	bacteria>actinobacteria	Serinicoccus sp. CNJ-927	hypothetical protein [Serinicoccus sp. CNJ-927]
OGO19041.1	A2Z15_06890	246	bacteria>chloroflexi	Chloroflexi bacterium RBG_16_50_11	hypothetical protein A2Z15_06890 [Chloroflexi bacterium RBG_16_50_11]
OGO23917.1	A2Z28_05440	246	bacteria>chloroflexi	Chloroflexi bacterium RBG_16_51_9	hypothetical protein A2Z28_05440 [Chloroflexi bacterium RBG_16_51_9]
WP_093173123.1	-	246	bacteria>actinobacteria	Sinosporangium album	hypothetical protein [Sinosporangium album]
WP_087026022.1	-	245	bacteria>actinobacteria	Arthrobacter agilis	hypothetical protein [Arthrobacter agilis]
OHV47159.1	BCD48_19910	242	bacteria>actinobacteria	Frankia sp. BMG5.36	hypothetical protein BCD48_19910 [Frankia sp. BMG5.36]
SCL61107.1	GA0070604_4443	242	bacteria>actinobacteria	Micromonospora eburnea	hypothetical protein GA0070604_4443 [Micromonospora eburnea]
WP_093944385.1	-	242	bacteria>actinobacteria	Actinoalloteichus hoggarensis	hypothetical protein [Actinoalloteichus hoggarensis]
WP_018801330.1	-	241	bacteria>actinobacteria	Salinispora arenicola	hypothetical protein [Salinispora arenicola]
WP_030530552.1	-	241	bacteria>actinobacteria	Prauserella rugosa	hypothetical protein [Prauserella rugosa]
WP_009075937.1	-	240	bacteria>actinobacteria	Streptomyces sp. AA4	hypothetical protein [Streptomyces sp. AA4]
WP_062437689.1	-	240	bacteria>actinobacteria	Herbidospora daliensis	hypothetical protein [Herbidospora daliensis]
WP_083672971.1	-	240	bacteria>actinobacteria	Micromonospora sp. CB01531	hypothetical protein [Micromonospora sp. CB01531]
OGN90658.1	A2Z70_02340	239	bacteria>chloroflexi	Chloroflexi bacterium RBG_13_48_17	hypothetical protein A2Z70_02340 [Chloroflexi bacterium RBG_13_48_17]
WP_062340352.1	-	239	bacteria>actinobacteria	Herbidospora sakaeratensis	hypothetical protein [Herbidospora sakaeratensis]
WP_075743743.1	-	239	bacteria>actinobacteria	Actinoalloteichus	MULTISPECIES: hypothetical protein [Actinoalloteichus]
SCG81229.1	GA0070623_5603	238	bacteria>actinobacteria	Micromonospora rifamycinica	hypothetical protein GA0070623_5603 [Micromonospora rifamycinica]
WP_051773462.1	-	238	bacteria>actinobacteria	Saccharothrix sp. NRRL B-16314	hypothetical protein [Saccharothrix sp. NRRL B-16314]
WP_081438878.1	-	238	bacteria>actinobacteria	Frankia asymbiotica	hypothetical protein [Frankia asymbiotica]
WP_091195796.1	-	238	bacteria>actinobacteria	Micromonospora narathiwatensis	hypothetical protein [Micromonospora narathiwatensis]
WP_067304434.1	-	237	bacteria>actinobacteria	Micromonospora rifamycinica	hypothetical protein [Micromonospora rifamycinica]
WP_089004379.1	-	237	bacteria>actinobacteria	Micromonospora rifamycinica	hypothetical protein [Micromonospora rifamycinica]
OGO44500.1	A2Z05_00895	236	bacteria>chloroflexi	Chloroflexi bacterium RBG_16_60_22	hypothetical protein A2Z05_00895 [Chloroflexi bacterium RBG_16_60_22]
WP_056340302.1	-	236	bacteria>actinobacteria	Arthrobacter sp. Soil762	hypothetical protein [Arthrobacter sp. Soil762]
WP_091051141.1	-	236	bacteria>actinobacteria	Glycomyces sambucus	hypothetical protein [Glycomyces sambucus]
WP_076441404.1	-	235	bacteria>actinobacteria	Microbispora rosea	hypothetical protein [Microbispora rosea]
WP_091378451.1	-	235	bacteria>actinobacteria	Geodermatophilus sp. DSM 45219	hypothetical protein [Geodermatophilus sp. DSM 45219]
KQO01022.1	ASF21_11745	234	bacteria>actinobacteria	Arthrobacter sp. Leaf234	hypothetical protein ASF21_11745 [Arthrobacter sp. Leaf234]
WP_082566152.1	-	234	bacteria>actinobacteria	Arthrobacter sp. Soil782	hypothetical protein [Arthrobacter sp. Soil782]
KQS63984.1	ASG41_17830	233	bacteria>actinobacteria	Modestobacter sp. Leaf380	hypothetical protein ASG41_17830 [Modestobacter sp. Leaf380]
WP_051899146.1	-	233	bacteria>actinobacteria	Sciscionella sp. SE31	hypothetical protein [Sciscionella sp. SE31]
SDH88239.1	SAMN05421505_12544	232	bacteria>actinobacteria	Sinosporangium album	hypothetical protein SAMN05421505_12544 [Sinosporangium album]
WP_061292524.1	-	232	bacteria>actinobacteria	Herbidospora cretacea	hypothetical protein [Herbidospora cretacea]
WP_066886058.1	-	232	bacteria>actinobacteria	Streptomyces thermoautotrophicus	hypothetical protein [Streptomyces thermoautotrophicus]
WP_069848853.1	-	232	bacteria>actinobacteria	Actinoalloteichus hymeniacidonis	hypothetical protein [Actinoalloteichus hymeniacidonis]
WP_074312756.1	-	232	bacteria>actinobacteria	Micromonospora cremea	hypothetical protein [Micromonospora cremea]
WP_090479704.1	-	232	bacteria>actinobacteria	Nakamurella panacisegetis	hypothetical protein [Nakamurella panacisegetis]
WP_030507273.1	-	231	bacteria>actinobacteria	Microbispora rosea	hypothetical protein [Microbispora rosea]
WP_089006142.1	-	231	bacteria>actinobacteria	Micromonospora viridifaciens	hypothetical protein [Micromonospora viridifaciens]
WP_091221971.1	-	231	bacteria>actinobacteria	Geodermatophilus siccatus	hypothetical protein [Geodermatophilus siccatus]
WP_091447684.1	-	231	bacteria>actinobacteria	Geodermatophilus poikilotrophus	hypothetical protein [Geodermatophilus poikilotrophus]
WP_091543905.1	-	231	bacteria>actinobacteria	Modestobacter sp. DSM 44400	hypothetical protein [Modestobacter sp. DSM 44400]
OGN88355.1	A2158_05295	230	bacteria>chloroflexi	Chloroflexi bacterium RBG_13_46_14	hypothetical protein A2158_05295 [Chloroflexi bacterium RBG_13_46_14]
WP_012183398.1	-	230	bacteria>actinobacteria	Salinispora arenicola	hypothetical protein [Salinispora arenicola]
WP_012950828.1	-	230	bacteria>actinobacteria	Geodermatophilus obscurus	hypothetical protein [Geodermatophilus obscurus]
WP_026174856.1	-	230	bacteria>actinobacteria	Acaricomes phytoseiuli	hypothetical protein [Acaricomes phytoseiuli]
WP_029023800.1	-	230	bacteria>actinobacteria	Salinispora arenicola	hypothetical protein [Salinispora arenicola]
WP_066936971.1	-	230	bacteria>actinobacteria	Microtetraspora fusca	hypothetical protein [Microtetraspora fusca]
WP_089302695.1	-	230	bacteria>actinobacteria	Haloechinothrix alba	hypothetical protein [Haloechinothrix alba]
WP_092658215.1	-	230	bacteria>actinobacteria	Actinopolymorpha singaporensis	hypothetical protein [Actinopolymorpha singaporensis]
WP_013287438.1	-	229	bacteria>actinobacteria	Micromonospora aurantiaca	hypothetical protein [Micromonospora aurantiaca]
WP_013475893.1	-	229	bacteria>actinobacteria	Micromonospora sp. L5	hypothetical protein [Micromonospora sp. L5]
WP_013601104.1	-	229	bacteria>actinobacteria	Pseudarthrobacter phenanthrenivorans	hypothetical protein [Pseudarthrobacter phenanthrenivorans]
WP_036311617.1	-	229	bacteria>actinobacteria	Micromonospora globosa	hypothetical protein [Micromonospora globosa]
WP_036343732.1	-	229	bacteria>actinobacteria	Micromonospora purpureochromogenes	hypothetical protein [Micromonospora purpureochromogenes]
WP_043329647.1	-	229	bacteria>actinobacteria	Micromonospora sp. M42	hypothetical protein [Micromonospora sp. M42]
WP_073252729.1	-	229	bacteria>actinobacteria	Cryptosporangium aurantiacum	hypothetical protein [Cryptosporangium aurantiacum]
WP_075850533.1	-	229	bacteria>actinobacteria	Saccharomonospora sp. CUA-673	hypothetical protein [Saccharomonospora sp. CUA-673]
WP_076803865.1	-	229	bacteria>actinobacteria	Arthrobacter sp. QXT-31	hypothetical protein [Arthrobacter sp. QXT-31]
WP_082171286.1	-	229	bacteria>actinobacteria	Micromonospora sp. RV43	hypothetical protein [Micromonospora sp. RV43]
WP_082930883.1	-	229	bacteria>actinobacteria	Micromonospora sp. NBRC 110037	hypothetical protein [Micromonospora sp. NBRC 110037]
WP_083297059.1	-	229	bacteria>actinobacteria	Micromonospora sp. II	hypothetical protein [Micromonospora sp. II]
WP_083395326.1	-	229	bacteria>actinobacteria	Micromonospora sp. WMMB235	hypothetical protein [Micromonospora sp. WMMB235]
WP_083419512.1	-	229	bacteria>actinobacteria	Frankia sp. BMG5.36	hypothetical protein [Frankia sp. BMG5.36]
WP_083664843.1	-	229	bacteria>actinobacteria	Micromonospora sp. TSRI0369	hypothetical protein [Micromonospora sp. TSRI0369]
WP_087874661.1	-	229	bacteria>actinobacteria	Arthrobacter globiformis	hypothetical protein [Arthrobacter globiformis]
WP_091034328.1	-	229	bacteria>actinobacteria	Glycomyces harbinensis	hypothetical protein [Glycomyces harbinensis]
WP_091331186.1	-	229	bacteria>actinobacteria	Micromonospora aurantiaca	hypothetical protein [Micromonospora aurantiaca]
WP_027344794.1	-	228	bacteria>actinobacteria	Hamadaea tsunoensis	hypothetical protein [Hamadaea tsunoensis]
WP_070349348.1	-	228	bacteria>actinobacteria	Arthrobacter sp. SW1	hypothetical protein [Arthrobacter sp. SW1]
WP_083606438.1	-	228	bacteria>actinobacteria	Geodermatophilus obscurus	hypothetical protein [Geodermatophilus obscurus]
WP_089019874.1	-	228	bacteria>actinobacteria	Micromonospora coriariae	hypothetical protein [Micromonospora coriariae]
WP_089210314.1	-	228	bacteria>actinobacteria	Streptosporangium subroseum	hypothetical protein [Streptosporangium subroseum]
WP_091157962.1	-	228	bacteria>actinobacteria	Geodermatophilus africanus	hypothetical protein [Geodermatophilus africanus]
WP_091238779.1	-	228	bacteria>actinobacteria	Micromonospora matsumotoense	hypothetical protein [Micromonospora matsumotoense]
WP_100500425.1	-	228	bacteria>actinobacteria	Geodermatophilus sp. B12	hypothetical protein [Geodermatophilus sp. B12]
SCG45505.1	GA0070614_1330	227	bacteria>actinobacteria	Micromonospora coxensis	hypothetical protein GA0070614_1330 [Micromonospora coxensis]
WP_010148515.1	-	227	bacteria>actinobacteria	Serinicoccus profundi	hypothetical protein [Serinicoccus profundi]
WP_013017713.1	-	227	bacteria>actinobacteria	Stackebrandtia nassauensis	hypothetical protein [Stackebrandtia nassauensis]
WP_020573198.1	-	227	bacteria>actinobacteria	Actinopolymorpha alba	hypothetical protein [Actinopolymorpha alba]
WP_063711724.1	-	227	bacteria>actinobacteria	Nonomuraea coxensis	hypothetical protein [Nonomuraea coxensis]
WP_077005243.1	-	227	bacteria>actinobacteria	Saccharothrix sp. ALI-22-I	hypothetical protein [Saccharothrix sp. ALI-22-I]
AOY71225.1	ARZXY2_1680	226	bacteria>actinobacteria	Arthrobacter sp. ZXY-2	hypothetical protein ARZXY2_1680 [Arthrobacter sp. ZXY-2]
KUR63830.1	JM67_13980	226	bacteria>actinobacteria	Arthrobacter sp. ATCC 21022	hypothetical protein JM67_13980 [Arthrobacter sp. ATCC 21022]
OJV30414.1	BGO26_08340	226	bacteria>actinobacteria	Actinobacteria bacterium 69-20	hypothetical protein BGO26_08340 [Actinobacteria bacterium 69-20]
SCG64366.1	GA0070613_3834	226	bacteria>actinobacteria	Micromonospora inositola	hypothetical protein GA0070613_3834 [Micromonospora inositola]
SCL71468.1	GA0070603_5941	226	bacteria>actinobacteria	Micromonospora chersina	hypothetical protein GA0070603_5941 [Micromonospora chersina]
SDP90183.1	SAMN04487905_112107	226	bacteria>actinobacteria	Actinopolyspora xinjiangensis	hypothetical protein SAMN04487905_112107 [Actinopolyspora xinjiangensis]
SFB85626.1	SAMN05216284_101373	226	bacteria>actinobacteria	Verrucosispora sediminis	hypothetical protein SAMN05216284_101373 [Verrucosispora sediminis]
WP_013421596.1	-	226	bacteria>actinobacteria	Frankia inefficax	hypothetical protein [Frankia inefficax]
WP_043574778.1	-	226	bacteria>actinobacteria	Actinopolyspora erythraea	hypothetical protein [Actinopolyspora erythraea]
WP_045073968.1	-	226	bacteria>actinobacteria	Arthrobacter sp. IHBB 11108	hypothetical protein [Arthrobacter sp. IHBB 11108]
WP_051770596.1	-	226	bacteria>actinobacteria	Lentzea albidocapillata	hypothetical protein [Lentzea albidocapillata]
WP_051784704.1	-	226	bacteria>actinobacteria	Lechevalieria aerocolonigenes	hypothetical protein [Lechevalieria aerocolonigenes]
WP_053719185.1	-	226	bacteria>actinobacteria	Saccharothrix sp. NRRL B-16348	hypothetical protein [Saccharothrix sp. NRRL B-16348]
WP_073900197.1	-	226	bacteria>actinobacteria	Saccharothrix sp. CB00851	hypothetical protein [Saccharothrix sp. CB00851]
WP_086668771.1	-	226	bacteria>actinobacteria	Lentzea kentuckyensis	hypothetical protein [Lentzea kentuckyensis]
WP_089306889.1	-	226	bacteria>actinobacteria	Geodermatophilus pulveris	hypothetical protein [Geodermatophilus pulveris]
WP_089916820.1	-	226	bacteria>actinobacteria	Lentzea albida	hypothetical protein [Lentzea albida]
WP_089955241.1	-	226	bacteria>actinobacteria	Lechevalieria xinjiangensis	hypothetical protein [Lechevalieria xinjiangensis]
WP_090011028.1	-	226	bacteria>actinobacteria	Lentzea violacea	hypothetical protein [Lentzea violacea]
WP_090056174.1	-	226	bacteria>actinobacteria	Lechevalieria fradiae	hypothetical protein [Lechevalieria fradiae]
WP_090065286.1	-	226	bacteria>actinobacteria	Lentzea flaviverrucosa	hypothetical protein [Lentzea flaviverrucosa]
WP_090105491.1	-	226	bacteria>actinobacteria	Lentzea jiangxiensis	hypothetical protein [Lentzea jiangxiensis]
WP_091808176.1	-	226	bacteria>actinobacteria	Prauserella marina	hypothetical protein [Prauserella marina]
WP_093604439.1	-	226	bacteria>actinobacteria	Lentzea waywayandensis	hypothetical protein [Lentzea waywayandensis]
WP_097187529.1	-	226	bacteria>actinobacteria	Ornithinimicrobium pekingense	hypothetical protein [Ornithinimicrobium pekingense]
KRF04584.1	ASH00_13300	225	bacteria>actinobacteria	Arthrobacter sp. Soil782	hypothetical protein ASH00_13300 [Arthrobacter sp. Soil782]
OKI52056.1	A6A27_33095	225	bacteria>actinobacteria	Micromonospora sp. CB01531	hypothetical protein A6A27_33095 [Micromonospora sp. CB01531]
SFQ49730.1	SAMN05421810_10896	225	bacteria>actinobacteria	Yuhushiella deserti	hypothetical protein SAMN05421810_10896 [Yuhushiella deserti]
WP_017975889.1	-	225	bacteria>actinobacteria	Actinopolyspora halophila	hypothetical protein [Actinopolyspora halophila]
WP_024477094.1	-	225	bacteria>actinobacteria	Arthrobacter sp. CAL618	hypothetical protein [Arthrobacter sp. CAL618]
WP_025357217.1	-	225	bacteria>actinobacteria	Kutzneria albida	hypothetical protein [Kutzneria albida]
WP_026448926.1	-	225	bacteria>actinobacteria	Actinopolyspora mortivallis	hypothetical protein [Actinopolyspora mortivallis]
WP_028267149.1	-	225	bacteria>actinobacteria	Arthrobacter sp. MA-N2	hypothetical protein [Arthrobacter sp. MA-N2]
WP_028849307.1	-	225	bacteria>actinobacteria	Thermocrispum municipale	hypothetical protein [Thermocrispum municipale]
WP_037713387.1	-	225	bacteria>actinobacteria	Amycolatopsis sp. AA4	hypothetical protein [Amycolatopsis sp. AA4]
WP_037814711.1	-	225	bacteria>actinobacteria	Streptomyces sp. NRRL F-3213	hypothetical protein [Streptomyces sp. NRRL F-3213]
WP_043833262.1	-	225	bacteria>actinobacteria	Amycolatopsis orientalis	hypothetical protein [Amycolatopsis orientalis]
WP_045317395.1	-	225	bacteria>actinobacteria	Lechevalieria aerocolonigenes	hypothetical protein [Lechevalieria aerocolonigenes]
WP_053736514.1	-	225	bacteria>actinobacteria	Nocardia sp. NRRL S-836	hypothetical protein [Nocardia sp. NRRL S-836]
WP_065917634.1	-	225	bacteria>actinobacteria	Lentzea guizhouensis	hypothetical protein [Lentzea guizhouensis]
WP_074702239.1	-	225	bacteria>actinobacteria	Arthrobacter crystallopoietes	hypothetical protein [Arthrobacter crystallopoietes]
WP_078108981.1	-	225	bacteria>actinobacteria	Arthrobacter sp. SRS-W-1-2016	hypothetical protein [Arthrobacter sp. SRS-W-1-2016]
WP_087075830.1	-	225	bacteria>actinobacteria	Arthrobacter agilis	hypothetical protein [Arthrobacter agilis]
WP_090584762.1	-	225	bacteria>actinobacteria	Arthrobacter subterraneus	hypothetical protein [Arthrobacter subterraneus]
WP_091074606.1	-	225	bacteria>actinobacteria	Friedmanniella sagamiharensis	hypothetical protein [Friedmanniella sagamiharensis]
WP_091419852.1	-	225	bacteria>actinobacteria	Arthrobacter sp. OV608	hypothetical protein [Arthrobacter sp. OV608]
WP_091672912.1	-	225	bacteria>actinobacteria	Amycolatopsis marina	hypothetical protein [Amycolatopsis marina]
WP_092520583.1	-	225	bacteria>actinobacteria	Actinopolyspora saharensis	hypothetical protein [Actinopolyspora saharensis]
WP_092627707.1	-	225	bacteria>actinobacteria	Actinopolyspora mzabensis	hypothetical protein [Actinopolyspora mzabensis]
WP_092927888.1	-	225	bacteria>actinobacteria	Actinopolyspora alba	hypothetical protein [Actinopolyspora alba]
WP_092973452.1	-	225	bacteria>actinobacteria	Actinopolyspora righensis	hypothetical protein [Actinopolyspora righensis]
CCI53325.1	BN13_360011	224	bacteria>actinobacteria	Tetrasphaera jenkinsii Ben 74	conserved hypothetical protein [Tetrasphaera jenkinsii Ben 74]
KHF42810.1	MINT15_30120	224	bacteria>actinobacteria	Saccharomonospora viridis	hypothetical protein MINT15_30120 [Saccharomonospora viridis]
KQS58668.1	ASG36_11610	224	bacteria>actinobacteria	Geodermatophilus sp. Leaf369	hypothetical protein ASG36_11610 [Geodermatophilus sp. Leaf369]
SCL37017.1	GA0070624_5729	224	bacteria>actinobacteria	Micromonospora rhizosphaerae	hypothetical protein GA0070624_5729 [Micromonospora rhizosphaerae]
WP_003800721.1	-	224	bacteria>actinobacteria	Arthrobacter globiformis	hypothetical protein [Arthrobacter globiformis]
WP_011873593.1	-	224	bacteria>actinobacteria	Saccharopolyspora erythraea	hypothetical protein [Saccharopolyspora erythraea]
WP_015937212.1	-	224	bacteria>actinobacteria	Pseudarthrobacter chlorophenolicus	hypothetical protein [Pseudarthrobacter chlorophenolicus]
WP_029434077.1	-	224	bacteria>actinobacteria	Blastococcus sp. URHD0036	hypothetical protein [Blastococcus sp. URHD0036]
WP_045732538.1	-	224	bacteria>actinobacteria	Pseudarthrobacter chlorophenolicus	hypothetical protein [Pseudarthrobacter chlorophenolicus]
WP_049830054.1	-	224	bacteria>actinobacteria	Arthrobacter sp. RIT-PI-e	hypothetical protein [Arthrobacter sp. RIT-PI-e]
WP_050054929.1	-	224	bacteria>actinobacteria	Pseudarthrobacter siccitolerans	hypothetical protein [Pseudarthrobacter siccitolerans]
WP_056079443.1	-	224	bacteria>actinobacteria	Arthrobacter sp. Leaf137	hypothetical protein [Arthrobacter sp. Leaf137]
WP_058932350.1	-	224	bacteria>actinobacteria	Pseudarthrobacter sulfonivorans	hypothetical protein [Pseudarthrobacter sulfonivorans]
WP_066291670.1	-	224	bacteria>actinobacteria	Arthrobacter sp. B6	hypothetical protein [Arthrobacter sp. B6]
WP_073420970.1	-	224	bacteria>actinobacteria	Geodermatophilus nigrescens	hypothetical protein [Geodermatophilus nigrescens]
WP_078027882.1	-	224	bacteria>actinobacteria	Arthrobacter sp. KBS0703	hypothetical protein [Arthrobacter sp. KBS0703]
WP_079596217.1	-	224	bacteria>actinobacteria	Arthrobacter sp. P2b	hypothetical protein [Arthrobacter sp. P2b]
WP_089335622.1	-	224	bacteria>actinobacteria	Blastococcus sp. DSM 44272	hypothetical protein [Blastococcus sp. DSM 44272]
WP_091720468.1	-	224	bacteria>actinobacteria	Pseudarthrobacter equi	hypothetical protein [Pseudarthrobacter equi]
WP_092865094.1	-	224	bacteria>actinobacteria	Quadrisphaera sp. DSM 44207	hypothetical protein [Quadrisphaera sp. DSM 44207]
WP_093417167.1	-	224	bacteria>actinobacteria	Saccharopolyspora flava	hypothetical protein [Saccharopolyspora flava]
OUM39515.1	B8W73_13600	223	bacteria>actinobacteria	Arthrobacter agilis	hypothetical protein B8W73_13600 [Arthrobacter agilis]
WP_009154866.1	-	223	bacteria>actinobacteria	Saccharomonospora marina	hypothetical protein [Saccharomonospora marina]
WP_016699278.1	-	223	bacteria>actinobacteria	Actinoalloteichus spitiensis	hypothetical protein [Actinoalloteichus spitiensis]
WP_020672753.1	-	223	bacteria>actinobacteria	Amycolatopsis nigrescens	hypothetical protein [Amycolatopsis nigrescens]
WP_026418820.1	-	223	bacteria>actinobacteria	Actinoalloteichus cyanogriseus	hypothetical protein [Actinoalloteichus cyanogriseus]
WP_026425086.1	-	223	bacteria>actinobacteria	Actinokineospora inagensis	hypothetical protein [Actinokineospora inagensis]
WP_028846110.1	-	223	bacteria>actinobacteria	Thermocrispum agreste	hypothetical protein [Thermocrispum agreste]
WP_030226786.1	-	223	bacteria>actinobacteria	Actinoalloteichus cyanogriseus	hypothetical protein [Actinoalloteichus cyanogriseus]
WP_030429296.1	-	223	bacteria>actinobacteria	Allokutzneria albata	hypothetical protein [Allokutzneria albata]
WP_037321057.1	-	223	bacteria>actinobacteria	Amycolatopsis orientalis	hypothetical protein [Amycolatopsis orientalis]
WP_044850323.1	-	223	bacteria>actinobacteria	Amycolatopsis orientalis	hypothetical protein [Amycolatopsis orientalis]
WP_056635198.1	-	223	bacteria>actinobacteria	Arthrobacter sp. Soil736	hypothetical protein [Arthrobacter sp. Soil736]
WP_056864355.1	-	223	bacteria>actinobacteria	Nocardioides sp. Leaf285	hypothetical protein [Nocardioides sp. Leaf285]
WP_056904001.1	-	223	bacteria>actinobacteria	Nocardioides sp. Leaf307	hypothetical protein [Nocardioides sp. Leaf307]
WP_073480962.1	-	223	bacteria>actinobacteria	Streptoalloteichus hindustanus	hypothetical protein [Streptoalloteichus hindustanus]
WP_075014119.1	-	223	bacteria>actinobacteria	Geodermatophilus obscurus	hypothetical protein [Geodermatophilus obscurus]
WP_086819846.1	-	223	bacteria>actinobacteria	Allokutzneria sp. NRRL B-24872	hypothetical protein [Allokutzneria sp. NRRL B-24872]
WP_088973875.1	-	223	bacteria>actinobacteria	Micromonospora siamensis	hypothetical protein [Micromonospora siamensis]
WP_089402761.1	-	223	bacteria>actinobacteria	Geodermatophilus saharensis	hypothetical protein [Geodermatophilus saharensis]
WP_091383888.1	-	223	bacteria>actinobacteria	Alloactinosynnema album	hypothetical protein [Alloactinosynnema album]
WP_092546769.1	-	223	bacteria>actinobacteria	Actinoplanes derwentensis	hypothetical protein [Actinoplanes derwentensis]
WP_092780253.1	-	223	bacteria>actinobacteria	Actinokineospora terrae	hypothetical protein [Actinokineospora terrae]
WP_097205272.1	-	223	bacteria>actinobacteria	Geodermatophilus sabuli	hypothetical protein [Geodermatophilus sabuli]
WP_005437627.1	-	222	bacteria>actinobacteria	Saccharomonospora azurea	hypothetical protein [Saccharomonospora azurea]
WP_005455791.1	-	222	bacteria>actinobacteria	Saccharomonospora cyanea	hypothetical protein [Saccharomonospora cyanea]
WP_005463781.1	-	222	bacteria>actinobacteria	Saccharomonospora glauca	hypothetical protein [Saccharomonospora glauca]
WP_006236592.1	-	222	bacteria>actinobacteria	Saccharomonospora xinjiangensis	hypothetical protein [Saccharomonospora xinjiangensis]
WP_015747554.1	-	222	bacteria>actinobacteria	Nakamurella multipartita	hypothetical protein [Nakamurella multipartita]
WP_015785977.1	-	222	bacteria>actinobacteria	Saccharomonospora viridis	hypothetical protein [Saccharomonospora viridis]
WP_016333191.1	-	222	bacteria>actinobacteria	Amycolatopsis orientalis	hypothetical protein [Amycolatopsis orientalis]
WP_018722895.1	-	222	bacteria>actinobacteria	Salinispora pacifica	hypothetical protein [Salinispora pacifica]
WP_018761440.1	-	222	bacteria>actinobacteria	Arthrobacter sp. 135MFCol5.1	hypothetical protein [Arthrobacter sp. 135MFCol5.1]
WP_018769325.1	-	222	bacteria>actinobacteria	Arthrobacter sp. 162MFSha1.1	hypothetical protein [Arthrobacter sp. 162MFSha1.1]
WP_020637140.1	-	222	bacteria>actinobacteria	Amycolatopsis alba	hypothetical protein [Amycolatopsis alba]
WP_024874224.1	-	222	bacteria>actinobacteria	Saccharomonospora sp. CNQ490	hypothetical protein [Saccharomonospora sp. CNQ490]
WP_026535624.1	-	222	bacteria>actinobacteria	Arthrobacter sp. H14	hypothetical protein [Arthrobacter sp. H14]
WP_029721878.1	-	222	bacteria>actinobacteria	Saccharopolyspora rectivirgula	hypothetical protein [Saccharopolyspora rectivirgula]
WP_030452284.1	-	222	bacteria>actinobacteria	Herbidospora cretacea	hypothetical protein [Herbidospora cretacea]
WP_034311832.1	-	222	bacteria>actinobacteria	Amycolatopsis lurida	hypothetical protein [Amycolatopsis lurida]
WP_036335840.1	-	222	bacteria>actinobacteria	Modestobacter caceresii	hypothetical protein [Modestobacter caceresii]
WP_037344070.1	-	222	bacteria>actinobacteria	Amycolatopsis sp. MJM2582	hypothetical protein [Amycolatopsis sp. MJM2582]
WP_038515463.1	-	222	bacteria>actinobacteria	Amycolatopsis	MULTISPECIES: hypothetical protein [Amycolatopsis]
WP_039915822.1	-	222	bacteria>actinobacteria	Amycolatopsis azurea	hypothetical protein [Amycolatopsis azurea]
WP_040338747.1	-	222	bacteria>actinobacteria	Candidatus Blastococcus massiliensis	hypothetical protein [Candidatus Blastococcus massiliensis]
WP_043454436.1	-	222	bacteria>actinobacteria	Pseudarthrobacter phenanthrenivorans	hypothetical protein [Pseudarthrobacter phenanthrenivorans]
WP_051005091.1	-	222	bacteria>actinobacteria	Blastococcus saxobsidens	hypothetical protein [Blastococcus saxobsidens]
WP_054046458.1	-	222	bacteria>actinobacteria	Alloactinosynnema sp. L-07	hypothetical protein [Alloactinosynnema sp. L-07]
WP_056336609.1	-	222	bacteria>actinobacteria	Arthrobacter sp. Soil764	hypothetical protein [Arthrobacter sp. Soil764]
WP_056390471.1	-	222	bacteria>actinobacteria	Arthrobacter sp. Soil761	hypothetical protein [Arthrobacter sp. Soil761]
WP_061980725.1	-	222	bacteria>actinobacteria	Amycolatopsis regifaucium	hypothetical protein [Amycolatopsis regifaucium]
WP_062348734.1	-	222	bacteria>actinobacteria	Herbidospora yilanensis	hypothetical protein [Herbidospora yilanensis]
WP_063276038.1	-	222	bacteria>actinobacteria	Amycolatopsis keratiniphila	hypothetical protein [Amycolatopsis keratiniphila]
WP_066276233.1	-	222	bacteria>actinobacteria	Arthrobacter sp. OY3WO11	hypothetical protein [Arthrobacter sp. OY3WO11]
WP_066368161.1	-	222	bacteria>actinobacteria	Herbidospora mongoliensis	hypothetical protein [Herbidospora mongoliensis]
WP_072027805.1	-	222	bacteria>actinobacteria	Amycolatopsis keratiniphila	hypothetical protein [Amycolatopsis keratiniphila]
WP_076165708.1	-	222	bacteria>actinobacteria	Amycolatopsis coloradensis	hypothetical protein [Amycolatopsis coloradensis]
WP_081190665.1	-	222	bacteria>actinobacteria	Saccharomonospora sp. LRS4.154	hypothetical protein [Saccharomonospora sp. LRS4.154]
WP_082557224.1	-	222	bacteria>actinobacteria	Modestobacter sp. Leaf380	hypothetical protein [Modestobacter sp. Leaf380]
WP_091363955.1	-	222	bacteria>actinobacteria	Geodermatophilus telluris	hypothetical protein [Geodermatophilus telluris]
WP_091616037.1	-	222	bacteria>actinobacteria	Micromonospora mirobrigensis	hypothetical protein [Micromonospora mirobrigensis]
WP_091618934.1	-	222	bacteria>actinobacteria	Amycolatopsis saalfeldensis	hypothetical protein [Amycolatopsis saalfeldensis]
WP_091768932.1	-	222	bacteria>actinobacteria	Blastococcus sp. DSM 44268	hypothetical protein [Blastococcus sp. DSM 44268]
WP_091982515.1	-	222	bacteria>actinobacteria	Blastococcus sp. DSM 44205	hypothetical protein [Blastococcus sp. DSM 44205]
WP_092199613.1	-	222	bacteria>actinobacteria	Blastococcus sp. DSM 46838	hypothetical protein [Blastococcus sp. DSM 46838]
WP_092533796.1	-	222	bacteria>actinobacteria	Yuhushiella deserti	hypothetical protein [Yuhushiella deserti]
WP_092603940.1	-	222	bacteria>actinobacteria	Actinopolyspora xinjiangensis	hypothetical protein [Actinopolyspora xinjiangensis]
WP_093579219.1	-	222	bacteria>actinobacteria	Geodermatophilus amargosae	hypothetical protein [Geodermatophilus amargosae]
WP_094863440.1	-	222	bacteria>actinobacteria	Amycolatopsis sp. AU-G6	hypothetical protein [Amycolatopsis sp. AU-G6]
WP_094904724.1	-	222	bacteria>actinobacteria	Actinopolyspora erythraea	hypothetical protein [Actinopolyspora erythraea]
WP_097182690.1	-	222	bacteria>actinobacteria	Blastococcus sp. DSM 44270	hypothetical protein [Blastococcus sp. DSM 44270]
WP_097196592.1	-	222	bacteria>actinobacteria	Blastococcus aggregatus	hypothetical protein [Blastococcus aggregatus]
AEB46276.1	VAB18032_25890	221	bacteria>actinobacteria	Verrucosispora maris AB-18-032	hypothetical protein VAB18032_25890 [Verrucosispora maris AB-18-032]
SFE20563.1	SAMN04487819_109104	221	bacteria>actinobacteria	Actinopolyspora alba	hypothetical protein SAMN04487819_109104 [Actinopolyspora alba]
SFT38117.1	SAMN04487904_101642	221	bacteria>actinobacteria	Actinopolyspora righensis	eRF1 domain-containing protein 2 [Actinopolyspora righensis]
WP_018680685.1	-	221	bacteria>actinobacteria	Actinokineospora enzanensis	hypothetical protein [Actinokineospora enzanensis]
WP_019482830.1	-	221	bacteria>actinobacteria	Arthrobacter sp. TB 23	hypothetical protein [Arthrobacter sp. TB 23]
WP_025274799.1	-	221	bacteria>actinobacteria	Haloglycomyces albus	hypothetical protein [Haloglycomyces albus]
WP_026856753.1	-	221	bacteria>actinobacteria	Geodermatophilaceae bacterium URHB0062	hypothetical protein [Geodermatophilaceae bacterium URHB0062]
WP_029338505.1	-	221	bacteria>actinobacteria	Geodermatophilaceae bacterium URHB0048	hypothetical protein [Geodermatophilaceae bacterium URHB0048]
WP_030161525.1	-	221	bacteria>actinobacteria	Glycomyces sp. NRRL B-16210	hypothetical protein [Glycomyces sp. NRRL B-16210]
WP_034270892.1	-	221	bacteria>actinobacteria	Amycolatopsis halophila	hypothetical protein [Amycolatopsis halophila]
WP_045876612.1	-	221	bacteria>actinobacteria	Frankia sp. DC12	hypothetical protein [Frankia sp. DC12]
WP_051478452.1	-	221	bacteria>actinobacteria	Arthrobacter sp. H5	hypothetical protein [Arthrobacter sp. H5]
WP_056675073.1	-	221	bacteria>actinobacteria	Angustibacter sp. Root456	hypothetical protein [Angustibacter sp. Root456]
WP_058267426.1	-	221	bacteria>actinobacteria	Arthrobacter enclensis	hypothetical protein [Arthrobacter enclensis]
WP_075959034.1	-	221	bacteria>actinobacteria	Ornithinimicrobium sp. CNJ-824	hypothetical protein [Ornithinimicrobium sp. CNJ-824]
WP_083933130.1	-	221	bacteria>actinobacteria	Kribbella catacumbae	hypothetical protein [Kribbella catacumbae]
WP_086780895.1	-	221	bacteria>actinobacteria	Crossiella equi	hypothetical protein [Crossiella equi]
WP_088645088.1	-	221	bacteria>actinobacteria	Micromonospora wenchangensis	hypothetical protein [Micromonospora wenchangensis]
WP_091087348.1	-	221	bacteria>actinobacteria	Nonomuraea wenchangensis	hypothetical protein [Nonomuraea wenchangensis]
WP_091117110.1	-	221	bacteria>actinobacteria	Geodermatophilus sp. DSM 44208	hypothetical protein [Geodermatophilus sp. DSM 44208]
WP_091127321.1	-	221	bacteria>actinobacteria	Micromonospora eburnea	hypothetical protein [Micromonospora eburnea]
WP_091292617.1	-	221	bacteria>actinobacteria	Amycolatopsis xylanica	hypothetical protein [Amycolatopsis xylanica]
WP_091554606.1	-	221	bacteria>actinobacteria	Geodermatophilus taihuensis	hypothetical protein [Geodermatophilus taihuensis]
SDN88257.1	SAMN05660199_00922	220	bacteria>actinobacteria	Geodermatophilus soli	hypothetical protein SAMN05660199_00922 [Geodermatophilus soli]
SIN07725.1	SAMN04489832_2832	220	bacteria>actinobacteria	Micromonospora cremea	hypothetical protein SAMN04489832_2832 [Micromonospora cremea]
WP_007462691.1	-	220	bacteria>actinobacteria	Micromonospora lupini	hypothetical protein [Micromonospora lupini]
WP_023360324.1	-	220	bacteria>actinobacteria	Actinoplanes friuliensis	hypothetical protein [Actinoplanes friuliensis]
WP_055500963.1	-	220	bacteria>actinobacteria	Nonomuraea sp. NBRC 110462	hypothetical protein [Nonomuraea sp. NBRC 110462]
WP_076467626.1	-	220	bacteria>actinobacteria	Micromonospora avicenniae	hypothetical protein [Micromonospora avicenniae]
WP_089016044.1	-	220	bacteria>actinobacteria	Micromonospora inositola	hypothetical protein [Micromonospora inositola]
WP_091101920.1	-	220	bacteria>actinobacteria	Nonomuraea pusilla	hypothetical protein [Nonomuraea pusilla]
WP_091320678.1	-	220	bacteria>actinobacteria	Geodermatophilus ruber	hypothetical protein [Geodermatophilus ruber]
WP_091322419.1	-	220	bacteria>actinobacteria	Micromonospora chersina	hypothetical protein [Micromonospora chersina]
WP_091447279.1	-	220	bacteria>actinobacteria	Alloactinosynnema iranicum	hypothetical protein [Alloactinosynnema iranicum]
WP_091930186.1	-	220	bacteria>actinobacteria	Blastococcus sp. DSM 46786	hypothetical protein [Blastococcus sp. DSM 46786]
WP_091944283.1	-	220	bacteria>actinobacteria	Blastococcus endophyticus	hypothetical protein [Blastococcus endophyticus]
WP_093401608.1	-	220	bacteria>actinobacteria	Verrucosispora sediminis	hypothetical protein [Verrucosispora sediminis]
WP_099851070.1	-	220	bacteria>actinobacteria	Verrucosispora sp. CNZ293	hypothetical protein [Verrucosispora sp. CNZ293]
OLB66922.1	AUI10_01390	219	bacteria>actinobacteria	Actinobacteria bacterium 13_2_20CM_2_72_6	hypothetical protein AUI10_01390 [Actinobacteria bacterium 13_2_20CM_2_72_6]
WP_012245389.1	-	219	bacteria>actinobacteria	Renibacterium salmoninarum	hypothetical protein [Renibacterium salmoninarum]
WP_013733148.1	-	219	bacteria>actinobacteria	Verrucosispora maris	hypothetical protein [Verrucosispora maris]
WP_018255456.1	-	219	bacteria>actinobacteria	Salinispora pacifica	hypothetical protein [Salinispora pacifica]
WP_018583035.1	-	219	bacteria>actinobacteria	Salinispora arenicola	hypothetical protein [Salinispora arenicola]
WP_018719899.1	-	219	bacteria>actinobacteria	Salinispora pacifica	hypothetical protein [Salinispora pacifica]
WP_018733423.1	-	219	bacteria>actinobacteria	Salinispora pacifica	hypothetical protein [Salinispora pacifica]
WP_018739133.1	-	219	bacteria>actinobacteria	Salinispora pacifica	hypothetical protein [Salinispora pacifica]
WP_018798020.1	-	219	bacteria>actinobacteria	Salinispora arenicola	hypothetical protein [Salinispora arenicola]
WP_018804488.1	-	219	bacteria>actinobacteria	Salinispora arenicola	hypothetical protein [Salinispora arenicola]
WP_018810972.1	-	219	bacteria>actinobacteria	Salinispora pacifica	hypothetical protein [Salinispora pacifica]
WP_018818478.1	-	219	bacteria>actinobacteria	Salinispora pacifica	hypothetical protein [Salinispora pacifica]
WP_018827943.1	-	219	bacteria>actinobacteria	Salinispora pacifica	hypothetical protein [Salinispora pacifica]
WP_025619509.1	-	219	bacteria>actinobacteria	Salinispora pacifica	hypothetical protein [Salinispora pacifica]
WP_026185862.1	-	219	bacteria>actinobacteria	Salinispora pacifica	hypothetical protein [Salinispora pacifica]
WP_026274833.1	-	219	bacteria>actinobacteria	Salinispora tropica	hypothetical protein [Salinispora tropica]
WP_026275922.1	-	219	bacteria>actinobacteria	Salinispora	MULTISPECIES: hypothetical protein [Salinispora]
WP_027650836.1	-	219	bacteria>actinobacteria	Salinispora pacifica	hypothetical protein [Salinispora pacifica]
WP_027660981.1	-	219	bacteria>actinobacteria	Salinispora pacifica	hypothetical protein [Salinispora pacifica]
WP_027940651.1	-	219	bacteria>actinobacteria	Amycolatopsis taiwanensis	hypothetical protein [Amycolatopsis taiwanensis]
WP_028184501.1	-	219	bacteria>actinobacteria	Salinispora pacifica	hypothetical protein [Salinispora pacifica]
WP_028193497.1	-	219	bacteria>actinobacteria	Salinispora pacifica	hypothetical protein [Salinispora pacifica]
WP_028564073.1	-	219	bacteria>actinobacteria	Salinispora tropica	hypothetical protein [Salinispora tropica]
WP_028568550.1	-	219	bacteria>actinobacteria	Salinispora tropica	hypothetical protein [Salinispora tropica]
WP_029025168.1	-	219	bacteria>actinobacteria	Salinispora arenicola	hypothetical protein [Salinispora arenicola]
WP_029028543.1	-	219	bacteria>actinobacteria	Salinispora arenicola	hypothetical protein [Salinispora arenicola]
WP_029126604.1	-	219	bacteria>actinobacteria	Salinispora tropica	hypothetical protein [Salinispora tropica]
WP_033666126.1	-	219	bacteria>actinobacteria	Salinispora pacifica	hypothetical protein [Salinispora pacifica]
WP_033668803.1	-	219	bacteria>actinobacteria	Salinispora pacifica	hypothetical protein [Salinispora pacifica]
WP_033773615.1	-	219	bacteria>actinobacteria	Salinispora pacifica	hypothetical protein [Salinispora pacifica]
WP_038840442.1	-	219	bacteria>actinobacteria	Salinispora arenicola	hypothetical protein [Salinispora arenicola]
WP_046766948.1	-	219	bacteria>actinobacteria	Jiangella alkaliphila	hypothetical protein [Jiangella alkaliphila]
WP_050684648.1	-	219	bacteria>actinobacteria	Arthrobacter sp. ZBG10	hypothetical protein [Arthrobacter sp. ZBG10]
WP_056598171.1	-	219	bacteria>actinobacteria	Arthrobacter sp. Leaf141	hypothetical protein [Arthrobacter sp. Leaf141]
WP_069951915.1	-	219	bacteria>actinobacteria	Arthrobacter sp. U41	hypothetical protein [Arthrobacter sp. U41]
WP_074590922.1	-	219	bacteria>actinobacteria	Arthrobacter cupressi	hypothetical protein [Arthrobacter cupressi]
WP_080037128.1	-	219	bacteria>actinobacteria	Nonomuraea sp. ATCC 55076	hypothetical protein [Nonomuraea sp. ATCC 55076]
WP_088285585.1	-	219	bacteria>actinobacteria	Kineosporia sp. A_224	hypothetical protein [Kineosporia sp. A_224]
WP_088313666.1	-	219	bacteria>actinobacteria	Kineosporia sp. R_H_3	hypothetical protein [Kineosporia sp. R_H_3]
WP_088961265.1	-	219	bacteria>actinobacteria	Micromonospora purpureochromogenes	hypothetical protein [Micromonospora purpureochromogenes]
WP_091060249.1	-	219	bacteria>actinobacteria	Geodermatophilus brasiliensis	hypothetical protein [Geodermatophilus brasiliensis]
WP_091240738.1	-	219	bacteria>actinobacteria	Geodermatophilus soli	hypothetical protein [Geodermatophilus soli]
WP_091308646.1	-	219	bacteria>actinobacteria	Amycolatopsis tolypomycina	hypothetical protein [Amycolatopsis tolypomycina]
WP_091560377.1	-	219	bacteria>actinobacteria	Arthrobacter sp. ok362	hypothetical protein [Arthrobacter sp. ok362]
WP_091619862.1	-	219	bacteria>actinobacteria	Micromonospora peucetia	hypothetical protein [Micromonospora peucetia]
WP_092383692.1	-	219	bacteria>actinobacteria	Xiangella phaseoli	hypothetical protein [Xiangella phaseoli]
WP_093261757.1	-	219	bacteria>actinobacteria	Thermomonospora chromogena	hypothetical protein [Thermomonospora chromogena]
SBT44241.1	GA0070611_2623	218	bacteria>actinobacteria	Micromonospora auratinigra	hypothetical protein GA0070611_2623 [Micromonospora auratinigra]
SEH00663.1	SAMN05444920_11794	218	bacteria>actinobacteria	Nonomuraea solani	hypothetical protein SAMN05444920_11794 [Nonomuraea solani]
SES17396.1	SAMN04487818_108101	218	bacteria>actinobacteria	Actinokineospora terrae	hypothetical protein SAMN04487818_108101 [Actinokineospora terrae]
SET87532.1	SAMN04488546_4021	218	bacteria>actinobacteria	Geodermatophilus poikilotrophus	hypothetical protein SAMN04488546_4021 [Geodermatophilus poikilotrophus]
WP_007512308.1	-	218	bacteria>actinobacteria	Frankia	MULTISPECIES: hypothetical protein [Frankia]
WP_012892443.1	-	218	bacteria>actinobacteria	Streptosporangium roseum	hypothetical protein [Streptosporangium roseum]
WP_022913828.1	-	218	bacteria>actinobacteria	Frankia sp. Iso899	hypothetical protein [Frankia sp. Iso899]
WP_022921812.1	-	218	bacteria>actinobacteria	Ornithinimicrobium pekingense	hypothetical protein [Ornithinimicrobium pekingense]
WP_026552163.1	-	218	bacteria>actinobacteria	Arthrobacter sp. H20	hypothetical protein [Arthrobacter sp. H20]
WP_026877893.1	-	218	bacteria>actinobacteria	Jiangella gansuensis	hypothetical protein [Jiangella gansuensis]
WP_026923057.1	-	218	bacteria>actinobacteria	Glycomyces arizonensis	hypothetical protein [Glycomyces arizonensis]
WP_026930540.1	-	218	bacteria>actinobacteria	Glycomyces tenuis	hypothetical protein [Glycomyces tenuis]
WP_030904789.1	-	218	bacteria>actinobacteria	Streptosporangium amethystogenes	hypothetical protein [Streptosporangium amethystogenes]
WP_031161667.1	-	218	bacteria>actinobacteria	Streptosporangium roseum	hypothetical protein [Streptosporangium roseum]
WP_053206527.1	-	218	bacteria>actinobacteria	Jiangella muralis	hypothetical protein [Jiangella muralis]
WP_053657029.1	-	218	bacteria>actinobacteria	Micromonospora sp. NRRL B-16802	hypothetical protein [Micromonospora sp. NRRL B-16802]
WP_058891053.1	-	218	bacteria>actinobacteria	Serinicoccus chungangensis	hypothetical protein [Serinicoccus chungangensis]
WP_068899244.1	-	218	bacteria>actinobacteria	Planomonospora sphaerica	hypothetical protein [Planomonospora sphaerica]
WP_068924104.1	-	218	bacteria>actinobacteria	Planobispora rosea	hypothetical protein [Planobispora rosea]
WP_069109604.1	-	218	bacteria>actinobacteria	Jiangella alba	hypothetical protein [Jiangella alba]
WP_076389476.1	-	218	bacteria>actinobacteria	Propionimicrobium sp. Marseille-P3275	hypothetical protein [Propionimicrobium sp. Marseille-P3275]
WP_086575127.1	-	218	bacteria>actinobacteria	Streptosporangium sp. M26	hypothetical protein [Streptosporangium sp. M26]
WP_090765060.1	-	218	bacteria>actinobacteria	Nonomuraea maritima	hypothetical protein [Nonomuraea maritima]
WP_090942808.1	-	218	bacteria>actinobacteria	Nonomuraea jiangxiensis	hypothetical protein [Nonomuraea jiangxiensis]
WP_091076185.1	-	218	bacteria>actinobacteria	Micromonospora nigra	hypothetical protein [Micromonospora nigra]
WP_091349984.1	-	218	bacteria>actinobacteria	Micromonospora rhizosphaerae	hypothetical protein [Micromonospora rhizosphaerae]
WP_091460245.1	-	218	bacteria>actinobacteria	Micromonospora inyonensis	hypothetical protein [Micromonospora inyonensis]
WP_091507409.1	-	218	bacteria>actinobacteria	Amycolatopsis sacchari	hypothetical protein [Amycolatopsis sacchari]
WP_092626428.1	-	218	bacteria>actinobacteria	Jiangella sp. DSM 45060	hypothetical protein [Jiangella sp. DSM 45060]
WP_093887489.1	-	218	bacteria>actinobacteria	Streptosporangium canum	hypothetical protein [Streptosporangium canum]
WP_100444911.1	-	218	bacteria>actinobacteria	Glycomyces sp. TRM 41368	hypothetical protein [Glycomyces sp. TRM 41368]
CEA08228.1	BN1051_01566	217	bacteria>actinobacteria	Arthrobacter saudimassiliensis	peptide chain release factor 1 [Arthrobacter saudimassiliensis]
SBO98158.1	BN4615_P7674	217	bacteria>actinobacteria	Nonomuraea gerenzanensis	hypothetical protein BN4615_P7674 [Nonomuraea gerenzanensis]
SBT47677.1	GA0070621_2964	217	bacteria>actinobacteria	Micromonospora narathiwatensis	hypothetical protein GA0070621_2964 [Micromonospora narathiwatensis]
SCF21175.1	GA0070618_4076	217	bacteria>actinobacteria	Micromonospora echinospora	hypothetical protein GA0070618_4076 [Micromonospora echinospora]
WP_007072026.1	-	217	bacteria>actinobacteria	Micromonospora sp. ATCC 39149	hypothetical protein [Micromonospora sp. ATCC 39149]
WP_030491558.1	-	217	bacteria>actinobacteria	Micromonospora chokoriensis	hypothetical protein [Micromonospora chokoriensis]
WP_036318540.1	-	217	bacteria>actinobacteria	Microbispora sp. ATCC PTA-5024	hypothetical protein [Microbispora sp. ATCC PTA-5024]
WP_043521541.1	-	217	bacteria>actinobacteria	Actinoplanes utahensis	hypothetical protein [Actinoplanes utahensis]
WP_049568045.1	-	217	bacteria>actinobacteria	Nonomuraea sp. SBT364	hypothetical protein [Nonomuraea sp. SBT364]
WP_088951898.1	-	217	bacteria>actinobacteria	Micromonospora zamorensis	hypothetical protein [Micromonospora zamorensis]
WP_088991531.1	-	217	bacteria>actinobacteria	Micromonospora chokoriensis	hypothetical protein [Micromonospora chokoriensis]
WP_088992915.1	-	217	bacteria>actinobacteria	Micromonospora echinaurantiaca	hypothetical protein [Micromonospora echinaurantiaca]
WP_088999381.1	-	217	bacteria>actinobacteria	Micromonospora echinofusca	hypothetical protein [Micromonospora echinofusca]
WP_089253827.1	-	217	bacteria>actinobacteria	Asanoa hainanensis	hypothetical protein [Asanoa hainanensis]
WP_090790889.1	-	217	bacteria>actinobacteria	Asanoa ishikariensis	hypothetical protein [Asanoa ishikariensis]
WP_091105245.1	-	217	bacteria>actinobacteria	Micromonospora citrea	hypothetical protein [Micromonospora citrea]
WP_091557872.1	-	217	bacteria>actinobacteria	Micromonospora pattaloongensis	hypothetical protein [Micromonospora pattaloongensis]

WP_091629977.1	-	217	bacteria>actinobacteria	Micromonospora peucetia	hypothetical protein [Micromonospora peucetia]
WP_092801716.1	-	217	bacteria>actinobacteria	Actinobacteria bacterium DSM 45722	hypothetical protein [Actinobacteria bacterium DSM 45722]
WP_099857783.1	-	217	bacteria>actinobacteria	Micromonospora sp. CNZ299	hypothetical protein [Micromonospora sp. CNZ299]
PKQ23645.1	CVT65_07040	216	bacteria>actinobacteria	Actinobacteria bacterium HGW-Actinobacteria-5	hypothetical protein CVT65_07040 [Actinobacteria bacterium HGW-Actinobacteria-5]
SDY44232.1	SAMN05661080_03482	216	bacteria>actinobacteria	Modestobacter sp. DSM 44400	hypothetical protein SAMN05661080_03482 [Modestobacter sp. DSM 44400]
WP_003071244.1	-	216	bacteria>actinobacteria	Amycolatopsis vancoresmycina	hypothetical protein [Amycolatopsis vancoresmycina]
WP_005152176.1	-	216	bacteria>actinobacteria	Amycolatopsis azurea	hypothetical protein [Amycolatopsis azurea]
WP_007029725.1	-	216	bacteria>actinobacteria	Amycolatopsis decaplanina	hypothetical protein [Amycolatopsis decaplanina]
WP_013132207.1	-	216	bacteria>actinobacteria	Thermobispora bispora	hypothetical protein [Thermobispora bispora]
WP_035856836.1	-	216	bacteria>actinobacteria	Cryptosporangium arvum	hypothetical protein [Cryptosporangium arvum]
WP_052358887.1	-	216	bacteria>actinobacteria	Corynebacterium variabile	hypothetical protein [Corynebacterium variabile]
WP_072477998.1	-	216	bacteria>actinobacteria	Amycolatopsis australiensis	hypothetical protein [Amycolatopsis australiensis]
WP_083433613.1	-	216	bacteria>actinobacteria	Tetrasphaera australiensis	hypothetical protein [Tetrasphaera australiensis]
WP_084958253.1	-	216	bacteria>actinobacteria	Thermoactinospora rubra	hypothetical protein [Thermoactinospora rubra]
WP_086672476.1	-	216	bacteria>actinobacteria	Amycolatopsis pretoriensis	hypothetical protein [Amycolatopsis pretoriensis]
WP_086858416.1	-	216	bacteria>actinobacteria	Amycolatopsis lexingtonensis	hypothetical protein [Amycolatopsis lexingtonensis]
WP_091597106.1	-	216	bacteria>actinobacteria	Micromonospora krabiensis	hypothetical protein [Micromonospora krabiensis]
WP_093934132.1	-	216	bacteria>actinobacteria	Amycolatopsis thailandensis	hypothetical protein [Amycolatopsis thailandensis]
WP_093976544.1	-	216	bacteria>actinobacteria	Amycolatopsis alba	hypothetical protein [Amycolatopsis alba]
SDY44420.1	SAMN05660209_02863	215	bacteria>actinobacteria	Geodermatophilus africanus	hypothetical protein SAMN05660209_02863 [Geodermatophilus africanus]
WP_011692128.1	-	215	bacteria>actinobacteria	Arthrobacter sp. FB24	hypothetical protein [Arthrobacter sp. FB24]
WP_013224727.1	-	215	bacteria>actinobacteria	Amycolatopsis mediterranei	hypothetical protein [Amycolatopsis mediterranei]
WP_020645323.1	-	215	bacteria>actinobacteria	Amycolatopsis balhimycina	hypothetical protein [Amycolatopsis balhimycina]
WP_028269105.1	-	215	bacteria>actinobacteria	Arthrobacter sp. UNC362MFTsu5.1	hypothetical protein [Arthrobacter sp. UNC362MFTsu5.1]
WP_030337310.1	-	215	bacteria>actinobacteria	Micromonospora parva	hypothetical protein [Micromonospora parva]
WP_043481838.1	-	215	bacteria>actinobacteria	Arthrobacter sp. SPG23	hypothetical protein [Arthrobacter sp. SPG23]
WP_043788222.1	-	215	bacteria>actinobacteria	Amycolatopsis rifamycinica	hypothetical protein [Amycolatopsis rifamycinica]
WP_050814775.1	-	215	bacteria>actinobacteria	Verrucosispora maris	hypothetical protein [Verrucosispora maris]
WP_055482137.1	-	215	bacteria>actinobacteria	Microbispora sp. GMKU363	hypothetical protein [Microbispora sp. GMKU363]
WP_056429614.1	-	215	bacteria>actinobacteria	Arthrobacter sp. Leaf69	hypothetical protein [Arthrobacter sp. Leaf69]
WP_056739157.1	-	215	bacteria>actinobacteria	Arthrobacter sp. Soil763	hypothetical protein [Arthrobacter sp. Soil763]
WP_057323758.1	-	215	bacteria>actinobacteria	Nocardioides sp. Soil797	hypothetical protein [Nocardioides sp. Soil797]
WP_066437670.1	-	215	bacteria>actinobacteria	Arthrobacter sp. EPSL27	hypothetical protein [Arthrobacter sp. EPSL27]
WP_067365643.1	-	215	bacteria>actinobacteria	Micromonospora rosaria	hypothetical protein [Micromonospora rosaria]
WP_067430061.1	-	215	bacteria>actinobacteria	Nocardioides jensenii	hypothetical protein [Nocardioides jensenii]
WP_079554530.1	-	215	bacteria>actinobacteria	Arthrobacter sp. 49Tsu3.1M3	hypothetical protein [Arthrobacter sp. 49Tsu3.1M3]
WP_082563559.1	-	215	bacteria>actinobacteria	Nocardioides	MULTISPECIES: hypothetical protein [Nocardioides]
WP_086847876.1	-	215	bacteria>actinobacteria	Amycolatopsis kentuckyensis	hypothetical protein [Amycolatopsis kentuckyensis]
WP_088985686.1	-	215	bacteria>actinobacteria	Micromonospora echinospora	hypothetical protein [Micromonospora echinospora]
WP_090581139.1	-	215	bacteria>actinobacteria	Arthrobacter sp. ov407	hypothetical protein [Arthrobacter sp. ov407]
WP_090592900.1	-	215	bacteria>actinobacteria	Auraticoccus monumenti	hypothetical protein [Auraticoccus monumenti]
WP_090953578.1	-	215	bacteria>actinobacteria	Arthrobacter sp. ov118	hypothetical protein [Arthrobacter sp. ov118]
WP_091251853.1	-	215	bacteria>actinobacteria	Arthrobacter sp. ok909	hypothetical protein [Arthrobacter sp. ok909]
WP_091405209.1	-	215	bacteria>actinobacteria	Micromonospora saelicesensis	hypothetical protein [Micromonospora saelicesensis]
WP_091440691.1	-	215	bacteria>actinobacteria	Micromonospora yangpuensis	hypothetical protein [Micromonospora yangpuensis]
WP_091639007.1	-	215	bacteria>actinobacteria	Micromonospora pallida	hypothetical protein [Micromonospora pallida]
WP_092382500.1	-	215	bacteria>actinobacteria	Xiangella phaseoli	hypothetical protein [Xiangella phaseoli]
WP_093952051.1	-	215	bacteria>actinobacteria	Amycolatopsis sp. H5	hypothetical protein [Amycolatopsis sp. H5]
ANH39807.1	I601_3401	214	bacteria>actinobacteria	Nocardioides dokdonensis FR1436	peptide chain release factor 1 [Nocardioides dokdonensis FR1436]
WP_012920902.1	-	214	bacteria>actinobacteria	Kribbella flavida	hypothetical protein [Kribbella flavida]
WP_018773160.1	-	214	bacteria>actinobacteria	Arthrobacter sp. 131MFCol6.1	hypothetical protein [Arthrobacter sp. 131MFCol6.1]
WP_020510162.1	-	214	bacteria>actinobacteria	Actinoplanes globisporus	hypothetical protein [Actinoplanes globisporus]
WP_026537251.1	-	214	bacteria>actinobacteria	Arthrobacter sp. 9MFCol3.1	hypothetical protein [Arthrobacter sp. 9MFCol3.1]
WP_033340388.1	-	214	bacteria>actinobacteria	Catenuloplanes japonicus	hypothetical protein [Catenuloplanes japonicus]
WP_051088496.1	-	214	bacteria>actinobacteria	Arthrobacter	MULTISPECIES: hypothetical protein [Arthrobacter]
WP_055803267.1	-	214	bacteria>actinobacteria	Arthrobacter sp. Leaf337	hypothetical protein [Arthrobacter sp. Leaf337]
WP_055975945.1	-	214	bacteria>actinobacteria	Arthrobacter sp. Leaf145	hypothetical protein [Arthrobacter sp. Leaf145]
WP_064722208.1	-	214	bacteria>actinobacteria	Paenarthrobacter nicotinovorans	hypothetical protein [Paenarthrobacter nicotinovorans]
WP_071803534.1	-	214	bacteria>actinobacteria	Couchioplanes caeruleus	hypothetical protein [Couchioplanes caeruleus]
WP_077013954.1	-	214	bacteria>actinobacteria	Kribbella sp. ALI-6-A	hypothetical protein [Kribbella sp. ALI-6-A]
WP_079581383.1	-	214	bacteria>actinobacteria	Arthrobacter sp. 31Cvi3.1E	hypothetical protein [Arthrobacter sp. 31Cvi3.1E]
ELT44908.1	G205_08668	213	bacteria>actinobacteria	Arthrobacter nitrophenolicus	hypothetical protein G205_08668 [Arthrobacter nitrophenolicus]
SDQ43781.1	SAMN05428996_1700	213	bacteria>actinobacteria	Quadrisphaera sp. DSM 44207	hypothetical protein SAMN05428996_1700 [Quadrisphaera sp. DSM 44207]
WP_034715701.1	-	213	bacteria>actinobacteria	Intrasporangium chromatireducens	hypothetical protein [Intrasporangium chromatireducens]
WP_043967548.1	-	213	bacteria>actinobacteria	Micromonospora carbonacea	hypothetical protein [Micromonospora carbonacea]
WP_049822901.1	-	213	bacteria>actinobacteria	Arthrobacter sp. H41	hypothetical protein, partial [Arthrobacter sp. H41]
WP_074474323.1	-	213	bacteria>actinobacteria	Micromonospora carbonacea	hypothetical protein [Micromonospora carbonacea]
WP_077939175.1	-	213	bacteria>actinobacteria	Micromonospora	MULTISPECIES: hypothetical protein [Micromonospora]
WP_098460903.1	-	213	bacteria>actinobacteria	Propionicimonas paludicola	hypothetical protein [Propionicimonas paludicola]
ODB78017.1	A8711_06385	212	bacteria>actinobacteria	Micromonospora sp. II	hypothetical protein A8711_06385 [Micromonospora sp. II]
OHX06710.1	BFV98_28860	212	bacteria>actinobacteria	Micromonospora sp. WMMB235	hypothetical protein BFV98_28860 [Micromonospora sp. WMMB235]
OKJ35871.1	AMK25_28000	212	bacteria>actinobacteria	Micromonospora sp. TSRI0369	hypothetical protein AMK25_28000 [Micromonospora sp. TSRI0369]
PKQ32226.1	CVT62_04170	212	bacteria>actinobacteria	Actinobacteria bacterium HGW-Actinobacteria-2	hypothetical protein CVT62_04170 [Actinobacteria bacterium HGW-Actinobacteria-2]
SCG68389.1	GA0074704_4376	212	bacteria>actinobacteria	Micromonospora siamensis	hypothetical protein GA0074704_4376 [Micromonospora siamensis]
WP_018216242.1	-	212	bacteria>actinobacteria	Salinispora pacifica	hypothetical protein [Salinispora pacifica]
WP_018223388.1	-	212	bacteria>actinobacteria	Salinispora pacifica	hypothetical protein [Salinispora pacifica]
WP_018251539.1	-	212	bacteria>actinobacteria	Salinispora pacifica	hypothetical protein [Salinispora pacifica]
WP_018727340.1	-	212	bacteria>actinobacteria	Salinispora pacifica	hypothetical protein [Salinispora pacifica]
WP_018731666.1	-	212	bacteria>actinobacteria	Salinispora pacifica	hypothetical protein [Salinispora pacifica]
WP_018737027.1	-	212	bacteria>actinobacteria	Salinispora pacifica	hypothetical protein [Salinispora pacifica]
WP_018741300.1	-	212	bacteria>actinobacteria	Salinispora pacifica	hypothetical protein [Salinispora pacifica]
WP_018788241.1	-	212	bacteria>actinobacteria	Micromonospora	MULTISPECIES: hypothetical protein [Micromonospora]
WP_018811200.1	-	212	bacteria>actinobacteria	Salinispora pacifica	hypothetical protein [Salinispora pacifica]
WP_018817327.1	-	212	bacteria>actinobacteria	Salinispora pacifica	hypothetical protein [Salinispora pacifica]
WP_018823293.1	-	212	bacteria>actinobacteria	Salinispora pacifica	hypothetical protein [Salinispora pacifica]
WP_018827387.1	-	212	bacteria>actinobacteria	Salinispora pacifica	hypothetical protein [Salinispora pacifica]
WP_018868789.1	-	212	bacteria>actinobacteria	Salinispora pacifica	hypothetical protein [Salinispora pacifica]
WP_019877387.1	-	212	bacteria>actinobacteria	Sporichthya polymorpha	hypothetical protein [Sporichthya polymorpha]
WP_025619096.1	-	212	bacteria>actinobacteria	Salinispora pacifica	hypothetical protein [Salinispora pacifica]
WP_026275268.1	-	212	bacteria>actinobacteria	Salinispora	MULTISPECIES: hypothetical protein [Salinispora]
WP_027648017.1	-	212	bacteria>actinobacteria	Salinispora pacifica	hypothetical protein [Salinispora pacifica]
WP_027651525.1	-	212	bacteria>actinobacteria	Salinispora pacifica	hypothetical protein [Salinispora pacifica]
WP_027656092.1	-	212	bacteria>actinobacteria	Salinispora pacifica	hypothetical protein [Salinispora pacifica]
WP_027658756.1	-	212	bacteria>actinobacteria	Salinispora pacifica	hypothetical protein [Salinispora pacifica]
WP_028185887.1	-	212	bacteria>actinobacteria	Salinispora pacifica	hypothetical protein [Salinispora pacifica]
WP_029122196.1	-	212	bacteria>actinobacteria	Salinispora pacifica	hypothetical protein [Salinispora pacifica]
WP_029128976.1	-	212	bacteria>actinobacteria	Salinispora pacifica	hypothetical protein [Salinispora pacifica]
WP_046563214.1	-	212	bacteria>actinobacteria	Micromonospora sp. HK10	hypothetical protein [Micromonospora sp. HK10]
WP_088979275.1	-	212	bacteria>actinobacteria	Micromonospora coxensis	hypothetical protein [Micromonospora coxensis]
WP_089156161.1	-	212	bacteria>actinobacteria	Micromonospora sp. NBS 11-29	hypothetical protein [Micromonospora sp. NBS 11-29]
WP_091071857.1	-	212	bacteria>actinobacteria	Micromonospora humi	hypothetical protein [Micromonospora humi]
WP_091270083.1	-	212	bacteria>actinobacteria	Micromonospora chaiyaphumensis	hypothetical protein [Micromonospora chaiyaphumensis]
WP_091290953.1	-	212	bacteria>actinobacteria	Micromonospora halophytica	hypothetical protein [Micromonospora halophytica]
WP_091420387.1	-	212	bacteria>actinobacteria	Micromonospora tulbaghiae	hypothetical protein [Micromonospora tulbaghiae]
WP_091573420.1	-	212	bacteria>actinobacteria	Micromonospora	MULTISPECIES: hypothetical protein [Micromonospora]
WP_091672851.1	-	212	bacteria>actinobacteria	Micromonospora auratinigra	hypothetical protein [Micromonospora auratinigra]
WP_099164396.1	-	212	bacteria>actinobacteria	Micromonospora sp. WMMA2032	hypothetical protein [Micromonospora sp. WMMA2032]
SNS85783.1	SAMN06893096_10986	211	bacteria>actinobacteria	Geodermatophilus pulveris	hypothetical protein SAMN06893096_10986 [Geodermatophilus pulveris]
WP_045741234.1	-	211	bacteria>actinobacteria	Actinoplanes rectilineatus	hypothetical protein [Actinoplanes rectilineatus]
WP_091360577.1	-	211	bacteria>actinobacteria	Frankineae bacterium MT45	hypothetical protein [Frankineae bacterium MT45]
WP_091410053.1	-	211	bacteria>actinobacteria	Friedmanniella luteola	hypothetical protein [Friedmanniella luteola]
KWW99929.1	LI90_1569	210	bacteria>actinobacteria	Streptomyces thermoautotrophicus	hypothetical protein LI90_1569 [Streptomyces thermoautotrophicus]
WP_014441756.1	-	210	bacteria>actinobacteria	Actinoplanes missouriensis	hypothetical protein [Actinoplanes missouriensis]
WP_014688856.1	-	210	bacteria>actinobacteria	Actinoplanes	MULTISPECIES: hypothetical protein [Actinoplanes]
WP_026377752.1	-	210	bacteria>actinobacteria	Aestuariimicrobium kwangyangense	hypothetical protein [Aestuariimicrobium kwangyangense]
WP_026847120.1	-	210	bacteria>actinobacteria	Geodermatophilaceae bacterium URHA0031	hypothetical protein [Geodermatophilaceae bacterium URHA0031]
WP_030439337.1	-	210	bacteria>actinobacteria	Actinoplanes subtropicus	hypothetical protein [Actinoplanes subtropicus]
WP_033360311.1	-	210	bacteria>actinobacteria	Dactylosporangium aurantiacum	hypothetical protein [Dactylosporangium aurantiacum]
WP_034804791.1	-	210	bacteria>actinobacteria	Intrasporangium oryzae	hypothetical protein [Intrasporangium oryzae]
WP_041832100.1	-	210	bacteria>actinobacteria	Actinoplanes sp. N902-109	hypothetical protein [Actinoplanes sp. N902-109]
WP_043427418.1	-	210	bacteria>actinobacteria	Arthrobacter	MULTISPECIES: hypothetical protein [Arthrobacter]
WP_062099210.1	-	210	bacteria>actinobacteria	Arthrobacter sp. ATCC 21022	hypothetical protein [Arthrobacter sp. ATCC 21022]
WP_067704121.1	-	210	bacteria>actinobacteria	Actinoplanes awajinensis	hypothetical protein [Actinoplanes awajinensis]
WP_069694601.1	-	210	bacteria>actinobacteria	Arthrobacter	MULTISPECIES: hypothetical protein [Arthrobacter]
WP_089292184.1	-	210	bacteria>actinobacteria	Actinoplanes regularis	hypothetical protein [Actinoplanes regularis]
WP_091522673.1	-	210	bacteria>actinobacteria	Microlunatus soli	hypothetical protein [Microlunatus soli]
WP_093611189.1	-	210	bacteria>actinobacteria	Actinoplanes philippinensis	hypothetical protein [Actinoplanes philippinensis]
WP_097322434.1	-	210	bacteria>actinobacteria	Actinoplanes atraurantiacus	hypothetical protein [Actinoplanes atraurantiacus]
SDK18110.1	SAMN04487820_105118	209	bacteria>actinobacteria	Actinopolyspora mzabensis	hypothetical protein SAMN04487820_105118 [Actinopolyspora mzabensis]
SEU30516.1	SAMN05421811_11096	209	bacteria>actinobacteria	Nonomuraea wenchangensis	hypothetical protein SAMN05421811_11096 [Nonomuraea wenchangensis]
WP_013492538.1	-	209	bacteria>actinobacteria	Intrasporangium calvum	hypothetical protein [Intrasporangium calvum]
WP_014743194.1	-	209	bacteria>actinobacteria	Modestobacter marinus	hypothetical protein [Modestobacter marinus]
WP_082523128.1	-	209	bacteria>actinobacteria	Geodermatophilus sp. Leaf369	hypothetical protein [Geodermatophilus sp. Leaf369]
WP_089774507.1	-	209	bacteria>actinobacteria	Haloactinobacterium album	hypothetical protein [Haloactinobacterium album]
WP_018586680.1	-	208	bacteria>actinobacteria	Salinispora arenicola	hypothetical protein [Salinispora arenicola]
WP_018792320.1	-	208	bacteria>actinobacteria	Salinispora arenicola	hypothetical protein [Salinispora arenicola]
WP_018795275.1	-	208	bacteria>actinobacteria	Salinispora arenicola	hypothetical protein [Salinispora arenicola]
WP_018824251.1	-	208	bacteria>actinobacteria	Salinispora arenicola	hypothetical protein [Salinispora arenicola]
WP_019031451.1	-	208	bacteria>actinobacteria	Salinispora arenicola	hypothetical protein [Salinispora arenicola]
WP_020144130.1	-	208	bacteria>actinobacteria	Terracoccus sp. 273MFTsu3.1	hypothetical protein [Terracoccus sp. 273MFTsu3.1]
WP_020216903.1	-	208	bacteria>actinobacteria	Salinispora arenicola	hypothetical protein [Salinispora arenicola]
WP_020521137.1	-	208	bacteria>actinobacteria	Catelliglobosispora koreensis	hypothetical protein [Catelliglobosispora koreensis]
WP_026550338.1	-	208	bacteria>actinobacteria	Arthrobacter sp. Br18	hypothetical protein, partial [Arthrobacter sp. Br18]
WP_026863452.1	-	208	bacteria>actinobacteria	Intrasporangiaceae bacterium URHB0013	hypothetical protein [Intrasporangiaceae bacterium URHB0013]
WP_029021708.1	-	208	bacteria>actinobacteria	Salinispora arenicola	hypothetical protein [Salinispora arenicola]
WP_029026748.1	-	208	bacteria>actinobacteria	Salinispora arenicola	hypothetical protein [Salinispora arenicola]
WP_029539250.1	-	208	bacteria>actinobacteria	Salinispora arenicola	hypothetical protein [Salinispora arenicola]
WP_056757097.1	-	208	bacteria>actinobacteria	Terrabacter sp. Root181	hypothetical protein [Terrabacter sp. Root181]
WP_056776780.1	-	208	bacteria>actinobacteria	Terrabacter	MULTISPECIES: hypothetical protein [Terrabacter]
WP_057588230.1	-	208	bacteria>actinobacteria	Terrabacter sp. Soil810	hypothetical protein [Terrabacter sp. Soil810]
WP_070190956.1	-	208	bacteria>actinobacteria	Humibacillus sp. DSM 29435	hypothetical protein [Humibacillus sp. DSM 29435]
WP_081709970.1	-	208	bacteria>actinobacteria	Arthrobacter sp. 35W	hypothetical protein [Arthrobacter sp. 35W]
WP_091726379.1	-	208	bacteria>actinobacteria	Marmoricola scoriae	hypothetical protein [Marmoricola scoriae]
ALE06100.1	AL755_12495	207	bacteria>actinobacteria	Arthrobacter sp. ERGS1:01	hypothetical protein AL755_12495 [Arthrobacter sp. ERGS1:01]
CCG05528.1	BLASA_4734	207	bacteria>actinobacteria	Blastococcus saxobsidens DD2	conserved protein of unknown function [Blastococcus saxobsidens DD2]
KQY64374.1	ASD30_05390	207	bacteria>actinobacteria	Nocardioides sp. Root140	hypothetical protein ASD30_05390 [Nocardioides sp. Root140]
SDP98298.1	SAMN05421507_1385	207	bacteria>actinobacteria	Lentzea jiangxiensis	hypothetical protein SAMN05421507_1385 [Lentzea jiangxiensis]
SDY39155.1	SAMN05421504_105404	207	bacteria>actinobacteria	Amycolatopsis xylanica	hypothetical protein SAMN05421504_105404 [Amycolatopsis xylanica]
WP_028660782.1	-	207	bacteria>actinobacteria	Nocardioides insulae	hypothetical protein [Nocardioides insulae]
WP_084181538.1	-	207	bacteria>actinobacteria	Jatrophihabitans endophyticus	hypothetical protein [Jatrophihabitans endophyticus]
WP_085870766.1	-	207	bacteria>actinobacteria	Nocardioides	MULTISPECIES: hypothetical protein [Nocardioides]
WP_090856186.1	-	207	bacteria>actinobacteria	Nocardioides lianchengensis	hypothetical protein [Nocardioides lianchengensis]
KOV81688.1	ADL03_28020	206	bacteria>actinobacteria	Nocardia sp. NRRL S-836	hypothetical protein ADL03_28020 [Nocardia sp. NRRL S-836]
KOX21646.1	ADK67_26395	206	bacteria>actinobacteria	Saccharothrix sp. NRRL B-16348	hypothetical protein ADK67_26395 [Saccharothrix sp. NRRL B-16348]
WP_027660041.1	-	206	bacteria>actinobacteria	Salinispora pacifica	hypothetical protein [Salinispora pacifica]
WP_038679040.1	-	206	bacteria>actinobacteria	Pimelobacter simplex	hypothetical protein [Pimelobacter simplex]
WP_051265113.1	-	206	bacteria>actinobacteria	Nakamurella lactea	hypothetical protein [Nakamurella lactea]
WP_056893581.1	-	206	bacteria>actinobacteria	Nocardioides	MULTISPECIES: hypothetical protein [Nocardioides]
WP_084733927.1	-	206	bacteria>actinobacteria	Tetrasphaera jenkinsii	hypothetical protein [Tetrasphaera jenkinsii]
ETK38112.1	MPTA5024_00440	205	bacteria>actinobacteria	Microbispora sp. ATCC PTA-5024	hypothetical protein MPTA5024_00440 [Microbispora sp. ATCC PTA-5024]
PIF84612.1	CLT68_5895	205	bacteria>actinobacteria	Micromonospora sp. CNZ299	hypothetical protein CLT68_5895 [Micromonospora sp. CNZ299]
SCF02750.1	GA0070612_3147	205	bacteria>actinobacteria	Micromonospora chokoriensis	hypothetical protein GA0070612_3147 [Micromonospora chokoriensis]
SCG58303.1	GA0070619_3789	205	bacteria>actinobacteria	Micromonospora zamorensis	hypothetical protein GA0070619_3789 [Micromonospora zamorensis]
WP_020488053.1	-	205	bacteria>firmicutes	Dehalobacter sp. FTH1	hypothetical protein [Dehalobacter sp. FTH1]
WP_028472943.1	-	205	bacteria>actinobacteria	Nocardioides alkalitolerans	hypothetical protein [Nocardioides alkalitolerans]
WP_028654050.1	-	205	bacteria>actinobacteria	Nocardioides sp. J54	hypothetical protein [Nocardioides sp. J54]
WP_051110550.1	-	205	bacteria>actinobacteria	Longispora albida	hypothetical protein [Longispora albida]
WP_062069455.1	-	205	bacteria>actinobacteria	Arthrobacter sp. EpRS71	hypothetical protein [Arthrobacter sp. EpRS71]
EGD44195.1	NBCG_01390	204	bacteria>actinobacteria	Nocardioidaceae bacterium Broad-1	hypothetical protein NBCG_01390 [Nocardioidaceae bacterium Broad-1]
SDL02024.1	SAMN05421869_12188	204	bacteria>actinobacteria	Nonomuraea jiangxiensis	hypothetical protein SAMN05421869_12188 [Nonomuraea jiangxiensis]
WP_018744267.1	-	204	bacteria>actinobacteria	Salinispora pacifica	hypothetical protein [Salinispora pacifica]
WP_045549653.1	-	204	bacteria>actinobacteria	Nocardioides luteus	hypothetical protein [Nocardioides luteus]
WP_056538808.1	-	204	bacteria>actinobacteria	Marmoricola sp. Leaf446	hypothetical protein [Marmoricola sp. Leaf446]
WP_056716858.1	-	204	bacteria>actinobacteria	Nocardioides	MULTISPECIES: hypothetical protein [Nocardioides]
WP_068115146.1	-	204	bacteria>actinobacteria	Nocardioides dokdonensis	hypothetical protein [Nocardioides dokdonensis]
WP_091041058.1	-	204	bacteria>actinobacteria	Nocardioides sp. YR527	hypothetical protein [Nocardioides sp. YR527]
PID97096.1	CSA84_01755	203	bacteria>actinobacteria	Actinomycetales bacterium	hypothetical protein CSA84_01755 [Actinomycetales bacterium]
SCL59013.1	GA0070617_3979	203	bacteria>actinobacteria	Micromonospora yangpuensis	hypothetical protein GA0070617_3979 [Micromonospora yangpuensis]
WP_028652227.1	-	203	bacteria>actinobacteria	Nocardioides halotolerans	hypothetical protein [Nocardioides halotolerans]
WP_056756243.1	-	203	bacteria>actinobacteria	Nocardioides sp. Root190	hypothetical protein [Nocardioides sp. Root190]
WP_091022646.1	-	203	bacteria>actinobacteria	Nocardioides szechwanensis	hypothetical protein [Nocardioides szechwanensis]
KJK10673.1	UB45_18205	202	bacteria>actinobacteria	Terrabacter sp. 28	hypothetical protein UB45_18205 [Terrabacter sp. 28]
WP_011756001.1	-	202	bacteria>actinobacteria	Nocardioides	MULTISPECIES: hypothetical protein [Nocardioides]
WP_028644209.1	-	202	bacteria>actinobacteria	Nocardioides sp. URHA0020	hypothetical protein [Nocardioides sp. URHA0020]
WP_062288405.1	-	202	bacteria>actinobacteria	Arthrobacter alpinus	hypothetical protein [Arthrobacter alpinus]
WP_084635265.1	-	202	bacteria>actinobacteria	Propionicicella superfundia	hypothetical protein [Propionicicella superfundia]
WP_091114157.1	-	202	bacteria>actinobacteria	Nocardioides psychrotolerans	hypothetical protein [Nocardioides psychrotolerans]
ALV45540.1	MB46_08580	201	bacteria>actinobacteria	Arthrobacter sp. A3	hypothetical protein MB46_08580 [Arthrobacter sp. A3]
SCF25428.1	GA0070561_4659	201	bacteria>actinobacteria	Micromonospora saelicesensis	hypothetical protein GA0070561_4659 [Micromonospora saelicesensis]
SEE13628.1	SAMN04489740_0734	201	bacteria>actinobacteria	Arthrobacter alpinus	hypothetical protein SAMN04489740_0734 [Arthrobacter alpinus]
WP_027860720.1	-	201	bacteria>actinobacteria	Marmoricola sp. URHB0036	hypothetical protein [Marmoricola sp. URHB0036]
WP_028638375.1	-	201	bacteria>actinobacteria	Nocardioides sp. URHA0032	hypothetical protein [Nocardioides sp. URHA0032]
WP_056147455.1	-	201	bacteria>actinobacteria	Nocardioides	MULTISPECIES: hypothetical protein [Nocardioides]
WP_056909505.1	-	201	bacteria>actinobacteria	Nocardioides sp. Root122	hypothetical protein [Nocardioides sp. Root122]
WP_090971330.1	-	201	bacteria>actinobacteria	Nocardioides exalbidus	hypothetical protein [Nocardioides exalbidus]
WP_091199345.1	-	201	bacteria>actinobacteria	Nocardioides alpinus	hypothetical protein [Nocardioides alpinus]
SBT65479.1	GA0070622_2474	200	bacteria>actinobacteria	Micromonospora sediminicola	hypothetical protein GA0070622_2474 [Micromonospora sediminicola]
SCF46649.1	GA0070564_11430	200	bacteria>actinobacteria	Micromonospora mirobrigensis	hypothetical protein GA0070564_11430 [Micromonospora mirobrigensis]
WP_010833022.1	-	200	bacteria>actinobacteria	Nocardioides sp. CF8	hypothetical protein [Nocardioides sp. CF8]
WP_024816840.1	-	200	bacteria>actinobacteria	Arthrobacter sp. 31Y	hypothetical protein [Arthrobacter sp. 31Y]
WP_056606406.1	-	200	bacteria>actinobacteria	Nocardioides sp. Soil774	hypothetical protein [Nocardioides sp. Soil774]
WP_056888669.1	-	200	bacteria>actinobacteria	Nocardioides sp. Soil777	hypothetical protein [Nocardioides sp. Soil777]
WP_056924540.1	-	200	bacteria>actinobacteria	Nocardioides sp. Soil805	hypothetical protein [Nocardioides sp. Soil805]
WP_062006717.1	-	200	bacteria>actinobacteria	Arthrobacter alpinus	hypothetical protein [Arthrobacter alpinus]
WP_089595240.1	-	200	bacteria>actinobacteria	Arthrobacter sp. YN	hypothetical protein [Arthrobacter sp. YN]
WP_011774970.1	-	199	bacteria>actinobacteria	Micrococcaceae	MULTISPECIES: hypothetical protein [Micrococcaceae]
WP_017197539.1	-	199	bacteria>actinobacteria	Arthrobacter sp. M2012083	hypothetical protein [Arthrobacter sp. M2012083]
WP_038462740.1	-	199	bacteria>actinobacteria	Arthrobacter sp. PAMC 25486	hypothetical protein [Arthrobacter sp. PAMC 25486]
WP_090821683.1	-	199	bacteria>actinobacteria	Arthrobacter	MULTISPECIES: hypothetical protein [Arthrobacter]
WP_091467833.1	-	199	bacteria>actinobacteria	Arthrobacter sp. HG	hypothetical protein [Arthrobacter sp. HG]
SCF32354.1	GA0070215_117104	198	bacteria>actinobacteria	Micromonospora marina	hypothetical protein GA0070215_117104 [Micromonospora marina]
SCG15329.1	GA0070610_1560	198	bacteria>actinobacteria	Micromonospora echinofusca	hypothetical protein GA0070610_1560 [Micromonospora echinofusca]
SNY50595.1	SAMN05421748_11155	198	bacteria>actinobacteria	Actinoplanes atraurantiacus	hypothetical protein SAMN05421748_11155 [Actinoplanes atraurantiacus]
WP_026540821.1	-	198	bacteria>actinobacteria	Paenarthrobacter nicotinovorans	hypothetical protein [Paenarthrobacter nicotinovorans]
WP_026546887.1	-	198	bacteria>actinobacteria	Paenarthrobacter nicotinovorans	hypothetical protein [Paenarthrobacter nicotinovorans]
WP_055995591.1	-	198	bacteria>actinobacteria	Janibacter sp. Soil728	hypothetical protein [Janibacter sp. Soil728]
WP_091551917.1	-	198	bacteria>actinobacteria	Arthrobacter sp. UNCCL28	hypothetical protein [Arthrobacter sp. UNCCL28]
SNR36977.1	SAMN06272737_104196	197	bacteria>actinobacteria	Blastococcus sp. DSM 44272	hypothetical protein SAMN06272737_104196 [Blastococcus sp. DSM 44272]
WP_017935269.1	-	197	bacteria>actinobacteria	Nocardioides sp. Iso805N	hypothetical protein [Nocardioides sp. Iso805N]
WP_091180115.1	-	197	bacteria>actinobacteria	Friedmanniella flava	hypothetical protein [Friedmanniella flava]
AGL15695.1	L083_2185	196	bacteria>actinobacteria	Actinoplanes sp. N902-109	hypothetical protein L083_2185 [Actinoplanes sp. N902-109]
KIA72773.1	ANMWB30_25410	196	bacteria>actinobacteria	Arthrobacter sp. MWB30	hypothetical protein ANMWB30_25410 [Arthrobacter sp. MWB30]
WP_030484261.1	-	196	bacteria>actinobacteria	Marmoricola aequoreus	hypothetical protein [Marmoricola aequoreus]
WP_082369237.1	-	196	bacteria>actinobacteria	Arthrobacter sp. ERGS1:01	hypothetical protein [Arthrobacter sp. ERGS1:01]
KKJ95205.1	LQ51_26960	193	bacteria>actinobacteria	Micromonospora sp. HK10	hypothetical protein LQ51_26960 [Micromonospora sp. HK10]
WP_028276476.1	-	192	bacteria>actinobacteria	Arthrobacter sp. I3	hypothetical protein, partial [Arthrobacter sp. I3]
WP_091760403.1	-	192	bacteria>actinobacteria	Phycicoccus cremeus	hypothetical protein [Phycicoccus cremeus]
WP_068316329.1	-	191	bacteria>actinobacteria	Janibacter anophelis	hypothetical protein [Janibacter anophelis]
CCH33465.1	BN6_62150	190	bacteria>actinobacteria	Saccharothrix espanaensis DSM 44229	hypothetical protein BN6_62150 [Saccharothrix espanaensis DSM 44229]
WP_068323859.1	-	189	bacteria>actinobacteria	Janibacter terrae	hypothetical protein [Janibacter terrae]
WP_068421331.1	-	189	bacteria>actinobacteria	Janibacter terrae	hypothetical protein [Janibacter terrae]
GAT86946.1	CVCC1112_1605	187	bacteria>actinobacteria	Paenarthrobacter nicotinovorans	hypothetical protein CVCC1112_1605 [Paenarthrobacter nicotinovorans]
WP_045192470.1	-	187	bacteria>actinobacteria	Terrabacter sp. 28	hypothetical protein [Terrabacter sp. 28]
WP_068250845.1	-	187	bacteria>actinobacteria	Janibacter corallicola	hypothetical protein [Janibacter corallicola]
WP_056921252.1	-	186	bacteria>actinobacteria	Phycicoccus sp. Soil802	hypothetical protein [Phycicoccus sp. Soil802]
WP_057378855.1	-	186	bacteria>actinobacteria	Phycicoccus sp. Soil803	hypothetical protein [Phycicoccus sp. Soil803]
WP_068258294.1	-	186	bacteria>actinobacteria	Janibacter limosus	hypothetical protein [Janibacter limosus]
WP_072624666.1	-	186	bacteria>actinobacteria	Janibacter indicus	hypothetical protein [Janibacter indicus]
WP_084451627.1	-	186	bacteria>actinobacteria	Janibacter indicus	hypothetical protein [Janibacter indicus]
SDM95272.1	SAMN05660642_03681	185	bacteria>actinobacteria	Geodermatophilus siccatus	hypothetical protein SAMN05660642_03681 [Geodermatophilus siccatus]
WP_048551658.1	-	184	bacteria>actinobacteria	Tetrasphaera japonica	hypothetical protein [Tetrasphaera japonica]
WP_068275945.1	-	183	bacteria>actinobacteria	Janibacter melonis	hypothetical protein [Janibacter melonis]
SHN85434.1	SAMN05660350_03761	182	bacteria>actinobacteria	Geodermatophilus obscurus	hypothetical protein SAMN05660350_03761 [Geodermatophilus obscurus]
WP_056885212.1	-	181	bacteria>actinobacteria	Phycicoccus sp. Soil748	hypothetical protein [Phycicoccus sp. Soil748]
WP_091782640.1	-	179	bacteria>actinobacteria	Phycicoccus dokdonensis	hypothetical protein [Phycicoccus dokdonensis]
WP_056914344.1	-	178	bacteria>actinobacteria	Phycicoccus sp. Root563	hypothetical protein [Phycicoccus sp. Root563]
WP_056918962.1	-	178	bacteria>actinobacteria	Phycicoccus sp. Root101	hypothetical protein [Phycicoccus sp. Root101]
WP_009774976.1	-	177	bacteria>actinobacteria	Janibacter sp. HTCC2649	hypothetical protein [Janibacter sp. HTCC2649]
WP_035915559.1	-	177	bacteria>actinobacteria	Knoellia sinensis	hypothetical protein [Knoellia sinensis]
KHL11283.1	LK11_42810	176	bacteria>actinobacteria	Mumia flava	UNVERIFIED: hypothetical protein LK11_42810 [Mumia flava]
WP_035903137.1	-	176	bacteria>actinobacteria	Knoellia subterranea	hypothetical protein [Knoellia subterranea]
WP_035932215.1	-	176	bacteria>actinobacteria	Knoellia aerolata	hypothetical protein [Knoellia aerolata]
WP_035949717.1	-	176	bacteria>actinobacteria	Knoellia flava	hypothetical protein [Knoellia flava]
WP_056143992.1	-	176	bacteria>actinobacteria	Knoellia sp. Soil729	hypothetical protein [Knoellia sp. Soil729]
SDU94556.1	SAMN04488544_2381	175	bacteria>actinobacteria	Friedmanniella sagamiharensis	hypothetical protein SAMN04488544_2381 [Friedmanniella sagamiharensis]
WP_010851530.1	-	175	bacteria>actinobacteria	Tetrasphaera elongata	hypothetical protein [Tetrasphaera elongata]
WP_084696765.1	-	174	bacteria>actinobacteria	Phycicoccus jejuensis	hypothetical protein [Phycicoccus jejuensis]
WP_055812447.1	-	173	bacteria>actinobacteria	Tetrasphaera sp. Soil756	hypothetical protein [Tetrasphaera sp. Soil756]
SEQ59021.1	SAMN05421756_104162	172	bacteria>actinobacteria	Friedmanniella flava	hypothetical protein SAMN05421756_104162 [Friedmanniella flava]
WP_024366471.1	-	146	bacteria>actinobacteria	Arthrobacter sp. TB 26	hypothetical protein, partial [Arthrobacter sp. TB 26]
EHK85366.1	SZMC14600_16606	143	bacteria>actinobacteria	Saccharomonospora azurea SZMC 14600	hypothetical protein SZMC14600_16606, partial [Saccharomonospora azurea SZMC 14600]
OLE24119.1	AUG44_20210	142	bacteria>actinobacteria	Actinobacteria bacterium 13_1_20CM_3_71_11	hypothetical protein AUG44_20210, partial [Actinobacteria bacterium 13_1_20CM_3_71_11]
SDC19711.1	SAMN05660690_0885	126	bacteria>actinobacteria	Geodermatophilus telluris	hypothetical protein SAMN05660690_0885 [Geodermatophilus telluris]
BAS18185.1	AHiyo8_64880	119	bacteria>actinobacteria	Arthrobacter sp. Hiyo8	hypothetical protein AHiyo8_64880 [Arthrobacter sp. Hiyo8]
KMS83974.1	ACZ91_50300	86	bacteria>actinobacteria	Streptomyces regensis	hypothetical protein ACZ91_50300, partial [Streptomyces regensis]

3. bacteroidetes VLRF1 (bVLRF1) family

##;the bVLRF1 clade is predicted to lack any genunie domain fusions; however, it does
##;contain two conserved N-terminal beta-strands, which we predict form an extension
##;to the core VLRF1 domain and may have originally been derived from the codon
##;recognition domain.

EJF10944.1	O71_06067	340	bacteria>bacteroidetes	Pontibacter sp. BAB1700	hypothetical protein O71_06067 [Pontibacter sp. BAB1700]
WP_040573710.1	-	281	bacteria>bacteroidetes	Pontibacter	MULTISPECIES: hypothetical protein [Pontibacter]
WP_076666794.1	-	271	bacteria>bacteroidetes	Pontibacter indicus	hypothetical protein [Pontibacter indicus]
SFV00446.1	SAMN04487941_4057	260	bacteria>bacteroidetes	Pontibacter akesuensis	hypothetical protein SAMN04487941_4057 [Pontibacter akesuensis]
WP_018478774.1	-	260	bacteria>bacteroidetes	Pontibacter roseus	hypothetical protein [Pontibacter roseus]
WP_073854991.1	-	258	bacteria>bacteroidetes	Pontibacter sp. S10-8	hypothetical protein [Pontibacter sp. S10-8]
WP_092103661.1	-	254	bacteria>bacteroidetes	Pontibacter chinhatensis	hypothetical protein [Pontibacter chinhatensis]
WP_017731912.1	-	252	bacteria>bacteroidetes	Nafulsella turpanensis	hypothetical protein [Nafulsella turpanensis]
WP_066509551.1	-	251	bacteria>bacteroidetes	Rufibacter sp. DG15C	hypothetical protein [Rufibacter sp. DG15C]
OJJ17315.1	BKI52_31945	250	bacteria>bacteroidetes	marine bacterium AO1-C	hypothetical protein BKI52_31945 [marine bacterium AO1-C]
WP_002703322.1	-	250	bacteria>bacteroidetes	Microscilla marina	hypothetical protein [Microscilla marina]
WP_067547328.1	-	250	bacteria>bacteroidetes	Algoriphagus sp. M8-2	hypothetical protein [Algoriphagus sp. M8-2]
WP_062545422.1	-	249	bacteria>bacteroidetes	Rufibacter tibetensis	hypothetical protein [Rufibacter tibetensis]
WP_014798631.1	-	247	bacteria>bacteroidetes	Bernardetia litoralis	hypothetical protein [Bernardetia litoralis]
WP_051295788.1	-	247	bacteria>bacteroidetes	Eisenibacter elegans	hypothetical protein [Eisenibacter elegans]
PIY08114.1	COZ18_13970	243	bacteria>bacteroidetes	Flexibacter sp. CG_4_10_14_3_um_filter_32_15	hypothetical protein COZ18_13970 [Flexibacter sp. CG_4_10_14_3_um_filter_32_15]
WP_009053428.1	-	243	bacteria>bacteroidetes	Nitritalea halalkaliphila	hypothetical protein [Nitritalea halalkaliphila]
WP_025604632.1	-	243	bacteria>bacteroidetes	Pontibacter actiniarum	hypothetical protein [Pontibacter actiniarum]
WP_066835988.1	-	241	bacteria>bacteroidetes	Rufibacter ruber	hypothetical protein [Rufibacter ruber]
WP_068840199.1	-	240	bacteria>bacteroidetes	Pontibacter akesuensis	hypothetical protein [Pontibacter akesuensis]
WP_014773396.1	-	239	bacteria>bacteroidetes	Belliella baltica	hypothetical protein [Belliella baltica]
WP_008200803.1	-	238	bacteria>bacteroidetes	Algoriphagus machipongonensis	hypothetical protein [Algoriphagus machipongonensis]
WP_009032562.1	-	238	bacteria>bacteroidetes	Indibacter alkaliphilus	hypothetical protein [Indibacter alkaliphilus]
WP_057936408.1	-	238	bacteria>bacteroidetes	Algoriphagus resistens	hypothetical protein [Algoriphagus resistens]
WP_074224130.1	-	238	bacteria>bacteroidetes	Algoriphagus halophilus	hypothetical protein [Algoriphagus halophilus]
WP_009183449.1	-	237	bacteria>bacteroidetes	Cecembia lonarensis	hypothetical protein [Cecembia lonarensis]
WP_019600284.1	-	237	bacteria>bacteroidetes	Rhodonellum	MULTISPECIES: hypothetical protein [Rhodonellum]
WP_046311552.1	-	237	bacteria>bacteroidetes	Pontibacter korlensis	hypothetical protein [Pontibacter korlensis]
WP_075351281.1	-	237	bacteria>bacteroidetes	Algoriphagus marinus	hypothetical protein [Algoriphagus marinus]
WP_084121832.1	-	237	bacteria>bacteroidetes	Aquiflexum balticum	hypothetical protein [Aquiflexum balticum]
WP_087938147.1	-	237	bacteria>bacteroidetes	Algoriphagus faecimaris	hypothetical protein [Algoriphagus faecimaris]
WP_091691780.1	-	237	bacteria>bacteroidetes	Algoriphagus locisalis	hypothetical protein [Algoriphagus locisalis]
WP_008628892.1	-	236	bacteria>bacteroidetes	Mariniradius saccharolyticus	hypothetical protein [Mariniradius saccharolyticus]
WP_018473944.1	-	236	bacteria>bacteroidetes	Echinicola pacifica	hypothetical protein [Echinicola pacifica]
WP_076499750.1	-	236	bacteria>bacteroidetes	Belliella pelovolcani	hypothetical protein [Belliella pelovolcani]
WP_086501375.1	-	236	bacteria>bacteroidetes	Algoriphagus ratkowskyi	hypothetical protein [Algoriphagus ratkowskyi]
WP_086541575.1	-	236	bacteria>bacteroidetes	Algoriphagus antarcticus	hypothetical protein [Algoriphagus antarcticus]
WP_089240947.1	-	236	bacteria>bacteroidetes	Belliella buryatensis	hypothetical protein [Belliella buryatensis]
WP_092789693.1	-	236	bacteria>bacteroidetes	Algoriphagus hitonicola	hypothetical protein [Algoriphagus hitonicola]
WP_092897802.1	-	236	bacteria>bacteroidetes	Algoriphagus aquimarinus	hypothetical protein [Algoriphagus aquimarinus]
SEG12643.1	SAMN03080598_02566	235	bacteria>bacteroidetes	Algoriphagus boritolerans DSM 17298 = JCM 18970	hypothetical protein SAMN03080598_02566 [Algoriphagus boritolerans DSM 17298 = JCM 18970]
SNS87147.1	SAMN06296052_11577	235	bacteria>bacteroidetes	Pontibacter ummariensis	hypothetical protein SAMN06296052_11577 [Pontibacter ummariensis]
WP_024282443.1	-	235	bacteria>bacteroidetes	Algoriphagus marincola	hypothetical protein [Algoriphagus marincola]
WP_035072667.1	-	235	bacteria>bacteroidetes	Anditalea andensis	hypothetical protein [Anditalea andensis]
WP_051260724.1	-	235	bacteria>bacteroidetes	Algoriphagus marincola	hypothetical protein [Algoriphagus marincola]
WP_051315124.1	-	235	bacteria>bacteroidetes	Algoriphagus terrigena	hypothetical protein [Algoriphagus terrigena]
WP_092729863.1	-	235	bacteria>bacteroidetes	Algoriphagus alkaliphilus	hypothetical protein [Algoriphagus alkaliphilus]
PIQ20891.1	COW65_12350	234	bacteria>bacteroidetes	Cytophagales bacterium CG18_big_fil_WC_8_21_14_2_50_42_9	hypothetical protein COW65_12350 [Cytophagales bacterium CG18_big_fil_WC_8_21_14_2_50_42_9]
WP_010852296.1	-	234	bacteria>bacteroidetes	Lunatimonas lonarensis	hypothetical protein [Lunatimonas lonarensis]
WP_062616153.1	-	234	bacteria>bacteroidetes	Flammeovirga sp. SJP92	hypothetical protein [Flammeovirga sp. SJP92]
WP_026462407.1	-	233	bacteria>bacteroidetes	Adhaeribacter aquaticus	hypothetical protein [Adhaeribacter aquaticus]
WP_048919991.1	-	233	bacteria>bacteroidetes	Rufibacter sp. DG31D	hypothetical protein [Rufibacter sp. DG31D]
WP_051315894.1	-	233	bacteria>bacteroidetes	Algoriphagus vanfongensis	hypothetical protein [Algoriphagus vanfongensis]
WP_073570220.1	-	233	bacteria>bacteroidetes	Algoriphagus zhangzhouensis	hypothetical protein [Algoriphagus zhangzhouensis]
WP_077350233.1	-	233	bacteria>bacteroidetes	Algoriphagus sp. A40	hypothetical protein [Algoriphagus sp. A40]
WP_091650797.1	-	233	bacteria>bacteroidetes	Algoriphagus ornithinivorans	hypothetical protein [Algoriphagus ornithinivorans]
WP_015267208.1	-	232	bacteria>bacteroidetes	Echinicola vietnamensis	hypothetical protein [Echinicola vietnamensis]
WP_026951646.1	-	231	bacteria>bacteroidetes	Algoriphagus mannitolivorans	hypothetical protein [Algoriphagus mannitolivorans]
WP_051203823.1	-	231	bacteria>bacteroidetes	Hugenholtzia roseola	hypothetical protein [Hugenholtzia roseola]
WP_092169649.1	-	231	bacteria>bacteroidetes	Cyclobacterium halophilum	hypothetical protein [Cyclobacterium halophilum]
PIQ47286.1	COW03_15965	230	bacteria>bacteroidetes	Cytophagales bacterium CG12_big_fil_rev_8_21_14_0_65_40_12	hypothetical protein COW03_15965 [Cytophagales bacterium CG12_big_fil_rev_8_21_14_0_65_40_12]
WP_007276807.1	-	230	bacteria>lentisphaerae	Lentisphaera araneosa	hypothetical protein [Lentisphaera araneosa]
WP_061984517.1	-	229	bacteria>bacteroidetes	Flammeovirgaceae bacterium 311	hypothetical protein [Flammeovirgaceae bacterium 311]
WP_073093936.1	-	228	bacteria>bacteroidetes	Cyclobacterium lianum	hypothetical protein [Cyclobacterium lianum]
WP_053224652.1	-	227	bacteria>bacteroidetes	Roseivirga seohaensis	hypothetical protein [Roseivirga seohaensis]
WP_062300159.1	-	227	bacteria>bacteroidetes	Roseivirga seohaensis	hypothetical protein [Roseivirga seohaensis]
WP_062594005.1	-	227	bacteria>bacteroidetes	Roseivirga ehrenbergii	hypothetical protein [Roseivirga ehrenbergii]
WP_014022263.1	-	226	bacteria>bacteroidetes	Cyclobacterium marinum	hypothetical protein [Cyclobacterium marinum]
WP_020891528.1	-	226	bacteria>bacteroidetes	Cyclobacterium qasimii	hypothetical protein [Cyclobacterium qasimii]
WP_048643676.1	-	226	bacteria>bacteroidetes	Cyclobacterium amurskyense	hypothetical protein [Cyclobacterium amurskyense]
WP_073118735.1	-	225	bacteria>bacteroidetes	Reichenbachiella agariperforans	hypothetical protein [Reichenbachiella agariperforans]
XP_005646001.1	COCSUDRAFT_66896	225	eukaryota>viridiplantae>chlorophyta	Coccomyxa subellipsoidea C-169	hypothetical protein COCSUDRAFT_66896 [Coccomyxa subellipsoidea C-169]
WP_013453031.1	-	224	bacteria>bacteroidetes	Marivirga tractuosa	hypothetical protein [Marivirga tractuosa]
WP_068413778.1	-	224	bacteria>bacteroidetes	Roseivirga echinicomitans	hypothetical protein [Roseivirga echinicomitans]
WP_090259905.1	-	224	bacteria>bacteroidetes	Fabibacter pacificus	hypothetical protein [Fabibacter pacificus]
WP_085517541.1	-	223	bacteria>bacteroidetes	Marivirga sericea	hypothetical protein [Marivirga sericea]
WP_068224041.1	-	222	bacteria>bacteroidetes	Roseivirga spongicola	hypothetical protein [Roseivirga spongicola]
WP_084370467.1	-	222	bacteria>bacteroidetes	Reichenbachiella faecimaris	hypothetical protein [Reichenbachiella faecimaris]
WP_009579783.1	-	221	bacteria>bacteroidetes	Fulvivirga imtechensis	hypothetical protein [Fulvivirga imtechensis]
WP_052444386.1	-	220	bacteria>bacteroidetes	Flammeovirga sp. OC4	hypothetical protein [Flammeovirga sp. OC4]
WP_066215457.1	-	220	bacteria>bacteroidetes	Flammeovirga sp. MY04	hypothetical protein [Flammeovirga sp. MY04]
WP_088125005.1	-	220	bacteria>bacteroidetes	Fabibacter misakiensis	hypothetical protein [Fabibacter misakiensis]
SMF74470.1	SAMN06296036_12847	219	bacteria>proteobacteria	Pseudobacteriovorax antillogorgiicola	hypothetical protein SAMN06296036_12847 [Pseudobacteriovorax antillogorgiicola]
WP_044220529.1	-	219	bacteria>bacteroidetes	Flammeovirga pacifica	hypothetical protein [Flammeovirga pacifica]
WP_069840927.1	-	204	bacteria>bacteroidetes	Fabibacter sp. 4D4	hypothetical protein [Fabibacter sp. 4D4]
OEK05552.1	BFP71_11380	199	bacteria>bacteroidetes	Fabibacter misakiensis	hypothetical protein BFP71_11380 [Fabibacter misakiensis]
AHM58668.1	D770_01980	197	bacteria>bacteroidetes	Flammeovirgaceae bacterium 311	hypothetical protein D770_01980 [Flammeovirgaceae bacterium 311]
GAQ88565.1	KFL_004400030	190	eukaryota>viridiplantae	Klebsormidium nitens	hypothetical protein KFL_004400030 [Klebsormidium nitens]
OLS24615.1	HeimC3_18870	187	archaea>asgard group	Candidatus Heimdallarchaeota archaeon LC_3	hypothetical protein HeimC3_18870 [Candidatus Heimdallarchaeota archaeon LC_3]
WP_099598532.1	-	170	bacteria>bacteroidetes	Reichenbachiella sp. 5M10	hypothetical protein [Reichenbachiella sp. 5M10]
WP_100626964.1	-	164	bacteria>bacteroidetes	Algoriphagus sp. XAY3209	hypothetical protein [Algoriphagus sp. XAY3209]
XP_005845277.1	CHLNCDRAFT_53862	435	eukaryota>viridiplantae>chlorophyta	Chlorella variabilis	hypothetical protein CHLNCDRAFT_53862 [Chlorella variabilis]
XP_013903721.1	MNEG_3249	150	eukaryota>viridiplantae>chlorophyta	Monoraphidium neglectum	hypothetical protein MNEG_3249 [Monoraphidium neglectum]
GAX75057.1	CEUSTIGMA_g2501.t1	304	eukaryota>viridiplantae>chlorophyta	Chlamydomonas eustigma	hypothetical protein CEUSTIGMA_g2501.t1 [Chlamydomonas eustigma]
KXZ41914.1	GPECTOR_244g595	744	eukaryota>viridiplantae>chlorophyta	Gonium pectorale	hypothetical protein GPECTOR_244g595 [Gonium pectorale]
PIE24939.1	CSA62_01745	222	bacteria>planctomycetes	Planctomycetes bacterium	hypothetical protein CSA62_01745 [Planctomycetes bacterium]

4. eukaryotic VLRF1 family

a. canonical Vms1-like architecture and variants

##;The core architecture contains the following domains/motifs: Reil-like Zinc finger, VLRF domain (with N-
##;terminal extension), a characteristic, highly-charged alpha-helix, 3 Ankyrin repeats, a region enriched
##;in arginine residues ('R patch'), and a VIM motif (Cdc48-interacting motif). A large assemblage of these
##;proteins contains a further zinc-binding treble clef domain at the extreme C-terminus, the VTC domain
##;(for Vms1-like Treble Clef domain).

#;VTC-contianing versions

###;some with unusual fusions

OAQ32784.1	K457DRAFT_29647	890	eukaryota>fungi	Mortierella elongata AG-77	hypothetical protein K457DRAFT_29647 [Mortierella elongata AG-77].
OAQ24774.1	K457DRAFT_23807	810	eukaryota>fungi	Mortierella elongata AG-77	hypothetical protein K457DRAFT_23807 [Mortierella elongata AG-77].
KFH62947.1	MVEG_10985	776	eukaryota>fungi	Mortierella verticillata NRRL 6337	hypothetical protein MVEG_10985 [Mortierella verticillata NRRL 6337].
CDI98778.1	EmuJ_000265000	895	eukaryota>metazoa	Echinococcus multilocularis	Ankyrin repeats (many copies) [Echinococcus multilocularis].
CDS24153.1	EgrG_000265000	893	eukaryota>metazoa	Echinococcus granulosus	ankyrin repeat and zinc finger domain containing protein [Echinococcus granulosus].
XP_012795213.1	MS3_03688	893	eukaryota>metazoa	Schistosoma haematobium	Ankyrin repeat and zinc finger domain-containing protein 1 [Schistosoma haematobium].
XP_006004356.1	ANKZF1	893	eukaryota>metazoa>chordata>vertebrata	Latimeria chalumnae	PREDICTED: ankyrin repeat and zinc finger domain-containing protein 1 isoform X1 [Latimeria chalumnae].
XP_004346890.2	CAOG_05205	890	eukaryota	Capsaspora owczarzaki ATCC 30864	hypothetical protein CAOG_05205 [Capsaspora owczarzaki ATCC 30864].
XP_006004357.1	ANKZF1	889	eukaryota>metazoa>chordata>vertebrata	Latimeria chalumnae	PREDICTED: ankyrin repeat and zinc finger domain-containing protein 1 isoform X2 [Latimeria chalumnae].
XP_006004359.1	ANKZF1	874	eukaryota>metazoa>chordata>vertebrata	Latimeria chalumnae	PREDICTED: ankyrin repeat and zinc finger domain-containing protein 1 isoform X3 [Latimeria chalumnae].
XP_012522959.1	LOC105828927	846	eukaryota>metazoa>hexapoda	Monomorium pharaonis	PREDICTED: ankyrin repeat and zinc finger domain-containing protein 1-like [Monomorium pharaonis].
XP_014161118.1	SARC_00652	721	eukaryota	Sphaeroforma arctica JP610	hypothetical protein, variant [Sphaeroforma arctica JP610].
XP_004348743.1	ACA1_035590	695	eukaryota>amoebozoa>acanthamoebidae	Acanthamoeba castellanii str. Neff	ankyrin repeat and zinc finger domain protein, putative [Acanthamoeba castellanii str. Neff].
XP_012756077.1	SAMD00019534_041180	714	eukaryota>amoebozoa>mycetozoa>dictyosteliida	Acytostelium subglobosum LB1	hypothetical protein SAMD00019534_041180 [Acytostelium subglobosum LB1].
XP_646045.1	DDB_G0270552	787	eukaryota>amoebozoa>mycetozoa>dictyosteliida	Dictyostelium discoideum AX4	hypothetical protein DDB_G0270552 [Dictyostelium discoideum AX4].
XP_004360440.1	DFA_04719	710	eukaryota>amoebozoa>mycetozoa>dictyosteliida	Dictyostelium fasciculatum	hypothetical protein DFA_04719 [Dictyostelium fasciculatum].
EFA75080.1	PPL_11154	704	eukaryota>amoebozoa>mycetozoa>dictyosteliida	Polysphondylium pallidum PN500	hypothetical protein PPL_11154 [Polysphondylium pallidum PN500].
KYQ88554.1	DLAC_11283	691	eukaryota>amoebozoa>mycetozoa>dictyosteliida	Dictyostelium lacteum	hypothetical protein DLAC_11283 [Dictyostelium lacteum].
XP_003288608.1	DICPUDRAFT_79406	750	eukaryota>amoebozoa>mycetozoa>dictyosteliida	Dictyostelium purpureum	hypothetical protein DICPUDRAFT_79406 [Dictyostelium purpureum].
XP_005826124.1	GUITHDRAFT_143749	550	eukaryota>cryptophyta	Guillardia theta CCMP2712	hypothetical protein GUITHDRAFT_143749 [Guillardia theta CCMP2712].
KFH63206.1	MVEG_10617	794	eukaryota>fungi	Mortierella verticillata NRRL 6337	hypothetical protein MVEG_10617 [Mortierella verticillata NRRL 6337].
KXS17237.1	M427DRAFT_133667	693	eukaryota>fungi>chytridiomycota	Gonapodya prolifera JEL478	hypothetical protein M427DRAFT_133667 [Gonapodya prolifera JEL478].
XP_016605920.1	SPPG_06872	612	eukaryota>fungi>chytridiomycota	Spizellomyces punctatus DAOM BR117	hypothetical protein SPPG_06872 [Spizellomyces punctatus DAOM BR117].
OAJ39635.1	BDEG_23468	445	eukaryota>fungi>chytridiomycota	Batrachochytrium dendrobatidis JEL423	hypothetical protein BDEG_23468 [Batrachochytrium dendrobatidis JEL423].
XP_006677057.1	BATDEDRAFT_86433	579	eukaryota>fungi>chytridiomycota	Batrachochytrium dendrobatidis JAM81	hypothetical protein BATDEDRAFT_86433 [Batrachochytrium dendrobatidis JAM81].
ERZ95730.1	GLOINDRAFT_308807	686	eukaryota>fungi>glomeromycota	Rhizophagus irregularis DAOM 181602	hypothetical protein GLOINDRAFT_308807 [Rhizophagus irregularis DAOM 181602].
EXX76148.1	RirG_035850	622	eukaryota>fungi>glomeromycota	Rhizophagus irregularis DAOM 197198w	Vms1p [Rhizophagus irregularis DAOM 197198w].
XP_003384545.1	LOC100638760	692	eukaryota>metazoa	Amphimedon queenslandica	PREDICTED: ankyrin repeat and zinc finger domain-containing protein 1-like [Amphimedon queenslandica].
XP_014667024.1	LOC106808707	729	eukaryota>metazoa	Priapulus caudatus	PREDICTED: ankyrin repeat and zinc finger domain-containing protein 1-like [Priapulus caudatus].
CCD80995.1	Smp_057430	810	eukaryota>metazoa	Schistosoma mansoni	hypothetical protein Smp_057430 [Schistosoma mansoni].
CAX72842.1	-	822	eukaryota>metazoa	Schistosoma japonicum	Ankyrin repeat and zinc finger domain-containing protein 1 [Schistosoma japonicum].
GAA56500.1	CLF_111010	781	eukaryota>metazoa	Clonorchis sinensis	ankyrin repeat and zinc finger domain-containing protein 1 [Clonorchis sinensis].
EUB58031.1	EGR_07137	801	eukaryota>metazoa	Echinococcus granulosus	Ankyrin repeat and zinc finger domain-containing protein [Echinococcus granulosus].
XP_013399111.1	LOC106165449	756	eukaryota>metazoa	Lingula anatina	PREDICTED: ankyrin repeat and zinc finger domain-containing protein 1-like isoform X2 [Lingula anatina].
CDS29323.1	HmN_000228000	758	eukaryota>metazoa	Hymenolepis microstoma	ankyrin repeat and zinc finger domain containing protein [Hymenolepis microstoma].
XP_013416997.1	LOC106178390	773	eukaryota>metazoa	Lingula anatina	PREDICTED: ankyrin repeat and zinc finger domain-containing protein 1-like [Lingula anatina].
XP_013399110.1	LOC106165449	767	eukaryota>metazoa	Lingula anatina	PREDICTED: ankyrin repeat and zinc finger domain-containing protein 1-like isoform X1 [Lingula anatina].
XP_002400030.1	IscW_ISCW000843	597	eukaryota>metazoa	Ixodes scapularis	hypothetical protein IscW_ISCW000843 [Ixodes scapularis].
ELU10697.1	CAPTEDRAFT_99754	581	eukaryota>metazoa>annelida	Capitella teleta	hypothetical protein CAPTEDRAFT_99754 [Capitella teleta].
CBY39090.1	GSOID_T00019634001	584	eukaryota>metazoa>chordata	Oikopleura dioica	unnamed protein product [Oikopleura dioica].
XP_002123606.1	LOC100184375	692	eukaryota>metazoa>chordata	Ciona intestinalis	PREDICTED: ankyrin repeat and zinc finger domain-containing protein 1-like [Ciona intestinalis].
XP_002600691.1	BRAFLDRAFT_118554	798	eukaryota>metazoa>chordata	Branchiostoma floridae	hypothetical protein BRAFLDRAFT_118554 [Branchiostoma floridae].
CBY18552.1	GSOID_T00002419001	584	eukaryota>metazoa>chordata	Oikopleura dioica	unnamed protein product [Oikopleura dioica].
CBY41826.1	GSOID_T00023919001	587	eukaryota>metazoa>chordata	Oikopleura dioica	unnamed protein product, partial [Oikopleura dioica].
XP_006168696.1	ANKZF1	670	eukaryota>metazoa>chordata>vertebrata	Tupaia chinensis	PREDICTED: ankyrin repeat and zinc finger domain-containing protein 1 isoform X2 [Tupaia chinensis].
XP_009442531.1	ANKZF1	670	eukaryota>metazoa>chordata>vertebrata	Pan troglodytes	PREDICTED: ankyrin repeat and zinc finger domain-containing protein 1 isoform X2 [Pan troglodytes].
XP_006059048.1	ANKZF1	671	eukaryota>metazoa>chordata>vertebrata	Bubalus bubalis	PREDICTED: ankyrin repeat and zinc finger domain-containing protein 1 isoform X5 [Bubalus bubalis].
XP_008166069.1	ANKZF1	789	eukaryota>metazoa>chordata>vertebrata	Chrysemys picta bellii	PREDICTED: ankyrin repeat and zinc finger domain-containing protein 1 [Chrysemys picta bellii].
XP_014138427.1	ANKZF1	646	eukaryota>metazoa>chordata>vertebrata	Falco cherrug	PREDICTED: ankyrin repeat and zinc finger domain-containing protein 1 isoform X1 [Falco cherrug].
ELK30539.1	MDA_GLEAN10022951	787	eukaryota>metazoa>chordata>vertebrata	Myotis davidii	Ankyrin repeat and zinc finger domain-containing protein 1 [Myotis davidii].
KFZ55146.1	N321_07121	584	eukaryota>metazoa>chordata>vertebrata	Caprimulgus carolinensis	Ankyrin repeat and zinc finger domain-containing protein 1, partial [Caprimulgus carolinensis].
XP_015841023.1	Ankzf1	596	eukaryota>metazoa>chordata>vertebrata	Peromyscus maniculatus bairdii	PREDICTED: ankyrin repeat and zinc finger domain-containing protein 1 isoform X2 [Peromyscus maniculatus bairdii].
XP_004762883.1	ANKZF1	603	eukaryota>metazoa>chordata>vertebrata	Mustela putorius furo	PREDICTED: ankyrin repeat and zinc finger domain-containing protein 1 isoform X2 [Mustela putorius furo].
XP_011231815.1	ANKZF1	605	eukaryota>metazoa>chordata>vertebrata	Ailuropoda melanoleuca	PREDICTED: ankyrin repeat and zinc finger domain-containing protein 1 isoform X3 [Ailuropoda melanoleuca].
KFR04468.1	N306_14030	659	eukaryota>metazoa>chordata>vertebrata	Opisthocomus hoazin	Ankyrin repeat and zinc finger domain-containing protein 1, partial [Opisthocomus hoazin].
XP_010156845.1	ANKZF1	668	eukaryota>metazoa>chordata>vertebrata	Eurypyga helias	PREDICTED: ankyrin repeat and zinc finger domain-containing protein 1, partial [Eurypyga helias].
XP_008997785.1	ANKZF1	673	eukaryota>metazoa>chordata>vertebrata	Callithrix jacchus	PREDICTED: ankyrin repeat and zinc finger domain-containing protein 1 isoform X2 [Callithrix jacchus].
XP_012301341.1	ANKZF1	778	eukaryota>metazoa>chordata>vertebrata	Aotus nancymaae	PREDICTED: ankyrin repeat and zinc finger domain-containing protein 1 isoform X1 [Aotus nancymaae].
XP_012301342.1	ANKZF1	777	eukaryota>metazoa>chordata>vertebrata	Aotus nancymaae	PREDICTED: ankyrin repeat and zinc finger domain-containing protein 1 isoform X2 [Aotus nancymaae].
XP_017390711.1	ANKZF1	673	eukaryota>metazoa>chordata>vertebrata	Cebus capucinus imitator	PREDICTED: ankyrin repeat and zinc finger domain-containing protein 1 isoform X3 [Cebus capucinus imitator].
XP_009944891.1	ANKZF1	673	eukaryota>metazoa>chordata>vertebrata	Leptosomus discolor	PREDICTED: ankyrin repeat and zinc finger domain-containing protein 1, partial [Leptosomus discolor].
ERE86584.1	H671_2g4401	775	eukaryota>metazoa>chordata>vertebrata	Cricetulus griseus	ankyrin repeat and zinc finger domain-containing protein 1 [Cricetulus griseus].
XP_008688660.1	ANKZF1	605	eukaryota>metazoa>chordata>vertebrata	Ursus maritimus	PREDICTED: ankyrin repeat and zinc finger domain-containing protein 1 isoform X3 [Ursus maritimus].
XP_010009599.1	ANKZF1	674	eukaryota>metazoa>chordata>vertebrata	Nestor notabilis	PREDICTED: ankyrin repeat and zinc finger domain-containing protein 1, partial [Nestor notabilis].
XP_010190828.1	ANKZF1	674	eukaryota>metazoa>chordata>vertebrata	Mesitornis unicolor	PREDICTED: ankyrin repeat and zinc finger domain-containing protein 1, partial [Mesitornis unicolor].
XP_013041959.1	ANKZF1	607	eukaryota>metazoa>chordata>vertebrata	Anser cygnoides domesticus	PREDICTED: ankyrin repeat and zinc finger domain-containing protein 1 [Anser cygnoides domesticus].
XP_004467819.1	ANKZF1	765	eukaryota>metazoa>chordata>vertebrata	Dasypus novemcinctus	PREDICTED: ankyrin repeat and zinc finger domain-containing protein 1 isoform X1 [Dasypus novemcinctus].
XP_009988353.1	ANKZF1	674	eukaryota>metazoa>chordata>vertebrata	Tauraco erythrolophus	PREDICTED: LOW QUALITY PROTEIN: ankyrin repeat and zinc finger domain-containing protein 1, partial [Tauraco erythrolophus].
XP_008688659.1	ANKZF1	675	eukaryota>metazoa>chordata>vertebrata	Ursus maritimus	PREDICTED: ankyrin repeat and zinc finger domain-containing protein 1 isoform X2 [Ursus maritimus].
XP_009868455.1	ANKZF1	615	eukaryota>metazoa>chordata>vertebrata	Apaloderma vittatum	PREDICTED: LOW QUALITY PROTEIN: ankyrin repeat and zinc finger domain-containing protein 1 [Apaloderma vittatum].
XP_007646381.1	Ankzf1	757	eukaryota>metazoa>chordata>vertebrata	Cricetulus griseus	PREDICTED: ankyrin repeat and zinc finger domain-containing protein 1 isoform X3 [Cricetulus griseus].
XP_011231809.1	ANKZF1	675	eukaryota>metazoa>chordata>vertebrata	Ailuropoda melanoleuca	PREDICTED: ankyrin repeat and zinc finger domain-containing protein 1 isoform X2 [Ailuropoda melanoleuca].
XP_010213662.1	ANKZF1	666	eukaryota>metazoa>chordata>vertebrata	Tinamus guttatus	PREDICTED: ankyrin repeat and zinc finger domain-containing protein 1 [Tinamus guttatus].
XP_012430258.1	ANKZF1	579	eukaryota>metazoa>chordata>vertebrata	Taeniopygia guttata	PREDICTED: ankyrin repeat and zinc finger domain-containing protein 1 [Taeniopygia guttata].
XP_014922132.1	ANKZF1	755	eukaryota>metazoa>chordata>vertebrata	Acinonyx jubatus	PREDICTED: ankyrin repeat and zinc finger domain-containing protein 1 [Acinonyx jubatus].
KFZ67158.1	N338_06351	647	eukaryota>metazoa>chordata>vertebrata	Podiceps cristatus	Ankyrin repeat and zinc finger domain-containing protein 1, partial [Podiceps cristatus].
XP_009707805.1	ANKZF1	677	eukaryota>metazoa>chordata>vertebrata	Cariama cristata	PREDICTED: ankyrin repeat and zinc finger domain-containing protein 1, partial [Cariama cristata].
XP_008945247.1	ANKZF1	410	eukaryota>metazoa>chordata>vertebrata	Merops nubicus	PREDICTED: ankyrin repeat and zinc finger domain-containing protein 1, partial [Merops nubicus].
XP_010288812.1	ANKZF1	677	eukaryota>metazoa>chordata>vertebrata	Phaethon lepturus	PREDICTED: ankyrin repeat and zinc finger domain-containing protein 1, partial [Phaethon lepturus].
KQK84457.1	AAES_87839	631	eukaryota>metazoa>chordata>vertebrata	Amazona aestiva	ankyrin repeat and zinc finger domain-containing protein 1 [Amazona aestiva].
XP_015336900.1	Ankzf1	749	eukaryota>metazoa>chordata>vertebrata	Marmota marmota marmota	PREDICTED: ankyrin repeat and zinc finger domain-containing protein 1 isoform X1 [Marmota marmota marmota].
XP_006168697.1	ANKZF1	564	eukaryota>metazoa>chordata>vertebrata	Tupaia chinensis	PREDICTED: ankyrin repeat and zinc finger domain-containing protein 1 isoform X3 [Tupaia chinensis].
EDL75398.1	rCG_23889	748	eukaryota>metazoa>chordata>vertebrata	Rattus norvegicus	ankyrin repeat containing protein RGD1359242, isoform CRA_c [Rattus norvegicus].
Q80UU1.2	-	748	eukaryota>metazoa>chordata>vertebrata	Mus musculus	RecName: Full=Ankyrin repeat and zinc finger domain-containing protein 1.
NP_080463.3	Ankzf1	748	eukaryota>metazoa>chordata>vertebrata	Mus musculus	ankyrin repeat and zinc finger domain-containing protein 1 isoform 1 [Mus musculus].
XP_010117653.1	ANKZF1	678	eukaryota>metazoa>chordata>vertebrata	Chlamydotis macqueenii	PREDICTED: ankyrin repeat and zinc finger domain-containing protein 1, partial [Chlamydotis macqueenii].
OBS60584.1	A6R68_08289	561	eukaryota>metazoa>chordata>vertebrata	Neotoma lepida	hypothetical protein A6R68_08289 [Neotoma lepida].
XP_007909706.1	ankzf1	744	eukaryota>metazoa>chordata>vertebrata	Callorhinchus milii	PREDICTED: ankyrin repeat and zinc finger domain-containing protein 1 [Callorhinchus milii].
XP_016012231.1	ANKZF1	744	eukaryota>metazoa>chordata>vertebrata	Rousettus aegyptiacus	PREDICTED: ankyrin repeat and zinc finger domain-containing protein 1 isoform X1 [Rousettus aegyptiacus].
XP_012401823.1	ANKZF1	561	eukaryota>metazoa>chordata>vertebrata	Sarcophilus harrisii	PREDICTED: ankyrin repeat and zinc finger domain-containing protein 1 isoform X2 [Sarcophilus harrisii].
KFW67668.1	AS28_08297	632	eukaryota>metazoa>chordata>vertebrata	Pygoscelis adeliae	Ankyrin repeat and zinc finger domain-containing protein 1, partial [Pygoscelis adeliae].
KFP51321.1	N323_05126	679	eukaryota>metazoa>chordata>vertebrata	Cathartes aura	Ankyrin repeat and zinc finger domain-containing protein 1, partial [Cathartes aura].
XP_005202879.1	ANKZF1	679	eukaryota>metazoa>chordata>vertebrata	Bos taurus	PREDICTED: ankyrin repeat and zinc finger domain-containing protein 1 isoform X3 [Bos taurus].
ELR60774.1	M91_13952	741	eukaryota>metazoa>chordata>vertebrata	Bos mutus	Ankyrin repeat and zinc finger domain-containing protein 1 [Bos mutus].
XP_011356984.1	ANKZF1	558	eukaryota>metazoa>chordata>vertebrata	Pteropus vampyrus	PREDICTED: ankyrin repeat and zinc finger domain-containing protein 1 [Pteropus vampyrus].
XP_009994570.1	ANKZF1	681	eukaryota>metazoa>chordata>vertebrata	Chaetura pelagica	PREDICTED: ankyrin repeat and zinc finger domain-containing protein 1 [Chaetura pelagica].
XP_009460439.1	ANKZF1	682	eukaryota>metazoa>chordata>vertebrata	Nipponia nippon	PREDICTED: ankyrin repeat and zinc finger domain-containing protein 1 [Nipponia nippon].
KFO20164.1	H920_18450	738	eukaryota>metazoa>chordata>vertebrata	Fukomys damarensis	Ankyrin repeat and zinc finger domain-containing protein 1 [Fukomys damarensis].
XP_015841028.1	Ankzf1	556	eukaryota>metazoa>chordata>vertebrata	Peromyscus maniculatus bairdii	PREDICTED: ankyrin repeat and zinc finger domain-containing protein 1 isoform X3 [Peromyscus maniculatus bairdii].
XP_010138184.1	ANKZF1	555	eukaryota>metazoa>chordata>vertebrata	Buceros rhinoceros silvestris	PREDICTED: ankyrin repeat and zinc finger domain-containing protein 1 [Buceros rhinoceros silvestris].
XP_010800629.1	ANKZF1	737	eukaryota>metazoa>chordata>vertebrata	Bos taurus	PREDICTED: ankyrin repeat and zinc finger domain-containing protein 1 isoform X1 [Bos taurus].
KFQ46920.1	N333_05810	684	eukaryota>metazoa>chordata>vertebrata	Nestor notabilis	Ankyrin repeat and zinc finger domain-containing protein 1, partial [Nestor notabilis].
ELV10672.1	TREES_T100005928	551	eukaryota>metazoa>chordata>vertebrata	Tupaia chinensis	Ankyrin repeat and zinc finger domain-containing protein 1 [Tupaia chinensis].
KFO07440.1	N312_06312	658	eukaryota>metazoa>chordata>vertebrata	Balearica regulorum gibbericeps	Ankyrin repeat and zinc finger domain-containing protein 1, partial [Balearica regulorum gibbericeps].
XP_008626970.1	ANKZF1	685	eukaryota>metazoa>chordata>vertebrata	Corvus brachyrhynchos	PREDICTED: ankyrin repeat and zinc finger domain-containing protein 1 [Corvus brachyrhynchos].
XP_009962325.1	ANKZF1	634	eukaryota>metazoa>chordata>vertebrata	Tyto alba	PREDICTED: LOW QUALITY PROTEIN: ankyrin repeat and zinc finger domain-containing protein 1, partial [Tyto alba].
XP_015288326.1	ANKZF1	546	eukaryota>metazoa>chordata>vertebrata	Macaca fascicularis	PREDICTED: ankyrin repeat and zinc finger domain-containing protein 1 isoform X1 [Macaca fascicularis].
XP_014966604.1	ANKZF1	546	eukaryota>metazoa>chordata>vertebrata	Macaca mulatta	PREDICTED: ankyrin repeat and zinc finger domain-containing protein 1 isoform X1 [Macaca mulatta].
XP_009674417.1	ANKZF1	687	eukaryota>metazoa>chordata>vertebrata	Struthio camelus australis	PREDICTED: ankyrin repeat and zinc finger domain-containing protein 1 isoform X2 [Struthio camelus australis].
KFW11657.1	N326_08036	688	eukaryota>metazoa>chordata>vertebrata	Eurypyga helias	Ankyrin repeat and zinc finger domain-containing protein 1, partial [Eurypyga helias].
ETE70721.1	L345_03452	688	eukaryota>metazoa>chordata>vertebrata	Ophiophagus hannah	Ankyrin repeat and zinc finger domain-containing protein 1 [Ophiophagus hannah].
KFQ06619.1	N330_07653	689	eukaryota>metazoa>chordata>vertebrata	Leptosomus discolor	Ankyrin repeat and zinc finger domain-containing protein 1, partial [Leptosomus discolor].
XP_014459981.1	ANKZF1	538	eukaryota>metazoa>chordata>vertebrata	Alligator mississippiensis	PREDICTED: ankyrin repeat and zinc finger domain-containing protein 1 [Alligator mississippiensis].
XP_006004360.1	ANKZF1	733	eukaryota>metazoa>chordata>vertebrata	Latimeria chalumnae	PREDICTED: ankyrin repeat and zinc finger domain-containing protein 1 isoform X4 [Latimeria chalumnae].
XP_005672286.1	ANKZF1	733	eukaryota>metazoa>chordata>vertebrata	Sus scrofa	PREDICTED: ankyrin repeat and zinc finger domain-containing protein 1 isoform X1 [Sus scrofa].
KFQ78342.1	N335_06424	689	eukaryota>metazoa>chordata>vertebrata	Phaethon lepturus	Ankyrin repeat and zinc finger domain-containing protein 1, partial [Phaethon lepturus].
XP_015672180.1	ANKZF1	689	eukaryota>metazoa>chordata>vertebrata	Protobothrops mucrosquamatus	PREDICTED: ankyrin repeat and zinc finger domain-containing protein 1 [Protobothrops mucrosquamatus].
XP_012418672.1	ANKZF1	732	eukaryota>metazoa>chordata>vertebrata	Odobenus rosmarus divergens	PREDICTED: ankyrin repeat and zinc finger domain-containing protein 1 isoform X1 [Odobenus rosmarus divergens].
XP_012956030.1	LOC101804890	519	eukaryota>metazoa>chordata>vertebrata	Anas platyrhynchos	PREDICTED: LOW QUALITY PROTEIN: ankyrin repeat and zinc finger domain-containing protein 1-like [Anas platyrhynchos].
XP_005672287.1	ANKZF1	731	eukaryota>metazoa>chordata>vertebrata	Sus scrofa	PREDICTED: ankyrin repeat and zinc finger domain-containing protein 1 isoform X2 [Sus scrofa].
XP_007964546.1	ANKZF1	730	eukaryota>metazoa>chordata>vertebrata	Chlorocebus sabaeus	PREDICTED: ankyrin repeat and zinc finger domain-containing protein 1 isoform X4 [Chlorocebus sabaeus].
XP_010986669.1	ANKZF1	690	eukaryota>metazoa>chordata>vertebrata	Camelus dromedarius	PREDICTED: LOW QUALITY PROTEIN: ankyrin repeat and zinc finger domain-containing protein 1 [Camelus dromedarius].
XP_010307777.1	ANKZF1	518	eukaryota>metazoa>chordata>vertebrata	Balearica regulorum gibbericeps	PREDICTED: ankyrin repeat and zinc finger domain-containing protein 1 [Balearica regulorum gibbericeps].
XP_009943606.1	ANKZF1	661	eukaryota>metazoa>chordata>vertebrata	Opisthocomus hoazin	PREDICTED: ankyrin repeat and zinc finger domain-containing protein 1 [Opisthocomus hoazin].
XP_009813090.1	ANKZF1	649	eukaryota>metazoa>chordata>vertebrata	Gavia stellata	PREDICTED: LOW QUALITY PROTEIN: ankyrin repeat and zinc finger domain-containing protein 1, partial [Gavia stellata].
XP_005889741.1	ANKZF1	728	eukaryota>metazoa>chordata>vertebrata	Bos mutus	PREDICTED: ankyrin repeat and zinc finger domain-containing protein 1 [Bos mutus].
NP_001019668.2	ANKZF1	728	eukaryota>metazoa>chordata>vertebrata	Bos taurus	ankyrin repeat and zinc finger domain-containing protein 1 [Bos taurus].
XP_006059044.1	ANKZF1	728	eukaryota>metazoa>chordata>vertebrata	Bubalus bubalis	PREDICTED: ankyrin repeat and zinc finger domain-containing protein 1 isoform X1 [Bubalus bubalis].
XP_005985201.1	ANKZF1	728	eukaryota>metazoa>chordata>vertebrata	Pantholops hodgsonii	PREDICTED: ankyrin repeat and zinc finger domain-containing protein 1 isoform X1 [Pantholops hodgsonii].
EHA97561.1	GW7_07281	728	eukaryota>metazoa>chordata>vertebrata	Heterocephalus glaber	Ankyrin repeat and zinc finger domain-containing protein 1 [Heterocephalus glaber].
EAW70708.1	hCG_15582	728	eukaryota>metazoa>chordata>vertebrata	Homo sapiens	ankyrin repeat and zinc finger domain containing 1, isoform CRA_a [Homo sapiens].
EPQ18454.1	D623_10032932	727	eukaryota>metazoa>chordata>vertebrata	Myotis brandtii	Ankyrin repeat and zinc finger domain-containing protein 1 [Myotis brandtii].
KFQ83272.1	N337_07917	644	eukaryota>metazoa>chordata>vertebrata	Phoenicopterus ruber ruber	Ankyrin repeat and zinc finger domain-containing protein 1, partial [Phoenicopterus ruber ruber].
XP_008143365.1	ANKZF1	727	eukaryota>metazoa>chordata>vertebrata	Eptesicus fuscus	PREDICTED: ankyrin repeat and zinc finger domain-containing protein 1 isoform X1 [Eptesicus fuscus].
XP_004662967.1	Ankzf1	727	eukaryota>metazoa>chordata>vertebrata	Jaculus jaculus	PREDICTED: ankyrin repeat and zinc finger domain-containing protein 1 isoform X1 [Jaculus jaculus].
XP_005001104.1	Ankzf1	727	eukaryota>metazoa>chordata>vertebrata	Cavia porcellus	PREDICTED: ankyrin repeat and zinc finger domain-containing protein 1 [Cavia porcellus].
XP_010358595.1	ANKZF1	726	eukaryota>metazoa>chordata>vertebrata	Rhinopithecus roxellana	PREDICTED: ankyrin repeat and zinc finger domain-containing protein 1 [Rhinopithecus roxellana].
XP_010610142.1	Ankzf1	726	eukaryota>metazoa>chordata>vertebrata	Fukomys damarensis	PREDICTED: ankyrin repeat and zinc finger domain-containing protein 1 isoform X1 [Fukomys damarensis].
XP_011739035.1	ANKZF1	726	eukaryota>metazoa>chordata>vertebrata	Macaca nemestrina	PREDICTED: ankyrin repeat and zinc finger domain-containing protein 1 isoform X1 [Macaca nemestrina].
XP_011787593.1	ANKZF1	726	eukaryota>metazoa>chordata>vertebrata	Colobus angolensis palliatus	PREDICTED: ankyrin repeat and zinc finger domain-containing protein 1 isoform X1 [Colobus angolensis palliatus].
EHH21693.1	EGK_04817	726	eukaryota>metazoa>chordata>vertebrata	Macaca mulatta	hypothetical protein EGK_04817 [Macaca mulatta].
XP_014966607.1	ANKZF1	726	eukaryota>metazoa>chordata>vertebrata	Macaca mulatta	PREDICTED: ankyrin repeat and zinc finger domain-containing protein 1 isoform X2 [Macaca mulatta].
XP_004864596.1	Ankzf1	726	eukaryota>metazoa>chordata>vertebrata	Heterocephalus glaber	PREDICTED: ankyrin repeat and zinc finger domain-containing protein 1 [Heterocephalus glaber].
NP_060559.2	ANKZF1	726	eukaryota>metazoa>chordata>vertebrata	Homo sapiens	ankyrin repeat and zinc finger domain-containing protein 1 isoform 1 [Homo sapiens].
XP_012497978.1	ANKZF1	726	eukaryota>metazoa>chordata>vertebrata	Propithecus coquereli	PREDICTED: ankyrin repeat and zinc finger domain-containing protein 1 isoform X1 [Propithecus coquereli].
XP_002812922.1	ANKZF1	726	eukaryota>metazoa>chordata>vertebrata	Pongo abelii	PREDICTED: ankyrin repeat and zinc finger domain-containing protein 1 isoform X1 [Pongo abelii].
EHH55183.1	EGM_04335	726	eukaryota>metazoa>chordata>vertebrata	Macaca fascicularis	hypothetical protein EGM_04335 [Macaca fascicularis].
XP_005574412.1	ANKZF1	726	eukaryota>metazoa>chordata>vertebrata	Macaca fascicularis	PREDICTED: ankyrin repeat and zinc finger domain-containing protein 1 isoform X2 [Macaca fascicularis].
XP_007964547.1	ANKZF1	726	eukaryota>metazoa>chordata>vertebrata	Chlorocebus sabaeus	PREDICTED: ankyrin repeat and zinc finger domain-containing protein 1 isoform X5 [Chlorocebus sabaeus].
XP_003908030.1	ANKZF1	726	eukaryota>metazoa>chordata>vertebrata	Papio anubis	PREDICTED: ankyrin repeat and zinc finger domain-containing protein 1 isoform X1 [Papio anubis].
XP_011918214.1	ANKZF1	726	eukaryota>metazoa>chordata>vertebrata	Cercocebus atys	PREDICTED: ankyrin repeat and zinc finger domain-containing protein 1 isoform X1 [Cercocebus atys].
XP_007445881.1	ANKZF1	726	eukaryota>metazoa>chordata>vertebrata	Lipotes vexillifer	PREDICTED: LOW QUALITY PROTEIN: ankyrin repeat and zinc finger domain-containing protein 1 [Lipotes vexillifer].
XP_009442532.1	ANKZF1	661	eukaryota>metazoa>chordata>vertebrata	Pan troglodytes	PREDICTED: ankyrin repeat and zinc finger domain-containing protein 1 isoform X3 [Pan troglodytes].
XP_016012233.1	ANKZF1	743	eukaryota>metazoa>chordata>vertebrata	Rousettus aegyptiacus	PREDICTED: ankyrin repeat and zinc finger domain-containing protein 1 isoform X2 [Rousettus aegyptiacus].
NP_001267298.1	ANKZF1	726	eukaryota>metazoa>chordata>vertebrata	Pan troglodytes	ankyrin repeat and zinc finger domain-containing protein 1 [Pan troglodytes].
XP_003818694.1	ANKZF1	726	eukaryota>metazoa>chordata>vertebrata	Pan paniscus	PREDICTED: ankyrin repeat and zinc finger domain-containing protein 1 isoform X1 [Pan paniscus].
XP_011820035.1	ANKZF1	726	eukaryota>metazoa>chordata>vertebrata	Mandrillus leucophaeus	PREDICTED: ankyrin repeat and zinc finger domain-containing protein 1 isoform X1 [Mandrillus leucophaeus].
XP_005330555.1	Ankzf1	725	eukaryota>metazoa>chordata>vertebrata	Ictidomys tridecemlineatus	PREDICTED: ankyrin repeat and zinc finger domain-containing protein 1 [Ictidomys tridecemlineatus].
KFQ94699.1	Y956_16282	693	eukaryota>metazoa>chordata>vertebrata	Nipponia nippon	Ankyrin repeat and zinc finger domain-containing protein 1, partial [Nipponia nippon].
XP_008970249.1	ANKZF1	725	eukaryota>metazoa>chordata>vertebrata	Pan paniscus	PREDICTED: ankyrin repeat and zinc finger domain-containing protein 1 isoform X2 [Pan paniscus].
XP_012595647.1	ANKZF1	725	eukaryota>metazoa>chordata>vertebrata	Microcebus murinus	PREDICTED: ankyrin repeat and zinc finger domain-containing protein 1 isoform X1 [Microcebus murinus].
XP_009442530.1	ANKZF1	725	eukaryota>metazoa>chordata>vertebrata	Pan troglodytes	PREDICTED: ankyrin repeat and zinc finger domain-containing protein 1 isoform X1 [Pan troglodytes].
XP_010610145.1	Ankzf1	725	eukaryota>metazoa>chordata>vertebrata	Fukomys damarensis	PREDICTED: ankyrin repeat and zinc finger domain-containing protein 1 isoform X2 [Fukomys damarensis].
XP_011509694.1	ANKZF1	725	eukaryota>metazoa>chordata>vertebrata	Homo sapiens	PREDICTED: ankyrin repeat and zinc finger domain-containing protein 1 isoform X1 [Homo sapiens].
XP_011739037.1	ANKZF1	725	eukaryota>metazoa>chordata>vertebrata	Macaca nemestrina	PREDICTED: ankyrin repeat and zinc finger domain-containing protein 1 isoform X2 [Macaca nemestrina].
XP_003272446.2	ANKZF1	725	eukaryota>metazoa>chordata>vertebrata	Nomascus leucogenys	PREDICTED: ankyrin repeat and zinc finger domain-containing protein 1 [Nomascus leucogenys].
XP_003406101.1	ANKZF1	724	eukaryota>metazoa>chordata>vertebrata	Loxodonta africana	PREDICTED: ankyrin repeat and zinc finger domain-containing protein 1 isoform X1 [Loxodonta africana].
XP_011231803.1	ANKZF1	724	eukaryota>metazoa>chordata>vertebrata	Ailuropoda melanoleuca	PREDICTED: ankyrin repeat and zinc finger domain-containing protein 1 isoform X1 [Ailuropoda melanoleuca].
XP_008688662.1	ANKZF1	724	eukaryota>metazoa>chordata>vertebrata	Ursus maritimus	PREDICTED: ankyrin repeat and zinc finger domain-containing protein 1 isoform X5 [Ursus maritimus].
XP_009323130.1	ANKZF1	517	eukaryota>metazoa>chordata>vertebrata	Pygoscelis adeliae	PREDICTED: LOW QUALITY PROTEIN: ankyrin repeat and zinc finger domain-containing protein 1 [Pygoscelis adeliae].
XP_004402799.1	ANKZF1	724	eukaryota>metazoa>chordata>vertebrata	Odobenus rosmarus divergens	PREDICTED: ankyrin repeat and zinc finger domain-containing protein 1 isoform X2 [Odobenus rosmarus divergens].
XP_010076727.1	ANKZF1	516	eukaryota>metazoa>chordata>vertebrata	Pterocles gutturalis	PREDICTED: ankyrin repeat and zinc finger domain-containing protein 1 [Pterocles gutturalis].
XP_017390709.1	ANKZF1	724	eukaryota>metazoa>chordata>vertebrata	Cebus capucinus imitator	PREDICTED: ankyrin repeat and zinc finger domain-containing protein 1 isoform X1 [Cebus capucinus imitator].
XP_006867892.1	ANKZF1	724	eukaryota>metazoa>chordata>vertebrata	Chrysochloris asiatica	PREDICTED: ankyrin repeat and zinc finger domain-containing protein 1 [Chrysochloris asiatica].
XP_006906759.1	ANKZF1	724	eukaryota>metazoa>chordata>vertebrata	Pteropus alecto	PREDICTED: ankyrin repeat and zinc finger domain-containing protein 1 isoform X1 [Pteropus alecto].
XP_002749856.1	ANKZF1	724	eukaryota>metazoa>chordata>vertebrata	Callithrix jacchus	PREDICTED: ankyrin repeat and zinc finger domain-containing protein 1 isoform X1 [Callithrix jacchus].
XP_016012234.1	ANKZF1	724	eukaryota>metazoa>chordata>vertebrata	Rousettus aegyptiacus	PREDICTED: ankyrin repeat and zinc finger domain-containing protein 1 isoform X3 [Rousettus aegyptiacus].
XP_003925555.1	ANKZF1	724	eukaryota>metazoa>chordata>vertebrata	Saimiri boliviensis boliviensis	PREDICTED: ankyrin repeat and zinc finger domain-containing protein 1 isoform X1 [Saimiri boliviensis boliviensis].
XP_007957233.1	ANKZF1	724	eukaryota>metazoa>chordata>vertebrata	Orycteropus afer afer	PREDICTED: ankyrin repeat and zinc finger domain-containing protein 1 [Orycteropus afer afer].
XP_006742952.1	ANKZF1	724	eukaryota>metazoa>chordata>vertebrata	Leptonychotes weddellii	PREDICTED: ankyrin repeat and zinc finger domain-containing protein 1 [Leptonychotes weddellii].
XP_004427640.1	LOC101393544	724	eukaryota>metazoa>chordata>vertebrata	Ceratotherium simum simum	PREDICTED: ankyrin repeat and zinc finger domain-containing protein 1 [Ceratotherium simum simum].
XP_009555796.1	ANKZF1	724	eukaryota>metazoa>chordata>vertebrata	Cuculus canorus	PREDICTED: ankyrin repeat and zinc finger domain-containing protein 1 [Cuculus canorus].
XP_008688658.1	ANKZF1	724	eukaryota>metazoa>chordata>vertebrata	Ursus maritimus	PREDICTED: ankyrin repeat and zinc finger domain-containing protein 1 isoform X1 [Ursus maritimus].
XP_009477351.1	ANKZF1	513	eukaryota>metazoa>chordata>vertebrata	Pelecanus crispus	PREDICTED: ankyrin repeat and zinc finger domain-containing protein 1 [Pelecanus crispus].
XP_007646382.1	Ankzf1	723	eukaryota>metazoa>chordata>vertebrata	Cricetulus griseus	PREDICTED: ankyrin repeat and zinc finger domain-containing protein 1 isoform X4 [Cricetulus griseus].
XP_009913850.1	ANKZF1	439	eukaryota>metazoa>chordata>vertebrata	Haliaeetus albicilla	PREDICTED: ankyrin repeat and zinc finger domain-containing protein 1, partial [Haliaeetus albicilla].
XP_015724460.1	ANKZF1	723	eukaryota>metazoa>chordata>vertebrata	Coturnix japonica	PREDICTED: ankyrin repeat and zinc finger domain-containing protein 1 isoform X4 [Coturnix japonica].
XP_016052325.1	ANKZF1	723	eukaryota>metazoa>chordata>vertebrata	Miniopterus natalensis	PREDICTED: ankyrin repeat and zinc finger domain-containing protein 1 [Miniopterus natalensis].
XP_004376392.1	LOC101350199	723	eukaryota>metazoa>chordata>vertebrata	Trichechus manatus latirostris	PREDICTED: ankyrin repeat and zinc finger domain-containing protein 1 [Trichechus manatus latirostris].
XP_015440885.1	ANKZF1	723	eukaryota>metazoa>chordata>vertebrata	Pteropus alecto	PREDICTED: ankyrin repeat and zinc finger domain-containing protein 1 isoform X2 [Pteropus alecto].
XP_008823978.1	Ankzf1	723	eukaryota>metazoa>chordata>vertebrata	Nannospalax galili	PREDICTED: ankyrin repeat and zinc finger domain-containing protein 1 [Nannospalax galili].
XP_016804835.1	ANKZF1	654	eukaryota>metazoa>chordata>vertebrata	Pan troglodytes	PREDICTED: ankyrin repeat and zinc finger domain-containing protein 1 isoform X4 [Pan troglodytes].
KFV81945.1	N308_12640	723	eukaryota>metazoa>chordata>vertebrata	Struthio camelus australis	Ankyrin repeat and zinc finger domain-containing protein 1, partial [Struthio camelus australis].
XP_017390710.1	ANKZF1	723	eukaryota>metazoa>chordata>vertebrata	Cebus capucinus imitator	PREDICTED: ankyrin repeat and zinc finger domain-containing protein 1 isoform X2 [Cebus capucinus imitator].
XP_006035441.1	ANKZF1	498	eukaryota>metazoa>chordata>vertebrata	Alligator sinensis	PREDICTED: ankyrin repeat and zinc finger domain-containing protein 1 [Alligator sinensis].
XP_004662968.1	Ankzf1	723	eukaryota>metazoa>chordata>vertebrata	Jaculus jaculus	PREDICTED: ankyrin repeat and zinc finger domain-containing protein 1 isoform X2 [Jaculus jaculus].
XP_008528192.1	ANKZF1	723	eukaryota>metazoa>chordata>vertebrata	Equus przewalskii	PREDICTED: ankyrin repeat and zinc finger domain-containing protein 1 isoform X1 [Equus przewalskii].
XP_001493185.1	ANKZF1	723	eukaryota>metazoa>chordata>vertebrata	Equus caballus	PREDICTED: ankyrin repeat and zinc finger domain-containing protein 1 isoform X1 [Equus caballus].
KFP76662.1	N310_05081	722	eukaryota>metazoa>chordata>vertebrata	Acanthisitta chloris	Ankyrin repeat and zinc finger domain-containing protein 1, partial [Acanthisitta chloris].
XP_006168695.1	ANKZF1	722	eukaryota>metazoa>chordata>vertebrata	Tupaia chinensis	PREDICTED: ankyrin repeat and zinc finger domain-containing protein 1 isoform X1 [Tupaia chinensis].
XP_004467824.1	ANKZF1	722	eukaryota>metazoa>chordata>vertebrata	Dasypus novemcinctus	PREDICTED: ankyrin repeat and zinc finger domain-containing protein 1 isoform X2 [Dasypus novemcinctus].
XP_015742758.1	LOC103057704	493	eukaryota>metazoa>chordata>vertebrata	Python bivittatus	PREDICTED: ankyrin repeat and zinc finger domain-containing protein 1 isoform X2 [Python bivittatus].
XP_015724457.1	ANKZF1	722	eukaryota>metazoa>chordata>vertebrata	Coturnix japonica	PREDICTED: ankyrin repeat and zinc finger domain-containing protein 1 isoform X1 [Coturnix japonica].
KGL86420.1	N301_00309	722	eukaryota>metazoa>chordata>vertebrata	Charadrius vociferus	Ankyrin repeat and zinc finger domain-containing protein 1, partial [Charadrius vociferus].
XP_012418673.1	ANKZF1	722	eukaryota>metazoa>chordata>vertebrata	Odobenus rosmarus divergens	PREDICTED: ankyrin repeat and zinc finger domain-containing protein 1 isoform X3 [Odobenus rosmarus divergens].
XP_012657634.1	ANKZF1	721	eukaryota>metazoa>chordata>vertebrata	Otolemur garnettii	PREDICTED: ankyrin repeat and zinc finger domain-containing protein 1 isoform X3 [Otolemur garnettii].
XP_004576869.1	ANKZF1	721	eukaryota>metazoa>chordata>vertebrata	Ochotona princeps	PREDICTED: LOW QUALITY PROTEIN: ankyrin repeat and zinc finger domain-containing protein 1 [Ochotona princeps].
XP_008585683.1	ANKZF1	721	eukaryota>metazoa>chordata>vertebrata	Galeopterus variegatus	PREDICTED: ankyrin repeat and zinc finger domain-containing protein 1 [Galeopterus variegatus].
KYO17215.1	Y1Q_0013972	488	eukaryota>metazoa>chordata>vertebrata	Alligator mississippiensis	ankyrin repeat and zinc finger domain-containing protein 1 [Alligator mississippiensis].
XP_005640745.1	ANKZF1	721	eukaryota>metazoa>chordata>vertebrata	Canis lupus familiaris	PREDICTED: ankyrin repeat and zinc finger domain-containing protein 1 [Canis lupus familiaris].
XP_006496570.1	Ankzf1	814	eukaryota>metazoa>chordata>vertebrata	Mus musculus	PREDICTED: ankyrin repeat and zinc finger domain-containing protein 1 isoform X1 [Mus musculus].
XP_006890308.1	ANKZF1	721	eukaryota>metazoa>chordata>vertebrata	Elephantulus edwardii	PREDICTED: ankyrin repeat and zinc finger domain-containing protein 1 [Elephantulus edwardii].
XP_010162946.1	ANKZF1	463	eukaryota>metazoa>chordata>vertebrata	Caprimulgus carolinensis	PREDICTED: ankyrin repeat and zinc finger domain-containing protein 1 [Caprimulgus carolinensis].

XP_001232760.2	ANKZF1	720	eukaryota>metazoa>chordata>vertebrata	Gallus gallus	PREDICTED: ankyrin repeat and zinc finger domain-containing protein 1 isoform X1 [Gallus gallus].
XP_013816089.1	ANKZF1	720	eukaryota>metazoa>chordata>vertebrata	Apteryx australis mantelli	PREDICTED: ankyrin repeat and zinc finger domain-containing protein 1 isoform X1 [Apteryx australis mantelli].
XP_011969031.1	ANKZF1	720	eukaryota>metazoa>chordata>vertebrata	Ovis aries musimon	PREDICTED: ankyrin repeat and zinc finger domain-containing protein 1 [Ovis aries musimon].
XP_010859540.1	ANKZF1	720	eukaryota>metazoa>chordata>vertebrata	Bison bison bison	PREDICTED: ankyrin repeat and zinc finger domain-containing protein 1 [Bison bison bison].
XP_006059047.1	ANKZF1	720	eukaryota>metazoa>chordata>vertebrata	Bubalus bubalis	PREDICTED: ankyrin repeat and zinc finger domain-containing protein 1 isoform X4 [Bubalus bubalis].
KFV96856.1	N327_03658	649	eukaryota>metazoa>chordata>vertebrata	Fulmarus glacialis	Ankyrin repeat and zinc finger domain-containing protein 1, partial [Fulmarus glacialis].
XP_014809565.1	LOC106895007	720	eukaryota>metazoa>chordata>vertebrata	Calidris pugnax	PREDICTED: ankyrin repeat and zinc finger domain-containing protein 1-like isoform X1 [Calidris pugnax].
KFV63392.1	N307_04363	695	eukaryota>metazoa>chordata>vertebrata	Picoides pubescens	Ankyrin repeat and zinc finger domain-containing protein 1 [Picoides pubescens].
KFW80141.1	N305_07386	720	eukaryota>metazoa>chordata>vertebrata	Manacus vitellinus	Ankyrin repeat and zinc finger domain-containing protein 1, partial [Manacus vitellinus].
XP_005676637.1	ANKZF1	720	eukaryota>metazoa>chordata>vertebrata	Capra hircus	PREDICTED: ankyrin repeat and zinc finger domain-containing protein 1 [Capra hircus].
KGL77735.1	N309_05888	720	eukaryota>metazoa>chordata>vertebrata	Tinamus guttatus	Ankyrin repeat and zinc finger domain-containing protein 1, partial [Tinamus guttatus].
XP_004762880.1	ANKZF1	720	eukaryota>metazoa>chordata>vertebrata	Mustela putorius furo	PREDICTED: ankyrin repeat and zinc finger domain-containing protein 1 isoform X1 [Mustela putorius furo].
XP_005498745.1	ANKZF1	720	eukaryota>metazoa>chordata>vertebrata	Columba livia	PREDICTED: ankyrin repeat and zinc finger domain-containing protein 1 [Columba livia].
KFV11151.1	N339_12939	651	eukaryota>metazoa>chordata>vertebrata	Pterocles gutturalis	Ankyrin repeat and zinc finger domain-containing protein 1, partial [Pterocles gutturalis].
XP_008143366.1	ANKZF1	719	eukaryota>metazoa>chordata>vertebrata	Eptesicus fuscus	PREDICTED: ankyrin repeat and zinc finger domain-containing protein 1 isoform X2 [Eptesicus fuscus].
XP_005883523.1	ANKZF1	719	eukaryota>metazoa>chordata>vertebrata	Myotis brandtii	PREDICTED: ankyrin repeat and zinc finger domain-containing protein 1 [Myotis brandtii].
XP_012657635.1	ANKZF1	719	eukaryota>metazoa>chordata>vertebrata	Otolemur garnettii	PREDICTED: ankyrin repeat and zinc finger domain-containing protein 1 isoform X4 [Otolemur garnettii].
XP_015419125.1	ANKZF1	719	eukaryota>metazoa>chordata>vertebrata	Myotis davidii	PREDICTED: ankyrin repeat and zinc finger domain-containing protein 1 isoform X1 [Myotis davidii].
XP_006082611.1	ANKZF1	719	eukaryota>metazoa>chordata>vertebrata	Myotis lucifugus	PREDICTED: ankyrin repeat and zinc finger domain-containing protein 1 [Myotis lucifugus].
XP_005361651.1	Ankzf1	719	eukaryota>metazoa>chordata>vertebrata	Microtus ochrogaster	PREDICTED: ankyrin repeat and zinc finger domain-containing protein 1 [Microtus ochrogaster].
XP_004638250.1	Ankzf1	719	eukaryota>metazoa>chordata>vertebrata	Octodon degus	PREDICTED: ankyrin repeat and zinc finger domain-containing protein 1 isoform X1 [Octodon degus].
XP_015742743.1	LOC103057704	461	eukaryota>metazoa>chordata>vertebrata	Python bivittatus	PREDICTED: ankyrin repeat and zinc finger domain-containing protein 1 isoform X1 [Python bivittatus].
XP_004674325.1	ANKZF1	719	eukaryota>metazoa>chordata>vertebrata	Condylura cristata	PREDICTED: ankyrin repeat and zinc finger domain-containing protein 1 isoform X1 [Condylura cristata].
XP_012859868.1	ANKZF1	719	eukaryota>metazoa>chordata>vertebrata	Echinops telfairi	PREDICTED: LOW QUALITY PROTEIN: ankyrin repeat and zinc finger domain-containing protein 1 [Echinops telfairi].
XP_005154149.1	LOC101873265	718	eukaryota>metazoa>chordata>vertebrata	Melopsittacus undulatus	PREDICTED: ankyrin repeat and zinc finger domain-containing protein 1 [Melopsittacus undulatus].
KFP03302.1	N300_03553	718	eukaryota>metazoa>chordata>vertebrata	Calypte anna	Ankyrin repeat and zinc finger domain-containing protein 1, partial [Calypte anna].
XP_010566285.1	ANKZF1	718	eukaryota>metazoa>chordata>vertebrata	Haliaeetus leucocephalus	PREDICTED: ankyrin repeat and zinc finger domain-containing protein 1 isoform X1 [Haliaeetus leucocephalus].
XP_014711573.1	ANKZF1	718	eukaryota>metazoa>chordata>vertebrata	Equus asinus	PREDICTED: ankyrin repeat and zinc finger domain-containing protein 1 isoform X1 [Equus asinus].
XP_007520624.1	ANKZF1	718	eukaryota>metazoa>chordata>vertebrata	Erinaceus europaeus	PREDICTED: ankyrin repeat and zinc finger domain-containing protein 1 [Erinaceus europaeus].
XP_003991219.1	ANKZF1	718	eukaryota>metazoa>chordata>vertebrata	Felis catus	PREDICTED: ankyrin repeat and zinc finger domain-containing protein 1 [Felis catus].
XP_011578794.1	ANKZF1	718	eukaryota>metazoa>chordata>vertebrata	Aquila chrysaetos canadensis	PREDICTED: ankyrin repeat and zinc finger domain-containing protein 1 isoform X1 [Aquila chrysaetos canadensis].
XP_009882399.1	ANKZF1	717	eukaryota>metazoa>chordata>vertebrata	Charadrius vociferus	PREDICTED: ankyrin repeat and zinc finger domain-containing protein 1 [Charadrius vociferus].
XP_005234380.1	ANKZF1	717	eukaryota>metazoa>chordata>vertebrata	Falco peregrinus	PREDICTED: ankyrin repeat and zinc finger domain-containing protein 1 isoform X1 [Falco peregrinus].
XP_004638251.1	Ankzf1	717	eukaryota>metazoa>chordata>vertebrata	Octodon degus	PREDICTED: ankyrin repeat and zinc finger domain-containing protein 1 isoform X2 [Octodon degus].
XP_007086225.1	ANKZF1	717	eukaryota>metazoa>chordata>vertebrata	Panthera tigris altaica	PREDICTED: ankyrin repeat and zinc finger domain-containing protein 1 [Panthera tigris altaica].
KFQ59419.1	N334_06936	696	eukaryota>metazoa>chordata>vertebrata	Pelecanus crispus	Ankyrin repeat and zinc finger domain-containing protein 1, partial [Pelecanus crispus].
XP_009273291.1	ANKZF1	715	eukaryota>metazoa>chordata>vertebrata	Aptenodytes forsteri	PREDICTED: LOW QUALITY PROTEIN: ankyrin repeat and zinc finger domain-containing protein 1 [Aptenodytes forsteri].
XP_007188030.1	ANKZF1	714	eukaryota>metazoa>chordata>vertebrata	Balaenoptera acutorostrata scammoni	PREDICTED: LOW QUALITY PROTEIN: ankyrin repeat and zinc finger domain-containing protein 1 [Balaenoptera acutorostrata scammoni
ERE86583.1	H671_2g4401	714	eukaryota>metazoa>chordata>vertebrata	Cricetulus griseus	ankyrin repeat and zinc finger domain-containing protein 1 [Cricetulus griseus].
XP_009069278.1	ANKZF1	714	eukaryota>metazoa>chordata>vertebrata	Acanthisitta chloris	PREDICTED: ankyrin repeat and zinc finger domain-containing protein 1 [Acanthisitta chloris].
KFO92069.1	N320_06086	643	eukaryota>metazoa>chordata>vertebrata	Buceros rhinoceros silvestris	Ankyrin repeat and zinc finger domain-containing protein 1, partial [Buceros rhinoceros silvestris].
XP_010405223.1	ANKZF1	714	eukaryota>metazoa>chordata>vertebrata	Corvus cornix cornix	PREDICTED: ankyrin repeat and zinc finger domain-containing protein 1 isoform X1 [Corvus cornix cornix].
XP_012001996.1	ANKZF1	714	eukaryota>metazoa>chordata>vertebrata	Ovis aries	PREDICTED: LOW QUALITY PROTEIN: ankyrin repeat and zinc finger domain-containing protein 1 [Ovis aries].
XP_006972224.1	Ankzf1	713	eukaryota>metazoa>chordata>vertebrata	Peromyscus maniculatus bairdii	PREDICTED: ankyrin repeat and zinc finger domain-containing protein 1 isoform X1 [Peromyscus maniculatus bairdii].
XP_008495253.1	ANKZF1	713	eukaryota>metazoa>chordata>vertebrata	Calypte anna	PREDICTED: ankyrin repeat and zinc finger domain-containing protein 1 [Calypte anna].
XP_012790122.1	ANKZF1	713	eukaryota>metazoa>chordata>vertebrata	Sorex araneus	PREDICTED: ankyrin repeat and zinc finger domain-containing protein 1 [Sorex araneus].
XP_013816090.1	ANKZF1	712	eukaryota>metazoa>chordata>vertebrata	Apteryx australis mantelli	PREDICTED: ankyrin repeat and zinc finger domain-containing protein 1 isoform X2 [Apteryx australis mantelli].
XP_005519773.1	ANKZF1	712	eukaryota>metazoa>chordata>vertebrata	Pseudopodoces humilis	PREDICTED: LOW QUALITY PROTEIN: ankyrin repeat and zinc finger domain-containing protein 1 [Pseudopodoces humilis].
XP_013839974.1	ANKZF1	712	eukaryota>metazoa>chordata>vertebrata	Sus scrofa	PREDICTED: ankyrin repeat and zinc finger domain-containing protein 1 isoform X4 [Sus scrofa].
XP_014809566.1	LOC106895007	712	eukaryota>metazoa>chordata>vertebrata	Calidris pugnax	PREDICTED: ankyrin repeat and zinc finger domain-containing protein 1-like isoform X2 [Calidris pugnax].
XP_013919985.1	ANKZF1	712	eukaryota>metazoa>chordata>vertebrata	Thamnophis sirtalis	PREDICTED: ankyrin repeat and zinc finger domain-containing protein 1 isoform X1 [Thamnophis sirtalis].
XP_015145575.1	ANKZF1	712	eukaryota>metazoa>chordata>vertebrata	Gallus gallus	PREDICTED: ankyrin repeat and zinc finger domain-containing protein 1 isoform X2 [Gallus gallus].
XP_015490569.1	ANKZF1	712	eukaryota>metazoa>chordata>vertebrata	Parus major	PREDICTED: ankyrin repeat and zinc finger domain-containing protein 1 isoform X1 [Parus major].
XP_006245310.1	Ankzf1	696	eukaryota>metazoa>chordata>vertebrata	Rattus norvegicus	PREDICTED: ankyrin repeat and zinc finger domain-containing protein 1 isoform X1 [Rattus norvegicus].
XP_017198631.1	ANKZF1	711	eukaryota>metazoa>chordata>vertebrata	Oryctolagus cuniculus	PREDICTED: LOW QUALITY PROTEIN: ankyrin repeat and zinc finger domain-containing protein 1 [Oryctolagus cuniculus].
KFP61711.1	N322_04146	696	eukaryota>metazoa>chordata>vertebrata	Cariama cristata	Ankyrin repeat and zinc finger domain-containing protein 1, partial [Cariama cristata].
XP_010405224.1	ANKZF1	649	eukaryota>metazoa>chordata>vertebrata	Corvus cornix cornix	PREDICTED: ankyrin repeat and zinc finger domain-containing protein 1 isoform X2 [Corvus cornix cornix].
XP_010711938.1	ANKZF1	710	eukaryota>metazoa>chordata>vertebrata	Meleagris gallopavo	PREDICTED: LOW QUALITY PROTEIN: ankyrin repeat and zinc finger domain-containing protein 1 [Meleagris gallopavo].
XP_011578808.1	ANKZF1	710	eukaryota>metazoa>chordata>vertebrata	Aquila chrysaetos canadensis	PREDICTED: ankyrin repeat and zinc finger domain-containing protein 1 isoform X2 [Aquila chrysaetos canadensis].
XP_008921224.1	ANKZF1	710	eukaryota>metazoa>chordata>vertebrata	Manacus vitellinus	PREDICTED: ankyrin repeat and zinc finger domain-containing protein 1 [Manacus vitellinus].
XP_010566300.1	ANKZF1	710	eukaryota>metazoa>chordata>vertebrata	Haliaeetus leucocephalus	PREDICTED: ankyrin repeat and zinc finger domain-containing protein 1 isoform X2 [Haliaeetus leucocephalus].
XP_013152225.1	ANKZF1	709	eukaryota>metazoa>chordata>vertebrata	Falco peregrinus	PREDICTED: ankyrin repeat and zinc finger domain-containing protein 1 isoform X2 [Falco peregrinus].
XP_009086156.1	ANKZF1	709	eukaryota>metazoa>chordata>vertebrata	Serinus canaria	PREDICTED: ankyrin repeat and zinc finger domain-containing protein 1 [Serinus canaria].
XP_004320386.1	ANKZF1	709	eukaryota>metazoa>chordata>vertebrata	Tursiops truncatus	PREDICTED: LOW QUALITY PROTEIN: ankyrin repeat and zinc finger domain-containing protein 1 [Tursiops truncatus].
XP_015724458.1	ANKZF1	709	eukaryota>metazoa>chordata>vertebrata	Coturnix japonica	PREDICTED: ankyrin repeat and zinc finger domain-containing protein 1 isoform X2 [Coturnix japonica].
XP_014743573.1	ANKZF1	709	eukaryota>metazoa>chordata>vertebrata	Sturnus vulgaris	PREDICTED: ankyrin repeat and zinc finger domain-containing protein 1 isoform X1 [Sturnus vulgaris].
XP_012826407.1	ankzf1	709	eukaryota>metazoa>chordata>vertebrata	Xenopus tropicalis	PREDICTED: ankyrin repeat and zinc finger domain-containing protein 1 [Xenopus tropicalis].
XP_004262743.1	ANKZF1	709	eukaryota>metazoa>chordata>vertebrata	Orcinus orca	PREDICTED: LOW QUALITY PROTEIN: ankyrin repeat and zinc finger domain-containing protein 1 [Orcinus orca].
XP_015724459.1	ANKZF1	708	eukaryota>metazoa>chordata>vertebrata	Coturnix japonica	PREDICTED: ankyrin repeat and zinc finger domain-containing protein 1 isoform X3 [Coturnix japonica].
XP_005049569.1	ANKZF1	707	eukaryota>metazoa>chordata>vertebrata	Ficedula albicollis	PREDICTED: ankyrin repeat and zinc finger domain-containing protein 1 isoform X1 [Ficedula albicollis].
XP_005415856.1	ANKZF1	707	eukaryota>metazoa>chordata>vertebrata	Geospiza fortis	PREDICTED: ankyrin repeat and zinc finger domain-containing protein 1 [Geospiza fortis].
XP_008057063.1	ANKZF1	442	eukaryota>metazoa>chordata>vertebrata	Carlito syrichta	PREDICTED: LOW QUALITY PROTEIN: ankyrin repeat and zinc finger domain-containing protein 1 [Carlito syrichta].
XP_013816091.1	ANKZF1	707	eukaryota>metazoa>chordata>vertebrata	Apteryx australis mantelli	PREDICTED: ankyrin repeat and zinc finger domain-containing protein 1 isoform X3 [Apteryx australis mantelli].
XP_008110324.1	ankzf1	706	eukaryota>metazoa>chordata>vertebrata	Anolis carolinensis	PREDICTED: ankyrin repeat and zinc finger domain-containing protein 1 [Anolis carolinensis].
XP_009909392.1	ANKZF1	706	eukaryota>metazoa>chordata>vertebrata	Picoides pubescens	PREDICTED: ankyrin repeat and zinc finger domain-containing protein 1 [Picoides pubescens].
XP_009674416.1	ANKZF1	705	eukaryota>metazoa>chordata>vertebrata	Struthio camelus australis	PREDICTED: ankyrin repeat and zinc finger domain-containing protein 1 isoform X1 [Struthio camelus australis].
KFU88493.1	M959_07099	705	eukaryota>metazoa>chordata>vertebrata	Chaetura pelagica	Ankyrin repeat and zinc finger domain-containing protein 1, partial [Chaetura pelagica].
XP_015490573.1	ANKZF1	704	eukaryota>metazoa>chordata>vertebrata	Parus major	PREDICTED: ankyrin repeat and zinc finger domain-containing protein 1 isoform X2 [Parus major].
XP_015280558.1	ANKZF1	704	eukaryota>metazoa>chordata>vertebrata	Gekko japonicus	PREDICTED: ankyrin repeat and zinc finger domain-containing protein 1 [Gekko japonicus].
XP_005395620.1	Ankzf1	726	eukaryota>metazoa>chordata>vertebrata	Chinchilla lanigera	PREDICTED: ankyrin repeat and zinc finger domain-containing protein 1 isoform X1 [Chinchilla lanigera].
XP_015097204.1	ANKZF1	703	eukaryota>metazoa>chordata>vertebrata	Vicugna pacos	PREDICTED: ankyrin repeat and zinc finger domain-containing protein 1 isoform X1 [Vicugna pacos].
XP_007501748.1	ANKZF1	703	eukaryota>metazoa>chordata>vertebrata	Monodelphis domestica	PREDICTED: ankyrin repeat and zinc finger domain-containing protein 1 isoform X2 [Monodelphis domestica].
XP_012886102.1	Ankzf1	702	eukaryota>metazoa>chordata>vertebrata	Dipodomys ordii	PREDICTED: ankyrin repeat and zinc finger domain-containing protein 1 [Dipodomys ordii].
ELK17186.1	PAL_GLEAN10014402	702	eukaryota>metazoa>chordata>vertebrata	Pteropus alecto	Ankyrin repeat and zinc finger domain-containing protein 1 [Pteropus alecto].
XP_005985202.1	ANKZF1	720	eukaryota>metazoa>chordata>vertebrata	Pantholops hodgsonii	PREDICTED: ankyrin repeat and zinc finger domain-containing protein 1 isoform X2 [Pantholops hodgsonii].
XP_014121174.1	ANKZF1	701	eukaryota>metazoa>chordata>vertebrata	Zonotrichia albicollis	PREDICTED: ankyrin repeat and zinc finger domain-containing protein 1 [Zonotrichia albicollis].
XP_015097205.1	ANKZF1	701	eukaryota>metazoa>chordata>vertebrata	Vicugna pacos	PREDICTED: ankyrin repeat and zinc finger domain-containing protein 1 isoform X2 [Vicugna pacos].
KFQ36060.1	N331_06306	452	eukaryota>metazoa>chordata>vertebrata	Merops nubicus	Ankyrin repeat and zinc finger domain-containing protein 1, partial [Merops nubicus].
XP_014416488.1	ANKZF1	701	eukaryota>metazoa>chordata>vertebrata	Camelus ferus	PREDICTED: ankyrin repeat and zinc finger domain-containing protein 1 isoform X1 [Camelus ferus].
XP_009643995.1	ANKZF1	699	eukaryota>metazoa>chordata>vertebrata	Egretta garzetta	PREDICTED: ankyrin repeat and zinc finger domain-containing protein 1 [Egretta garzetta].
KFP21608.1	Z169_04688	699	eukaryota>metazoa>chordata>vertebrata	Egretta garzetta	Ankyrin repeat and zinc finger domain-containing protein 1, partial [Egretta garzetta].
KFP96306.1	N329_05446	458	eukaryota>metazoa>chordata>vertebrata	Haliaeetus albicilla	Ankyrin repeat and zinc finger domain-containing protein 1, partial [Haliaeetus albicilla].
EDL00378.1	mCG_133867	458	eukaryota>metazoa>chordata>vertebrata	Mus musculus	mCG133867, isoform CRA_b, partial [Mus musculus].
XP_017280050.1	ankzf1	754	eukaryota>metazoa>chordata>vertebrata>actinopterygii	Kryptolebias marmoratus	PREDICTED: ankyrin repeat and zinc finger domain-containing protein 1 isoform X2 [Kryptolebias marmoratus].
XP_014021238.1	ankzf1	810	eukaryota>metazoa>chordata>vertebrata>actinopterygii	Salmo salar	PREDICTED: ankyrin repeat and zinc finger domain-containing protein 1 isoform X1 [Salmo salar].
KPP70506.1	Z043_110662	712	eukaryota>metazoa>chordata>vertebrata>actinopterygii	Scleropages formosus	ankyrin repeat and zinc finger domain-containing protein 1-like [Scleropages formosus].
XP_015822130.1	ankzf1	717	eukaryota>metazoa>chordata>vertebrata>actinopterygii	Nothobranchius furzeri	PREDICTED: ankyrin repeat and zinc finger domain-containing protein 1 [Nothobranchius furzeri].
XP_015234045.1	ankzf1	579	eukaryota>metazoa>chordata>vertebrata>actinopterygii	Cyprinodon variegatus	PREDICTED: ankyrin repeat and zinc finger domain-containing protein 1 [Cyprinodon variegatus].
KTG39464.1	cypCar_00020013	719	eukaryota>metazoa>chordata>vertebrata>actinopterygii	Cyprinus carpio	hypothetical protein cypCar_00020013 [Cyprinus carpio].
XP_003962026.1	ankzf1	719	eukaryota>metazoa>chordata>vertebrata>actinopterygii	Takifugu rubripes	PREDICTED: ankyrin repeat and zinc finger domain-containing protein 1 [Takifugu rubripes].
XP_016328299.1	LOC107677786	720	eukaryota>metazoa>chordata>vertebrata>actinopterygii	Sinocyclocheilus anshuiensis	PREDICTED: ankyrin repeat and zinc finger domain-containing protein 1-like isoform X3 [Sinocyclocheilus anshuiensis].
XP_016420596.1	LOC107749886	721	eukaryota>metazoa>chordata>vertebrata>actinopterygii	Sinocyclocheilus rhinocerous	PREDICTED: ankyrin repeat and zinc finger domain-containing protein 1-like isoform X3 [Sinocyclocheilus rhinocerous].
XP_011488001.1	ankzf1	721	eukaryota>metazoa>chordata>vertebrata>actinopterygii	Oryzias latipes	PREDICTED: ankyrin repeat and zinc finger domain-containing protein 1 [Oryzias latipes].
XP_016328298.1	LOC107677786	722	eukaryota>metazoa>chordata>vertebrata>actinopterygii	Sinocyclocheilus anshuiensis	PREDICTED: ankyrin repeat and zinc finger domain-containing protein 1-like isoform X2 [Sinocyclocheilus anshuiensis].
XP_016420595.1	LOC107749886	723	eukaryota>metazoa>chordata>vertebrata>actinopterygii	Sinocyclocheilus rhinocerous	PREDICTED: ankyrin repeat and zinc finger domain-containing protein 1-like isoform X2 [Sinocyclocheilus rhinocerous].
XP_696813.2	ankzf1	723	eukaryota>metazoa>chordata>vertebrata>actinopterygii	Danio rerio	PREDICTED: ankyrin repeat and zinc finger domain-containing protein 1 isoform X2 [Danio rerio].
XP_016328297.1	LOC107677786	723	eukaryota>metazoa>chordata>vertebrata>actinopterygii	Sinocyclocheilus anshuiensis	PREDICTED: ankyrin repeat and zinc finger domain-containing protein 1-like isoform X1 [Sinocyclocheilus anshuiensis].
XP_017335772.1	ankzf1	724	eukaryota>metazoa>chordata>vertebrata>actinopterygii	Ictalurus punctatus	PREDICTED: ankyrin repeat and zinc finger domain-containing protein 1 isoform X2 [Ictalurus punctatus].
XP_016420594.1	LOC107749886	724	eukaryota>metazoa>chordata>vertebrata>actinopterygii	Sinocyclocheilus rhinocerous	PREDICTED: ankyrin repeat and zinc finger domain-containing protein 1-like isoform X1 [Sinocyclocheilus rhinocerous].
XP_017335770.1	ankzf1	725	eukaryota>metazoa>chordata>vertebrata>actinopterygii	Ictalurus punctatus	PREDICTED: ankyrin repeat and zinc finger domain-containing protein 1 isoform X1 [Ictalurus punctatus].
XP_016315712.1	LOC107668378	726	eukaryota>metazoa>chordata>vertebrata>actinopterygii	Sinocyclocheilus anshuiensis	PREDICTED: ankyrin repeat and zinc finger domain-containing protein 1-like isoform X2 [Sinocyclocheilus anshuiensis].
XP_010729264.1	ankzf1	727	eukaryota>metazoa>chordata>vertebrata>actinopterygii	Larimichthys crocea	PREDICTED: ankyrin repeat and zinc finger domain-containing protein 1 [Larimichthys crocea].
XP_005800774.1	ankzf1	729	eukaryota>metazoa>chordata>vertebrata>actinopterygii	Xiphophorus maculatus	PREDICTED: ankyrin repeat and zinc finger domain-containing protein 1 [Xiphophorus maculatus].
XP_015458575.1	LOC103038847	646	eukaryota>metazoa>chordata>vertebrata>actinopterygii	Astyanax mexicanus	PREDICTED: ankyrin repeat and zinc finger domain-containing protein 1-like [Astyanax mexicanus].
XP_016315710.1	LOC107668378	729	eukaryota>metazoa>chordata>vertebrata>actinopterygii	Sinocyclocheilus anshuiensis	PREDICTED: ankyrin repeat and zinc finger domain-containing protein 1-like isoform X1 [Sinocyclocheilus anshuiensis].
XP_013872159.1	ankzf1	730	eukaryota>metazoa>chordata>vertebrata>actinopterygii	Austrofundulus limnaeus	PREDICTED: ankyrin repeat and zinc finger domain-containing protein 1 isoform X2 [Austrofundulus limnaeus].
XP_014021249.1	ankzf1	756	eukaryota>metazoa>chordata>vertebrata>actinopterygii	Salmo salar	PREDICTED: ankyrin repeat and zinc finger domain-containing protein 1 isoform X3 [Salmo salar].
XP_010766649.1	ankzf1	732	eukaryota>metazoa>chordata>vertebrata>actinopterygii	Notothenia coriiceps	PREDICTED: ankyrin repeat and zinc finger domain-containing protein 1 isoform X2 [Notothenia coriiceps].
XP_010766648.1	ankzf1	733	eukaryota>metazoa>chordata>vertebrata>actinopterygii	Notothenia coriiceps	PREDICTED: ankyrin repeat and zinc finger domain-containing protein 1 isoform X1 [Notothenia coriiceps].
XP_005453080.1	ankzf1	734	eukaryota>metazoa>chordata>vertebrata>actinopterygii	Oreochromis niloticus	PREDICTED: ankyrin repeat and zinc finger domain-containing protein 1 [Oreochromis niloticus].
XP_014852850.1	ankzf1	734	eukaryota>metazoa>chordata>vertebrata>actinopterygii	Poecilia mexicana	PREDICTED: ankyrin repeat and zinc finger domain-containing protein 1 [Poecilia mexicana].
XP_008297370.1	ankzf1	734	eukaryota>metazoa>chordata>vertebrata>actinopterygii	Stegastes partitus	PREDICTED: ankyrin repeat and zinc finger domain-containing protein 1 isoform X3 [Stegastes partitus].
XP_014021248.1	ankzf1	800	eukaryota>metazoa>chordata>vertebrata>actinopterygii	Salmo salar	PREDICTED: ankyrin repeat and zinc finger domain-containing protein 1 isoform X2 [Salmo salar].
XP_016536318.1	ankzf1	734	eukaryota>metazoa>chordata>vertebrata>actinopterygii	Poecilia formosa	PREDICTED: ankyrin repeat and zinc finger domain-containing protein 1 [Poecilia formosa].
XP_008297369.1	ankzf1	734	eukaryota>metazoa>chordata>vertebrata>actinopterygii	Stegastes partitus	PREDICTED: ankyrin repeat and zinc finger domain-containing protein 1 isoform X2 [Stegastes partitus].
XP_005167550.1	ankzf1	735	eukaryota>metazoa>chordata>vertebrata>actinopterygii	Danio rerio	PREDICTED: ankyrin repeat and zinc finger domain-containing protein 1 isoform X1 [Danio rerio].
XP_008297368.1	ankzf1	735	eukaryota>metazoa>chordata>vertebrata>actinopterygii	Stegastes partitus	PREDICTED: ankyrin repeat and zinc finger domain-containing protein 1 isoform X1 [Stegastes partitus].
XP_008326978.1	ankzf1	735	eukaryota>metazoa>chordata>vertebrata>actinopterygii	Cynoglossus semilaevis	PREDICTED: ankyrin repeat and zinc finger domain-containing protein 1 [Cynoglossus semilaevis].
XP_015214736.1	ankzf1	735	eukaryota>metazoa>chordata>vertebrata>actinopterygii	Lepisosteus oculatus	PREDICTED: ankyrin repeat and zinc finger domain-containing protein 1 isoform X2 [Lepisosteus oculatus].
XP_006788633.1	LOC102776095	736	eukaryota>metazoa>chordata>vertebrata>actinopterygii	Neolamprologus brichardi	PREDICTED: ankyrin repeat and zinc finger domain-containing protein 1-like isoform X2 [Neolamprologus brichardi].
KKF19568.1	EH28_03783	642	eukaryota>metazoa>chordata>vertebrata>actinopterygii	Larimichthys crocea	Ankyrin repeat and zinc finger domain-containing protein 1 [Larimichthys crocea].
XP_008402181.1	ankzf1	736	eukaryota>metazoa>chordata>vertebrata>actinopterygii	Poecilia reticulata	PREDICTED: ankyrin repeat and zinc finger domain-containing protein 1 [Poecilia reticulata].
XP_013872157.1	ankzf1	736	eukaryota>metazoa>chordata>vertebrata>actinopterygii	Austrofundulus limnaeus	PREDICTED: ankyrin repeat and zinc finger domain-containing protein 1 isoform X1 [Austrofundulus limnaeus].
XP_016142783.1	LOC107596542	663	eukaryota>metazoa>chordata>vertebrata>actinopterygii	Sinocyclocheilus grahami	PREDICTED: ankyrin repeat and zinc finger domain-containing protein 1-like [Sinocyclocheilus grahami].
XP_016138079.1	LOC107593010	637	eukaryota>metazoa>chordata>vertebrata>actinopterygii	Sinocyclocheilus grahami	PREDICTED: ankyrin repeat and zinc finger domain-containing protein 1-like [Sinocyclocheilus grahami].
XP_015214732.1	ankzf1	737	eukaryota>metazoa>chordata>vertebrata>actinopterygii	Lepisosteus oculatus	PREDICTED: ankyrin repeat and zinc finger domain-containing protein 1 isoform X1 [Lepisosteus oculatus].
CAG09306.1	GSTEN:00030538:G:001	627	eukaryota>metazoa>chordata>vertebrata>actinopterygii	Tetraodon nigroviridis	unnamed protein product [Tetraodon nigroviridis].
XP_014896653.1	ankzf1	737	eukaryota>metazoa>chordata>vertebrata>actinopterygii	Poecilia latipinna	PREDICTED: ankyrin repeat and zinc finger domain-containing protein 1 [Poecilia latipinna].
XP_012733816.1	ankzf1	738	eukaryota>metazoa>chordata>vertebrata>actinopterygii	Fundulus heteroclitus	PREDICTED: ankyrin repeat and zinc finger domain-containing protein 1 [Fundulus heteroclitus].
XP_005723423.1	ankzf1	741	eukaryota>metazoa>chordata>vertebrata>actinopterygii	Pundamilia nyererei	PREDICTED: ankyrin repeat and zinc finger domain-containing protein 1 [Pundamilia nyererei].
XP_004555486.1	ankzf1	741	eukaryota>metazoa>chordata>vertebrata>actinopterygii	Maylandia zebra	PREDICTED: ankyrin repeat and zinc finger domain-containing protein 1 [Maylandia zebra].
XP_006788632.1	LOC102776095	741	eukaryota>metazoa>chordata>vertebrata>actinopterygii	Neolamprologus brichardi	PREDICTED: ankyrin repeat and zinc finger domain-containing protein 1-like isoform X1 [Neolamprologus brichardi].
XP_005918320.1	ankzf1	741	eukaryota>metazoa>chordata>vertebrata>actinopterygii	Haplochromis burtoni	PREDICTED: ankyrin repeat and zinc finger domain-containing protein 1 [Haplochromis burtoni].
KTF94704.1	cypCar_00002081	616	eukaryota>metazoa>chordata>vertebrata>actinopterygii	Cyprinus carpio	hypothetical protein cypCar_00002081 [Cyprinus carpio].
XP_017335776.1	ankzf1	600	eukaryota>metazoa>chordata>vertebrata>actinopterygii	Ictalurus punctatus	PREDICTED: ankyrin repeat and zinc finger domain-containing protein 1 isoform X5 [Ictalurus punctatus].
XP_012675984.1	ankzf1	769	eukaryota>metazoa>chordata>vertebrata>actinopterygii	Clupea harengus	PREDICTED: ankyrin repeat and zinc finger domain-containing protein 1 [Clupea harengus].
XP_017280049.1	ankzf1	761	eukaryota>metazoa>chordata>vertebrata>actinopterygii	Kryptolebias marmoratus	PREDICTED: ankyrin repeat and zinc finger domain-containing protein 1 isoform X1 [Kryptolebias marmoratus].
XP_012986567.1	ankzf1	756	eukaryota>metazoa>chordata>vertebrata>actinopterygii	Esox lucius	PREDICTED: ankyrin repeat and zinc finger domain-containing protein 1 [Esox lucius].
KXJ28197.1	AC249_AIPGENE1827	731	eukaryota>metazoa>cnidaria	Exaiptasia pallida	Ankyrin repeat and zinc finger domain-containing protein 1 [Exaiptasia pallida].
EFX71896.1	DAPPUDRAFT_326770	654	eukaryota>metazoa>crustacea	Daphnia pulex	hypothetical protein DAPPUDRAFT_326770 [Daphnia pulex].
XP_006822109.1	LOC102805323	825	eukaryota>metazoa>hemichordata	Saccoglossus kowalevskii	PREDICTED: ankyrin repeat and zinc finger domain-containing protein 1-like [Saccoglossus kowalevskii].
KYN27142.1	ALC57_03485	752	eukaryota>metazoa>hexapoda	Trachymyrmex cornetzi	Ankyrin repeat and zinc finger domain-containing protein 1 [Trachymyrmex cornetzi].
XP_011696807.1	LOC105455284	751	eukaryota>metazoa>hexapoda	Wasmannia auropunctata	PREDICTED: ankyrin repeat and zinc finger domain-containing protein 1-like isoform X2 [Wasmannia auropunctata].
XP_013145331.1	LOC106108624	676	eukaryota>metazoa>hexapoda	Papilio polytes	PREDICTED: ankyrin repeat and zinc finger domain-containing protein 1-like [Papilio polytes].
XP_011170666.1	LOC105203547	749	eukaryota>metazoa>hexapoda	Solenopsis invicta	PREDICTED: ankyrin repeat and zinc finger domain-containing protein 1-like isoform X2 [Solenopsis invicta].
XP_016663849.1	LOC103307675	676	eukaryota>metazoa>hexapoda	Acyrthosiphon pisum	PREDICTED: ankyrin repeat and zinc finger domain-containing protein 1 isoform X1 [Acyrthosiphon pisum].
XP_014360206.1	LOC106712224	678	eukaryota>metazoa>hexapoda	Papilio machaon	PREDICTED: ankyrin repeat and zinc finger domain-containing protein 1-like isoform X1 [Papilio machaon].
XP_012226347.1	LOC105674536	747	eukaryota>metazoa>hexapoda	Linepithema humile	PREDICTED: ankyrin repeat and zinc finger domain-containing protein 1-like isoform X2 [Linepithema humile].
XP_013194292.1	LOC106137900	678	eukaryota>metazoa>hexapoda	Amyelois transitella	PREDICTED: ankyrin repeat and zinc finger domain-containing protein 1-like [Amyelois transitella].
XP_011341841.1	LOC105281945	765	eukaryota>metazoa>hexapoda	Cerapachys biroi	PREDICTED: ankyrin repeat and zinc finger domain-containing protein 1-like [Cerapachys biroi].
KPI95693.1	RR46_11406	678	eukaryota>metazoa>hexapoda	Papilio xuthus	Ankyrin repeat and zinc finger domain-containing protein 1 [Papilio xuthus].
KPJ19926.1	RR48_04087	772	eukaryota>metazoa>hexapoda	Papilio machaon	Ankyrin repeat and zinc finger domain-containing protein 1 [Papilio machaon].
KOC66669.1	WH47_00878	776	eukaryota>metazoa>hexapoda	Habropoda laboriosa	Ankyrin repeat and zinc finger domain-containing protein 1 [Habropoda laboriosa].
XP_011308898.1	LOC105269961	776	eukaryota>metazoa>hexapoda	Fopius arisanus	PREDICTED: ankyrin repeat and zinc finger domain-containing protein 1-like isoform X1 [Fopius arisanus].
XP_003492750.1	LOC100744548	779	eukaryota>metazoa>hexapoda	Bombus impatiens	PREDICTED: ankyrin repeat and zinc finger domain-containing protein 1-like isoform X1 [Bombus impatiens].
XP_003395480.1	LOC100651247	779	eukaryota>metazoa>hexapoda	Bombus terrestris	PREDICTED: LOW QUALITY PROTEIN: ankyrin repeat and zinc finger domain-containing protein 1-like [Bombus terrestris].
XP_011261642.1	LOC105254574	781	eukaryota>metazoa>hexapoda	Camponotus floridanus	PREDICTED: ankyrin repeat and zinc finger domain-containing protein 1-like isoform X2 [Camponotus floridanus].
XP_002426497.1	Phum_PHUM256970	582	eukaryota>metazoa>hexapoda	Pediculus humanus corporis	conserved hypothetical protein [Pediculus humanus corporis].
XP_012139083.1	LOC100882662	782	eukaryota>metazoa>hexapoda	Megachile rotundata	PREDICTED: ankyrin repeat and zinc finger domain-containing protein 1-like [Megachile rotundata].
OAD54422.1	WN48_07786	784	eukaryota>metazoa>hexapoda	Eufriesea mexicana	Ankyrin repeat and zinc finger domain-containing protein 1 [Eufriesea mexicana].
XP_015440018.1	LOC107194829	785	eukaryota>metazoa>hexapoda	Dufourea novaeangliae	PREDICTED: ankyrin repeat and zinc finger domain-containing protein 1-like [Dufourea novaeangliae].
XP_011636292.1	LOC105426668	789	eukaryota>metazoa>hexapoda	Pogonomyrmex barbatus	PREDICTED: ankyrin repeat and zinc finger domain-containing protein 1-like isoform X1 [Pogonomyrmex barbatus].
XP_015111543.1	LOC107037490	791	eukaryota>metazoa>hexapoda	Diachasma alloeum	PREDICTED: ankyrin repeat and zinc finger domain-containing protein 1-like [Diachasma alloeum].
XP_015190620.1	LOC107074083	791	eukaryota>metazoa>hexapoda	Polistes dominula	PREDICTED: ankyrin repeat and zinc finger domain-containing protein 1-like [Polistes dominula].
XP_006567709.1	LOC409046	791	eukaryota>metazoa>hexapoda	Apis mellifera	PREDICTED: ankyrin repeat and zinc finger domain-containing protein 1-like [Apis mellifera].
XP_012257096.1	LOC105686650	791	eukaryota>metazoa>hexapoda	Athalia rosae	PREDICTED: ankyrin repeat and zinc finger domain-containing protein 1-like isoform X2 [Athalia rosae].
XP_003696594.1	LOC100864090	791	eukaryota>metazoa>hexapoda	Apis florea	PREDICTED: ankyrin repeat and zinc finger domain-containing protein 1-like [Apis florea].
KMQ97754.1	RF55_1908	746	eukaryota>metazoa>hexapoda	Lasius niger	ankyrin repeat and zinc finger domain-containing protein 1-like protein [Lasius niger].
XP_012257095.1	LOC105686650	792	eukaryota>metazoa>hexapoda	Athalia rosae	PREDICTED: ankyrin repeat and zinc finger domain-containing protein 1-like isoform X1 [Athalia rosae].
KYN36049.1	ALC56_09586	792	eukaryota>metazoa>hexapoda	Trachymyrmex septentrionalis	Ankyrin repeat and zinc finger domain-containing protein 1 [Trachymyrmex septentrionalis].
XP_008482417.1	LOC103519111	668	eukaryota>metazoa>hexapoda	Diaphorina citri	PREDICTED: ankyrin repeat and zinc finger domain-containing protein 1 [Diaphorina citri].
XP_016912234.1	LOC107997850	792	eukaryota>metazoa>hexapoda	Apis cerana	PREDICTED: ankyrin repeat and zinc finger domain-containing protein 1-like [Apis cerana].
XP_011170659.1	LOC105203547	792	eukaryota>metazoa>hexapoda	Solenopsis invicta	PREDICTED: ankyrin repeat and zinc finger domain-containing protein 1-like isoform X1 [Solenopsis invicta].
XP_011063458.1	LOC105151449	792	eukaryota>metazoa>hexapoda	Acromyrmex echinatior	PREDICTED: ankyrin repeat and zinc finger domain-containing protein 1-like isoform X1 [Acromyrmex echinatior].
XP_014614077.1	LOC106792279	792	eukaryota>metazoa>hexapoda	Polistes canadensis	PREDICTED: ankyrin repeat and zinc finger domain-containing protein 1-like isoform X1 [Polistes canadensis].
XP_966567.1	LOC655005	612	eukaryota>metazoa>hexapoda	Tribolium castaneum	PREDICTED: ankyrin repeat and zinc finger domain-containing protein 1 [Tribolium castaneum].
KYN01265.1	ALC62_07884	793	eukaryota>metazoa>hexapoda	Cyphomyrmex costatus	Ankyrin repeat and zinc finger domain-containing protein 1 [Cyphomyrmex costatus].
XP_011867592.1	LOC105561853	793	eukaryota>metazoa>hexapoda	Vollenhovia emeryi	PREDICTED: ankyrin repeat and zinc finger domain-containing protein 1-like [Vollenhovia emeryi].
XP_011636293.1	LOC105426668	744	eukaryota>metazoa>hexapoda	Pogonomyrmex barbatus	PREDICTED: ankyrin repeat and zinc finger domain-containing protein 1-like isoform X2 [Pogonomyrmex barbatus].
XP_013170279.1	LOC106119698	682	eukaryota>metazoa>hexapoda	Papilio xuthus	PREDICTED: ankyrin repeat and zinc finger domain-containing protein 1-like [Papilio xuthus].
KYM84879.1	ALC53_05028	793	eukaryota>metazoa>hexapoda	Atta colombica	Ankyrin repeat and zinc finger domain-containing protein 1 [Atta colombica].
XP_012057182.1	LOC105620289	793	eukaryota>metazoa>hexapoda	Atta cephalotes	PREDICTED: ankyrin repeat and zinc finger domain-containing protein 1-like [Atta cephalotes].
XP_012226346.1	LOC105674536	793	eukaryota>metazoa>hexapoda	Linepithema humile	PREDICTED: ankyrin repeat and zinc finger domain-containing protein 1-like isoform X1 [Linepithema humile].
XP_014242281.1	LOC106662597	683	eukaryota>metazoa>hexapoda	Cimex lectularius	PREDICTED: ankyrin repeat and zinc finger domain-containing protein 1-like isoform X1 [Cimex lectularius].
XP_012546639.1	LOC101735746	683	eukaryota>metazoa>hexapoda	Bombyx mori	PREDICTED: ankyrin repeat and zinc finger domain-containing protein 1-like isoform X4 [Bombyx mori].
XP_011696806.1	LOC105455284	794	eukaryota>metazoa>hexapoda	Wasmannia auropunctata	PREDICTED: ankyrin repeat and zinc finger domain-containing protein 1-like isoform X1 [Wasmannia auropunctata].
XP_015524854.1	LOC107228026	794	eukaryota>metazoa>hexapoda	Neodiprion lecontei	PREDICTED: ankyrin repeat and zinc finger domain-containing protein 1-like [Neodiprion lecontei].
XP_012546638.1	LOC101735746	684	eukaryota>metazoa>hexapoda	Bombyx mori	PREDICTED: ankyrin repeat and zinc finger domain-containing protein 1-like isoform X3 [Bombyx mori].
KZC06435.1	WN55_10345	794	eukaryota>metazoa>hexapoda	Dufourea novaeangliae	Ankyrin repeat and zinc finger domain-containing protein 1 [Dufourea novaeangliae].
KOB76285.1	OBRU01_05946	266	eukaryota>metazoa>hexapoda	Operophtera brumata	Ankyrin repeat and zinc finger domain-containing protein 1 [Operophtera brumata].
XP_011552151.1	LOC105383751	688	eukaryota>metazoa>hexapoda	Plutella xylostella	PREDICTED: ankyrin repeat and zinc finger domain-containing protein 1-like [Plutella xylostella].
XP_014276055.1	LOC106680691	691	eukaryota>metazoa>hexapoda	Halyomorpha halys	PREDICTED: ankyrin repeat and zinc finger domain-containing protein 1-like [Halyomorpha halys].
XP_015606845.1	LOC107273300	800	eukaryota>metazoa>hexapoda	Cephus cinctus	PREDICTED: ankyrin repeat and zinc finger domain-containing protein 1-like [Cephus cinctus].
KYQ48057.1	ALC60_13017	800	eukaryota>metazoa>hexapoda	Trachymyrmex zeteki	Ankyrin repeat and zinc finger domain-containing protein 1 [Trachymyrmex zeteki].
XP_011152539.1	LOC105191093	803	eukaryota>metazoa>hexapoda	Harpegnathos saltator	PREDICTED: ankyrin repeat and zinc finger domain-containing protein 1-like [Harpegnathos saltator].
XP_015364279.1	LOC107162078	657	eukaryota>metazoa>hexapoda	Diuraphis noxia	PREDICTED: ankyrin repeat and zinc finger domain-containing protein 1-like [Diuraphis noxia].
XP_014477833.1	LOC106746121	810	eukaryota>metazoa>hexapoda	Dinoponera quadriceps	PREDICTED: ankyrin repeat and zinc finger domain-containing protein 1-like [Dinoponera quadriceps].
XP_012546637.1	LOC101735746	696	eukaryota>metazoa>hexapoda	Bombyx mori	PREDICTED: ankyrin repeat and zinc finger domain-containing protein 1-like isoform X2 [Bombyx mori].
XP_011261640.1	LOC105254574	790	eukaryota>metazoa>hexapoda	Camponotus floridanus	PREDICTED: ankyrin repeat and zinc finger domain-containing protein 1-like isoform X1 [Camponotus floridanus].
EFN75723.1	EAI_09725	817	eukaryota>metazoa>hexapoda	Harpegnathos saltator	Ankyrin repeat and zinc finger domain-containing protein 1 [Harpegnathos saltator].
XP_008187953.2	LOC103307675	650	eukaryota>metazoa>hexapoda	Acyrthosiphon pisum	PREDICTED: ankyrin repeat and zinc finger domain-containing protein 1 isoform X2 [Acyrthosiphon pisum].
XP_012546636.1	LOC101735746	697	eukaryota>metazoa>hexapoda	Bombyx mori	PREDICTED: ankyrin repeat and zinc finger domain-containing protein 1-like isoform X1 [Bombyx mori].
ENN70520.1	YQE_12696	829	eukaryota>metazoa>hexapoda	Dendroctonus ponderosae	hypothetical protein YQE_12696, partial [Dendroctonus ponderosae].
XP_005091111.1	LOC101854545	795	eukaryota>metazoa>mollusca	Aplysia californica	PREDICTED: ankyrin repeat and zinc finger domain-containing protein 1-like [Aplysia californica].
XP_013087952.1	LOC106072169	723	eukaryota>metazoa>mollusca	Biomphalaria glabrata	PREDICTED: ankyrin repeat and zinc finger domain-containing protein 1-like [Biomphalaria glabrata].
EYB86765.1	Y032_0273g972	607	eukaryota>metazoa>nematoda	Ancylostoma ceylanicum	hypothetical protein Y032_0273g972 [Ancylostoma ceylanicum].
KRX60246.1	T09_6192	647	eukaryota>metazoa>nematoda	Trichinella sp. T9	Ankyrin repeat and zinc finger domain-containing protein 1 [Trichinella sp. T9].
CAP39465.2	CBG_22970	648	eukaryota>metazoa>nematoda	Caenorhabditis briggsae	Protein CBR-VMS-1 [Caenorhabditis briggsae].
XP_003138977.1	LOAG_03392	609	eukaryota>metazoa>nematoda	Loa loa	hypothetical protein LOAG_03392 [Loa loa].
KHN78173.1	Tcan_03575	611	eukaryota>metazoa>nematoda	Toxocara canis	Protein vms-1 [Toxocara canis].
CRZ21894.1	BM_Bm4842	611	eukaryota>metazoa>nematoda	Brugia malayi	BMA-VMS-1 [Brugia malayi].
EGT48953.1	CAEBREN_28663	618	eukaryota>metazoa>nematoda	Caenorhabditis brenneri	hypothetical protein CAEBREN_28663 [Caenorhabditis brenneri].
NP_498765.1	CELE_K06H7.3	618	eukaryota>metazoa>nematoda	Caenorhabditis elegans	Protein vms-1 [Caenorhabditis elegans].
XP_003104884.1	CRE_24512	621	eukaryota>metazoa>nematoda	Caenorhabditis remanei	hypothetical protein CRE_24512 [Caenorhabditis remanei].
XP_002643054.1	CBG22970	626	eukaryota>metazoa>nematoda	Caenorhabditis briggsae	Hypothetical protein CBG22970 [Caenorhabditis briggsae].
KRY48798.1	T03_7338	640	eukaryota>metazoa>nematoda	Trichinella britovi	Ankyrin repeat and zinc finger domain-containing protein 1 [Trichinella britovi].
KRX72925.1	T06_5250	640	eukaryota>metazoa>nematoda	Trichinella sp. T6	Protein vms-1 [Trichinella sp. T6].
KRY28970.1	T01_15347	640	eukaryota>metazoa>nematoda	Trichinella spiralis	Ankyrin repeat and zinc finger domain-containing protein 1 [Trichinella spiralis].
KRY15191.1	T12_15949	640	eukaryota>metazoa>nematoda	Trichinella patagoniensis	Protein vms-1 [Trichinella patagoniensis].
KRZ87345.1	T08_8267	640	eukaryota>metazoa>nematoda	Trichinella sp. T8	Protein vms-1 [Trichinella sp. T8].
KRZ72390.1	T10_5141	640	eukaryota>metazoa>nematoda	Trichinella papuae	Protein vms-1 [Trichinella papuae].
KRY92007.1	T4D_14982	640	eukaryota>metazoa>nematoda	Trichinella pseudospiralis	Protein vms-1 [Trichinella pseudospiralis].
KRX90437.1	T4E_11699	640	eukaryota>metazoa>nematoda	Trichinella pseudospiralis	Protein vms-1 [Trichinella pseudospiralis].
XP_003371988.1	Tsp_05644	673	eukaryota>metazoa>nematoda	Trichinella spiralis	hypothetical protein Tsp_05644 [Trichinella spiralis].
KRX60247.1	T09_6192	640	eukaryota>metazoa>nematoda	Trichinella sp. T9	Ankyrin repeat and zinc finger domain-containing protein 1 [Trichinella sp. T9].
KRX20490.1	T07_12543	640	eukaryota>metazoa>nematoda	Trichinella nelsoni	Ankyrin repeat and zinc finger domain-containing protein 1 [Trichinella nelsoni].
KRX40218.1	T05_13785	640	eukaryota>metazoa>nematoda	Trichinella murrelli	Ankyrin repeat and zinc finger domain-containing protein 1 [Trichinella murrelli].
KRZ03804.1	T11_13347	640	eukaryota>metazoa>nematoda	Trichinella zimbabwensis	Protein vms-1 [Trichinella zimbabwensis].
KRZ52616.1	T02_1570	640	eukaryota>metazoa>nematoda	Trichinella nativa	Protein vms-1 [Trichinella nativa].
ERG79328.1	ASU_13837	638	eukaryota>metazoa>nematoda	Ascaris suum	protein vms-1 [Ascaris suum].
XP_011017511.1	LOC105120823	664	eukaryota>viridiplantae	Populus euphratica	PREDICTED: ankyrin repeat and zinc finger domain-containing protein 1 isoform X2 [Populus euphratica].
XP_003623737.2	MTR_7g075170	635	eukaryota>viridiplantae	Medicago truncatula	ankyrin repeat and zinc finger protein, putative [Medicago truncatula].
CAN65446.1	VITISV_011424	634	eukaryota>viridiplantae	Vitis vinifera	hypothetical protein VITISV_011424 [Vitis vinifera].
XP_002302543.2	POPTR_0002s15080g	664	eukaryota>viridiplantae	Populus trichocarpa	hypothetical protein POPTR_0002s15080g [Populus trichocarpa].
XP_011017509.1	LOC105120823	665	eukaryota>viridiplantae	Populus euphratica	PREDICTED: ankyrin repeat and zinc finger domain-containing protein 1 isoform X1 [Populus euphratica].
XP_012843620.1	LOC105963724	665	eukaryota>viridiplantae	Erythranthe guttata	PREDICTED: ankyrin repeat and zinc finger domain-containing protein 1 [Erythranthe guttata].
XP_006386575.1	POPTR_0002s15080g	640	eukaryota>viridiplantae	Populus trichocarpa	zinc finger family protein [Populus trichocarpa].
XP_008343071.1	LOC103405836	661	eukaryota>viridiplantae	Malus domestica	PREDICTED: ankyrin repeat and zinc finger domain-containing protein 1 isoform X2 [Malus domestica].
XP_010544372.1	LOC104817014	622	eukaryota>viridiplantae	Tarenaya hassleriana	PREDICTED: ankyrin repeat and zinc finger domain-containing protein 1 [Tarenaya hassleriana].
XP_009379277.1	LOC103967708	661	eukaryota>viridiplantae	Pyrus x bretschneideri	PREDICTED: ankyrin repeat and zinc finger domain-containing protein 1 isoform X1 [Pyrus x bretschneideri].
XP_011469125.1	LOC101313737	644	eukaryota>viridiplantae	Fragaria vesca subsp. vesca	PREDICTED: ankyrin repeat and zinc finger domain-containing protein 1 isoform X2 [Fragaria vesca subsp. vesca].
XP_017419540.1	LOC108329706	660	eukaryota>viridiplantae	Vigna angularis	PREDICTED: ankyrin repeat and zinc finger domain-containing protein 1 [Vigna angularis].
EEC77836.1	OsI_17059	666	eukaryota>viridiplantae	Oryza sativa Indica Group	hypothetical protein OsI_17059 [Oryza sativa Indica Group].
XP_010041600.1	LOC104430555	615	eukaryota>viridiplantae	Eucalyptus grandis	PREDICTED: LOW QUALITY PROTEIN: ankyrin repeat and zinc finger domain-containing protein 1-like [Eucalyptus grandis].
XP_015636890.1	LOC4336732	666	eukaryota>viridiplantae	Oryza sativa Japonica Group	PREDICTED: ankyrin repeat and zinc finger domain-containing protein 1 [Oryza sativa Japonica Group].
XP_006586659.1	LOC100527709	666	eukaryota>viridiplantae	Glycine max	PREDICTED: uncharacterized protein LOC100527709 isoform X1 [Glycine max].
XP_015954057.1	LOC107478432	666	eukaryota>viridiplantae	Arachis duranensis	PREDICTED: ankyrin repeat and zinc finger domain-containing protein 1 [Arachis duranensis].
EPS61203.1	M569_13596	645	eukaryota>viridiplantae	Genlisea aurea	hypothetical protein M569_13596, partial [Genlisea aurea].
XP_014490235.1	LOC106752964	660	eukaryota>viridiplantae	Vigna radiata var. radiata	PREDICTED: ankyrin repeat and zinc finger domain-containing protein 1 [Vigna radiata var. radiata].
KMZ57535.1	ZOSMA_85G01060	660	eukaryota>viridiplantae	Zostera marina	hypothetical protein ZOSMA_85G01060 [Zostera marina].
KVH90302.1	Ccrd_007681	608	eukaryota>viridiplantae	Cynara cardunculus var. scolymus	Ankyrin repeat-containing protein [Cynara cardunculus var. scolymus].
XP_004492649.1	LOC101503474	646	eukaryota>viridiplantae	Cicer arietinum	PREDICTED: ankyrin repeat and zinc finger domain-containing protein 1 isoform X1 [Cicer arietinum].
KYP64276.1	KK1_018868	666	eukaryota>viridiplantae	Cajanus cajan	Ankyrin repeat and zinc finger domain-containing protein 1 [Cajanus cajan].
XP_015888808.1	LOC107423709	606	eukaryota>viridiplantae	Ziziphus jujuba	PREDICTED: ankyrin repeat and zinc finger domain-containing protein 1 [Ziziphus jujuba].
XP_003581552.1	LOC100840893	668	eukaryota>viridiplantae	Brachypodium distachyon	PREDICTED: ankyrin repeat and zinc finger domain-containing protein 1 [Brachypodium distachyon].
XP_017248062.1	LOC108219243	668	eukaryota>viridiplantae	Daucus carota subsp. sativus	PREDICTED: ankyrin repeat and zinc finger domain-containing protein 1 [Daucus carota subsp. sativus].
KXG26960.1	SORBI_006G186400	668	eukaryota>viridiplantae	Sorghum bicolor	hypothetical protein SORBI_006G186400 [Sorghum bicolor].
XP_002270342.1	LOC100254117	668	eukaryota>viridiplantae	Vitis vinifera	PREDICTED: ankyrin repeat and zinc finger domain-containing protein 1 [Vitis vinifera].
KDO85868.1	CISIN_1g005932mg	669	eukaryota>viridiplantae	Citrus sinensis	hypothetical protein CISIN_1g005932mg [Citrus sinensis].
XP_006445227.1	CICLE_v10019178mg	669	eukaryota>viridiplantae	Citrus clementina	hypothetical protein CICLE_v10019178mg [Citrus clementina].
XP_010277447.1	LOC104611881	596	eukaryota>viridiplantae	Nelumbo nucifera	PREDICTED: ankyrin repeat and zinc finger domain-containing protein 1 isoform X2 [Nelumbo nucifera].
OAY62393.1	MANES_01G264800	670	eukaryota>viridiplantae	Manihot esculenta	hypothetical protein MANES_01G264800 [Manihot esculenta].
XP_016717635.1	LOC107930478	671	eukaryota>viridiplantae	Gossypium hirsutum	PREDICTED: ankyrin repeat and zinc finger domain-containing protein 1-like [Gossypium hirsutum].
XP_007220900.1	PRUPE_ppa002451mg	671	eukaryota>viridiplantae	Prunus persica	hypothetical protein PRUPE_ppa002451mg [Prunus persica].
XP_007139984.1	PHAVU_008G075100g	671	eukaryota>viridiplantae	Phaseolus vulgaris	hypothetical protein PHAVU_008G075100g [Phaseolus vulgaris].
XP_007052019.1	TCM_005485	672	eukaryota>viridiplantae	Theobroma cacao	Zinc finger protein-related [Theobroma cacao].
XP_002511739.1	LOC8270657	666	eukaryota>viridiplantae	Ricinus communis	PREDICTED: ankyrin repeat and zinc finger domain-containing protein 1 [Ricinus communis].
KHN20464.1	glysoja_037340	676	eukaryota>viridiplantae	Glycine soja	Ankyrin repeat and zinc finger domain-containing protein 1 [Glycine soja].
XP_011650787.1	LOC101216211	676	eukaryota>viridiplantae	Cucumis sativus	PREDICTED: ankyrin repeat and zinc finger domain-containing protein 1 [Cucumis sativus].
XP_009397504.1	LOC103982329	677	eukaryota>viridiplantae	Musa acuminata subsp. malaccensis	PREDICTED: ankyrin repeat and zinc finger domain-containing protein 1 [Musa acuminata subsp. malaccensis].
XP_008438141.1	LOC103483337	678	eukaryota>viridiplantae	Cucumis melo	PREDICTED: ankyrin repeat and zinc finger domain-containing protein 1 [Cucumis melo].
XP_016575551.1	LOC107873281	679	eukaryota>viridiplantae	Capsicum annuum	PREDICTED: ankyrin repeat and zinc finger domain-containing protein 1 [Capsicum annuum].
XP_010092858.1	L484_022453	679	eukaryota>viridiplantae	Morus notabilis	hypothetical protein L484_022453 [Morus notabilis].
XP_012083548.1	LOC105643100	680	eukaryota>viridiplantae	Jatropha curcas	PREDICTED: ankyrin repeat and zinc finger domain-containing protein 1 [Jatropha curcas].
XP_010692314.1	LOC104905460	682	eukaryota>viridiplantae	Beta vulgaris subsp. vulgaris	PREDICTED: ankyrin repeat and zinc finger domain-containing protein 1 [Beta vulgaris subsp. vulgaris].
KNA25809.1	SOVF_003120	683	eukaryota>viridiplantae	Spinacia oleracea	hypothetical protein SOVF_003120 isoform A [Spinacia oleracea].
XP_012473352.1	LOC105790341	684	eukaryota>viridiplantae	Gossypium raimondii	PREDICTED: ankyrin repeat and zinc finger domain-containing protein 1 [Gossypium raimondii].
XP_006858020.2	LOC18447872	685	eukaryota>viridiplantae	Amborella trichopoda	PREDICTED: LOW QUALITY PROTEIN: ankyrin repeat and zinc finger domain-containing protein 1 [Amborella trichopoda].
XP_016708226.1	LOC107922626	686	eukaryota>viridiplantae	Gossypium hirsutum	PREDICTED: ankyrin repeat and zinc finger domain-containing protein 1-like [Gossypium hirsutum].
KHG29693.1	F383_15567	686	eukaryota>viridiplantae	Gossypium arboreum	Ankyrin repeat and zinc finger domain-containing 1 [Gossypium arboreum].
XP_006602758.1	LOC100808912	689	eukaryota>viridiplantae	Glycine max	PREDICTED: ankyrin repeat and zinc finger domain-containing protein 1-like [Glycine max].
XP_010053885.1	LOC104442231	689	eukaryota>viridiplantae	Eucalyptus grandis	PREDICTED: ankyrin repeat and zinc finger domain-containing protein 1 [Eucalyptus grandis].
GAQ84657.1	KFL_001990100	690	eukaryota>viridiplantae	Klebsormidium flaccidum	hypothetical protein KFL_001990100 [Klebsormidium flaccidum].
XP_016501968.1	LOC107820234	693	eukaryota>viridiplantae	Nicotiana tabacum	PREDICTED: ankyrin repeat and zinc finger domain-containing protein 1-like [Nicotiana tabacum].
XP_016477806.1	LOC107799232	693	eukaryota>viridiplantae	Nicotiana tabacum	PREDICTED: ankyrin repeat and zinc finger domain-containing protein 1-like [Nicotiana tabacum].
XP_009770763.1	LOC104221397	693	eukaryota>viridiplantae	Nicotiana sylvestris	PREDICTED: ankyrin repeat and zinc finger domain-containing protein 1 [Nicotiana sylvestris].
KXG26959.1	SORBI_006G186400	507	eukaryota>viridiplantae	Sorghum bicolor	hypothetical protein SORBI_006G186400 [Sorghum bicolor].
XP_009605450.1	LOC104100000	693	eukaryota>viridiplantae	Nicotiana tomentosiformis	PREDICTED: ankyrin repeat and zinc finger domain-containing protein 1 [Nicotiana tomentosiformis].
XP_006339446.1	LOC102583786	693	eukaryota>viridiplantae	Solanum tuberosum	PREDICTED: ankyrin repeat and zinc finger domain-containing protein 1 [Solanum tuberosum].
XP_016191904.1	LOC107632759	491	eukaryota>viridiplantae	Arachis ipaensis	PREDICTED: ankyrin repeat and zinc finger domain-containing protein 1-like [Arachis ipaensis].
XP_015078718.1	LOC107022333	694	eukaryota>viridiplantae	Solanum pennellii	PREDICTED: ankyrin repeat and zinc finger domain-containing protein 1 [Solanum pennellii].
XP_004229826.1	LOC101256416	694	eukaryota>viridiplantae	Solanum lycopersicum	PREDICTED: ankyrin repeat and zinc finger domain-containing protein 1 [Solanum lycopersicum].
XP_008232691.1	LOC103331810	695	eukaryota>viridiplantae	Prunus mume	PREDICTED: ankyrin repeat and zinc finger domain-containing protein 1 [Prunus mume].
XP_011090984.1	LOC105171529	697	eukaryota>viridiplantae	Sesamum indicum	PREDICTED: ankyrin repeat and zinc finger domain-containing protein 1 [Sesamum indicum].
CDP17791.1	GSCOC_T00003914001	697	eukaryota>viridiplantae	Coffea canephora	unnamed protein product [Coffea canephora].
KNA25810.1	SOVF_003120	701	eukaryota>viridiplantae	Spinacia oleracea	hypothetical protein SOVF_003120 isoform B [Spinacia oleracea].
OAY64546.1	ACMD2_14358	702	eukaryota>viridiplantae	Ananas comosus	Ankyrin repeat and zinc finger domain-containing protein 1 [Ananas comosus].
XP_004492648.1	LOC101503474	707	eukaryota>viridiplantae	Cicer arietinum	PREDICTED: ankyrin repeat and zinc finger domain-containing protein 1 isoform X2 [Cicer arietinum].
XP_004976556.1	LOC101783976	659	eukaryota>viridiplantae	Setaria italica	PREDICTED: ankyrin repeat and zinc finger domain-containing protein 1 [Setaria italica].
XP_001767776.1	PHYPADRAFT_165800	752	eukaryota>viridiplantae	Physcomitrella patens	predicted protein [Physcomitrella patens].
OAY82497.1	ACMD2_19679	357	eukaryota>viridiplantae	Ananas comosus	hypothetical protein ACMD2_19679, partial [Ananas comosus].
XP_008780028.1	LOC103699796	655	eukaryota>viridiplantae	Phoenix dactylifera	PREDICTED: ankyrin repeat and zinc finger domain-containing protein 1 [Phoenix dactylifera].
XP_010277446.1	LOC104611881	711	eukaryota>viridiplantae	Nelumbo nucifera	PREDICTED: ankyrin repeat and zinc finger domain-containing protein 1 isoform X1 [Nelumbo nucifera].
XP_003743377.1	LOC100897251	559	eukaryota>metazoa	Metaseiulus occidentalis	PREDICTED: ankyrin repeat and zinc finger domain-containing protein 1-like [Metaseiulus occidentalis].
OAF67209.1	A3Q56_05034	485	eukaryota>metazoa	Intoshia linei	hypothetical protein A3Q56_05034 [Intoshia linei].
XP_012595649.1	ANKZF1	664	eukaryota>metazoa>chordata>vertebrata	Microcebus murinus	PREDICTED: ankyrin repeat and zinc finger domain-containing protein 1 isoform X3 [Microcebus murinus].
XP_005246720.1	ANKZF1	661	eukaryota>metazoa>chordata>vertebrata	Homo sapiens	PREDICTED: ankyrin repeat and zinc finger domain-containing protein 1 isoform X2 [Homo sapiens].
XP_009236404.1	ANKZF1	661	eukaryota>metazoa>chordata>vertebrata	Pongo abelii	PREDICTED: ankyrin repeat and zinc finger domain-containing protein 1 isoform X2 [Pongo abelii].
XP_011236998.1	Ankzf1	580	eukaryota>metazoa>chordata>vertebrata	Mus musculus	PREDICTED: ankyrin repeat and zinc finger domain-containing protein 1 isoform X2 [Mus musculus].
XP_010947113.1	LOC105064038	493	eukaryota>metazoa>chordata>vertebrata	Camelus bactrianus	PREDICTED: ankyrin repeat and zinc finger domain-containing protein 1-like [Camelus bactrianus].
XP_016859909.1	ANKZF1	660	eukaryota>metazoa>chordata>vertebrata	Homo sapiens	PREDICTED: ankyrin repeat and zinc finger domain-containing protein 1 isoform X3 [Homo sapiens].
XP_015145576.1	ANKZF1	657	eukaryota>metazoa>chordata>vertebrata	Gallus gallus	PREDICTED: ankyrin repeat and zinc finger domain-containing protein 1 isoform X3 [Gallus gallus].
XP_009509772.1	ANKZF1	571	eukaryota>metazoa>chordata>vertebrata	Phalacrocorax carbo	PREDICTED: ankyrin repeat and zinc finger domain-containing protein 1, partial [Phalacrocorax carbo].
XP_014711576.1	ANKZF1	508	eukaryota>metazoa>chordata>vertebrata	Equus asinus	PREDICTED: ankyrin repeat and zinc finger domain-containing protein 1 isoform X2 [Equus asinus].
XP_012583408.1	ANKZF1	513	eukaryota>metazoa>chordata>vertebrata	Condylura cristata	PREDICTED: ankyrin repeat and zinc finger domain-containing protein 1 isoform X2 [Condylura cristata].
EDL00376.1	mCG_133867	514	eukaryota>metazoa>chordata>vertebrata	Mus musculus	mCG133867, isoform CRA_a [Mus musculus].
XP_010334589.1	ANKZF1	514	eukaryota>metazoa>chordata>vertebrata	Saimiri boliviensis boliviensis	PREDICTED: ankyrin repeat and zinc finger domain-containing protein 1 isoform X2 [Saimiri boliviensis boliviensis].
XP_015419126.1	ANKZF1	688	eukaryota>metazoa>chordata>vertebrata	Myotis davidii	PREDICTED: ankyrin repeat and zinc finger domain-containing protein 1 isoform X2 [Myotis davidii].
EDL75397.1	rCG_23889	514	eukaryota>metazoa>chordata>vertebrata	Rattus norvegicus	ankyrin repeat containing protein RGD1359242, isoform CRA_b [Rattus norvegicus].
NP_001269721.1	ANKZF1	516	eukaryota>metazoa>chordata>vertebrata	Homo sapiens	ankyrin repeat and zinc finger domain-containing protein 1 isoform 2 [Homo sapiens].
XP_011787595.1	ANKZF1	516	eukaryota>metazoa>chordata>vertebrata	Colobus angolensis palliatus	PREDICTED: ankyrin repeat and zinc finger domain-containing protein 1 isoform X2 [Colobus angolensis palliatus].
XP_012497980.1	ANKZF1	665	eukaryota>metazoa>chordata>vertebrata	Propithecus coquereli	PREDICTED: ankyrin repeat and zinc finger domain-containing protein 1 isoform X2 [Propithecus coquereli].
XP_010610146.1	Ankzf1	516	eukaryota>metazoa>chordata>vertebrata	Fukomys damarensis	PREDICTED: ankyrin repeat and zinc finger domain-containing protein 1 isoform X3 [Fukomys damarensis].
XP_011739038.1	ANKZF1	516	eukaryota>metazoa>chordata>vertebrata	Macaca nemestrina	PREDICTED: ankyrin repeat and zinc finger domain-containing protein 1 isoform X3 [Macaca nemestrina].
XP_011820037.1	ANKZF1	516	eukaryota>metazoa>chordata>vertebrata	Mandrillus leucophaeus	PREDICTED: ankyrin repeat and zinc finger domain-containing protein 1 isoform X2 [Mandrillus leucophaeus].
XP_009442535.1	ANKZF1	516	eukaryota>metazoa>chordata>vertebrata	Pan troglodytes	PREDICTED: ankyrin repeat and zinc finger domain-containing protein 1 isoform X6 [Pan troglodytes].
XP_009181390.1	ANKZF1	516	eukaryota>metazoa>chordata>vertebrata	Papio anubis	PREDICTED: ankyrin repeat and zinc finger domain-containing protein 1 isoform X3 [Papio anubis].
XP_010600201.1	ANKZF1	516	eukaryota>metazoa>chordata>vertebrata	Loxodonta africana	PREDICTED: ankyrin repeat and zinc finger domain-containing protein 1 isoform X2 [Loxodonta africana].
XP_011918215.1	ANKZF1	516	eukaryota>metazoa>chordata>vertebrata	Cercocebus atys	PREDICTED: ankyrin repeat and zinc finger domain-containing protein 1 isoform X2 [Cercocebus atys].
XP_008688661.1	ANKZF1	538	eukaryota>metazoa>chordata>vertebrata	Ursus maritimus	PREDICTED: ankyrin repeat and zinc finger domain-containing protein 1 isoform X4 [Ursus maritimus].
XP_008528194.1	ANKZF1	513	eukaryota>metazoa>chordata>vertebrata	Equus przewalskii	PREDICTED: ankyrin repeat and zinc finger domain-containing protein 1 isoform X2 [Equus przewalskii].
XP_001925674.2	ANKZF1	718	eukaryota>metazoa>chordata>vertebrata	Sus scrofa	PREDICTED: ankyrin repeat and zinc finger domain-containing protein 1 isoform X3 [Sus scrofa].
NP_001014241.1	Ankzf1	722	eukaryota>metazoa>chordata>vertebrata	Rattus norvegicus	ankyrin repeat and zinc finger domain-containing protein 1 [Rattus norvegicus].
XP_012301343.1	ANKZF1	724	eukaryota>metazoa>chordata>vertebrata	Aotus nancymaae	PREDICTED: ankyrin repeat and zinc finger domain-containing protein 1 isoform X3 [Aotus nancymaae].
XP_009573054.1	ANKZF1	520	eukaryota>metazoa>chordata>vertebrata	Fulmarus glacialis	PREDICTED: ankyrin repeat and zinc finger domain-containing protein 1 [Fulmarus glacialis].
XP_015336901.1	Ankzf1	727	eukaryota>metazoa>chordata>vertebrata	Marmota marmota marmota	PREDICTED: ankyrin repeat and zinc finger domain-containing protein 1 isoform X2 [Marmota marmota marmota].
OCA23982.1	XENTR_v90024536mg	646	eukaryota>metazoa>chordata>vertebrata	Xenopus tropicalis	hypothetical protein XENTR_v90024536mg [Xenopus tropicalis].
XP_017335773.1	ankzf1	695	eukaryota>metazoa>chordata>vertebrata>actinopterygii	Ictalurus punctatus	PREDICTED: ankyrin repeat and zinc finger domain-containing protein 1 isoform X3 [Ictalurus punctatus].
KOX70984.1	WN51_03525	697	eukaryota>metazoa>hexapoda	Melipona quadrifasciata	Ankyrin repeat and zinc finger domain-containing protein 1 [Melipona quadrifasciata].
XP_012246036.1	LOC100744548	740	eukaryota>metazoa>hexapoda	Bombus impatiens	PREDICTED: ankyrin repeat and zinc finger domain-containing protein 1-like isoform X2 [Bombus impatiens].
XP_011063459.1	LOC105151449	774	eukaryota>metazoa>hexapoda	Acromyrmex echinatior	PREDICTED: ankyrin repeat and zinc finger domain-containing protein 1-like isoform X2 [Acromyrmex echinatior].
EFZ22210.1	SINV_80223	778	eukaryota>metazoa>hexapoda	Solenopsis invicta	hypothetical protein SINV_80223, partial [Solenopsis invicta].
EYB86764.1	Y032_0273g972	580	eukaryota>metazoa>nematoda	Ancylostoma ceylanicum	hypothetical protein Y032_0273g972 [Ancylostoma ceylanicum].
XP_001894846.1	Bm1_16945	610	eukaryota>metazoa>nematoda	Brugia malayi	hypothetical protein [Brugia malayi].
OAE25219.1	AXG93_2210s1030	779	eukaryota>viridiplantae	Marchantia polymorpha subsp. polymorpha	hypothetical protein AXG93_2210s1030 [Marchantia polymorpha subsp. polymorpha].
KZV41613.1	F511_15382	734	eukaryota>viridiplantae	Dorcoceras hygrometricum	ankyrin repeat and zinc finger domain-containing protein 1-like [Dorcoceras hygrometricum].
KHN29437.1	glysoja_005161	693	eukaryota>viridiplantae	Glycine soja	Ankyrin repeat and zinc finger domain-containing protein 1 [Glycine soja].
CBI38788.3	VIT_00027532001	666	eukaryota>viridiplantae	Vitis vinifera	unnamed protein product, partial [Vitis vinifera].
DAA36387.1	ZEAMMB73_368644	673	eukaryota>viridiplantae	Zea mays	TPA: hypothetical protein ZEAMMB73_368644 [Zea mays].
CEM27100.1	Vbra_663	672	eukaryota>alveolata	Vitrella brassicaformis CCMP3155	unnamed protein product [Vitrella brassicaformis CCMP3155].

##;similar to the above core architecture, with VATC and VIM inverted

CBN79933.1	Esi_0015_0120	718	eukaryota>stramenopiles	Ectocarpus siliculosus	conserved unknown protein [Ectocarpus siliculosus].
XP_002999091.1	PITG_05448	548	eukaryota>stramenopiles	Phytophthora infestans T30-4	conserved hypothetical protein [Phytophthora infestans T30-4].
XP_008870600.1	H310_07086	601	eukaryota>stramenopiles	Aphanomyces invadans	hypothetical protein H310_07086 [Aphanomyces invadans].
XP_009519082.1	PHYSODRAFT_556459	565	eukaryota>stramenopiles	Phytophthora sojae	hypothetical protein PHYSODRAFT_556459 [Phytophthora sojae].
KUF80018.1	AM587_10017611	586	eukaryota>stramenopiles	Phytophthora nicotianae	vms-1 protein [Phytophthora nicotianae].
CEG35171.1	PHALS_13923	586	eukaryota>stramenopiles	Plasmopara halstedii	Ankyrin repeat protein [Plasmopara halstedii].
XP_008892034.1	PPTG_02611	586	eukaryota>stramenopiles	Phytophthora parasitica INRA-310	hypothetical protein PPTG_02611 [Phytophthora parasitica INRA-310].
ETK89597.1	L915_06378	586	eukaryota>stramenopiles	Phytophthora parasitica	hypothetical protein L915_06378 [Phytophthora parasitica].
ETI49713.1	F443_06512	586	eukaryota>stramenopiles	Phytophthora parasitica P1569	hypothetical protein F443_06512 [Phytophthora parasitica P1569].
XP_009826525.1	H257_03915	597	eukaryota>stramenopiles	Aphanomyces astaci	hypothetical protein H257_03915 [Aphanomyces astaci].
CCA22720.1	ALNC14_088630	587	eukaryota>stramenopiles	Albugo laibachii Nc14	conserved hypothetical protein [Albugo laibachii Nc14].
XP_012206926.1	SPRG_11355	590	eukaryota>stramenopiles	Saprolegnia parasitica CBS 223.65	hypothetical protein SPRG_11355 [Saprolegnia parasitica CBS 223.65].
XP_002178737.1	PHATRDRAFT_44792	613	eukaryota>stramenopiles	Phaeodactylum tricornutum CCAP 1055/1	predicted protein [Phaeodactylum tricornutum CCAP 1055/1].
XP_002296773.1	THAPSDRAFT_25110	722	eukaryota>stramenopiles	Thalassiosira pseudonana CCMP1335	predicted protein [Thalassiosira pseudonana CCMP1335].
XP_009036381.1	AURANDRAFT_63449	575	eukaryota>stramenopiles	Aureococcus anophagefferens	hypothetical protein AURANDRAFT_63449 [Aureococcus anophagefferens].

#;lacking VIM motif?

EJK73680.1	THAOC_04683	716	eukaryota>stramenopiles	Thalassiosira oceanica	hypothetical protein THAOC_04683, partial [Thalassiosira oceanica].

#;further C-terminal fusion to calponin homology(CH) domain

CCI39829.1	BN9_006120	827	eukaryota>stramenopiles	Albugo candida	unnamed protein product [Albugo candida].

##;additional variants to the core architecture

#;N-terminal Sec7+PH domain fusion

EYB86761.1	Y032_0273g972	949	eukaryota>metazoa>nematoda	Ancylostoma ceylanicum	hypothetical protein Y032_0273g972 [Ancylostoma ceylanicum].
CDJ96277.1	HCOI_02001800	930	eukaryota>metazoa>nematoda	Haemonchus contortus	SEC7 and Pleckstrin homology and Ankyrin domain containing protein [Haemonchus contortus].
EYB86763.1	Y032_0273g972	958	eukaryota>metazoa>nematoda	Ancylostoma ceylanicum	hypothetical protein Y032_0273g972 [Ancylostoma ceylanicum].
CDJ90413.1	HCOI_01196000	959	eukaryota>metazoa>nematoda	Haemonchus contortus	SEC7 and Pleckstrin homology and Ankyrin domain containing protein [Haemonchus contortus].
EYB86762.1	Y032_0273g972	976	eukaryota>metazoa>nematoda	Ancylostoma ceylanicum	hypothetical protein Y032_0273g972 [Ancylostoma ceylanicum].

#;C-terminal transmembrane-region

XP_015692140.1	LOC102707668	970	eukaryota>viridiplantae	Oryza brachyantha	PREDICTED: ankyrin repeat and zinc finger domain-containing protein 1 [Oryza brachyantha].
EMT33432.1	F775_09928	924	eukaryota>viridiplantae	Aegilops tauschii	Ankyrin repeat and zinc finger domain-containing protein 1 [Aegilops tauschii].
XP_008671135.1	LOC103648441	1263	eukaryota>viridiplantae	Zea mays	PREDICTED: uncharacterized protein LOC103648441 [Zea mays].
XP_010919422.1	LOC105043544	1261	eukaryota>viridiplantae	Elaeis guineensis	PREDICTED: uncharacterized protein LOC105043544 [Elaeis guineensis].

#;N-terminal transmembrane region

EFN64724.1	EAG_08850	1013	eukaryota>metazoa>hexapoda	Camponotus floridanus	Ankyrin repeat and zinc finger domain-containing protein 1 [Camponotus floridanus].
XP_006608123.1	LOC102673839	979	eukaryota>metazoa>hexapoda	Apis dorsata	PREDICTED: ankyrin repeat and zinc finger domain-containing protein 1-like [Apis dorsata].

##;in the below cases, Vms1/ANKZF1-like proteins otherwise largely retain the core architecture lack the VATC domain.

KGQ10041.1	BBAD15_g4608	1330	eukaryota>fungi>ascomycota	Beauveria bassiana D1-5	VMS1 protein [Beauveria bassiana D1-5].
XP_007878622.1	PFL1_02919	955	eukaryota>fungi>basidiomycota	Anthracocystis flocculosa PF-1	hypothetical protein PFL1_02919 [Anthracocystis flocculosa PF-1].
OAJ07793.1	A4X13_g235	885	eukaryota>fungi>basidiomycota	Tilletia indica	hypothetical protein A4X13_g235 [Tilletia indica].
XP_016294198.1	PSEUBRA_SCAF12g01781	758	eukaryota>fungi>basidiomycota	Kalmanozyma brasiliensis GHG001	hypothetical protein PSEUBRA_SCAF12g01781 [Kalmanozyma brasiliensis GHG001].
KOS16572.1	Malapachy_3659	773	eukaryota>fungi>basidiomycota	Malassezia pachydermatis	hypothetical protein Malapachy_3659 [Malassezia pachydermatis].
CEH18795.1	CBOM_05504	776	eukaryota>fungi>basidiomycota	Ceraceosorus bombacis	Ankyrin repeat protein [Ceraceosorus bombacis].
CCU98346.1	MSY001_1052	639	eukaryota>fungi>basidiomycota	Malassezia sympodialis ATCC 42132	unnamed protein product [Malassezia sympodialis ATCC 42132].
OAJ14610.1	A4X09_g5366	884	eukaryota>fungi>basidiomycota	Tilletia walkeri	hypothetical protein A4X09_g5366 [Tilletia walkeri].
XP_013240889.1	K437DRAFT_250949	830	eukaryota>fungi>basidiomycota	Tilletiaria anomala UBC 951	hypothetical protein K437DRAFT_250949 [Tilletiaria anomala UBC 951].
XP_001730961.1	MGL_1960	860	eukaryota>fungi>basidiomycota	Malassezia globosa CBS 7966	hypothetical protein MGL_1960 [Malassezia globosa CBS 7966].
XP_011391271.1	UMAG_04964	870	eukaryota>fungi>basidiomycota	Ustilago maydis 521	hypothetical protein UMAG_04964 [Ustilago maydis 521].
CCF50116.1	UHOR_07101	860	eukaryota>fungi>basidiomycota	Ustilago hordei	uncharacterized protein UHOR_07101 [Ustilago hordei].
CDI55502.1	BN887_03894	860	eukaryota>fungi>basidiomycota	Melanopsichium pennsylvanicum 4	conserved hypothetical protein [Melanopsichium pennsylvanicum 4].
GAC71452.1	PANT_3c00034	864	eukaryota>fungi>basidiomycota	Moesziomyces antarcticus T-34	ankyrin repeat protein [Moesziomyces antarcticus T-34].
XP_012189502.1	PHSY_003493	870	eukaryota>fungi>basidiomycota	Pseudozyma hubeiensis SY62	hypothetical protein PHSY_003493 [Pseudozyma hubeiensis SY62].
CBQ69379.1	sr15844	869	eukaryota>fungi>basidiomycota	Sporisorium reilianum SRZ2	conserved hypothetical protein [Sporisorium reilianum SRZ2].
XP_014653611.1	PAN0_049c6424	863	eukaryota>fungi>basidiomycota	Moesziomyces antarcticus	C2H2 finger and ankyrin domain [Moesziomyces antarcticus].
ETS65345.1	PaG_00077	864	eukaryota>fungi>basidiomycota	Moesziomyces aphidis DSM 70725	hypothetical protein PaG_00077 [Moesziomyces aphidis DSM 70725].
CDR99428.1	SSCI20430.1	869	eukaryota>fungi>basidiomycota	Sporisorium scitamineum	hypothetical protein [Sporisorium scitamineum].
OAJ20743.1	A4X03_g4278	877	eukaryota>fungi>basidiomycota	Tilletia caries	hypothetical protein A4X03_g4278 [Tilletia caries].
XP_013771933.1	LOC106457096	898	eukaryota>metazoa	Limulus polyphemus	PREDICTED: ankyrin repeat and zinc finger domain-containing protein 1-like [Limulus polyphemus].
XP_011431698.1	LOC105331279	889	eukaryota>metazoa>mollusca	Crassostrea gigas	PREDICTED: ankyrin repeat and zinc finger domain-containing protein 1-like [Crassostrea gigas].
XP_009496375.1	H696_04223	879	eukaryota	Fonticula alba	hypothetical protein H696_04223 [Fonticula alba].
XP_009496376.1	H696_04223	835	eukaryota	Fonticula alba	hypothetical protein, variant 1 [Fonticula alba].
XP_009496377.1	H696_04223	658	eukaryota	Fonticula alba	hypothetical protein, variant 2 [Fonticula alba].
XP_007646379.1	Ankzf1	827	eukaryota>metazoa>chordata>vertebrata	Cricetulus griseus	PREDICTED: ankyrin repeat and zinc finger domain-containing protein 1 isoform X1 [Cricetulus griseus].
XP_007646380.1	Ankzf1	793	eukaryota>metazoa>chordata>vertebrata	Cricetulus griseus	PREDICTED: ankyrin repeat and zinc finger domain-containing protein 1 isoform X2 [Cricetulus griseus].
XP_016833698.1	Ankzf1	619	eukaryota>metazoa>chordata>vertebrata	Cricetulus griseus	PREDICTED: ankyrin repeat and zinc finger domain-containing protein 1 isoform X5 [Cricetulus griseus].
XP_007964543.1	ANKZF1	819	eukaryota>metazoa>chordata>vertebrata	Chlorocebus sabaeus	PREDICTED: ankyrin repeat and zinc finger domain-containing protein 1 isoform X1 [Chlorocebus sabaeus].
XP_007964544.1	ANKZF1	818	eukaryota>metazoa>chordata>vertebrata	Chlorocebus sabaeus	PREDICTED: ankyrin repeat and zinc finger domain-containing protein 1 isoform X2 [Chlorocebus sabaeus].
XP_007964545.1	ANKZF1	813	eukaryota>metazoa>chordata>vertebrata	Chlorocebus sabaeus	PREDICTED: ankyrin repeat and zinc finger domain-containing protein 1 isoform X3 [Chlorocebus sabaeus].
XP_007291064.1	MBM_03175	774	eukaryota>fungi>ascomycota	Marssonina brunnea f. sp. 'multigermtubi' MB_m1	ankyrin repeat and zinc finger domain-containing protein [Marssonina brunnea f. sp. 'multigermtubi' MB_m1].
KZZ93310.1	AAL_05695	710	eukaryota>fungi>ascomycota	Aschersonia aleyrodis RCEF 2490	ankyrin repeat and zinc finger domain-containing protein 1 [Aschersonia aleyrodis RCEF 2490].
XP_003336465.2	PGTG_18136	820	eukaryota>fungi>basidiomycota	Puccinia graminis f. sp. tritici CRL 75-36-700-3	hypothetical protein PGTG_18136 [Puccinia graminis f. sp. tritici CRL 75-36-700-3].
OAD06885.1	MUCCIDRAFT_107478	637	eukaryota>fungi	Mucor circinelloides f. lusitanicus CBS 277.49	C2H2-type zinc finger transcription factor [Mucor circinelloides f. lusitanicus CBS 277.49].
EPZ35195.1	O9G_004206	309	eukaryota>fungi	Rozella allomycis CSF55	Zinc finger, C2H2 domain-containing protein [Rozella allomycis CSF55].
KNE65431.1	AMAG_11060	608	eukaryota>fungi	Allomyces macrogynus ATCC 38327	hypothetical protein AMAG_11060 [Allomyces macrogynus ATCC 38327].
EIE84577.1	RO3G_09287	617	eukaryota>fungi	Rhizopus delemar RA 99-880	hypothetical protein RO3G_09287 [Rhizopus delemar RA 99-880].
CEI87338.1	RMCBS344292_01755	596	eukaryota>fungi	Rhizopus microsporus	hypothetical protein RMCBS344292_01755 [Rhizopus microsporus].
CEG79447.1	RMATCC62417_13913	595	eukaryota>fungi	Rhizopus microsporus	hypothetical protein RMATCC62417_13913 [Rhizopus microsporus].
CEG80858.1	RMATCC62417_15128	333	eukaryota>fungi	Rhizopus microsporus	hypothetical protein RMATCC62417_15128 [Rhizopus microsporus].
CDS14279.1	LRAMOSA06449	647	eukaryota>fungi	Lichtheimia ramosa	hypothetical protein LRAMOSA06449 [Lichtheimia ramosa].
KXN74193.1	CONCODRAFT_14944	644	eukaryota>fungi	Conidiobolus coronatus NRRL 28638	hypothetical protein CONCODRAFT_14944 [Conidiobolus coronatus NRRL 28638].
OAD77380.1	PHYBLDRAFT_154532	642	eukaryota>fungi	Phycomyces blakesleeanus NRRL 1555(-)	hypothetical protein PHYBLDRAFT_154532 [Phycomyces blakesleeanus NRRL 1555(-)].
CEP12209.1	PARPA_06142.1 scaffold 21287	638	eukaryota>fungi	Parasitella parasitica	hypothetical protein [Parasitella parasitica].
OBZ89160.1	A0J61_02790	615	eukaryota>fungi	Choanephora cucurbitarum	hypothetical protein A0J61_02790 [Choanephora cucurbitarum].
GAM87314.1	ANO11243_053370	682	eukaryota>fungi	fungal sp. No.11243	hypothetical protein ANO11243_053370 [fungal sp. No.11243].
GAN10941.1	MAM1_0433d10491	637	eukaryota>fungi	Mucor ambiguus	ankyrin repeat-containing protein [Mucor ambiguus].
KNE66544.1	AMAG_11674	607	eukaryota>fungi	Allomyces macrogynus ATCC 38327	hypothetical protein AMAG_11674 [Allomyces macrogynus ATCC 38327].
CEG79448.1	RMATCC62417_13913	429	eukaryota>fungi	Rhizopus microsporus	hypothetical protein RMATCC62417_13913 [Rhizopus microsporus].
EPB87277.1	HMPREF1544_05898	637	eukaryota>fungi	Mucor circinelloides f. circinelloides 1006PhL	hypothetical protein HMPREF1544_05898 [Mucor circinelloides f. circinelloides 1006PhL].
SAM04274.1	ABSGL_10134.1 scaffold 11786	708	eukaryota>fungi	Absidia glauca	hypothetical protein [Absidia glauca].
KGU21473.1	MGM_05924	537	eukaryota>fungi>ascomycota	Candida albicans P75063	hypothetical protein MGM_05924 [Candida albicans P75063].
KHC65019.1	MGE_02467	537	eukaryota>fungi>ascomycota	Candida albicans P75010	hypothetical protein MGE_02467 [Candida albicans P75010].
KHC52966.1	MEW_02413	537	eukaryota>fungi>ascomycota	Candida albicans P60002	hypothetical protein MEW_02413 [Candida albicans P60002].
KGR11441.1	MG5_02460	537	eukaryota>fungi>ascomycota	Candida albicans P57072	hypothetical protein MG5_02460 [Candida albicans P57072].
KGU34144.1	MGK_02463	537	eukaryota>fungi>ascomycota	Candida albicans P57055	hypothetical protein MGK_02463 [Candida albicans P57055].
KHC56976.1	MGC_02463	537	eukaryota>fungi>ascomycota	Candida albicans P37039	hypothetical protein MGC_02463 [Candida albicans P37039].
KGQ95625.1	MEU_02464	537	eukaryota>fungi>ascomycota	Candida albicans P37005	hypothetical protein MEU_02464 [Candida albicans P37005].
KGQ88301.1	MEO_02467	537	eukaryota>fungi>ascomycota	Candida albicans P94015	hypothetical protein MEO_02467 [Candida albicans P94015].
KGQ99660.1	MG1_02487	537	eukaryota>fungi>ascomycota	Candida albicans GC75	hypothetical protein MG1_02487 [Candida albicans GC75].
KHC44780.1	W5O_02472	537	eukaryota>fungi>ascomycota	Candida albicans Ca6	hypothetical protein W5O_02472 [Candida albicans Ca6].
EPE08011.1	F503_00794	727	eukaryota>fungi>ascomycota	Ophiostoma piceae UAMH 11346	ankyrin repeat-containing protein [Ophiostoma piceae UAMH 11346].
EPQ66745.1	BGT96224_3028	543	eukaryota>fungi>ascomycota	Blumeria graminis f. sp. tritici 96224	Zinc finger protein transcription factor [Blumeria graminis f. sp. tritici 96224].
CCF44017.1	CH063_13556	543	eukaryota>fungi>ascomycota	Colletotrichum higginsianum	ankyrin repeat and zinc finger protein, partial [Colletotrichum higginsianum].
KHC38923.1	MGQ_02456	537	eukaryota>fungi>ascomycota	Candida albicans P76067	hypothetical protein MGQ_02456 [Candida albicans P76067].
XP_001804022.1	SNOG_13819	437	eukaryota>fungi>ascomycota	Parastagonospora nodorum SN15	hypothetical protein SNOG_13819 [Parastagonospora nodorum SN15].
KFZ20644.1	V502_03091	698	eukaryota>fungi>ascomycota	Pseudogymnoascus sp. VKM F-4520 (FW-2644)	hypothetical protein V502_03091 [Pseudogymnoascus sp. VKM F-4520 (FW-2644)].
KFZ01221.1	V500_00840	698	eukaryota>fungi>ascomycota	Pseudogymnoascus sp. VKM F-4518 (FW-2643)	hypothetical protein V500_00840 [Pseudogymnoascus sp. VKM F-4518 (FW-2643)].
XP_007580462.1	UCRNP2_1141	552	eukaryota>fungi>ascomycota	Neofusicoccum parvum UCRNP2	putative ankyrin repeat and zinc finger domain containing protein 1 protein [Neofusicoccum parvum UCRNP2].
KFY26077.1	V493_04286	696	eukaryota>fungi>ascomycota	Pseudogymnoascus sp. VKM F-4281 (FW-2241)	hypothetical protein V493_04286 [Pseudogymnoascus sp. VKM F-4281 (FW-2241)].
OBA15994.1	OGAPODRAFT_88647	555	eukaryota>fungi>ascomycota	Ogataea polymorpha	hypothetical protein OGAPODRAFT_88647 [Ogataea polymorpha].
CRK19340.1	BN1723_002551	695	eukaryota>fungi>ascomycota	Verticillium longisporum	hypothetical protein BN1723_002551 [Verticillium longisporum].
KKY16473.1	UCDDS831_g06972	555	eukaryota>fungi>ascomycota	Diplodia seriata	putative ankyrin repeat and zinc finger domain containing protein 1 [Diplodia seriata].
XP_003651538.1	THITE_2149790	694	eukaryota>fungi>ascomycota	Thielavia terrestris NRRL 8126	hypothetical protein THITE_2149790 [Thielavia terrestris NRRL 8126].
KXX73758.1	MMYC01_210120	432	eukaryota>fungi>ascomycota	Madurella mycetomatis	Protein VMS1 [Madurella mycetomatis].
EGA75560.1	AWRI796_0797	561	eukaryota>fungi>ascomycota	Saccharomyces cerevisiae AWRI796	YDR049W-like protein [Saccharomyces cerevisiae AWRI796].
XP_003017088.1	ARB_03964	564	eukaryota>fungi>ascomycota	Arthroderma benhamiae CBS 112371	hypothetical protein ARB_03964 [Arthroderma benhamiae CBS 112371].
GAQ44112.1	ABL_06773	691	eukaryota>fungi>ascomycota	Aspergillus niger	C2H2 finger and ankyrin domain protein [Aspergillus niger].
XP_014171858.1	CMQ_2425	690	eukaryota>fungi>ascomycota	Grosmannia clavigera kw1407	c2h2 finger and ankyrin domain protein [Grosmannia clavigera kw1407].
KKF95880.1	CFO_g1758	429	eukaryota>fungi>ascomycota	Ceratocystis platani	hypothetical protein CFO_g1758 [Ceratocystis platani].
KGU27825.1	MG7_02482	537	eukaryota>fungi>ascomycota	Candida albicans P34048	hypothetical protein MG7_02482 [Candida albicans P34048].
CRK23406.1	BN1708_003675	687	eukaryota>fungi>ascomycota	Verticillium longisporum	hypothetical protein BN1708_003675, partial [Verticillium longisporum].
OAA62460.1	SPI_04000	730	eukaryota>fungi>ascomycota	Sporothrix insectorum RCEF 264	c2h2 finger and ankyrin domain protein [Sporothrix insectorum RCEF 264].
XP_003840980.1	LEMA_P106320.1	686	eukaryota>fungi>ascomycota	Leptosphaeria maculans JN3	hypothetical protein LEMA_P106320.1 [Leptosphaeria maculans JN3].
XP_002418925.1	CD36_24520	568	eukaryota>fungi>ascomycota	Candida dubliniensis CD36	zinc finger-containing protein [Candida dubliniensis CD36].
GAA91314.1	AKAW_09428	685	eukaryota>fungi>ascomycota	Aspergillus kawachii IFO 4308	C2H2 finger and ankyrin domain protein, partial [Aspergillus kawachii IFO 4308].
XP_007673955.1	BAUCODRAFT_381036	685	eukaryota>fungi>ascomycota	Baudoinia panamericana UAMH 10762	hypothetical protein BAUCODRAFT_381036 [Baudoinia panamericana UAMH 10762].
KGR13297.1	MG3_02482	537	eukaryota>fungi>ascomycota	Candida albicans P78048	hypothetical protein MG3_02482 [Candida albicans P78048].
EME45637.1	DOTSEDRAFT_52861	683	eukaryota>fungi>ascomycota	Dothistroma septosporum NZE10	hypothetical protein DOTSEDRAFT_52861 [Dothistroma septosporum NZE10].
XP_009656080.1	VDAG_01756	682	eukaryota>fungi>ascomycota	Verticillium dahliae VdLs.17	ankyrin repeat-containing protein [Verticillium dahliae VdLs.17].
EXK31292.1	FOMG_13015	682	eukaryota>fungi>ascomycota	Fusarium oxysporum f. sp. melonis 26406	hypothetical protein FOMG_13015 [Fusarium oxysporum f. sp. melonis 26406].
EWZ39306.1	FOZG_08442	682	eukaryota>fungi>ascomycota	Fusarium oxysporum Fo47	hypothetical protein FOZG_08442 [Fusarium oxysporum Fo47].
KGU11361.1	MEQ_02443	537	eukaryota>fungi>ascomycota	Candida albicans P87	hypothetical protein MEQ_02443 [Candida albicans P87].
EXA40111.1	FOVG_09050	682	eukaryota>fungi>ascomycota	Fusarium oxysporum f. sp. pisi HDV247	hypothetical protein FOVG_09050 [Fusarium oxysporum f. sp. pisi HDV247].
EXK98235.1	FOQG_01208	682	eukaryota>fungi>ascomycota	Fusarium oxysporum f. sp. raphani 54005	hypothetical protein FOQG_01208 [Fusarium oxysporum f. sp. raphani 54005].
EWZ83735.1	FOWG_12668	682	eukaryota>fungi>ascomycota	Fusarium oxysporum f. sp. lycopersici MN25	hypothetical protein FOWG_12668 [Fusarium oxysporum f. sp. lycopersici MN25].
KZF23012.1	L228DRAFT_247454	682	eukaryota>fungi>ascomycota	Xylona heveae TC161	hypothetical protein L228DRAFT_247454 [Xylona heveae TC161].
EXL95586.1	FOIG_12074	682	eukaryota>fungi>ascomycota	Fusarium oxysporum f. sp. cubense tropical race 4 54006	hypothetical protein FOIG_12074 [Fusarium oxysporum f. sp. cubense tropical race 4 54006].
EXM23485.1	FOTG_09329	682	eukaryota>fungi>ascomycota	Fusarium oxysporum f. sp. vasinfectum 25433	hypothetical protein FOTG_09329 [Fusarium oxysporum f. sp. vasinfectum 25433].
EWY89161.1	FOYG_10095	681	eukaryota>fungi>ascomycota	Fusarium oxysporum FOSC 3-a	hypothetical protein FOYG_10095 [Fusarium oxysporum FOSC 3-a].
EMG49408.1	G210_5821	569	eukaryota>fungi>ascomycota	Candida maltosa Xu316	hypothetical protein G210_5821 [Candida maltosa Xu316].
CCE44600.1	CPAR2_404030	570	eukaryota>fungi>ascomycota	Candida parapsilosis	hypothetical protein CPAR2_404030 [Candida parapsilosis].
KXL46479.1	FE78DRAFT_146100	678	eukaryota>fungi>ascomycota	Acidomyces richmondensis	hypothetical protein FE78DRAFT_146100 [Acidomyces richmondensis].
EKG18330.1	MPH_04412	571	eukaryota>fungi>ascomycota	Macrophomina phaseolina MS6	hypothetical protein MPH_04412 [Macrophomina phaseolina MS6].
CCE32742.1	CPUR_06606	674	eukaryota>fungi>ascomycota	Claviceps purpurea 20.1	uncharacterized protein CPUR_06606 [Claviceps purpurea 20.1].
XP_016758480.1	SEPMUDRAFT_48804	674	eukaryota>fungi>ascomycota	Sphaerulina musiva SO2202	hypothetical protein SEPMUDRAFT_48804 [Sphaerulina musiva SO2202].
XP_717170.1	CaO19.5368	537	eukaryota>fungi>ascomycota	Candida albicans SC5314	hypothetical protein CaO19.5368 [Candida albicans SC5314].
CEP22675.1	BN1211_3078	579	eukaryota>fungi>ascomycota	Cyberlindnera jadinii	hypothetical protein BN1211_3078 [Cyberlindnera jadinii].
XP_003660031.1	MYCTH_2297796	674	eukaryota>fungi>ascomycota	Myceliophthora thermophila ATCC 42464	hypothetical protein MYCTH_2297796 [Myceliophthora thermophila ATCC 42464].
EXL83892.1	FOPG_03490	674	eukaryota>fungi>ascomycota	Fusarium oxysporum f. sp. conglutinans race 2 54008	hypothetical protein FOPG_03490 [Fusarium oxysporum f. sp. conglutinans race 2 54008].
KDB17739.1	UV8b_1623	673	eukaryota>fungi>ascomycota	Ustilaginoidea virens	ankyrin repeat and zinc finger domain containing protein 1 [Ustilaginoidea virens].
XP_007795140.1	UCREL1_7254	386	eukaryota>fungi>ascomycota	Eutypa lata UCREL1	putative ankyrin repeat-containing protein [Eutypa lata UCREL1].
EEQ47564.1	CAWG_06144	537	eukaryota>fungi>ascomycota	Candida albicans WO-1	conserved hypothetical protein [Candida albicans WO-1].
KJX92563.1	TI39_contig5841g00023	673	eukaryota>fungi>ascomycota	Zymoseptoria brevis	ankyrin repeat and zinc finger domain-containing protein [Zymoseptoria brevis].
KFY62651.1	V496_04481	673	eukaryota>fungi>ascomycota	Pseudogymnoascus sp. VKM F-4515 (FW-2607)	hypothetical protein V496_04481 [Pseudogymnoascus sp. VKM F-4515 (FW-2607)].
KEQ67866.1	M437DRAFT_71646	672	eukaryota>fungi>ascomycota	Aureobasidium melanogenum CBS 110374	hypothetical protein M437DRAFT_71646 [Aureobasidium melanogenum CBS 110374].
CCU77460.1	BGHDH14_bgh02844	532	eukaryota>fungi>ascomycota	Blumeria graminis f. sp. hordei DH14	hypothetical protein BGHDH14_bgh02844 [Blumeria graminis f. sp. hordei DH14].
XP_011125341.1	AOL_s00112g9	517	eukaryota>fungi>ascomycota	Arthrobotrys oligospora ATCC 24927	hypothetical protein AOL_s00112g9 [Arthrobotrys oligospora ATCC 24927].
XP_013431577.1	M436DRAFT_34907	672	eukaryota>fungi>ascomycota	Aureobasidium namibiae CBS 147.97	hypothetical protein M436DRAFT_34907 [Aureobasidium namibiae CBS 147.97].
XP_001222486.1	CHGG_06391	736	eukaryota>fungi>ascomycota	Chaetomium globosum CBS 148.51	hypothetical protein CHGG_06391 [Chaetomium globosum CBS 148.51].
KEQ78627.1	M438DRAFT_329605	672	eukaryota>fungi>ascomycota	Aureobasidium pullulans EXF-150	hypothetical protein M438DRAFT_329605 [Aureobasidium pullulans EXF-150].
GAP88063.1	SAMD00023353_2701040	672	eukaryota>fungi>ascomycota	Rosellinia necatrix	putative ankyrin repeat and zinc finger domain-containing protein [Rosellinia necatrix].
XP_013342259.1	AUEXF2481DRAFT_30813	671	eukaryota>fungi>ascomycota	Aureobasidium subglaciale EXF-2481	hypothetical protein AUEXF2481DRAFT_30813 [Aureobasidium subglaciale EXF-2481].
KKA02997.1	D499_0D00450	586	eukaryota>fungi>ascomycota	Hanseniaspora uvarum DSM 2768	hypothetical protein D499_0D00450 [Hanseniaspora uvarum DSM 2768].
XP_001595365.1	SS1G_03454	671	eukaryota>fungi>ascomycota	Sclerotinia sclerotiorum 1980 UF-70	hypothetical protein SS1G_03454 [Sclerotinia sclerotiorum 1980 UF-70].
OBT56926.1	VE04_03006	670	eukaryota>fungi>ascomycota	Pseudogymnoascus sp. 24MN13	hypothetical protein VE04_03006 [Pseudogymnoascus sp. 24MN13].
KND97279.1	QG37_06499	587	eukaryota>fungi>ascomycota	Candida auris	hypothetical protein QG37_06499 [Candida auris].
XP_003681128.1	TDEL_0D03330	587	eukaryota>fungi>ascomycota	Torulaspora delbrueckii	hypothetical protein TDEL_0D03330 [Torulaspora delbrueckii].
KXT17494.1	AC579_3218	670	eukaryota>fungi>ascomycota	Pseudocercospora musae	hypothetical protein AC579_3218 [Pseudocercospora musae].
XP_012739934.1	GMDG_01536	669	eukaryota>fungi>ascomycota	Pseudogymnoascus destructans 20631-21	hypothetical protein GMDG_01536 [Pseudogymnoascus destructans 20631-21].
XP_001553481.1	BC1G_07890	669	eukaryota>fungi>ascomycota	Botrytis cinerea B05.10	hypothetical protein BC1G_07890 [Botrytis cinerea B05.10].
OBT83660.1	VE02_07656	669	eukaryota>fungi>ascomycota	Pseudogymnoascus sp. 03VT05	hypothetical protein VE02_07656 [Pseudogymnoascus sp. 03VT05].
OBT91575.1	VE01_10387	669	eukaryota>fungi>ascomycota	Pseudogymnoascus verrucosus	hypothetical protein VE01_10387 [Pseudogymnoascus verrucosus].
OBT72665.1	VF21_08542	669	eukaryota>fungi>ascomycota	Pseudogymnoascus sp. 05NY08	hypothetical protein VF21_08542 [Pseudogymnoascus sp. 05NY08].
CCX04744.1	PCON_03457	588	eukaryota>fungi>ascomycota	Pyronema omphalodes CBS 100304	Similar to VMS1 homolog C1827.04; acc. no. O74977 [Pyronema omphalodes CBS 100304].
XP_007924333.1	MYCFIDRAFT_65421	669	eukaryota>fungi>ascomycota	Cercospora fijiensis CIRAD86	hypothetical protein MYCFIDRAFT_65421 [Cercospora fijiensis CIRAD86].
KFY17481.1	V491_04991	669	eukaryota>fungi>ascomycota	Pseudogymnoascus sp. VKM F-3775	hypothetical protein V491_04991 [Pseudogymnoascus sp. VKM F-3775].
OBT70521.1	VE03_00350	669	eukaryota>fungi>ascomycota	Pseudogymnoascus sp. 23342-1-I1	hypothetical protein VE03_00350 [Pseudogymnoascus sp. 23342-1-I1].
EMR90441.1	BcDW1_864	669	eukaryota>fungi>ascomycota	Botrytis cinerea BcDW1	putative ankyrin repeat-containing protein [Botrytis cinerea BcDW1].
KLU83410.1	MAPG_02470	590	eukaryota>fungi>ascomycota	Magnaporthiopsis poae ATCC 64411	ankyrin repeat-containing protein [Magnaporthiopsis poae ATCC 64411].
CCG84658.1	TAPDE_005116	515	eukaryota>fungi>ascomycota	Taphrina deformans PYCC 5710	protein of unknown function [Taphrina deformans PYCC 5710].
KTW28254.1	T551_02673	594	eukaryota>fungi>ascomycota	Pneumocystis jirovecii RU7	hypothetical protein T551_02673 [Pneumocystis jirovecii RU7].
KTW25970.1	T552_03244	594	eukaryota>fungi>ascomycota	Pneumocystis carinii B80	hypothetical protein T552_03244 [Pneumocystis carinii B80].
OAX83618.1	ACJ72_02018	501	eukaryota>fungi>ascomycota	Emmonsia sp. CAC-2015a	hypothetical protein ACJ72_02018 [Emmonsia sp. CAC-2015a].
XP_009157498.1	HMPREF1120_05089	666	eukaryota>fungi>ascomycota	Exophiala dermatitidis NIH/UT8656	hypothetical protein HMPREF1120_05089 [Exophiala dermatitidis NIH/UT8656].
XP_003024060.1	TRV_01827	595	eukaryota>fungi>ascomycota	Trichophyton verrucosum HKI 0517	hypothetical protein TRV_01827 [Trichophyton verrucosum HKI 0517].
XP_003868625.1	CORT_0C03470	595	eukaryota>fungi>ascomycota	Candida orthopsilosis Co 90-125	hypothetical protein CORT_0C03470 [Candida orthopsilosis Co 90-125].
EIF46332.1	AWRI1499_3793	317	eukaryota>fungi>ascomycota	Brettanomyces bruxellensis AWRI1499	zinc finger protein [Brettanomyces bruxellensis AWRI1499].
OBT41878.1	VE00_07379	665	eukaryota>fungi>ascomycota	Pseudogymnoascus sp. WSF 3629	hypothetical protein VE00_07379 [Pseudogymnoascus sp. WSF 3629].
XP_016588037.1	SPSK_08687	747	eukaryota>fungi>ascomycota	Sporothrix schenckii 1099-18	c2h2 finger and ankyrin domain protein [Sporothrix schenckii 1099-18].
CAK47847.1	An03g04080	665	eukaryota>fungi>ascomycota	Aspergillus niger	unnamed protein product [Aspergillus niger].
KFH42762.1	ACRE_065200	665	eukaryota>fungi>ascomycota	Acremonium chrysogenum ATCC 11550	VMS1 -like protein [Acremonium chrysogenum ATCC 11550].
XP_002172216.1	SJAG_00948	597	eukaryota>fungi>ascomycota	Schizosaccharomyces japonicus yFS275	hypothetical protein SJAG_00948 [Schizosaccharomyces japonicus yFS275].
XP_006691353.1	CTHT_0008230	665	eukaryota>fungi>ascomycota	Chaetomium thermophilum var. thermophilum DSM 1495	hypothetical protein CTHT_0008230 [Chaetomium thermophilum var. thermophilum DSM 1495].
XP_006690086.1	CANTEDRAFT_128572	597	eukaryota>fungi>ascomycota	Candida tenuis ATCC 10573	hypothetical protein CANTEDRAFT_128572 [Candida tenuis ATCC 10573].
KMU72652.1	CISG_09745	664	eukaryota>fungi>ascomycota	Coccidioides immitis RMSCC 3703	ankyrin repeat and zinc finger domain- containing protein 1 [Coccidioides immitis RMSCC 3703].
ESZ96331.1	SBOR_3263	664	eukaryota>fungi>ascomycota	Sclerotinia borealis F-4157	hypothetical protein SBOR_3263 [Sclerotinia borealis F-4157].
EGC44906.1	HCEG_04121	664	eukaryota>fungi>ascomycota	Histoplasma capsulatum H88	ankyrin repeat protein [Histoplasma capsulatum H88].
XP_010761420.1	PADG_05808	664	eukaryota>fungi>ascomycota	Paracoccidioides brasiliensis Pb18	hypothetical protein PADG_05808 [Paracoccidioides brasiliensis Pb18].
KMP03255.1	CIRG_02946	664	eukaryota>fungi>ascomycota	Coccidioides immitis RMSCC 2394	ankyrin repeat and zinc finger domain-containing protein 1 [Coccidioides immitis RMSCC 2394].
XP_004199215.1	GNLVRS01_PISO0J16245g	598	eukaryota>fungi>ascomycota	Millerozyma farinosa CBS 7064	Piso0_002633 [Millerozyma farinosa CBS 7064].
KIN07946.1	OIDMADRAFT_151872	663	eukaryota>fungi>ascomycota	Oidiodendron maius Zn	hypothetical protein OIDMADRAFT_151872 [Oidiodendron maius Zn].
EGE06582.1	TEQG_05580	662	eukaryota>fungi>ascomycota	Trichophyton equinum CBS 127.97	ankyrin repeat and zinc finger domain-containing protein 1 [Trichophyton equinum CBS 127.97].
EEH23282.1	PABG_05493	662	eukaryota>fungi>ascomycota	Paracoccidioides brasiliensis Pb03	hypothetical protein PABG_05493 [Paracoccidioides brasiliensis Pb03].
XP_008076409.1	GLAREA_09257	662	eukaryota>fungi>ascomycota	Glarea lozoyensis ATCC 20868	Ankyrin repeat-containing protein [Glarea lozoyensis ATCC 20868].
XP_002790770.2	PAAG_07307	661	eukaryota>fungi>ascomycota	Paracoccidioides lutzii Pb01	ankyrin repeat-containing protein [Paracoccidioides lutzii Pb01].
KGK40418.1	JL09_g473	598	eukaryota>fungi>ascomycota	Pichia kudriavzevii	hypothetical protein JL09_g473 [Pichia kudriavzevii].
EGD92468.1	TESG_00043	661	eukaryota>fungi>ascomycota	Trichophyton tonsurans CBS 112818	ankyrin domain-containing protein [Trichophyton tonsurans CBS 112818].
XP_004200068.1	GNLVRS01_PISO0I14440g	598	eukaryota>fungi>ascomycota	Millerozyma farinosa CBS 7064	Piso0_002633 [Millerozyma farinosa CBS 7064].
XP_003854202.1	MYCGRDRAFT_70436	661	eukaryota>fungi>ascomycota	Zymoseptoria tritici IPO323	hypothetical protein MYCGRDRAFT_70436 [Zymoseptoria tritici IPO323].
CDR37716.1	CYFA0S_01e15610g	598	eukaryota>fungi>ascomycota	Cyberlindnera fabianii	CYFA0S01e15610g1_1 [Cyberlindnera fabianii].
NP_588550.1	SPCC1827.04	600	eukaryota>fungi>ascomycota	Schizosaccharomyces pombe 972h-	VMS1-like protein quality control [Schizosaccharomyces pombe 972h-].
KHJ32184.1	EV44_g1509	661	eukaryota>fungi>ascomycota	Erysiphe necator	putative ankyrin repeat and zinc finger domain-containing protein [Erysiphe necator].
XP_007779808.1	W97_03723	661	eukaryota>fungi>ascomycota	Coniosporium apollinis CBS 100218	hypothetical protein W97_03723 [Coniosporium apollinis CBS 100218].
XP_007374235.1	SPAPADRAFT_137135	602	eukaryota>fungi>ascomycota	Spathaspora passalidarum NRRL Y-27907	hypothetical protein SPAPADRAFT_137135 [Spathaspora passalidarum NRRL Y-27907].
OAL48418.1	IQ07DRAFT_571089	660	eukaryota>fungi>ascomycota	Pyrenochaeta sp. DS3sAY3a	hypothetical protein IQ07DRAFT_571089 [Pyrenochaeta sp. DS3sAY3a].
GAO51568.1	G7K_5667-t1	603	eukaryota>fungi>ascomycota	Saitoella complicata NRRL Y-17804	hypothetical protein G7K_5667-t1 [Saitoella complicata NRRL Y-17804].
GAO14843.1	UVI_02005700	660	eukaryota>fungi>ascomycota	Ustilaginoidea virens	hypothetical protein UVI_02005700 [Ustilaginoidea virens].
OBA27501.1	HANVADRAFT_52249	603	eukaryota>fungi>ascomycota	Hanseniaspora valbyensis NRRL Y-1626	hypothetical protein HANVADRAFT_52249 [Hanseniaspora valbyensis NRRL Y-1626].
XP_009227538.1	GGTG_11388	660	eukaryota>fungi>ascomycota	Gaeumannomyces graminis var. tritici R3-111a-1	ankyrin repeat-containing protein [Gaeumannomyces graminis var. tritici R3-111a-1].
XP_001384952.2	PICST_60913	604	eukaryota>fungi>ascomycota	Scheffersomyces stipitis CBS 6054	hypothetical protein PICST_60913 [Scheffersomyces stipitis CBS 6054].
KXJ93953.1	Micbo1qcDRAFT_193259	659	eukaryota>fungi>ascomycota	Microdochium bolleyi	hypothetical protein Micbo1qcDRAFT_193259 [Microdochium bolleyi].
EZF32040.1	H101_04369	659	eukaryota>fungi>ascomycota	Trichophyton interdigitale H6	hypothetical protein H101_04369 [Trichophyton interdigitale H6].
XP_002547714.1	CTRG_02021	605	eukaryota>fungi>ascomycota	Candida tropicalis MYA-3404	conserved hypothetical protein [Candida tropicalis MYA-3404].
CDK26436.1	KUCA_T00002408001	606	eukaryota>fungi>ascomycota	Kuraishia capsulata CBS 1993	unnamed protein product [Kuraishia capsulata CBS 1993].
XP_504411.1	YALI0E26081g	606	eukaryota>fungi>ascomycota	Yarrowia lipolytica CLIB122	YALI0E26081p [Yarrowia lipolytica CLIB122].
XP_007840690.1	PFICI_13918	659	eukaryota>fungi>ascomycota	Pestalotiopsis fici W106-1	hypothetical protein PFICI_13918 [Pestalotiopsis fici W106-1].
XP_003234884.1	TERG_03934	658	eukaryota>fungi>ascomycota	Trichophyton rubrum CBS 118892	ankyrin domain-containing protein [Trichophyton rubrum CBS 118892].
XP_016209279.1	PV09_08948	658	eukaryota>fungi>ascomycota	Verruconis gallopava	hypothetical protein PV09_08948 [Verruconis gallopava].
XP_002621113.1	BDBG_08485	658	eukaryota>fungi>ascomycota	Blastomyces gilchristii SLH14081	C2H2 finger and ankyrin domain-containing protein [Blastomyces gilchristii SLH14081].
OAL69808.1	A7D00_5847	658	eukaryota>fungi>ascomycota	Trichophyton violaceum	ankyrin domain-containing protein [Trichophyton violaceum].
EGE82845.1	BDDG_05789	658	eukaryota>fungi>ascomycota	Blastomyces dermatitidis ATCC 18188	hypothetical protein BDDG_05789 [Blastomyces dermatitidis ATCC 18188].
EZG06191.1	H106_03976	658	eukaryota>fungi>ascomycota	Trichophyton rubrum CBS 735.88	hypothetical protein H106_03976 [Trichophyton rubrum CBS 735.88].
KUJ09284.1	LY89DRAFT_676095	658	eukaryota>fungi>ascomycota	Phialocephala scopiformis	hypothetical protein LY89DRAFT_676095 [Phialocephala scopiformis].
EQL32778.1	BDFG_05034	658	eukaryota>fungi>ascomycota	Blastomyces dermatitidis ATCC 26199	hypothetical protein BDFG_05034 [Blastomyces dermatitidis ATCC 26199].
XP_003173333.1	MGYG_03505	658	eukaryota>fungi>ascomycota	Microsporum gypseum CBS 118893	ankyrin repeat and zinc finger domain-containing protein 1 [Microsporum gypseum CBS 118893].
XP_007805255.1	EPUS_01152	657	eukaryota>fungi>ascomycota	Endocarpon pusillum Z07020	hypothetical protein EPUS_01152 [Endocarpon pusillum Z07020].
EEH10432.1	HCBG_02077	657	eukaryota>fungi>ascomycota	Histoplasma capsulatum G186AR	ankyrin repeat and zinc finger domain-containing protein [Histoplasma capsulatum G186AR].
KKZ68290.1	EMCG_06046	657	eukaryota>fungi>ascomycota	Emmonsia crescens UAMH 3008	hypothetical protein EMCG_06046 [Emmonsia crescens UAMH 3008].
XP_011275784.1	BN7_1903	607	eukaryota>fungi>ascomycota	Wickerhamomyces ciferrii	Ankyrin repeat-containing protein [Wickerhamomyces ciferrii].
OBA18998.1	METBIDRAFT_33625	607	eukaryota>fungi>ascomycota	Metschnikowia bicuspidata var. bicuspidata NRRL YB-4993	hypothetical protein METBIDRAFT_33625 [Metschnikowia bicuspidata var. bicuspidata NRRL YB-4993].
KOS21958.1	ESCO_001652	657	eukaryota>fungi>ascomycota	Escovopsis weberi	VMS1 -like protein [Escovopsis weberi].
ERT00183.1	HMPREF1624_03552	747	eukaryota>fungi>ascomycota	Sporothrix schenckii ATCC 58251	hypothetical protein HMPREF1624_03552 [Sporothrix schenckii ATCC 58251].

OAL30646.1	AYO20_08608	656	eukaryota>fungi>ascomycota	Fonsecaea nubica	hypothetical protein AYO20_08608 [Fonsecaea nubica].
OAG43026.1	AYO21_02645	656	eukaryota>fungi>ascomycota	Fonsecaea monophora	hypothetical protein AYO21_02645 [Fonsecaea monophora].
XP_003720619.1	MGG_03061	656	eukaryota>fungi>ascomycota	Magnaporthe oryzae 70-15	ankyrin repeat-containing protein [Magnaporthe oryzae 70-15].
XP_013279928.1	Z517_10865	656	eukaryota>fungi>ascomycota	Fonsecaea pedrosoi CBS 271.37	hypothetical protein Z517_10865 [Fonsecaea pedrosoi CBS 271.37].
KIH91403.1	SPBR_01398	760	eukaryota>fungi>ascomycota	Sporothrix brasiliensis 5110	c2h2 finger and ankyrin domain protein [Sporothrix brasiliensis 5110].
XP_001485720.1	PGUG_01391	608	eukaryota>fungi>ascomycota	Meyerozyma guilliermondii ATCC 6260	hypothetical protein PGUG_01391 [Meyerozyma guilliermondii ATCC 6260].
EDK37293.2	PGUG_01391	608	eukaryota>fungi>ascomycota	Meyerozyma guilliermondii ATCC 6260	hypothetical protein PGUG_01391 [Meyerozyma guilliermondii ATCC 6260].
XP_002489762.1	PAS_chr1-1_0119	609	eukaryota>fungi>ascomycota	Komagataella phaffii GS115	Zinc finger protein [Komagataella phaffii GS115].
XP_007743715.1	A1O5_04921	655	eukaryota>fungi>ascomycota	Cladophialophora psammophila CBS 110553	hypothetical protein A1O5_04921 [Cladophialophora psammophila CBS 110553].
XP_002845048.1	MCYG_07012	655	eukaryota>fungi>ascomycota	Arthroderma otae CBS 113480	ankyrin repeat and zinc finger domain-containing protein 1 [Arthroderma otae CBS 113480].
KKY22664.1	UCRPC4_g03166	655	eukaryota>fungi>ascomycota	Phaeomoniella chlamydospora	putative c2h2 finger and ankyrin domain [Phaeomoniella chlamydospora].
XP_013025427.1	SPOG_03558	609	eukaryota>fungi>ascomycota	Schizosaccharomyces cryophilus OY26	hypothetical protein SPOG_03558 [Schizosaccharomyces cryophilus OY26].
XP_013019045.1	SOCG_03679	610	eukaryota>fungi>ascomycota	Schizosaccharomyces octosporus yFS286	hypothetical protein SOCG_03679 [Schizosaccharomyces octosporus yFS286].
GAD97403.1	PVAR5_6078	655	eukaryota>fungi>ascomycota	Byssochlamys spectabilis No. 5	conserved hypothetical protein [Byssochlamys spectabilis No. 5].
CCJ28242.1	PNEJI1_003814	206	eukaryota>fungi>ascomycota	Pneumocystis jirovecii	unnamed protein product, partial [Pneumocystis jirovecii].
GAT29697.1	RIB2604_03100300	654	eukaryota>fungi>ascomycota	Aspergillus luchuensis	C2H2 finger and ankyrin domain protein [Aspergillus luchuensis].
KZM23566.1	ST47_g5292	654	eukaryota>fungi>ascomycota	Ascochyta rabiei	metal ion binding [Ascochyta rabiei].
XP_002614848.1	CLUG_04863	611	eukaryota>fungi>ascomycota	Clavispora lusitaniae ATCC 42720	hypothetical protein CLUG_04863 [Clavispora lusitaniae ATCC 42720].
KLJ08965.1	EMPG_15611	612	eukaryota>fungi>ascomycota	Emmonsia parva UAMH 139	hypothetical protein EMPG_15611 [Emmonsia parva UAMH 139].
AGO13664.1	AACERI_AaceriAGL211C	612	eukaryota>fungi>ascomycota	Saccharomycetaceae sp. 'Ashbya aceri'	AaceriAGL211Cp [Saccharomycetaceae sp. 'Ashbya aceri'].
EMD93029.1	COCHEDRAFT_1193365	654	eukaryota>fungi>ascomycota	Bipolaris maydis C5	hypothetical protein COCHEDRAFT_1193365 [Bipolaris maydis C5].
OAG04570.1	CC84DRAFT_1147262	654	eukaryota>fungi>ascomycota	Paraphaeosphaeria sporulosa	hypothetical protein CC84DRAFT_1147262 [Paraphaeosphaeria sporulosa].
NP_986456.1	AGOS_AGL211C	612	eukaryota>fungi>ascomycota	Eremothecium gossypii ATCC 10895	AGL211Cp [Eremothecium gossypii ATCC 10895].
KNG46192.1	TW65_97086	654	eukaryota>fungi>ascomycota	Stemphylium lycopersici	ankyrin repeat and zinc finger domain-containing protein [Stemphylium lycopersici].
AMD22389.1	AW171_hschr74424	613	eukaryota>fungi>ascomycota	Eremothecium sinecaudum	HGR050Wp [Eremothecium sinecaudum].
XP_003645204.1	Ecym_2677	615	eukaryota>fungi>ascomycota	Eremothecium cymbalariae DBVPG#7215	hypothetical protein Ecym_2677 [Eremothecium cymbalariae DBVPG#7215].
CRK23917.1	BN1708_013853	495	eukaryota>fungi>ascomycota	Verticillium longisporum	hypothetical protein BN1708_013853 [Verticillium longisporum].
CUS21473.1	LAQU0_S03e03400g	621	eukaryota>fungi>ascomycota	Lachancea quebecensis	LAQU0S03e03400g1_1 [Lachancea quebecensis].
XP_002552443.1	KLTH0C05016g	621	eukaryota>fungi>ascomycota	Lachancea thermotolerans CBS 6340	KLTH0C05016p [Lachancea thermotolerans CBS 6340].
XP_455615.1	KLLA0F11792g	621	eukaryota>fungi>ascomycota	Kluyveromyces lactis NRRL Y-1140	hypothetical protein [Kluyveromyces lactis NRRL Y-1140].
OAP61274.1	AYL99_03475	653	eukaryota>fungi>ascomycota	Fonsecaea erecta	hypothetical protein AYL99_03475 [Fonsecaea erecta].
KUI63964.1	VM1G_10743	653	eukaryota>fungi>ascomycota	Valsa mali	hypothetical protein VM1G_10743 [Valsa mali].
CDO95861.1	KLDO_g4086	623	eukaryota>fungi>ascomycota	Kluyveromyces dobzhanskii CBS 2104	unnamed protein product [Kluyveromyces dobzhanskii CBS 2104].
OAG26179.1	CC77DRAFT_28654	653	eukaryota>fungi>ascomycota	Alternaria alternata	ankyrin repeat and zinc finger domain-containing protein 1 [Alternaria alternata].
KUI59892.1	VP1G_07110	653	eukaryota>fungi>ascomycota	Valsa mali var. pyri	hypothetical protein VP1G_07110 [Valsa mali var. pyri].
XP_016630925.1	Z520_07522	653	eukaryota>fungi>ascomycota	Fonsecaea multimorphosa CBS 102226	hypothetical protein Z520_07522 [Fonsecaea multimorphosa CBS 102226].
XP_008595740.1	BBA_02421	652	eukaryota>fungi>ascomycota	Beauveria bassiana ARSEF 2860	ankyrin repeat and zinc finger domain-containing protein [Beauveria bassiana ARSEF 2860].
KYK59853.1	DCS_00987	652	eukaryota>fungi>ascomycota	Drechmeria coniospora	ankyrin repeat and zinc finger domain containing protein 1 [Drechmeria coniospora].
XP_008028992.1	SETTUDRAFT_119817	652	eukaryota>fungi>ascomycota	Setosphaeria turcica Et28A	hypothetical protein SETTUDRAFT_119817 [Setosphaeria turcica Et28A].
OAK97176.1	IQ06DRAFT_255342	652	eukaryota>fungi>ascomycota	Stagonospora sp. SRC1lsM3a	ankyrin repeat and zinc finger domain-containing protein 1 [Stagonospora sp. SRC1lsM3a].
XP_016247648.1	PV07_07169	652	eukaryota>fungi>ascomycota	Cladophialophora immunda	hypothetical protein PV07_07169 [Cladophialophora immunda].
XP_007915652.1	UCRPA7_4910	652	eukaryota>fungi>ascomycota	Phaeoacremonium minimum UCRPA7	putative ankyrin repeat-containing protein [Phaeoacremonium minimum UCRPA7].
XP_013331787.1	T310_0766	652	eukaryota>fungi>ascomycota	Rasamsonia emersonii CBS 393.64	C2H2 finger and ankyrin domain protein [Rasamsonia emersonii CBS 393.64].
CEP62824.1	LALA0_S06e04676g	623	eukaryota>fungi>ascomycota	Lachancea lanzarotensis	LALA0S06e04676g1_1 [Lachancea lanzarotensis].
XP_016624024.1	Z519_02747	652	eukaryota>fungi>ascomycota	Cladophialophora bantiana CBS 173.52	hypothetical protein Z519_02747 [Cladophialophora bantiana CBS 173.52].
CCK71526.1	KNAG_0H01130	625	eukaryota>fungi>ascomycota	Kazachstania naganishii CBS 8797	hypothetical protein KNAG_0H01130 [Kazachstania naganishii CBS 8797].
XP_001542812.1	HCAG_02983	651	eukaryota>fungi>ascomycota	Histoplasma capsulatum NAm1	conserved hypothetical protein [Histoplasma capsulatum NAm1].
KKY39242.1	UCDDA912_g00720	651	eukaryota>fungi>ascomycota	Diaporthe ampelina	putative ankyrin repeat-containing protein [Diaporthe ampelina].
DAA79236.1	A8136_0009	627	eukaryota>fungi>ascomycota	Arthroderma benhamiae CBS 112371	TPA_exp: Uncharacterized protein A8136_0009 [Arthroderma benhamiae CBS 112371].
KOH00903.1	DI49_0244	628	eukaryota>fungi>ascomycota	Saccharomyces eubayanus	VMS1-like protein [Saccharomyces eubayanus].
EYE93095.1	EURHEDRAFT_461082	651	eukaryota>fungi>ascomycota	Aspergillus ruber CBS 135680	putative C2H2 finger and ankyrin domain protein [Aspergillus ruber CBS 135680].
KPI41509.1	AB675_9149	650	eukaryota>fungi>ascomycota	Phialophora attae	hypothetical protein AB675_9149 [Phialophora attae].
KFZ00771.1	V501_10475	650	eukaryota>fungi>ascomycota	Pseudogymnoascus sp. VKM F-4519 (FW-2642)	hypothetical protein V501_10475 [Pseudogymnoascus sp. VKM F-4519 (FW-2642)].
XP_004181776.1	TBLA_0G03200	628	eukaryota>fungi>ascomycota	Tetrapisispora blattae CBS 6284	hypothetical protein TBLA_0G03200 [Tetrapisispora blattae CBS 6284].
KTB15941.1	AO443_002192	650	eukaryota>fungi>ascomycota	Candida glabrata	Protein VMS1 [Candida glabrata].
XP_447162.1	CAGL0H08349g	650	eukaryota>fungi>ascomycota	Candida glabrata CBS 138	hypothetical protein [Candida glabrata CBS 138].
XP_001526059.1	LELG_02617	628	eukaryota>fungi>ascomycota	Lodderomyces elongisporus NRRL YB-4239	conserved hypothetical protein [Lodderomyces elongisporus NRRL YB-4239].
ANB15145.1	AWJ20_2767	628	eukaryota>fungi>ascomycota	Sugiyamaella lignohabitans	Vms1p [Sugiyamaella lignohabitans].
EGU77083.1	FOXB_12381	650	eukaryota>fungi>ascomycota	Fusarium oxysporum Fo5176	hypothetical protein FOXB_12381 [Fusarium oxysporum Fo5176].
XP_003958680.1	KAFR_0H01350	629	eukaryota>fungi>ascomycota	Kazachstania africana CBS 2517	hypothetical protein KAFR_0H01350 [Kazachstania africana CBS 2517].
CDO54687.1	BN980_GECA08s03156g	631	eukaryota>fungi>ascomycota	Geotrichum candidum	similar to Saccharomyces cerevisiae YDR049W VMS1 Component of a Cdc48p-complex involved in protein quality control [Geotrichum candidum].
KTB04645.1	AO440_002253	650	eukaryota>fungi>ascomycota	Candida glabrata	Protein VMS1 [Candida glabrata].
KIW70894.1	PV04_03127	649	eukaryota>fungi>ascomycota	Capronia semi-immersa	hypothetical protein PV04_03127 [Capronia semi-immersa].
XP_009855236.1	NEUTE1DRAFT_89133	649	eukaryota>fungi>ascomycota	Neurospora tetrasperma FGSC 2508	hypothetical protein NEUTE1DRAFT_89133 [Neurospora tetrasperma FGSC 2508].
XP_003344072.1	SMAC_09055	649	eukaryota>fungi>ascomycota	Sordaria macrospora k-hell	hypothetical protein SMAC_09055 [Sordaria macrospora k-hell].
EJT42855.1	SKUD_136202	631	eukaryota>fungi>ascomycota	Saccharomyces kudriavzevii IFO 1802	VMS1-like protein [Saccharomyces kudriavzevii IFO 1802].
XP_964506.2	NCU02147	649	eukaryota>fungi>ascomycota	Neurospora crassa OR74A	ankyrin repeat and zinc finger domain-containing protein 1 [Neurospora crassa OR74A].
BAO38638.1	KLMA_20180	632	eukaryota>fungi>ascomycota	Kluyveromyces marxianus DMKU3-1042	zinc finger protein YDR049W [Kluyveromyces marxianus DMKU3-1042].
XP_016639036.1	SAPIO_CDS9908	649	eukaryota>fungi>ascomycota	Scedosporium apiospermum	hypothetical protein SAPIO_CDS9908 [Scedosporium apiospermum].
XP_013277036.1	Z518_00981	649	eukaryota>fungi>ascomycota	Rhinocladiella mackenziei CBS 650.93	hypothetical protein Z518_00981 [Rhinocladiella mackenziei CBS 650.93].
XP_007692257.1	COCMIDRAFT_106713	649	eukaryota>fungi>ascomycota	Bipolaris oryzae ATCC 44560	hypothetical protein COCMIDRAFT_106713 [Bipolaris oryzae ATCC 44560].
XP_007755176.1	A1O7_02960	649	eukaryota>fungi>ascomycota	Cladophialophora yegresii CBS 114405	hypothetical protein A1O7_02960 [Cladophialophora yegresii CBS 114405].
KKO98432.1	THAR02_09459	649	eukaryota>fungi>ascomycota	Trichoderma harzianum	hypothetical protein THAR02_09459 [Trichoderma harzianum].
XP_006964350.1	TRIREDRAFT_59270	649	eukaryota>fungi>ascomycota	Trichoderma reesei QM6a	predicted protein [Trichoderma reesei QM6a].
EYB28595.1	FG05_08845	632	eukaryota>fungi>ascomycota	Fusarium graminearum	hypothetical protein FG05_08845 [Fusarium graminearum].
AAU09687.1	-	632	eukaryota>fungi>ascomycota	Saccharomyces cerevisiae	YDR049W [Saccharomyces cerevisiae].
EIW11250.1	CENPK1137D_3868	632	eukaryota>fungi>ascomycota	Saccharomyces cerevisiae CEN.PK113-7D	Vms1p [Saccharomyces cerevisiae CEN.PK113-7D].
EGZ78412.1	NEUTE2DRAFT_154825	649	eukaryota>fungi>ascomycota	Neurospora tetrasperma FGSC 2509	hypothetical protein NEUTE2DRAFT_154825 [Neurospora tetrasperma FGSC 2509].
XP_008727986.1	G647_05433	649	eukaryota>fungi>ascomycota	Cladophialophora carrionii CBS 160.54	hypothetical protein G647_05433 [Cladophialophora carrionii CBS 160.54].
XP_014078492.1	COCC4DRAFT_196778	648	eukaryota>fungi>ascomycota	Bipolaris maydis ATCC 48331	hypothetical protein COCC4DRAFT_196778 [Bipolaris maydis ATCC 48331].
XP_001216951.1	ATEG_08330	648	eukaryota>fungi>ascomycota	Aspergillus terreus NIH2624	conserved hypothetical protein [Aspergillus terreus NIH2624].
XP_013312353.1	PV05_10455	648	eukaryota>fungi>ascomycota	Exophiala xenobiotica	hypothetical protein PV05_10455 [Exophiala xenobiotica].
CCJ29583.1	PNEJI1_003021	648	eukaryota>fungi>ascomycota	Pneumocystis jirovecii	unnamed protein product [Pneumocystis jirovecii].
AJU60663.1	H751_YJM248D00285	632	eukaryota>fungi>ascomycota	Saccharomyces cerevisiae YJM248	Vms1p [Saccharomyces cerevisiae YJM248].
AJU67689.1	H761_YJM470D00286	632	eukaryota>fungi>ascomycota	Saccharomyces cerevisiae YJM470	Vms1p [Saccharomyces cerevisiae YJM470].
CAY78557.1	EC1118_1D0_2938g	632	eukaryota>fungi>ascomycota	Saccharomyces cerevisiae EC1118	EC1118_1D0_2938p [Saccharomyces cerevisiae EC1118].
EEU05963.1	C1Q_03626	632	eukaryota>fungi>ascomycota	Saccharomyces cerevisiae JAY291	YDR049W-like protein [Saccharomyces cerevisiae JAY291].
XP_007700458.1	COCSADRAFT_37586	648	eukaryota>fungi>ascomycota	Bipolaris sorokiniana ND90Pr	hypothetical protein COCSADRAFT_37586 [Bipolaris sorokiniana ND90Pr].
GAA22286.1	SYK7_012001	632	eukaryota>fungi>ascomycota	Saccharomyces cerevisiae Kyokai no. 7	K7_Ydr049wp [Saccharomyces cerevisiae Kyokai no. 7].
KUE95473.1	TGAM01_10066	647	eukaryota>fungi>ascomycota	Trichoderma gamsii	hypothetical protein TGAM01_10066 [Trichoderma gamsii].
KZL72020.1	CT0861_01094	647	eukaryota>fungi>ascomycota	Colletotrichum tofieldiae	ankyrin repeat and zinc finger domain-containing protein [Colletotrichum tofieldiae].
KZZ86913.1	AAP_06103	490	eukaryota>fungi>ascomycota	Ascosphaera apis ARSEF 7405	Ankyrin repeat-containing domain protein [Ascosphaera apis ARSEF 7405].
XP_007715889.1	COCCADRAFT_105563	647	eukaryota>fungi>ascomycota	Bipolaris zeicola 26-R-13	hypothetical protein COCCADRAFT_105563 [Bipolaris zeicola 26-R-13].
XP_001242270.1	CIMG_06166	647	eukaryota>fungi>ascomycota	Coccidioides immitis RS	C2H2 finger and ankyrin domain-containing protein [Coccidioides immitis RS].
XP_003069529.1	CPC735_027200	647	eukaryota>fungi>ascomycota	Coccidioides posadasii C735 delta SOWgp	hypothetical protein CPC735_027200 [Coccidioides posadasii C735 delta SOWgp].
XP_014552072.1	COCVIDRAFT_19783	647	eukaryota>fungi>ascomycota	Bipolaris victoriae FI3	hypothetical protein COCVIDRAFT_19783 [Bipolaris victoriae FI3].
XP_016262356.1	PV06_05723	647	eukaryota>fungi>ascomycota	Exophiala oligosperma	hypothetical protein PV06_05723 [Exophiala oligosperma].
XP_006665999.1	CCM_00777	647	eukaryota>fungi>ascomycota	Cordyceps militaris CM01	ankyrin repeat and zinc finger domain containing protein 1 [Cordyceps militaris CM01].
EFW23021.1	CPSG_00920	647	eukaryota>fungi>ascomycota	Coccidioides posadasii str. Silveira	hypothetical protein CPSG_00920 [Coccidioides posadasii str. Silveira].
OAR00501.1	LLEC1_03033	647	eukaryota>fungi>ascomycota	Cordyceps confragosa	hypothetical protein LLEC1_03033 [Cordyceps confragosa].
XP_008717127.1	HMPREF1541_04560	646	eukaryota>fungi>ascomycota	Cyphellophora europaea CBS 101466	hypothetical protein HMPREF1541_04560 [Cyphellophora europaea CBS 101466].
EPS34614.1	PDE_09578	646	eukaryota>fungi>ascomycota	Penicillium oxalicum 114-2	hypothetical protein PDE_09578 [Penicillium oxalicum 114-2].
XP_013956801.1	TRIVIDRAFT_83943	646	eukaryota>fungi>ascomycota	Trichoderma virens Gv29-8	hypothetical protein TRIVIDRAFT_83943 [Trichoderma virens Gv29-8].
AJU74037.1	H770_YJM693D00282	632	eukaryota>fungi>ascomycota	Saccharomyces cerevisiae YJM693	Vms1p [Saccharomyces cerevisiae YJM693].
CEJ91301.1	VHEMI07023	646	eukaryota>fungi>ascomycota	Torrubiella hemipterigena	hypothetical protein VHEMI07023 [Torrubiella hemipterigena].
XP_007726443.1	A1O1_07383	646	eukaryota>fungi>ascomycota	Capronia coronata CBS 617.96	hypothetical protein A1O1_07383 [Capronia coronata CBS 617.96].
OAA45034.1	NOR_03788	646	eukaryota>fungi>ascomycota	Metarhizium rileyi RCEF 4871	ankyrin repeat and zinc finger domain containing protein 1 [Metarhizium rileyi RCEF 4871].
EWG97025.1	R103_D22001	632	eukaryota>fungi>ascomycota	Saccharomyces cerevisiae R103	hypothetical protein R103_D22001 [Saccharomyces cerevisiae R103].
OAA60007.1	ISF_06018	646	eukaryota>fungi>ascomycota	Isaria fumosorosea ARSEF 2679	ankyrin repeat and zinc finger domain containing protein 1 [Isaria fumosorosea ARSEF 2679].
EDV08248.1	SCRG_00465	632	eukaryota>fungi>ascomycota	Saccharomyces cerevisiae RM11-1a	conserved hypothetical protein [Saccharomyces cerevisiae RM11-1a].
CEJ60301.1	PMG11_08879	646	eukaryota>fungi>ascomycota	Penicillium brasilianum	Putative Ankyrin repeat protein [Penicillium brasilianum].
XP_007732913.1	A1O3_04595	645	eukaryota>fungi>ascomycota	Capronia epimyces CBS 606.96	hypothetical protein A1O3_04595 [Capronia epimyces CBS 606.96].
XP_002565491.1	Pc22g15750	645	eukaryota>fungi>ascomycota	Penicillium rubens Wisconsin 54-1255	Pc22g15750 [Penicillium rubens Wisconsin 54-1255].
CRL28694.1	PCAMFM013_S029g000110	645	eukaryota>fungi>ascomycota	Penicillium camemberti	Zinc finger, C2H2-type [Penicillium camemberti].
NP_010334.1	YDR049W	632	eukaryota>fungi>ascomycota	Saccharomyces cerevisiae S288c	Vms1p [Saccharomyces cerevisiae S288c].
XP_014534231.1	PDIP_52370	645	eukaryota>fungi>ascomycota	Penicillium digitatum Pd1	hypothetical protein PDIP_52370 [Penicillium digitatum Pd1].
EGA79534.1	VIN13_0784	632	eukaryota>fungi>ascomycota	Saccharomyces cerevisiae Vin13	YDR049W-like protein [Saccharomyces cerevisiae Vin13].
XP_002544695.1	UREG_04212	645	eukaryota>fungi>ascomycota	Uncinocarpus reesii 1704	conserved hypothetical protein [Uncinocarpus reesii 1704].
AJU82415.1	H781_YJM1133D00286	632	eukaryota>fungi>ascomycota	Saccharomyces cerevisiae YJM1133	Vms1p [Saccharomyces cerevisiae YJM1133].
AJU84420.1	H784_YJM1202D00287	632	eukaryota>fungi>ascomycota	Saccharomyces cerevisiae YJM1202	Vms1p [Saccharomyces cerevisiae YJM1202].
AJU85131.1	H785_YJM1208D00287	632	eukaryota>fungi>ascomycota	Saccharomyces cerevisiae YJM1208	Vms1p [Saccharomyces cerevisiae YJM1208].
XP_016600573.1	PEX2_073320	645	eukaryota>fungi>ascomycota	Penicillium expansum	hypothetical protein PEX2_073320 [Penicillium expansum].
KUM61627.1	ACN42_g5490	645	eukaryota>fungi>ascomycota	Penicillium freii	hypothetical protein ACN42_g5490 [Penicillium freii].
KXG49974.1	PGRI_059420	645	eukaryota>fungi>ascomycota	Penicillium griseofulvum	hypothetical protein PGRI_059420 [Penicillium griseofulvum].
KGO73507.1	PITC_044260	645	eukaryota>fungi>ascomycota	Penicillium italicum	hypothetical protein PITC_044260 [Penicillium italicum].
OAA75170.1	LEL_07158	645	eukaryota>fungi>ascomycota	Cordyceps confragosa RCEF 1005	ankyrin repeat and zinc finger domain containing protein 1 [Cordyceps confragosa RCEF 1005].
OBR08881.1	CH63R_07646	645	eukaryota>fungi>ascomycota	Colletotrichum higginsianum IMI 349063	ankyrin repeat and zinc finger domain-containing protein [Colletotrichum higginsianum IMI 349063].
OAQ86359.1	VFPBJ_00399	645	eukaryota>fungi>ascomycota	Purpureocillium lilacinum	C2H2 finger and ankyrin domain-containing protein [Purpureocillium lilacinum].
KOS44448.1	ACN38_g4630	644	eukaryota>fungi>ascomycota	Penicillium nordicum	hypothetical protein ACN38_g4630 [Penicillium nordicum].
EDN60395.1	SCY_0953	632	eukaryota>fungi>ascomycota	Saccharomyces cerevisiae YJM789	conserved protein [Saccharomyces cerevisiae YJM789].
XP_008090849.1	GLRG_02649	644	eukaryota>fungi>ascomycota	Colletotrichum graminicola M1.001	ankyrin repeat and zinc finger domain-containing protein [Colletotrichum graminicola M1.001].
KIV77050.1	PV11_08886	644	eukaryota>fungi>ascomycota	Exophiala sideris	hypothetical protein PV11_08886 [Exophiala sideris].
KID89374.1	MGU_03421	644	eukaryota>fungi>ascomycota	Metarhizium guizhouense ARSEF 977	ankyrin repeat and zinc finger domain containing protein 1 [Metarhizium guizhouense ARSEF 977].
CDM36006.1	PROQFM164_S04g000887	644	eukaryota>fungi>ascomycota	Penicillium roqueforti FM164	Ankyrin repeat-containing domain [Penicillium roqueforti FM164].
XP_011319867.1	FGSG_08845	632	eukaryota>fungi>ascomycota	Fusarium graminearum PH-1	hypothetical protein FGSG_08845 [Fusarium graminearum PH-1].
XP_003007611.1	VDBG_01799	473	eukaryota>fungi>ascomycota	Verticillium alfalfae VaMs.102	conserved hypothetical protein [Verticillium alfalfae VaMs.102].
KDN72332.1	CSUB01_00015	644	eukaryota>fungi>ascomycota	Colletotrichum sublineola	putative ankyrin repeat and zinc finger domain-containing protein [Colletotrichum sublineola].
XP_007599588.1	CFIO01_05112	644	eukaryota>fungi>ascomycota	Colletotrichum fioriniae PJ7	ankyrin repeat and zinc finger domain-containing protein [Colletotrichum fioriniae PJ7].
EQL03537.1	OCS_00729	644	eukaryota>fungi>ascomycota	Ophiocordyceps sinensis CO18	ankyrin repeat and zinc finger domain containing protein 1 [Ophiocordyceps sinensis CO18].
KMU84835.1	CIHG_02618	487	eukaryota>fungi>ascomycota	Coccidioides immitis H538.4	hypothetical protein CIHG_02618 [Coccidioides immitis H538.4].
XP_001274253.1	ACLA_012550	644	eukaryota>fungi>ascomycota	Aspergillus clavatus NRRL 1	C2H2 finger and ankyrin domain protein, putative [Aspergillus clavatus NRRL 1].
KND92587.1	TOPH_02817	644	eukaryota>fungi>ascomycota	Tolypocladium ophioglossoides CBS 100239	VMS1-like protein [Tolypocladium ophioglossoides CBS 100239].
KUL84732.1	ZTR_07591	643	eukaryota>fungi>ascomycota	Talaromyces verruculosus	hypothetical protein ZTR_07591 [Talaromyces verruculosus].
XP_001259889.1	NFIA_079330	643	eukaryota>fungi>ascomycota	Aspergillus fischeri NRRL 181	C2H2 finger and ankyrin domain protein, putative [Aspergillus fischeri NRRL 181].
XP_002147910.1	PMAA_084050	643	eukaryota>fungi>ascomycota	Talaromyces marneffei ATCC 18224	C2H2 finger and ankyrin domain protein, putative [Talaromyces marneffei ATCC 18224].
KEY78847.1	BA78_5462	643	eukaryota>fungi>ascomycota	Aspergillus fumigatus var. RP-2014	transcription factor C2H2 [Aspergillus fumigatus var. RP-2014].
AJV04076.1	H812_YJM1401D00286	632	eukaryota>fungi>ascomycota	Saccharomyces cerevisiae YJM1401	Vms1p [Saccharomyces cerevisiae YJM1401].
GAQ09304.1	ALT_6625	643	eukaryota>fungi>ascomycota	Aspergillus lentulus	VMS1 homolog C1827.04 [Aspergillus lentulus].
AJV02701.1	H810_YJM1399D00286	632	eukaryota>fungi>ascomycota	Saccharomyces cerevisiae YJM1399	Vms1p [Saccharomyces cerevisiae YJM1399].
GAM36070.1	TCE0_018r04873	643	eukaryota>fungi>ascomycota	Talaromyces cellulolyticus	hypothetical protein TCE0_018r04873 [Talaromyces cellulolyticus].
KZL86769.1	CI238_03285	643	eukaryota>fungi>ascomycota	Colletotrichum incanum	ankyrin repeat and zinc finger domain-containing protein [Colletotrichum incanum].
KID69932.1	MAN_02446	643	eukaryota>fungi>ascomycota	Metarhizium anisopliae ARSEF 549	ankyrin repeat and zinc finger domain containing protein 1, partial [Metarhizium anisopliae ARSEF 549].
AJV01995.1	H809_YJM1389D00286	632	eukaryota>fungi>ascomycota	Saccharomyces cerevisiae YJM1389	Vms1p [Saccharomyces cerevisiae YJM1389].
XP_007819963.1	MAA_03774	643	eukaryota>fungi>ascomycota	Metarhizium robertsii ARSEF 23	ankyrin repeat and zinc finger domain containing protein 1 [Metarhizium robertsii ARSEF 23].
XP_014541656.1	MBR_08249	643	eukaryota>fungi>ascomycota	Metarhizium brunneum ARSEF 3297	ankyrin repeat and zinc finger domain containing protein 1, partial [Metarhizium brunneum ARSEF 3297].
ENH71019.1	FOC1_g10008390	643	eukaryota>fungi>ascomycota	Fusarium oxysporum f. sp. cubense race 1	hypothetical protein FOC1_g10008390 [Fusarium oxysporum f. sp. cubense race 1].
XP_003298069.1	PTT_08665	643	eukaryota>fungi>ascomycota	Pyrenophora teres f. teres 0-1	hypothetical protein PTT_08665 [Pyrenophora teres f. teres 0-1].
AJV01282.1	H808_YJM1388D00286	632	eukaryota>fungi>ascomycota	Saccharomyces cerevisiae YJM1388	Vms1p [Saccharomyces cerevisiae YJM1388].
KEY68147.1	S7711_08335	642	eukaryota>fungi>ascomycota	Stachybotrys chartarum IBT 7711	hypothetical protein S7711_08335 [Stachybotrys chartarum IBT 7711].
KFA69784.1	S40285_02446	642	eukaryota>fungi>ascomycota	Stachybotrys chlorohalonata IBT 40285	hypothetical protein S40285_02446 [Stachybotrys chlorohalonata IBT 40285].
XP_013941152.1	TRIATDRAFT_285583	642	eukaryota>fungi>ascomycota	Trichoderma atroviride IMI 206040	hypothetical protein TRIATDRAFT_285583 [Trichoderma atroviride IMI 206040].
EWG39703.1	FVEG_02433	642	eukaryota>fungi>ascomycota	Fusarium verticillioides 7600	hypothetical protein FVEG_02433 [Fusarium verticillioides 7600].
GAO82430.1	AUD_1390	642	eukaryota>fungi>ascomycota	Aspergillus udagawae	VMS1 homolog C1827.04 [Aspergillus udagawae].
CCD33991.1	BofuT4_P043870.1	486	eukaryota>fungi>ascomycota	Botrytis cinerea T4	similar to transcription factor Zn, C2H2, partial sequence [Botrytis cinerea T4].
XP_001390289.2	ANI_1_482034	642	eukaryota>fungi>ascomycota	Aspergillus niger CBS 513.88	C2H2 finger and ankyrin domain protein [Aspergillus niger CBS 513.88].
AJU98461.1	H804_YJM1383D00283	632	eukaryota>fungi>ascomycota	Saccharomyces cerevisiae YJM1383	Vms1p [Saccharomyces cerevisiae YJM1383].
EHA21200.1	ASPNIDRAFT_50680	642	eukaryota>fungi>ascomycota	Aspergillus niger ATCC 1015	hypothetical protein ASPNIDRAFT_50680 [Aspergillus niger ATCC 1015].
ENH76017.1	Cob_02598	642	eukaryota>fungi>ascomycota	Colletotrichum orbiculare MAFF 240422	ankyrin repeat and zinc finger domain containing protein 1 [Colletotrichum orbiculare MAFF 240422].
KFA49718.1	S40293_01438	642	eukaryota>fungi>ascomycota	Stachybotrys chartarum IBT 40293	hypothetical protein S40293_01438 [Stachybotrys chartarum IBT 40293].
XP_753840.1	AFUA_5G07960	642	eukaryota>fungi>ascomycota	Aspergillus fumigatus Af293	C2H2 finger and ankyrin domain protein [Aspergillus fumigatus Af293].
XP_002482140.1	TSTA_119120	642	eukaryota>fungi>ascomycota	Talaromyces stipitatus ATCC 10500	C2H2 finger and ankyrin domain protein, putative [Talaromyces stipitatus ATCC 10500].
CEL04777.1	ASPCAL05902	642	eukaryota>fungi>ascomycota	Aspergillus calidoustus	Putative Ankyrin repeat protein [Aspergillus calidoustus].
XP_001939622.1	PTRG_09290	642	eukaryota>fungi>ascomycota	Pyrenophora tritici-repentis Pt-1C-BFP	ankyrin repeat and zinc finger domain containing protein 1 [Pyrenophora tritici-repentis Pt-1C-BFP].
XP_001644521.1	Kpol_1052p10	641	eukaryota>fungi>ascomycota	Vanderwaltozyma polyspora DSM 70294	hypothetical protein Kpol_1052p10 [Vanderwaltozyma polyspora DSM 70294].
KLP13521.1	LW94_10844	641	eukaryota>fungi>ascomycota	Fusarium fujikuroi	Found in Mitochondrial Proteome [Fusarium fujikuroi].
XP_016240904.1	PV08_01265	641	eukaryota>fungi>ascomycota	Exophiala spinifera	hypothetical protein PV08_01265 [Exophiala spinifera].
XP_007278021.1	CGGC5_7060	641	eukaryota>fungi>ascomycota	Colletotrichum gloeosporioides Nara gc5	ankyrin repeat and zinc finger domain containing protein 1 [Colletotrichum gloeosporioides Nara gc5].
OAQ68657.1	VFPPC_04868	641	eukaryota>fungi>ascomycota	Pochonia chlamydosporia 170	C2H2 finger and ankyrin domain-containing protein [Pochonia chlamydosporia 170].
KLO84474.1	LW93_1273	641	eukaryota>fungi>ascomycota	Fusarium fujikuroi	Found in Mitochondrial Proteome [Fusarium fujikuroi].
EQB52813.1	CGLO_07531	641	eukaryota>fungi>ascomycota	Colletotrichum gloeosporioides Cg-14	hypothetical protein CGLO_07531 [Colletotrichum gloeosporioides Cg-14].
XP_007807328.1	MAC_00988	641	eukaryota>fungi>ascomycota	Metarhizium acridum CQMa 102	ankyrin repeat and zinc finger domain containing protein 1 [Metarhizium acridum CQMa 102].
KKK18273.1	AOCH_001564	641	eukaryota>fungi>ascomycota	Aspergillus ochraceoroseus	hypothetical protein AOCH_001564 [Aspergillus ochraceoroseus].
AJU88556.1	H790_YJM1252D00282	632	eukaryota>fungi>ascomycota	Saccharomyces cerevisiae YJM1252	Vms1p [Saccharomyces cerevisiae YJM1252].
CCT69822.1	FFUJ_05741	641	eukaryota>fungi>ascomycota	Fusarium fujikuroi IMI 58289	related to Found in Mitochondrial Proteome [Fusarium fujikuroi IMI 58289].
XP_003683903.1	TPHA_0A03930	641	eukaryota>fungi>ascomycota	Tetrapisispora phaffii CBS 4417	hypothetical protein TPHA_0A03930 [Tetrapisispora phaffii CBS 4417].
GAT29698.1	RIB2604_03100310	641	eukaryota>fungi>ascomycota	Aspergillus luchuensis	C2H2 finger and ankyrin domain protein [Aspergillus luchuensis].
KFG83491.1	MANI_017959	640	eukaryota>fungi>ascomycota	Metarhizium anisopliae	ankyrin repeat and zinc finger domain containing protein 1 [Metarhizium anisopliae].
XP_013262922.1	A1O9_01892	640	eukaryota>fungi>ascomycota	Exophiala aquamarina CBS 119918	hypothetical protein A1O9_01892 [Exophiala aquamarina CBS 119918].
KPM39107.1	AK830_g7466	640	eukaryota>fungi>ascomycota	Neonectria ditissima	hypothetical protein AK830_g7466 [Neonectria ditissima].
XP_015410001.1	ANOM_003426	640	eukaryota>fungi>ascomycota	Aspergillus nomius NRRL 13137	C2H2 finger and ankyrin domain protein [Aspergillus nomius NRRL 13137].
EIT76179.1	Ao3042_07708	640	eukaryota>fungi>ascomycota	Aspergillus oryzae 3.042	ankyrin repeat protein [Aspergillus oryzae 3.042].

[illegible]

WP_014819922.1	<-codon_recognition+baeRF1+PELOTA*<-HPF_like	-	388	bacteria>proteobacteria>gammaproteobacteria	Pseudomonas stutzeri group	MULTISPECIES: hypothetical protein [Pseudomonas stutzeri group].
WP_015277577.1	HPF_like->codon_recognition+baeRF1+PELOTA*->	-	388	bacteria>proteobacteria>gammaproteobacteria	Pseudomonas stutzeri	hypothetical protein [Pseudomonas stutzeri].
WP_017245786.1	<-codon_recognition+baeRF1+PELOTA*<-HPF_like	-	388	bacteria>proteobacteria>gammaproteobacteria	Pseudomonas stutzeri	hypothetical protein [Pseudomonas stutzeri].
WP_019342403.1	<-codon_recognition+baeRF1+PELOTA*<-HPF_like	-	388	bacteria>proteobacteria>gammaproteobacteria	Pseudomonas stutzeri	hypothetical protein [Pseudomonas stutzeri].
WP_021206568.1	HPF_like->codon_recognition+baeRF1+PELOTA*->	-	388	bacteria>proteobacteria>gammaproteobacteria	Pseudomonas stutzeri	hypothetical protein [Pseudomonas stutzeri].
WP_025240940.1	<-codon_recognition+baeRF1+PELOTA*<-HPF_like	-	388	bacteria>proteobacteria>gammaproteobacteria	Pseudomonas stutzeri	hypothetical protein [Pseudomonas stutzeri].
WP_037042041.1	<-codon_recognition+baeRF1+PELOTA*<-HPF_like	-	388	bacteria>proteobacteria>gammaproteobacteria	Pseudomonas sp. BAY1663	hypothetical protein [Pseudomonas sp. BAY1663].
WP_038661604.1	HPF_like->codon_recognition+baeRF1+PELOTA*->	-	388	bacteria>proteobacteria>gammaproteobacteria	Pseudomonas stutzeri	hypothetical protein [Pseudomonas stutzeri].
WP_042925644.1	HPF_like->codon_recognition+baeRF1+PELOTA*->	-	388	bacteria>proteobacteria>gammaproteobacteria	Pseudomonas chloritidismutans	hypothetical protein [Pseudomonas chloritidismutans].
WP_044314624.1	<-codon_recognition+baeRF1+PELOTA*<-HPF_like	-	388	bacteria>proteobacteria>gammaproteobacteria	Pseudomonas stutzeri	hypothetical protein [Pseudomonas stutzeri].
WP_045158460.1	HPF_like->codon_recognition+baeRF1+PELOTA*->	-	388	bacteria>proteobacteria>gammaproteobacteria	Pseudomonas stutzeri	hypothetical protein [Pseudomonas stutzeri].
WP_045161303.1	<-codon_recognition+baeRF1+PELOTA*<-HPF_like	-	388	bacteria>proteobacteria>gammaproteobacteria	Pseudomonas stutzeri	hypothetical protein [Pseudomonas stutzeri].
WP_045428981.1	<-codon_recognition+baeRF1+PELOTA*<-HPF_like	-	388	bacteria>proteobacteria>gammaproteobacteria	Pseudomonas	MULTISPECIES: hypothetical protein [Pseudomonas].
WP_046162022.1	HPF_like->codon_recognition+baeRF1+PELOTA*->	-	388	bacteria>proteobacteria>gammaproteobacteria	Pseudomonas sp. 10B238	hypothetical protein [Pseudomonas sp. 10B238].
WP_049338994.1	<-codon_recognition+baeRF1+PELOTA*<-HPF_like	-	388	bacteria>proteobacteria>gammaproteobacteria	Pseudomonas stutzeri	hypothetical protein [Pseudomonas stutzeri].
WP_058065700.1	HPF_like->codon_recognition+baeRF1+PELOTA*->	-	388	bacteria>proteobacteria>gammaproteobacteria	Pseudomonas sp. TTU2014-105ASC	hypothetical protein [Pseudomonas sp. TTU2014-105ASC].
WP_063543817.1	<-codon_recognition+baeRF1+PELOTA*<-HPF_like	-	388	bacteria>proteobacteria>gammaproteobacteria	Pseudomonas stutzeri	hypothetical protein [Pseudomonas stutzeri].
WP_063625703.1	HPF_like->codon_recognition+baeRF1+PELOTA*->	-	388	bacteria>proteobacteria>gammaproteobacteria	Pseudomonas marginalis	hypothetical protein [Pseudomonas marginalis].
WP_063921074.1	<-codon_recognition+baeRF1+PELOTA*<-HPF_like	-	388	bacteria>proteobacteria>gammaproteobacteria	Pseudomonas viridiflava	hypothetical protein [Pseudomonas viridiflava].
OCX95356.1	HPF_like->codon_recognition+baeRF1+PELOTA*->	BCV62_14365	388	bacteria>proteobacteria>gammaproteobacteria	Pseudomonas sp. K35	hypothetical protein BCV62_14365 [Pseudomonas sp. K35].
WP_028239525.1	HPF_like->codon_recognition+baeRF1+PELOTA*->	-	387	bacteria>proteobacteria>gammaproteobacteria	Pseudomonas azotifigens	hypothetical protein [Pseudomonas azotifigens].
WP_041104505.1	<-codon_recognition+baeRF1+PELOTA*<-HPF_like	-	387	bacteria>proteobacteria>gammaproteobacteria	Pseudomonas stutzeri group	MULTISPECIES: hypothetical protein [Pseudomonas stutzeri group].
WP_043221030.1	HPF_like->codon_recognition+baeRF1+PELOTA*->	-	387	bacteria>proteobacteria>gammaproteobacteria	Pseudomonas balearica	hypothetical protein [Pseudomonas balearica].
CJK44726.1	<-codon_recognition+baeRF1+PELOTA*<-HPF_like	ERS022045_00214	387	bacteria>firmicutes	Streptococcus pneumoniae	Uncharacterised protein [Streptococcus pneumoniae].
WP_061337157.1	<-codon_recognition+baeRF1+PELOTA*<-HPF_like	-	387	bacteria>proteobacteria>gammaproteobacteria	Pseudomonas balearica	hypothetical protein [Pseudomonas balearica].
WP_018167960.1	codon_recognition+baeRF1+PELOTA*->HPF_like->	-	384	bacteria>proteobacteria>gammaproteobacteria	Thioalkalivibrio	MULTISPECIES: hypothetical protein [Thioalkalivibrio].
WP_018941681.1	<-HPF_like<-codon_recognition+baeRF1+PELOTA*	-	384	bacteria>proteobacteria>gammaproteobacteria	Thioalkalivibrio sp. AKL11	hypothetical protein [Thioalkalivibrio sp. AKL11].
WP_018948852.1	<-HPF_like<-codon_recognition+baeRF1+PELOTA*	-	384	bacteria>proteobacteria>gammaproteobacteria	Thioalkalivibrio sp. ALMg11	hypothetical protein [Thioalkalivibrio sp. ALMg11].
WP_019594886.1	<-HPF_like<-codon_recognition+baeRF1+PELOTA*	-	384	bacteria>proteobacteria>gammaproteobacteria	Thioalkalivibrio sp. ALM2T	hypothetical protein [Thioalkalivibrio sp. ALM2T].
WP_019600464.1	<-HPF_like<-codon_recognition+baeRF1+PELOTA*	-	384	bacteria>bacteroidetes	Rhodonellum psychrophilum	hypothetical protein [Rhodonellum psychrophilum].
WP_019627362.1	<-HPF_like<-codon_recognition+baeRF1+PELOTA*	-	384	bacteria>proteobacteria>gammaproteobacteria	Thioalkalivibrio sp. AKL10	hypothetical protein [Thioalkalivibrio sp. AKL10].
WP_024326469.1	<-HPF_like<-codon_recognition+baeRF1+PELOTA*	-	384	bacteria>proteobacteria>gammaproteobacteria	Thioalkalivibrio sp. AKL19	hypothetical protein [Thioalkalivibrio sp. AKL19].
WP_044117711.1	<-a/b_hydrolase<-HPF_like<-codon_recognition+baeRF1+PELOTA*	-	384	bacteria>bacteroidetes	Alkaliflexus imshenetskii	hypothetical protein [Alkaliflexus imshenetskii].
WP_051497679.1	<-HPF_like<-codon_recognition+baeRF1+PELOTA*	-	384	bacteria>proteobacteria>gammaproteobacteria	Candidatus Contendobacter odensis	hypothetical protein [Candidatus Contendobacter odensis].
WP_051663980.1	<-HPF_like<-codon_recognition+baeRF1+PELOTA*	-	384	bacteria>bacteroidetes	Dyadobacter crusticola	hypothetical protein [Dyadobacter crusticola].
WP_016988111.1	<-HPF_like<-codon_recognition+baeRF1+PELOTA*	-	383	bacteria>bacteroidetes	Flavobacterium sp. ACAM 123	hypothetical protein [Flavobacterium sp. ACAM 123].
WP_026977713.1	codon_recognition+baeRF1+PELOTA*->HPF_like->	-	383	bacteria>bacteroidetes	Flavobacterium tegetincola	hypothetical protein [Flavobacterium tegetincola].
WP_046851667.1	<-HPF_like<-codon_recognition+baeRF1+PELOTA*	-	383	bacteria>proteobacteria>betaproteobacteria	Nitrosomonas communis	hypothetical protein [Nitrosomonas communis].
WP_058521739.1	codon_recognition+baeRF1+PELOTA*->HPF_like->	-	382	bacteria>proteobacteria>gammaproteobacteria	Legionella tucsonensis	hypothetical protein [Legionella tucsonensis].
WP_003235861.1	<-codon_recognition+baeRF1+PELOTA*<-HPF_like	-	375	bacteria>proteobacteria>gammaproteobacteria	Pseudomonas fluorescens	hypothetical protein [Pseudomonas fluorescens].
WP_054136995.1	<-HPF_like<-codon_recognition+baeRF1+PELOTA*	-	375	bacteria>proteobacteria>betaproteobacteria	unclassified Betaproteobacteria (miscellaneous)	MULTISPECIES: hypothetical protein [unclassified Betaproteobacteria (miscellaneous)].
WP_057427733.1	<-codon_recognition+baeRF1+PELOTA*<-HPF_like	-	375	bacteria>proteobacteria>gammaproteobacteria	Pseudomonas syringae	hypothetical protein [Pseudomonas syringae].
WP_057724992.1	<-codon_recognition+baeRF1+PELOTA*<-HPF_like	-	375	bacteria>proteobacteria>gammaproteobacteria	Pseudomonas orientalis	hypothetical protein [Pseudomonas orientalis].

#;other associations

WP_008476919.1	<-HPF_like<-codon_recognition+baeRF1+PELOTA*	-	410	bacteria>chloroflexi	Nitrolancea hollandica	hypothetical protein [Nitrolancea hollandica].
WP_011521408.1	HPF_like+HTH+HTH->codon_recognition+baeRF1+PELOTA*->	-	388	bacteria>acidobacteria	Candidatus Koribacter versatilis	hypothetical protein [Candidatus Koribacter versatilis].
WP_043833110.1	<-HPF_like<-codon_recognition+baeRF1+PELOTA*	-	366	bacteria>proteobacteria>alphaproteobacteria	Roseomonas aerilata	hypothetical protein [Roseomonas aerilata].
WP_012521203.1	<-HPF_like+S1COLD<-baeRF1*	-	149	bacteria>proteobacteria>alphaproteobacteria	Phenylobacterium zucineum	hypothetical protein [Phenylobacterium zucineum].
WP_008476919.1	<-HPF_like<-codon_recognition+baeRF1+PELOTA*	-	410	bacteria>chloroflexi	Nitrolancea hollandica	hypothetical protein [Nitrolancea hollandica].
WP_043833110.1	<-HPF_like<-codon_recognition+baeRF1+PELOTA*	-	366	bacteria>proteobacteria>alphaproteobacteria	Roseomonas aerilata	hypothetical protein [Roseomonas aerilata].
WP_011521408.1	HPF_like+HTH+HTH->codon_recognition+baeRF1+PELOTA*->	-	388	bacteria>acidobacteria	Candidatus Koribacter versatilis	hypothetical protein [Candidatus Koribacter versatilis].

III. Other lists of sequences

A. Newly-defined ArfT-like family of ribosome rescue factors

1056;

CAJ35087.1	118	LRC55	archaea>euryarchaeota	Methanocella arvoryzae MRE50	hypothetical protein LRC55 [Methanocella arvoryzae MRE50]	110619809
CAJ36479.1	117	gsiB	archaea>euryarchaeota	Methanocella arvoryzae MRE50	putative general stress protein B [Methanocella arvoryzae MRE50]	110621201
BAI60074.1	113	MCP_0002	archaea>euryarchaeota	Methanocella paludicola SANAE	conserved hypothetical protein [Methanocella paludicola SANAE]	282154986
AFD00218.1	101	Mtc_1466	archaea>euryarchaeota	Methanocella conradii HZ254	Small hydrophilic plant seed protein [Methanocella conradii HZ254]	379321265
BAI62067.1	101	MCP_1995	archaea>euryarchaeota	Methanocella paludicola SANAE	conserved hypothetical protein [Methanocella paludicola SANAE]	282156979
BAI61812.1	96	MCP_1740	archaea>euryarchaeota	Methanocella paludicola SANAE	conserved hypothetical protein [Methanocella paludicola SANAE]	282156724
BAI60673.1	93	MCP_0601	archaea>euryarchaeota	Methanocella paludicola SANAE	conserved hypothetical protein [Methanocella paludicola SANAE]	282155585
CAJ38038.1	87	RRC309	archaea>euryarchaeota	Methanocella arvoryzae MRE50	hypothetical protein RRC309 [Methanocella arvoryzae MRE50]	110622760
AFD00460.1	84	Mtc_1716	archaea>euryarchaeota	Methanocella conradii HZ254	hypothetical protein Mtc_1716 [Methanocella conradii HZ254]	379321507
BAI62068.1	84	MCP_1996	archaea>euryarchaeota	Methanocella paludicola SANAE	hypothetical protein MCP_1996 [Methanocella paludicola SANAE]	282156980
BAI61160.1	83	MCP_1088	archaea>euryarchaeota	Methanocella paludicola SANAE	conserved hypothetical protein [Methanocella paludicola SANAE]	282156072
CAJ35261.1	83	LRC281	archaea>euryarchaeota	Methanocella arvoryzae MRE50	hypothetical protein LRC281 [Methanocella arvoryzae MRE50]	110619983
AFD00932.1	80	Mtc_2197	archaea>euryarchaeota	Methanocella conradii HZ254	hypothetical protein Mtc_2197 [Methanocella conradii HZ254]	379321979
BAI62410.1	80	MCP_2338	archaea>euryarchaeota	Methanocella paludicola SANAE	conserved hypothetical protein [Methanocella paludicola SANAE]	282157322
AFD00872.1	79	Mtc_2134	archaea>euryarchaeota	Methanocella conradii HZ254	hypothetical protein Mtc_2134 [Methanocella conradii HZ254]	379321919
CAJ38039.1	78	RRC310	archaea>euryarchaeota	Methanocella arvoryzae MRE50	hypothetical protein RRC310 [Methanocella arvoryzae MRE50]	110622761
BAI60107.1	77	MCP_0035	archaea>euryarchaeota	Methanocella paludicola SANAE	conserved hypothetical protein [Methanocella paludicola SANAE]	282155019
BAI61165.1	66	MCP_1093	archaea>euryarchaeota	Methanocella paludicola SANAE	hypothetical protein MCP_1093 [Methanocella paludicola SANAE]	282156077
AEB67303.1	63	MCON_0452	archaea>euryarchaeota	Methanothrix soehngenii GP6	conserved hypothetical protein [Methanothrix soehngenii GP6]	328927501
BAI61164.1	61	MCP_1092	archaea>euryarchaeota	Methanocella paludicola SANAE	hypothetical protein MCP_1092 [Methanocella paludicola SANAE]	282156076
BAI62390.1	61	MCP_2318	archaea>euryarchaeota	Methanocella paludicola SANAE	hypothetical protein MCP_2318 [Methanocella paludicola SANAE]	282157302
BAI61181.1	59	MCP_1109	archaea>euryarchaeota	Methanocella paludicola SANAE	hypothetical protein MCP_1109 [Methanocella paludicola SANAE]	282156093
CAJ37734.1	58	RCIX2690	archaea>euryarchaeota	Methanocella arvoryzae MRE50	hypothetical protein RCIX2690 [Methanocella arvoryzae MRE50]	110622456
AFD00493.1	57	Mtc_1749	archaea>euryarchaeota	Methanocella conradii HZ254	hypothetical protein Mtc_1749 [Methanocella conradii HZ254]	379321540
BAI61589.1	57	MCP_1517	archaea>euryarchaeota	Methanocella paludicola SANAE	conserved hypothetical protein [Methanocella paludicola SANAE]	282156501
AFD00055.1	56	Mtc_1301	archaea>euryarchaeota	Methanocella conradii HZ254	putative small hydrophilic plant seed protein [Methanocella conradii HZ254]	379321102
BAI61572.1	55	MCP_1500	archaea>euryarchaeota	Methanocella paludicola SANAE	conserved hypothetical protein [Methanocella paludicola SANAE]	282156484
BAI61883.1	54	MCP_1811	archaea>euryarchaeota	Methanocella paludicola SANAE	conserved hypothetical protein [Methanocella paludicola SANAE]	282156795
AFU59187.1	158	gsiB	archaea>thaumarchaeota	Candidatus Nitrososphaera gargensis Ga9.2	putative general stress protein GsiB [Candidatus Nitrososphaera gargensis Ga9.2]	408365457
AFU57121.1	107	Ngar_c01720	archaea>thaumarchaeota	Candidatus Nitrososphaera gargensis Ga9.2	putative glucose starvation-inducible protein B [Candidatus Nitrososphaera gargensis Ga9.2]	408363391
OGY07671.1	112	A2694_01635	bacteria	Candidatus Blackburnbacteria bacterium RIFCSPHIGHO2_01_FULL_40_17	hypothetical protein A2694_01635 [Candidatus Blackburnbacteria bacterium RIFCSPHIGHO2_01_FULL_40_17]	1087922498
OGY07740.1	112	A3D24_02730	bacteria	Candidatus Blackburnbacteria bacterium RIFCSPHIGHO2_02_FULL_39_13	hypothetical protein A3D24_02730 [Candidatus Blackburnbacteria bacterium RIFCSPHIGHO2_02_FULL_39_13]	1087924782
OGY12799.1	112	A3A77_02895	bacteria	Candidatus Blackburnbacteria bacterium RIFCSPLOWO2_01_FULL_40_20	hypothetical protein A3A77_02895 [Candidatus Blackburnbacteria bacterium RIFCSPLOWO2_01_FULL_40_20]	1087930211
AKM82718.1	98	UT28_C0001G0941	bacteria	Berkelbacteria bacterium GW2011_GWE1_39_12	hypothetical protein UT28_C0001G0941 [Berkelbacteria bacterium GW2011_GWE1_39_12]	837352681
KKQ74671.1	98	US94_C0001G0072	bacteria	Berkelbacteria bacterium GW2011_GWB1_38_5	General stress protein [Berkelbacteria bacterium GW2011_GWB1_38_5]	818395579
KKQ90845.1	98	UT15_C0003G0020	bacteria	Berkelbacteria bacterium GW2011_GWA1_39_10	General stress protein [Berkelbacteria bacterium GW2011_GWA1_39_10]	818413086
KKW30830.1	96	UY72_C0002G0005	bacteria	Candidatus Uhrbacteria bacterium GW2011_GWD2_52_7	hypothetical protein UY72_C0002G0005 [Candidatus Uhrbacteria bacterium GW2011_GWD2_52_7]	818896403
OLC62758.1	95	AUH76_07720	bacteria	Candidatus Rokubacteria bacterium 13_1_40CM_4_67_11	stress-induced protein [Candidatus Rokubacteria bacterium 13_1_40CM_4_67_11]	1125321024
OIP22264.1	94	AUJ93_04665	bacteria	bacterium CG2_30_33_46	hypothetical protein AUJ93_04665 [bacterium CG2_30_33_46]	1101168671
KPJ62963.1	93	AMK68_04330	bacteria	candidate division KD3-62 bacterium DG_56	general stress protein B [candidate division KD3-62 bacterium DG_56]	931371833
KKQ67000.1	89	US86_C0002G0117	bacteria	Candidatus Daviesbacteria bacterium GW2011_GWA2_38_24	Glucose starvation-inducible protein B [Candidatus Daviesbacteria bacterium GW2011_GWA2_38_24]	818387578
KKQ80760.1	89	UT01_C0006G0021	bacteria	Candidatus Daviesbacteria bacterium GW2011_GWA1_38_7	Glucose starvation-inducible protein B [Candidatus Daviesbacteria bacterium GW2011_GWA1_38_7]	818402320
OGE22746.1	89	A2688_01380	bacteria	Candidatus Daviesbacteria bacterium RIFCSPHIGHO2_01_FULL_38_8	hypothetical protein A2688_01380 [Candidatus Daviesbacteria bacterium RIFCSPHIGHO2_01_FULL_38_8]	1083126736
OGH90699.1	88	A2479_00645	bacteria	Candidatus Magasanikbacteria bacterium RIFOXYC2_FULL_39_8	hypothetical protein A2479_00645 [Candidatus Magasanikbacteria bacterium RIFOXYC2_FULL_39_8]	1083704780
OGH11778.1	86	A2857_04785	bacteria	Candidatus Levybacteria bacterium RIFCSPHIGHO2_01_FULL_36_15	hypothetical protein A2857_04785 [Candidatus Levybacteria bacterium RIFCSPHIGHO2_01_FULL_36_15]	1083619658
OGH38589.1	86	A2905_04105	bacteria	Candidatus Levybacteria bacterium RIFCSPLOWO2_01_FULL_36_10	hypothetical protein A2905_04105 [Candidatus Levybacteria bacterium RIFCSPLOWO2_01_FULL_36_10]	1083648911
OGY32585.1	86	A3A57_01330	bacteria	Candidatus Woykebacteria bacterium RIFCSPLOWO2_01_FULL_41_12	general stress protein [Candidatus Woykebacteria bacterium RIFCSPLOWO2_01_FULL_41_12]	1087951209
OGY21637.1	85	A2113_03690	bacteria	Candidatus Woykebacteria bacterium GWA1_44_8	general stress protein [Candidatus Woykebacteria bacterium GWA1_44_8]	1087939499
OGY23584.1	85	A2126_03195	bacteria	Candidatus Woykebacteria bacterium GWB1_45_5	general stress protein [Candidatus Woykebacteria bacterium GWB1_45_5]	1087941512
OGY25077.1	85	A2134_03550	bacteria	Candidatus Woykebacteria bacterium RBG_16_39_9b	hypothetical protein A2134_03550 [Candidatus Woykebacteria bacterium RBG_16_39_9b]	1087943109
OGY26200.1	85	A2Z24_01370	bacteria	Candidatus Woykebacteria bacterium RBG_16_44_10	general stress protein [Candidatus Woykebacteria bacterium RBG_16_44_10]	1087944307
OGY30261.1	85	A3F35_01290	bacteria	Candidatus Woykebacteria bacterium RIFCSPHIGHO2_12_FULL_45_10	general stress protein [Candidatus Woykebacteria bacterium RIFCSPHIGHO2_12_FULL_45_10]	1087948718
OGY24885.1	84	A2Y57_02135	bacteria	Candidatus Woykebacteria bacterium RBG_13_40_7b	hypothetical protein A2Y57_02135 [Candidatus Woykebacteria bacterium RBG_13_40_7b]	1087942906
OGY24357.1	83	A2172_00345	bacteria	Candidatus Woykebacteria bacterium RBG_13_40_15	general stress protein [Candidatus Woykebacteria bacterium RBG_13_40_15]	1087942320
OGY25635.1	83	A2Z11_04215	bacteria	Candidatus Woykebacteria bacterium RBG_16_43_9	general stress protein [Candidatus Woykebacteria bacterium RBG_16_43_9]	1087943702
OGY26868.1	83	A2Z11_01700	bacteria	Candidatus Woykebacteria bacterium RBG_16_43_9	general stress protein [Candidatus Woykebacteria bacterium RBG_16_43_9]	1087945028
OGY28332.1	83	A2Z42_03125	bacteria	Candidatus Woykebacteria bacterium RBG_19FT_COMBO_43_10	general stress protein [Candidatus Woykebacteria bacterium RBG_19FT_COMBO_43_10]	1087946639
OIP22263.1	83	AUJ93_04660	bacteria	bacterium CG2_30_33_46	hypothetical protein AUJ93_04660 [bacterium CG2_30_33_46]	1101168670
AKM82410.1	82	UT28_C0001G0609	bacteria	Berkelbacteria bacterium GW2011_GWE1_39_12	hypothetical protein UT28_C0001G0609 [Berkelbacteria bacterium GW2011_GWE1_39_12]	837352373
OGE74323.1	80	A2717_02150	bacteria	Candidatus Doudnabacteria bacterium RIFCSPHIGHO2_01_FULL_41_86	hypothetical protein A2717_02150 [Candidatus Doudnabacteria bacterium RIFCSPHIGHO2_01_FULL_41_86]	1083354615
OGE75566.1	80	A3K07_01905	bacteria	Candidatus Doudnabacteria bacterium RIFCSPHIGHO2_01_43_10	hypothetical protein A3K07_01905 [Candidatus Doudnabacteria bacterium RIFCSPHIGHO2_01_43_10]	1083356022
OGE85362.1	80	A3E28_01720	bacteria	Candidatus Doudnabacteria bacterium RIFCSPHIGHO2_12_FULL_42_22	hypothetical protein A3E28_01720 [Candidatus Doudnabacteria bacterium RIFCSPHIGHO2_12_FULL_42_22]	1083367448
OGE86900.1	80	A3C49_02555	bacteria	Candidatus Doudnabacteria bacterium RIFCSPHIGHO2_02_FULL_42_25	hypothetical protein A3C49_02555 [Candidatus Doudnabacteria bacterium RIFCSPHIGHO2_02_FULL_42_25]	1083369163
OGE92499.1	80	A2895_02710	bacteria	Candidatus Doudnabacteria bacterium RIFCSPLOWO2_01_FULL_42_60	hypothetical protein A2895_02710 [Candidatus Doudnabacteria bacterium RIFCSPLOWO2_01_FULL_42_60]	1083377703
OGE99283.1	80	A3G89_01135	bacteria	Candidatus Doudnabacteria bacterium RIFCSPLOWO2_12_FULL_42_9	hypothetical protein A3G89_01135 [Candidatus Doudnabacteria bacterium RIFCSPLOWO2_12_FULL_42_9]	1083386260
OGF00223.1	80	A3I07_02620	bacteria	Candidatus Doudnabacteria bacterium RIFCSPLOWO2_02_FULL_42_9	hypothetical protein A3I07_02620 [Candidatus Doudnabacteria bacterium RIFCSPLOWO2_02_FULL_42_9]	1083387495
KKQ93958.1	78	UT18_C0013G0005	bacteria	candidate division CPR2 bacterium GW2011_GWC2_39_10	hypothetical protein UT18_C0013G0005 [candidate division CPR2 bacterium GW2011_GWC2_39_10]	818416381
KKR34690.1	78	UT66_C0018G0029	bacteria	candidate division CPR2 bacterium GW2011_GWC1_39_9	hypothetical protein UT66_C0018G0029 [candidate division CPR2 bacterium GW2011_GWC1_39_9]	818479767
OGH41406.1	78	A3H79_02025	bacteria	Candidatus Levybacteria bacterium RIFCSPLOWO2_02_FULL_36_8b	hypothetical protein A3H79_02025 [Candidatus Levybacteria bacterium RIFCSPLOWO2_02_FULL_36_8b]	1083651862
OGL21623.1	77	A2707_06345	bacteria	Candidatus Saccharibacteria bacterium RIFCSPHIGHO2_01_FULL_45_15	hypothetical protein A2707_06345 [Candidatus Saccharibacteria bacterium RIFCSPHIGHO2_01_FULL_45_15]	1084123946
OGL27699.1	77	A3C39_04925	bacteria	Candidatus Saccharibacteria bacterium RIFCSPHIGHO2_02_FULL_46_12	hypothetical protein A3C39_04925 [Candidatus Saccharibacteria bacterium RIFCSPHIGHO2_02_FULL_46_12]	1084130385
OGL32079.1	77	A3E76_02285	bacteria	Candidatus Saccharibacteria bacterium RIFCSPHIGHO2_12_FULL_44_22	hypothetical protein A3E76_02285 [Candidatus Saccharibacteria bacterium RIFCSPHIGHO2_12_FULL_44_22]	1084135069
OGE30976.1	75	A2631_04825	bacteria	Candidatus Daviesbacteria bacterium RIFCSPHIGHO2_01_FULL_44_29	hypothetical protein A2631_04825 [Candidatus Daviesbacteria bacterium RIFCSPHIGHO2_01_FULL_44_29]	1083135472
OGE40045.1	75	A3D25_04555	bacteria	Candidatus Daviesbacteria bacterium RIFCSPHIGHO2_02_FULL_43_12	hypothetical protein A3D25_04555 [Candidatus Daviesbacteria bacterium RIFCSPHIGHO2_02_FULL_43_12]	1083144951
OGE41473.1	75	A3E86_05255	bacteria	Candidatus Daviesbacteria bacterium RIFCSPHIGHO2_12_FULL_47_45	hypothetical protein A3E86_05255 [Candidatus Daviesbacteria bacterium RIFCSPHIGHO2_12_FULL_47_45]	1083146422
OGE70275.1	75	A3B55_01015	bacteria	Candidatus Daviesbacteria bacterium RIFCSPLOWO2_01_FULL_43_15	hypothetical protein A3B55_01015 [Candidatus Daviesbacteria bacterium RIFCSPLOWO2_01_FULL_43_15]	1083348871
KKQ46148.1	73	US63_C0006G0025	bacteria	Candidatus Moranbacteria bacterium GW2011_GWC2_37_8	hypothetical protein US63_C0006G0025 [Candidatus Moranbacteria bacterium GW2011_GWC2_37_8]	818365561
KKQ62451.1	73	US82_C0011G0025	bacteria	Parcubacteria group bacterium GW2011_GWC1_38_22	hypothetical protein US82_C0011G0025 [Parcubacteria group bacterium GW2011_GWC1_38_22]	818382880
KKQ81404.1	73	UT03_C0003G0013	bacteria	Candidatus Moranbacteria bacterium GW2011_GWD2_38_7	hypothetical protein UT03_C0003G0013 [Candidatus Moranbacteria bacterium GW2011_GWD2_38_7]	818403013
KKR18639.1	73	UT47_C0001G0029	bacteria	candidate division CPR2 bacterium GW2011_GWC2_39_35	hypothetical protein UT47_C0001G0029 [candidate division CPR2 bacterium GW2011_GWC2_39_35]	818462139
KKR29469.1	73	UT60_C0001G0005	bacteria	candidate division CPR2 bacterium GW2011_GWD2_39_7	hypothetical protein UT60_C0001G0005 [candidate division CPR2 bacterium GW2011_GWD2_39_7]	818473733
KKR29694.1	73	UT59_C0001G0003	bacteria	candidate division CPR2 bacterium GW2011_GWD1_39_7	hypothetical protein UT59_C0001G0003 [candidate division CPR2 bacterium GW2011_GWD1_39_7]	818473972
KKS09624.1	73	UU65_C0001G0029	bacteria	candidate division CPR2 bacterium GW2011_GWC1_41_48	hypothetical protein UU65_C0001G0029 [candidate division CPR2 bacterium GW2011_GWC1_41_48]	818558410
OGB59479.1	73	A2Y27_00805	bacteria	candidate division CPR2 bacterium GWD1_39_7	hypothetical protein A2Y27_00805 [candidate division CPR2 bacterium GWD1_39_7]	1082845836
OGB71697.1	73	A2Y26_03680	bacteria	candidate division CPR2 bacterium GWD2_39_7	hypothetical protein A2Y26_03680 [candidate division CPR2 bacterium GWD2_39_7]	1082858598
OGE88915.1	73	A3B77_03205	bacteria	Candidatus Doudnabacteria bacterium RIFCSPHIGHO2_02_FULL_49_24	hypothetical protein A3B77_03205 [Candidatus Doudnabacteria bacterium RIFCSPHIGHO2_02_FULL_49_24]	1083373575
OGE89125.1	73	A2760_04140	bacteria	Candidatus Doudnabacteria bacterium RIFCSPHIGHO2_01_FULL_50_67	hypothetical protein A2760_04140 [Candidatus Doudnabacteria bacterium RIFCSPHIGHO2_01_FULL_50_67]	1083373810
OGE90145.1	73	A3E29_03500	bacteria	Candidatus Doudnabacteria bacterium RIFCSPHIGHO2_12_FULL_48_16	hypothetical protein A3E29_03500 [Candidatus Doudnabacteria bacterium RIFCSPHIGHO2_12_FULL_48_16]	1083374936
OGE97232.1	73	A2990_01405	bacteria	Candidatus Doudnabacteria bacterium RIFCSPLOWO2_01_FULL_49_40	hypothetical protein A2990_01405 [Candidatus Doudnabacteria bacterium RIFCSPLOWO2_01_FULL_49_40]	1083383932
OGF03287.1	73	A3J07_04360	bacteria	Candidatus Doudnabacteria bacterium RIFCSPLOWO2_02_FULL_49_13	hypothetical protein A3J07_04360 [Candidatus Doudnabacteria bacterium RIFCSPLOWO2_02_FULL_49_13]	1083391086
OGM97652.1	73	A2735_03545	bacteria	Candidatus Yanofskybacteria bacterium RIFCSPHIGHO2_01_FULL_41_21	hypothetical protein A2735_03545 [Candidatus Yanofskybacteria bacterium RIFCSPHIGHO2_01_FULL_41_21]	1084430131
OGH69108.1	71	A2754_02760	bacteria	Candidatus Magasanikbacteria bacterium RIFCSPHIGHO2_01_FULL_47_8	general stress protein [Candidatus Magasanikbacteria bacterium RIFCSPHIGHO2_01_FULL_47_8]	1083681767
OHB25461.1	70	A2542_00535	bacteria	Parcubacteria group bacterium RIFOXYD2_FULL_52_8	stress-induced protein [Parcubacteria group bacterium RIFOXYD2_FULL_52_8]	1088269929

[illegible]

AHX13536.1	108	CH75_10225	bacteria>proteobacteria>gammaproteobacteria	Dyella jiangningensis	general stress protein [Dyella jiangningensis]	612503698
SMS12203.1	108	CFBP1590__4617	bacteria>proteobacteria>gammaproteobacteria	Pseudomonas viridiflava	Conserved hypothetical protein [Pseudomonas viridiflava]	1206261666
ARQ90003.1	107	A7326_10500	bacteria>proteobacteria>gammaproteobacteria	Stenotrophomonas maltophilia	general stress protein [Stenotrophomonas maltophilia]	1192694603
CAQ46016.1	107	Smlt2536	bacteria>proteobacteria>gammaproteobacteria	Stenotrophomonas maltophilia K279a	conserved hypothetical protein [Stenotrophomonas maltophilia K279a]	190012388
SNW10238.1	107	SAMEA4076705_02527	bacteria>proteobacteria>gammaproteobacteria	Stenotrophomonas maltophilia	General stress protein [Stenotrophomonas maltophilia]	1231784370
ABE60094.1	105	Csal_2747	bacteria>proteobacteria>gammaproteobacteria	Chromohalobacter salexigens DSM 3043	hypothetical protein Csal_2747 [Chromohalobacter salexigens DSM 3043]	91797955
AGI07344.1	103	XCAW_01544	bacteria>proteobacteria>gammaproteobacteria	Xanthomonas citri subsp. citri Aw12879	Hypothetical Protein XCAW_01544 [Xanthomonas citri subsp. citri Aw12879]	471210594
AKK01180.1	103	VM99_25105	bacteria>proteobacteria>gammaproteobacteria	Pseudomonas chlororaphis	hypothetical protein VM99_25105 [Pseudomonas chlororaphis]	829495307
ARZ76052.1	103	CCR98_18375	bacteria>proteobacteria>gammaproteobacteria	Stenotrophomonas sp. WZN-1	general stress protein [Stenotrophomonas sp. WZN-1]	1206172678
ARQ91489.1	101	A7326_18505	bacteria>proteobacteria>gammaproteobacteria	Stenotrophomonas maltophilia	general stress protein [Stenotrophomonas maltophilia]	1192696089
ASE54317.1	101	CEQ03_17325	bacteria>proteobacteria>gammaproteobacteria	Stenotrophomonas maltophilia	general stress protein [Stenotrophomonas maltophilia]	1210982580
CAQ47531.1	101	Smlt4140	bacteria>proteobacteria>gammaproteobacteria	Stenotrophomonas maltophilia K279a	conserved hypothetical protein [Stenotrophomonas maltophilia K279a]	190013891
SNW13817.1	101	SAMEA4076705_04117	bacteria>proteobacteria>gammaproteobacteria	Stenotrophomonas maltophilia	General stress protein [Stenotrophomonas maltophilia]	1231785926
ALA84012.1	100	VN11_19130	bacteria>proteobacteria>gammaproteobacteria	Stenotrophomonas maltophilia	general stress protein [Stenotrophomonas maltophilia]	922683216
AMU97528.1	100	TP37_04940	bacteria>proteobacteria>gammaproteobacteria	Xanthomonas citri pv. aurantifolii	stress-induced protein [Xanthomonas citri pv. aurantifolii]	1015415731
ABP85066.1	98	Pmen_2308	bacteria>proteobacteria>gammaproteobacteria	Pseudomonas mendocina ymp	hypothetical protein Pmen_2308 [Pseudomonas mendocina ymp]	145575534
AHX13203.1	96	CH75_08105	bacteria>proteobacteria>gammaproteobacteria	Dyella jiangningensis	general stress protein [Dyella jiangningensis]	612503365
ACO76491.1	95	Avin_02300	bacteria>proteobacteria>gammaproteobacteria	Azotobacter vinelandii DJ	conserved hypothetical protein [Azotobacter vinelandii DJ]	226717320
AGK17380.1	95	AvCA_02300	bacteria>proteobacteria>gammaproteobacteria	Azotobacter vinelandii CA	hypothetical protein AvCA_02300 [Azotobacter vinelandii CA]	482534253
AGK19161.1	95	AvCA6_02300	bacteria>proteobacteria>gammaproteobacteria	Azotobacter vinelandii CA6	hypothetical protein AvCA6_02300 [Azotobacter vinelandii CA6]	482536035
APR17472.1	94	BI315_07525	bacteria>proteobacteria>gammaproteobacteria	Xanthomonas citri pv. citri	stress-induced protein [Xanthomonas citri pv. citri]	1125893224
APR22425.1	94	BI316_18910	bacteria>proteobacteria>gammaproteobacteria	Xanthomonas citri pv. citri	stress-induced protein [Xanthomonas citri pv. citri]	1125898181
ARR15103.1	94	B7L66_11995	bacteria>proteobacteria>gammaproteobacteria	Xanthomonas citri pv. citri	stress-induced protein [Xanthomonas citri pv. citri]	1192727144
ABP85017.1	93	Pmen_2258	bacteria>proteobacteria>gammaproteobacteria	Pseudomonas mendocina ymp	hypothetical protein Pmen_2258 [Pseudomonas mendocina ymp]	145575485
CAP51091.1	92	XCCB100_1741	bacteria>proteobacteria>gammaproteobacteria	Xanthomonas campestris pv. campestris	hypothetical protein XCCB100_1741 [Xanthomonas campestris pv. campestris]	167732897
AJE17095.1	91	CL52_19330	bacteria>proteobacteria>gammaproteobacteria	Pseudomonas balearica DSM 6083	glucose starvation-inducible protein B [Pseudomonas balearica DSM 6083]	747152729
AAM37408.1	87	XAC2557	bacteria>proteobacteria>gammaproteobacteria	Xanthomonas citri pv. citri str. 306	conserved hypothetical protein [Xanthomonas citri pv. citri str. 306]	21108829
CDF62081.1	87	XFF4834R_chr25810	bacteria>proteobacteria>gammaproteobacteria	Xanthomonas citri pv. fuscans	Conserved hypothetical protein [Xanthomonas citri pv. fuscans]	549146362
AAM37009.1	85	XAC2156	bacteria>proteobacteria>gammaproteobacteria	Xanthomonas citri pv. citri str. 306	conserved hypothetical protein [Xanthomonas citri pv. citri str. 306]	21108386
AAN68876.1	85	PP_3269	bacteria>proteobacteria>gammaproteobacteria	Pseudomonas putida KT2440	conserved protein of unknown function [Pseudomonas putida KT2440]	24984906
ACD59889.1	85	PXO_01502	bacteria>proteobacteria>gammaproteobacteria	Xanthomonas oryzae pv. oryzae PXO99A	hypothetical protein PXO_01502 [Xanthomonas oryzae pv. oryzae PXO99A]	188521944
AEL07185.1	85	XCR_2300	bacteria>proteobacteria>gammaproteobacteria	Xanthomonas campestris pv. raphani 756C	conserved hypothetical protein [Xanthomonas campestris pv. raphani 756C]	341937046
AEO42348.1	85	XACM_2076	bacteria>proteobacteria>gammaproteobacteria	Xanthomonas axonopodis pv. citrumelo F1	hypothetical protein XACM_2076 [Xanthomonas axonopodis pv. citrumelo F1]	346649724
AEQ96592.1	85	XOC_2461	bacteria>proteobacteria>gammaproteobacteria	Xanthomonas oryzae pv. oryzicola BLS256	hypothetical protein XOC_2461 [Xanthomonas oryzae pv. oryzicola BLS256]	353462313
AFK72604.1	85	YSA_10767	bacteria>proteobacteria>gammaproteobacteria	Pseudomonas putida ND6	hypothetical protein YSA_10767 [Pseudomonas putida ND6]	388563463
AGH77641.1	85	XAC29_10900	bacteria>proteobacteria>gammaproteobacteria	Xanthomonas axonopodis Xac29-1	hypothetical protein XAC29_10900 [Xanthomonas axonopodis Xac29-1]	469767224
AJQ82640.1	85	AZ54_08500	bacteria>proteobacteria>gammaproteobacteria	Xanthomonas oryzae pv. oryzae PXO86	stress-induced protein [Xanthomonas oryzae pv. oryzae PXO86]	765201342
AMU98481.1	85	TP37_10555	bacteria>proteobacteria>gammaproteobacteria	Xanthomonas citri pv. aurantifolii	stress-induced protein [Xanthomonas citri pv. aurantifolii]	1015416684
AMV03137.1	85	TP50_12310	bacteria>proteobacteria>gammaproteobacteria	Xanthomonas citri pv. aurantifolii	stress-induced protein [Xanthomonas citri pv. aurantifolii]	1015421342
AMV08608.1	85	AC028_18715	bacteria>proteobacteria>gammaproteobacteria	Xanthomonas citri pv. aurantifolii	stress-induced protein [Xanthomonas citri pv. aurantifolii]	1015426817
AOD15011.1	85	BER92_09985	bacteria>proteobacteria>gammaproteobacteria	Xanthomonas fragariae	stress-induced protein [Xanthomonas fragariae]	1052528460
AOY63059.1	85	BHE84_13470	bacteria>proteobacteria>gammaproteobacteria	Xanthomonas citri pv. glycines str. 8ra	stress-induced protein [Xanthomonas citri pv. glycines str. 8ra]	1092209125
AOY65673.1	85	BHE83_03165	bacteria>proteobacteria>gammaproteobacteria	Xanthomonas campestris pv. vesicatoria str. 85-10	stress-induced protein [Xanthomonas campestris pv. vesicatoria str. 85-10]	1092212232
APO90651.1	85	BJD11_11940	bacteria>proteobacteria>gammaproteobacteria	Xanthomonas euvesicatoria	stress-induced protein [Xanthomonas euvesicatoria]	1120740204
APO93932.1	85	BI313_04325	bacteria>proteobacteria>gammaproteobacteria	Xanthomonas vesicatoria	stress-induced protein [Xanthomonas vesicatoria]	1120743488
APO98687.1	85	BJD13_06080	bacteria>proteobacteria>gammaproteobacteria	Xanthomonas perforans	stress-induced protein [Xanthomonas perforans]	1120748248
APP74180.1	85	BJD12_01630	bacteria>proteobacteria>gammaproteobacteria	Xanthomonas vesicatoria ATCC 35937	stress-induced protein [Xanthomonas vesicatoria ATCC 35937]	1120824840
APP84865.1	85	BI317_12555	bacteria>proteobacteria>gammaproteobacteria	Xanthomonas gardneri	stress-induced protein [Xanthomonas gardneri]	1120835531
APR13748.1	85	BI315_01610	bacteria>proteobacteria>gammaproteobacteria	Xanthomonas citri pv. citri	stress-induced protein [Xanthomonas citri pv. citri]	1125889500
APR20302.1	85	BI316_12985	bacteria>proteobacteria>gammaproteobacteria	Xanthomonas citri pv. citri	stress-induced protein [Xanthomonas citri pv. citri]	1125896058
AQS75246.1	85	XPE_02025	bacteria>proteobacteria>gammaproteobacteria	Xanthomonas perforans 91-118	stress-induced protein [Xanthomonas perforans 91-118]	1148989523
ARR12538.1	85	B7L66_10070	bacteria>proteobacteria>gammaproteobacteria	Xanthomonas citri pv. citri	stress-induced protein [Xanthomonas citri pv. citri]	1192724579
ARV23144.1	85	A9D66_11155	bacteria>proteobacteria>gammaproteobacteria	Xanthomonas citri pv. glycines str. 12-2	stress-induced protein [Xanthomonas citri pv. glycines str. 12-2]	1199793313
ASK92125.1	85	XcvCFBP7111P_11930	bacteria>proteobacteria>gammaproteobacteria	Xanthomonas citri pv. vignicola	stress-induced protein [Xanthomonas citri pv. vignicola]	1215509332
ASW45181.1	85	XJ27_03665	bacteria>proteobacteria>gammaproteobacteria	Xanthomonas hortorum	stress-induced protein [Xanthomonas hortorum]	1239648845
BAE69694.1	85	XOO2939	bacteria>proteobacteria>gammaproteobacteria	Xanthomonas oryzae pv. oryzae MAFF 311018	conserved hypothetical protein [Xanthomonas oryzae pv. oryzae MAFF 311018]	84368536
CAJ23774.1	85	XCV2097	bacteria>proteobacteria>gammaproteobacteria	Xanthomonas campestris pv. vesicatoria str. 85-10	conserved hypothetical protein [Xanthomonas campestris pv. vesicatoria str. 85-10]	78036083
CAP51674.1	85	XCCB100_2319	bacteria>proteobacteria>gammaproteobacteria	Xanthomonas campestris pv. campestris	hypothetical protein XCCB100_2319 [Xanthomonas campestris pv. campestris]	167733473
CDF61796.1	85	XFF4834R_chr22720	bacteria>proteobacteria>gammaproteobacteria	Xanthomonas citri pv. fuscans	conserved hypothetical proteine [Xanthomonas citri pv. fuscans]	549146077
ALV92611.1	84	LK04_10850	bacteria>proteobacteria>gammaproteobacteria	Pantoea vagans	stress-induced bacterial acidophilic repeat motif [Pantoea vagans]	972389141
AAM42095.1	83	XCC2823	bacteria>proteobacteria>gammaproteobacteria	Xanthomonas campestris pv. campestris str. ATCC 33913	conserved hypothetical protein [Xanthomonas campestris pv. campestris str. ATCC 33913]	21114015
AMA45113.1	83	APT63_05420	bacteria>proteobacteria>gammaproteobacteria	Pseudomonas monteilii	hypothetical protein APT63_05420 [Pseudomonas monteilii]	982199874
ADY81355.1	82	BDGL_000769	bacteria>proteobacteria>gammaproteobacteria	Acinetobacter pittii PHEA-2	hypothetical protein BDGL_000769 [Acinetobacter pittii PHEA-2]	325121832
AIX74026.1	81	PSNIH2_09685	bacteria>proteobacteria>gammaproteobacteria	Pantoea sp. PSNIH2	stress-induced protein [Pantoea sp. PSNIH2]	723241390
ADO09657.1	80	yciG	bacteria>proteobacteria>gammaproteobacteria	Pantoea vagans C9-1	hypothetical protein Pvag_1467 [Pantoea vagans C9-1]	308057485
AFJ99425.1	79	ES15_1852	bacteria>proteobacteria>gammaproteobacteria	Cronobacter sakazakii ES15	hypothetical protein ES15_1852 [Cronobacter sakazakii ES15]	387851328
AGE86218.1	79	CSSP291_08115	bacteria>proteobacteria>gammaproteobacteria	Cronobacter sakazakii SP291	hypothetical protein CSSP291_08115 [Cronobacter sakazakii SP291]	449098184
AHB70246.1	79	P262_02645	bacteria>proteobacteria>gammaproteobacteria	Cronobacter malonaticus	hypothetical protein P262_02645 [Cronobacter malonaticus]	564117925
AKE95876.1	79	CSK29544_02925	bacteria>proteobacteria>gammaproteobacteria	Cronobacter sakazakii	hypothetical protein CSK29544_02925 [Cronobacter sakazakii]	816222488
ALB70570.1	79	AFK63_08115	bacteria>proteobacteria>gammaproteobacteria	Cronobacter muytjensii ATCC 51329	stress-induced protein [Cronobacter muytjensii ATCC 51329]	924334551
ALX78537.1	79	AFK66_008765	bacteria>proteobacteria>gammaproteobacteria	Cronobacter malonaticus LMG 23826	stress-induced protein [Cronobacter malonaticus LMG 23826]	973755628
CBA31194.1	79	yciG	bacteria>proteobacteria>gammaproteobacteria	Cronobacter turicensis z3032	Uncharacterized protein yciG [Cronobacter turicensis z3032]	260217263
AAN42875.1	78	yciG	bacteria>proteobacteria>gammaproteobacteria	Shigella flexneri 2a str. 301	conserved hypothetical protein [Shigella flexneri 2a str. 301]	24051570
AEL08061.1	78	XCR_3197	bacteria>proteobacteria>gammaproteobacteria	Xanthomonas campestris pv. raphani 756C	conserved hypothetical protein [Xanthomonas campestris pv. raphani 756C]	341937922
AFD50761.1	78	FSC454_06385	bacteria>proteobacteria>gammaproteobacteria	Francisella hispaniensis FSC454	hypothetical protein FSC454_06385 [Francisella hispaniensis FSC454]	1104559225
CAP50686.1	78	XCCB100_1336	bacteria>proteobacteria>gammaproteobacteria	Xanthomonas campestris pv. campestris	hypothetical protein XCCB100_1336 [Xanthomonas campestris pv. campestris]	167732492
AAM41703.1	77	XCC2425	bacteria>proteobacteria>gammaproteobacteria	Xanthomonas campestris pv. campestris str. ATCC 33913	conserved hypothetical protein [Xanthomonas campestris pv. campestris str. ATCC 33913]	21113582
ABU77427.1	77	ESA_02178	bacteria>proteobacteria>gammaproteobacteria	Cronobacter sakazakii ATCC BAA-894	hypothetical protein ESA_02178 [Cronobacter sakazakii ATCC BAA-894]	156532601
AFJ99899.1	77	ES15_2326	bacteria>proteobacteria>gammaproteobacteria	Cronobacter sakazakii ES15	hypothetical protein ES15_2326 [Cronobacter sakazakii ES15]	387851802
AKE96409.1	77	CSK29544_03462	bacteria>proteobacteria>gammaproteobacteria	Cronobacter sakazakii	hypothetical protein CSK29544_03462 [Cronobacter sakazakii]	816223021
ALB66907.1	77	AFK67_10580	bacteria>proteobacteria>gammaproteobacteria	Cronobacter dublinensis subsp. dublinensis LMG 23823	stress-induced protein [Cronobacter dublinensis subsp. dublinensis LMG 23823]	924330886
AQW68701.1	75	B2J77_10995	bacteria>proteobacteria>gammaproteobacteria	Pseudomonas parafulva	stress-induced protein [Pseudomonas parafulva]	1153110260
AGN78868.1	72	L483_17755	bacteria>proteobacteria>gammaproteobacteria	Pseudomonas putida H8234	stress-induced protein [Pseudomonas putida H8234]	511521327
AHC82285.1	72	X969_09990	bacteria>proteobacteria>gammaproteobacteria	Pseudomonas monteilii SB3078	stress-induced protein [Pseudomonas monteilii SB3078]	567350088
AHC87663.1	72	X970_09650	bacteria>proteobacteria>gammaproteobacteria	Pseudomonas monteilii SB3101	stress-induced protein [Pseudomonas monteilii SB3101]	567357675
AHI77098.1	72	DW66_2586	bacteria>proteobacteria>gammaproteobacteria	Pseudomonas putida	stress-induced protein [Pseudomonas putida]	635294356
AMO49793.1	72	yciG	bacteria>proteobacteria>gammaproteobacteria	Enterobacter sp. FY-07	Stress-induced protein, KGG, repeat protein [Enterobacter sp. FY-07]	1003371003
AIZ32805.1	71	NJ69_07235	bacteria>proteobacteria>gammaproteobacteria	Pseudomonas parafulva	stress-induced protein [Pseudomonas parafulva]	730588422
AFO51426.1	69	T1E_5605	bacteria>proteobacteria>gammaproteobacteria	Pseudomonas putida DOT-T1E	hypothetical protein T1E_5605 [Pseudomonas putida DOT-T1E]	397335067
AGN79192.1	69	L483_17335	bacteria>proteobacteria>gammaproteobacteria	Pseudomonas putida H8234	stress-induced protein [Pseudomonas putida H8234]	511521651
AHC82790.1	69	X969_12855	bacteria>proteobacteria>gammaproteobacteria	Pseudomonas monteilii SB3078	stress-induced protein [Pseudomonas monteilii SB3078]	567350593
AHC88166.1	69	X970_12500	bacteria>proteobacteria>gammaproteobacteria	Pseudomonas monteilii SB3101	stress-induced protein [Pseudomonas monteilii SB3101]	567358178
AHZ77471.1	69	DW66_2962	bacteria>proteobacteria>gammaproteobacteria	Pseudomonas putida	Conidiation-specific protein [Pseudomonas putida]	635294729
AIN58506.1	69	O165_009460	bacteria>proteobacteria>gammaproteobacteria	Pseudomonas soli	stress-induced protein [Pseudomonas soli]	684196277
AJG13484.1	69	RK21_01976	bacteria>proteobacteria>gammaproteobacteria	Pseudomonas plecoglossicida	hypothetical protein RK21_01976 [Pseudomonas plecoglossicida]	752310875
AOE40825.1	68	BEE12_13760	bacteria>proteobacteria>gammaproteobacteria	Pantoea agglomerans	hypothetical protein BEE12_13760 [Pantoea agglomerans]	1057483931
CUU24068.1	68	EM595_1834	bacteria>proteobacteria>gammaproteobacteria	Erwinia gerundensis	hypothetical protein EM595_1834 [Erwinia gerundensis]	972720631
AIN58454.1	67	O165_009180	bacteria>proteobacteria>gammaproteobacteria	Pseudomonas soli	stress-induced protein [Pseudomonas soli]	684196225
AJA71212.1	67	PSNIH1_p01730	bacteria>proteobacteria>gammaproteobacteria	Pantoea sp. PSNIH1	stress-induced protein (plasmid) [Pantoea sp. PSNIH1]	736031586
ANK06524.1	67	WLH_05263	bacteria>proteobacteria>gammaproteobacteria	Escherichia coli O25b:H4	hypothetical protein WLH_05263 [Escherichia coli O25b:H4]	1036415189
BAB35254.1	67	ECs1831	bacteria>proteobacteria>gammaproteobacteria	Escherichia coli O157:H7 str. Sakai	hypothetical protein [Escherichia coli O157:H7 str. Sakai]	13361296
BAN97223.1	67	E05_24570	bacteria>proteobacteria>gammaproteobacteria	Plautia stali symbiont	stress-induced protein, KGG, repeat [Plautia stali symbiont]	549070261
AEO42798.1	66	XACM_2538	bacteria>proteobacteria>gammaproteobacteria	Xanthomonas axonopodis pv. citrumelo F1	hypothetical protein XACM_2538 [Xanthomonas axonopodis pv. citrumelo F1]	346650174
AGH78047.1	66	XAC29_13025	bacteria>proteobacteria>gammaproteobacteria	Xanthomonas axonopodis Xac29-1	hypothetical protein XAC29_13025 [Xanthomonas axonopodis Xac29-1]	469767630
AGI08026.1	66	XCAW_02237	bacteria>proteobacteria>gammaproteobacteria	Xanthomonas citri subsp. citri Aw12879	Hypothetical Protein XCAW_02237 [Xanthomonas citri subsp. citri Aw12879]	471211276
ALZ85205.1	66	APT59_13755	bacteria>proteobacteria>gammaproteobacteria	Pseudomonas oryzihabitans	general stress protein [Pseudomonas oryzihabitans]	975032413
AMU98992.1	66	TP37_13555	bacteria>proteobacteria>gammaproteobacteria	Xanthomonas citri pv. aurantifolii	general stress protein [Xanthomonas citri pv. aurantifolii]	1015417195
AMV04241.1	66	TP50_18730	bacteria>proteobacteria>gammaproteobacteria	Xanthomonas citri pv. aurantifolii	general stress protein [Xanthomonas citri pv. aurantifolii]	1015422446
AMV08881.1	66	AC028_20260	bacteria>proteobacteria>gammaproteobacteria	Xanthomonas citri pv. aurantifolii	general stress protein [Xanthomonas citri pv. aurantifolii]	1015427090
AOD14386.1	66	BER92_06150	bacteria>proteobacteria>gammaproteobacteria	Xanthomonas fragariae	general stress protein [Xanthomonas fragariae]	1052527835
AOY62612.1	66	BHE84_10900	bacteria>proteobacteria>gammaproteobacteria	Xanthomonas citri pv. glycines str. 8ra	general stress protein [Xanthomonas citri pv. glycines str. 8ra]	1092208678
AOY66236.1	66	BHE83_06425	bacteria>proteobacteria>gammaproteobacteria	Xanthomonas campestris pv. vesicatoria str. 85-10	general stress protein [Xanthomonas campestris pv. vesicatoria str. 85-10]	1092212795
APO90189.1	66	BJD11_09110	bacteria>proteobacteria>gammaproteobacteria	Xanthomonas euvesicatoria	general stress protein [Xanthomonas euvesicatoria]	1120739742
APO98277.1	66	BJD13_03690	bacteria>proteobacteria>gammaproteobacteria	Xanthomonas perforans	general stress protein [Xanthomonas perforans]	1120747838
APQ10839.1	66	BJP27_04715	bacteria>proteobacteria>gammaproteobacteria	Pseudomonas psychrotolerans	general stress protein [Pseudomonas psychrotolerans]	1123413747
APR14099.1	66	BI315_03740	bacteria>proteobacteria>gammaproteobacteria	Xanthomonas citri pv. citri	general stress protein [Xanthomonas citri pv. citri]	1125889851
APR20652.1	66	BI316_15115	bacteria>proteobacteria>gammaproteobacteria	Xanthomonas citri pv. citri	general stress protein [Xanthomonas citri pv. citri]	1125896408
AQS78493.1	66	XPE_21545	bacteria>proteobacteria>gammaproteobacteria	Xanthomonas perforans 91-118	general stress protein [Xanthomonas perforans 91-118]	1148992770
ARR12181.1	66	B7L66_07910	bacteria>proteobacteria>gammaproteobacteria	Xanthomonas citri pv. citri	general stress protein [Xanthomonas citri pv. citri]	1192724222
ARV23663.1	66	A9D66_13910	bacteria>proteobacteria>gammaproteobacteria	Xanthomonas citri pv. glycines str. 12-2	general stress protein [Xanthomonas citri pv. glycines str. 12-2]	1199793832
ASK91891.1	66	XcvCFBP7111P_10555	bacteria>proteobacteria>gammaproteobacteria	Xanthomonas citri pv. vignicola	general stress protein [Xanthomonas citri pv. vignicola]	1215509098
AEL07602.1	65	XCR_2729	bacteria>proteobacteria>gammaproteobacteria	Xanthomonas campestris pv. raphani 756C	conserved hypothetical protein [Xanthomonas campestris pv. raphani 756C]	341937463
AIX49241.1	65	PSNIH1_02765	bacteria>proteobacteria>gammaproteobacteria	Pantoea sp. PSNIH1	stress-induced protein [Pantoea sp. PSNIH1]	723255220
AJE22515.1	64	Achr_31060	bacteria>proteobacteria>gammaproteobacteria	Azotobacter chroococcum NCIMB 8003	Hypothetical protein Achr_31060 [Azotobacter chroococcum NCIMB 8003]	747128374
ASL27665.1	64	ACG10_16225	bacteria>proteobacteria>gammaproteobacteria	Azotobacter chroococcum	general stress protein [Azotobacter chroococcum]	1216350530
ACO80557.1	63	Avin_44390	bacteria>proteobacteria>gammaproteobacteria	Azotobacter vinelandii DJ	conserved hypothetical protein [Azotobacter vinelandii DJ]	226721386
AGK14353.1	63	AvCA_44390	bacteria>proteobacteria>gammaproteobacteria	Azotobacter vinelandii CA	hypothetical protein AvCA_44390 [Azotobacter vinelandii CA]	482531226
AGK22007.1	63	AvCA6_44390	bacteria>proteobacteria>gammaproteobacteria	Azotobacter vinelandii CA6	hypothetical protein AvCA6_44390 [Azotobacter vinelandii CA6]	482538881
AHF74292.1	63	SOPEG_2640	bacteria>proteobacteria>gammaproteobacteria	Candidatus Sodalis pierantonius str. SOPE	putative cytoplasmic protein [Candidatus Sodalis pierantonius str. SOPE]	573018755
AHF76696.1	63	Sant_1641	bacteria>proteobacteria>gammaproteobacteria	Sodalis praecaptivus	hypothetical protein Sant_1641 [Sodalis praecaptivus]	573021160
AMM02924.1	63	PP_5593	bacteria>proteobacteria>gammaproteobacteria	Pseudomonas putida KT2440	conserved protein of unknown function [Pseudomonas putida KT2440]	1001555848
ATB67738.1	63	CLJ08_25250	bacteria>proteobacteria>gammaproteobacteria	Pseudomonas mosselii	stress-induced protein [Pseudomonas mosselii]	1243308310
CAK15855.1	63	PSEEN3087	bacteria>proteobacteria>gammaproteobacteria	Pseudomonas entomophila L48	conserved hypothetical protein [Pseudomonas entomophila L48]	95111135
AHF74446.1	62	SOPEG_2917	bacteria>proteobacteria>gammaproteobacteria	Candidatus Sodalis pierantonius str. SOPE	hypothetical protein SOPEG_2917 [Candidatus Sodalis pierantonius str. SOPE]	573018909
AHF76337.1	62	Sant_1275	bacteria>proteobacteria>gammaproteobacteria	Sodalis praecaptivus	hypothetical protein Sant_1275 [Sodalis praecaptivus]	573020801
AHF78562.1	62	Sant_3580	bacteria>proteobacteria>gammaproteobacteria	Sodalis praecaptivus	hypothetical protein Sant_3580 [Sodalis praecaptivus]	573023026
AIE65325.1	62	ECNIH2_18635	bacteria>proteobacteria>gammaproteobacteria	Enterobacter cloacae ECNIH2	hypothetical protein ECNIH2_18635 [Enterobacter cloacae ECNIH2]	662715746
AJB72184.1	62	LI64_17220	bacteria>proteobacteria>gammaproteobacteria	Enterobacter hormaechei subsp. hormaechei	stress-induced bacterial acidophilic repeat motif [Enterobacter hormaechei subsp. hormaechei]	742987550
AJB83215.1	62	LI66_18315	bacteria>proteobacteria>gammaproteobacteria	Enterobacter hormaechei subsp. xiangfangensis	stress-induced bacterial acidophilic repeat motif [Enterobacter hormaechei subsp. xiangfangensis]	742992614
ALA03658.1	62	LI63_020620	bacteria>proteobacteria>gammaproteobacteria	Enterobacter hormaechei subsp. xiangfangensis	stress-induced bacterial acidophilic repeat motif [Enterobacter hormaechei subsp. xiangfangensis]	918060550
ANS16485.1	62	AB284_07475	bacteria>proteobacteria>gammaproteobacteria	Enterobacter hormaechei	stress-induced bacterial acidophilic repeat motif [Enterobacter hormaechei]	1043695548
AOP79447.1	62	BFV68_18040	bacteria>proteobacteria>gammaproteobacteria	Enterobacter hormaechei subsp. steigerwaltii	stress-induced bacterial acidophilic repeat motif [Enterobacter hormaechei subsp. steigerwaltii]	1067033072
AOP92683.1	62	BFV63_17945	bacteria>proteobacteria>gammaproteobacteria	Enterobacter hormaechei subsp. xiangfangensis	stress-induced bacterial acidophilic repeat motif [Enterobacter hormaechei subsp. xiangfangensis]	1067046312
ASP00906.1	62	MS7884_2650	bacteria>proteobacteria>gammaproteobacteria	Enterobacter cloacae	general stress protein [Enterobacter cloacae]	1226078513
ABP61790.1	61	Ent638_3126	bacteria>proteobacteria>gammaproteobacteria	Enterobacter sp. 638	hypothetical protein Ent638_3126 [Enterobacter sp. 638]	145319643
ACO77344.1	61	Avin_11130	bacteria>proteobacteria>gammaproteobacteria	Azotobacter vinelandii DJ	hypothetical protein Avin_11130 [Azotobacter vinelandii DJ]	226718173
ADC58353.1	61	Kvar_2462	bacteria>proteobacteria>gammaproteobacteria	Klebsiella variicola At-22	Stress-induced protein, KGG, repeat protein [Klebsiella variicola At-22]	288890035
ADC59096.1	61	Kvar_3211	bacteria>proteobacteria>gammaproteobacteria	Klebsiella variicola At-22	Stress-induced protein, KGG, repeat protein [Klebsiella variicola At-22]	288890778
ADF61594.1	61	ECL_02042	bacteria>proteobacteria>gammaproteobacteria	Enterobacter cloacae subsp. cloacae ATCC 13047	hypothetical protein ECL_02042 [Enterobacter cloacae subsp. cloacae ATCC 13047]	295056856
AEG98668.1	61	EAE_18805	bacteria>proteobacteria>gammaproteobacteria	Klebsiella aerogenes KCTC 2190	hypothetical protein EAE_18805 [Klebsiella aerogenes KCTC 2190]	334736293
AEW61505.1	61	KPHS_28070	bacteria>proteobacteria>gammaproteobacteria	Klebsiella pneumoniae subsp. pneumoniae HS11286	hypothetical protein KPHS_28070 [Klebsiella pneumoniae subsp. pneumoniae HS11286]	364518377
AEX06153.1	61	KOX_22165	bacteria>proteobacteria>gammaproteobacteria	Klebsiella michiganensis KCTC 1686	hypothetical protein KOX_22165 [Klebsiella michiganensis KCTC 1686]	365910700
AFN32756.1	61	A225_3311	bacteria>proteobacteria>gammaproteobacteria	Klebsiella michiganensis E718	Conidiation-specific protein 10 [Klebsiella michiganensis E718]	394344635
AGK17081.1	61	AvCA_11130	bacteria>proteobacteria>gammaproteobacteria	Azotobacter vinelandii CA	hypothetical protein AvCA_11130 [Azotobacter vinelandii CA]	482533954
AGK19736.1	61	AvCA6_11130	bacteria>proteobacteria>gammaproteobacteria	Azotobacter vinelandii CA6	hypothetical protein AvCA6_11130 [Azotobacter vinelandii CA6]	482536610
AHY13226.1	61	CFNIH1_17360	bacteria>proteobacteria>gammaproteobacteria	Citrobacter freundii CFNIH1	hypothetical protein CFNIH1_17360 [Citrobacter freundii CFNIH1]	619737987
AID90564.1	61	KONIH1_16225	bacteria>proteobacteria>gammaproteobacteria	Klebsiella oxytoca KONIH1	hypothetical protein KONIH1_16225 [Klebsiella oxytoca KONIH1]	660570644
AIE70744.1	61	HR38_20485	bacteria>proteobacteria>gammaproteobacteria	Klebsiella michiganensis	hypothetical protein HR38_20485 [Klebsiella michiganensis]	662710435
AIX72357.1	61	PSNIH2_00265	bacteria>proteobacteria>gammaproteobacteria	Pantoea sp. PSNIH2	hypothetical protein PSNIH2_00265 [Pantoea sp. PSNIH2]	723239721
AJA96694.1	61	KR75_18120	bacteria>proteobacteria>gammaproteobacteria	Klebsiella variicola	stress-induced bacterial acidophilic repeat motif [Klebsiella variicola]	742128559
AJA97433.1	61	KR75_22000	bacteria>proteobacteria>gammaproteobacteria	Klebsiella variicola	stress-induced bacterial acidophilic repeat motif [Klebsiella variicola]	742129298
AJF73369.1	61	TE10_15450	bacteria>proteobacteria>gammaproteobacteria	Raoultella ornithinolytica	stress-induced bacterial acidophilic repeat motif [Raoultella ornithinolytica]	751397283
AJF73594.1	61	TE10_16715	bacteria>proteobacteria>gammaproteobacteria	Raoultella ornithinolytica	stress-induced bacterial acidophilic repeat motif [Raoultella ornithinolytica]	751397508
ANK99863.1	61	AB190_04505	bacteria>proteobacteria>gammaproteobacteria	Enterobacter asburiae	stress-induced bacterial acidophilic repeat motif [Enterobacter asburiae]	828922707
AKL35983.1	61	AB185_19685	bacteria>proteobacteria>gammaproteobacteria	Klebsiella oxytoca	stress-induced bacterial acidophilic repeat motif [Klebsiella oxytoca]	828962314
ALX95461.1	61	APT61_05365	bacteria>proteobacteria>gammaproteobacteria	Leclercia adecarboxylata	stress-induced bacterial acidophilic repeat motif [Leclercia adecarboxylata]	975036936
AMA03325.1	61	ACJ69_06560	bacteria>proteobacteria>gammaproteobacteria	Enterobacter asburiae	stress-induced bacterial acidophilic repeat motif [Enterobacter asburiae]	975831429
AMG54216.1	61	AL524_14570	bacteria>proteobacteria>gammaproteobacteria	Citrobacter amalonaticus	stress-induced acidophilic repeat motif-containing protein [Citrobacter amalonaticus]	991890997
AMH07693.1	61	AL511_00445	bacteria>proteobacteria>gammaproteobacteria	Klebsiella aerogenes	stress-induced bacterial acidophilic repeat motif [Klebsiella aerogenes]	991944492
AML35399.1	61	EAG7_01653	bacteria>proteobacteria>gammaproteobacteria	Klebsiella aerogenes	hypothetical protein EAG7_01653 [Klebsiella aerogenes]	1000411153
AMQ62083.1	61	AL497_20995	bacteria>proteobacteria>gammaproteobacteria	Klebsiella aerogenes	stress-induced acidophilic repeat motif-containing protein [Klebsiella aerogenes]	1006710914
AMR15586.1	61	AVR78_15075	bacteria>proteobacteria>gammaproteobacteria	Klebsiella quasipneumoniae	stress-induced bacterial acidophilic repeat motif [Klebsiella quasipneumoniae]	1008883912
AMR16160.1	61	AVR78_18230	bacteria>proteobacteria>gammaproteobacteria	Klebsiella quasipneumoniae	stress-induced bacterial acidophilic repeat motif [Klebsiella quasipneumoniae]	1008884486
AMX05269.1	61	A0R60_0962	bacteria>proteobacteria>gammaproteobacteria	Enterobacter asburiae	hypothetical protein A0R60_0962 [Enterobacter asburiae]	1016274690
ANG93846.1	61	A8A57_16140	bacteria>proteobacteria>gammaproteobacteria	Lelliottia amnigena	stress-induced acidophilic repeat motif-containing protein [Lelliottia amnigena]	1032542267
AOL11796.1	61	EnteroDNA1_00571	bacteria>proteobacteria>gammaproteobacteria	Enterobacter sp. HK169	Stress-induced bacterial acidophilic repeat motif [Enterobacter sp. HK169]	1062061998
AOP86883.1	61	BFV64_11195	bacteria>proteobacteria>gammaproteobacteria	Enterobacter kobei	stress-induced bacterial acidophilic repeat motif [Enterobacter kobei]	1067040510
AOV12518.1	61	BJF97_16330	bacteria>proteobacteria>gammaproteobacteria	Klebsiella sp. LTGPAF-6F	stress-induced bacterial acidophilic repeat motif [Klebsiella sp. LTGPAF-6F]	1073972804
APV30923.1	61	A6P37_13160	bacteria>proteobacteria>gammaproteobacteria	Klebsiella pneumoniae	stress-induced bacterial acidophilic repeat motif [Klebsiella pneumoniae]	1132366710
APV31562.1	61	A6P37_16560	bacteria>proteobacteria>gammaproteobacteria	Klebsiella pneumoniae	stress-induced bacterial acidophilic repeat motif [Klebsiella pneumoniae]	1132367349
APZ06055.1	61	BW195_13865	bacteria>proteobacteria>gammaproteobacteria	Kosakonia cowanii JCM 10956 = DSM 18146	stress-induced protein [Kosakonia cowanii JCM 10956 = DSM 18146]	1137379060
APZ06821.1	61	BW195_18130	bacteria>proteobacteria>gammaproteobacteria	Kosakonia cowanii JCM 10956 = DSM 18146	stress-induced acidophilic repeat motif-containing protein [Kosakonia cowanii JCM 10956 = DSM 18146]	1137379826
AQL15566.1	61	BBD63_09995	bacteria>proteobacteria>gammaproteobacteria	Klebsiella variicola	stress-induced bacterial acidophilic repeat motif [Klebsiella variicola]	1142869019
AQL16271.1	61	BBD63_13765	bacteria>proteobacteria>gammaproteobacteria	Klebsiella variicola	stress-induced bacterial acidophilic repeat motif [Klebsiella variicola]	1142869724
AQL20654.1	61	BBD64_09990	bacteria>proteobacteria>gammaproteobacteria	Klebsiella variicola	stress-induced bacterial acidophilic repeat motif [Klebsiella variicola]	1142874108
AQL21359.1	61	BBD64_13760	bacteria>proteobacteria>gammaproteobacteria	Klebsiella variicola	stress-induced bacterial acidophilic repeat motif [Klebsiella variicola]	1142874813
AQL26419.1	61	BBD65_09990	bacteria>proteobacteria>gammaproteobacteria	Klebsiella variicola	stress-induced bacterial acidophilic repeat motif [Klebsiella variicola]	1142879883
AQL27121.1	61	BBD65_13760	bacteria>proteobacteria>gammaproteobacteria	Klebsiella variicola	stress-induced bacterial acidophilic repeat motif [Klebsiella variicola]	1142880585
ARI09047.1	61	BWI76_16555	bacteria>proteobacteria>gammaproteobacteria	Klebsiella sp. M5al	stress-induced acidophilic repeat motif-containing protein [Klebsiella sp. M5al]	1179081162
ARM21879.1	61	B5G58_07975	bacteria>proteobacteria>gammaproteobacteria	Klebsiella pneumoniae	stress-induced acidophilic repeat motif-containing protein [Klebsiella pneumoniae]	1187398935
ARM22581.1	61	B5G58_11745	bacteria>proteobacteria>gammaproteobacteria	Klebsiella pneumoniae	stress-induced acidophilic repeat motif-containing protein [Klebsiella pneumoniae]	1187399637
ARX03495.1	61	AM359_11875	bacteria>proteobacteria>gammaproteobacteria	Klebsiella pneumoniae	stress-induced acidophilic repeat motif-containing protein [Klebsiella pneumoniae]	1202220514
ARX04275.1	61	AM359_16135	bacteria>proteobacteria>gammaproteobacteria	Klebsiella pneumoniae	stress-induced acidophilic repeat motif-containing protein [Klebsiella pneumoniae]	1202221294
ASC23763.1	61	AM386_19060	bacteria>proteobacteria>gammaproteobacteria	Klebsiella pneumoniae	stress-induced acidophilic repeat motif-containing protein [Klebsiella pneumoniae]	1208679829
ASC24576.1	61	AM386_23510	bacteria>proteobacteria>gammaproteobacteria	Klebsiella pneumoniae	stress-induced bacterial acidophilic repeat motif [Klebsiella pneumoniae]	1208680642
ASD59207.1	61	WM95_11855	bacteria>proteobacteria>gammaproteobacteria	Enterobacter cloacae complex sp. ECNIH7	stress-induced bacterial acidophilic repeat motif [Enterobacter cloacae complex sp. ECNIH7]	1210339276
ASE45379.1	61	CEP69_23075	bacteria>proteobacteria>gammaproteobacteria	Citrobacter braakii	stress-induced acidophilic repeat motif-containing protein [Citrobacter braakii]	1210973630
ASK72447.1	61	CF000_04660	bacteria>proteobacteria>gammaproteobacteria	Klebsiella michiganensis	stress-induced acidophilic repeat motif-containing protein [Klebsiella michiganensis]	1215230610
ASR20212.1	61	AWV58_04940	bacteria>proteobacteria>gammaproteobacteria	Klebsiella quasipneumoniae	stress-induced bacterial acidophilic repeat motif [Klebsiella quasipneumoniae]	1227429012
ASR20851.1	61	AWV58_08435	bacteria>proteobacteria>gammaproteobacteria	Klebsiella quasipneumoniae	stress-induced bacterial acidophilic repeat motif [Klebsiella quasipneumoniae]	1227429651
ASR25660.1	61	AWV59_08570	bacteria>proteobacteria>gammaproteobacteria	Klebsiella quasipneumoniae	stress-induced bacterial acidophilic repeat motif [Klebsiella quasipneumoniae]	1227434429
ASR26300.1	61	AWV59_12075	bacteria>proteobacteria>gammaproteobacteria	Klebsiella quasipneumoniae	stress-induced bacterial acidophilic repeat motif [Klebsiella quasipneumoniae]	1227435069
ASR29248.1	61	AWV60_02050	bacteria>proteobacteria>gammaproteobacteria	Klebsiella quasipneumoniae	stress-induced bacterial acidophilic repeat motif [Klebsiella quasipneumoniae]	1227438018
ASR33160.1	61	AWV60_23375	bacteria>proteobacteria>gammaproteobacteria	Klebsiella quasipneumoniae	stress-induced bacterial acidophilic repeat motif [Klebsiella quasipneumoniae]	1227441930
AST79643.1	61	CI104_11480	bacteria>proteobacteria>gammaproteobacteria	Citrobacter farmeri	stress-induced acidophilic repeat motif-containing protein [Citrobacter farmeri]	1231140424
ASV20193.1	61	B8P98_13310	bacteria>proteobacteria>gammaproteobacteria	Klebsiella quasivariicola	stress-induced acidophilic repeat motif-containing protein [Klebsiella quasivariicola]	1236388321
ASV20797.1	61	B8P98_16620	bacteria>proteobacteria>gammaproteobacteria	Klebsiella quasivariicola	stress-induced acidophilic repeat motif-containing protein [Klebsiella quasivariicola]	1236388925
ASZ58341.1	61	CKQ55_25540	bacteria>proteobacteria>gammaproteobacteria	Klebsiella michiganensis	stress-induced acidophilic repeat motif-containing protein [Klebsiella michiganensis]	1241293506
BAN97427.1	61	E05_26610	bacteria>proteobacteria>gammaproteobacteria	Plautia stali symbiont	hypothetical protein E05_26610 [Plautia stali symbiont]	549070465
CCG30128.1	61	-	bacteria>proteobacteria>gammaproteobacteria	Klebsiella aerogenes EA1509E	Conidiation-specific protein 10 [Klebsiella aerogenes EA1509E]	443902354
ACY88301.1	60	STM14_1829	bacteria>proteobacteria>gammaproteobacteria	Salmonella enterica subsp. enterica serovar Typhimurium str. 14028S	putative cytoplasmic protein [Salmonella enterica subsp. enterica serovar Typhimurium str. 14028S]	267993416
ACY88558.1	60	yciG	bacteria>proteobacteria>gammaproteobacteria	Salmonella enterica subsp. enterica serovar Typhimurium str. 14028S	putative cytoplasmic protein [Salmonella enterica subsp. enterica serovar Typhimurium str. 14028S]	267993673
ADD76921.1	60	ymdF	bacteria>proteobacteria>gammaproteobacteria	Pantoea ananatis LMG 20103	YmdF [Pantoea ananatis LMG 20103]	291152337
AFO51448.1	60	T1E_5627	bacteria>proteobacteria>gammaproteobacteria	Pseudomonas putida DOT-T1E	hypothetical protein T1E_5627 [Pseudomonas putida DOT-T1E]	397335089
AGB79467.1	60	D782_3538	bacteria>proteobacteria>gammaproteobacteria	Enterobacteriaceae bacterium strain FGI 57	stress-induced protein [Enterobacteriaceae bacterium strain FGI 57]	440048809
AGN79206.1	60	L483_17430	bacteria>proteobacteria>gammaproteobacteria	Pseudomonas putida H8234	stress-induced protein [Pseudomonas putida H8234]	511521665
AGN83819.1	60	H650_01085	bacteria>proteobacteria>gammaproteobacteria	Enterobacter sp. R4-368	hypothetical protein H650_01085 [Enterobacter sp. R4-368]	511526279

[illegible]

NP_001106364.1	324	mrps35	eukaryota>metazoa>chordata>vertebrata	Xenopus laevis	mitochondrial ribosomal protein S35 L homeolog [Xenopus laevis]
NP_001271644.1	323	MRPS35	eukaryota>metazoa>chordata>vertebrata	Macaca fascicularis	28S ribosomal protein S35, mitochondrial [Macaca fascicularis]
NP_068593.2	323	HDCMD11P,MDS023,MRP-S28,MRPS28	eukaryota>metazoa>chordata>vertebrata	Homo sapiens	28S ribosomal protein S35, mitochondrial isoform 1 precursor [Homo sapiens]
XP_003265690.1	323	MRPS35	eukaryota>metazoa>chordata>vertebrata	Nomascus leucogenys	PREDICTED: 28S ribosomal protein S35, mitochondrial isoform X1 [Nomascus leucogenys]
NP_663548.2	320	MDSO23,MRP-S28,MRPS28	eukaryota>metazoa>chordata>vertebrata	Mus musculus	28S ribosomal protein S35, mitochondrial [Mus musculus]
XP_018110000.1	317	LOC108712266	eukaryota>metazoa>chordata>vertebrata	Xenopus laevis	PREDICTED: 28S ribosomal protein S35, mitochondrial-like [Xenopus laevis]
NP_001265021.1	282	null	eukaryota>metazoa>chordata>vertebrata	Gallus gallus	28S ribosomal protein S35, mitochondrial isoform 2 [Gallus gallus]
XP_006507098.1	276	MDSO23,MRP-S28,MRPS28	eukaryota>metazoa>chordata>vertebrata	Mus musculus	PREDICTED: 28S ribosomal protein S35, mitochondrial isoform X1 [Mus musculus]
XP_006507100.1	276	MDSO23,MRP-S28,MRPS28	eukaryota>metazoa>chordata>vertebrata	Mus musculus	PREDICTED: 28S ribosomal protein S35, mitochondrial isoform X1 [Mus musculus]
XP_005570508.1	264	MRPS35	eukaryota>metazoa>chordata>vertebrata	Macaca fascicularis	PREDICTED: 28S ribosomal protein S35, mitochondrial isoform X1 [Macaca fascicularis]
XP_012357923.1	264	MRPS35	eukaryota>metazoa>chordata>vertebrata	Nomascus leucogenys	PREDICTED: 28S ribosomal protein S35, mitochondrial isoform X2 [Nomascus leucogenys]
NP_001177793.1	194	HDCMD11P,MDS023,MRP-S28,MRPS28	eukaryota>metazoa>chordata>vertebrata	Homo sapiens	28S ribosomal protein S35, mitochondrial isoform 2 precursor [Homo sapiens]
XP_017177050.1	179	MDSO23,MRP-S28,MRPS28	eukaryota>metazoa>chordata>vertebrata	Mus musculus	PREDICTED: 28S ribosomal protein S35, mitochondrial isoform X2 [Mus musculus]
XP_016875269.1	176	HDCMD11P,MDS023,MRP-S28,MRPS28	eukaryota>metazoa>chordata>vertebrata	Homo sapiens	28S ribosomal protein S35, mitochondrial isoform X1 [Homo sapiens]
XP_018098280.1	112	LOC108706169	eukaryota>metazoa>chordata>vertebrata	Xenopus laevis	PREDICTED: 28S ribosomal protein S35, mitochondrial-like, partial [Xenopus laevis]
NP_001188143.1	336	rt35	eukaryota>metazoa>chordata>vertebrata>actinopterygii	Ictalurus punctatus	28S ribosomal protein S35, mitochondrial [Ictalurus punctatus]
XP_003967536.1	333	mrps35	eukaryota>metazoa>chordata>vertebrata>actinopterygii	Takifugu rubripes	PREDICTED: 28S ribosomal protein S35, mitochondrial [Takifugu rubripes]
NP_001026839.1	330	im:6894309,zgc:114150	eukaryota>metazoa>chordata>vertebrata>actinopterygii	Danio rerio	28S ribosomal protein S35, mitochondrial precursor [Danio rerio]
CAF90759.1	267	GSTEN:00005164:G:001	eukaryota>metazoa>chordata>vertebrata>actinopterygii	Tetraodon nigroviridis	unnamed protein product [Tetraodon nigroviridis]
KXJ18051.1	318	MRPS35	eukaryota>metazoa>cnidaria	Exaiptasia pallida	28S ribosomal protein S35, mitochondrial [Exaiptasia pallida]
XP_001622247.1	313	NEMVEDRAFT_v1g220979	eukaryota>metazoa>cnidaria	Nematostella vectensis	predicted protein [Nematostella vectensis]
XP_001630476.1	230	NEMVEDRAFT_v1g210365	eukaryota>metazoa>cnidaria	Nematostella vectensis	predicted protein [Nematostella vectensis]
XP_002167644.2	173	LOC100200092	eukaryota>metazoa>cnidaria	Hydra vulgaris	PREDICTED: 28S ribosomal protein S35, mitochondrial-like, partial [Hydra vulgaris]
XP_004213366.1	120	LOC101238915	eukaryota>metazoa>cnidaria	Hydra vulgaris	PREDICTED: 28S ribosomal protein S35, mitochondrial-like [Hydra vulgaris]
XP_015780795.1	109	LOC107358718	eukaryota>metazoa>cnidaria	Acropora digitifera	PREDICTED: 28S ribosomal protein S35, mitochondrial-like [Acropora digitifera]
EFX66309.1	333	DAPPUDRAFT_302746	eukaryota>metazoa>crustacea	Daphnia pulex	hypothetical protein DAPPUDRAFT_302746 [Daphnia pulex]
EFX82761.1	220	DAPPUDRAFT_240975	eukaryota>metazoa>crustacea	Daphnia pulex	hypothetical protein DAPPUDRAFT_240975 [Daphnia pulex]
EFX74616.1	151	DAPPUDRAFT_324195	eukaryota>metazoa>crustacea	Daphnia pulex	hypothetical protein DAPPUDRAFT_324195 [Daphnia pulex]
EFX83150.1	121	DAPPUDRAFT_316038	eukaryota>metazoa>crustacea	Daphnia pulex	hypothetical protein DAPPUDRAFT_316038 [Daphnia pulex]
EFX86513.1	112	DAPPUDRAFT_44321	eukaryota>metazoa>crustacea	Daphnia pulex	hypothetical protein DAPPUDRAFT_44321, partial [Daphnia pulex]
EFX82284.1	104	DAPPUDRAFT_49266	eukaryota>metazoa>crustacea	Daphnia pulex	hypothetical protein DAPPUDRAFT_49266, partial [Daphnia pulex]
EFX65565.1	60	DAPPUDRAFT_264531	eukaryota>metazoa>crustacea	Daphnia pulex	hypothetical protein DAPPUDRAFT_264531 [Daphnia pulex]
XP_011670562.1	364	LOC591072	eukaryota>metazoa>echinodermata	Strongylocentrotus purpuratus	PREDICTED: 28S ribosomal protein S35, mitochondrial isoform X2 [Strongylocentrotus purpuratus]
XP_011670569.1	364	LOC591072	eukaryota>metazoa>echinodermata	Strongylocentrotus purpuratus	PREDICTED: 28S ribosomal protein S35, mitochondrial isoform X3 [Strongylocentrotus purpuratus]
XP_795743.4	364	LOC591072	eukaryota>metazoa>echinodermata	Strongylocentrotus purpuratus	PREDICTED: 28S ribosomal protein S35, mitochondrial isoform X1 [Strongylocentrotus purpuratus]
XP_006819385.1	272	LOC100378381	eukaryota>metazoa>hemichordata	Saccoglossus kowalevskii	PREDICTED: 28S ribosomal protein S35, mitochondrial-like [Saccoglossus kowalevskii]
XP_004922258.1	343	LOC101742763	eukaryota>metazoa>hexapoda	Bombyx mori	28S ribosomal protein S35, mitochondrial [Bombyx mori]
NP_001280291.1	334	LOC100165929	eukaryota>metazoa>hexapoda	Acyrthosiphon pisum	28S ribosomal protein S35, mitochondrial [Acyrthosiphon pisum]
XP_001846307.1	332	CpipJ_CPIJ004543	eukaryota>metazoa>hexapoda	Culex quinquefasciatus	mitochondrial ribosomal protein, S35 [Culex quinquefasciatus]
XP_019700635.1	331	LOC105191170	eukaryota>metazoa>hexapoda	Harpegnathos saltator	PREDICTED: 28S ribosomal protein S35, mitochondrial [Harpegnathos saltator]
NP_523893.1	326	CG2101,Dmel\CG2101,MRP-S28	eukaryota>metazoa>hexapoda	Drosophila melanogaster	mitochondrial ribosomal protein S35 [Drosophila melanogaster]
XP_624325.2	323	LOC551939	eukaryota>metazoa>hexapoda	Apis mellifera	PREDICTED: 28S ribosomal protein S35, mitochondrial [Apis mellifera]
XP_967727.1	320	LOC656083	eukaryota>metazoa>hexapoda	Tribolium castaneum	PREDICTED: 28S ribosomal protein S35, mitochondrial [Tribolium castaneum]
XP_009064431.1	365	LOTGIDRAFT_236049	eukaryota>metazoa>mollusca	Lottia gigantea	hypothetical protein LOTGIDRAFT_236049 [Lottia gigantea]
XP_011429850.1	350	LOC105330006	eukaryota>metazoa>mollusca	Crassostrea gigas	PREDICTED: 28S ribosomal protein S35, mitochondrial-like [Crassostrea gigas]
XP_014774526.1	296	LOC106872158	eukaryota>metazoa>mollusca	Octopus bimaculoides	PREDICTED: 28S ribosomal protein S35, mitochondrial-like, partial [Octopus bimaculoides]
XP_001896553.1	393	Bm1_25465	eukaryota>metazoa>nematoda	Brugia malayi	MRPS35 protein [Brugia malayi]
NP_001256837.1	365	mrps-35	eukaryota>metazoa>nematoda	Caenorhabditis elegans	Mitochondrial Ribosomal Protein, Small [Caenorhabditis elegans]
ERG82368.1	262	ASU_08700	eukaryota>metazoa>nematoda	Ascaris suum	28s ribosomal protein [Ascaris suum]
XP_003379553.1	227	Tsp_03211	eukaryota>metazoa>nematoda	Trichinella spiralis	conserved hypothetical protein, partial [Trichinella spiralis]
NP_001256838.1	151	mrps-35	eukaryota>metazoa>nematoda	Caenorhabditis elegans	Mitochondrial Ribosomal Protein, Small [Caenorhabditis elegans]
XP_002116924.1	211	TRIADDRAFT_31814	eukaryota>metazoa>placozoa	Trichoplax adhaerens	hypothetical protein TRIADDRAFT_31814 [Trichoplax adhaerens]
CEO98068.1	259	PBRA_006182	eukaryota>rhizaria>cercozoa>plasmodiophorida	Plasmodiophora brassicae	hypothetical protein PBRA_006182 [Plasmodiophora brassicae]
XP_014530030.1	257	JH06_0345	eukaryota>stramenopiles	Blastocystis sp. subtype 4	ribosomal protein S37 [Blastocystis sp. subtype 4]
XP_002287918.1	252	THAPSDRAFT_21363	eukaryota>stramenopiles	Thalassiosira pseudonana CCMP1335	predicted protein [Thalassiosira pseudonana CCMP1335]
XP_002178539.1	182	PHATRDRAFT_44449	eukaryota>stramenopiles	Phaeodactylum tricornutum CCAP 1055/1	predicted protein [Phaeodactylum tricornutum CCAP 1055/1]
OEU10088.1	176	FRACYDRAFT_194544	eukaryota>stramenopiles	Fragilariopsis cylindrus CCMP1102	hypothetical protein FRACYDRAFT_194544 [Fragilariopsis cylindrus CCMP1102]
XP_008868871.1	230	H310_05817	eukaryota>stramenopiles	Aphanomyces invadans	hypothetical protein H310_05817 [Aphanomyces invadans]
XP_009827115.1	227	H257_04350	eukaryota>stramenopiles	Aphanomyces astaci	hypothetical protein H257_04350 [Aphanomyces astaci]
XP_008614767.1	217	SDRG_10549	eukaryota>stramenopiles	Saprolegnia diclina VS20	hypothetical protein SDRG_10549 [Saprolegnia diclina VS20]
XP_012201816.1	217	SPRG_06963	eukaryota>stramenopiles	Saprolegnia parasitica CBS 223.65	hypothetical protein SPRG_06963 [Saprolegnia parasitica CBS 223.65]
XP_009524571.1	214	PHYSODRAFT_557720	eukaryota>stramenopiles	Phytophthora sojae	hypothetical protein PHYSODRAFT_557720 [Phytophthora sojae]
XP_015161744.1	559	LOC102598901	eukaryota>viridiplantae	Solanum tuberosum	PREDICTED: uncharacterized protein LOC102598901 [Solanum tuberosum]
NP_001328638.1	428	F18E5.80,F18E5 80	eukaryota>viridiplantae	Arabidopsis thaliana	Ribosomal protein S24/S35 [Arabidopsis thaliana]

[illegible]
